# Supplementary material for: Systematic review and network meta-analysis comparing antithrombotic agents for the prevention of stroke and major bleeding in patients with atrial fibrillation
Source: BMJ Open. 2014 Jun 2;4(6):e004301. doi: 10.1136/bmjopen-2013-004301 (PMC4054633; doi:10.1136/bmjopen-2013-004301)
Supplement: Author's manuscript [file bmjopen-2013-004301.draft_revisions.pdf]

Systematic Review and Network Meta-analysis comparing Antithrombotic Agents for the Prevention of Stroke and Major Bleeding in Patients with Atrial Fibrillation

Journal:	*BMJ Open*
Manuscript ID:	bmjopen-2013-004301
Article Type:	Research
Date Submitted by the Author:	21-Oct-2013
Complete List of Authors:	Cameron, Chris; University of Ottawa, Department of Community Medicine and Epidemiology<br>Coyle, Douglas; University of Ottawa, Epidemiology and Community Medicine<br>Richter, Trevor; Canadian Agency for Drugs and Technologies in Health,<br>Kelly, Shannon; Ottawa Hospital Research Institute, Medicine<br>Gauthier, Kasandra; Canadian Agency for Drugs and Technologies in Health,<br>Steiner, Sabine; Medical University of Vienna, Department of Internal Medicine/Cardiology<br>Carrier, Marc; Ottawa Hospital Research Institute, Medicine<br>Coyle, Kathryn; Applied Health Economics Research Unit,<br>Bai, Annie; Canadian Agency for Drugs and Technologies in Health,<br>Moulton, Kristen; Canadian Agency for Drugs and Technologies in Health,<br>Clifford, Tammy; University of Ottawa, Department of Epidemiology and Community Medicine; University of Ottawa, Department of Pediatrics<br>Wells, George ; University of Ottawa, Department of Epidemiology and Community Medicine; University of Ottawa Heart Institute,
**Primary Subject Heading**:	Cardiovascular medicine
Secondary Subject Heading:	Epidemiology, Health policy
Keywords:	Stroke < NEUROLOGY, CARDIOLOGY, EPIDEMIOLOGY

SCHOLARONE™
Manuscripts

Systematic Review and Network Meta-analysis comparing Antithrombotic Agents for the Prevention of Stroke and Major Bleeding in Patients with Atrial Fibrillation

Chris Cameron, PhD Student[1,2], Doug Coyle, Professor[1], Trevor Richter, Manager of Clinical Research – Health Economics[3], Shannon Kelly, Research coordinator[2], Kasandra Gauthier, Clinical Research Officer[3], Sabine Steiner, Associate Professor[4], Marc Carrier, Associate professor[6], Kathryn Coyle, Health Economist[5], Kristen Moulton, Clinical Research Officer[3], Annie Bai , Clinical Research Officer[3], Tammy Clifford, Adjunct Professor[1,7], George Wells, Professor[1,2]

[1] Department of Community Medicine and Epidemiology, University of Ottawa, Canada
[2] University of Ottawa Heart Institute, Ottawa, Canada
[3] Canadian Agency for Drugs and Technologies in Health, Ottawa, Canada
[4] Medical University of Vienna, Vienna, Austria
[5] Applied Health Economics Research Unit, Ottawa, Canada
[6] Thrombosis Program, Division of Hematology, Department of Medicine, University of Ottawa, Ottawa, Canada
[7] Department of Pediatrics, University of Ottawa, Ottawa, Canada

Word count, abstract: 300
Word count, body of essay: 3,987
Number of figures: 4
Number of tables: 1
Number of References: 36
Supplementary Material: 12

Corresponding author:
Dr. George Wells
Professor and Director, Cardiovascular Research Methods Centre
Department of Community Medicine and Epidemiology, University of Ottawa
Ottawa Heart Institute
40 Ruskin Street, Ottawa, Ontario, Canada K1Y 4W7
Phone: (613) 798-5555, ext. 18640
Fax: (613) 761-4412
Email: gawells@ottawaheart.ca

Keywords: atrial fibrillation, antithrombotic, anticoagulants, stroke, bleeding

For peer review only

Abstract

Objective: To compare the efficacy and safety of antithrombotic treatments (dabigatran, rivaroxaban, apixaban, and vitamin K antagonists (VKA) at a standard adjusted dose (target international normalised ratio 2.0-3.0), acetylsalicylic acid, acetylsalicylic acid and clopidogrel) for non-valvular atrial fibrillation and among sub-populations.

Design: Systematic review and network meta-analysis.

Data sources: A systematic literature search strategy was designed and carried out using MEDLINE, EMBASE, Cochrane Register of Controlled Trials, and grey literature including the websites of regulatory agencies and health technology assessment organizations.

Eligibility criteria for selecting studies: Randomized controlled trials were selected for inclusion if they were published in English, included at least one antithrombotic treatment and involved patients with non-valvular atrial fibrillation eligible to receive anticoagulant therapy.

Results: For stroke or systemic embolism, apixaban and dabigatran 150 mg twice daily were associated with reductions relative to standard adjusted dose VKA whereas low-dose ASA and the combination of clopidogrel plus low-dose ASA were associated with increases. Absolute risk reductions ranged from 6 fewer events per 1,000 patients treated for dabigatran 150 mg twice daily to 14 more events for clopidogrel plus ASA. For major bleeding, apixaban and dabigatran 110 mg twice daily were associated with reductions compared to standard adjusted dose VKA. Absolute risk reductions with these agents ranged from 21 fewer per 1,000 patients treated each year for no treatment/placebo to 23 more for medium dose ASA.

Conclusions: Compared with standard adjusted dose VKA, new oral anticoagulants were associated with modest reductions in the absolute risk of stroke and major bleeding. People on antiplatelet drugs experienced more strokes compared with anticoagulant drugs without any reduction in bleeding risk. To fully elucidate the comparative benefits and harms of antithrombotic agents across the various sub-populations, rigorously conducted comparative studies or network meta-regression analyses of patient-level data are required.

(PROSPERO registry - CRD42012002721).

For peer review only

Article Summary

Strengths and limitations of this study

- The studies pooled in this meta-analysis include antiplatelet agents in addition to newer oral anticoagulants and standard adjusted dose vitamin K antagonists
- We report detailed *a priori* sub-group analyses for stroke or systemic embolism and major bleeding using data derived from FDA Public Summary Documents
- We present findings on both the relative and absolute scale, and graphically illustrate absolute risks using icon arrays
- Comparisons between antithrombotic treatments were largely comprised of single studies, albeit large studies
- There is notable heterogeneity and the small number of studies limits the analyses that can be conducted to account for heterogeneity in the absence of patient-level data

For peer review only

For peer review only - http://bmjopen.bmj.com/site/about/guidelines.xhtml

INTRODUCTION

Atrial fibrillation (AF) is a common cardiac arrhythmia associated with increased morbidity and mortality.[1,2] Patients with AF sustain an increased risk of arterial thromboembolism and stroke, associated with high recurrence and substantial debilitating impact. Therefore, antithrombotic strategies using anticoagulant drugs and antiplatelet agents are recommended for AF patients presenting with risk factors for stroke. Antithrombotic therapy is also associated with a risk of bleeding; therefore, the beneficial effects on stroke prevention should always be compared against a patient's risk of major bleeding.[1,2]

Existing guidelines recommend anticoagulant therapy for patients at intermediate or high risk of stroke.[1,2] Although standard adjusted dose vitamin K antagonists (VKA) (e.g., warfarin) has been the cornerstone treatment for reducing the risk of stroke or systemic embolism (SE) in this population, it is associated with several drawbacks such as significant inter- and intra-patient variability in dose response; need for laboratory monitoring; increased risk of bleeding complications; and several food and drug interactions.[2] These limitations have prompted the development of newer oral anticoagulants such as direct thrombin (dabigatran) and Xa (e.g. apixaban, rivaroxaban) inhibitors. [1,2] While some of these newer treatment options have been demonstrated to be efficacious in preventing stroke or SE compared to standard adjusted dose VKA, the relative efficacy and risk of bleeding of newer oral anticoagulants, both among themselves and in comparison to antiplatelet agents, is not clear, especially in certain sub-populations of patients, e.g., $CHADS_2$ score <2 or ≥2; time in therapeutic range (TTR) < 66% or ≥ 66%; Age <75 or ≥ 75 years. Therefore, the objective of this paper was to compare antithrombotic agents for the prevention of stroke and major bleeding in patients with non-valvular AF and among sub-populations.

For peer review only - http://bmjopen.bmj.com/site/about/guidelines.xhtml

METHODS

Published literature was identified by searching MEDLINE, MEDLINE In-Process & Other Non-Indexed Citations, EMBASE, BIOSIS Previews, PubMed and the Cochrane Central Register of Controlled Trials were searched through the Ovid interface to identify English-language clinical articles published from 1988 to June 7, 2012. Regular alerts were also established. Websites of regulatory agencies, Canadian and major international health technology assessment agencies, clinical practice guidelines as well as The Cochrane Library (2012, Issue 6) and University of York Centre for Reviews and Dissemination databases were searched. Complete details of the electronic search strategy, including any limits used, are reported in Appendix 1. The protocol was published online and was registered in the PROSPERO International Prospective Register of Systematic Reviews (PROSPERO registry - CRD42012002721).

The population of interest was individuals with non-valvular AF requiring anticoagulation (including all risk levels and regardless of any comorbidities). The following treatments were included in the review: apixaban, dabigatran, rivaroxaban, standard adjusted dose VKA, acetylsalicylic acid (ASA), ASA and clopidogrel. Numerous outcomes were considered and results are reported elsewhere; however, the focus of this publication is the outcomes of all-cause stroke or SE and major bleeding as defined by the International Society on Thrombosis and Haemostasis [ISTH] definition),[3] which were the primary efficacy and safety outcomes used in the newer oral anticoagulant studies.

For peer review only - http://bmjopen.bmj.com/site/about/guidelines.xhtml

Active and placebo-controlled randomized controlled trials (RCTs) were selected for inclusion if they were published in English, included at least one antithrombotic treatment under review, reported any of the pre-specified outcomes related to patient safety or clinical efficacy, and involved patients with non-valvular AF eligible to receive anticoagulant therapy, regardless the level of stroke risk. Trials that included patients with contraindication to anticoagulant treatment were excluded. VKA trials were included if the dose was adjusted to a target international normalised ratio (INR) 2.0-3.0. Any dose of ASA was considered for inclusion, but ASA dose was stratified in the analysis as low, medium, or high. Three reviewers independently extracted the data on baseline characteristics, intervention(s) evaluated, including dose, duration, and relevant co-medication and results for each included article, using a standardized template. All extracted data was checked for accuracy by three independent reviewers. Any disagreements in the assessment of these data were resolved through discussion until consensus was reached. Quality assessment of RCTs was also performed independently by two reviewers using a standardized table based on major items from the SIGN 50 instrument for internal validity.[4] The trial selection process is presented in a flowchart based on the Preferred Reporting Items for Systematic Reviews and Meta-Analyses (PRISMA)[5] statement (Appendix 2).

Bayesian network meta-analyses were conducted using WinBUGS software (MRC Biostatistics Unit, Cambridge, UK). A binomial likelihood model[6] which allows for the use of multi-arm trials was used for the analyses given the datasets provided were dichotomous outcomes and included multi-arm trials. Small trials (<100 patients in each arm) with zero cells in both arms or nodes where there were no events were excluded from the evidence networks because they do

For peer review only - http://bmjopen.bmj.com/site/about/guidelines.xhtml

not contribute information or allow interpretable information.[6] Both fixed and random-effects network meta-analyses were conducted, although the fixed-effects model was chosen for the reference case analysis, as the evidence network was largely comprised of single study connections. We modeled point estimates and 95% credible intervals for odds ratios (OR) using Markov Chain Monte Carlo methods. The absolute risk difference per 1000 patients treated each year for each outcome was also calculated, based on the standard adjusted dose VKA arm of the Randomized Evaluation of Long-Term Anti-coagulation Therapy (RE-LY) trial.[7] The RE-LY trial[7] was selected because it was the most recent trial which contained data for both $CHADS_2<2$ and $CHADS_2 \geq 2$ and had detailed data available from the Food and Drug Administration (FDA) Public Summary Report.[8] We also assessed the probability that each treatment was the most efficacious regimen, the second best, the third best, and so on.[9] Vague or flat priors, such as $N(0, 100^2)$ were assigned for basic parameters throughout, although informative priors were considered.[10] Assessment of model fit was based on deviance information criterion (DIC) and comparison of residual deviance to number of unconstrained data points.[6,11] To ensure convergence was reached, trace plots and the Brooks-Gelman-Rubin statistic were assessed.[12] Three chains were fit in WinBUGS for each analysis, with at least 40,000 iterations, and a burn-in of at least 40,000 iterations. We also qualitatively compared the results from our network meta-analysis with direct pair-wise estimates. Frequentist pair-wise meta-analyses were conducted using R (R Language and Environment for Statistical Computing, Vienna, Austria) and package meta.[13]

A key assumption behind network meta-analysis is that the analysed network is consistent, i.e., there is no conflict between direct and indirect evidence.[14] To assess inconsistency, we compared

For peer review only - http://bmjopen.bmj.com/site/about/guidelines.xhtml

deviance and DIC statistics in fitted consistency and inconsistency models.[14] We also plotted the posterior mean deviance of the individual data points in the inconsistency model against their posterior mean deviance in the consistency model to identify any loops in where inconsistency is present (Appendix 11).[14] Additionally, the results from our network meta-analysis were qualitatively compared with direct frequentist pair-wise estimates.

Network meta-analysis also requires that studies are sufficiently similar in order to pool their results.[15] Available study and patient characteristics were assessed to ensure similarity and to investigate the potential impact of heterogeneity on effect estimates. Clinical and methodological heterogeneity was identified in a number of areas. The issues identified were similar to those reported in previous systematic reviews of anticoagulant drugs (e.g., differences in TTR, $CHADS_2$ score).[16] Heterogeneity was assessed by conducting network meta-analysis with the following pre-specified subgroup data reported in the individual RCTs: TTR $< 66\%$ or $\geq 66\%$; $CHADS_2$ score <2 or ≥ 2; Age <75 or ≥ 75 years.[16] The data to conduct these sub-group analyses were derived from FDA Public Summary Documents when not available in the publications.[8,17,18] Secondary evidence networks were constructed for each of the sub-group analyses. The WinBUGS code[6] and data needed to replicate all analyses are available online.

This research was supported through a grant by the Canadian Institute of Health Research (CIHR) Drug Safety and Effectiveness Network and financial support from the Canadian Agency for Drugs and Technologies in Health (CADTH).

For peer review only - http://bmjopen.bmj.com/site/about/guidelines.xhtml

RESULTS

Study Characteristics of included studies

The systematic review included 12 individual RCTs (reported in 28 publications and FDA reports); all evaluated the efficacy and safety of antithrombotic agents: (dabigatran (150 or 110 mg twice daily), rivaroxaban (20 mg daily) and apixaban (5 mg twice daily)), standard adjusted dose VKA, ASA (low dose (< 100 mg daily); medium dose (100-300 mg daily), or low dose-ASA plus clopidogrel (75 mg daily) in patients with non-valvular AF. None of the included studies directly compared one new oral anticoagulant drug with another (Appendix 4). Of the 61,050 randomized patients included in this review, four large multicentre trials account for 57,284 patients (94%): ARISTOTLE (n=18,201),[19] RE-LY (n=18,113),[7] ROCKET-AF (n=14,264)[20] and ACTIVE-W (n=6706).[21] Most trials included stroke and SE as a primary efficacy outcome, while bleeding events were a frequent safety outcome. Follow-up ranged from 12 weeks to 3.5 years. All trials published results between 1989 and 2011. Mean $CHADS_2$ score was reported in five trials, encompassing the vast majority of patients included in the systematic review. Reported $CHADS_2$ scores were consistent with a high-risk population ($CHADS_2 \geq 2$), with patients from ROCKET-AF[20] showing the highest risk for stroke (mean $CHADS_2$ = 3.5).

Mean age across the included trials ranged from 65 years to 83 years (Appendix 3). All trials included patients of both gender and had a higher proportion of male participants in most studies. However, the proportions of patients with a prior stroke or TIA varied substantially across the included trials, ranging from 3% in JAST to 55% in ROCKET-AF.[20] The same observation can be made regarding the proportions of patients with other concomitant conditions, i.e. congestive heart failure, hypertension, diabetes infarction, and prior vitamin K antagonist experience. TTR

For peer review only - http://bmjopen.bmj.com/site/about/guidelines.xhtml

was reported in all but one trial that included a standard adjusted dose VKA treatment arm. Patient baseline characteristics are summarized in Appendix 3.

The studies were individually critically appraised and the details are reported elsewhere. Overall, there was substantial variation in study quality. However, large multicentre trials comparing newer anticoagulants with standard adjusted dose VKA such as ARISTOTLE (n = 18,201), RE-LY (n = 18,113), and ROCKET-AF (n = 14,264), which account for the vast majority of patients included in the systematic review, appear to be methodologically rigorous.

Stroke or Systemic Embolism

The evidence network (Figure 1) for the primary analysis was comprised of nine RCTs representing eight treatments in addition to placebo/observation (N = 60,424). Three RCTs (PETRO,[22] ARISTOTLE J,[23] WASPO[24]) were not included in the analysis because they had with zeroes in both arms or treatments where there were no events (e.g., Apixaban 2.5 mg).

Apixaban and dabigatran (150 mg twice daily) were associated with reductions in stroke or SE relative to standard adjusted dose VKA (Figure 2). The use of these two agents led to absolute risk reductions ranging from 4 to 6 fewer events per 1,000 patients treated each year. By contrast, low-dose ASA and the combination of clopidogrel plus low-dose ASA appeared to have a higher risk of stroke or SE than standard adjusted dose VKA, leading to an increase in the number of stroke or SE ranging from 14 to 15 more events per 1,000 patients treated each year (Figure 4 and Appendix 6). No differences were detected between standard adjusted dose VKA

For peer review only - http://bmjopen.bmj.com/site/about/guidelines.xhtml

and each of the following interventions: rivaroxaban, dabigatran (110 mg twice daily), medium-dose ASA, and no treatment/placebo (Figure 3).

The estimates of effect derived from the direct pair-wise comparisons aligned well with those obtained from the network meta-analysis in both direction and magnitude (Appendix 6). Furthermore, the posterior mean residual deviance (19.28) is close to the number of unconstrained data points (19), which is an indication of reasonable model fit. A number of sub-group analyses and alternative modeling strategies were conducted on the primary analysis, including adjusting for $CHADS_2$, TTR, and age (Table 1). We also conducted a sensitivity analysis where we included the AVERROES[25] and ACTIVE A[26] trial (Appendix 12). These studies were excluded from the main analysis because we required patients to be eligible for anticoagulant therapy, including treatment with a VKA. The results differed slightly from the reference case (Appendix 12). The primary analysis was conducted using a fixed-effects model because the network was comprised of single study connections; these results were also compared against those obtained using a random-effects model, and found to be similar in magnitude although wider credible intervals were observed (Appendix 9).

Major bleeding

The evidence network for the primary analysis for major bleeding was comprised of 10 RCTs representing eight treatments in addition to placebo/observation (N =60,503). The evidence network for major bleeding is similar to the evidence network for stroke or SE (Figure 1) but includes one extra RCT (WASPO[24]) comparing medium dose ASA with standard adjusted dose VKA (Appendix 5).

For peer review only - http://bmjopen.bmj.com/site/about/guidelines.xhtml

Apixaban and dabigatran 110 mg twice daily were associated with reductions in the risk of major bleeding compared with standard adjusted dose VKA (Figure 2). No differences for major bleeding were detected between standard adjusted dose VKA and each of the remaining interventions: rivaroxaban, dabigatran 150 mg twice daily, clopidogrel plus low-dose ASA, and all ASA dosages (Figure 2). The absolute risk difference of major bleeding relative to standard adjusted dose VKA ranged from 22 fewer to 24 more events per 1000 patients treated per year **(**Figure 4 and Appendix 7**)**. A summary of the results for the Bayesian and direct pair-wise meta-analyses is shown in Appendix 7, and Bayesian network meta-analysis results are represented graphically in Figure 2. Complete results from the Bayesian network meta-analysis for all possible comparisons are presented in Figure 3**.**

The estimates of effect derived from the direct pair-wise comparisons aligned well with those obtained from the network meta-analysis in both direction and magnitude (Appendix 7). Furthermore, the posterior mean residual deviance (22.1) is close to the number of unconstrained data points (22), which is an indication of reasonable model fit. A number of sub-group analyses and alternative modeling strategies were conducted on the primary analysis (Table 1). We also conducted a sensitivity analysis where we included the AVERROES[25] and ACTIVE A[26] trial (Appendix 12), as well as analyses using a random-effects model with vague and informative priors (Appendix 9).

Benefit-Harm Assessment – Stroke or systemic embolism versus major bleeding

For peer review only - http://bmjopen.bmj.com/site/about/guidelines.xhtml

Figure 3 summarises the results for all pair-wise comparisons derived from the Bayesian fixed-effects network meta-analysis. Results relative to standard adjusted dose VKA have been discussed above. For pair-wise comparisons among newer oral anticoagulants for stroke or SE, dabigatran 150 mg twice daily was associated fewer events versus dabigatran 110 mg twice daily and rivaroxaban. No other differences were observed between the new anticoagulant agents. There were no differences among low-dose ASA, medium-dose ASA, and clopidogrel plus low-dose ASA. However, low-dose ASA and the combination of clopidogrel plus low-dose ASA were associated with increases in stroke or systemic embolism compared to all anticoagulants, including standard adjusted dose VKA, dabigatran (both 150 and 110 mg twice daily), apixaban, and rivaroxaban. For major bleeding, apixaban and dabigatran 110 mg twice daily were associated with fewer events versus dabigatran 150 mg twice daily and rivaroxaban. The risk of major bleeding for apixaban was lower compared to clopidogrel plus low-dose ASA. There were no differences associated with the ASA treatments, both for comparisons among themselves and compared to the anticoagulant treatments.

Figure 4 plots the absolute risk difference for stroke or SE versus major bleeding per 1000 treated for the 8 treatments analyzed, each relative to standard adjusted dose VKA. Data in Figure 4 were obtained from the results presented in Appendix 6 and 7. Examination of the data in Figure 4 suggests that the balance of benefits and harms of the new oral anticoagulants for stroke or systemic embolism and major bleeding is positive compared to standard adjusted dose VKA (decrease in stroke or systemic embolism and/or major bleeding) and largely similar among one another. Comparison of all anticoagulant treatment (including standard adjusted dose VKA) to ASA, or ASA in combination with clopidogrel indicates that whereas the new oral anticoagulants decrease the risk of both stroke or systemic embolism and major bleeding, ASA

For peer review only - http://bmjopen.bmj.com/site/about/guidelines.xhtml

For peer review only

has a less favorable balance of benefits and harms, i.e., fails to minimize the risk of stroke or systemic embolism and/or major bleeding.

DISCUSSION

We identified 12 RCTs comparing antithrombotic agents for the prevention of all cause stroke or systemic embolism and major bleeding in patients with AF. To the best of our knowledge, this is the most comprehensive systematic review and network meta-analysis[27] to synthesize the available benefits and harms on newer oral anticoagulants, standard adjusted dose VKA, and antiplatelet agents for the prevention of stroke and major bleeding in patients with non-valvular AF. Harenberg et al[27] recently summarized the published indirect comparisons and network meta-analyses evaluating newer oral anticoagulants for AF and an earlier unpublished version of our analysis[16] was considered among the most comprehensive analyses at that time.[27] This network meta-analysis expands upon that unpublished report.[16] We include antiplatelet agents in addition to newer oral anticoagulants and standard adjusted dose VKA; present detailed evidence networks[28] to illustrate the body of evidence for each outcome; report detailed *a priori* sub-group analyses for each outcome using data derived from FDA Public Summary Documents;[8,17] present findings on both the relative and absolute scale,[29,30] and graphically illustrate results using icon arrays. Our systematic review and network meta-analysis is also the only one published to date that was registered in PROSPERO, and adheres to PRISMA reporting standards (Appendix 10).

For stroke or systemic embolism, apixaban and dabigatran 150 mg twice daily were associated with reductions in stroke or systemic embolism relative to standard adjusted dose VKA. By contrast, low-dose ASA and the combination of clopidogrel plus low-dose ASA appeared were

For peer review only - http://bmjopen.bmj.com/site/about/guidelines.xhtml

associated with increases in stroke or systemic embolism relative to standard adjusted dose VKA. No differences were detected between standard adjusted dose VKA and each of the following interventions: rivaroxaban, dabigatran 110 mg twice daily, medium-dose ASA, and no treatment/placebo. The absolute risk reduction of stroke or systemic embolism relative to standard adjusted dose VKA ranged from 6 fewer to 15 more events per 1000 patients treated per year, with dabigatran 150 mg twice daily yielding the largest aboslute risk reduction and ASA 100 mg daily yielding clopidogrel plus low-dose ASA the largest absolute risk increase.

For major bleeding, apixaban and dabigatran 110 mg twice daily were associated with in the risk of major bleeding relative to standard adjusted dose VKA. No differences for major bleeding were detected between standard adjusted dose VKA and each of the remaining interventions: rivaroxaban, dabigatran 150 mg twice daily, clopidogrel plus low-dose ASA, and all ASA dosages. The absolute risk difference of major bleeding relative to standard adjusted dose VKA ranged from 22 fewer to 23 more events per 1000 patients treated, with placebo/no treatment yielding the largest absolute risk reduction and medium dose ASA the largest absolute risk increase. Results of network meta-analyses reflect the relative efficacy and safety profile of antiplatelet and anticoagulant drugs as documented by large clinical trials. Thus, international guidelines[1,2] for prevention of stroke in patients with AF also recommended oral anticoagulation over antiplatelet therapy as the latter had only modest protective effects. With respect to the new oral anticoagulants none of the guidelines has given a recommendation to prefer one of the new oral anticoagulants over another.[1,2]

For peer review only - http://bmjopen.bmj.com/site/about/guidelines.xhtml

Network meta-analysis involves pooling of trials. We observed variability across studies included in the evidence network in terms of $CHADS_2$, age and TTR (Appendix 3 and 4). For example, patients using rivaroxaban had a higher risk of stroke because ROCKET-AF[20] included patients with $CHADS_2 \geq 2$. As such, we conducted sub-group analysis to adjust for differences in patient and study-level characteristics. We derived data for sub-group analyses from FDA Public Summary documents[8,17,18] which enhanced our ability to account for variability across studies (e.g., use intention to treat (ITT) population for rivaroxaban versus per-protocol as-treated population reported in publication[20]). Results from these sub-group analyses differed slightly from those reported in the primary analysis, indicating that there may be differences in the benefits and harms across sub-groups. This becomes more apparent when sub-group analyses are reported on the absolute scale (reported elsewhere). It is unclear whether the absolute risk reductions associated with these differences translate into clinically meaningful benefits in practice, especially in sub-populations of patients with a lower baseline risk of stroke or systemic embolism. To fully elucidate the comparative benefits and harms of antithrombotic agents across the various sub-populations, rigorously conducted comparative studies,[31,32] or network meta-regression analyses of patient-level data[33] are required.

There are several limitations with the analysis we employed. First, there is insufficient sub-group data available for other outcomes reported in the RCTs included. As a consequence, detailed analysis was limited to two outcomes – stroke and SE and major bleeding. Second, we reported the results from the fixed-effects model in the main text. We felt that this was appropriate as the nodes in evidence networks are connected largely by single studies. Effect estimates derived from the fixed-effects model aligned more closely with the direct estimates. Reporting results

For peer review only - http://bmjopen.bmj.com/site/about/guidelines.xhtml

from the fixed-effects model in the main text likely biases results in favour of the new oral anticoagulants, as treatments that achieved statistically significant results in the primary RCTs retained statistically significant findings when using the fixed-effects model, but not always when using the random-effects model due to the prior[10] on the between study variance. Nevertheless, the results from the random-effects model have also been reported in Appendix 9, along with detailed discussing around the limitations in using the fixed-effects model. Third, the absolute risk reduction for the network meta-analysis was calculated using the event rate in the standard adjusted dose VKA arm of the RE-LY study. The RE-LY trial was selected because it was the most recent trial which contained data for both $CHADS_2 < 2$ and $CHADS_2 \geq 2$ and had detailed data made publicly available following an FDA review of dabigatran. Use of a different study (e.g., ARISTOTLE) for baseline event rate data is unlikely to change the results substantially given that the event rates in standard adjusted dose VKA arms were similar across studies. Fourth, we present findings using graphics that have been largely applied in the network meta-analysis literature,[34] such as a table with odds ratios for all pair-wise comparisons. This method of presenting findings on the relative scale is problematic when you are conducting assessments to compare the balance of benefits and harms, which need to be standardized. As such, we also provided results in absolute terms. We report our findings using icon arrays, although it should be noted that these results do not account for the utility values based upon patient preferences for each of the outcomes,[35,36] nor do they reflect uncertainty although we report absolute reductions on the risk benefit plane reflecting uncertainty in Appendix 8. There are other methods available that can incorporate patient preferences for outcomes.[35,36] Further research is needed comparing the balance of benefit and harms using other research

For peer review only - http://bmjopen.bmj.com/site/about/guidelines.xhtml

methodologies, and incorporating other relevant outcomes, and patient preferences for each of the outcomes.[35,36]

CONCLUSIONS

Compared with standard adjusted dose VKA, apixaban and dabigatran 150 mg twice daily were associated with reductions in the incidence of stroke or systemic embolism in patients with AF, whereas, low-dose ASA and the combination of clopidogrel plus low-dose ASA increased the risk relative to standard adjusted dose VKA at preventing stroke or systemic embolism. Apixaban, and Dabigatran 110 mg twice daily was associated with a reduced risk in major bleeding compared with standard adjusted dose VKA. Although the results of the current review revealed that there were differences in stroke or systemic embolism and major bleeding among antithrombotic agents in the management of patients with AF in relative terms, it is unclear whether the absolute risk reductions associated with these differences translate into clinically meaningful benefits in practice, especially in sub-populations of patients with a lower baseline risk of stroke or systemic embolism. Rigorously conducted comparative studies or network meta-regression analyses of patient-level data are required to fully elucidate the comparative benefits and harms of antithrombotic agents across the various sub-populations.

For peer review only

For peer review only - http://bmjopen.bmj.com/site/about/guidelines.xhtml

TABLES

Table 1: Sub-group and sensitivity analyses for stroke or systemic embolism and major bleeding relative to standard adjusted dose VKA, Odds ratios ± 95% Credible Intervals versus standard adjusted dose VKA

Treatment	Reference Case – Fixed Effect NMA	Subgroup analysis by $CHADS_2$ Score		Subgroup analysis by Age		Subgroup analysis by TTR	
		$CHADS_2$ < 2	$CHADS_2$ ≥ 2	Age < 75 years	Age ≥ 75 years	TTR < 66%	TTR ≥ 66%
**Stroke or Systemic Embolism**							
**Apixaban 5 mg twice daily**	**0.79 (0.66,0.95)**	0.86 (0.57,1.28)	**0.78 (0.63,0.95)**	0.85 (0.67,1.07)	**0.71 (0.53,0.95)**	0.79 (0.62,1.01)	0.79 (0.59,1.05)
**Dabigatran 110 mg twice daily**	0.91 (0.74,1.12)	1 (0.64,1.55)	0.89 (0.71,1.12)	0.93 (0.7,1.23)	0.88 (0.66,1.18)	0.91 (0.69,1.19)	0.9 (0.66,1.24)
**Dabigatran 150 mg twice daily**	**0.66 (0.53,0.82)**	0.61 (0.37,1)	**0.66 (0.52,0.85)**	0.63 (0.46,0.87)	**0.66 (0.48,0.9)**	0.54 (0.39,0.73)	0.81 (0.58,1.11)
**Rivaroxaban 20 mg daily**	0.88 (0.74,1.03)	NA	0.88 (0.74,1.04)	0.95 (0.75,1.2)	0.8 (0.63,1.02)	0.81 (0.65,1.01)	0.72 (0.47,1.1)
**No treatment/Placebo**	1.53 (0.9,2.63)	1.88 (0.69,5.74)	NA	NA	NA	NA	NA
**Low Dose ASA (≤ 100 mg daily)**	**1.87 (1.27,2.8)**	2.2 (1.18,4.25)	2.1 (0.91,5.15) *	NA	2.04 (1.32,3.19)	NA	NA
**Medium Dose ASA (> 100 mg and ≤ 300 mg daily)**	1.35 (0.75,2.46)	1.97 (0.63,6.73) **	NA	NA	NA	NA	NA
**Clopidogrel 75 mg daily & Low dose Aspirin (≤ 100 mg daily)**	1.93 (1.42,2.65)	3.16 (1.39,8.22)	1.15 (0.84,1.57)	NA	NA	1.52 (0.96,2.45)	**2.4 (1.59,3.71)**
**Major Bleeding**							
**Apixaban 5 mg twice daily**	**0.69 (0.6,0.8)**	**0.59 (0.44,0.78)**	**0.73 (0.62,0.87)**	**0.73 (0.6,0.89)**	**0.65 (0.52,0.81)**	**0.57 (0.45,0.71)**	**0.81 (0.67,0.98)**
**Dabigatran 110 mg twice daily**	**0.8 (0.69,0.93)**	**0.65 (0.47,0.89)**	0.86 (0.73,1.02)	**0.61 (0.49,0.77)**	1.02 (0.84,1.25)	0.74 (0.61,0.91)	0.86 (0.69,1.06)
**Dabigatran 150 mg twice daily**	0.93 (0.8,1.08)	0.77 (0.57,1.04)	1.01 (0.86,1.19)	**0.7 (0.56,0.86)**	1.19 (0.98,1.45)	0.76 (0.62,0.94)	1.15 (0.94,1.4)
**Rivaroxaban 20 mg daily**	1.03 (0.89,1.19)	NA	1.03 (0.89,1.19)	0.93 (0.76,1.13)	1.15 (0.93,1.41)	0.92 (0.77,1.1)	**1.3 (1.01,1.69)**
**No treatment/Placebo**	0.33 (0.08,1.08)	0.35 (0.04,1.75)	NA	NA	NA	NA	NA

For peer review only - http://bmjopen.bmj.com/site/about/guidelines.xhtml

Treatment	Reference Case – Fixed Effect NMA	Subgroup analysis by $CHADS_2$ Score		Subgroup analysis by Age		Subgroup analysis by TTR	
		$CHADS_2$ < 2	$CHADS_2$ ≥ 2	Age < 75 years	Age ≥ 75 years	TTR < 66%	TTR ≥ 66%
**Low Dose ASA (≤ 100 mg daily)**	1.05 (0.6,1.86)	0.83 (0.42,1.64)	1.6 (0.55,5.02) *	NA	1.01 (0.57,1.79)	NA	NA
**Medium Dose ASA (> 100 mg and ≤ 300 mg daily)**	1.78 (0.62,5.59)	1.48 (0.11,19.07)**	NA	NA	NA	NA	NA
**Clopidogrel 75 mg daily & Low dose Aspirin (≤ 100 mg daily)**	1.1 (0.83,1.46)	1.51 (0.89,2.61)	0.97 (0.69,1.36) †	NA	NA	0.66 (0.42,1.02)	1.66 (1.12,2.47)

ASA= acetylsalicylic acid; mg= milligram; NA=not available; NMA= network meta-analysis; OR=odds ratio; TTR= time in therapeutic range; VKA=vitamin K antagonist
* Results may be slightly biased against LDASA. Derived from BAFTA study where $CHADS_2$ subgroup data stratified by $CHADS_2$ 1-2 (vs. 0-1) and $CHADS_2$ 3-6 (vs. 2-6).
** Results may be slightly biased against medium-dose ASA. Based on study level results from CAFA and JAST studies. Although these studies consisted primarily of low-risk populations, some patients may have $CHADS_2$ scores greater than 2.
† Estimated from subgroup study by Healey et al 2008

For peer review only - http://bmjopen.bmj.com/site/about/guidelines.xhtml

FIGURES

Figure 1: Evidence network for all cause stroke or systemic embolism. The width of the lines is proportional to the number of randomized controlled trials comparing each pair of treatments, and the size of each treatment node is proportional to the number of randomised participants (sample size). A dotted line indicates a three-arm randomized controlled trial

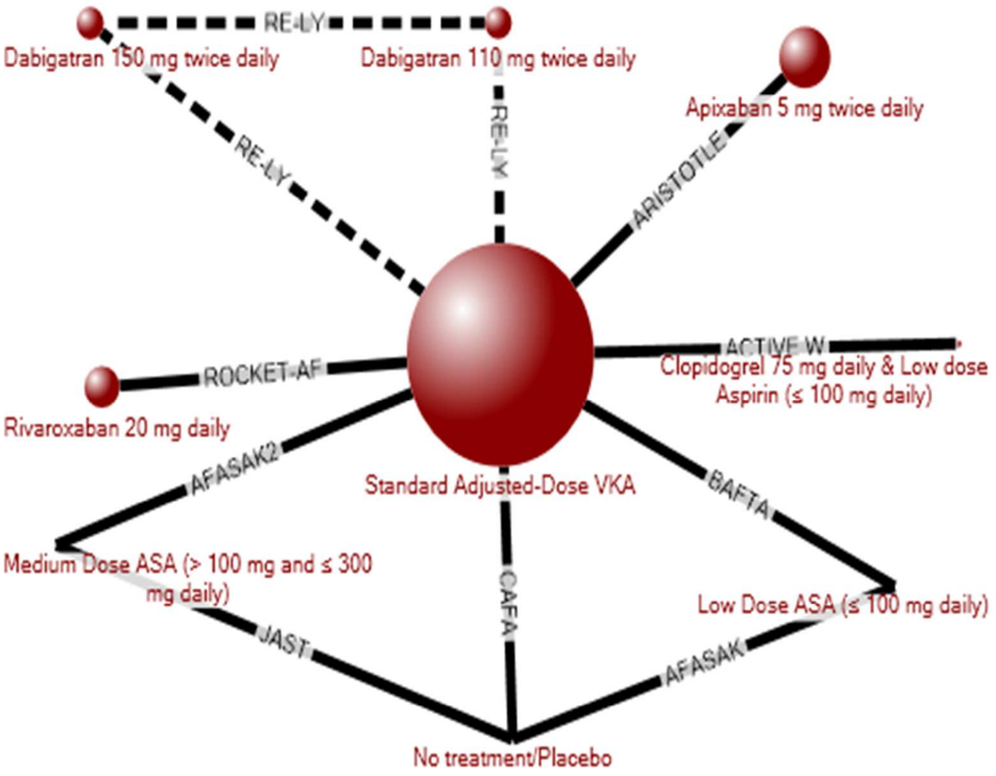

ASA= acetylsalicylic acid; mg= milligram; RCT= RCT = randomized controlled trial; VKA= vitamin K antagonist

only

For peer review only - http://bmjopen.bmj.com/site/about/guidelines.xhtml

Figure 2: Odds ratio for all cause stroke or systemic embolism (A) and major bleeding (B) in Bayesian network meta-analysis versus standard adjusted dose VKA.

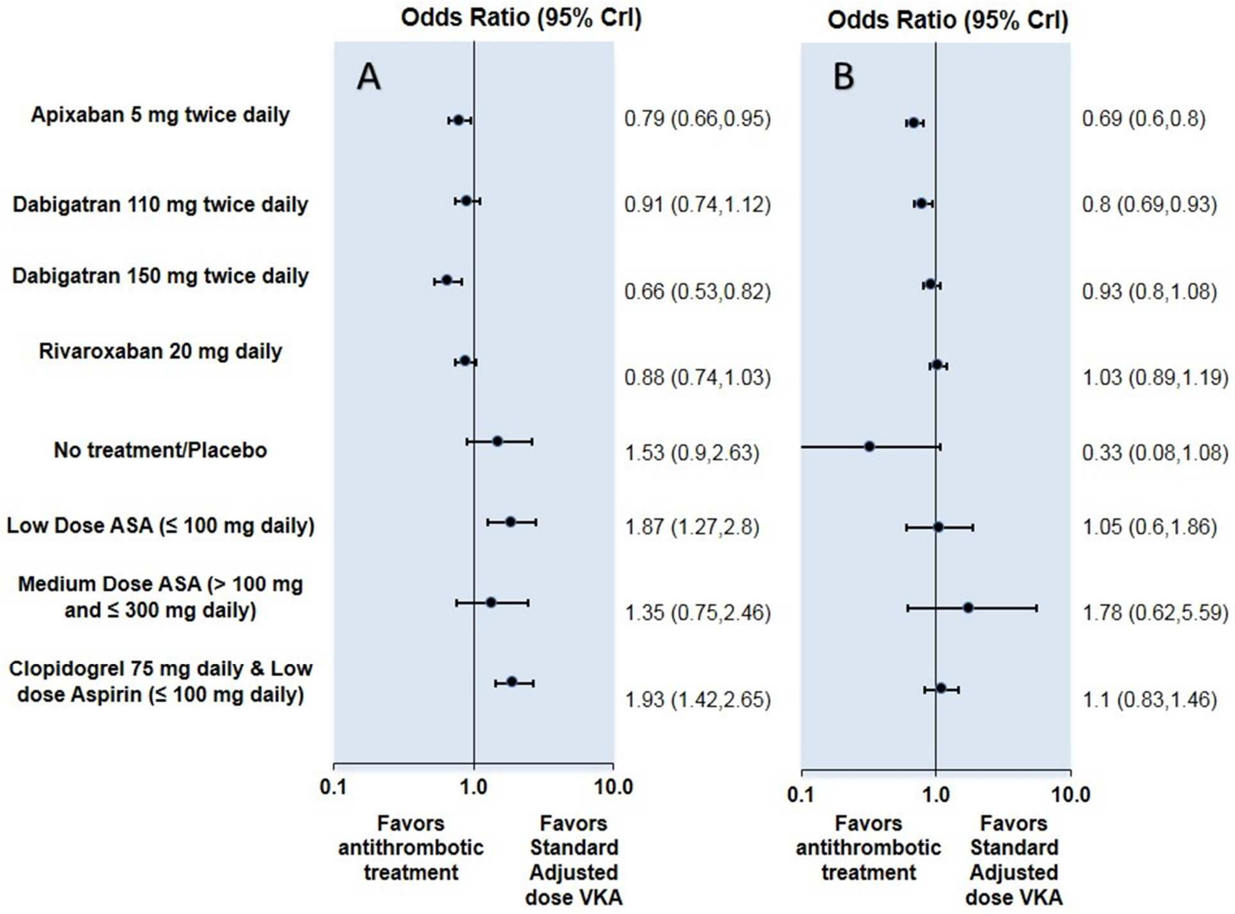

ASA= acetylsalicylic acid; CrI= credible interval; mg= milligram; VKA= vitamin K antagonist

only

For peer review only - http://bmjopen.bmj.com/site/about/guidelines.xhtml

Figure 3: Odds Ratios (OR) from network meta-analyses for stroke or systemic embolism and major bleeding for all pair-wise comparisons.* ORs for recurrence of stroke or systemic embolism are below the diagonal (row-defining treatment versus column-defining treatment) and those for major bleeding are above the diagonal and in blue (column-defining treatment versus row-defining treatment). To obtain ORs for comparisons in the opposite direction, reciprocals should be taken (e.g., the OR for standard dose warfarin compared with apixaban 5 mg twice daily for stroke or systemic embolism is 1/0.79=1.27). Significant results are in bold and underscored (and in larger font).

Standard adjusted dose VKA	**0.69 (0.6,0.8)**	**0.8 (0.69,0.93)**	0.93 (0.8,1.08)	1.03 (0.89,1.19)	0.33 (0.08,1.08)	1.05 (0.6,1.86)	1.78 (0.62,5.59)	1.1 (0.83,1.46)
**0.79 (0.66,0.95)**	Apixaban 5 mg twice daily	1.15 (0.94,1.43)	**1.34 (1.09,1.65)**	**1.48 (1.2,1.82)**	0.48 (0.12,1.57)	1.52 (0.85,2.73)	2.57 (0.88,8.16)	**1.59 (1.15,2.19)**
0.91 (0.74,1.12)	1.15 (0.88,1.52)	Dabigatran 110 mg twice daily	1.16 (0.997,1.36)	**1.28 (1.04,1.58)**	0.41 (0.1,1.36)	1.31 (0.74,2.37)	2.23 (0.77,7.06)	1.38 (0.99,1.9)
**0.66 (0.53,0.82)**	0.83 (0.62,1.11)	**0.72 (0.58,0.9)**	Dabigatran 150 mg twice daily	1.1 (0.9,1.35)	0.35 (0.09,1.16)	1.13 (0.63,2.04)	1.91 (0.66,6.06)	1.18 (0.86,1.63)
0.88 (0.74,1.03)	1.11 (0.86,1.42)	0.96 (0.74,1.25)	**1.33 (1.01,1.76)**	Rivaroxaban 20 mg daily	0.32 (0.08,1.06)	1.03 (0.57,1.84)	1.73 (0.6,5.5)	1.07 (0.78,1.48)
1.53 (0.9,2.63)	**1.94 (1.1,3.43)**	1.68 (0.95,3)	**2.33 (1.3,4.18)**	**1.75 (1,3.08)**	No treatment/ Placebo	3.19 (0.89,13.7)	**5.41 (1.6,23.81)**	3.33 (0.99,13.6)
**1.87 (1.27,2.8)**	**2.37 (1.54,3.68)**	**2.05 (1.32,3.23)**	**2.84 (1.82,4.5)**	**2.14 (1.4,3.31)**	1.22 (0.72,2.08)	Low Dose ASA	1.69 (0.52,5.94)	1.05 (0.55,1.97)
1.35 (0.75,2.46)	1.71 (0.92,3.19)	1.48 (0.79,2.8)	**2.05 (1.09,3.9)**	1.55 (0.83,2.87)	0.88 (0.55,1.41)	0.72 (0.39,1.35)	Medium Dose ASA	0.62 (0.19,1.84)
**1.93 (1.42,2.65)**	**2.44 (1.71,3.51)**	**2.12 (1.46,3.08)**	**2.93 (2.01,4.31)**	**2.21 (1.55,3.15)**	1.26 (0.68,2.34)	1.03 (0.62,1.7)	1.43 (0.73,2.79)	Clopidogrel 75 mg daily & Low dose ASA

ASA= acetylsalicylic acid; mg= milligram; VKA= vitamin K antagonist

* Results between individual treatments, especially newer oral anticoagulants, should be interpreted with caution given the limitations associated with using a fixed-effects model. See Appendix 7 for additional details.

For peer review only - http://bmjopen.bmj.com/site/about/guidelines.xhtml

Figure 4: Icon array illustrating the absolute risks of Stroke or systemic embolism (blue) and major bleeding episodes (red) per 1000 patients treated

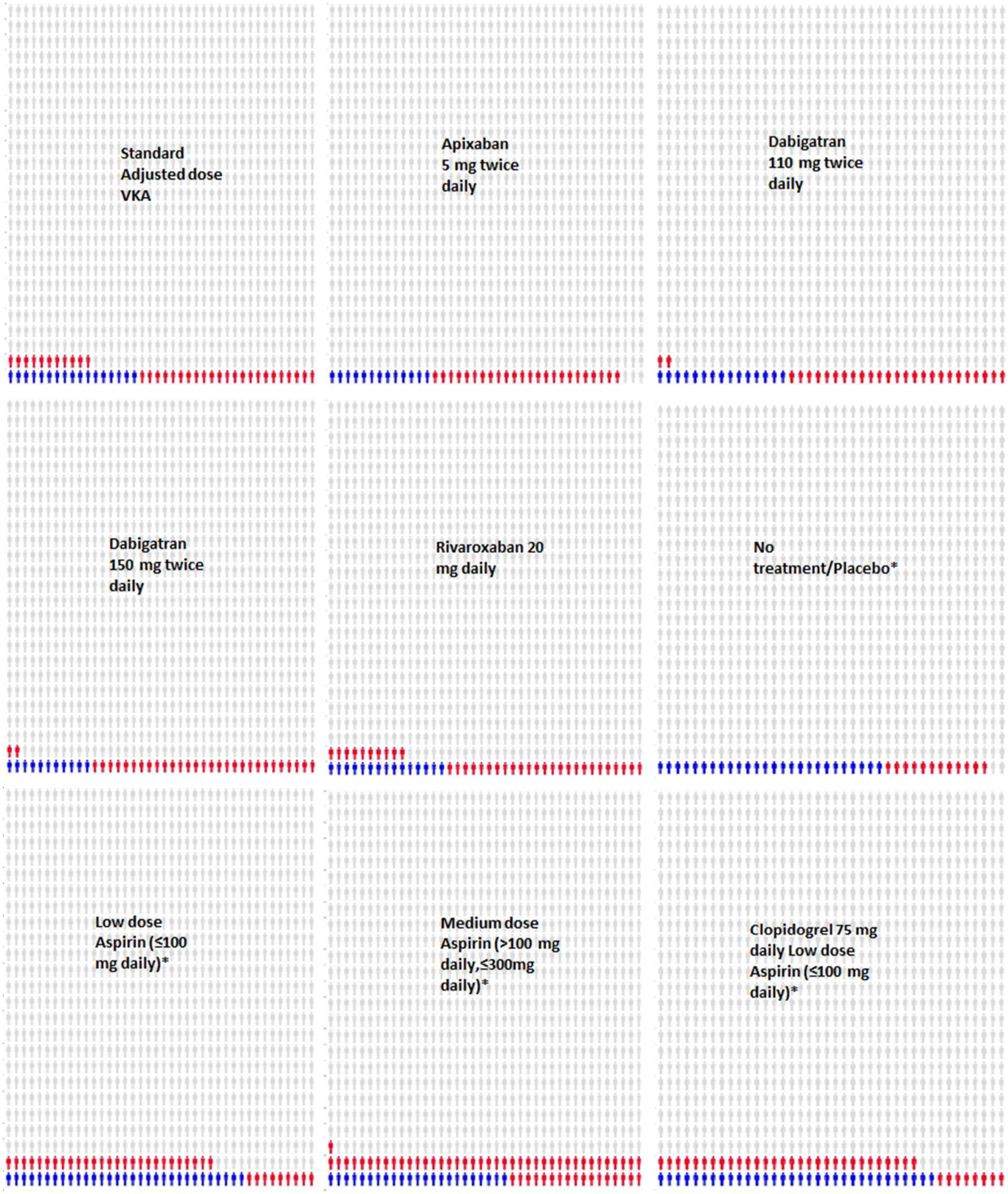

* Figures do not reflect uncertainty around effect estimates and there is more uncertainty (i.e., wider credible intervals) for no treatment/placebo, low dose ASA, medium dose ASA, and clopidogrel plus low dose ASA (See Appendix 6)

For peer review only - http://bmjopen.bmj.com/site/about/guidelines.xhtml

Acknowledgments: The authors would like to acknowledge Carolyn Spry. She was the Information Specialist from CADTH on the review who conducted the search strategy.

Contributors: CC conceived the study design, performed the data extraction, analysis, and drafted the manuscript. DC and GW conceived the study design, helped with the data analysis, and reviewed the manuscript for important intellectual content. TR conceived the study design, performed the systematic review, data extraction, and reviewed the manuscript for important intellectual content. SK, SS, KG, AB, KM, KC performed the systematic review, data extraction, and reviewed the manuscript for important intellectual content. TC, MC, and KC conceived the study and reviewed the manuscript for important intellectual content. All authors have read and approved the final version of the manuscript. All authors agree to be accountable for all aspects of the work in ensuring that questions related to the accuracy or integrity of any part of the work are appropriately investigated and resolved.

Funding information: This research was supported through a grant by the Canadian Institute of Health Research (CIHR) Drug Safety and Effectiveness Network and financial support from the Canadian Agency for Drugs and Technologies in Health (CADTH). The CIHR Drug Safety and Effectiveness Network played no role in study design, collection, analysis, interpretation of data, writing of the manuscript, or in the decision to submit the paper for publication. They accept no responsibility for the contents. Employees from CADTH were involved in the study design, collection, analysis, interpretation of data, and writing of the manuscript. CADTH, funded by Canadian federal, provincial and territorial governments, is an independent, not-for-profit producer and broker of health technology assessments. Federal, provincial, and territorial health care decision-makers rely on our evidence-based information to make informed decisions about the effectiveness and efficiency of drugs and other health technologies.

Competing interests: All authors have completed the ICMJE uniform disclosure form at www.icmje.org/coi_disclosure.pdf (available on request from the corresponding author) and declare: no support from any organisation for the submitted work; CC is a recipient of a Vanier Canada Graduate Scholarship from the Canadian Institutes of Health Research and has received funding from Canadian Network and Centre for Trials Internationally (CANNeCTIN) and is a trainee on the CIHR Drug Safety and Effectiveness Network team grant. MC is a recipient of a New Investigator Award from the Heart and Stroke Foundation of Canada and holds a T2 research chair in cancer and thrombosis from the University of Ottawa.

Ethical approval: Not required.

Data sharing: The statistical code and dataset used for analyses are reported online: http://www.cadth.ca/media/pdf/TR0003_AntithromboticAgents-AF_ScienceReport-e.pdf. Additional data can be requested by emailing George Wells: gawells@ottawaheart.ca .

The Corresponding Author has the right to grant on behalf of all authors and does grant on behalf of all authors, an exclusive licence (or non exclusive for government employees) on a worldwide basis to the BMJ Publishing Group Ltd to permit this article (if accepted) to be published in BMJ editions and any other BMJPGL products and sublicences such use and exploit all subsidiary rights, as set out in our licence

For peer review only

For peer review only - http://bmjopen.bmj.com/site/about/guidelines.xhtml

REFERENCES

1. Kirchhof, P. *et al.* Atrial fibrillation guidelines across the Atlantic: a comparison of the current recommendations of the European Society of Cardiology/European Heart Rhythm Association/European Association of Cardiothoracic Surgeons, the American College of Cardiology Fou. *European heart journal* 1471–1474 (2013).doi:10.1093/eurheartj/ehs446

2. Skanes, A. C. *et al.* Focused 2012 update of the Canadian Cardiovascular Society atrial fibrillation guidelines: recommendations for stroke prevention and rate/rhythm control. *The Canadian journal of cardiology* **28**, 125–36 (2012).

3. Schulman, S. & Kearon, C. Definition of major bleeding in clinical investigations of antihemostatic medicinal products in non-surgical patients. *Journal of thrombosis and haemostasis□: JTH* **3**, 692–4 (2005).

4. NHS Quality Improvement Scotland (NHS QIS) *SIGN 50. A guideline developer's handbook*. (2008).at <http://www.sign.ac.uk/pdf/sign50.pdf>

5. Moher, D., Liberati, A., Tetzlaff, J. & Altman, D. G. Preferred reporting items for systematic reviews and meta-analyses: the PRISMA statement. *PLoS medicine* **6**, e1000097 (2009).

6. Dias, S., Welton, N. J., Sutton, A. J. & Ades, A. *NICE DSU Technical Support Document 2: A Generalised Linear Modelling Framework for Pairwise and Network Meta-Analysis of Randomised Controlled Trials*. *Tropical Medicine* 1–96 (2011).at <www.nicedsu.org.uk>

7. Connolly, S. & Ezekowitz, M. Dabigatran versus warfarin in patients with atrial fibrillation. *... England Journal of ...* 1139–1151 (2009).at <http://www.nejm.org/doi/full/10.1056/NEJMoa0905561>

8. Ingelheim, B. *Advisory committee briefing document: dabigatran etexilate [Internet]*. 1–168 (2010).at <http://www.fda.gov/downloads/advisorycommittees/committeesmeetingmaterials/drugs/cardiovas>

9. Cipriani, A. *et al.* Comparative efficacy and acceptability of 12 new-generation antidepressants: a multiple-treatments meta-analysis. *Lancet* **373**, 746–58 (2009).

10. Turner, R. M., Davey, J., Clarke, M. J., Thompson, S. G. & Higgins, J. P. Predicting the extent of heterogeneity in meta-analysis, using empirical data from the Cochrane Database of Systematic Reviews. *International journal of epidemiology* **41**, 818–27 (2012).

For peer review only

For peer review only - http://bmjopen.bmj.com/site/about/guidelines.xhtml

11. Spiegelhalter, D. J., Best, N. G., Carlin, B. P. & Van der Linde, A. Bayesian measures of model complexity and fit. *Journal of the Royal Statistical Society: Series B (Statistical Methodology)* **64**, 583–639 (2002).

12. Spiegelhalter, D., Thomas, A. & Best, N. WinBUGS user manual. *MRC Biostatistics Unit* **2**, (2004).

13. meta: Meta-Analysis with R. at <http://cran.r-project.org/web/packages/meta/index.html>

14. Dias, S., Welton, N.J., Sutton, A.J., Caldwell, D.M., Guobing, L. & Ades, A. E. *NICE Technical Support Document 4: Inconsistency in Networks of Evidence Based on Randomised Controlled Trials*. 1–39 (2011).at <www.nicedsu.org.uk>

15. Salanti, G., Marinho, V. & Higgins, J. P. T. A case study of multiple-treatments meta-analysis demonstrates that covariates should be considered. *Journal of clinical epidemiology* **62**, 857–64 (2009).

16. Wells, G., Coyle, D., Cameron, C., Steiner, S. & Kelly, S. *Safety, effectiveness, and costeffectiveness of new oral anticoagulants compared with warfarin in preventing stroke and other cardiovascular events in patients with atrial fibrillation*. (2012).at <http://www.cadth.ca/media/pdf/NOAC_Therapeutic_Review_final_report.pdf>

17. Johnson & Johnson Pharmaceutical Research & Development *Rivaroxaban for the Prevention of Stroke and Non-Central Nervous System (CNS) Systemic Embolism in Patients With Atrial Fibrillation*. **7939**, 222 (2011).

18. McDonagh, M. S., Peterson, K., Balshem, H. & Helfand, M. US Food and Drug Administration documents can provide unpublished evidence relevant to systematic reviews. *Journal of clinical epidemiology* (2013).doi:10.1016/j.jclinepi.2013.05.006

19. Granger, C. & Alexander, J. Apixaban versus warfarin in patients with atrial fibrillation. *... England Journal of ...* 981–992 (2011).at <http://www.nejm.org/doi/full/10.1056/NEJMoa1107039>

20. Patel, M., Mahaffey, K. & Garg, J. Rivaroxaban versus warfarin in nonvalvular atrial fibrillation. *... England Journal of ...* 883–891 (2011).at <http://www.nejm.org/doi/full/10.1056/nejmoa1009638>

21. Connolly, S. *et al.* Clopidogrel plus aspirin versus oral anticoagulation for atrial fibrillation in the Atrial fibrillation Clopidogrel Trial with Irbesartan for prevention of Vascular Events (ACTIVE W): a randomised controlled trial. *Lancet* **367**, 1903–12 (2006).

22. Ezekowitz, M. D. *et al.* Dabigatran with or without concomitant aspirin compared with warfarin alone in patients with nonvalvular atrial fibrillation (PETRO Study). *The American journal of cardiology* **100**, 1419–26 (2007).

For peer review only - http://bmjopen.bmj.com/site/about/guidelines.xhtml

23. Ogawa, S., Shinohara, Y. & Kanmuri, K. Safety and Efficacy of the Oral Direct Factor Xa Inhibitor Apixaban in Japanese Patients With Non-Valvular Atrial Fibrillation. *Circulation Journal* **75**, 1852–1859 (2011).

24. Rash, A. *et al.* A randomised controlled trial of warfarin versus aspirin for stroke prevention in octogenarians with atrial fibrillation (WASPO). *Age and ageing* **36**, 151–6 (2007).

25. Connolly, S. & Eikelboom, J. Apixaban in patients with atrial fibrillation. ... *England Journal of ...* (2011).at <http://www.nejm.org/doi/full/10.1056/NEJMoa1007432>

26. Connolly, S. J. *et al.* Effect of clopidogrel added to aspirin in patients with atrial fibrillation. *The New England journal of medicine* **360**, 2066–78 (2009).

27. Harenberg, J. & Weiss, C. Clinical trials with new oral anticoagulants. Additive value of indirect comparisons also named network meta-analyses. *Hämostaseologie* **33**, 62–70 (2013).

28. Salanti, G. Exploring the geometry of treatment networks. *Annals of internal ...* (2008).at <http://annals.org/article.aspx?articleid=740377>

29. Barratt, A. *et al.* Tips for learners of evidence-based medicine: 1. Relative risk reduction, absolute risk reduction and number needed to treat. *Canadian Medical Association journal* **171**, 353–8 (2004).

30. Schechtman, E. Odds ratio, relative risk, absolute risk reduction, and the number needed to treat--which of these should we use? *Value in health : the journal of the International Society for Pharmacoeconomics and Outcomes Research* **5**, 431–6 (2002).

31. Berger, M. L., Mamdani, M., Atkins, D. & Johnson, M. L. Good research practices for comparative effectiveness research: defining, reporting and interpreting nonrandomized studies of treatment effects using secondary data sources: the ISPOR Good Research Practices for Retrospective Database Analysis Task Force . *Value in health : the journal of the International Society for Pharmacoeconomics and Outcomes Research* **12**, 1044–52 (2009).

32. Berger, M. L. *et al.* Prospective observational studies to assess comparative effectiveness: the ISPOR good research practices task force report. *Value in health : the journal of the International Society for Pharmacoeconomics and Outcomes Research* **15**, 217–30 (2012).

33. Jansen, J. P. Network meta-analysis of individual and aggregate level data. *Research Synthesis Methods* **3**, 177–190 (2012).

For peer review only

34. Salanti, G., Ades, a E. & Ioannidis, J. P. a Graphical methods and numerical summaries for presenting results from multiple-treatment meta-analysis: an overview and tutorial. *Journal of clinical epidemiology* **64**, 163–71 (2011).

35. Guo, J. J. *et al.* A review of quantitative risk-benefit methodologies for assessing drug safety and efficacy-report of the ISPOR risk-benefit management working group. *Value in health : the journal of the International Society for Pharmacoeconomics and Outcomes Research* **13**, 657–66 (2010).

36. Boyd, C. *et al. Methods Research Report Methods for Benefit and Harm Assessment in Systematic Reviews*. 52 (Rockville, MD, 2012).at <www.ncbi.nlm.nih.gov/books/NBK115750/>

For peer review only

SUPPLEMENTARY APPENDIX

Appendix 1: Search Strategy

**OVERVIEW**	
Interface:	Ovid
Databases:	EBM Reviews - Cochrane Central Register of Controlled Trials <May 2012><br>Ovid Embase 1974 to June 6, 2012<br>Ovid MEDLINE 1946 to June 7, 2012<br>Ovid MEDLINE Daily<br>Ovid MEDLINE In-Process & Other Non-Indexed Citations<br>**Note:** Subject headings have been customized for each database. Duplicates between databases were removed in Ovid.
Date of Search:	June 7, 2012
Alerts:	Monthly search updates began June 11, 2012 and ran until project completion
Study Types:	Randomized Controlled Trials
Limits:	Publication years 1988-present<br>Humans<br>Conference abstracts omitted

**SYNTAX GUIDE**	
/	At the end of a phrase, searches the phrase as a subject heading
.sh	At the end of a phrase, searches the phrase as a subject heading
MeSH	Medical Subject Heading
exp	Explode a subject heading
*	Before a word, indicates that the marked subject heading is a primary topic;<br>or, after a word, a truncation symbol (wildcard) to retrieve plurals or varying endings
#	Truncation symbol for one character
ADJ	Requires words are adjacent to each other (in any order)
ADJ#	Adjacency within # number of words (in any order)
.ti	Title
.ab	Abstract
.hw	Heading Word; usually includes subject headings and controlled vocabulary
.nm.	Name of substance word
.ot	Original title
.pt	Publication type
.rn	CAS registry number
use cctr	Select results from Cochrane database of Clinical Trials
use oemezd	Select results from Embase database
use pmez	Select results from Medline database

For peer review only - http://bmjopen.bmj.com/site/about/guidelines.xhtml

Clinical Multi-database Strategy	
Line #	Search Strategy
1	(211914-51-1 or 211915-06-9).rn,nm. or (dabigatran* or Pradaxa or Pradax or Prazaxa or Praxada or bibr 1048 or bibr1048 or bibr953 or bibr 953 or rendix).ti,ot,ab,sh,rn,hw,nm.
2	(BMS 562247 or BMS562247 or apixaban* or Eliquis or Eliques).ti,ot,ab,sh,rn,hw,nm. or 503612-47-3.rn,nm.
3	(rivaroxaban* or Xarelto or BAY 59-7939 or BAY59-7939 or BAY597939 or BAY 597939).ti,ot,ab,sh,rn,hw,nm. or 366789-02-8.rn,nm.
4	(90055-48-4 or 113665-84-2).rn,nm. or (clopidogrel* or clopilet or grepid or iscover or plavix or zopya or zylagren or zyllt or duocover or duoplavin).ti,ot,ab,sh,rn,hw,nm.
5	Aspirin/ or 50-78-2.rn,nm. or (acetylsalicylic acid* or ASA* or acenterine or acesal or acetan or acetard or aceticil or aceticyl or acetilum or acetonyl or acetilum or acetonyl or acetophen or acetosal or acetosalicylic acid or acetosalin or acetosalum or acetyl salicylate or acetyl salicylic acid or acetylin or acetylo or acetylon or acetylosalicylic acid or acetylsal or acetysal or acetylsalicyclic acid or acetylsalicyl or acetylsalicylate or acetylsalycic acid or acetylsalycylic acid or oracidulatum or actorin or Acylpyrin or acylpyrine or acytosal or adiro or alabunkun or alasil or albyl-e or alka seltzer or alkASA or Aloxiprimum or anacin or anasprin or anopyrin or ansin or anthrom or aptor or arthralgyl or asaflow or ascription or orasaphen or asapor or asatard or asawin or aspec or aspent or aspergum or aspex or aspilets or aspirem or aspirgran or ASAa or ASAe or ASAine or aspirisucre or aspisol or aspo cid or aspro or asrina or asrivo or asta or asteric or astrix or bebesan or biprin or bokey or boxazin or breoprin or bufferin or cafenol or caprin or cASA or catalgine or catalgix or cemerit or cemirit or claradin or claragine or Colfarit or comoprin or contrheuma or daga or darosal or Dispril or dispirin or dolean or dusil or Easprin or ecasil or Ecotrin or egalgic or emocin or empirin or encaprin or encine em or Endosprin or entaprin or entercin or enteroprin or enterosarine or enterospirine or entrophen or eskotrin or euthermine or extren or genasprin or globentyl or godamed or gotosan or helicon or idotyl or infatabs a or istopirin or istopyrine or ivepirine or juvepirine or keypo or kilios or kinderASA or Magnecyl or measurin or mejoral or melabon or Micristin or micropyrin or mikristin or miniasal or mycrisitin or naspro or novasen or nu seal or nu seals or ortho acetoxybenzoate or ortho acetoxybenzoic acid or ortho acetyloxybenzoate or ortho acetyloxybenzoic acid or ostoprin or pancemol or paracin or paynocil or pengo or plewin or Polopirin or polopiryna or premaspin or primaspan or proprin or pyronoval or reumyl or rhodine or rhonal or ronal or salacetin or salisalido or sargepirine or soldral or solpyron or Solprin or solucetyl or solupsa or Solupsan or spren or super tru or tapal or temagin or tevapirin or th 2152 or thrombo-aspilets or treupahlin or treuphalin or tromalyt or tromcor or turiital or verin or vitalink or xaxa or Zorprin).ti,ot,ab,sh,rn,hw,nm.
6	Warfarin/ or 81-81-2.rn,nm. or (adoisine or aldocumar or athrombin* or befarin or brumolin or carfin or circuvit or compound 42 or coumadan or coumadin or coumadine or coumafen or coumafene or coumefene or coumaphene or coumarins or dagonal or farin or jantoven or kumatox or maforan or marevan or orfarin or panwarfarin or panwarfin or prothromadin or simarc 2 or sofarin or tedicumar or tintorane or waran or warfant or warfar or warfarin or warfarine or warfil or warfilone or warnerin).ti,ot,ab,sh,rn,hw,nm.
7	Acenocoumarol/ or 152-72-7.rn,nm. or (acenocoumarol* or nicoumalone or acenocoumarin or acenocoumarine or acenocoumarole or acenocoumarolum or acenocumarol or acenocumarolo or acenocumerol or acenkumarin or acitrom or ascumar or neo sintrom or neosintrom or neositron or nicoumalone or nicumalon or nitrovarfarian or nitrowarfarin or sincoumar or sincumar or sinkumar or sinthrom or sinthrome or sintrom or sintroma or sintron or syncoumar or syncumar or synthrom or syntrom or syntroma or sintron or syncoumar or syncumar or syntrom or trombostop or zotil or sinthrome or synthrom or syncoumar or syncumar or sinkumar or sintrom or minisintrom or trombostop or zotil).ti,ot,ab,sh,rn,hw,nm.
8	or/1-7
9	Atrial fibrillation/
10	((atrial or atrium or auricular or heart) adj1 fibrillation*).ti,ab.
11	(a-fib or afib).ti,ab.
12	9 or 10 or 11
13	8 and 12
14	13 use pmez
15	13 use cctr
16	14 or 15
17	*dabigatran/ or (dabigatran* or Pradaxa or Pradax or Prazaxa or praxada or bibr 1048 or bibr1048 or

For peer review only - http://bmjopen.bmj.com/site/about/guidelines.xhtml

	bibr953 or bibr 953 or rendix).ti,ab.
18	*apixaban/ or (BMS 562247 or BMS562247 or apixaban* or Eliquis or Eliques).ti,ab.
19	*rivaroxaban/ or (rivaroxaban* or Xarelto or BAY 59-7939 or BAY59-7939 or BAY597939 or BAY 597939).ti,ab.
20	*clopidogrel/ or *acetylsalicylic acid plus clopidogrel/ or (clopidogrel* or clopilet or grepid or iscover or plavix or zopya or zylagren or zyllt or duocover or duoplavin).ti,ab.
21	*warfarin/ or (adoisine or aldocumar or athrombin* or befarin or brumolin or carfin or circuvit or compound 42 or coumadan or coumadin or coumadine or coumafen or coumafene or coumefene or coumaphene or coumarins or dagonal or farin or jantoven or kumatox or maforan or marevan or orfarin or panwarfarin or panwarfin or prothromadin or simarc 2 or sofarin or tedicumar or tintorane or waran or warfant or warfar or warfarin* or warfarine or warfil or warfilone or warnerin).ti,ab.
22	*acetylsalicylic acid/ or (acetylsalicylic acid* or ASA* or acenterine or acesal or acetan or acetard or aceticil or aceticyl or acetilum or acetonyl or acetilum or acetonyl or acetophen or acetosal or acetosalicylic acid or acetosalin or acetosalum or acetyl salicylate or acetyl salicylic acid or acetylin or acetylo or acetylon or acetylosalicylic acid or acetylsal or acetysal or acetylsalicyclic acid or acetylsalicyl or acetylsalicylate or acetylsalycic acid or acetylsalycylic acid or oracidulatum or actorin or Acylpyrin or acylpyrine or acytosal or adiro or alabunkun or alasil or albyl-e or alka seltzer or alkASA or Aloxiprimum or anasprin or anopyrin or ansin or anthrom or aptor or arthralgyl or asaflow or orasaphen or asapor or asatard or asawin or aspec or aspent or aspergum or aspex or aspilets or aspirem or aspirgran or ASAa or ASAe or ASAine or aspirisucre or aspisol or aspo cid or aspro or asrina or asrivo or asta or asteric or astrix or bebesan or biprin or bokey or boxazin or breoprin or bufferin or cafenol or caprin or cASA or catalgine or catalgix or cemerit or cemirit or claradin or claragine or Colfarit or comoprin or contrheuma or daga or darosal or Dispril or dispirin or dolean or dusil or Easprin or ecasil or Ecotrin or egalgic or emocin or empirin or encaprin or encine em or Endosprin or entaprin or entercin or enteroprin or enterosarine or enterospirine or entrophen or eskotrin or euthermine or extren or genasprin or globentyl or godamed or gotosan or helicon or idotyl or infatabs a or istopirin or istopyrine or ivepirine or juvepirine or keypo or kilios or kinderASA or Magnecyl or measurin or mejoral or melabon or Micristin or micropyrin or mikristin or miniasal or mycrisitin or naspro or novasen or nu seal or nu seals or ortho acetoxybenzoate or ortho acetoxybenzoic acid or ortho acetyloxybenzoate or ortho acetyloxybenzoic acid or ostoprin or pancemol or paracin or paynocil or pengo or plewin or Polopirin or polopiryna or premaspin or primaspan or proprin or pyronoval or reumyl or rhodine or rhonal or ronal or salacetin or salisalido or sargepirine or soldral or solpyron or Solprin or solucetyl or solupsa or Solupsan or spren or super tru or tapal or temagin or tevapirin or th 2152 or thrombo-aspilets or treupahlin or treuphalin or tromalyt or tromcor or turiital or verin or vitalink or xaxa or Zorprin).ti,ab.
23	*Acenocoumarol/ or (acenocoumarol* or nicoumalone or acenocoumarin or acenocoumarine or acenocoumarole or acenocoumarolum or acenocumarol or acenocumarolo or acenocumerol or acenkumarin or acitrom or ascumar or neo sintrom or neosintrom or neositron or nicoumalone or nicumalon or nitrovarfarian or nitrowarfarin or sincoumar or sincumar or sinkumar or sinthrom or sinthrome or sintrom or sintroma or sintron or syncoumar or syncumar or synthrom or syntrom or syntroma or sintron or syncoumar or syncumar or syntrom or trombostop or zotil or sinthrome or synthrom or syncoumar or syncumar or sinkumar or sintrom or minisintrom or trombostop or zotil).ti,ab.
24	or/17-23
25	Heart atrium fibrillation/
26	((atrial or atrium or auricular or heart) adj1 fibrillation*).ti,ab.
27	(a-fib or afib).ti,ab.
28	25 or 26 or 27
29	24 and 28
30	29 use oemezd
31	30 not conference abstract.pt.
32	16 or 31
33	32
34	limit 33 to english language [Limit not valid in CCTR; records were retained]
35	(Randomized Controlled Trial or Controlled Clinical Trial).pt.
36	Randomized Controlled Trial/
37	Randomized Controlled Trials as Topic/
38	"Randomized Controlled Trial (topic)"/

For peer review only - http://bmjopen.bmj.com/site/about/guidelines.xhtml

39	Controlled Clinical Trial/
40	Controlled Clinical Trials as Topic/
41	"Controlled Clinical Trial (topic)"/
42	Randomization/
43	Random Allocation/
44	Double-Blind Method/
45	Double Blind Procedure/
46	Double-Blind Studies/
47	Single-Blind Method/
48	Single Blind Procedure/
49	Single-Blind Studies/
50	Placebos/
51	Placebo/
52	Control Groups/
53	Control Group/
54	(random* or sham or placebo*).ti,ab,hw.
55	((singl* or doubl*) adj (blind* or dumm* or mask*)).ti,ab,hw.
56	((tripl* or trebl*) adj (blind* or dumm* or mask*)).ti,ab,hw.
57	(control* adj3 (study or studies or trial*)).ti,ab.
58	(Nonrandom* or non random* or non-random* or quasi-random* or quasirandom*).ti,ab,hw.
59	allocated.ti,ab,hw.
60	((open label or open-label) adj5 (study or studies or trial*)).ti,ab,hw.
61	or/35-60
62	34 and 61
63	limit 62 to yr = "1988 -Current"
64	remove duplicates from 63

**OTHER DATABASES**	
PubMed	Same MeSH, keywords, limits, and study types used as per MEDLINE search, with appropriate syntax used.

Grey Literature

**Dates for Search:**	**June 12, 2012 – July 8, 2012**
**Keywords:**	dabigatran, Pradax, Pradaxa, apixaban, Eliquis, rivaroxaban, Xarelto, clopidogrel, Plavix, acetylsalicylic acid, Aspirin, warfarin, Coumadin, acenocoumarol, Sintrom, Sinthrome<br>Atrial fibrillation
**Limits:**	Publication years 1988-present, English

Relevant items from the following sections of the CADTH grey literature checklist, "Grey matters: a practical tool for evidence-based searching" (http://www.cadth.ca/resources/grey-matters) were searched:

- Health Technology Assessment Agencies
- Health Economic
- Clinical Practice Guidelines
- Databases (free)

Internet Search

For peer review only - http://bmjopen.bmj.com/site/about/guidelines.xhtml

Appendix 2: PRISMA Diagram

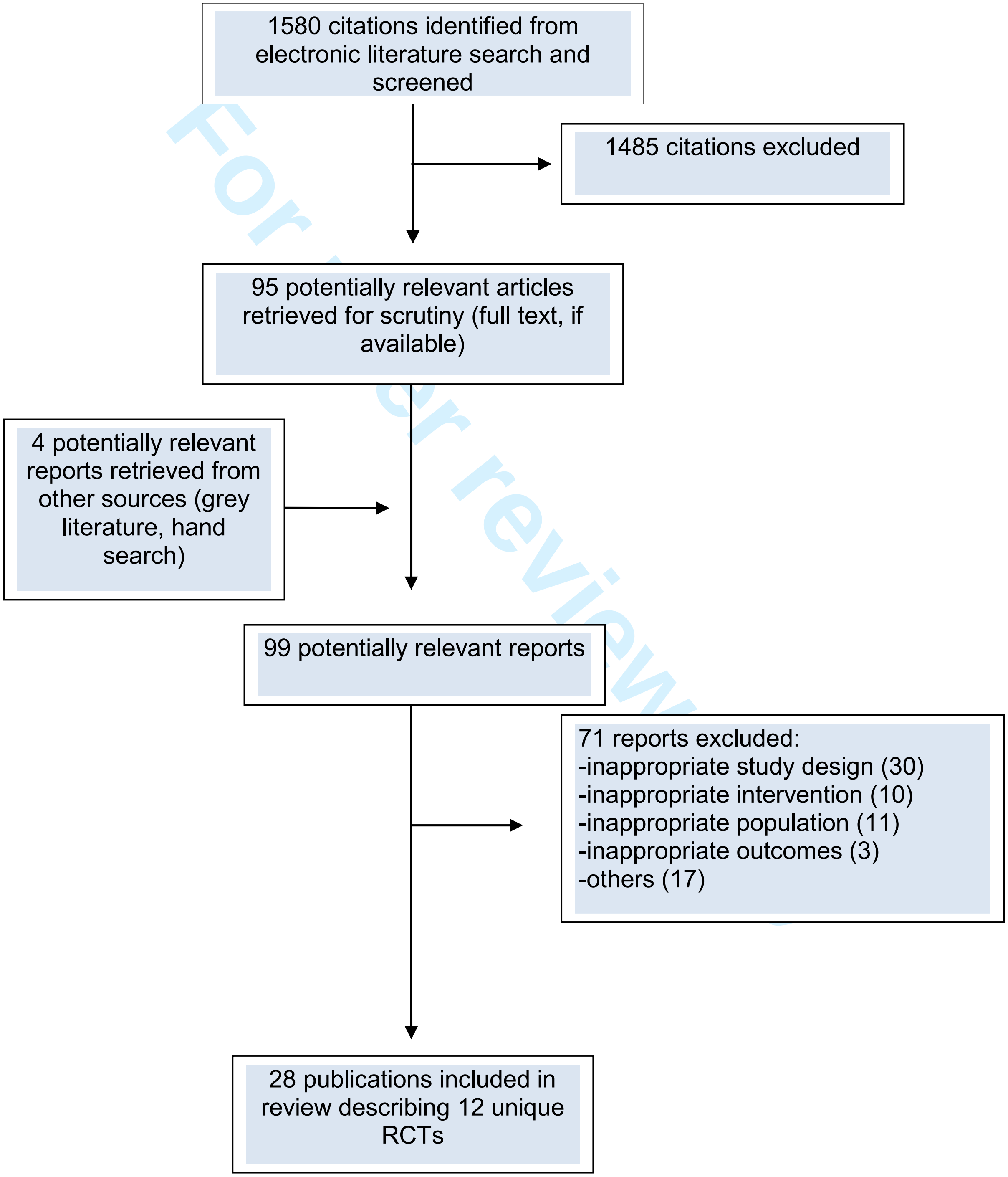

For peer review only - http://bmjopen.bmj.com/site/about/guidelines.xhtml

Appendix 3: Summary of Patient Baseline Characteristics

Study	Baseline Characteristics															Centre above TTR Throughout follow-up		
	Age, Mean ± SD (y)	Age Category (%)		Sex (%)		CHADS$_2$, Mean ± SD	CHADS$_2$ Category (%)			Prior Stroke /TIA (%)	DM (%)	Heart Failure (%)	HBP (%)	MI (%)	VKA Naïve (%)	Mean TTR (%)	TTR Category (%)	
		≤75y	>75 y	M	F		0	1	≥2								≤66%	>66%
ACTIVE-W n=6706	70 ± NR	NR	NR	66	34	2 ± 1.1	NR	NR	NR	15	21	30	82	17	23	64	48	52
AFASAK n=672	75 ± NR	NR	NR	54	46	NR ± NR	NR	NR	NR	6	9	53	32	7	NR	N/A	N/A	N/A
AFASAK-2 n=339	73 ± NR	NR	NR	61	39	NR ± NR	NR	NR	NR	8	12	70	45	7	NR	NR	NR	NR
ARISTOTLE n=18201	70 ± NR	69	31	65	35	2.1 ± 1.1	0	34	66	19	25	35	87	14	43	62	NR	NR
ARISTOTLE-J n=222	70 ± NR	69	31	83	17	1.9 ± NR	1	42	57	28	23	1	83	NR	16	60	NR	NR
BAFTA n=973	82 ± 4	0	100	55	45	NR ± NR	NR	NR	NR	13	13	20	54	11	NR	67	NR	NR
CAFA n=378	68 ± NR	NR	NR	75	25	NR ± NR	NR	NR	NR	4	12	22	39	13	NR	44	NR	NR
JAST n=871	65 ± NR	NR	NR	70	30	NR ± NR	NR	NR	NR	3	14	9	39	NR	92	N/A	N/A	N/A
PETRO n=236	70 ± NR	70	30	82	18	NR ± NR	NR	NR	NR	18	25	32	71	NR	0	57	NR	NR
RE-LY n=18113	71 ± NR	60	40	64	36	2.1 ± NR	32		68	20	20	23	32	79	17	64	50	50
ROCKET-AF n=14264	73 ± NR	NR	NR	60	40	3.5 ± NR	0	0	100	55	40	62	91	17	38	55	NR	NR
WASPO n=75	83 ± NR	0	100	47	53	NR ± NR	NR	NR	NR	NR	4	NR	47	NR	NR	69	NR	NR

DM = diabetes mellitus; F = female sex; HBP = high blood pressure; M = male sex; MI = myocardial infarction; N/A = not applicable; NR = not reported; SD = standard deviation; TIA = transient ischemic attack; TTR = time in therapeutic range; VKA = vitamin K antagonist; y = year.

Note: Only data from the included relevant treatment arms have been extracted.

For peer review only - http://bmjopen.bmj.com/site/about/guidelines.xhtml

Appendix 4: Summary of Included Trials

Study and Design	Disposition	Population	Interventions	Follow-up	Primary Outcome(s)
**ACTIVE-W**[13-16]<br>OL RCT, 2006.<br>Multicenter, 32 countries (including Europe / America).	Randomized: n=6706<br>Completed: n=6026 (90%)	AF patients eligible / willing to take anticoagulant therapy, with ≥ 1 pre-specified risk factor for stroke.	**Clopidogrel / Low-dose ASA** (n=3335)<br>**Warfarin – INR 2.0 to 3.0** (n=3371)	1.3 years (stopped early)	First occurrence of stroke, non-CNS SE, MI or vascular death.
**AFASAK**[17]<br>OL/DB RCT, 1989.<br>Multicenter, Copenhagen.	Randomized: n=1007<br>Completed: n=785 (80%)	AF patients, with no recent anticoagulation or cerebrovascular event, valve replacement or rheumatic heart disease.	***OL Warfarin – INR 2.8 to 4.2**** *(n=335)*<br>**Low-dose ASA** (n=336)<br>**PL** (n=336)	2 years	Thromboembolic complication: stroke, TIA or embolic complication to viscera / extremities.
**AFASAK-2**[18,19]<br>OL RCT, 1998.<br>Multicenter, Copenhagen.	Randomized: n=677<br>Completed: n=507 (75%)	AF patients ≥ 60 years (unless with CV or pulmonary disease), without recent TIA or risk factors for bleeding.	***Fixed-dose Warfarin**** *(n=167)*<br>***Fixed-dose Warfarin / Medium-dose ASA**** *(n=171)*<br>**Medium Dose ASA** (n=169)<br>**Warfarin – INR 2.0 to 3.0** (n=170)	3.5 years	Stroke or SE.
**ARISTOTLE**[20-23]<br>DB RCT, 2011.<br>Multicenter, 39 countries (including Europe / America).	Randomized: n=18201<br>Completed: n=13468 (74%)	AF patients with ≥ 1 risk factor for stroke, without recent stroke or ASA > 165 mg.	**Apixaban 5 mg BID** (n=9120)<br>**Warfarin – INR 2.0 to 3.0** (n=9081)<br>Concomitant use of **ASA** (≤ 165 mg daily) or **NSAIDs** permitted.	NR	Stroke or SE;<br>Major bleeding.
**ARISTOTLE-J**[24]<br>OL/DB RCT, 2011.<br>Multicenter, Japan.	Randomized: n=222<br>Completed: n=197 (89%)	AF patients with ≥ 1 risk factor for stroke, without recent TIA or ASA > 100 mg.	**Apixaban 2.5 mg BID** (n=74)<br>**Apixaban 5 mg BID** (n=74)<br>**OL Warfarin – INR 2.0 to 3.0** (n=74)<br>Concomitant use of **ASA** (≤ 100 mg daily) permitted.	12 weeks	Composite of major bleeding and CRNM bleeding events.
**BAFTA**[25]<br>OL RCT, 2007.<br>Multicenter, UK.	Randomized: n=973<br>Completed: n=694 (72%)	AF patients ≥ 75 years, with clinical uncertainty around optimal treatment.	**Low-dose ASA** (n=485)<br>**Warfarin – INR 2.0 to 3.0** (n=488)	2.7 years	Disabling stroke, ICH and other clinically significant arterial embolism.
**CAFA**[26]<br>DB RCT, 1991.<br>Multicenter, Canada.	Randomized: n=378<br>Completed: n=286 (76%)	AF patients without recent stroke, TIA or MI, and who did not require antiplatelet drug therapy.	**Warfarin – INR 2.0 to 3.0** (n=187)<br>**PL** (n=191)	1.2 years (stopped early)	Ischemic stroke, SE, ICH or fatal hemorrhage.
**JAST**[27]<br>OL RCT, 2006.<br>Multicenter, Japan.	Randomized: n=871<br>Completed: n=686 (79%)	AF patients without recent symptomatic thromboembolic disease, ICH or GI bleeding.	**Medium-dose ASA** (n=426)<br>**No treatment** (n=445)	2.1 years (stopped early)	CV death, symptomatic brain infarction, or TIA.

For peer review only - http://bmjopen.bmj.com/site/about/guidelines.xhtml

Study and Design	Disposition	Population	Interventions	Follow-up	Primary Outcome(s)
**PETRO**[28]<br>OL/DB RCT, 2007. Multicenter, 4 countries (Europe and USA).	Randomized: n=502<br>Completed: n=464 (92%)	AF patients with ≥ 1 risk factor for stroke and with coronary artery disease (removed halfway to facilitate recruitment). No recent MI, stroke, TIA or major bleed. Warfarin pre-treatment required for ≥ 8 weeks.	***Dabigatran 50 mg BID ± Low-dose or High-dose ASA*** ** (n=105)*<br>***Dabigatran 300 mg BID ± Low-dose or High-dose ASA*** ** (n=161)*<br>**Dabigatran 150 mg BID ± Low-dose or High-dose ASA** † (n=166)<br>**OL Warfarin – INR 2.0 to 3.0** (n=70)	12 weeks	Thromboembolic events and bleeding events.
**RE-LY**[29-36]<br>OL/DB RCT, 2009. Multicenter, 44 countries (including Europe / America).	Randomized: n=18113<br>Completed: n=14580 (80%)	AF patients with ≥ 1 risk factor for stroke, without recent stroke or increased risk of bleeding.	**Dabigatran 110 mg BID** (n=6015)<br>**Dabigatran 150 mg BID** (n=6076)<br>**OL Warfarin – INR 2.0 to 3.0** (n=6022)<br>Concomitant use of **ASA** (<100 mg daily) or other **antiplatelet agents** permitted.	2 years	Stroke or SE and major haemorrhage.
**ROCKET-AF**[37-39]<br>DB RCT, 2011. Multicenter, 45 countries (including Europe / America).	Randomized: n=14264<br>Completed: n=10957 (77%)	AF patients at moderate to high risk of stroke (CHADS2 score ≥ 2).	**Rivaroxaban 20 mg OD** (n=7131)<br>**Warfarin – INR 2.0 to 3.0** (n=7133)<br>Over a third of patients in both arms took concomitant ASA.	1.9 years	Composite of stroke and SE;<br>Composite of major and NMCR bleeding.
**WASPO**[40]<br>OL RCT, 2007. Multicenter, UK.	Randomized: n=75<br>Completed: n=64 (85%)	Ambulant AF patients > 80 and < 90 years with Folstein score > 25, without previous fall, ICH or GI bleeding. Patients with prior stroke or TIA were excluded.	**Warfarin – INR 2.0 to 3.0** (n=36)<br>**Medium-dose ASA** (n=39)	1 year	Combined endpoints: death; stroke, TIA or SE; serious bleeding; withdrawal.
**AF** = atrial fibrillation; **ASA** = acetylsalicylic acid; **CNS** = central nervous system; **CRNM** = clinically relevant non-major bleeding; **CV** = cardiovascular; **DB** = double-blind; **GI** = gastrointestinal; **ICH** = intracerebral hemorrhage; **MI** = myocardial infarction; **NR** = not reported; **NSAIDs** = non-steroidal anti-inflammatory drugs; **OL** = open label; **PL** = placebo; **RCT** = randomized controlled trial; **SE** = systemic embolism; **TIA** = transient ischemic attack; **UK** = United-Kingdom; **VKA** = vitamin K antagonist.					

* No data was extracted from these treatment arms since they did not meet the inclusion criteria specified in the protocol. These trials were nevertheless included in the systematic review because they had at least one relevant comparison between two interventions of interest.

† Data was extracted only for dabigatran without ASA (n=100).

For peer review only - http://bmjopen.bmj.com/site/about/guidelines.xhtml

Appendix 5: Evidence network for major bleeding. The width of the lines is proportional to the number of randomized controlled trials (RCTs) comparing each pair of treatments, and the size of each treatment node is proportional to the number of randomised participants (sample size). A dotted line indicates a three-arm RCT

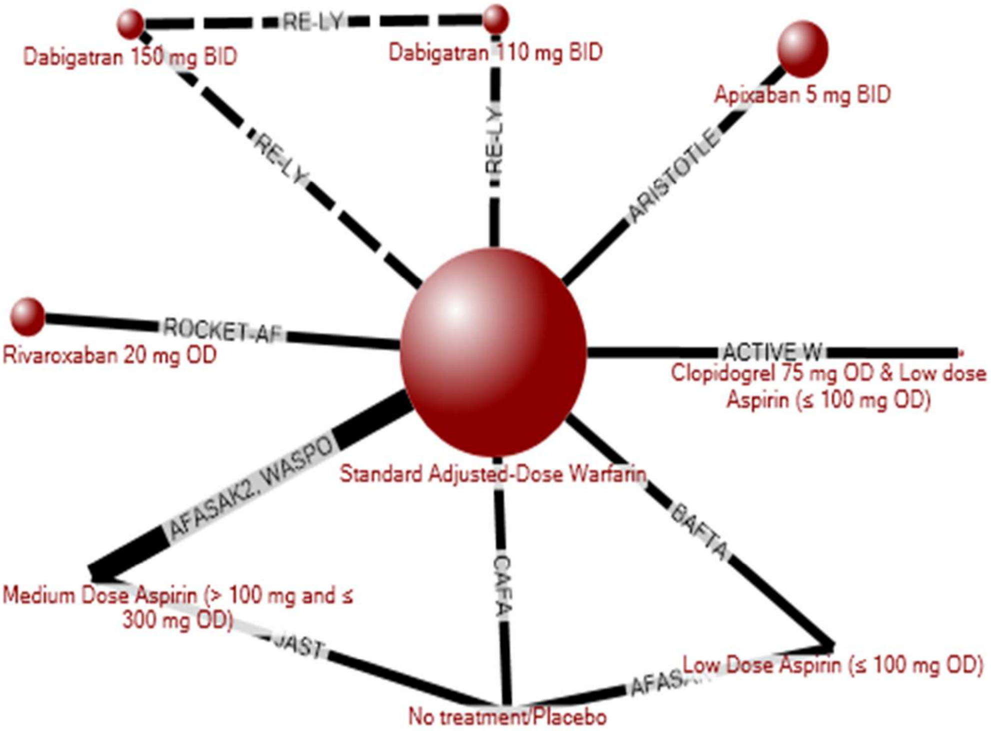

mg= milligram; RCT = randomized controlled trial

review only

For peer review only - http://bmjopen.bmj.com/site/about/guidelines.xhtml

Appendix 6: Summary of results from Network Meta-analysis for All-cause Stroke or Systemic Embolism

Treatment	Pair-wise Meta-Analysis, OR (95% CI) versus standard adjusted dose VKA	NMA, Odds Ratio (95% CrI) versus standard adjusted dose VKA	NMA, Absolute Risk Difference per 1,000 treated (95% CrI) versus standard adjusted dose VKA	Probability Best (%) for Outcome	Rank for Outcome
Standard Adjusted-dose VKA	NA	NA	NA	0.00%	5.00
Apixaban 5 mg twice daily	0.79 (0.66,0.94)	0.79 (0.66,0.95)	4 fewer (1 fewer-6 fewer)	9.90%	2.30
Dabigatran 110 mg twice daily	0.91 (0.75,1.11)	0.91 (0.74,1.12)	2 fewer (2 more-5 fewer)	0.13%	3.80
Dabigatran 150 mg twice daily	0.66 (0.53,0.82)	0.66 (0.53,0.82)	6 fewer (3 fewer-8 fewer)	87.50%	1.10
Rivaroxaban 20 mg daily	0.88 (0.74,1.05)	0.88 (0.74,1.03)	2 fewer (1 more-5 fewer)	1.22%	3.30
No treatment/Placebo	1.84 (0.66,5.1)	1.53 (0.9,2.63)	9 more (26 more-2 fewer)	0.12%	7.00
Low Dose ASA (≤ 100 mg daily)	2.03 (1.32,3.12)	1.87 (1.27,2.8)	14 more (29 more-5 more)	0.00%	8.10
Medium Dose ASA (> 100 mg and ≤ 300 mg daily)	0.83 (0.35,1.97)	1.35 (0.75,2.46)	6 more (23 more-5 fewer)	1.14%	6.20
Clopidogrel 75 mg daily & Low dose Aspirin (≤ 100 mg daily)	1.93 (1.41,2.64)	1.93 (1.42,2.65)	15 more (26 more-7 more)	0.00%	8.20

CI = confidence interval; CrI = credible interval; OR= odds ratio

Appendix 7: Summary of results from Network Meta-analysis for Major Bleeding

Treatment	Pair-wise Meta-Analysis, OR (95% CI) versus standard adjusted dose VKA	NMA, Odds Ratio (95% CrI) versus standard adjusted dose VKA	NMA, Absolute Risk Difference per 1,000 treated (95% CrI) versus standard adjusted dose VKA	Probability Best (%) for Outcome	Rank for Outcome
Standard Adjusted-dose VKA	NA	NA	NA	0.00%	6.00
Apixaban 5 mg twice daily	0.69 (0.6,0.79)	0.69 (0.6,0.8)	10 fewer (7 fewer-13 fewer)	10.13%	2.10
Dabigatran 110 mg twice daily	0.8 (0.68,0.94)	0.8 (0.69,0.93)	7 fewer (3 fewer-10 fewer)	0.84%	3.20
Dabigatran 150 mg twice daily	0.93 (0.81,1.07)	0.93 (0.8,1.08)	3 fewer (3 more-7 fewer)	0.01%	4.90
Rivaroxaban 20 mg daily	1.03 (0.9,1.18)	1.03 (0.89,1.19)	1 more (6 more-4 fewer)	0.00%	6.40
No treatment/Placebo	0.39 (0.08,2.02)	0.33 (0.08,1.08)	22 fewer (3 more-31 fewer)	87.97%	1.40
Low Dose ASA (≤ 100 mg daily)	1.01 (0.57,1.78)	1.05 (0.6,1.86)	2 more (26 more-13 fewer)	0.96%	6.10
Medium Dose ASA (> 100 mg and ≤ 300 mg daily)	1.89 (0.59,6.03)	1.78 (0.62,5.59)	24 more (118 more-13 fewer)	0.09%	8.10
Clopidogrel 75 mg daily & Low dose Aspirin (≤ 100 mg daily)	1.1 (0.82,1.48)	1.1 (0.83,1.46)	4 more (15 more-6 fewer)	0.02%	7.00

BID= twice daily; CI = confidence interval; CrI = credible interval; OD= once daily; OR= odds ratio

For peer review only - http://bmjopen.bmj.com/site/about/guidelines.xhtml

Appendix 8: Benefit-Harm plot for each treatment (absolute harm difference per 1,000 patients treated). The absolute risk difference of recurrence of Stroke or systemic embolism relative to standard adjusted dose VKA is presented on the x-axis, while the absolute risk difference of major bleeding relative to standard adjusted dose VKA is on the y-axis. The dot is representative of the median estimate for each treatment while the 95% credible intervals are presented as error bars.

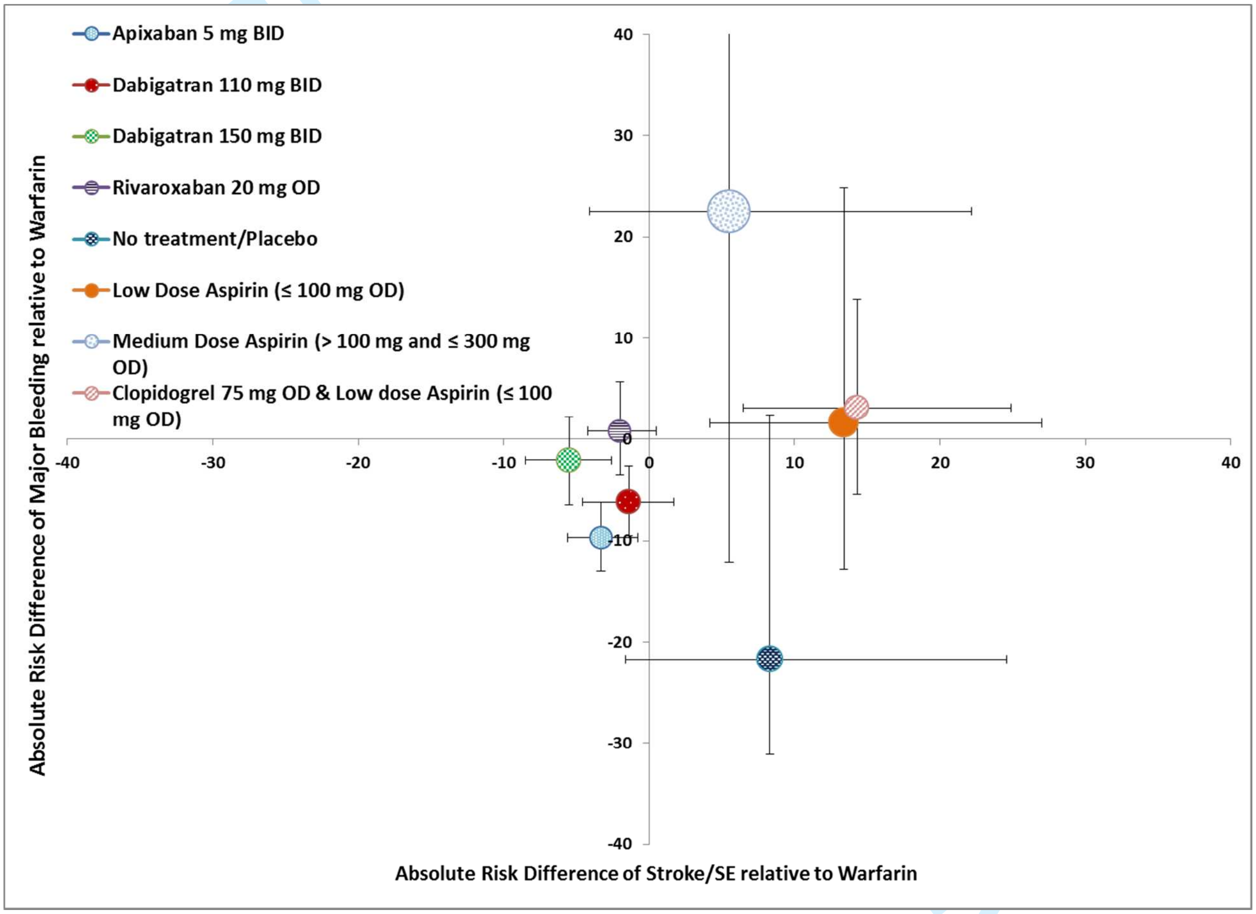

For peer review only - http://bmjopen.bmj.com/site/about/guidelines.xhtml

Appendix 9: Rationale of choice of fixed-effects model and limitations

It is challenging to conduct network meta-analyses when evidence structures are comprised of single study connections, especially when there is obvious heterogeneity in the network. The use of a random-effects model with a vague prior on the between study variance exerts a large degree of influence on the credible intervals (CrI's) because there is insufficient studies to reign in the prior and provide an accurate estimate of the between study variance. Accordingly, we also considered informative priors on the between study variance from the recently published reports.[10] However, even when these priors were applied on the between study variance, they still did not reign in CrI's effect estimates again because the network was comprised of single study connections. The use of the random effects model with either a vague or informative prior was problematic because treatments that were original associated with statistically significant reductions in stroke or SE relative to standard adjusted dose VKA were no longer associated with significant reductions, limiting external validity of the model (see Figures below on direct estimates in Online Appendix 4 and 5). While we could have opted to use less vague priors (e.g., ¼ of prior in Turner et al[10]), the choice of the prior would have seemed somewhat arbitrary because it was not based on any empirical evidence. As such, we opted to use the fixed-effects model despite the known issues in doing so. By using the fixed-effects model, treatments that were associated with significant reductions in the risk of stroke or SE relative to standard adjusted dose VKA retained significance. We felt that it would have been inappropriate to remove statistical significance by applying vague priors given the large investment involved in developing clinical trials. However, a consequence of retaining statistical significance is seen when comparing newer oral anticoagulants versus one another in Figure 5. Because we assumed the new oral anticoagulants were all associated with reductions in Stroke or SE relative to standard adjusted dose VKA (if that was the case in the underlying trials), significant reductions in the risk of stroke or SE among new oral anticoagulants are observed in Figure 5. This may or may not be the case in clinical practice. As noted in tables 1 and Online Appendix 2, there is considerable heterogeneity across studies. However, the network structure itself (i.e., single study connections), made it difficult to properly adjust for differences in patient and study characteristics using standard approaches. Accordingly, results in Figure 5 should be interpreted with caution. We conducted simple sub-group analyses (breaking randomization) to help adjust for this heterogeneity, which is a very crude way to adjust for heterogeneity. To fully elucidate the comparative benefits and harms of antithrombotic agents, especially across the various sub-populations, rigorously conducted comparative studies, or network meta-regression analyses of patient-level data are required.

For peer review only

For peer review only - http://bmjopen.bmj.com/site/about/guidelines.xhtml

Forest Plot – Odds ratio (95% Credible interval) when using vague prior on the sd (i.e, sd~unif(0,2))

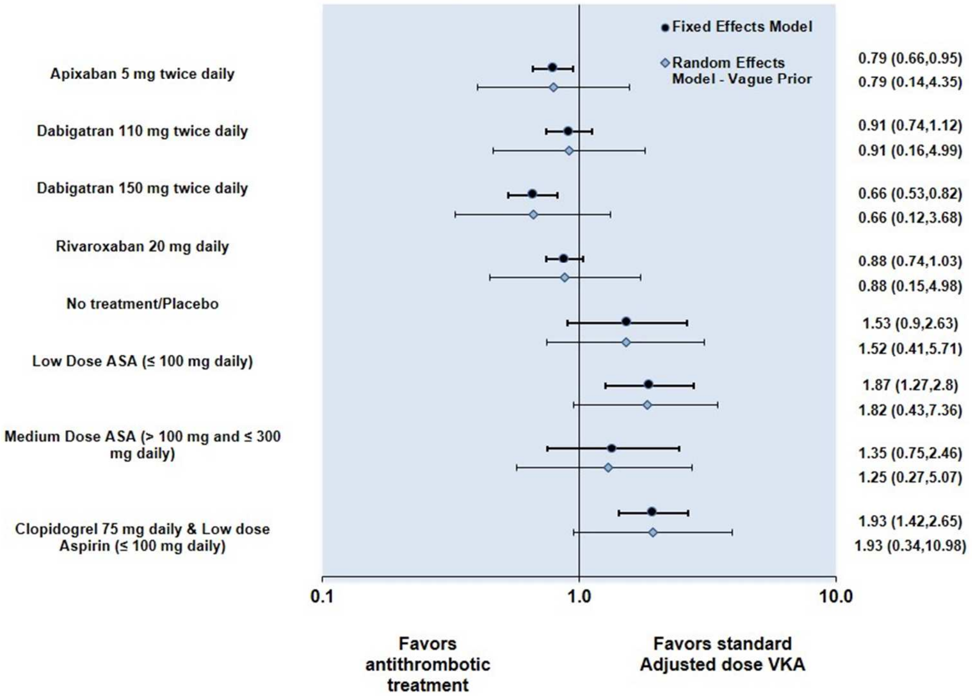

Forest Plot – Odds ratio (95% Credible interval) when using informative prior on the sd (i.e, dlnorm(-3.02,0.29)[10]

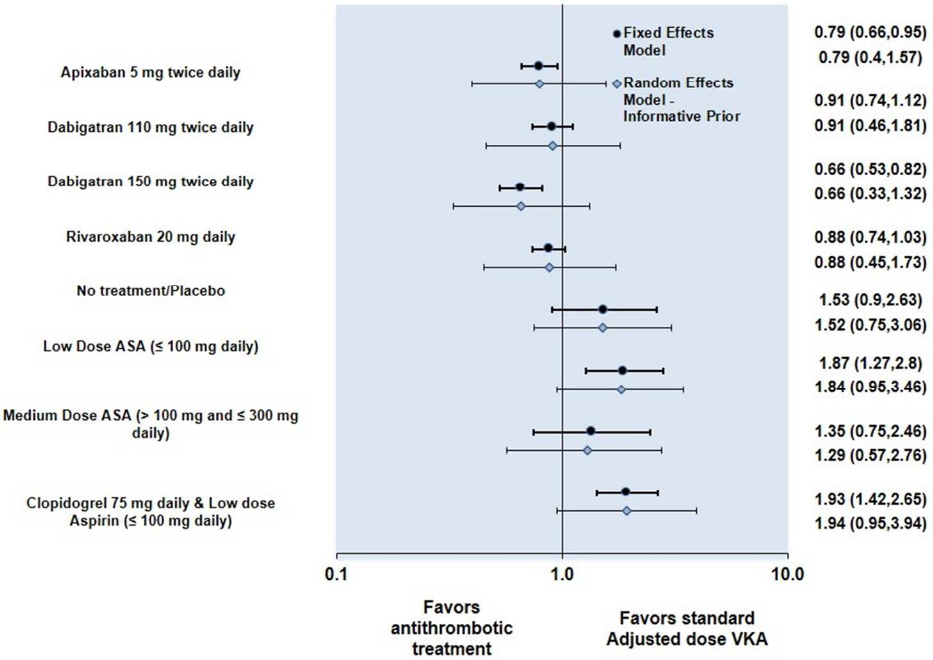

For peer review only - http://bmjopen.bmj.com/site/about/guidelines.xhtml

Appendix 10: PRISMA Checklist

Section/topic	#	Checklist item	Reported on page #
**TITLE**			
Title	1	Identify the report as a systematic review, meta-analysis, or both.	0
**ABSTRACT**			
Structured summary	2	Provide a structured summary including, as applicable: background; objectives; data sources; study eligibility criteria, participants, and interventions; study appraisal and synthesis methods; results; limitations; conclusions and implications of key findings; systematic review registration number.	0
**INTRODUCTION**			
Rationale	3	Describe the rationale for the review in the context of what is already known.	1
Objectives	4	Provide an explicit statement of questions being addressed with reference to participants, interventions, comparisons, outcomes, and study design (PICOS).	1
**METHODS**			
Protocol and registration	5	Indicate if a review protocol exists, if and where it can be accessed (e.g., Web address), and, if available, provide registration information including registration number.	2
Eligibility criteria	6	Specify study characteristics (e.g., PICOS, length of follow-up) and report characteristics (e.g., years considered, language, publication status) used as criteria for eligibility, giving rationale.	2-3
Information sources	7	Describe all information sources (e.g., databases with dates of coverage, contact with study authors to identify additional studies) in the search and date last searched.	2-3
Search	8	Present full electronic search strategy for at least one database, including any limits used, such that it could be repeated.	2-3
Study selection	9	State the process for selecting studies (i.e., screening, eligibility, included in systematic review, and, if applicable, included in the meta-analysis).	3
Data collection process	10	Describe method of data extraction from reports (e.g., piloted forms, independently, in duplicate) and any processes for obtaining and confirming data from investigators.	3
Data items	11	List and define all variables for which data were sought (e.g., PICOS, funding sources) and any assumptions and simplifications made.	3-4
Risk of bias in individual studies	12	Describe methods used for assessing risk of bias of individual studies (including specification of whether this was done at the study or outcome level), and how this information is to be used in any data synthesis.	3,6
Summary measures	13	State the principal summary measures (e.g., risk ratio, difference in means).	3
Synthesis of results	14	Describe the methods of handling data and combining results of studies, if done, including measures of consistency (e.g., I^2) for each meta-analysis.	4
Risk of bias across studies	15	Specify any assessment of risk of bias that may affect the cumulative evidence (e.g., publication bias, selective reporting within studies).	6
Additional analyses	16	Describe methods of additional analyses (e.g., sensitivity or subgroup analyses, meta-regression), if done, indicating which were pre-specified.	5
**RESULTS**			

For peer review only - http://bmjopen.bmj.com/site/about/guidelines.xhtml

Study selection	17	Give numbers of studies screened, assessed for eligibility, and included in the review, with reasons for exclusions at each stage, ideally with a flow diagram.	3,5
Study characteristics	18	For each study, present characteristics for which data were extracted (e.g., study size, PICOS, follow-up period) and provide the citations.	5-6, Table 1, Online Appendix 2
Risk of bias within studies	19	Present data on risk of bias of each study and, if available, any outcome level assessment (see item 12).	6
Results of individual studies	20	For all outcomes considered (benefits or harms), present, for each study: (a) simple summary data for each intervention group (b) effect estimates and confidence intervals, ideally with a forest plot.	Figure 3-4
Synthesis of results	21	Present results of each meta-analysis done, including confidence intervals and measures of consistency.	Figure 3-4, Online Appendix 4 and 5
Risk of bias across studies	22	Present results of any assessment of risk of bias across studies (see Item 15).	6
Additional analysis	23	Give results of additional analyses, if done (e.g., sensitivity or subgroup analyses, meta-regression [see Item 16]).	Table 2-3, Online Appendix 10
**DISCUSSION**			
Summary of evidence	24	Summarize the main findings including the strength of evidence for each main outcome; consider their relevance to key groups (e.g., healthcare providers, users, and policy makers).	11-12
Limitations	25	Discuss limitations at study and outcome level (e.g., risk of bias), and at review-level (e.g., incomplete retrieval of identified research, reporting bias).	13-14
Conclusions	26	Provide a general interpretation of the results in the context of other evidence, and implications for future research.	10-14
**FUNDING**			
Funding	27	Describe sources of funding for the systematic review and other support (e.g., supply of data); role of funders for the systematic review.	0

For peer review only

Appendix 11: Assessment of Inconsistency

We plotted the posterior mean deviance of the individual data points in the inconsistency model against their posterior mean deviance in the consistency model to help identify loops where inconsistency is present. In our analysis, the posterior mean deviance contributions are very similar and close to 1, for both models. The consistency model has a similar posterior mean of the residual deviance (19.28 vs. 19.13) and DIC (146.15 vs. 148.04) and hence is a similar fit for both models. A few points show a lower value of the posterior mean deviance in the inconsistency model but these values are not too far from 1. Similarly, a few points have higher values of the posterior mean deviance in the consistency model (again with values not too far from 1) suggesting that each model may fit the data points better for certain points. The parameter estimates are also similar for both models and there is considerable overlap in the 95% credible intervals (see table below), suggesting no evidence of inconsistency in the network.

Plot of posterior mean deviance of the individual data points in the inconsistency model against their posterior mean deviance in the consistency model

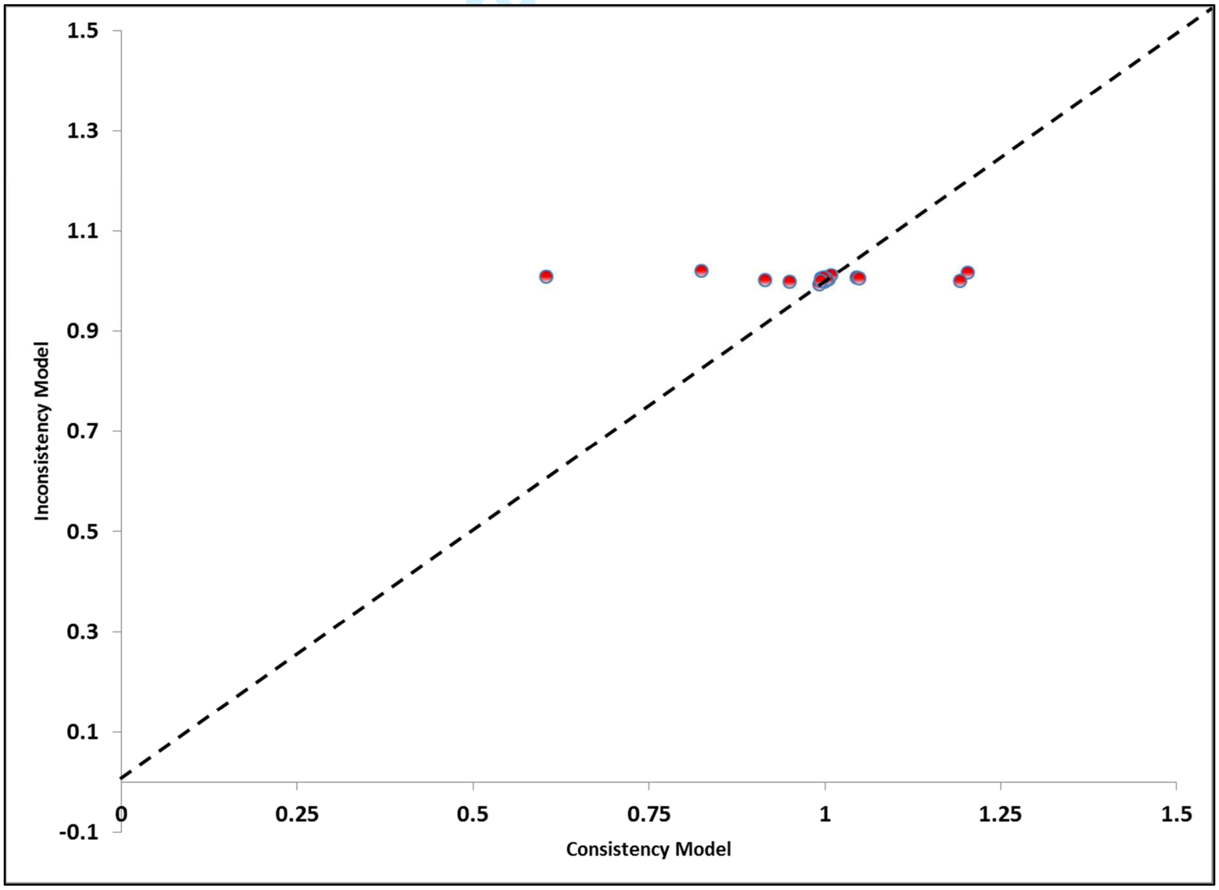

For peer review only - http://bmjopen.bmj.com/site/about/guidelines.xhtml

Comparison of effect estimates from consistency and inconsistency models where there is data available for comparison

Comparison	Consistency Model	Inconsistency Model
Apixaban 5 mg twice daily vs Standard Standard adjusted dose VKA	0.79 (0.66,0.95)	0.79 (0.66,0.95)
Dabigatran 110 mg twice daily vs Standard adjusted dose VKA	0.91 (0.74,1.12)	0.91 (0.74,1.12)
Dabigatran 150 mg twice daily vs Standard adjusted dose VKA	0.66 (0.53,0.82)	0.66 (0.53,0.82)
Rivaroxaban 20 mg daily vs Standard adjusted dose VKA	0.88 (0.74,1.03)	0.88 (0.74,1.03)
No treatment/Placebo vs Standard adjusted dose VKA	1.53 (0.9,2.63)	1.89 (0.7,5.72)
Low Dose Aspirin (≤ 100 mg daily) vs Standard adjusted dose VKA	1.87 (1.27,2.8)	2.04 (1.33,3.19)
Medium Dose Aspirin (> 100 mg and ≤ 300 mg daily) vs Standard adjusted dose VKA	1.35 (0.75,2.46)	0.82 (0.33,1.99)
Clopidogrel 75 mg daily & Low dose Aspirin (≤ 100 mg daily) vs Standard adjusted dose VKA	1.93 (1.42,2.65)	1.93 (1.42,2.64)
Low Dose Aspirin (≤ 100 mg daily) vs No treatment/Placebo	1.22 (0.72,2.08)	1.00 (0.51,1.98)
Medium Dose Aspirin (> 100 mg and ≤ 300 mg daily) vs No treatment/Placebo	0.88 (0.55,1.41)	1.05 (0.62,1.76)
Residual deviance	19.28	19.13
DIC	146.15	148.044

For peer review only - http://bmjopen.bmj.com/site/about/guidelines.xhtml

Appendix 12: Sensitivity analysis with AVERROES and ACTIVE A

A. Stroke or SE

Evidence Networks for Stroke or SE NMA including AVEROES and ACTIVE A studies

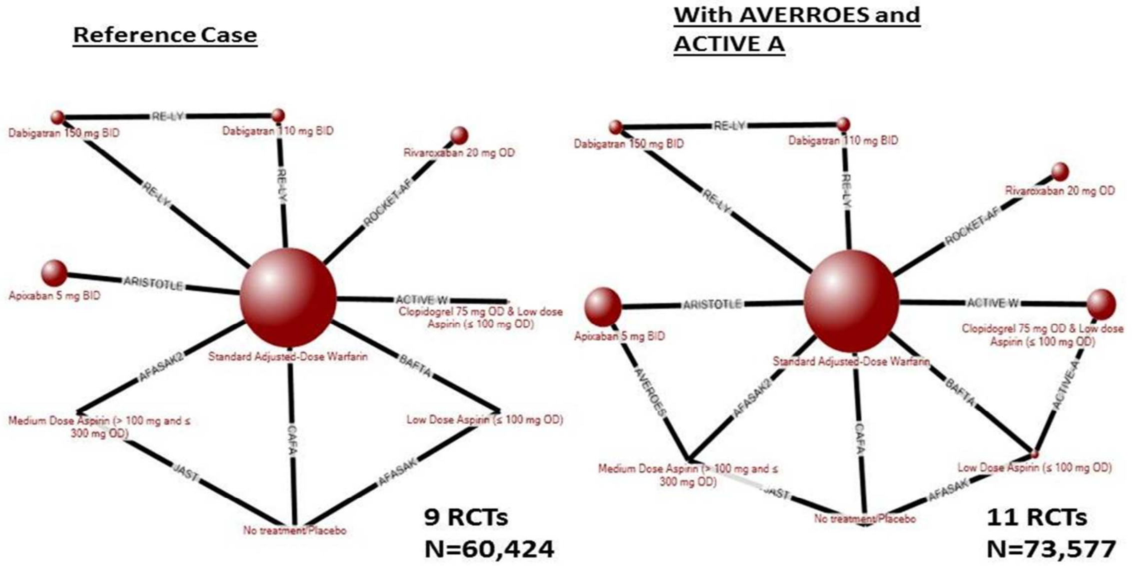

Forest Plot for Stroke or SE NMA including AVERROES and ACTIVE A studies

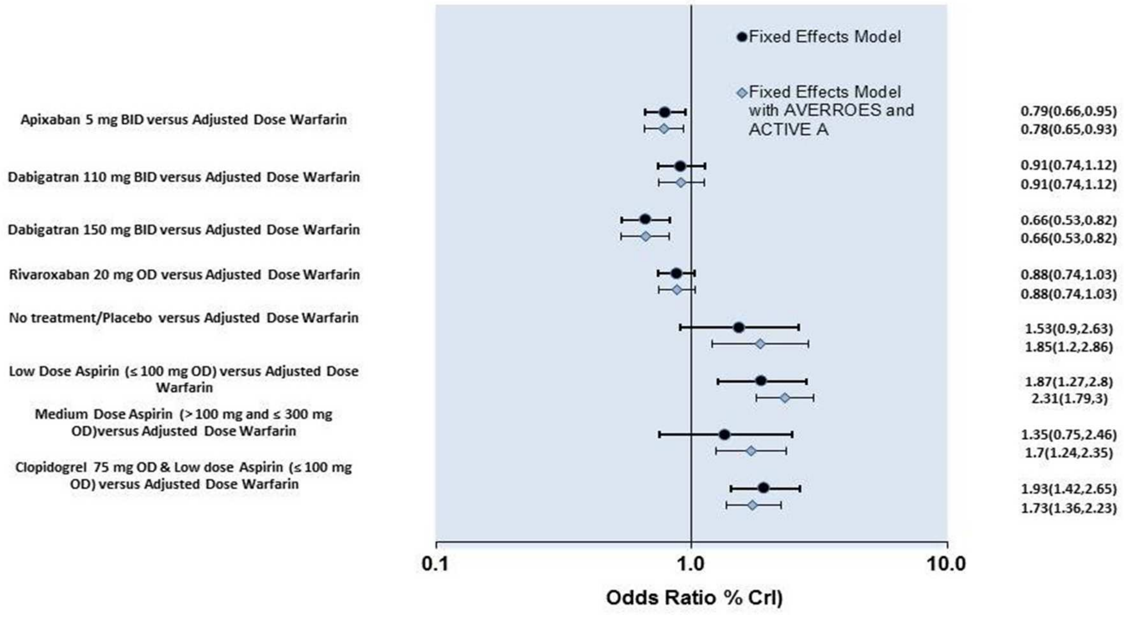

B. Major Bleeding

Evidence Networks for Major Bleeding NMA including AVERROES and ACTIVE A studies

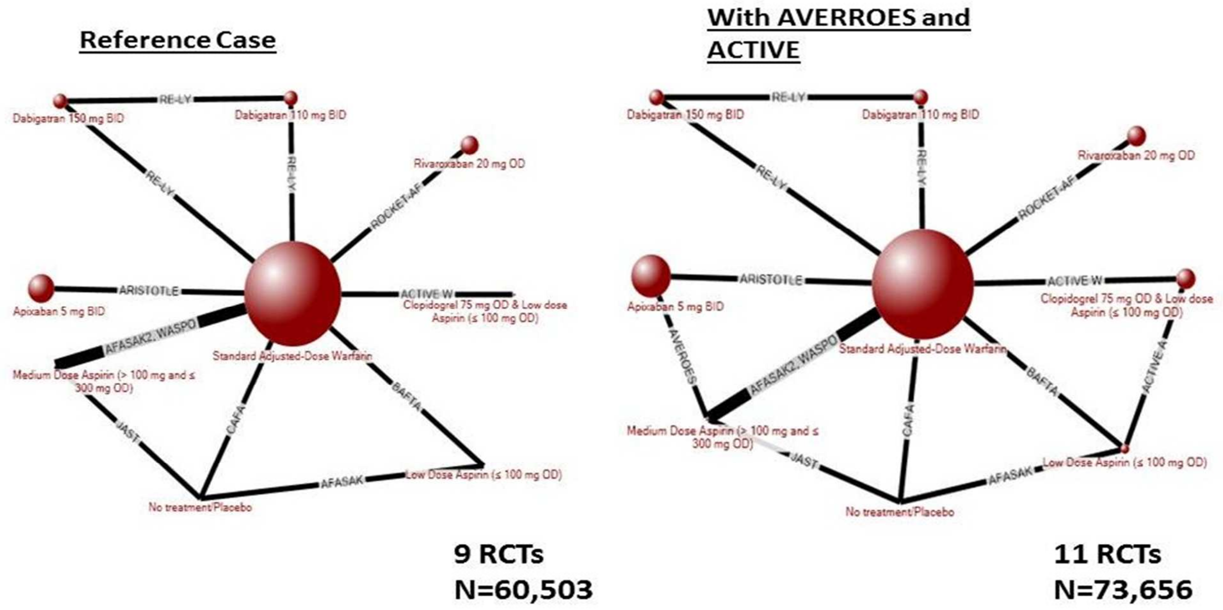

Forest Plot for Major Bleeding NMA including AVERROES and ACTIVE A studies

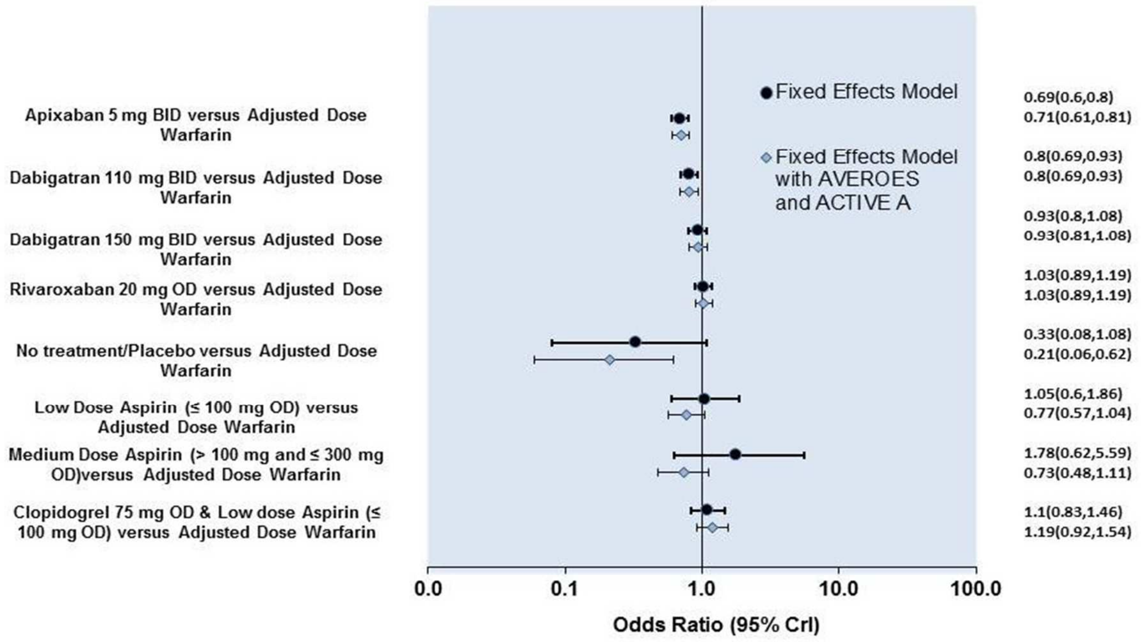

Systematic Review and Network Meta-analysis comparing Antithrombotic Agents for the Prevention of Stroke and Major Bleeding in Patients with Atrial Fibrillation

Journal:	*BMJ Open*
Manuscript ID:	bmjopen-2013-004301.R1
Article Type:	Research
Date Submitted by the Author:	13-Feb-2014

Complete List of Authors:	Cameron, Chris; University of Ottawa, Department of Community Medicine and Epidemiology<br>Coyle, Douglas; University of Ottawa, Epidemiology and Community Medicine<br>Richter, Trevor; Canadian Agency for Drugs and Technologies in Health,<br>Kelly, Shannon; Ottawa Hospital Research Institute, Medicine<br>Gauthier, Kasandra; Canadian Agency for Drugs and Technologies in Health,<br>Steiner, Sabine; Medical University of Vienna, Department of Internal Medicine/Cardiology<br>Carrier, Marc; Ottawa Hospital Research Institute, Medicine<br>Coyle, Kathryn; Applied Health Economics Research Unit,<br>Bai, Annie; Canadian Agency for Drugs and Technologies in Health,<br>Moulton, Kristen; Canadian Agency for Drugs and Technologies in Health,<br>Clifford, Tammy; University of Ottawa, Department of Epidemiology and Community Medicine; University of Ottawa, Department of Pediatrics<br>Wells, George ; University of Ottawa, Department of Epidemiology and Community Medicine; University of Ottawa Heart Institute,

**Primary Subject Heading**:	Cardiovascular medicine
Secondary Subject Heading:	Epidemiology, Health policy
Keywords:	Stroke < NEUROLOGY, CARDIOLOGY, EPIDEMIOLOGY

SCHOLARONE™ Manuscripts

Systematic Review and Network Meta-analysis comparing Antithrombotic Agents for the Prevention of Stroke and Major Bleeding in Patients with Atrial Fibrillation

Chris Cameron, PhD Student[1,2], Doug Coyle, Professor[1], Trevor Richter, Manager of Clinical Research [3], Shannon Kelly, Research coordinator[2] , Kasandra Gauthier, Clinical Research Officer[3], Sabine Steiner, Associate Professor[4], Marc Carrier, Associate professor[6], Kathryn Coyle, Health Economist[5], Annie Bai , Clinical Research Officer[3], Kristen Moulton, Clinical Research Officer[3], Tammy Clifford, Adjunct Professor[1,7], George Wells, Professor[1,2]

[1] Department of Community Medicine and Epidemiology, University of Ottawa, Canada
[2] University of Ottawa Heart Institute, Ottawa, Canada
[3] Canadian Agency for Drugs and Technologies in Health, Ottawa, Canada
[4] Medical University of Vienna, Vienna, Austria
[5] Applied Health Economics Research Unit, Ottawa, Canada
[6] Thrombosis Program, Division of Hematology, Department of Medicine, University of Ottawa, Ottawa, Canada
[7] Department of Pediatrics, University of Ottawa, Ottawa, Canada

For peer review only

Word count, abstract: 299
Word count, body of essay: 4,194
Number of figures: 4
Number of tables: 1
Number of References: 41
Supplementary Material: 15

Corresponding author:
Dr. George Wells
Professor and Director, Cardiovascular Research Methods Centre
Department of Community Medicine and Epidemiology, University of Ottawa
Ottawa Heart Institute
40 Ruskin Street, Ottawa, Ontario, Canada K1Y 4W7
Phone: (613) 798-5555, ext. 18640
Fax: (613) 761-4412
Email: gawells@ottawaheart.ca

Keywords: atrial fibrillation, antithrombotic, anticoagulants, stroke, bleeding

Abstract

Objective: To examine the comparative efficacy and safety of antithrombotic treatments (dabigatran, rivaroxaban, apixaban, edoxaban, and vitamin K antagonists (VKA) at a standard adjusted dose, acetylsalicylic acid, acetylsalicylic acid and clopidogrel) for non-valvular atrial fibrillation and among sub-populations.

Design: Systematic review and network meta-analysis.

Data sources: A systematic literature search strategy using MEDLINE, EMBASE, Cochrane Register of Controlled Trials, and grey literature including the websites of regulatory agencies for trials published in English from 1988 to January 2014.

Eligibility criteria for selecting studies: Randomized controlled trials were selected for inclusion if they were published in English, included at least one antithrombotic treatment and involved patients with non-valvular atrial fibrillation eligible to receive anticoagulant therapy.

Results: For stroke or systemic embolism, apixaban and dabigatran 150mg twice daily were associated with reductions relative to standard adjusted dose VKA whereas low-dose ASA and the combination of clopidogrel plus low-dose ASA were associated with increases. Absolute risk reductions ranged from 6 fewer events per 1,000 patients treated for dabigatran 150mg twice daily to 15 more events for clopidogrel plus ASA. For major bleeding, apixaban, dabigatran 110mg twice daily, edoxaban 30mg daily, and edoxaban 60mg daily were associated with reductions compared to standard adjusted dose VKA. Absolute risk reductions with these agents ranged from 18 fewer per 1,000 patients treated each year for edoxaban 30mg daily to 23 more for medium dose ASA.

Conclusions: Compared with standard adjusted dose VKA, new oral anticoagulants were associated with modest reductions in the absolute risk of stroke and major bleeding. Antiplatelet drugs were associated with more strokes compared with anticoagulant drugs without any reduction in bleeding risk. To fully elucidate the comparative benefits and harms of antithrombotic agents across the various sub-populations, rigorously conducted comparative studies or network meta-regression analyses of patient-level data are required.

Registration: PROSPERO-CRD42012002721

For peer review only - http://bmjopen.bmj.com/site/about/guidelines.xhtml

Article Summary

Strengths and limitations of this study

- This network meta-analysis includes data on edoxaban from the recently published ENGAGE AF-TIMI 48 trial
- The studies pooled in this meta-analysis include antiplatelet agents in addition to newer oral anticoagulants and standard adjusted dose vitamin K antagonists
- We present findings on both the relative and absolute scale, and graphically illustrate absolute risks using icon arrays
- We report detailed *a priori* sub-group analyses for stroke or systemic embolism and major bleeding using data derived from FDA Public Summary Documents
- Comparisons between antithrombotic treatments were largely comprised of single studies, albeit large studies
- There is notable heterogeneity and the small number of studies limits the analyses that can be conducted to account for heterogeneity in the absence of patient-level data

For peer review only

INTRODUCTION

Atrial fibrillation (AF) is a common cardiac arrhythmia associated with increased morbidity and mortality.[1,2] Patients with AF sustain an increased risk of arterial thromboembolism and stroke, associated with high recurrence and substantial debilitating impact. Therefore, antithrombotic strategies using anticoagulant drugs and antiplatelet agents are recommended for AF patients presenting with risk factors for stroke. Antithrombotic therapy is also associated with a risk of bleeding; therefore, the beneficial effects on stroke prevention should always be compared against a patient's risk of major bleeding.[1,2]

Existing guidelines recommend anticoagulant therapy for patients at intermediate or high risk of stroke.[1,2] Although standard adjusted dose vitamin K antagonists (VKA) (e.g., warfarin) has been the cornerstone treatment for reducing the risk of stroke or systemic embolism (SE) in this population, it is associated with several drawbacks.[2] which have prompted the development of newer oral anticoagulants such as direct thrombin (dabigatran) and Xa (e.g. apixaban, rivaroxaban, edoxaban) inhibitors. [1,2] While some of these newer treatment options have been demonstrated to be efficacious in preventing stroke or SE compared to standard adjusted dose VKA, the relative efficacy and risk of bleeding of newer oral anticoagulants, both among themselves and in comparison to antiplatelet agents, is not clear, especially in certain sub-populations of patients, e.g., $CHADS_2$ score <2 or ≥2; time in therapeutic range (TTR) < 66% or ≥ 66%; Age <75 or ≥ 75 years. Therefore, the objective of this paper was to compare antithrombotic agents for the prevention of stroke and major bleeding in patients with non-valvular AF and among sub-populations.

For peer review only - http://bmjopen.bmj.com/site/about/guidelines.xhtml

METHODS

Published literature was identified by searching MEDLINE, MEDLINE In-Process & Other Non-Indexed Citations, EMBASE, BIOSIS Previews, PubMed and the Cochrane Central Register of Controlled Trials were searched through the Ovid interface to identify English-language clinical articles published from 1988 to January 23, 2014. Regular alerts were also established. Websites of regulatory agencies, Canadian and major international health technology assessment agencies, clinical practice guidelines as well as The Cochrane Library (2012, Issue 6) and University of York Centre for Reviews and Dissemination databases were searched. Complete details of the electronic search strategy, including any limits used, are reported in Appendix 1. The protocol was published online and was registered in the PROSPERO International Prospective Register of Systematic Reviews (PROSPERO registry - CRD42012002721).

The population of interest was individuals with non-valvular AF requiring anticoagulation (including all risk levels and regardless of any comorbidities). The following treatments were included in the review: apixaban, dabigatran, edoxaban, rivaroxaban, standard adjusted dose VKA, acetylsalicylic acid (ASA), ASA and clopidogrel. Numerous outcomes were considered and results are reported elsewhere; however, the focus of this publication is the outcomes of all-cause stroke or SE and major bleeding as defined by the International Society on Thrombosis and Haemostasis [ISTH] definition),[3] which were the primary efficacy and safety outcomes used in the newer oral anticoagulant studies.

For peer review only - http://bmjopen.bmj.com/site/about/guidelines.xhtml

Active and placebo-controlled randomized controlled trials (RCTs) were selected for inclusion if they were published in English, included at least one antithrombotic treatment under review (using pre-specified doses), reported any of the pre-specified outcomes related to patient safety or clinical efficacy, and involved patients with non-valvular AF eligible to receive anticoagulant therapy, regardless the level of stroke risk. Trials that included patients with contraindication to anticoagulant treatment were excluded. VKA trials were included if the dose was adjusted to a target international normalised ratio (INR) 2.0-3.0. Any dose of ASA was considered for inclusion, but ASA dose was stratified in the analysis as low (≤ 100 mg daily), medium (>100 mg to ≤ 300 mg daily), or high (>300 mg daily). Three reviewers independently extracted the data on baseline characteristics, intervention(s) evaluated, including dose, duration, and relevant co-medication and results for each included article, using a standardized template. All extracted data was checked for accuracy by three independent reviewers. Any disagreements in the assessment of these data were resolved through discussion until consensus was reached. Quality assessment of RCTs was also performed independently by two reviewers using a standardized table based on major items from the SIGN 50 instrument for internal validity.[4] The trial selection process is presented in a flowchart based on the Preferred Reporting Items for Systematic Reviews and Meta-Analyses (PRISMA)[5] statement (Appendix 2).

Bayesian network meta-analyses were conducted using WinBUGS software (MRC Biostatistics Unit, Cambridge, UK). A binomial likelihood model[6] which accounts for the use of multi-arm trials was used for the analyses given the datasets provided were dichotomous outcomes and included multi-arm trials. Trials with zero cells in both arms or nodes where there were no events were excluded from the evidence networks because they do not contribute information or allow

For peer review only - http://bmjopen.bmj.com/site/about/guidelines.xhtml

interpretable information.[6] Both fixed and random-effects network meta-analyses were conducted, although the fixed-effects model was chosen for the reference case analysis, as the evidence network was largely comprised of single study connections. We modeled point estimates and 95% credible intervals for odds ratios (OR) using Markov Chain Monte Carlo methods. The absolute risk difference per 1000 patients treated each year for each outcome was also calculated, based on the standard adjusted dose VKA arm of the Randomized Evaluation of Long-Term Anti-coagulation Therapy (RE-LY) trial.[7] The RE-LY trial[7] was selected because it contained data for both $CHADS_2<2$ and $CHADS_2 \geq 2$ and had detailed data available from the Food and Drug Administration (FDA) Public Summary Report.[8] Vague or flat priors, such as $N(0, 100^2)$ were assigned for basic parameters throughout, although informative priors were considered.[9] Assessment of model fit was based on deviance information criterion (DIC) and comparison of residual deviance to number of unconstrained data points.[6,10] To ensure convergence was reached, trace plots and the Brooks-Gelman-Rubin statistic were assessed.[11] Three chains were fit in WinBUGS for each analysis, with at least 40,000 iterations, and a burn-in of at least 40,000 iterations. We also qualitatively compared the results from our network meta-analysis with direct pair-wise estimates.

A key assumption behind network meta-analysis is that the analysed network is consistent, i.e., there is no conflict between direct and indirect evidence.[12] To assess inconsistency, we compared deviance and DIC statistics in fitted consistency and inconsistency models.[12] We also plotted the posterior mean deviance of the individual data points in the inconsistency model against their posterior mean deviance in the consistency model to identify any loops in where inconsistency is

For peer review only - http://bmjopen.bmj.com/site/about/guidelines.xhtml

present (Appendix 11).[12] Additionally, the results from our network meta-analysis were qualitatively compared with direct frequentist pair-wise estimates.

Network meta-analysis also requires that studies are sufficiently similar in order to pool their results.[13] Available study and patient characteristics were assessed to ensure similarity and to investigate the potential impact of heterogeneity on effect estimates. Clinical and methodological heterogeneity was identified in a number of areas. The issues identified were similar to those reported in previous systematic reviews of anticoagulant drugs (e.g., differences in TTR, $CHADS_2$ score).[14] Heterogeneity was assessed by conducting network meta-analysis with the following pre-specified subgroup data reported in the individual RCTs: TTR $< 66\%$ or $\geq 66\%$; $CHADS_2$ score <2 or ≥ 2; Age <75 or ≥ 75 years.[14] The data to conduct these sub-group analyses were derived from FDA Public Summary Documents when not available in the publications.[8,15,16] Secondary evidence networks were constructed for each of the sub-group analyses. The WinBUGS code[6] and data needed to replicate all analyses are available online.

This research was supported through a grant by the Canadian Institute of Health Research (CIHR) Drug Safety and Effectiveness Network and financial support from the Canadian Agency for Drugs and Technologies in Health (CADTH).

RESULTS

Study Characteristics of included studies

The systematic review included 13 individual RCTs reported in 29 publications and FDA reports (Appendix 2); all evaluated the efficacy and safety of antithrombotic agents: dabigatran (150 or

For peer review only - http://bmjopen.bmj.com/site/about/guidelines.xhtml

110 mg twice daily), rivaroxaban (20 mg daily), apixaban (5 mg twice daily), edoxaban (30 or 60 mg daily), standard adjusted dose VKA, ASA (low dose (< 100 mg daily); medium dose (100-300 mg daily), or low dose-ASA plus clopidogrel (75 mg daily) in patients with non-valvular AF. None of the included studies directly compared one new oral anticoagulant drug with another (Appendix 4). Of the 81,677 randomized patients included in this review, five large multicentre trials account for 78,296 patients (96%): ENGAGE AF-TIMI 48 (n=21,105),[17] ARISTOTLE (n=18,201),[18] RE-LY (n=18,113),[7] ROCKET-AF (n=14,264)[19] and ACTIVE-W (n=6706).[20] Most trials included stroke and SE as a primary efficacy outcome, while bleeding events were a frequent safety outcome. Follow-up ranged from 12 weeks to 3.5 years. All trials published results between 1989 and 2013. Mean $CHADS_2$ score was reported in six trials, encompassing the vast majority of patients included in the systematic review. Reported $CHADS_2$ scores were consistent with a high-risk population ($CHADS_2 \geq 2$), with patients from ROCKET-AF[19] showing the highest risk for stroke (mean $CHADS_2 = 3.5$).

Mean age across the included trials ranged from 65 years to 83 years (Appendix 3). All trials included patients of both gender and had a higher proportion of male participants in most studies. However, the proportions of patients with a prior stroke or TIA varied substantially across the included trials, ranging from 3% in JAST to 55% in ROCKET-AF.[19] The same observation can be made regarding the proportions of patients with other concomitant conditions, i.e. congestive heart failure, hypertension, diabetes infarction, and prior vitamin K antagonist experience. TTR was reported in all but one trial that included a standard adjusted dose VKA treatment arm. Patient baseline characteristics are summarized in Appendix 3.

For peer review only - http://bmjopen.bmj.com/site/about/guidelines.xhtml

The studies were individually critically appraised and the details are reported elsewhere. Overall, there was substantial variation in study quality. However, large multicentre trials comparing newer anticoagulants with standard adjusted dose VKA such as ENGAGE AF-TIMI 48 (n=21,105), ARISTOTLE (n = 18,201), RE-LY (n = 18,113), and ROCKET-AF (n = 14,264), which account for the vast majority of patients included in the systematic review, appear to be methodologically rigorous.

Stroke or Systemic Embolism

The evidence network (Figure 1) for the primary analysis was comprised of eleven RCTs representing eight treatments in addition to placebo/observation (N =81,677). Two RCTs (PETRO,[21] WASPO[22]) were not included in the analysis because they did not report data for this outcome or had zeroes in both arms..

Apixaban and dabigatran (150 mg twice daily) were associated with reductions in stroke or SE relative to standard adjusted dose VKA (Figure 2). The use of these two agents led to absolute risk reductions ranging from 4 to 6 fewer events per 1,000 patients treated each year. By contrast, low-dose ASA and the combination of clopidogrel plus low-dose ASA appeared to have a higher risk of stroke or SE than standard adjusted dose VKA, leading to an increase in the number of stroke or SE ranging from 14 to 15 more events per 1,000 patients treated each year (Figure 4 and Appendix 6). No differences were detected between standard adjusted dose VKA and each of the following interventions: rivaroxaban, edoxaban (30 mg or 60 mg daily), dabigatran (110 mg twice daily), and medium-dose ASA (Figure 3).

For peer review only - http://bmjopen.bmj.com/site/about/guidelines.xhtml

The estimates of effect derived from the direct pair-wise comparisons aligned well with those obtained from the network meta-analysis in both direction and magnitude (Appendix 6). Furthermore, the posterior mean residual deviance (26.81) is close to the number of unconstrained data points (24), which is an indication of reasonable model fit. A number of sub-group analyses and alternative modeling strategies were conducted on the primary analysis, including adjusting for $CHADS_2$, TTR, and age (Table 1). We also conducted a sensitivity analysis where we included the AVERROES[23] and ACTIVE A[24] trial (Appendix 12). These studies were excluded from the main analysis because we required patients to be eligible for anticoagulant therapy, including treatment with a VKA (Appendix 14). The results differed slightly from the reference case (Appendix 12). The primary analysis was conducted using a fixed-effects model because the network was comprised of single study connections; these results were also compared against those obtained using a random-effects model with vague and informative priors on the standard deviation, and found to be similar in magnitude although wider credible intervals were observed (Appendix 9).

Major bleeding

The evidence network for the primary analysis for major bleeding was comprised of 12 RCTs representing eight treatments in addition to placebo/observation (N =81,677). The evidence network for major bleeding is similar to the evidence network for stroke or SE (Figure 1) but includes one extra RCT (WASPO[22]) comparing medium dose ASA with standard adjusted dose VKA (Appendix 5).

For peer review only - http://bmjopen.bmj.com/site/about/guidelines.xhtml

Apixaban,dabigatran 110 mg twice daily, edoxaban 30 mg daily, and edoxaban 60 mg daily were associated with reductions in the risk of major bleeding compared with standard adjusted dose VKA (Figure 2). No differences for major bleeding were detected between standard adjusted dose VKA and each of the remaining interventions: rivaroxaban, dabigatran 150 mg twice daily, clopidogrel plus low-dose ASA, and all ASA dosages (Figure 2). The absolute risk difference of major bleeding relative to standard adjusted dose VKA ranged from 18 fewer to 24 more events per 1000 patients treated per year (Figure 4 and Appendix 7). A summary of the results for the Bayesian and direct pair-wise meta-analyses is shown in Appendix 7, and Bayesian network meta-analysis results are represented graphically in Figure 2. Complete results from the Bayesian network meta-analysis for all possible comparisons are presented in Figure 3**.**

The estimates of effect derived from the direct pair-wise comparisons aligned well with those obtained from the network meta-analysis in both direction and magnitude (Appendix 7). Furthermore, the posterior mean residual deviance (27.1) is close to the number of unconstrained data points (26), which is an indication of reasonable model fit. A number of sub-group analyses and alternative modeling strategies were conducted on the primary analysis (Table 1). We also conducted a sensitivity analysis where we included the AVERROES[23] and ACTIVE A[24] trial (Appendix 12), as well as analyses using a random-effects model with vague and informative priors (Appendix 9).

Benefit-Harm Assessment – Stroke or systemic embolism versus major bleeding

Figure 3 summarises the results for all pair-wise comparisons derived from the Bayesian fixed-effects network meta-analysis (Appendix 9). Results relative to standard adjusted dose VKA

For peer review only - http://bmjopen.bmj.com/site/about/guidelines.xhtml

have been discussed above. For pair-wise comparisons among newer oral anticoagulants for stroke or SE, dabigatran 150 mg twice daily was associated fewer events versus dabigatran 110 mg twice daily, rivaroxaban, edoxaban 30mg daily, and edoxaban 60 mg daily. Apixaban, and rivaroxaban were also associated with fewer events compared to edoxaban 30mg daily. There were no differences among low-dose ASA, medium-dose ASA, and clopidogrel plus low-dose ASA. However, low-dose ASA and the combination of clopidogrel plus low-dose ASA were associated with increases in stroke or systemic embolism compared to all newer oral anticoagulants. For major bleeding, apixaban, dabigatran 110 mg twice daily were associated with fewer events versus dabigatran 150 mg twice daily and rivaroxaban. Edoxaban 30 mg daily was associated with fewer events than other new oral anticoagulants, while edoxaban 60 mg daily was associated with fewer events compared with rivaroxaban and clopidogrel plus low-dose ASA. The risk of major bleeding for apixaban was lower compared to clopidogrel plus low-dose ASA. There were no differences associated with the ASA treatments, both for comparisons among themselves and compared to the anticoagulant treatments.

Figure 4 plots the absolute risk for stroke or SE and major bleeding per 1000 treated for the 10 treatments analyzed. Data in Figure 4 were obtained from the results presented in Appendix 6 and 7. Examination of the data in Figure 4 suggests that the balance of benefits and harms of the new oral anticoagulants for stroke or systemic embolism and major bleeding is positive compared to standard adjusted dose VKA (decrease in stroke or systemic embolism and/or major bleeding) and largely similar among one another. Comparison of all anticoagulant treatment (including standard adjusted dose VKA) to ASA, or ASA in combination with clopidogrel indicates that whereas the new oral anticoagulants decrease the risk of both stroke or systemic

For peer review only - http://bmjopen.bmj.com/site/about/guidelines.xhtml

embolism and major bleeding, ASA has a less favorable balance of benefits and harms, i.e., fails to minimize the risk of stroke or systemic embolism and/or major bleeding.

DISCUSSION

We identified 13 RCTs comparing antithrombotic agents for the prevention of all cause stroke or systemic embolism and major bleeding in patients with AF. To the best of our knowledge, this is the most up-to-date systematic review and network meta-analysis[25–29] to synthesize the available benefits and harms on newer oral anticoagulants, standard adjusted dose VKA, and antiplatelet agents for the prevention of stroke and major bleeding in patients with non-valvular AF (Appendix 13). Harenberg et al[25] recently summarized the published indirect comparisons and network meta-analyses evaluating newer oral anticoagulants for AF and an earlier unpublished version of our analysis[14] was considered among the most comprehensive analyses at that time.[25] This network meta-analysis expands upon that unpublished report.[14] Overall, our findings align with those reported in previously published network meta-analyses;[25,26,28] however, unlike previous analyses,[25] we include recently published edoxaban data,[17] and also include antiplatelet agents. We also present detailed evidence networks[30] to illustrate the body of evidence for each outcome; report analysis using multiple statistical models (Appendix 9); report detailed *a priori* sub-group analyses for each outcome using data derived from FDA Public Summary Documents;[8,15] present findings on both the relative and absolute scale,[31,32] and graphically illustrate results using icon arrays. Our network meta-analysis is also the only one published to date that was registered in PROSPERO, and also adheres to PRISMA reporting standards (Appendix 10). It also differs from a recently published traditional meta-analysis by Ruff et al[27]

For peer review only - http://bmjopen.bmj.com/site/about/guidelines.xhtml

given we report comparison of newer oral anticoagulants versus each other, and also include comparisons with antiplatelet agents.

For stroke or systemic embolism, apixaban and dabigatran 150 mg twice daily were associated with reductions in stroke or systemic embolism relative to standard adjusted dose VKA. By contrast, low-dose ASA and the combination of clopidogrel plus low-dose ASA appeared were associated with increases in stroke or systemic embolism relative to standard adjusted dose VKA. No differences were detected between standard adjusted dose VKA and each of the following interventions: rivaroxaban, dabigatran 110 mg twice daily, edoxaban 30 and 60 mg daily, medium-dose ASA, and no treatment/placebo. The absolute risk reduction of stroke or systemic embolism relative to standard adjusted dose VKA ranged from 6 fewer to 15 more events per 1000 patients treated per year, with dabigatran 150 mg twice daily yielding the largest aboslute risk reduction and clopidogrel plus low-dose ASA the largest absolute risk increase.

For major bleeding, apixaban, dabigatran 110 mg twice daily, edoxaban 30 mg daily, and edoxaban 60 mg daily were associated with an increased risk of major bleeding relative to standard adjusted dose VKA. No differences for major bleeding were detected between standard adjusted dose VKA and each of the remaining interventions: rivaroxaban, dabigatran 150 mg twice daily, clopidogrel plus low-dose ASA, and all ASA dosages. The absolute risk difference of major bleeding relative to standard adjusted dose VKA ranged from 18 fewer to 24 more events per 1000 patients treated, with edoxaban 30 mg daily yielding the largest absolute risk reduction and medium dose ASA the largest absolute risk increase. Results of network meta-analyses reflect the relative efficacy and safety profile of antiplatelet and anticoagulant drugs as

For peer review only - http://bmjopen.bmj.com/site/about/guidelines.xhtml

documented by large clinical trials. Thus, international guidelines[1,2] for prevention of stroke in patients with AF also recommended oral anticoagulation over antiplatelet therapy as the latter had only modest protective effects. With respect to the new oral anticoagulants none of the guidelines has given a recommendation to prefer one of the new oral anticoagulants over another.[1,2]

Network meta-analysis involves pooling of trials. We observed variability across studies included in the evidence network in terms of $CHADS_2$, age and TTR (Appendix 3 and 4). For example, patients using rivaroxaban had a higher risk of stroke because ROCKET-AF[19] included patients with $CHADS_2 \geq 2$. As such, we conducted sub-group analysis to adjust for differences in patient and study-level characteristics. We derived data for sub-group analyses from FDA Public Summary documents[8,15,16] which enhanced our ability to account for variability across studies (e.g., use intention to treat (ITT) population for rivaroxaban versus per-protocol as-treated population reported in publication[19]). Results from these sub-group analyses differed slightly from those reported in the primary analysis, indicating that there may be differences in the benefits and harms across sub-groups. This becomes more apparent when sub-group analyses are reported on the absolute scale (reported elsewhere). It is unclear whether the absolute risk reductions associated with these differences translate into clinically meaningful benefits in practice, especially in sub-populations of patients with a lower baseline risk of stroke or systemic embolism. To fully elucidate the comparative benefits and harms of antithrombotic agents across the various sub-populations, rigorously conducted comparative studies,[33,34] or network meta-regression analyses of patient-level data[35] are required.

For peer review only - http://bmjopen.bmj.com/site/about/guidelines.xhtml

There are several limitations with the analysis we employed. First, there is notable heterogeneity in patients and study characteristics (Appendix 3, 4, and 15). For example, there were differences in patients enrolled in the underlying studies and completion rates. However, the small number of studies limits the analyses that can be conducted to account for heterogeneity in the absence of patient-level data. Second, there is insufficient sub-group data available for other outcomes reported in the RCTs included. As a consequence, detailed analysis was limited to two outcomes – stroke and SE and major bleeding. Third, we reported the results from the fixed-effects model in the main text. We felt that this was appropriate as the nodes in evidence networks are connected largely by single studies. Effect estimates derived from the fixed-effects model aligned more closely with the direct estimates. Reporting results from the fixed-effects model in the main text likely biases results in favour of the new oral anticoagulants, as treatments that achieved statistically significant results in the primary RCTs retained statistically significant findings when using the fixed-effects model, but not always when using the random-effects model due to the prior[9] on the between study variance. Nevertheless, the results from the random-effects model have also been reported in Appendix 9, along with detailed discussion around the limitations in using the fixed-effects model. Fourth, the absolute risk reduction for the network meta-analysis was calculated using the event rate in the standard adjusted dose VKA arm of the RE-LY study. The RE-LY trial was selected because it contained data for both $CHADS_2 < 2$ and $CHADS_2 \geq 2$ and had detailed data made publicly available following an FDA review of dabigatran. Use of a different study (e.g., ARISTOTLE) for baseline event rate data is unlikely to change the results substantially given that the event rates in standard adjusted dose VKA arms were similar across studies. Fifth, we present findings using graphics that have been largely applied in the network meta-analysis literature,[36] such as a table with odds ratios for all

For peer review only - http://bmjopen.bmj.com/site/about/guidelines.xhtml

pair-wise comparisons. This method of presenting findings on the relative scale is problematic when you are conducting assessments to compare the balance of benefits and harms, which need to be standardized. As such, we also provided results in absolute terms. We report our findings using icon arrays, although it should be noted that these results do not account for the utility values based upon patient preferences for each of the outcomes,[37,38] nor do they reflect uncertainty although we report absolute reductions on the risk benefit plane reflecting uncertainty in Appendix 8. There are other methods available that can incorporate patient preferences for outcomes.[37,38] Further, estimates of benefit and harm with several of the therapies (e.g., rivaroxaban) come from one trial and thus such data are not particularly robust. Further research is needed comparing the balance of benefit and harms using other research methodologies, incorporating other relevant outcomes, patient preferences for each of the outcomes,[37,38] and additional studies when they become available. Finally, haemorrhagic stroke is also considered a major bleed in underlying studies. Accordingly, we may have double counted haemorrhage stroke in this analysis. Unfortunately, we are not able to account for this issue in all included studies. Nonetheless, it is important to note this limitation and highlight that this potentially biases results in favor of newer oral anticoagulants given these agents were associated with reductions in these events.

CONCLUSIONS

Compared with standard adjusted dose VKA, apixaban and dabigatran 150 mg twice daily were associated with reductions in the incidence of stroke or systemic embolism in patients with AF, whereas, low-dose ASA and the combination of clopidogrel plus low-dose ASA increased the risk relative to standard adjusted dose VKA at preventing stroke or systemic embolism.

For peer review only - http://bmjopen.bmj.com/site/about/guidelines.xhtml

Apixaban, dabigatran 110 mg twice daily, edoxaban 30 mg daily, and edoxaban 60 mg daily and was associated with a reduced risk in major bleeding compared with standard adjusted dose VKA. Although the results of the current review revealed that there were differences in stroke or systemic embolism and major bleeding among antithrombotic agents in the management of patients with AF in relative terms, it is unclear whether the absolute risk reductions associated with these differences translate into clinically meaningful benefits in practice, especially in sub-populations of patients with a lower baseline risk of stroke or systemic embolism. Rigorously conducted comparative studies or network meta-regression analyses of patient-level data are required to fully elucidate the comparative benefits and harms of antithrombotic agents across the various sub-populations.

For peer review only - http://bmjopen.bmj.com/site/about/guidelines.xhtml

TABLES

Table 1: Sub-group and sensitivity analyses for stroke or systemic embolism and major bleeding relative to standard adjusted dose VKA, Odds ratios ± 95% Credible Intervals versus standard adjusted dose VKA

Treatment	Reference Case – Fixed Effect NMA	Subgroup analysis by $CHADS_2$ Score		Subgroup analysis by Age		Subgroup analysis by TTR	
		$CHADS_2 < 2$	$CHADS_2 \geq 2$	Age < 75 years	Age ≥ 75 years	TTR < 66%	TTR ≥ 66%
**Stroke or Systemic Embolism**							
**Apixaban 5 mg twice daily**	**0.79 (0.66,0.95)**	0.86 (0.57,1.28)	**0.78 (0.63,0.95)**	0.85 (0.67,1.07)	**0.71 (0.53,0.95)**	0.79 (0.62,1.01)	0.79 (0.59,1.05)
**Dabigatran 110 mg twice daily**	0.91 (0.74,1.12)	1 (0.64,1.55)	0.89 (0.71,1.12)	0.93 (0.7,1.23)	0.88 (0.66,1.18)	0.91 (0.69,1.19)	0.9 (0.66,1.24)
**Dabigatran 150 mg twice daily**	**0.66 (0.53,0.82)**	0.61 (0.37,1)	**0.66 (0.52,0.85)**	0.63 (0.46,0.87)	**0.66 (0.48,0.9)**	0.54 (0.39,0.73)	0.81 (0.58,1.11)
**Rivaroxaban 20 mg daily**	0.88 (0.74,1.03)	NA	0.88 (0.74,1.04)	0.95 (0.75,1.2)	0.8 (0.63,1.02)	0.81 (0.65,1.01)	0.72 (0.47,1.1)
**Low Dose ASA (≤ 100 mg daily)**	**1.87 (1.27,2.8)**	2.2 (1.18,4.25)	2.1 (0.91,5.15) *	NA	2.04 (1.32,3.19)	NA	NA
**Medium Dose ASA (> 100 mg and ≤ 300 mg daily)**	1.35 (0.75,2.46)	1.97 (0.63,6.73) **	NA	NA	NA	NA	NA
**Clopidogrel 75 mg daily & Low dose Aspirin (≤ 100 mg daily)**	1.93 (1.42,2.65)	3.16 (1.39,8.22)	1.15 (0.84,1.57)	NA	NA	1.52 (0.96,2.45)	**2.4 (1.59,3.71)**
**Edoxaban 30 mg daily**	1.14(0.99,1.33)	NA	1.14(0.99,1.33)	NA¶	NA¶	NA¶	NA¶
**Edoxaban 60 mg daily**	0.87(0.74,1.02)	NA	0.87(0.74,1.02)	NA¶	NA¶	NA¶	NA¶
**Major Bleeding**							
**Apixaban 5 mg twice daily**	**0.69 (0.6,0.8)**	**0.59 (0.44,0.78)**	**0.73 (0.62,0.87)**	**0.73 (0.6,0.89)**	**0.65 (0.52,0.81)**	**0.57 (0.45,0.71)**	**0.81 (0.67,0.98)**
**Dabigatran 110 mg twice daily**	**0.8 (0.69,0.93)**	**0.65 (0.47,0.89)**	0.86 (0.73,1.02)	**0.61 (0.49,0.77)**	1.02 (0.84,1.25)	0.74 (0.61,0.91)	0.86 (0.69,1.06)
**Dabigatran 150 mg twice daily**	0.93 (0.8,1.08)	0.77 (0.57,1.04)	1.01 (0.86,1.19)	**0.7 (0.56,0.86)**	1.19 (0.98,1.45)	0.76 (0.62,0.94)	1.15 (0.94,1.4)
**Rivaroxaban 20 mg daily**	1.03 (0.89,1.19)	NA	1.03 (0.89,1.19)	0.93 (0.76,1.13)	1.15 (0.93,1.41)	0.92 (0.77,1.1)	**1.3 (1.01,1.69)**
**Low Dose ASA (≤ 100 mg daily)**	1.05 (0.6,1.86)	0.83 (0.42,1.64)	1.6 (0.55,5.02) *	NA	1.01 (0.57,1.79)	NA	NA
**Medium Dose ASA (> 100 mg and ≤ 300 mg daily)**	1.78 (0.62,5.59)	1.48 (0.11,19.07)**	NA	NA	NA	NA	NA

For peer review only - http://bmjopen.bmj.com/site/about/guidelines.xhtml

Treatment	Reference Case – Fixed Effect NMA	Subgroup analysis by CHADS $_2$ Score		Subgroup analysis by Age		Subgroup analysis by TTR	
		CHADS $_2$ < 2	CHADS $_2 \geq 2$	Age < 75 years	Age ≥ 75 years	TTR < 66%	TTR ≥ 66%
**Clopidogrel 75 mg daily & Low dose Aspirin (≤ 100 mg daily)**	1.1 (0.83,1.46)	1.51 (0.89,2.61)	0.97 (0.69,1.36) †	NA	NA	0.66 (0.42,1.02)	1.66 (1.12,2.47)
**Edoxaban 30 mg daily**	0.47(0.4,0.54)	NA	0.47(0.4,0.54)	NA¶	NA¶	NA¶	NA¶
**Edoxaban 60 mg daily**	0.78(0.69,0.9)	NA	0.78(0.69,0.9)	NA¶	NA¶	NA¶	NA¶

ASA= acetylsalicylic acid; mg= milligram; NA=not available; NMA= network meta-analysis; OR=odds ratio; TTR= time in therapeutic range; VKA=vitamin K antagonist
* Results may be slightly biased against LDASA. Derived from BAFTA study where CHADS $_2$ subgroup data stratified by CHADS $_2$ 1-2 (vs. 0-1) and CHADS $_2$ 3-6 (vs. 2-6).
** Results may be slightly biased against medium-dose ASA. Based on study level results from CAFA and JAST studies. Although these studies consisted primarily of low-risk populations, some patients may have CHADS $_2$ scores greater than 2.
† Estimated from subgroup study by Healey et al 2008
¶ Raw data (i.e., total number of patients and patients who experienced an event for each sub-group) were not reported in ENGAGE AF-TIMI 48[17]

For peer review only

FIGURES

Figure 1: Evidence network for all cause stroke or systemic embolism. The width of the lines is proportional to the number of randomized controlled trials comparing each pair of treatments, and the size of each treatment node is proportional to the number of randomised participants (sample size). A dotted line indicates a three-arm randomized controlled trial and a black node indicates a node included in the analysis but not reported in the main text.

ASA= acetylsalicylic acid; mg= milligram; RCT= RCT = randomized controlled trial; VKA= vitamin K antagonist

Figure 2: Odds ratio for all cause stroke or systemic embolism (A) and major bleeding (B) in Bayesian network meta-analysis versus standard adjusted dose VKA.

CrI= credible interval; mg= milligram; VKA= vitamin K antagonist

Figure 3: Odds Ratios (OR) from network meta-analyses for stroke or systemic embolism and major bleeding for all pair-wise comparisons.* ORs for recurrence of stroke or systemic embolism are below the diagonal (row-defining treatment versus column-defining treatment) and those for major bleeding are above the diagonal and in blue (column-defining treatment versus row-defining treatment). To obtain ORs for comparisons in the opposite direction, reciprocals should be taken (e.g., the OR for standard dose warfarin compared with apixaban 5 mg twice daily for stroke or systemic embolism is 1/0.78=1.28). Significant results are in bold and underscored (and in larger font).

mg= milligram; VKA= vitamin K antagonist
* Results between individual treatments, especially newer oral anticoagulants, should be interpreted with caution given the limitations associated with using a fixed-effects model. See Appendix 9 for additional deta

Figure 4: Icon array illustrating the absolute risks of Stroke or systemic embolism (blue) and major bleeding episodes (red) per 1000 patients treated*
* Figures do not reflect uncertainty around effect estimates and there is more uncertainty (i.e., wider credible intervals) for low dose ASA, medium dose ASA, and clopidogrel plus low dose ASA (See Appendix 8). Underlying studies may also double count haemorrhage stroke.

For peer review only

For peer review only - http://bmjopen.bmj.com/site/about/guidelines.xhtml

For peer review only

4

Acknowledgments: The authors would like to acknowledge Carolyn Spry. She was the Information Specialist from CADTH on the review who conducted the search strategy.

Contributors: CC conceived the study design, performed the data extraction, analysis, and drafted the manuscript. DC and GW conceived the study design, helped with the data analysis, and reviewed the manuscript for important intellectual content. TR conceived the study design, performed the systematic review, data extraction, and reviewed the manuscript for important intellectual content. SK, SS, KG, AB, KM, KC performed the systematic review, data extraction, and reviewed the manuscript for important intellectual content. TC, MC, and KC conceived the study and reviewed the manuscript for important intellectual content. All authors have read and approved the final version of the manuscript. All authors agree to be accountable for all aspects of the work in ensuring that questions related to the accuracy or integrity of any part of the work are appropriately investigated and resolved.

Funding information: This research was supported through a grant by the Canadian Institute of Health Research (CIHR) Drug Safety and Effectiveness Network and financial support from the Canadian Agency for Drugs and Technologies in Health (CADTH). The CIHR Drug Safety and Effectiveness Network played no role in study design, collection, analysis, interpretation of data, writing of the manuscript, or in the decision to submit the paper for publication. They accept no responsibility for the contents. Employees from CADTH were involved in the study design, collection, analysis, interpretation of data, and writing of the manuscript. CADTH, funded by Canadian federal, provincial and territorial governments, is an independent, not-for-profit producer and broker of health technology assessments. Federal, provincial, and territorial health care decision-makers rely on our evidence-based information to make informed decisions about the effectiveness and efficiency of drugs and other health technologies.

Competing interests: All authors have completed the ICMJE uniform disclosure form at www.icmje.org/coi_disclosure.pdf (available on request from the corresponding author) and declare: no support from any organisation for the submitted work; CC is a recipient of a Vanier Canada Graduate Scholarship from the Canadian Institutes of Health Research and has received funding from Canadian Network and Centre for Trials Internationally (CANNeCTIN) and is a trainee on the CIHR Drug Safety and Effectiveness Network team grant. MC is a recipient of a New Investigator Award from the Heart and Stroke Foundation of Canada and holds a T2 research chair in cancer and thrombosis from the University of Ottawa.

Ethical approval: Not required.

Data sharing: The statistical code and dataset used for analyses are reported online: http://www.cadth.ca/media/pdf/TR0003_AntithromboticAgents-AF_ScienceReport-e.pdf. Additional data can be requested by emailing George Wells: gawells@ottawaheart.ca .

The Corresponding Author has the right to grant on behalf of all authors and does grant on behalf of all authors, an exclusive licence (or non exclusive for government employees) on a worldwide basis to the BMJ Publishing Group Ltd to permit this article (if accepted) to be published in BMJ editions and any other BMJPGL products and sublicences such use and exploit all subsidiary rights, as set out in our licence

For peer review only

REFERENCES

1. Kirchhof, P. *et al.* Atrial fibrillation guidelines across the Atlantic: a comparison of the current recommendations of the European Society of Cardiology/European Heart Rhythm Association/European Association of Cardiothoracic Surgeons, the American College of Cardiology Fou. *Eur. Heart J.* **34,** 1471–1474 (2013).

2. Skanes, A. C. *et al.* Focused 2012 update of the Canadian Cardiovascular Society atrial fibrillation guidelines: recommendations for stroke prevention and rate/rhythm control. *Can. J. Cardiol.* **28,** 125–36 (2012).

3. Schulman, S. & Kearon, C. Definition of major bleeding in clinical investigations of antihemostatic medicinal products in non-surgical patients. *J. Thromb. Haemost.* **3,** 692–4 (2005).

4. NHS Quality Improvement Scotland (NHS QIS). *SIGN 50. A guideline developer's handbook*. (2008). at <http://www.sign.ac.uk/pdf/sign50.pdf>

5. Moher, D., Liberati, A., Tetzlaff, J. & Altman, D. G. Preferred reporting items for systematic reviews and meta-analyses: the PRISMA statement. *PLoS Med.* **6,** e1000097 (2009).

6. Dias, S., Welton, N. J., Sutton, A. J. & Ades, A. *NICE DSU Technical Support Document 2: A Generalised Linear Modelling Framework for Pairwise and Network Meta-Analysis of Randomised Controlled Trials*. *Trop. Med.* 1–96 (2011). at <www.nicedsu.org.uk>

7. Connolly, S. & Ezekowitz, M. Dabigatran versus warfarin in patients with atrial fibrillation. *N. Engl. J. Med.* **361,** 1139–1151 (2009).

8. Ingelheim, B. *Advisory committee briefing document: dabigatran etexilate [Internet]*. 1–168 (2010). at <http://www.fda.gov/downloads/advisorycommittees/committeesmeetingmaterials/drugs/cardiovas>

9. Turner, R. M., Davey, J., Clarke, M. J., Thompson, S. G. & Higgins, J. P. Predicting the extent of heterogeneity in meta-analysis, using empirical data from the Cochrane Database of Systematic Reviews. *Int. J. Epidemiol.* **41,** 818–27 (2012).

10. Spiegelhalter, D. J., Best, N. G., Carlin, B. P. & van der Linde, A. Bayesian measures of model complexity and fit. *J. R. Stat. Soc. Ser. B (Statistical Methodol.* **64,** 583–639 (2002).

11. Spiegelhalter, D., Thomas, A. & Best, N. WinBUGS user manual. *MRC Biostat. Unit* **2,** (2004).

For peer review only - http://bmjopen.bmj.com/site/about/guidelines.xhtml

12. Dias, S., Welton, N.J., Sutton, A.J., Caldwell, D.M., Guobing, L. & Ades, A. E. *NICE Technical Support Document 4: Inconsistency in Networks of Evidence Based on Randomised Controlled Trials*. 1–39 (2011). at <www.nicedsu.org.uk>

13. Salanti, G., Marinho, V. & Higgins, J. P. T. A case study of multiple-treatments meta-analysis demonstrates that covariates should be considered. *J. Clin. Epidemiol.* **62,** 857–64 (2009).

14. Wells, G., Coyle, D., Cameron, C., Steiner, S. & Kelly, S. *Safety, effectiveness, and costeffectiveness of new oral anticoagulants compared with warfarin in preventing stroke and other cardiovascular events in patients with atrial fibrillation*. (2012). at <http://www.cadth.ca/media/pdf/NOAC_Therapeutic_Review_final_report.pdf>

15. Johnson & Johnson Pharmaceutical Research & Development. *Rivaroxaban for the Prevention of Stroke and Non-Central Nervous System (CNS) Systemic Embolism in Patients With Atrial Fibrillation*. **7939,** 222 (2011).

16. McDonagh, M. S., Peterson, K., Balshem, H. & Helfand, M. US Food and Drug Administration documents can provide unpublished evidence relevant to systematic reviews. *J. Clin. Epidemiol.* (2013). doi:10.1016/j.jclinepi.2013.05.006

17. Giugliano, R. P. *et al.* Edoxaban versus warfarin in patients with atrial fibrillation. *N. Engl. J. Med.* **369,** 2093–104 (2013).

18. Granger, C. & Alexander, J. Apixaban versus warfarin in patients with atrial fibrillation. *N. Engl. J. Med.* 981–992 (2011).

19. Patel, M., Mahaffey, K. & Garg, J. Rivaroxaban versus warfarin in nonvalvular atrial fibrillation. *N. Engl. J. Med.* 883–891 (2011).

20. Connolly, S. *et al.* Clopidogrel plus aspirin versus oral anticoagulation for atrial fibrillation in the Atrial fibrillation Clopidogrel Trial with Irbesartan for prevention of Vascular Events (ACTIVE W): a randomised controlled trial. *Lancet* **367,** 1903–12 (2006).

21. Ezekowitz, M. D. *et al.* Dabigatran with or without concomitant aspirin compared with warfarin alone in patients with nonvalvular atrial fibrillation (PETRO Study). *Am. J. Cardiol.* **100,** 1419–26 (2007).

22. Rash, A. *et al.* A randomised controlled trial of warfarin versus aspirin for stroke prevention in octogenarians with atrial fibrillation (WASPO). *Age Ageing* **36,** 151–6 (2007).

23. Connolly, S. & Eikelboom, J. Apixaban in patients with atrial fibrillation. *N. Engl. J. Med.* **364,** 806–817 (2011).

For peer review only - http://bmjopen.bmj.com/site/about/guidelines.xhtml

24. Connolly, S. J. *et al.* Effect of clopidogrel added to aspirin in patients with atrial fibrillation. *N. Engl. J. Med.* **360,** 2066–78 (2009).

25. Harenberg, J. & Weiss, C. Clinical trials with new oral anticoagulants. Additive value of indirect comparisons also named network meta-analyses. *Hamostaseologie* **33,** 62–70 (2013).

26. Dogliotti, A., Paolasso, E. & Giugliano, R. P. Current and new oral antithrombotics in non-valvular atrial fibrillation: a network meta-analysis of 79 808 patients. *Heart* (2013). doi:10.1136/heartjnl-2013-304347

27. Ruff, C. T. *et al.* Comparison of the efficacy and safety of new oral anticoagulants with warfarin in patients with atrial fi brillation□: a meta-analysis of randomised trials. *Lancet* **6736,** 1–8 (2013).

28. Assiri, A., Al-Majzoub, O., Kanaan, A. O., Donovan, J. L. & Silva, M. Mixed treatment comparison meta-analysis of aspirin, warfarin, and new anticoagulants for stroke prevention in patients with nonvalvular atrial fibrillation. *Clin. Ther.* **35,** 967–984.e2 (2013).

29. Kmh, B. S. & Berge, E. Factor Xa inhibitors versus vitamin K antagonists for preventing cerebral or systemic embolism in patients with atrial fibrillation (Review). (2013).

30. Salanti, Georgia, Kavvoura, F, Ioannidis, J. Exploring the geometry of treatment networks. *Ann. Intern. Med.* **148,** 544–553 (2008).

31. Barratt, A. *et al.* Tips for learners of evidence-based medicine: 1. Relative risk reduction, absolute risk reduction and number needed to treat. *Can. Med. Assoc. J.* **171,** 353–8 (2004).

32. Schechtman, E. Odds ratio, relative risk, absolute risk reduction, and the number needed to treat--which of these should we use? *Value Health* **5,** 431–6 (2002).

33. Berger, M. L., Mamdani, M., Atkins, D. & Johnson, M. L. Good research practices for comparative effectiveness research: defining, reporting and interpreting nonrandomized studies of treatment effects using secondary data sources: the ISPOR Good Research Practices for Retrospective Database Analysis Task Force . *Value Health* **12,** 1044–52 (2009).

34. Berger, M. L. *et al.* Prospective observational studies to assess comparative effectiveness: the ISPOR good research practices task force report. *Value Health* **15,** 217–30 (2012).

35. Jansen, J. P. Network meta-analysis of individual and aggregate level data. *Res. Synth. Methods* **3,** 177–190 (2012).

For peer review only

For peer review only - http://bmjopen.bmj.com/site/about/guidelines.xhtml

36. Salanti, G., Ades, A. & Ioannidis, J. Graphical methods and numerical summaries for presenting results from multiple-treatment meta-analysis: an overview and tutorial. *J. Clin. Epidemiol.* **64,** 163–71 (2011).

37. Guo, J. J. *et al.* A review of quantitative risk-benefit methodologies for assessing drug safety and efficacy-report of the ISPOR risk-benefit management working group. *Value Health* **13,** 657–66 (2010).

38. Boyd, C. *et al. Methods Research Report Methods for Benefit and Harm Assessment in Systematic Reviews*. 52 (2012). at <www.ncbi.nlm.nih.gov/books/NBK115750/>

39. Thorlund, K., Thabane, L. & Mills, E. J. Modelling heterogeneity variances in multiple treatment comparison meta-analysis – Are informative priors the better solution□? *BMC Med. Res. Methodol.* **13,** 1 (2013).

For peer review only

For peer review only - http://bmjopen.bmj.com/site/about/guidelines.xhtml

Systematic Review and Network Meta-analysis comparing Antithrombotic Agents for the Prevention of Stroke and Major Bleeding in Patients with Atrial Fibrillation

Chris Cameron, PhD Student[1,2], Doug Coyle, Professor[1], Trevor Richter, Manager of Clinical Research — Health Economics[3], Shannon Kelly, Research coordinator[2] , Kasandra Gauthier, Clinical Research Officer[3], Sabine Steiner, Associate Professor[4], Marc Carrier, Associate professor[6], Kathryn Coyle, Health Economist[5], Kristen Moulton, Clinical Research Officer[3], Annie Bai , Clinical Research Officer[3], Tammy Clifford, Adjunct Professor[1,7], George Wells, Professor[1,2]

[1] Department of Community Medicine and Epidemiology, University of Ottawa, Canada
[2] University of Ottawa Heart Institute, Ottawa, Canada
[3] Canadian Agency for Drugs and Technologies in Health, Ottawa, Canada
[4] Medical University of Vienna, Vienna, Austria
[5] Applied Health Economics Research Unit, Ottawa, Canada
[6] Thrombosis Program, Division of Hematology, Department of Medicine, University of Ottawa, Ottawa, Canada
[7] Department of Pediatrics, University of Ottawa, Ottawa, Canada

Word count, abstract: 29929900
Word count, body of essay: 4,1943,987
Number of figures: 4
Number of tables: 1
Number of References: 4136
Supplementary Material: 152

Corresponding author:
Dr. George Wells
Professor and Director, Cardiovascular Research Methods Centre
Department of Community Medicine and Epidemiology, University of Ottawa
Ottawa Heart Institute
40 Ruskin Street, Ottawa, Ontario, Canada K1Y 4W7
Phone: (613) 798-5555, ext. 18640
Fax: (613) 761-4412
Email: gawells@ottawaheart.ca

Keywords: atrial fibrillation, antithrombotic, anticoagulants, stroke, bleeding

For peer review only

Abstract

Objective: To examine the comparative ~~compare the~~ efficacy and safety of antithrombotic treatments ~~(~~dabigatran, rivaroxaban, apixaban, edoxaban, and vitamin K antagonists (VKA) at a standard adjusted dose, acetylsalicylic acid, acetylsalicylic acid and clopidogrel~~)~~ for non-valvular atrial fibrillation and among sub-populations.

Design: Systematic review and network meta-analysis.

Data sources: A systematic literature search strategy ~~was designed and carried out~~ using MEDLINE, EMBASE, Cochrane Register of Controlled Trials, and grey literature including the websites of regulatory agencies ~~and health technology assessment organizations .~~for trials published in English from 1988 to January 2014.

Eligibility criteria for selecting studies: Randomized controlled trials were selected for inclusion if they were published in English, included at least one antithrombotic treatment and involved patients with non-valvular atrial fibrillation eligible to receive anticoagulant therapy.

Results: For stroke or systemic embolism, apixaban and dabigatran 150mg twice daily were associated with reductions relative to standard adjusted dose VKA whereas low-dose ASA and the combination of clopidogrel plus low-dose ASA were associated with increases. Absolute risk reductions ranged from 6 fewer events per 1,000 patients treated for dabigatran 150mg twice daily to 154 more events for clopidogrel plus ASA. For major bleeding, apixaban, ~~and~~ dabigatran 110mg twice daily, edoxaban 30mg daily, and edoxaban 60mg daily were associated with reductions compared to standard adjusted dose VKA. Absolute risk reductions with these agents ranged from 18~~21~~ fewer per 1,000 patients treated each year for edoxaban 30mg daily ~~no treatment/placebo~~ to 23 more for medium dose ASA.

Conclusions: Compared with standard adjusted dose VKA, new oral anticoagulants were associated with modest reductions in the absolute risk of stroke and major bleeding. Antiplatelet drugs were associated with more strokes compared with anticoagulant drugs without any reduction in bleeding risk. To fully elucidate the comparative benefits and harms of antithrombotic agents across the various sub-populations, rigorously conducted comparative studies or network meta-regression analyses of patient-level data are required.

Registration: PROSPERO-CRD42012002721~~(PROSPERO registry - CRD42012002721).~~

Formatted: Font: Bold

For peer review only

For peer review only - http://bmjopen.bmj.com/site/about/guidelines.xhtml

Article Summary

Strengths and limitations of this study

- The studies pooled in this meta-analysis include antiplatelet agents in addition to newer oral anticoagulants and standard adjusted dose vitamin K antagonistsThis network meta-analysis includes data on edoxaban from the recently published ENGAGE AF-TIMI 48 trial
- The studies pooled in this meta-analysis include antiplatelet agents in addition to newer oral anticoagulants and standard adjusted dose vitamin K antagonists
- We present findings on both the relative and absolute scale, and graphically illustrate absolute risks using icon arrays
- We report detailed *a priori* sub-group analyses for stroke or systemic embolism and major bleeding using data derived from FDA Public Summary Documents
- We present findings on both the relative and absolute scale, and graphically illustrate absolute risks using icon arrays
- Comparisons between antithrombotic treatments were largely comprised of single studies, albeit large studies
- There is notable heterogeneity and the small number of studies limits the analyses that can be conducted to account for heterogeneity in the absence of patient-level data

For peer review only

INTRODUCTION

Atrial fibrillation (AF) is a common cardiac arrhythmia associated with increased morbidity and mortality.[1,2] Patients with AF sustain an increased risk of arterial thromboembolism and stroke, associated with high recurrence and substantial debilitating impact. Therefore, antithrombotic strategies using anticoagulant drugs and antiplatelet agents are recommended for AF patients presenting with risk factors for stroke. Antithrombotic therapy is also associated with a risk of bleeding; therefore, the beneficial effects on stroke prevention should always be compared against a patient's risk of major bleeding.[1,2]

Existing guidelines recommend anticoagulant therapy for patients at intermediate or high risk of stroke.[1,2] Although standard adjusted dose vitamin K antagonists (VKA) (e.g., warfarin) has been the cornerstone treatment for reducing the risk of stroke or systemic embolism (SE) in this population, it is associated with several drawbacks, such as significant inter- and intra-patient variability in dose response; need for laboratory monitoring; increased risk of bleeding complications; and several food and drug interactions.[2] which These limitations have prompted the development of newer oral anticoagulants such as direct thrombin (dabigatran) and Xa (e.g. apixaban, rivaroxaban, edoxaban) inhibitors. [1,2] While some of these newer treatment options have been demonstrated to be efficacious in preventing stroke or SE compared to standard adjusted dose VKA, the relative efficacy and risk of bleeding of newer oral anticoagulants, both among themselves and in comparison to antiplatelet agents, is not clear, especially in certain sub-populations of patients, e.g., $CHADS_2$ score <2 or ≥2; time in therapeutic range (TTR) < 66% or ≥ 66%; Age <75 or ≥ 75 years. Therefore, the objective of this paper was to compare antithrombotic agents for the prevention of stroke and major bleeding in patients with non-valvular AF and among sub-populations.

For peer review only - http://bmjopen.bmj.com/site/about/guidelines.xhtml

METHODS

Published literature was identified by searching MEDLINE, MEDLINE In-Process & Other Non-Indexed Citations, EMBASE, BIOSIS Previews, PubMed and the Cochrane Central Register of Controlled Trials were searched through the Ovid interface to identify English-language clinical articles published from 1988 to January~~une~~ 23~~7~~, 2014~~2~~. Regular alerts were also established. Websites of regulatory agencies, Canadian and major international health technology assessment agencies, clinical practice guidelines as well as The Cochrane Library (2012, Issue 6) and University of York Centre for Reviews and Dissemination ~~-~~databases were searched. Complete details of the electronic search strategy, including any limits used, are reported in Appendix 1. The protocol was published online and was registered in the PROSPERO International Prospective Register of Systematic Reviews (PROSPERO registry - CRD42012002721).

The population of interest was individuals with non-valvular AF requiring anticoagulation (including all risk levels and regardless of any comorbidities). The following treatments were included in the review: apixaban, dabigatran, edoxaban, rivaroxaban, standard adjusted dose VKA, acetylsalicylic acid (ASA), ASA and clopidogrel. Numerous outcomes were considered and results are reported elsewhere; however, the focus of this publication is the outcomes of all-cause stroke or SE and major bleeding as defined by the International Society on Thrombosis and Haemostasis [ISTH] definition),[3] which were the primary efficacy and safety outcomes used in the newer oral anticoagulant studies.

For peer review only - http://bmjopen.bmj.com/site/about/guidelines.xhtml

Active and placebo-controlled randomized controlled trials (RCTs) were selected for inclusion if they were published in English, included at least one antithrombotic treatment under review (using pre-specified doses), reported any of the pre-specified outcomes related to patient safety or clinical efficacy, and involved patients with non-valvular AF eligible to receive anticoagulant therapy, regardless the level of stroke risk. Trials that included patients with contraindication to anticoagulant treatment were excluded. VKA trials were included if the dose was adjusted to a target international normalised ratio (INR) 2.0-3.0. Any dose of ASA was considered for inclusion, but ASA dose was stratified in the analysis as low (≤ 100 mg daily), medium (>100 mg to ≤ 300 mg daily),, or high (>300 mg daily). Three reviewers independently extracted the data on baseline characteristics, intervention(s) evaluated, including dose, duration, and relevant co-medication and results for each included article, using a standardized template. All extracted data was checked for accuracy by three independent reviewers. Any disagreements in the assessment of these data were resolved through discussion until consensus was reached. Quality assessment of RCTs was also performed independently by two reviewers using a standardized table based on major items from the SIGN 50 instrument for internal validity.[4] The trial selection process is presented in a flowchart based on the Preferred Reporting Items for Systematic Reviews and Meta-Analyses (PRISMA)[5] statement (Appendix 2).

Bayesian network meta-analyses were conducted using WinBUGS software (MRC Biostatistics Unit, Cambridge, UK). A binomial likelihood model[6] which accounts allows for the use of multi-arm trials was used for the analyses given the datasets provided were dichotomous outcomes and included multi-arm trials. Trials Small trials (<100 patients in each arm) with zero

For peer review only

cells in both arms or nodes where there were no events were excluded from the evidence networks because they do not contribute information or allow interpretable information.[6] Both fixed and random-effects network meta-analyses were conducted, although the fixed-effects model was chosen for the reference case analysis, as the evidence network was largely comprised of single study connections. We modeled point estimates and 95% credible intervals for odds ratios (OR) using Markov Chain Monte Carlo methods. The absolute risk difference per 1000 patients treated each year for each outcome was also calculated, based on the standard adjusted dose VKA arm of the Randomized Evaluation of Long-Term Anti-coagulation Therapy (RE-LY) trial.[7] The RE-LY trial[7] was selected because it ~~was the most recent trial which~~ contained data for both $CHADS_2<2$ and $CHADS_2\geq2$ and had detailed data available from the Food and Drug Administration (FDA) Public Summary Report.[8] ~~We also assessed the probability that each treatment was the most efficacious regimen, the second best, the third best, and so on.[10]~~ Vague or flat priors, such as $N(0, 100^2)$ were assigned for basic parameters throughout, although informative priors were considered.[9] Assessment of model fit was based on deviance information criterion (DIC) and comparison of residual deviance to number of unconstrained data points.[6,10] To ensure convergence was reached, trace plots and the Brooks-Gelman-Rubin statistic were assessed.[11] Three chains were fit in WinBUGS for each analysis, with at least 40,000 iterations, and a burn-in of at least 40,000 iterations. We also qualitatively compared the results from our network meta-analysis with direct pair-wise estimates. ~~Frequentist pair-wise meta-analyses were conducted using R (R Language and Environment for Statistical Computing, Vienna, Austria) and package meta.[14]~~

For peer review only

A key assumption behind network meta-analysis is that the analysed network is consistent, i.e., there is no conflict between direct and indirect evidence.[12] To assess inconsistency, we compared deviance and DIC statistics in fitted consistency and inconsistency models.[12] We also plotted the posterior mean deviance of the individual data points in the inconsistency model against their posterior mean deviance in the consistency model to identify any loops in where inconsistency is present (Appendix 11).[12] Additionally, the results from our network meta-analysis were qualitatively compared with direct frequentist pair-wise estimates.

Network meta-analysis also requires that studies are sufficiently similar in order to pool their results.[13] Available study and patient characteristics were assessed to ensure similarity and to investigate the potential impact of heterogeneity on effect estimates. Clinical and methodological heterogeneity was identified in a number of areas. The issues identified were similar to those reported in previous systematic reviews of anticoagulant drugs (e.g., differences in TTR, $CHADS_2$ score).[14] Heterogeneity was assessed by conducting network meta-analysis with the following pre-specified subgroup data reported in the individual RCTs: TTR < 66% or ≥ 66%; $CHADS_2$ score <2 or ≥2; Age <75 or ≥ 75 years.[14] The data to conduct these sub-group analyses were derived from FDA Public Summary Documents when not available in the publications.[8,15,16] Secondary evidence networks were constructed for each of the sub-group analyses. The WinBUGS code[6] and data needed to replicate all analyses are available online.

This research was supported through a grant by the Canadian Institute of Health Research (CIHR) Drug Safety and Effectiveness Network and financial support from the Canadian Agency for Drugs and Technologies in Health (CADTH).

For peer review only
For peer review only - http://bmjopen.bmj.com/site/about/guidelines.xhtml

RESULTS

Study Characteristics of included studies

The systematic review included 13~~2~~ individual RCTs ~~(~~reported in 29~~8~~ publications and FDA reports (Appendix 2)~~)~~; all evaluated the efficacy and safety of antithrombotic agents: ~~(~~dabigatran (150 or 110 mg twice daily), rivaroxaban (20 mg daily), ~~and~~ apixaban (5 mg twice daily)~~)~~, edoxaban (30 or 60 mg daily), standard adjusted dose VKA, ASA (low dose (< 100 mg daily); medium dose (100-300 mg daily), or low dose-ASA plus clopidogrel (75 mg daily) in patients with non-valvular AF. None of the included studies directly compared one new oral anticoagulant drug with another (Appendix 4). Of the 81~~61~~,677~~050~~ randomized patients included in this review, five ~~four~~ large multicentre trials account for 78,296 ~~57,284~~ patients (96~~4~~%): ENGAGE AF-TIMI 48 (n=21,105),[17] ARISTOTLE (n=18,201),[18] RE-LY (n=18,113),[7] ROCKET-AF (n=14,264)[19] and ACTIVE-W (n=6706).[20] Most trials included stroke and SE as a primary efficacy outcome, while bleeding events were a frequent safety outcome. Follow-up ranged from 12 weeks to 3.5 years. All trials published results between 1989 and 2013~~1~~. Mean $CHADS_2$ score was reported in six ~~five~~ trials, encompassing the vast majority of patients included in the systematic review. Reported $CHADS_2$ scores were consistent with a high-risk population ($CHADS_2 \geq 2$), with patients from ROCKET-AF[19] showing the highest risk for stroke (mean $CHADS_2 = 3.5$).

Mean age across the included trials ranged from 65 years to 83 years (Appendix 3). All trials included patients of both gender and had a higher proportion of male participants in most studies. However, the proportions of patients with a prior stroke or TIA varied substantially across the

For peer review only - http://bmjopen.bmj.com/site/about/guidelines.xhtml

included trials, ranging from 3% in JAST to 55% in ROCKET-AF.[19] The same observation can be made regarding the proportions of patients with other concomitant conditions, i.e. congestive heart failure, hypertension, diabetes infarction, and prior vitamin K antagonist experience. TTR was reported in all but one trial that included a standard adjusted dose VKA treatment arm. Patient baseline characteristics are summarized in Appendix 3.

The studies were individually critically appraised and the details are reported elsewhere. Overall, there was substantial variation in study quality. However, large multicentre trials comparing newer anticoagulants with standard adjusted dose VKA such as ENGAGE AF-TIMI 48 (n=21,105), ARISTOTLE (n = 18,201), RE-LY (n = 18,113), and ROCKET-AF (n = 14,264), which account for the vast majority of patients included in the systematic review, appear to be methodologically rigorous.

Stroke or Systemic Embolism

The evidence network (Figure 1) for the primary analysis was comprised of elevennine RCTs representing eight treatments in addition to placebo/observation (N =81,677 60,424). Two Three RCTs (PETRO,[21] ARISTOTLE J,[23] WASPO[22]) were not included in the analysis because they did not report data for this outcome or had with zeroes in both arms. or treatments where there were no events (e.g., Apixaban 2.5 mg).

Apixaban and dabigatran (150 mg twice daily) were associated with reductions in stroke or SE relative to standard adjusted dose VKA (Figure 2). The use of these two agents led to absolute risk reductions ranging from 4 to 6 fewer events per 1,000 patients treated each year. By

For peer review only - http://bmjopen.bmj.com/site/about/guidelines.xhtml

contrast, low-dose ASA and the combination of clopidogrel plus low-dose ASA appeared to have a higher risk of stroke or SE than standard adjusted dose VKA, leading to an increase in the number of stroke or SE ranging from 14 to 15 more events per 1,000 patients treated each year (Figure 4 and Appendix 6). No differences were detected between standard adjusted dose VKA and each of the following interventions: rivaroxaban, edoxaban (30 mg or 60 mg daily), dabigatran (110 mg twice daily), and medium-dose ASA, and no treatment/placebo (Figure 3).

The estimates of effect derived from the direct pair-wise comparisons aligned well with those obtained from the network meta-analysis in both direction and magnitude (Appendix 6). Furthermore, the posterior mean residual deviance (2619.8128) is close to the number of unconstrained data points (2419), which is an indication of reasonable model fit. A number of sub-group analyses and alternative modeling strategies were conducted on the primary analysis, including adjusting for CHADS$_2$, TTR, and age (Table 1). We also conducted a sensitivity analysis where we included the AVERROES[23] and ACTIVE A[24] trial (-Appendix 12). These studies were excluded from the main analysis because we required patients to be eligible for anticoagulant therapy, including treatment with a VKA (Appendix 14). The results differed slightly from the reference case (Appendix 12). The primary analysis was conducted using a fixed-effects model because the network was comprised of single study connections; these results were also compared against those obtained using a random-effects model with vague and informative priors on the standard deviation, and found to be similar in magnitude although wider credible intervals were observed (Appendix 9).

For peer review only

Major bleeding

The evidence network for the primary analysis for major bleeding was comprised of 12~~0~~ RCTs representing eight treatments in addition to placebo/observation (N =81,677~~60,503~~). The evidence network for major bleeding is similar to the evidence network for stroke or SE (Figure 1) but includes one extra RCT (WASPO[22]) comparing medium dose ASA with standard adjusted dose VKA (Appendix 5).

Apixaban~~, and~~ dabigatran 110 mg twice daily, edoxaban 30 mg daily, and ~~-~~edoxaban 60 mg daily were associated with reductions in the risk of major bleeding compared with standard adjusted dose VKA (Figure 2). No differences for major bleeding were detected between standard adjusted dose VKA and each of the remaining interventions: rivaroxaban, dabigatran 150 mg twice daily, clopidogrel plus low-dose ASA, and all ASA dosages (Figure 2). The absolute risk difference of major bleeding relative to standard adjusted dose VKA ranged from 18~~22~~ fewer to 24 more events per 1000 patients treated per year **(**Figure 4 and Appendix 7**)**. A summary of the results for the Bayesian and direct pair-wise meta-analyses is shown in Appendix 7, and Bayesian network meta-analysis results are represented graphically in Figure 2. Complete results from the Bayesian network meta-analysis for all possible comparisons are presented in Figure 3**.**

The estimates of effect derived from the direct pair-wise comparisons aligned well with those obtained from the network meta-analysis in both direction and magnitude (Appendix 7). Furthermore, the posterior mean residual deviance (27~~2~~.1) is close to the number of unconstrained data points (26~~2~~), which is an indication of reasonable model fit. A number of sub-group analyses and alternative modeling strategies were conducted on the primary analysis

For peer review only

For peer review only - http://bmjopen.bmj.com/site/about/guidelines.xhtml

(Table 1). We also conducted a sensitivity analysis where we included the AVERROES[23] and ACTIVE A[24] trial (Appendix 12), as well as analyses using a random-effects model with vague and informative priors (-Appendix 9).

Benefit-Harm Assessment – Stroke or systemic embolism versus major bleeding

Figure 3 summarises the results for all pair-wise comparisons derived from the Bayesian fixed-effects network meta-analysis (Appendix 9). Results relative to standard adjusted dose VKA have been discussed above. For pair-wise comparisons among newer oral anticoagulants for stroke or SE, dabigatran 150 mg twice daily was associated fewer events versus dabigatran 110 mg twice daily, and rivaroxaban, edoxaban 30mg daily, and edoxaban 60 mg daily. Apixaban- , and rivaroxaban were also associated with fewer events compared to edoxaban 30mg daily. No other differences were observed between the new anticoagulant agents. There were no differences among low-dose ASA, medium-dose ASA, and clopidogrel plus low-dose ASA. However, low-dose ASA and the combination of clopidogrel plus low-dose ASA were associated with increases in stroke or systemic embolism compared to all newer oral anticoagulants., including standard adjusted dose VKA, dabigatran (both 150 and 110 mg twice daily), apixaban, and rivaroxaban. For major bleeding, apixaban, and dabigatran 110 mg twice daily -were associated with fewer events versus dabigatran 150 mg twice daily and rivaroxaban. Edoxaban 30 mg daily was associated with fewer events than other new oral anticoagulants, while edoxaban 60 mg daily was associated with fewer events compared with rivaroxaban and clopidogrel plus low-dose ASA. The risk of major bleeding for apixaban was lower compared to clopidogrel plus low-dose ASA. There were no differences associated with the ASA treatments, both for comparisons among themselves and compared to the anticoagulant treatments.

For peer review only

Figure 4 plots the absolute risk~~ difference~~ for stroke or SE and ~~versus~~ major bleeding per 1000 treated for the 10~~8~~ treatments analyzed~~, each relative to standard adjusted dose VKA~~. Data in Figure 4 were obtained from the results presented in Appendix 6 and 7. Examination of the data in Figure 4 suggests that the balance of benefits and harms of the new oral anticoagulants for stroke or systemic embolism and major bleeding is positive compared to standard adjusted dose VKA (decrease in stroke or systemic embolism and/or major bleeding) and largely similar among one another. Comparison of all anticoagulant treatment (including standard adjusted dose VKA) to ASA, or ASA in combination with clopidogrel indicates that whereas the new oral anticoagulants decrease the risk of both stroke or systemic embolism and major bleeding, ASA has a less favorable balance of benefits and harms, i.e., fails to minimize the risk of stroke or systemic embolism and/or major bleeding.

DISCUSSION

We identified 13~~2~~ RCTs comparing antithrombotic agents for the prevention of all cause stroke or systemic embolism and major bleeding in patients with AF. To the best of our knowledge, this is the most up-to-date ~~comprehensive~~ systematic review and network meta-analysis[25–29] to synthesize the available benefits and harms on newer oral anticoagulants, ~~,~~standard adjusted dose VKA, and antiplatelet agents for the prevention of stroke and major bleeding in patients with non-valvular AF (Appendix 13). Harenberg et al[25] recently summarized the published indirect comparisons and network meta-analyses evaluating newer oral anticoagulants for AF and an earlier unpublished version of our analysis[14] was considered among the most comprehensive analyses at that time.[25] This network meta-analysis expands upon that

For peer review only

unpublished report.[14] Overall, our findings align with those reported in previously published network meta-analyses;[25,26,28] however, unlike previous analyses,[25] Wwe include antiplatelet agents in addition to newer oral anticoagulants recently published edoxaban data,and standard adjusted dose VKA;[17] and also include antiplatelet agents. We also present detailed evidence networks[30] to illustrate the body of evidence for each outcome; report analysis using multiple statistical models (Appendix 9); report detailed *a priori* sub-group analyses for each outcome using data derived from FDA Public Summary Documents;[8,15] present findings on both the relative and absolute scale,[31,32] and graphically illustrate results using icon arrays. Our systematic review and network meta-analysis is also the only one published to date that was registered in PROSPERO, and also adheres to PRISMA reporting standards (Appendix 10). It also differs from a recently published traditional meta-analysis by Ruff et al[27] given we report comparison of newer oral anticoagulants versus each other, and also include comparisons with antiplatelet agents.

For stroke or systemic embolism, apixaban and dabigatran 150 mg twice daily were associated with reductions in stroke or systemic embolism relative to standard adjusted dose VKA. By contrast, low-dose ASA and the combination of clopidogrel plus low-dose ASA appeared were associated with increases in stroke or systemic embolism relative to standard adjusted dose VKA. No differences were detected between standard adjusted dose VKA and each of the following interventions: rivaroxaban, dabigatran 110 mg twice daily, edoxaban 30 and 60 mg daily, medium-dose ASA, and no treatment/placebo. The absolute risk reduction of stroke or systemic embolism relative to standard adjusted dose VKA ranged from 6 fewer to 15 more events per 1000 patients treated per year, with dabigatran 150 mg twice daily yielding the largest

For peer review only - http://bmjopen.bmj.com/site/about/guidelines.xhtml

aboslute risk reduction and ~~ASA 100 mg daily yielding~~ clopidogrel plus low-dose ASA the largest absolute risk increase.

For major bleeding, apixaban, ~~and~~ dabigatran 110 mg twice daily, edoxaban 30 mg daily, and edoxaban 60 mg daily were associated with an increased ~~the~~ risk of major bleeding relative to standard adjusted dose VKA. No differences for major bleeding were detected between standard adjusted dose VKA and each of the remaining interventions: rivaroxaban, dabigatran 150 mg twice daily, clopidogrel plus low-dose ASA, and all ASA dosages. The absolute risk difference of major bleeding relative to standard adjusted dose VKA ranged from 18~~22~~ fewer to 24~~3~~ more events per 1000 patients treated, with edoxaban 30 mg daily ~~placebo/no treatment~~ yielding the largest absolute risk reduction and medium dose ASA the largest absolute risk increase. Results of network meta-analyses reflect the relative efficacy and safety profile of antiplatelet and anticoagulant drugs as documented by large clinical trials. Thus, international guidelines[1,2] for prevention of stroke in patients with AF also recommended oral anticoagulation over antiplatelet therapy as the latter had only modest protective effects. With respect to the new oral anticoagulants none of the guidelines has given a recommendation to prefer one of the new oral anticoagulants over another.[1,2]

Network meta-analysis involves pooling of trials. We observed variability across studies included in the evidence network in terms of $CHADS_2$, age and TTR (Appendix 3 and 4). For example, patients using rivaroxaban had a higher risk of stroke because ROCKET-AF[19] included patients with $CHADS_2 \geq 2$. As such, we conducted sub-group analysis to adjust for differences in patient and study-level characteristics. We derived data for sub-group analyses from FDA Public

For peer review only

Summary documents[8,15,16] which enhanced our ability to account for variability across studies (e.g., use intention to treat (ITT) population for rivaroxaban versus per-protocol as-treated population reported in publication[19]). Results from these sub-group analyses differed slightly from those reported in the primary analysis, indicating that there may be differences in the benefits and harms across sub-groups. This becomes more apparent when sub-group analyses are reported on the absolute scale (reported elsewhere). It is unclear whether the absolute risk reductions associated with these differences translate into clinically meaningful benefits in practice, especially in sub-populations of patients with a lower baseline risk of stroke or systemic embolism. To fully elucidate the comparative benefits and harms of antithrombotic agents across the various sub-populations, rigorously conducted comparative studies,[33,34] or network meta-regression analyses of patient-level data[35] are required.

There are several limitations with the analysis we employed. First, there is notable heterogeneity in patients and study characteristics (Appendix 3, 4, and 15). For example, there were differences in patients enrolled in the underlying studies and completion rates. However, the small number of studies limits the analyses that can be conducted to account for heterogeneity in the absence of patient-level data. Second, there is insufficient sub-group data available for other outcomes reported in the RCTs included. As a consequence, detailed analysis was limited to two outcomes – stroke and SE and major bleeding. Third Second, we reported the results from the fixed-effects model in the main text. We felt that this was appropriate as the nodes in evidence networks are connected largely by single studies. Effect estimates derived from the fixed-effects model aligned more closely with the direct estimates. Reporting results from the fixed-effects model in the main text likely biases results in favour of the new oral anticoagulants, as treatments that

For peer review only

For peer review only - http://bmjopen.bmj.com/site/about/guidelines.xhtml

achieved statistically significant results in the primary RCTs retained statistically significant findings when using the fixed-effects model, but not always when using the random-effects model due to the prior[9] on the between study variance. Nevertheless, the results from the random-effects model have also been reported in Appendix 9, along with detailed discussionng around the limitations in using the fixed-effects model. ThirdFourth, the absolute risk reduction for the network meta-analysis was calculated using the event rate in the standard adjusted dose VKA arm of the RE-LY study. The RE-LY trial was selected because it was the most recent trial which contained data for both $CHADS_2 < 2$ and $CHADS_2 \geq 2$ and had detailed data made publicly available following an FDA review of dabigatran. Use of a different study (e.g., ARISTOTLE) for baseline event rate data is unlikely to change the results substantially given that the event rates in standard adjusted dose VKA arms were similar across studies. Fifourth, we present findings using graphics that have been largely applied in the network meta-analysis literature,[36] such as a table with odds ratios for all pair-wise comparisons. This method of presenting findings on the relative scale is problematic when you are conducting assessments to compare the balance of benefits and harms, which need to be standardized. As such, we also provided results in absolute terms. We report our findings using icon arrays, although it should be noted that these results do not account for the utility values based upon patient preferences for each of the outcomes,[37,38] nor do they reflect uncertainty although we report absolute reductions on the risk benefit plane reflecting uncertainty in Appendix 8. There are other methods available that can incorporate patient preferences for outcomes.[37,38] Further, estimates of benefit and harm with several of the therapies (e.g., rivaroxaban) come from one trial and thus such data are not particularly robust. Further research is needed comparing the balance of benefit and harms using other research methodologies, and incorporating other relevant outcomes, and patient preferences

For peer review only

for each of the outcomes,.[37,38] and additional studies when they become available. Finally, haemorrhagic stroke is also considered a major bleed in underlying studies. Accordingly, we may have double counted haemorrhage stroke in this analysis. Unfortunately, we are not able to account for this issue in all included studies. Nonetheless, it is important to note this limitation and highlight that this potentially biases results in favor of newer oral anticoagulants given these agents were associated with reductions in these events.

CONCLUSIONS

Compared with standard adjusted dose VKA, apixaban and dabigatran 150 mg twice daily were associated with reductions in the incidence of stroke or systemic embolism in patients with AF, whereas, low-dose ASA and the combination of clopidogrel plus low-dose ASA increased the risk relative to standard adjusted dose VKA at preventing stroke or systemic embolism. Apixaban, and dDabigatran 110 mg twice daily, edoxaban 30 mg daily, and edoxaban 60 mg daily and was associated with a reduced risk in major bleeding compared with standard adjusted dose VKA. Although the results of the current review revealed that there were differences in stroke or systemic embolism and major bleeding among antithrombotic agents in the management of patients with AF in relative terms, it is unclear whether the absolute risk reductions associated with these differences translate into clinically meaningful benefits in practice, especially in sub-populations of patients with a lower baseline risk of stroke or systemic embolism. Rigorously conducted comparative studies or network meta-regression analyses of patient-level data are required to fully elucidate the comparative benefits and harms of antithrombotic agents across the various sub-populations.

For peer review only - http://bmjopen.bmj.com/site/about/guidelines.xhtml

For peer review only

For peer review only - http://bmjopen.bmj.com/site/about/guidelines.xhtml

TABLES

Table 1: Sub-group and sensitivity analyses for stroke or systemic embolism and major bleeding relative to standard adjusted dose VKA, Odds ratios ± 95% Credible Intervals versus standard adjusted dose VKA

Treatment	Reference Case – Fixed Effect NMA	Subgroup analysis by CHADS $_2$ Score		Subgroup analysis by Age		Subgroup analysis by TTR	
		CHADS $_2$ < 2	CHADS $_2$ ≥ 2	Age < 75 years	Age ≥ 75 years	TTR < 66%	TTR ≥ 66%
**Stroke or Systemic Embolism**							
**Apixaban 5 mg twice daily**	**0.79 (0.66,0.95)**	0.86 (0.57,1.28)	**0.78 (0.63,0.95)**	0.85 (0.67,1.07)	**0.71 (0.53,0.95)**	0.79 (0.62,1.01)	0.79 (0.59,1.05)
**Dabigatran 110 mg twice daily**	0.91 (0.74,1.12)	1 (0.64,1.55)	0.89 (0.71,1.12)	0.93 (0.7,1.23)	0.88 (0.66,1.18)	0.91 (0.69,1.19)	0.9 (0.66,1.24)
**Dabigatran 150 mg twice daily**	**0.66 (0.53,0.82)**	0.61 (0.37,1)	**0.66 (0.52,0.85)**	0.63 (0.46,0.87)	**0.66 (0.48,0.9)**	0.54 (0.39,0.73)	0.81 (0.58,1.11)
**Rivaroxaban 20 mg daily**	0.88 (0.74,1.03)	NA	0.88 (0.74,1.04)	0.95 (0.75,1.2)	0.8 (0.63,1.02)	0.81 (0.65,1.01)	0.72 (0.47,1.1)
~~**No treatment/Placebo**~~	~~1.53 (0.9,2.63)~~	~~1.88 (0.69,5.74)~~	~~NA~~	~~NA~~	~~NA~~	~~NA~~	~~NA~~
**Low Dose ASA (≤ 100 mg daily)**	**1.87 (1.27,2.8)**	2.2 (1.18,4.25)	2.1 (0.91,5.15) *	NA	2.04 (1.32,3.19)	NA	NA
**Medium Dose ASA (> 100 mg and ≤ 300 mg daily)**	1.35 (0.75,2.46)	1.97 (0.63,6.73) **	NA	NA	NA	NA	NA
**Clopidogrel 75 mg daily & Low dose Aspirin (≤ 100 mg daily)**	1.93 (1.42,2.65)	3.16 (1.39,8.22)	1.15 (0.84,1.57)	NA	NA	1.52 (0.96,2.45)	**2.4 (1.59,3.71)**
**Edoxaban 30 mg daily**	1.14(0.99,1.33)	NA	1.14(0.99,1.33)	NA¶	NA¶	NA¶	NA¶
**Edoxaban 60 mg daily**	0.87(0.74,1.02)	NA	0.87(0.74,1.02)	NA¶	NA¶	NA¶	NA¶
**Major Bleeding**							
**Apixaban 5 mg twice daily**	**0.69 (0.6,0.8)**	**0.59 (0.44,0.78)**	**0.73 (0.62,0.87)**	**0.73 (0.6,0.89)**	**0.65 (0.52,0.81)**	**0.57 (0.45,0.71)**	**0.81 (0.67,0.98)**
**Dabigatran 110 mg twice daily**	**0.8 (0.69,0.93)**	**0.65 (0.47,0.89)**	0.86 (0.73,1.02)	**0.61 (0.49,0.77)**	1.02 (0.84,1.25)	0.74 (0.61,0.91)	0.86 (0.69,1.06)
**Dabigatran 150 mg twice daily**	0.93 (0.8,1.08)	0.77 (0.57,1.04)	1.01 (0.86,1.19)	**0.7 (0.56,0.86)**	1.19 (0.98,1.45)	0.76 (0.62,0.94)	1.15 (0.94,1.4)
**Rivaroxaban 20 mg daily**	1.03 (0.89,1.19)	NA	1.03 (0.89,1.19)	0.93 (0.76,1.13)	1.15 (0.93,1.41)	0.92 (0.77,1.1)	**1.3 (1.01,1.69)**
~~**No treatment/Placebo**~~	~~0.33 (0.08,1.08)~~	~~0.35 (0.04,1.75)~~	~~NA~~	~~NA~~	~~NA~~	~~NA~~	~~NA~~

Formatted Table

Formatted: Superscript

For peer review only - http://bmjopen.bmj.com/site/about/guidelines.xhtml

Treatment	Reference Case – Fixed Effect NMA	Subgroup analysis by $CHADS_2$ Score		Subgroup analysis by Age		Subgroup analysis by TTR	
		$CHADS_2$ < 2	$CHADS_2 \geq 2$	Age < 75 years	Age ≥ 75 years	TTR < 66%	TTR ≥ 66%
**Low Dose ASA (≤ 100 mg daily)**	1.05 (0.6,1.86)	0.83 (0.42,1.64)	1.6 (0.55,5.02) *	NA	1.01 (0.57,1.79)	NA	NA
**Medium Dose ASA (> 100 mg and ≤ 300 mg daily)**	1.78 (0.62,5.59)	1.48 (0.11,19.07)**	NA	NA	NA	NA	NA
**Clopidogrel 75 mg daily & Low dose Aspirin (≤ 100 mg daily)**	1.1 (0.83,1.46)	1.51 (0.89,2.61)	0.97 (0.69,1.36) †	NA	NA	0.66 (0.42,1.02)	1.66 (1.12,2.47)
**Edoxaban 30 mg daily**	0.47(0.4,0.54)	NA	0.47(0.4,0.54)	NA¶	NA¶	NA¶	NA¶
**Edoxaban 60 mg daily**	0.78(0.69,0.9)	NA	0.78(0.69,0.9)	NA¶	NA¶	NA¶	NA¶

ASA= acetylsalicylic acid; mg= milligram; NA=not available; NMA= network meta-analysis; OR=odds ratio; TTR= time in therapeutic range; VKA=vitamin K antagonist

* Results may be slightly biased against LDASA. Derived from BAFTA study where $CHADS_2$ subgroup data stratified by $CHADS_2$ 1-2 (vs. 0-1) and $CHADS_2$ 3-6 (vs. 2-6).

** Results may be slightly biased against medium-dose ASA. Based on study level results from CAFA and JAST studies. Although these studies consisted primarily of low-risk populations, some patients may have $CHADS_2$ scores greater than 2.

† Estimated from subgroup study by Healey et al 2008

¶ Raw data (i.e., total number of patients and patients who experienced an event for each sub-group) were not reported in ENGAGE AF-TIMI 48[17]

Formatted Table

Formatted: Superscript

For peer review only

FIGURES

Figure 1: Evidence network for all cause stroke or systemic embolism. The width of the lines is proportional to the number of randomized controlled trials comparing each pair of treatments, and the size of each treatment node is proportional to the number of randomised participants (sample size). A dotted line indicates a three-arm randomized controlled trial and a black node indicates a node included in the analysis but not reported in the main text.

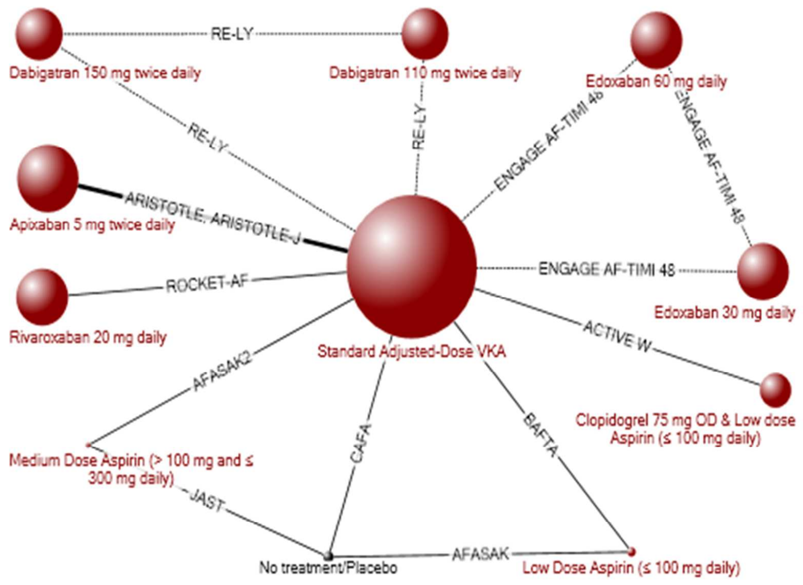

Formatted: Font: (Default) Arial, 11 pt

For peer review only - http://bmjopen.bmj.com/site/about/guidelines.xhtml

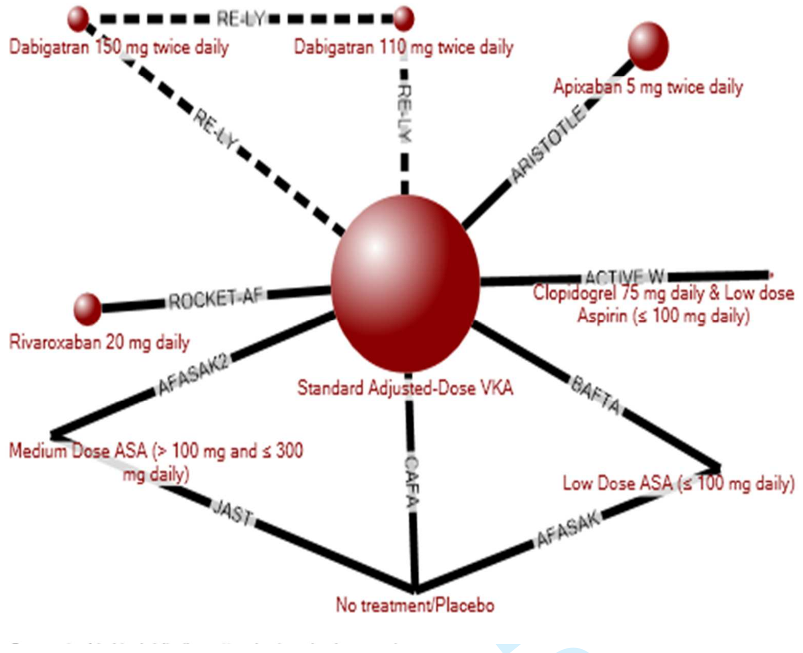

ASA= acetylsalicylic acid; mg= milligram; RCT= RCT = randomized controlled trial; VKA= vitamin K antagonist

Figure 2: Odds ratio for all cause stroke or systemic embolism (A) and major bleeding (B) in Bayesian network meta-analysis versus standard adjusted dose VKA.

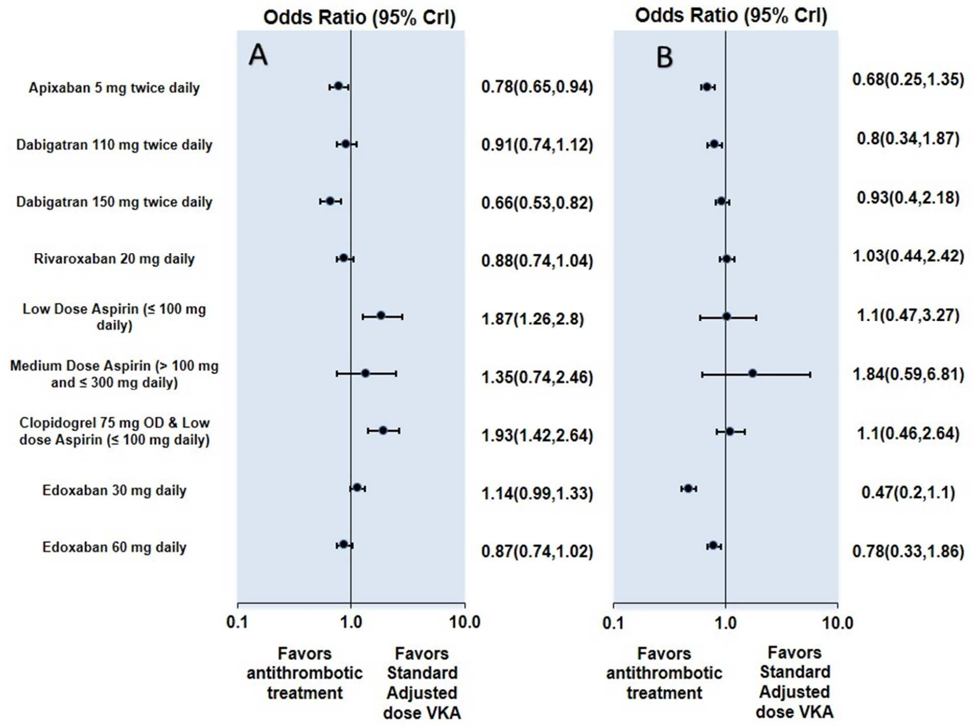

Formatted: Font: (Default) Arial, 11 pt

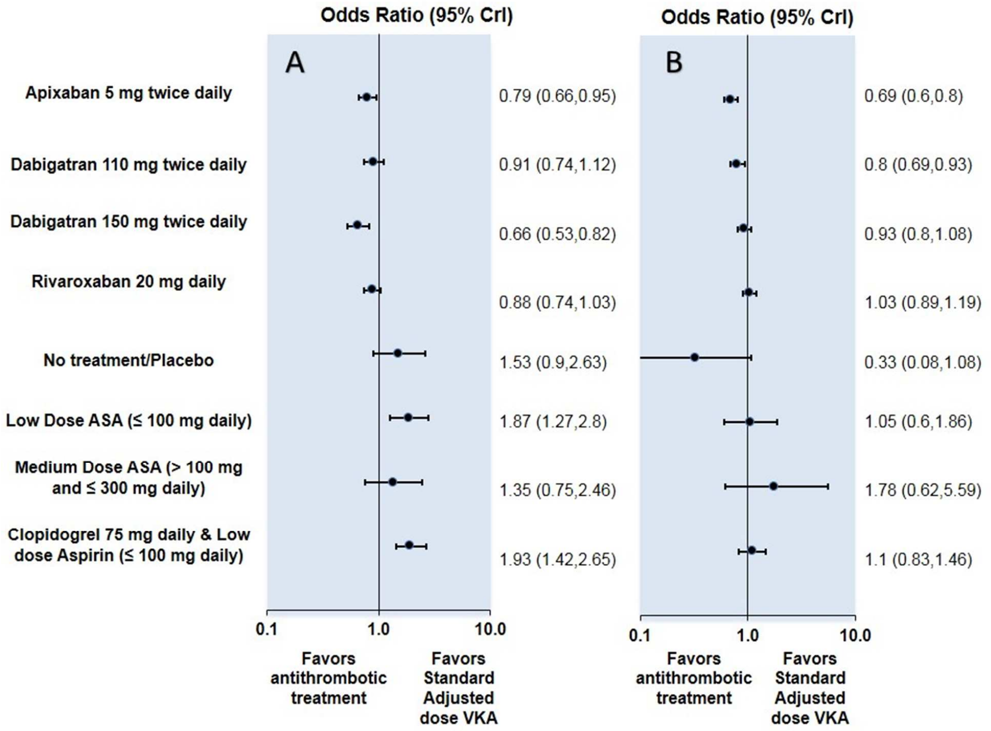

~~ASA= acetylsalicylic acid;~~ CrI= credible interval; mg= milligram; VKA= vitamin K antagonist

Figure 3: Odds Ratios (OR) from network meta-analyses for stroke or systemic embolism and major bleeding for all pair-wise comparisons.* ORs for recurrence of stroke or systemic embolism are below the diagonal (row-defining treatment versus column-defining treatment) and

For peer review only - http://bmjopen.bmj.com/site/about/guidelines.xhtml

those for major bleeding are above the diagonal and in blue (column-defining treatment versus row-defining treatment). To obtain ORs for comparisons in the opposite direction, reciprocals should be taken (e.g., the OR for standard dose warfarin compared with apixaban 5 mg twice daily for stroke or systemic embolism is 1/0.789=1.287). Significant results are in bold and underscored (and in larger font).

Standard Adjusted-Dose VKA	**0.69 (0.6,0.8)**	**0.8 (0.69,0.93)**	0.93 (0.81,1.08)	1.03 (0.89,1.19)	1.05 (0.59,1.86)	1.79 (0.62,5.64)	1.1 (0.83,1.47)	**0.47 (0.4,0.54)**	**0.78 (0.69,0.9)**
**0.78 (0.65,0.94)**	Apixaban 5 mg twice daily	1.16 (0.94,1.43)	**1.35 (1.1,1.65)**	**1.48 (1.21,1.82)**	1.52 (0.84,2.73)	2.59 (0.88,8.2)	**1.59 (1.15,2.19)**	**0.67 (0.54,0.83)**	1.13 (0.93,1.38)
0.91 (0.74,1.12)	1.17 (0.89,1.53)	Dabigatran 110 mg twice daily	1.16 (0.998,1.36)	**1.28 (1.04,1.58)**	1.31 (0.72,2.36)	2.23 (0.76,7.09)	1.38 (0.99,1.9)	**0.58 (0.47,0.72)**	0.98 (0.8,1.2)
**0.66 (0.53,0.82)**	0.84 (0.63,1.12)	**0.72 (0.57,0.9)**	Dabigatran 150 mg twice daily	1.1 (0.9,1.36)	1.13 (0.62,2.03)	1.92 (0.66,6.1)	1.18 (0.86,1.63)	**0.5 (0.4,0.62)**	0.84 (0.69,1.03)
0.88 (0.74,1.04)	1.12 (0.87,1.44)	0.96 (0.74,1.25)	**1.33 (1.01,1.76)**	Rivaroxaban 20 mg daily	1.03 (0.57,1.83)	1.74 (0.6,5.54)	1.07 (0.78,1.48)	**0.45 (0.37,0.56)**	**0.76 (0.63,0.93)**
**1.87 (1.26,2.8)**	**2.39 (1.55,3.72)**	**2.05 (1.31,3.22)**	**2.84 (1.8,4.5)**	**2.14 (1.39,3.3)**	Low Dose Aspirin (≤ 100 mg daily)	1.71 (0.51,6.03)	1.05 (0.55,1.99)	**0.44 (0.25,0.8)**	0.75 (0.42,1.34)
1.35 (0.74,2.46)	1.72 (0.92,3.24)	1.47 (0.78,2.79)	**2.04 (1.08,3.88)**	1.54 (0.83,2.88)	0.72 (0.38,1.35)	Medium Dose Aspirin (> 100 mg and ≤ 300 mg daily)	0.62 (0.19,1.85)	**0.26 (0.08,0.76)**	0.44 (0.14,1.28)
**1.93 (1.42,2.64)**	**2.47 (1.73,3.54)**	**2.11 (1.46,3.07)**	**2.93 (2.01,4.31)**	**2.2 (1.56,3.15)**	1.03 (0.62,1.71)	1.44 (0.73,2.82)	Clopidogrel 75 mg OD & Low dose Aspirin (≤ 100 mg daily)	**0.42 (0.3,0.58)**	**0.71 (0.52,0.98)**
1.14 (0.99,1.33)	**1.46 (1.16,1.85)**	1.25 (0.97,1.62)	**1.74 (1.33,2.28)**	**1.31 (1.04,1.64)**	**0.61 (0.4,0.93)**	0.85 (0.46,1.58)	**0.59 (0.42,0.83)**	Edoxaban 30 mg daily	1.69 (1.44,1.98)
0.87 (0.74,1.02)	1.12 (0.87,1.42)	0.95 (0.73,1.24)	**1.32 (1.01,1.74)**	1 (0.79,1.26)	**0.47 (0.3,0.71)**	0.65 (0.35,1.21)	**0.45 (0.32,0.64)**	**0.76 (0.65,0.89)**	Edoxaban 60 mg daily

mg= milligram; VKA= vitamin K antagonist

* Results between individual treatments, especially newer oral anticoagulants, should be interpreted with caution given the limitations associated with using a fixed-effects model. See Appendix 9 for additional details.

~~Standard adjusted dose VKA~~	~~**0.69 (0.6,0.8)**~~	~~**0.8 (0.69,0.93)**~~	~~0.93 (0.8,1.08)~~	~~1.03 (0.89,1.19)~~	~~0.33 (0.08,1.08)~~	~~1.05 (0.6,1.86)~~	~~1.78 (0.62,5.59)~~	~~1.1 (0.83,1.46)~~
~~**0.79 (0.66,0.95)**~~	~~Apixaban 5 mg twice daily~~	~~1.15 (0.94,1.43)~~	~~**1.34 (1.09,1.65)**~~	~~**1.48 (1.2,1.82)**~~	~~0.48 (0.12,1.57)~~	~~1.52 (0.85,2.73)~~	~~2.57 (0.88,8.16)~~	~~**1.59 (1.15,2.19)**~~

0.91 (0.74,1.12)	1.15 (0.88,1.52)	Dabigatran 110 mg twice daily	1.16 (0.997,1.36)	**1.28 (1.04,1.58)**	0.41 (0.1,1.36)	1.31 (0.74,2.37)	2.23 (0.77,7.06)	1.38 (0.99,1.9)
**0.66 (0.53,0.82)**	0.83 (0.62,1.11)	**0.72 (0.58,0.9)**	Dabigatran 150 mg twice daily	1.1 (0.9,1.35)	0.35 (0.09,1.16)	1.13 (0.63,2.04)	1.91 (0.66,6.06)	1.18 (0.86,1.63)
0.88 (0.74,1.03)	1.11 (0.86,1.42)	0.96 (0.74,1.25)	**1.33 (1.01,1.76)**	Rivaroxaban 20 mg daily	0.32 (0.08,1.06)	1.03 (0.57,1.84)	1.73 (0.6,5.5)	1.07 (0.78,1.48)
1.53 (0.9,2.63)	**1.94 (1.1,3.43)**	1.68 (0.95,3)	**2.33 (1.3,4.18)**	**1.75 (1,3.08)**	No treatment/ Placebo	3.19 (0.89,13.7)	**5.41 (1.6,23.81)**	3.33 (0.99,13.6)
**1.87 (1.27,2.8)**	**2.37 (1.54,3.68)**	**2.05 (1.32,3.23)**	**2.84 (1.82,4.5)**	**2.14 (1.4,3.31)**	1.22 (0.72,2.08)	Low Dose ASA	1.69 (0.52,5.94)	1.05 (0.55,1.97)
1.35 (0.75,2.46)	1.71 (0.92,3.19)	1.48 (0.79,2.8)	**2.05 (1.09,3.9)**	1.55 (0.83,2.87)	0.88 (0.55,1.41)	0.72 (0.39,1.35)	Medium Dose ASA	0.62 (0.19,1.84)
**1.93 (1.42,2.65)**	**2.44 (1.71,3.51)**	**2.12 (1.46,3.08)**	**2.93 (2.01,4.31)**	**2.21 (1.55,3.15)**	1.26 (0.68,2.34)	1.03 (0.62,1.7)	1.43 (0.73,2.79)	Clopidogrel 75 mg daily & Low dose ASA

ASA= acetylsalicylic acid; mg= milligram; VKA= vitamin K antagonist
* Results between individual treatments, especially newer oral anticoagulants, should be interpreted with caution given the limitations associated with using a fixed-effects model. See Appendix 7 for additional details.

For peer review only

For peer review only - http://bmjopen.bmj.com/site/about/guidelines.xhtml

Figure 4: Icon array illustrating the absolute risks of Stroke or systemic embolism (blue) and major bleeding episodes (red) per 1000 patients treated*

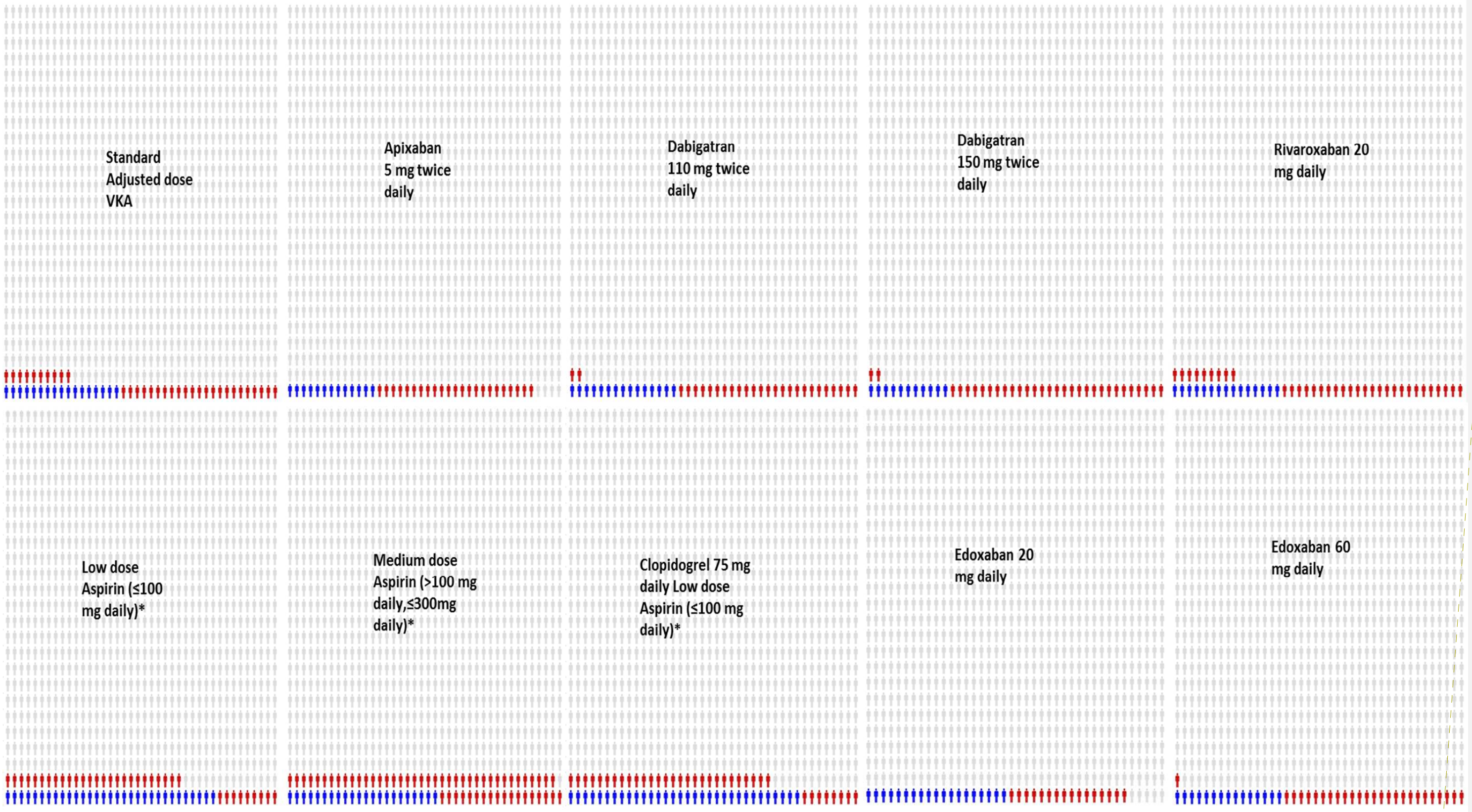

* Figures do not reflect uncertainty around effect estimates and there is more uncertainty (i.e., wider credible intervals) for low dose ASA, medium dose ASA, and clopidogrel plus low dose ASA (See Appendix 8). Underlying studies may also double count haemorrhage stroke.

Formatted: Font: (Default) Arial, 11 pt, Bold

Formatted: Space After: 10 pt, Line spacing: Multiple 1.15 li

For peer review only - http://bmjopen.bmj.com/site/about/guidelines.xhtml

Figure 4: Icon array illustrating the absolute risks of Stroke or systemic embolism (blue) and major bleeding episodes (red) per 1000 patients treated

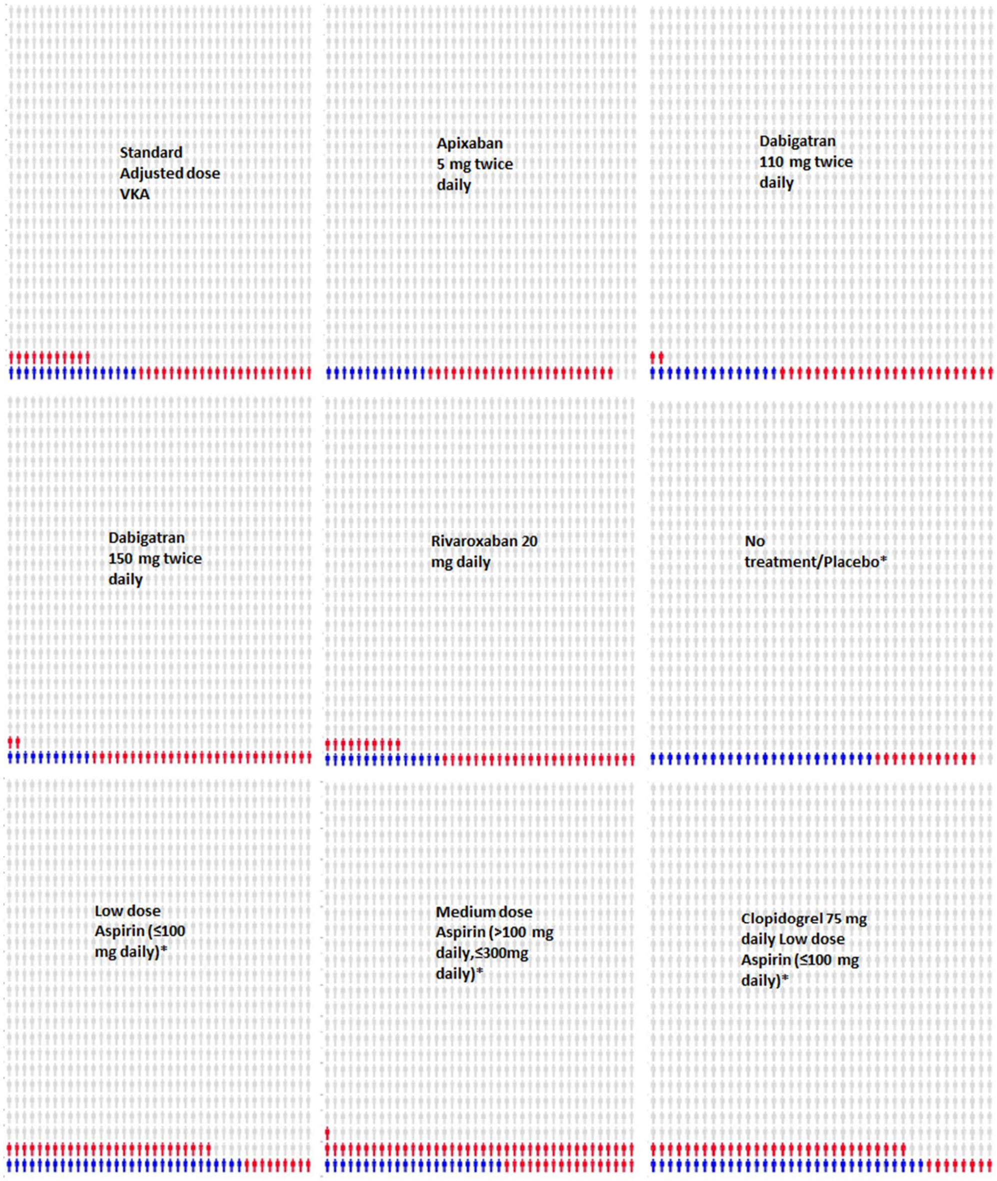

* Figures do not reflect uncertainty around effect estimates and there is more uncertainty (i.e., wider credible intervals) for no treatment/placebo, low dose ASA, medium dose ASA, and clopidogrel plus low dose ASA (See Appendix 6)

Formatted: Space After: 10 pt, Line spacing: Multiple 1.15 li

Acknowledgments: The authors would like to acknowledge Carolyn Spry. She was the Information Specialist from CADTH on the review who conducted the search strategy.

Contributors: CC conceived the study design, performed the data extraction, analysis, and drafted the manuscript. DC and GW conceived the study design, helped with the data analysis, and reviewed the manuscript for important intellectual content. TR conceived the study design, performed the systematic review, data extraction, and reviewed the manuscript for important intellectual content. SK, SS, KG, AB, KM, KC performed the systematic review, data extraction, and reviewed the manuscript for important intellectual content. TC, MC, and KC conceived the study and reviewed the manuscript for important intellectual content. All authors have read and approved the final version of the manuscript. All authors agree to be accountable for all aspects of the work in ensuring that questions related to the accuracy or integrity of any part of the work are appropriately investigated and resolved.

Funding information: This research was supported through a grant by the Canadian Institute of Health Research (CIHR) Drug Safety and Effectiveness Network and financial support from the Canadian Agency for Drugs and Technologies in Health (CADTH). The CIHR Drug Safety and Effectiveness Network played no role in study design, collection, analysis, interpretation of data, writing of the manuscript, or in the decision to submit the paper for publication. They accept no responsibility for the contents. Employees from CADTH were involved in the study design, collection, analysis, interpretation of data, and writing of the manuscript. CADTH, funded by Canadian federal, provincial and territorial governments, is an independent, not-for-profit producer and broker of health technology assessments. Federal, provincial, and territorial health care decision-makers rely on our evidence-based information to make informed decisions about the effectiveness and efficiency of drugs and other health technologies.

Competing interests: All authors have completed the ICMJE uniform disclosure form at www.icmje.org/coi_disclosure.pdf (available on request from the corresponding author) and declare: no support from any organisation for the submitted work; CC is a recipient of a Vanier Canada Graduate Scholarship from the Canadian Institutes of Health Research and has received funding from Canadian Network and Centre for Trials Internationally (CANNeCTIN) and is a trainee on the CIHR Drug Safety and Effectiveness Network team grant. MC is a recipient of a New Investigator Award from the Heart and Stroke Foundation of Canada and holds a T2 research chair in cancer and thrombosis from the University of Ottawa.

Ethical approval: Not required.

Data sharing: The statistical code and dataset used for analyses are reported online: http://www.cadth.ca/media/pdf/TR0003_AntithromboticAgents-AF_ScienceReport-e.pdf. Additional data can be requested by emailing George Wells: gawells@ottawaheart.ca .

The Corresponding Author has the right to grant on behalf of all authors and does grant on behalf of all authors, an exclusive licence (or non exclusive for government employees) on a worldwide basis to the BMJ Publishing Group Ltd to permit this article (if accepted) to be published in BMJ editions and any other BMJPGL products and sublicences such use and exploit all subsidiary rights, as set out in our licence

For peer review only - http://bmjopen.bmj.com/site/about/guidelines.xhtml

REFERENCES

1. Kirchhof, P. *et al.* Atrial fibrillation guidelines across the Atlantic: a comparison of the current recommendations of the European Society of Cardiology/European Heart Rhythm Association/European Association of Cardiothoracic Surgeons, the American College of Cardiology Fou. *Eur. Heart J.* **34,** 1471–1474 (2013).

2. Skanes, A. C. *et al.* Focused 2012 update of the Canadian Cardiovascular Society atrial fibrillation guidelines: recommendations for stroke prevention and rate/rhythm control. *Can. J. Cardiol.* **28,** 125–36 (2012).

3. Schulman, S. & Kearon, C. Definition of major bleeding in clinical investigations of antihemostatic medicinal products in non-surgical patients. *J. Thromb. Haemost.* **3,** 692–4 (2005).

4. NHS Quality Improvement Scotland (NHS QIS). *SIGN 50. A guideline developer's handbook*. (2008). at <http://www.sign.ac.uk/pdf/sign50.pdf>

5. Moher, D., Liberati, A., Tetzlaff, J. & Altman, D. G. Preferred reporting items for systematic reviews and meta-analyses: the PRISMA statement. *PLoS Med.* **6,** e1000097 (2009).

6. Dias, S., Welton, N. J., Sutton, A. J. & Ades, A. *NICE DSU Technical Support Document 2: A Generalised Linear Modelling Framework for Pairwise and Network Meta-Analysis of Randomised Controlled Trials*. *Trop. Med.* 1–96 (2011). at <www.nicedsu.org.uk>

7. Connolly, S. & Ezekowitz, M. Dabigatran versus warfarin in patients with atrial fibrillation. *N. Engl. J. Med.* **361,** 1139–1151 (2009).

8. Ingelheim, B. *Advisory committee briefing document: dabigatran etexilate [Internet]*. 1–168 (2010). at <http://www.fda.gov/downloads/advisorycommittees/committeesmeetingmaterials/drugs/cardiovas>

9. Turner, R. M., Davey, J., Clarke, M. J., Thompson, S. G. & Higgins, J. P. Predicting the extent of heterogeneity in meta-analysis, using empirical data from the Cochrane Database of Systematic Reviews. *Int. J. Epidemiol.* **41,** 818–27 (2012).

10. Spiegelhalter, D. J., Best, N. G., Carlin, B. P. & van der Linde, A. Bayesian measures of model complexity and fit. *J. R. Stat. Soc. Ser. B (Statistical Methodol.* **64,** 583–639 (2002).

11. Spiegelhalter, D., Thomas, A. & Best, N. WinBUGS user manual. *MRC Biostat. Unit* **2,** (2004).

For peer review only

12. Dias, S., Welton, N.J., Sutton, A.J., Caldwell, D.M., Guobing, L. & Ades, A. E. *NICE Technical Support Document 4: Inconsistency in Networks of Evidence Based on Randomised Controlled Trials*. 1–39 (2011). at <www.nicedsu.org.uk>

13. Salanti, G., Marinho, V. & Higgins, J. P. T. A case study of multiple-treatments meta-analysis demonstrates that covariates should be considered. *J. Clin. Epidemiol.* **62,** 857–64 (2009).

14. Wells, G., Coyle, D., Cameron, C., Steiner, S. & Kelly, S. *Safety, effectiveness, and costeffectiveness of new oral anticoagulants compared with warfarin in preventing stroke and other cardiovascular events in patients with atrial fibrillation*. (2012). at <http://www.cadth.ca/media/pdf/NOAC_Therapeutic_Review_final_report.pdf>

15. Johnson & Johnson Pharmaceutical Research & Development. *Rivaroxaban for the Prevention of Stroke and Non-Central Nervous System (CNS) Systemic Embolism in Patients With Atrial Fibrillation*. **7939,** 222 (2011).

16. McDonagh, M. S., Peterson, K., Balshem, H. & Helfand, M. US Food and Drug Administration documents can provide unpublished evidence relevant to systematic reviews. *J. Clin. Epidemiol.* (2013). doi:10.1016/j.jclinepi.2013.05.006

17. Giugliano, R. P. *et al.* Edoxaban versus warfarin in patients with atrial fibrillation. *N. Engl. J. Med.* **369,** 2093–104 (2013).

18. Granger, C. & Alexander, J. Apixaban versus warfarin in patients with atrial fibrillation. *N. Engl. J. Med.* 981–992 (2011).

19. Patel, M., Mahaffey, K. & Garg, J. Rivaroxaban versus warfarin in nonvalvular atrial fibrillation. *N. Engl. J. Med.* 883–891 (2011).

20. Connolly, S. *et al.* Clopidogrel plus aspirin versus oral anticoagulation for atrial fibrillation in the Atrial fibrillation Clopidogrel Trial with Irbesartan for prevention of Vascular Events (ACTIVE W): a randomised controlled trial. *Lancet* **367,** 1903–12 (2006).

21. Ezekowitz, M. D. *et al.* Dabigatran with or without concomitant aspirin compared with warfarin alone in patients with nonvalvular atrial fibrillation (PETRO Study). *Am. J. Cardiol.* **100,** 1419–26 (2007).

22. Rash, A. *et al.* A randomised controlled trial of warfarin versus aspirin for stroke prevention in octogenarians with atrial fibrillation (WASPO). *Age Ageing* **36,** 151–6 (2007).

23. Connolly, S. & Eikelboom, J. Apixaban in patients with atrial fibrillation. *N. Engl. J. Med.* **364,** 806–817 (2011).

For peer review only

24. Connolly, S. J. *et al.* Effect of clopidogrel added to aspirin in patients with atrial fibrillation. *N. Engl. J. Med.* **360,** 2066–78 (2009).

25. Harenberg, J. & Weiss, C. Clinical trials with new oral anticoagulants. Additive value of indirect comparisons also named network meta-analyses. *Hamostaseologie* **33,** 62–70 (2013).

26. Dogliotti, A., Paolasso, E. & Giugliano, R. P. Current and new oral antithrombotics in non-valvular atrial fibrillation: a network meta-analysis of 79 808 patients. *Heart* (2013). doi:10.1136/heartjnl-2013-304347

27. Ruff, C. T. *et al.* Comparison of the efficacy and safety of new oral anticoagulants with warfarin in patients with atrial fi brillation□: a meta-analysis of randomised trials. *Lancet* **6736,** 1–8 (2013).

28. Assiri, A., Al-Majzoub, O., Kanaan, A. O., Donovan, J. L. & Silva, M. Mixed treatment comparison meta-analysis of aspirin, warfarin, and new anticoagulants for stroke prevention in patients with nonvalvular atrial fibrillation. *Clin. Ther.* **35,** 967–984.e2 (2013).

29. Kmh, B. S. & Berge, E. Factor Xa inhibitors versus vitamin K antagonists for preventing cerebral or systemic embolism in patients with atrial fibrillation (Review). (2013).

30. Salanti, Georgia, Kavvoura, F, Ioannidis, J. Exploring the geometry of treatment networks. *Ann. Intern. Med.* **148,** 544–553 (2008).

31. Barratt, A. *et al.* Tips for learners of evidence-based medicine: 1. Relative risk reduction, absolute risk reduction and number needed to treat. *Can. Med. Assoc. J.* **171,** 353–8 (2004).

32. Schechtman, E. Odds ratio, relative risk, absolute risk reduction, and the number needed to treat--which of these should we use? *Value Health* **5,** 431–6 (2002).

33. Berger, M. L., Mamdani, M., Atkins, D. & Johnson, M. L. Good research practices for comparative effectiveness research: defining, reporting and interpreting nonrandomized studies of treatment effects using secondary data sources: the ISPOR Good Research Practices for Retrospective Database Analysis Task Force . *Value Health* **12,** 1044–52 (2009).

34. Berger, M. L. *et al.* Prospective observational studies to assess comparative effectiveness: the ISPOR good research practices task force report. *Value Health* **15,** 217–30 (2012).

35. Jansen, J. P. Network meta-analysis of individual and aggregate level data. *Res. Synth. Methods* **3,** 177–190 (2012).

For peer review only - http://bmjopen.bmj.com/site/about/guidelines.xhtml

36. Salanti, G., Ades, A. & Ioannidis, J. Graphical methods and numerical summaries for presenting results from multiple-treatment meta-analysis: an overview and tutorial. *J. Clin. Epidemiol.* **64,** 163–71 (2011).

37. Guo, J. J. *et al.* A review of quantitative risk-benefit methodologies for assessing drug safety and efficacy-report of the ISPOR risk-benefit management working group. *Value Health* **13,** 657–66 (2010).

38. Boyd, C. *et al. Methods Research Report Methods for Benefit and Harm Assessment in Systematic Reviews*. 52 (2012). at <www.ncbi.nlm.nih.gov/books/NBK115750/>

39. Thorlund, K., Thabane, L. & Mills, E. J. Modelling heterogeneity variances in multiple treatment comparison meta-analysis – Are informative priors the better solution□? *BMC Med. Res. Methodol.* **13,** 1 (2013).

For peer review only

For peer review only - http://bmjopen.bmj.com/site/about/guidelines.xhtml

SUPPLEMENTARY APPENDIX

Appendix 1: Search Strategy

**OVERVIEW**	
Interface:	Ovid
Databases:	EBM Reviews - Cochrane Central Register of Controlled Trials <May 2012><br>Ovid Embase 1974 to June 6, 2012<br>Ovid MEDLINE 1946 to June 7, 2012<br>Ovid MEDLINE Daily<br>Ovid MEDLINE In-Process & Other Non-Indexed Citations<br>**Note:** Subject headings have been customized for each database. Duplicates between databases were removed in Ovid.
Date of Search:	June 7, 2012; updated January 23, 2014
Alerts:	Monthly search updates began June 11, 2012 and ran until project completion
Study Types:	Randomized Controlled Trials
Limits:	Publication years 1988-present<br>Humans<br>Conference abstracts omitted

**SYNTAX GUIDE**	
/	At the end of a phrase, searches the phrase as a subject heading
.sh	At the end of a phrase, searches the phrase as a subject heading
MeSH	Medical Subject Heading
exp	Explode a subject heading
*	Before a word, indicates that the marked subject heading is a primary topic;<br>or, after a word, a truncation symbol (wildcard) to retrieve plurals or varying endings
#	Truncation symbol for one character
ADJ	Requires words are adjacent to each other (in any order)
ADJ#	Adjacency within # number of words (in any order)
.ti	Title
.ab	Abstract
.hw	Heading Word; usually includes subject headings and controlled vocabulary
.nm.	Name of substance word
.ot	Original title
.pt	Publication type
.rn	CAS registry number
use cctr	Select results from Cochrane database of Clinical Trials
use oemezd	Select results from Embase database

For peer review only

For peer review only - http://bmjopen.bmj.com/site/about/guidelines.xhtml

use pmez	Select results from Medline database

Clinical Multi-database Strategy

Line #	Search Strategy
1	(211914-51-1 or 211915-06-9).rn,nm. or (dabigatran* or Pradaxa or Pradax or Prazaxa or Praxada or bibr 1048 or bibr1048 or bibr953 or bibr 953 or rendix).ti,ot,ab,sh,rn,hw,nm.
2	(BMS 562247 or BMS562247 or apixaban* or Eliquis or Eliques).ti,ot,ab,sh,rn,hw,nm. or 503612-47-3.rn,nm.
3	(rivaroxaban* or Xarelto or BAY 59-7939 or BAY59-7939 or BAY597939 or BAY 597939).ti,ot,ab,sh,rn,hw,nm. or 366789-02-8.rn,nm.
4	(90055-48-4 or 113665-84-2).rn,nm. or (clopidogrel* or clopilet or grepid or iscover or plavix or zopya or zylagren or zyllt or duocover or duoplavin).ti,ot,ab,sh,rn,hw,nm.
5	Aspirin/ or 50-78-2.rn,nm. or (acetylsalicylic acid* or ASA* or acenterine or acesal or acetan or acetard or aceticil or aceticyl or acetilum or acetonyl or acetilum or acetonyl or acetophen or acetosal or acetosalicylic acid or acetosalin or acetosalum or acetyl salicylate or acetyl salicylic acid or acetylin or acetylo or acetylon or acetylosalicylic acid or acetylsal or acetysal or acetylsalicyclic acid or acetylsalicyl or acetylsalicylate or acetylsalycic acid or acetylsalycylic acid or oracidulatum or actorin or Acylpyrin or acylpyrine or acytosal or adiro or alabunkun or alasil or albyl-e or alka seltzer or alkASA or Aloxiprimum or anacin or anasprin or anopyrin or ansin or anthrom or aptor or arthralgyl or asaflow or ascription or orasaphen or asapor or asatard or asawin or aspec or aspent or aspergum or aspex or aspilets or aspirem or aspirgran or ASAa or ASAe or ASAine or aspirisucre or aspisol or aspo cid or aspro or asrina or asrivo or asta or asteric or astrix or bebesan or biprin or bokey or boxazin or breoprin or bufferin or cafenol or caprin or cASA or catalgine or catalgix or cemerit or cemirit or claradin or claragine or Colfarit or comoprin or contrheuma or daga or darosal or Dispril or dispirin or dolean or dusil or Easprin or ecasil or Ecotrin or egalgic or emocin or empirin or encaprin or encine em or Endosprin or entaprin or entercin or enteroprin or enterosarine or enterospirine or entrophen or eskotrin or euthermine or extren or genasprin or globentyl or godamed or gotosan or helicon or idotyl or infatabs a or istopirin or istopyrine or ivepirine or juvepirine or keypo or kilios or kinderASA or Magnecyl or measurin or mejoral or melabon or Micristin or micropyrin or mikristin or miniasal or mycrisitin or naspro or novasen or nu seal or nu seals or ortho acetoxybenzoate or ortho acetoxybenzoic acid or ortho acetyloxybenzoate or ortho acetyloxybenzoic acid or ostoprin or pancemol or paracin or paynocil or pengo or plewin or Polopirin or polopiryna or premaspin or primaspan or proprin or pyronoval or reumyl or rhodine or rhonal or ronal or salacetin or salisalido or sargepirine or soldral or solpyron or Solprin or solucetyl or solupsa or Solupsan or spren or super tru or tapal or temagin or tevapirin or th 2152 or thrombo-aspilets or treupahlin or treuphalin or tromalyt or tromcor or turiital or verin or vitalink or xaxa or Zorprin).ti,ot,ab,sh,rn,hw,nm.
6	Warfarin/ or 81-81-2.rn,nm. or (adoisine or aldocumar or athrombin* or befarin or brumolin or carfin or circuvit or compound 42 or coumadan or coumadin or coumadine or coumafen or coumafene or coumefene or coumaphene or coumarins or dagonal or farin or jantoven or kumatox or maforan or marevan or orfarin or panwarfarin or panwarfin or prothromadin or simarc 2 or sofarin or tedicumar or tintorane or waran or warfant or warfar or warfarin or warfarine or warfil or warfilone or warnerin).ti,ot,ab,sh,rn,hw,nm.
7	Acenocoumarol/ or 152-72-7.rn,nm. or (acenocoumarol* or nicoumalone or acenocoumarin or acenocoumarine or acenocoumarole or acenocoumarolum or acenocumarol or acenocumarolo or acenocumerol or acenkumarin or acitrom or ascumar or neo sintrom or neosintrom or neositron or nicoumalone or nicumalon or nitrovarfarian or nitrowarfarin or sincoumar or sincumar or sinkumar or sinthrom or sinthrome or sintrom or sintroma or sintron or syncoumar or syncumar or synthrom or syntrom or syntroma or sintron or syncoumar or syncumar or syntrom or trombostop or zotil or sinthrome or synthrom or syncoumar or syncumar or sinkumar or sintrom or minisintrom or trombostop or zotil).ti,ot,ab,sh,rn,hw,nm.
8	or/1-7
9	Atrial fibrillation/
10	((atrial or atrium or auricular or heart) adj1 fibrillation*).ti,ab.
11	(a-fib or afib).ti,ab.
12	9 or 10 or 11
13	8 and 12
14	13 use pmez
15	13 use cctr
16	14 or 15

For peer review only - http://bmjopen.bmj.com/site/about/guidelines.xhtml

17	*dabigatran/ or (dabigatran* or Pradaxa or Pradax or Prazaxa or praxada or bibr 1048 or bibr1048 or bibr953 or bibr 953 or rendix).ti,ab.
18	*apixaban/ or (BMS 562247 or BMS562247 or apixaban* or Eliquis or Eliques).ti,ab.
19	*rivaroxaban/ or (rivaroxaban* or Xarelto or BAY 59-7939 or BAY59-7939 or BAY597939 or BAY 597939).ti,ab.
20	*clopidogrel/ or *acetylsalicylic acid plus clopidogrel/ or (clopidogrel* or clopilet or grepid or iscover or plavix or zopya or zylagren or zyllt or duocover or duoplavin).ti,ab.
21	*warfarin/ or (adoisine or aldocumar or athrombin* or befarin or brumolin or carfin or circuvit or compound 42 or coumadan or coumadin or coumadine or coumafen or coumafene or coumefene or coumaphene or coumarins or dagonal or farin or jantoven or kumatox or maforan or marevan or orfarin or panwarfarin or panwarfin or prothromadin or simarc 2 or sofarin or tedicumar or tintorane or waran or warfant or warfar or warfarin* or warfarine or warfil or warfilone or warnerin).ti,ab.
22	*acetylsalicylic acid/ or (acetylsalicylic acid* or ASA* or acenterine or acesal or acetan or acetard or aceticil or aceticyl or acetilum or acetonyl or acetilum or acetonyl or acetophen or acetosal or acetosalicylic acid or acetosalin or acetosalum or acetyl salicylate or acetyl salicylic acid or acetylin or acetylo or acetylon or acetylosalicylic acid or acetylsal or acetysal or acetylsalicyclic acid or acetylsalicyl or acetylsalicylate or acetylsalycic acid or acetylsalycylic acid or oracidulatum or actorin or Acylpyrin or acylpyrine or acytosal or adiro or alabunkun or alasil or albyl-e or alka seltzer or alkASA or Aloxiprimum or anasprin or anopyrin or ansin or anthrom or aptor or arthralgyl or asaflow or orasaphen or asapor or asatard or asawin or aspec or aspent or aspergum or aspex or aspilets or aspirem or aspirgran or ASAa or ASAe or ASAine or aspirisucre or aspisol or aspo cid or aspro or asrina or asrivo or asta or asteric or astrix or bebesan or biprin or bokey or boxazin or breoprin or bufferin or cafenol or caprin or cASA or catalgine or catalgix or cemerit or cemirit or claradin or claragine or Colfarit or comoprin or contrheuma or daga or darosal or Dispril or dispirin or dolean or dusil or Easprin or ecasil or Ecotrin or egalgic or emocin or empirin or encaprin or encine em or Endosprin or entaprin or entercin or enteroprin or enterosarine or enterospirine or entrophen or eskotrin or euthermine or extren or genasprin or globentyl or godamed or gotosan or helicon or idotyl or infatabs a or istopirin or istopyrine or ivepirine or juvepirine or keypo or kilios or kinderASA or Magnecyl or measurin or mejoral or melabon or Micristin or micropyrin or mikristin or miniasal or mycrisitin or naspro or novasen or nu seal or nu seals or ortho acetoxybenzoate or ortho acetoxybenzoic acid or ortho acetyloxybenzoate or ortho acetyloxybenzoic acid or ostoprin or pancemol or paracin or paynocil or pengo or plewin or Polopirin or polopiryna or premaspin or primaspan or proprin or pyronoval or reumyl or rhodine or rhonal or ronal or salacetin or salisalido or sargepirine or soldral or solpyron or Solprin or solucetyl or solupsa or Solupsan or spren or super tru or tapal or temagin or tevapirin or th 2152 or thrombo-aspilets or treupahlin or treuphalin or tromalyt or tromcor or turiital or verin or vitalink or xaxa or Zorprin).ti,ab.
23	*Acenocoumarol/ or (acenocoumarol* or nicoumalone or acenocoumarin or acenocoumarine or acenocoumarole or acenocoumarolum or acenocumarol or acenocumarolo or acenocumerol or acenkumarin or acitrom or ascumar or neo sintrom or neosintrom or neositron or nicoumalone or nicumalon or nitrovarfarian or nitrowarfarin or sincoumar or sincumar or sinkumar or sinthrom or sinthrome or sintrom or sintroma or sintron or syncoumar or syncumar or synthrom or syntrom or syntroma or sintron or syncoumar or syncumar or syntrom or trombostop or zotil or sinthrome or synthrom or syncoumar or syncumar or sinkumar or sintrom or minisintrom or trombostop or zotil).ti,ab.
24	or/17-23
25	Heart atrium fibrillation/
26	((atrial or atrium or auricular or heart) adj1 fibrillation*).ti,ab.
27	(a-fib or afib).ti,ab.
28	25 or 26 or 27
29	24 and 28
30	29 use oemezd
31	30 not conference abstract.pt.
32	16 or 31
33	32
34	limit 33 to english language [Limit not valid in CCTR; records were retained]
35	(Randomized Controlled Trial or Controlled Clinical Trial).pt.
36	Randomized Controlled Trial/
37	Randomized Controlled Trials as Topic/

For peer review only

38	"Randomized Controlled Trial (topic)"/
39	Controlled Clinical Trial/
40	Controlled Clinical Trials as Topic/
41	"Controlled Clinical Trial (topic)"/
42	Randomization/
43	Random Allocation/
44	Double-Blind Method/
45	Double Blind Procedure/
46	Double-Blind Studies/
47	Single-Blind Method/
48	Single Blind Procedure/
49	Single-Blind Studies/
50	Placebos/
51	Placebo/
52	Control Groups/
53	Control Group/
54	(random* or sham or placebo*).ti,ab,hw.
55	((singl* or doubl*) adj (blind* or dumm* or mask*)).ti,ab,hw.
56	((tripl* or trebl*) adj (blind* or dumm* or mask*)).ti,ab,hw.
57	(control* adj3 (study or studies or trial*)).ti,ab.
58	(Nonrandom* or non random* or non-random* or quasi-random* or quasirandom*).ti,ab,hw.
59	allocated.ti,ab,hw.
60	((open label or open-label) adj5 (study or studies or trial*)).ti,ab,hw.
61	or/35-60
62	34 and 61
63	limit 62 to yr = "1988 -Current"
64	remove duplicates from 63

**OTHER DATABASES**	
PubMed	Same MeSH, keywords, limits, and study types used as per MEDLINE search, with appropriate syntax used.

Grey Literature

**Dates for Search:**	**June 12, 2012 – July 8, 2012; updated January 2014**
**Keywords:**	dabigatran, Pradax, Pradaxa, apixaban, Eliquis, rivaroxaban, Xarelto, clopidogrel, Plavix, acetylsalicylic acid, Aspirin, warfarin, Coumadin, acenocoumarol, Sintrom, Sinthrome<br>Atrial fibrillation
**Limits:**	Publication years 1988-present, English

Relevant items from the following sections of the CADTH grey literature checklist, “Grey matters: a practical tool for evidence-based searching” (http://www.cadth.ca/resources/grey-matters) were searched:

- Health Technology Assessment Agencies
- Health Economic
- Clinical Practice Guidelines
- Databases (free)

Internet Search

For peer review only - http://bmjopen.bmj.com/site/about/guidelines.xhtml

Formatted Table

OVERVIEW	
Interface:	Ovid
Databases:	EBM Reviews - Cochrane Central Register of Controlled Trials <May 2012><br>Ovid Embase 1974 to June 6, 2012<br>Ovid MEDLINE 1946 to June 7, 2012<br>Ovid MEDLINE Daily<br>Ovid MEDLINE In-Process & Other Non-Indexed Citations<br>**Note:** Subject headings have been customized for each database. Duplicates between databases were removed in Ovid.
Date of Search:	June 7, 2012
Alerts:	Monthly search updates began June 11, 2012 and ran until project completion
Study Types:	Randomized Controlled Trials
Limits:	Publication years 1988-present<br>Humans<br>Conference abstracts omitted

SYNTAX GUIDE	
/	At the end of a phrase, searches the phrase as a subject heading
.sh	At the end of a phrase, searches the phrase as a subject heading
MeSH	Medical Subject Heading
exp	Explode a subject heading
*	Before a word, indicates that the marked subject heading is a primary topic;<br>or, after a word, a truncation symbol (wildcard) to retrieve plurals or varying endings
#	Truncation symbol for one character
ADJ	Requires words are adjacent to each other (in any order)
ADJ#	Adjacency within # number of words (in any order)
.ti	Title
.ab	Abstract
.hw	Heading Word; usually includes subject headings and controlled vocabulary
.nm.	Name of substance word
.ot	Original title
.pt	Publication type
.rn	CAS registry number
use cctr	Select results from Cochrane database of Clinical Trials
use oemezd	Select results from Embase database
use pmez	Select results from Medline database

Clinical Multi-database Strategy

Line #	Search Strategy
1	(211914-51-1 or 211915-06-9).rn,nm. or (dabigatran* or Pradaxa or Pradax or Prazaxa or Praxada or bibr 1048 or bibr1048 or bibr953 or bibr 953 or rendix).ti,ot,ab,sh,rn,hw,nm.
2	(BMS 562247 or BMS562247 or apixaban* or Eliquis or Eliques).ti,ot,ab,sh,rn,hw,nm. or 503612-47-3.rn,nm.
3	(rivaroxaban* or Xarelto or BAY 59-7939 or BAY59-7939 or BAY597939 or BAY 597939).ti,ot,ab,sh,rn,hw,nm. or 366789-02-8.rn,nm.
4	(90055-48-4 or 113665-84-2).rn,nm. or (clopidogrel* or clopilet or grepid or iscover or plavix or zopya or zylagren or zyllt or duocover or duoplavin).ti,ot,ab,sh,rn,hw,nm.
5	Aspirin/ or 50-78-2.rn,nm. or (acetylsalicylic acid* or ASA* or acenterine or acesal or acetan or

For peer review only - http://bmjopen.bmj.com/site/about/guidelines.xhtml

	acetard or aceticil or aceticyl or acetilum or acetonyl or acetilum or acetonyl or acetophen or acetosal or acetosalicylic acid or acetosalin or acetosalum or acetyl salicylate or acetyl salicylic acid or acetylin or acetylo or acetylon or acetylosalicylic acid or acetylsal or acetysal or acetylsalicyclic acid or acetylsalicyl or acetylsalicylate or acetylsalycic acid or acetylsalycylic acid or oracidulatum or actorin or Acylpyrin or acylpyrine or acytosal or adiro or alabunkun or alasil or albyl-e or alka seltzer or alkASA or Aloxiprimum or anacin or anasprin or anopyrin or ansin or anthrom or aptor or arthralgyl or asaflow or ascription or orasaphen or asapor or asatard or asawin or aspec or aspent or aspergum or aspex or aspilets or aspirem or aspirgran or ASAa or ASAe or ASAine or aspirisucre or aspisol or aspo cid or aspro or asrina or asrivo or asta or asteric or astrix or bebesan or biprin or bokey or boxazin or breoprin or bufferin or cafenol or caprin or cASA or catalgine or catalgix or cemerit or cemirit or claradin or claragine or Colfarit or comoprin or contrheuma or daga or darosal or Dispril or dispirin or dolean or dusil or Easprin or ecasil or Ecotrin or egalgic or emocin or empirin or encaprin or encine em or Endosprin or entaprin or entercin or enteroprin or enterosarine or enterospirine or entrophen or eskotrin or euthermine or extren or genasprin or globentyl or godamed or gotosan or helicon or idotyl or infatabs a or istopirin or istopyrine or ivepirine or juvepirine or keypo or kilios or kinderASA or Magnecyl or measurin or mejoral or melabon or Micristin or micropyrin or mikristin or miniasal or mycrisitin or naspro or novasen or nu seal or nu seals or ortho acetoxybenzoate or ortho acetoxybenzoic acid or ortho acetyloxybenzoate or ortho acetyloxybenzoic acid or ostoprin or pancemol or paracin or paynocil or pengo or plewin or Polopirin or polopiryna or premaspin or primaspan or proprin or pyronoval or reumyl or rhodine or rhonal or ronal or salacetin or salisalido or sargepirine or soldral or solpyron or Solprin or solucetyl or solupsa or Solupsan or spren or super tru or tapal or temagin or tevapirin or th 2152 or thrombo-aspilets or treupahlin or treuphalin or tromalyt or tromcor or turiital or verin or vitalink or xaxa or Zorprin).ti,ot,ab,sh,rn,hw,nm.
6	Warfarin/ or 81-81-2.rn,nm. or (adoisine or aldocumar or athrombin* or befarin or brumolin or carfin or circuvit or compound 42 or coumadan or coumadin or coumadine or coumafen or coumafene or coumefene or coumaphene or coumarins or dagonal or farin or jantoven or kumatox or maforan or marevan or orfarin or panwarfarin or panwarfin or prothromadin or simarc 2 or sofarin or tedicumar or tintorane or waran or warfant or warfar or warfarin or warfarine or warfil or warfilone or warnerin).ti,ot,ab,sh,rn,hw,nm.
7	Acenocoumarol/ or 152-72-7.rn,nm. or (acenocoumarol* or nicoumalone or acenocoumarin or acenocoumarine or acenocoumarole or acenocoumarolum or acenocumarol or acenocumarolo or acenocumerol or acenkumarin or acitrom or ascumar or neo sintrom or neosintrom or neositron or nicoumalone or nicumalon or nitrovarfarian or nitrowarfarin or sincoumar or sincumar or sinkumar or sinthrom or sinthrome or sintrom or sintroma or sintron or syncoumar or syncumar or synthrom or syntrom or syntroma or sintron or syncoumar or syncumar or syntrom or trombostop or zotil or sinthrome or synthrom or syncoumar or syncumar or sinkumar or sintrom or minisintrom or trombostop or zotil).ti,ot,ab,sh,rn,hw,nm.
8	or/1-7
9	Atrial fibrillation/
10	((atrial or atrium or auricular or heart) adj1 fibrillation*).ti,ab.
11	(a-fib or afib).ti,ab.
12	9 or 10 or 11
13	8 and 12
14	13 use pmez
15	13 use cctr
16	14 or 15
17	*dabigatran/ or (dabigatran* or Pradaxa or Pradax or Prazaxa or praxada or bibr 1048 or bibr1048 or bibr953 or bibr 953 or rendix).ti,ab.
18	*apixaban/ or (BMS 562247 or BMS562247 or apixaban* or Eliquis or Eliques).ti,ab.
19	*rivaroxaban/ or (rivaroxaban* or Xarelto or BAY 59-7939 or BAY59-7939 or BAY597939 or BAY 597939).ti,ab.
20	*clopidogrel/ or *acetylsalicylic acid plus clopidogrel/ or (clopidogrel* or clopilet or grepid or iscover or plavix or zopya or zylagren or zyllt or duocover or duoplavin).ti,ab.
21	*warfarin/ or (adoisine or aldocumar or athrombin* or befarin or brumolin or carfin or circuvit or compound 42 or coumadan or coumadin or coumadine or coumafen or coumafene or coumefene or coumaphene or coumarins or dagonal or farin or jantoven or kumatox or maforan or marevan or orfarin or panwarfarin or panwarfin or prothromadin or simarc 2 or sofarin or tedicumar or tintorane or waran or warfant or warfar or warfarin* or warfarine or warfil or warfilone or warnerin).ti,ab.
22	*acetylsalicylic acid/ or (acetylsalicylic acid* or ASA* or acenterine or acesal or acetan or acetard or

For peer review only - http://bmjopen.bmj.com/site/about/guidelines.xhtml

	aceticil or aceticyl or acetilum or acetonyl or acetilum or acetonyl or acetophen or acetosal or acetosalicylic acid or acetosalin or acetosalum or acetyl salicylate or acetyl salicylic acid or acetylin or acetylo or acetylon or acetylosalicylic acid or acetylsal or acetysal or acetylsalicyclic acid or acetylsalicyl or acetylsalicylate or acetylsalycic acid or acetylsalycylic acid or oracidulatum or actorin or Acylpyrin or acylpyrine or acytosal or adiro or alabunkun or alasil or albyl-e or alka seltzer or alkASA or Aloxiprimum or anasprin or anopyrin or ansin or anthrom or aptor or arthralgyl or asaflow or orasaphen or asapor or asatard or asawin or aspec or aspent or aspergum or aspex or aspilets or aspirem or aspirgran or ASAa or ASAe or ASAine or aspirisucre or aspisol or aspo cid or aspro or asrina or asrivo or asta or asteric or astrix or bebesan or biprin or bokey or boxazin or breoprin or bufferin or cafenol or caprin or cASA or catalgine or catalgix or cemerit or cemirit or claradin or claragine or Colfarit or comoprin or contrheuma or daga or darosal or Dispril or dispirin or dolean or dusil or Easprin or ecasil or Ecotrin or egalgic or emocin or empirin or encaprin or encine em or Endosprin or entaprin or entercin or enteroprin or enterosarine or enterospirine or entrophen or eskotrin or euthermine or extren or genasprin or globentyl or godamed or gotosan or helicon or idotyl or infatabs a or istopirin or istopyrine or ivepirine or juvepirine or keypo or kilios or kinderASA or Magnecyl or measurin or mejoral or melabon or Micristin or micropyrin or mikristin or miniasal or mycrisitin or naspro or novasen or nu seal or nu seals or ortho acetoxybenzoate or ortho acetoxybenzoic acid or ortho acetyloxybenzoate or ortho acetyloxybenzoic acid or ostoprin or pancemol or paracin or paynocil or pengo or plewin or Polopirin or polopiryna or premaspin or primaspan or proprin or pyronoval or reumyl or rhodine or rhonal or ronal or salacetin or salisalido or sargepirine or soldral or solpyron or Solprin or solucetyl or solupsa or Solupsan or spren or super tru or tapal or temagin or tevapirin or th 2152 or thrombo-aspilets or treupahlin or treuphalin or tromalyt or tromcor or turiital or verin or vitalink or xaxa or Zorprin).ti,ab.
23	*Acenocoumarol/ or (acenocoumarol* or nicoumalone or acenocoumarin or acenocoumarine or acenocoumarole or acenocoumarolum or acenocumarol or acenocumarolo or acenocumerol or acenkumarin or acitrom or ascumar or neo sintrom or neosintrom or neositron or nicoumalone or nicumalon or nitrovarfarian or nitrowarfarin or sincoumar or sincumar or sinkumar or sinthrom or sinthrome or sintrom or sintroma or sintron or syncoumar or syncumar or synthrom or syntrom or syntroma or sintron or syncoumar or syncumar or syntrom or trombostop or zotil or sinthrome or synthrom or syncoumar or syncumar or sinkumar or sintrom or minisintrom or trombostop or zotil).ti,ab.
24	or/17-23
25	Heart atrium fibrillation/
26	((atrial or atrium or auricular or heart) adj1 fibrillation*).ti,ab.
27	(a-fib or afib).ti,ab.
28	25 or 26 or 27
29	24 and 28
30	29 use oemezd
31	30 not conference abstract.pt.
32	16 or 31
33	32
34	limit 33 to english language [Limit not valid in CCTR; records were retained]
35	(Randomized Controlled Trial or Controlled Clinical Trial).pt.
36	Randomized Controlled Trial/
37	Randomized Controlled Trials as Topic/
38	"Randomized Controlled Trial (topic)"/
39	Controlled Clinical Trial/
40	Controlled Clinical Trials as Topic/
41	"Controlled Clinical Trial (topic)"/
42	Randomization/
43	Random Allocation/
44	Double Blind Method/
45	Double Blind Procedure/
46	Double-Blind Studies/
47	Single-Blind Method/
48	Single Blind Procedure/
49	Single-Blind Studies/

For peer review only - http://bmjopen.bmj.com/site/about/guidelines.xhtml

50	Placebos/
51	Placebo/
52	Control Groups/
53	Control Group/
54	(random* or sham or placebo*).ti,ab,hw.
55	((singl* or doubl*) adj (blind* or dumm* or mask*)).ti,ab,hw.
56	((tripl* or trebl*) adj (blind* or dumm* or mask*)).ti,ab,hw.
57	(control* adj3 (study or studies or trial*)).ti,ab.
58	(Nonrandom* or non random* or non-random* or quasi-random* or quasirandom*).ti,ab,hw.
59	allocated.ti,ab,hw.
60	((open label or open-label) adj5 (study or studies or trial*)).ti,ab,hw.
61	or/35-60
62	34 and 61
63	limit 62 to yr = "1988 -Current"
64	remove duplicates from 63

OTHER DATABASES	
PubMed	Same MeSH, keywords, limits, and study types used as per MEDLINE search, with appropriate syntax used.

Grey Literature

**Dates for Search:**	**June 12, 2012 – July 8, 2012**
**Keywords:**	dabigatran, Pradax, Pradaxa, apixaban, Eliquis, rivaroxaban, Xarelto, clopidogrel, Plavix, acetylsalicylic acid, Aspirin, warfarin, Coumadin, acenocoumarol, Sintrom, Sinthrome<br>Atrial fibrillation
**Limits:**	Publication years 1988-present, English

Relevant items from the following sections of the CADTH grey literature checklist, "Grey matters: a practical tool for evidence-based searching" (http://www.cadth.ca/resources/grey-matters) were searched:

- Health Technology Assessment Agencies
- Health Economic
- Clinical Practice Guidelines
- Databases (free)

Internet Search

For peer review only - http://bmjopen.bmj.com/site/about/guidelines.xhtml

Appendix 2: PRISMA Diagram

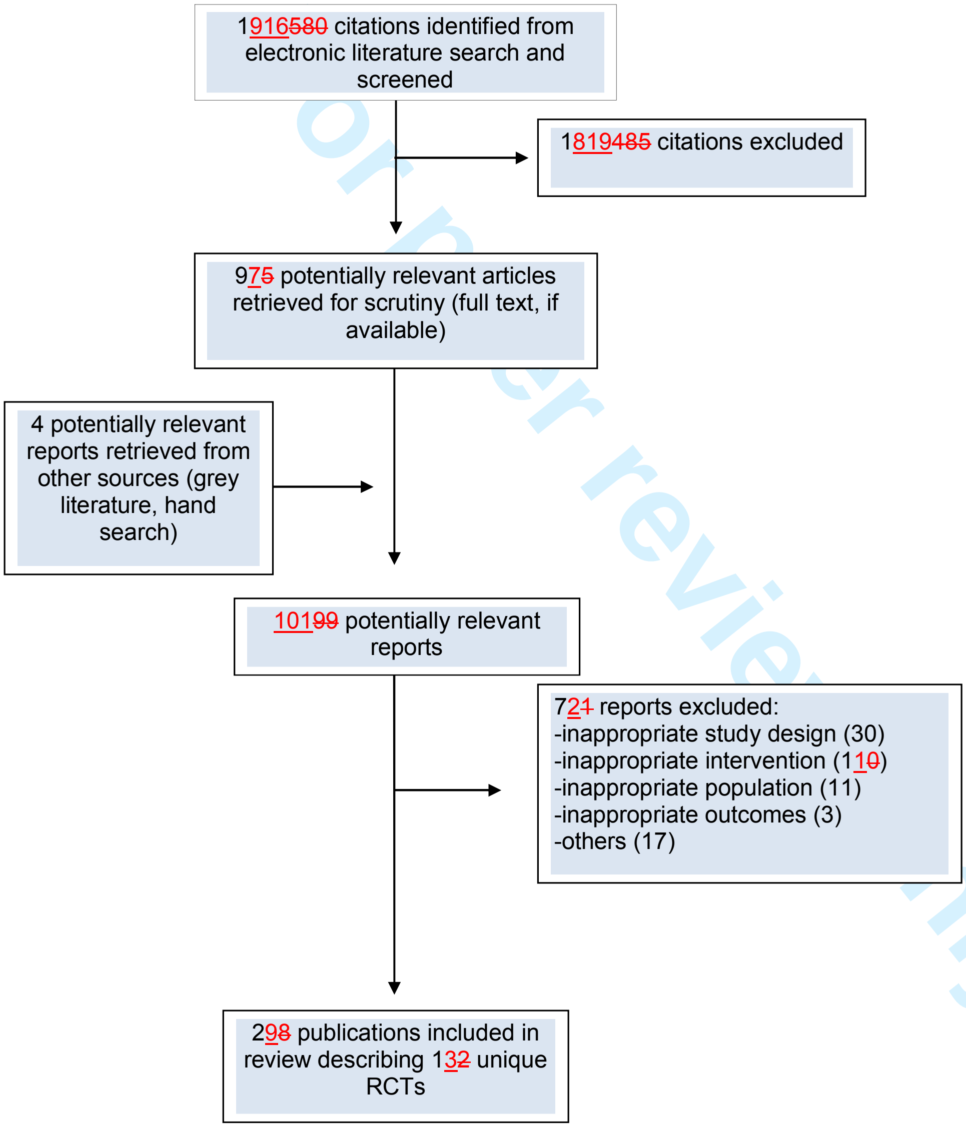

For peer review only - http://bmjopen.bmj.com/site/about/guidelines.xhtml

Appendix 3: Summary of Patient Baseline Characteristics

Study	Baseline Characteristics															Centre above TTR Throughout follow-up		
	Age, Mean ± SD (y)	Age Category (%)		Sex (%)		$CHADS_2$, Mean ± SD	$CHADS_2$ Category (%)			Prior Stroke /TIA (%)	DM (%)	Heart Failure (%)	HBP (%)	MI (%)	VKA Naïve (%)	Mean TTR (%)	TTR Category (%)	
		≤75y	>75 y	M	F		0	1	≥2								≤66%	>66%
ACTIVE-W n=6706	70 ± NR	NR	NR	66	34	2 ± 1.1	NR	NR	NR	15	21	30	82	17	23	64	48	52
AFASAK n=672	75 ± NR	NR	NR	54	46	NR ± NR	NR	NR	NR	6	9	53	32	7	NR	N/A	N/A	N/A
AFASAK-2 n=339	73 ± NR	NR	NR	61	39	NR ± NR	NR	NR	NR	8	12	70	45	7	NR	NR	NR	NR
ARISTOTLE n=18201	70 ± NR	69	31	65	35	2.1 ± 1.1	0	34	66	19	25	35	87	14	43	62	NR	NR
ARISTOTLE-J n=222	70 ± NR	69	31	83	17	1.9 ± NR	1	42	57	28	23	1	83	NR	16	60	NR	NR
BAFTA n=973	82 ± 4	0	100	55	45	NR ± NR	NR	NR	NR	13	13	20	54	11	NR	67	NR	NR
CAFA n=378	68 ± NR	NR	NR	75	25	NR ± NR	NR	NR	NR	4	12	22	39	13	NR	44	NR	NR
JAST n=871	65 ± NR	NR	NR	70	30	NR ± NR	NR	NR	NR	3	14	9	39	NR	92	N/A	N/A	N/A
PETRO n=236	70 ± NR	70	30	82	18	NR ± NR	NR	NR	NR	18	25	32	71	NR	0	57	NR	NR
RE-LY n=18113	71 ± NR	60	40	64	36	2.1 ± NR	32		68	20	20	23	32	79	17	64	50	50
ROCKET-AF n=14264	73 ± NR	NR	NR	60	40	3.5 ± NR	0	0	100	55	40	62	91	17	38	55	NR	NR
WASPO n=75	83 ± NR	0	100	47	53	NR ± NR	NR	NR	NR	NR	4	NR	47	NR	NR	69	NR	NR
ENGAGE AF-TIMI 48 n=21105	72 ± NR	60	40	62	38	2.8 ± 1.0	0	0	100	28	36	57	94	12	41	65	52	48

DM = diabetes mellitus; F = female sex; HBP = high blood pressure; M = male sex; MI = myocardial infarction; N/A = not applicable; NR = not reported; SD = standard deviation; TIA = transient ischemic attack; TTR = time in therapeutic range; VKA = vitamin K antagonist; y = year.

Note: Only data from the included relevant treatment arms have been extracted.

For peer review only - http://bmjopen.bmj.com/site/about/guidelines.xhtml

Appendix 4: Summary of Included Trials

Study and Design	Disposition	Population	Interventions	Follow-up	Primary Outcome(s)
**ACTIVE-W**[13-16] OL RCT, 2006. Multicenter, 32 countries (including Europe / America).	Randomized: n=6706 Completed: n=6026 (90%)	AF patients eligible / willing to take anticoagulant therapy, with ≥ 1 pre-specified risk factor for stroke.	**Clopidogrel / Low-dose ASA** (n=3335)<br>**Warfarin – INR 2.0 to 3.0** (n=3371)	1.3 years (stopped early)	First occurrence of stroke, non-CNS SE, MI or vascular death.
**AFASAK**[17] OL/DB RCT, 1989. Multicenter, Copenhagen.	Randomized: n=1007 Completed: n=785 (80%)	AF patients, with no recent anticoagulation or cerebrovascular event, valve replacement or rheumatic heart disease.	***OL Warfarin – INR 2.8 to 4.2 **** *(n=335)*<br>**Low-dose ASA** (n=336)<br>**PL** (n=336)	2 years	Thromboembolic complication: stroke, TIA or embolic complication to viscera / extremities.
**AFASAK-2**[18,19] OL RCT, 1998. Multicenter, Copenhagen.	Randomized: n=677 Completed: n=507 (75%)	AF patients ≥ 60 years (unless with CV or pulmonary disease), without recent TIA or risk factors for bleeding.	***Fixed-dose Warfarin **** *(n=167)*<br>***Fixed-dose Warfarin / Medium-dose ASA **** *(n=171)*<br>**Medium Dose ASA** (n=169)<br>**Warfarin – INR 2.0 to 3.0** (n=170)	3.5 years	Stroke or SE.
**ARISTOTLE**[20-23] DB RCT, 2011. Multicenter, 39 countries (including Europe / America).	Randomized: n=18201 Completed: n=13468 (74%)	AF patients with ≥ 1 risk factor for stroke, without recent stroke or ASA > 165 mg.	**Apixaban 5 mg BID** (n=9120)<br>**Warfarin – INR 2.0 to 3.0** (n=9081)<br>Concomitant use of **ASA** (≤ 165 mg daily) or **NSAIDs** permitted.	NR	Stroke or SE; Major bleeding.
**ARISTOTLE-J**[24] OL/DB RCT, 2011. Multicenter, Japan.	Randomized: n=222 Completed: n=197 (89%)	AF patients with ≥ 1 risk factor for stroke, without recent TIA or ASA > 100 mg.	**Apixaban 2.5 mg BID** (n=74)<br>**Apixaban 5 mg BID** (n=74)<br>**OL Warfarin – INR 2.0 to 3.0** (n=74)<br>Concomitant use of **ASA** (≤ 100 mg daily) permitted.	12 weeks	Composite of major bleeding and CRNM bleeding events.
**BAFTA**[25] OL RCT, 2007. Multicenter, UK.	Randomized: n=973 Completed: n=694 (72%)	AF patients ≥ 75 years, with clinical uncertainty around optimal treatment.	**Low-dose ASA** (n=485)<br>**Warfarin – INR 2.0 to 3.0** (n=488)	2.7 years	Disabling stroke, ICH and other clinically significant arterial embolism.
**CAFA**[26] DB RCT, 1991. Multicenter, Canada.	Randomized: n=378 Completed: n=286 (76%)	AF patients without recent stroke, TIA or MI, and who did not require antiplatelet drug therapy.	**Warfarin – INR 2.0 to 3.0** (n=187)<br>**PL** (n=191)	1.2 years (stopped early)	Ischemic stroke, SE, ICH or fatal hemorrhage.

For peer review only - http://bmjopen.bmj.com/site/about/guidelines.xhtml

Study and Design	Disposition	Population	Interventions	Follow-up	Primary Outcome(s)
**JAST**[27]<br>OL RCT, 2006.<br>Multicenter, Japan.	Randomized: n=871<br>Completed: n=686<br>(79%)	AF patients without recent symptomatic thromboembolic disease, ICH or GI bleeding.	**Medium-dose ASA** (n=426)<br>**No treatment** (n=445)	2.1 years<br>(stopped early)	CV death, symptomatic brain infarction, or TIA.
**PETRO**[28]<br>OL/DB RCT, 2007.<br>Multicenter, 4 countries (Europe and USA).	Randomized: n=502<br>Completed: n=464<br>(92%)	AF patients with ≥ 1 risk factor for stroke and with coronary artery disease (removed halfway to facilitate recruitment). No recent MI, stroke, TIA or major bleed. Warfarin pre-treatment required for ≥ 8 weeks.	***Dabigatran 50 mg BID ± Low-dose or High-dose ASA*** ** (n=105)*<br>***Dabigatran 300 mg BID ± Low-dose or High-dose ASA*** ** (n=161)*<br>**Dabigatran 150 mg BID ± Low-dose or High-dose ASA** † (n=166)<br>**OL Warfarin – INR 2.0 to 3.0** (n=70)	12 weeks	Thromboembolic events and bleeding events.
**RE-LY**[29-36]<br>OL/DB RCT, 2009.<br>Multicenter, 44 countries (including Europe / America).	Randomized: n=18113<br>Completed: n=14580<br>(80%)	AF patients with ≥ 1 risk factor for stroke, without recent stroke or increased risk of bleeding.	**Dabigatran 110 mg BID** (n=6015)<br>**Dabigatran 150 mg BID** (n=6076)<br>**OL Warfarin – INR 2.0 to 3.0** (n=6022)<br>Concomitant use of **ASA** (<100 mg daily) or other **antiplatelet agents** permitted.	2 years	Stroke or SE and major haemorrhage.
**ROCKET-AF**[37-39]<br>DB RCT, 2011.<br>Multicenter, 45 countries (including Europe / America).	Randomized: n=14264<br>Completed: n=10957<br>(77%)	AF patients at moderate to high risk of stroke (CHADS2 score ≥ 2).	**Rivaroxaban 20 mg OD** (n=7131)<br>**Warfarin – INR 2.0 to 3.0** (n=7133)<br>Over a third of patients in both arms took concomitant ASA.	1.9 years	Composite of stroke and SE;<br>Composite of major and NMCR bleeding.
**WASPO**[40]<br>OL RCT, 2007.<br>Multicenter, UK.	Randomized: n=75<br>Completed: n=64<br>(85%)	Ambulant AF patients > 80 and < 90 years with Folstein score > 25, without previous fall, ICH or GI bleeding. Patients with prior stroke or TIA were excluded.	**Warfarin – INR 2.0 to 3.0** (n=36)<br>**Medium-dose ASA** (n=39)	1 year	Combined endpoints: death; stroke, TIA or SE; serious bleeding; withdrawal.
**ENGAGE AF-TIMI 48**<br>DB RCT, 2013.<br>Multicenter, 46 countries (including Europe / America).	Randomized: n=21105<br>Completed: n=18635<br>(88%)	AF patients at moderate to high risk of stroke (CHADS2 score ≥ 2).	**Edoxaban 30 mg OD** (n=7034)<br>**Edoxaban 60 mg OD** (n=7035)<br>**Warfarin – INR 2.0 to 3.0** (n=7036)<br>Almost a third of patients in treatment arms took concomitant ASA.	2.8 years	Stroke or SE and major haemorrhage.

AF = atrial fibrillation; **ASA** = acetylsalicylic acid; **CNS** = central nervous system; **CRNM** = clinically relevant non-major bleeding; **CV** = cardiovascular; **DB** = double-blind; **GI** = gastrointestinal; **ICH** = intracerebral hemorrhage; **MI** = myocardial infarction; **NR** = not reported; **NSAIDs** = non-steroidal anti-inflammatory drugs; **OL** = open label; **PL** = placebo; **RCT** = randomized controlled trial; **SE** = systemic embolism; **TIA** = transient ischemic attack; **UK** = United-Kingdom; **VKA** = vitamin K antagonist.

* No data was extracted from these treatment arms since they did not meet the inclusion criteria specified in the protocol. These trials were nevertheless included in the systematic review because they had at least one relevant comparison between two interventions of interest.

Comment [CC1]: Insert Reference

For peer review only - http://bmjopen.bmj.com/site/about/guidelines.xhtml

Study and Design	Disposition	Population	Interventions	Follow-up	Primary Outcome(s)

† Data was extracted only for dabigatran without ASA (n=100).

For peer review only

For peer review only - http://bmjopen.bmj.com/site/about/guidelines.xhtml

Appendix 5: Evidence network for major bleeding. The width of the lines is proportional to the number of randomized controlled trials (RCTs) comparing each pair of treatments, and the size of each treatment node is proportional to the number of randomised participants (sample size). A dotted line indicates a three-arm RCT and a black node indicates a node included in the analysis but not reported in the main text.

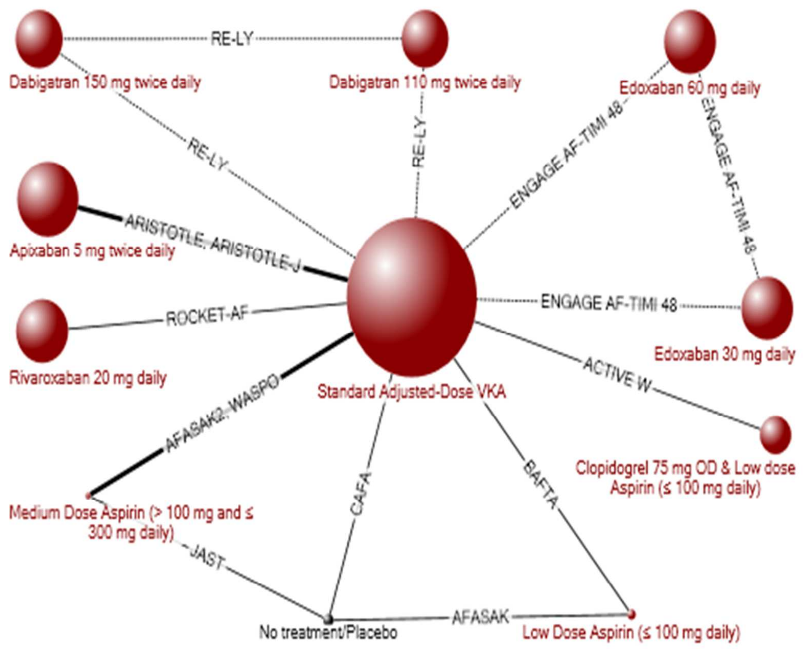

Formatted: Font: (Default) Arial, 11 pt

For peer review only - http://bmjopen.bmj.com/site/about/guidelines.xhtml

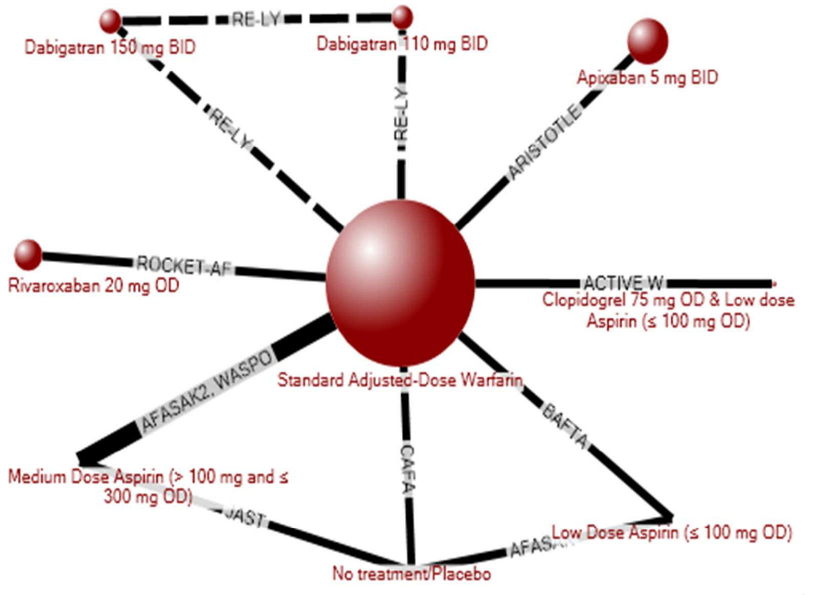

mg= milligram; RCT = randomized controlled trial

Appendix 6: Summary of results from Network Meta-analysis for All-cause Stroke or Systemic Embolism

Treatment	Pair-wise Meta-Analysis, OR (95% CI) versus standard adjusted	NMA, Odds Ratio (95% CrI) versus standard	NMA, Absolute Risk Difference per 1,000 treated (95% CrI) versus standard adjusted dose	Probability Best (%) for Outcome	Rank for Outcom	SUCRA

Formatted Table

For peer review only - http://bmjopen.bmj.com/site/about/guidelines.xhtml

	dose VKA	adjusted dose VKA	VKA		e	
Standard Adjusted-Dose VKAStandard Adjusted- dose VKA	NANA	NANA	NANA	0.0%0.00 %	6.05.00	50.4%
Apixaban 5 mg twice dailyApixaban 5 mg twice daily	0.79(0.66,0.94)0.79 (0.66,0.94)	0.78(0.65,0.94)0.79 (0.66,0.95)	4 fewer (6 fewer-1 fewer)4 fewer (1 fewer-6 fewer)	11.1%9.90 %	2.42.30	85.5%
Dabigatran 110 mg twice dailyDabigatran 110 mg twice daily	0.91(0.75,1.11)0.91 (0.75,1.11)	0.91(0.74,1.12)0.91 (0.74,1.12)	2 fewer (5 fewer-2 more)2 fewer (2 more-5 fewer)	0.1%0.13 %	4.53.80	64.9%
Dabigatran 150 mg twice dailyDabigatran 150 mg twice daily	0.66(0.53,0.82)0.66 (0.53,0.82)	0.66(0.53,0.82)0.66 (0.53,0.82)	6 fewer (8 fewer-3 fewer)6 fewer (3 fewer-8 fewer)	85.3%87.50%	1.21.10	98.1%
Rivaroxaban 20 mg dailyRivaroxaban 20 mg daily	0.88(0.74,1.05)0.88 (0.74,1.05)	0.88(0.74,1.04)0.88 (0.74,1.03)	2 fewer (5 fewer-1 more)2 fewer (1 more-5 fewer)	1.1%1.22 %	3.93.30	71.4%
Low Dose Aspirin (≤ 100 mg daily)No treatment/Placebo	2.03(1.32,3.12)1.84 (0.66,5.1)	1.87(1.26,2.8)1.53 (0.9,2.63)	14 more (5 more-29 more)9 more (26 more-2 fewer)	0.0%0.12 %	10.17.00	9.4%
Medium Dose Aspirin (> 100 mg and ≤ 300 mg daily)Low Dose ASA (≤ 100 mg daily)	1.21(0.51,2.87)2.03 (1.32,3.12)	1.35(0.74,2.46)1.87 (1.27,2.8)	6 more (5 fewer-24 more)14 more (29 more-5 more)	1.2%0.00 %	7.88.10	32.2%
Clopidogrel 75 mg OD & Low dose Aspirin (≤ 100 mg daily)Medium Dose ASA (> 100 mg and ≤ 300 mg daily)	1.93(1.41,2.64)0.83 (0.35,1.97)	1.93(1.42,2.64)1.35 (0.75,2.46)	15 more (7 more-26 more) 6 more (23 more-5 fewer)	0.0%1.14 %	10.26.20	8.3%
Edoxaban 30 mg dailyClopidogrel 75 mg daily & Low dose Aspirin (≤ 100 mg daily)	1.14(0.97,1.33)1.93 (1.41,2.64)	1.14(0.99,1.33)1.93 (1.42,2.65)	3 more (1 fewer-6 more) 15 more (26 more-7 more)	0.0%0.00 %	7.48.20	36.2%
Edoxaban 60 mg daily	0.87(0.74,1.02)	0.87(0.74,1.02)	3 fewer (5 fewer-1 more)	1.1%	3.8	72.1%
No treatment/Placebo	1.84(0.66,5.1)	1.53(0.9,2.61)	9 more (2 fewer-26 more)	0.1%	8.8	21.6%

CI = confidence interval; CrI = credible interval; OR= odds ratio

Formatted ... [1]
Formatted ... [5]
Formatted ... [2]
Formatted ... [3]
Formatted ... [4]
Formatted ... [6]
Formatted ... [7]
Formatted ... [8]
Formatted ... [10]
Formatted ... [11]
Formatted ... [9]
Formatted ... [12]
Formatted ... [13]
Formatted ... [14]
Formatted ... [15]
Formatted ... [17]
Formatted ... [18]
Formatted ... [16]
Formatted ... [19]
Formatted ... [20]
Formatted ... [21]
Formatted ... [22]
Formatted ... [24]
Formatted ... [25]
Formatted ... [23]
Formatted ... [26]
Formatted ... [27]
Formatted ... [28]
Formatted ... [29]
Formatted ... [31]
Formatted ... [32]
Formatted ... [30]
Formatted ... [33]
Formatted ... [34]
Formatted ... [35]
Formatted ... [36]
Formatted ... [38]
Formatted ... [39]
Formatted ... [37]
Formatted ... [40]
Formatted ... [41]
Formatted ... [42]
Formatted ... [43]
Formatted ... [45]
Formatted ... [46]
Formatted ... [44]
Formatted ... [47]
Formatted ... [48]
Formatted ... [49]
Formatted ... [50]
Formatted ... [52]
Formatted ... [53]
Formatted ... [51]
Formatted ... [54]
Formatted ... [55]
Formatted ... [56]
Formatted ... [57]
Formatted ... [59]

For peer review only - http://bmjopen.bmj.com/site/about/guidelines.xhtml

Appendix 7: Summary of results from Network Meta-analysis for Major Bleeding

Treatment	Pair-wise Meta-Analysis, OR (95% CI) versus standard adjusted dose VKA	NMA, Odds Ratio (95% CrI) versus standard adjusted dose VKA	NMA, Absolute Risk Difference per 1,000 treated (95% CrI) versus standard adjusted dose VKA	Probability Best (%) for Outcome	Rank for Outcome	SUCRA
Standard Adjusted-Dose VKAStandard Adjusted- dose VKA	NANA	NANA	NANA	0.0%0.00%	8.06.00	30.2%
Apixaban 5 mg twice dailyApixaban 5 mg twice daily	0.69(0.60,0.80)0.69 (0.6,0.79)	0.69(0.6,0.8)0.69 (0.6,0.8)	10 fewer (13 fewer-7 fewer)10 fewer (7 fewer-13 fewer)	0.0%10.13%	3.22.10	78.0%
Dabigatran 110 mg twice dailyDabigatran 110 mg twice daily	0.8(0.68,0.94)0.8 (0.68,0.94)	0.8(0.69,0.93)0.8 (0.69,0.93)	7 fewer (10 fewer-3 fewer)7 fewer (3 fewer-10 fewer)	0.0%0.84%	4.73.20	62.6%
Dabigatran 150 mg twice dailyDabigatran 150 mg twice daily	0.93(0.81,1.07)0.93 (0.81,1.07)	0.93(0.81,1.08) 0.93 (0.8,1.08)	3 fewer (7 fewer-3 more)3 fewer (3 more-7 fewer)	0.0%0.01%	6.84.90	41.6%
Rivaroxaban 20 mg dailyRivaroxaban 20 mg daily	1.03(0.9,1.18)1.03 (0.9,1.18)	1.03(0.89,1.19) 1.03 (0.89,1.19)	1 more (4 fewer-6 more)1 more (6 more-4 fewer)	0.0%0.00%	8.46.40	26.2%
Low Dose Aspirin (≤ 100 mg daily)No treatment/Placebo	1.01(0.57,1.78)0.39 (0.08,2.02)	1.05(0.59,1.86) 0.33 (0.08,1.08)	2 more (14 fewer-26 more)22 fewer (3 more-31 fewer)	0.1%87.97%	7.91.40	30.7%
Medium Dose Aspirin (> 100 mg and ≤ 300 mg daily)Low Dose ASA (≤ 100 mg daily)	1.68(0.50,5.69)1.01 (0.57,1.78)	1.79(0.62,5.64) 1.05 (0.6,1.86)	24 more (13 fewer-119 more)2 more (26 more-13 fewer)	0.0%0.96%	10.06.10	9.9%
Clopidogrel 75 mg OD & Low dose Aspirin (≤ 100 mg daily)Medium Dose ASA (> 100 mg and ≤ 300 mg daily)	1.1(0.82,1.48)1.89 (0.59,6.03)	1.1(0.83,1.47)1.78 (0.62,5.59)	4 more (6 fewer-15 more) 24 more (118 more-13 fewer)	0.0%0.09%	8.98.10	20.7%
Edoxaban 30 mg dailyClopidogrel 75 mg daily & Low dose Aspirin (≤ 100 mg daily)	0.47(0.4,0.55)1.1 (0.82,1.48)	0.47(0.4,0.54)1.1 (0.83,1.46)	18 fewer (20 fewer-15 fewer) 4 more (15 more-6 fewer)	29.3%0.02%	1.77.00	92.8%
Edoxaban 60 mg daily	0.78(0.68,0.89)	0.78(0.69,0.9)	7 fewer (10 fewer-4 fewer)	0.0%	4.5	64.7%
No treatment/Placebo	0.39(0.08,2.02)	0.33(0.08,1.08)	22 fewer (31 fewer-3 more)	70.6%	1.7	92.6%

BID= twice daily; CI = confidence interval; CrI = credible interval; OD= once daily; OR= odds ratio

Formatted Table ... [78]
Formatted ... [79]
Formatted ... [83]
Formatted ... [80]
Formatted ... [81]
Formatted ... [82]
Formatted ... [84]
Formatted ... [85]
Formatted ... [86]
Formatted ... [88]
Formatted ... [89]
Formatted ... [87]
Formatted ... [90]
Formatted ... [91]
Formatted ... [92]
Formatted ... [93]
Formatted ... [95]
Formatted ... [96]
Formatted ... [94]
Formatted ... [97]
Formatted ... [98]
Formatted ... [99]
Formatted ... [100]
Formatted ... [102]
Formatted ... [103]
Formatted ... [101]
Formatted ... [104]
Formatted ... [105]
Formatted ... [106]
Formatted ... [107]
Formatted ... [109]
Formatted ... [110]
Formatted ... [108]
Formatted ... [111]
Formatted ... [112]
Formatted ... [113]
Formatted ... [114]
Formatted ... [116]
Formatted ... [117]
Formatted ... [115]
Formatted ... [118]
Formatted ... [119]
Formatted ... [120]
Formatted ... [121]
Formatted ... [123]
Formatted ... [124]
Formatted ... [122]
Formatted ... [125]
Formatted ... [126]
Formatted ... [127]
Formatted ... [128]
Formatted ... [130]
Formatted ... [131]
Formatted ... [129]
Formatted ... [132]
Formatted ... [133]
Formatted ... [134]
Formatted ... [135]

For peer review only - http://bmjopen.bmj.com/site/about/guidelines.xhtml

Appendix 8: Benefit-Harm plot for each treatment (absolute harm difference per 1,000 patients treated). The absolute risk difference of recurrence of Stroke or systemic embolism relative to standard adjusted dose VKA is presented on the x-axis, while the absolute risk difference of major bleeding relative to standard adjusted dose VKA is on the y-axis. The dot is representative of the median estimate for each treatment while the 95% credible intervals are presented as error bars.

For peer review only

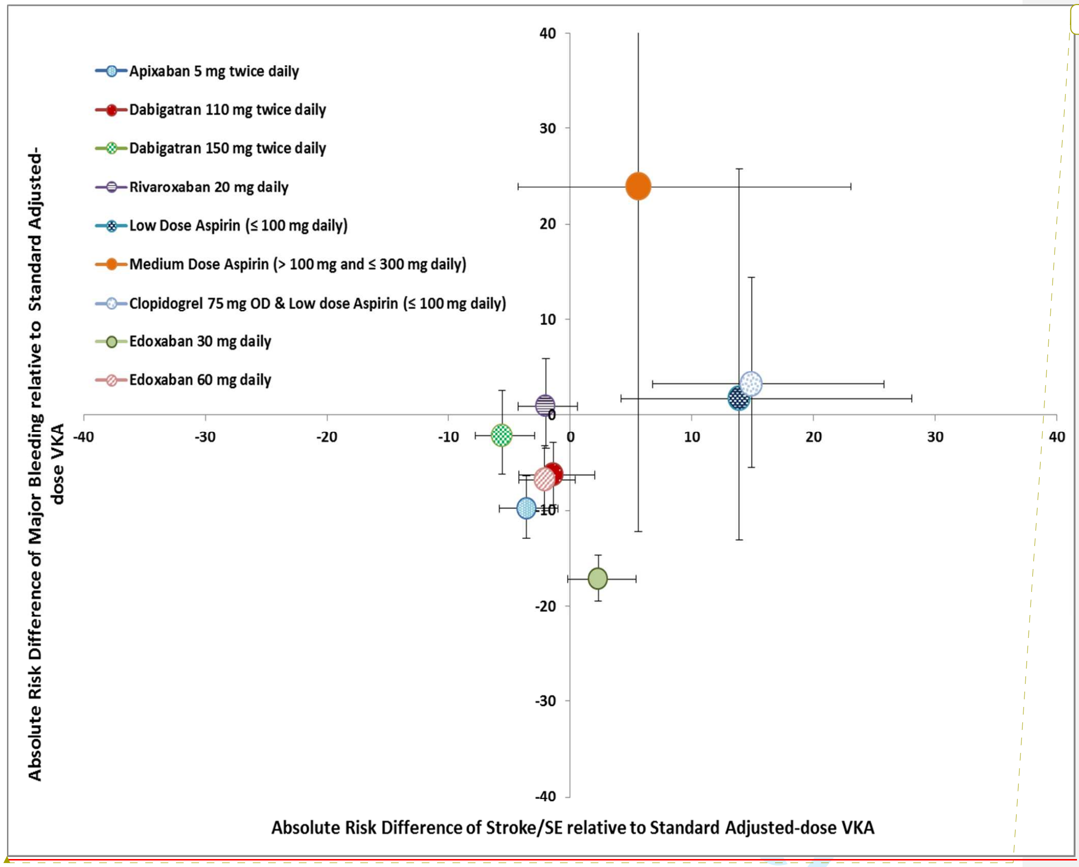

Formatted: Font:

For peer review only - http://bmjopen.bmj.com/site/about/guidelines.xhtml

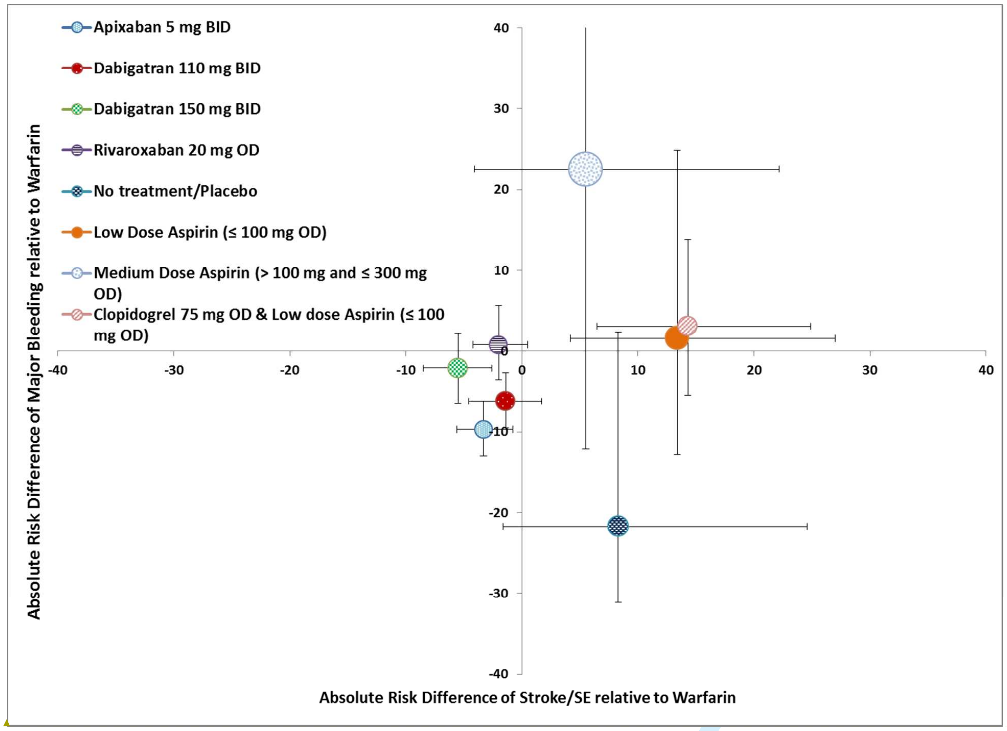

Formatted: Font:

Appendix 9: Rationale of choice of fixed-effects model and limitations

It is challenging to conduct network meta-analyses when evidence structures are comprised of single study connections, especially when there is obvious heterogeneity in the network. The use of a random-effects model with a vague prior on the between study variance exerts a large degree of influence on the credible intervals (CrI's) because there is insufficient studies to reign in the prior and provide an accurate estimate of the between study variance. Accordingly, we also considered informative priors on the between study variance from the recently published reports.[9,39] However, even when these priors were applied on the between study variance, they still did not reign in CrI's effect estimates again because the network was comprised of single study connections. The use of the random effects model with either a vague or informative prior was problematic because treatments that were originally associated with statistically significant reductions in stroke or SE relative to standard adjusted dose VKA were no longer associated with significant reductions, limiting external validity of the model (see Figures below on direct estimates in Online Appendix 4 and 5). While we could have opted to use less vague priors (e.g., ¼ of prior in Turner et al[9]), the choice of the prior would have seemed somewhat arbitrary because it was not based on any empirical evidence. As such, we opted to use the fixed-effects model despite the known issues in doing so. By using the fixed-effects model, treatments that were associated with significant reductions in the risk of stroke or SE relative to standard adjusted dose VKA retained significance. We felt that it would have been inappropriate to remove statistical significance by applying vague priors given the large investment involved in developing clinical trials. However, a consequence of retaining statistical significance is seen when comparing newer oral anticoagulants versus one another in Figure 5. Because we assumed the new oral anticoagulants were all associated with reductions in Stroke or SE relative to standard adjusted dose VKA (if that was the case in the underlying trials), significant reductions in the risk of stroke or SE among new oral anticoagulants are observed in Figure 5. This may or may not be the case in clinical practice. As noted in tables 1 and Online Appendix 2, there is considerable heterogeneity across studies. However, the network structure itself (i.e., single study connections), made it difficult to properly adjust for differences in patient and study characteristics using standard approaches. Accordingly, results in Figure 5 should be interpreted with caution. We conducted simple sub-group analyses (breaking randomization) to help adjust for this heterogeneity, which is a very crude way to adjust for heterogeneity. To fully elucidate the comparative benefits and harms of antithrombotic agents, especially across the various sub-populations, rigorously conducted comparative studies, or network meta-regression analyses of patient-level data are required.

For peer review only - http://bmjopen.bmj.com/site/about/guidelines.xhtml

Forest Plot – Odds ratio (95% Credible interval) when using vague prior on the sd (i.e, sd~unif(0,2))

Formatted: Font: (Default) Arial, 11 pt, Bold

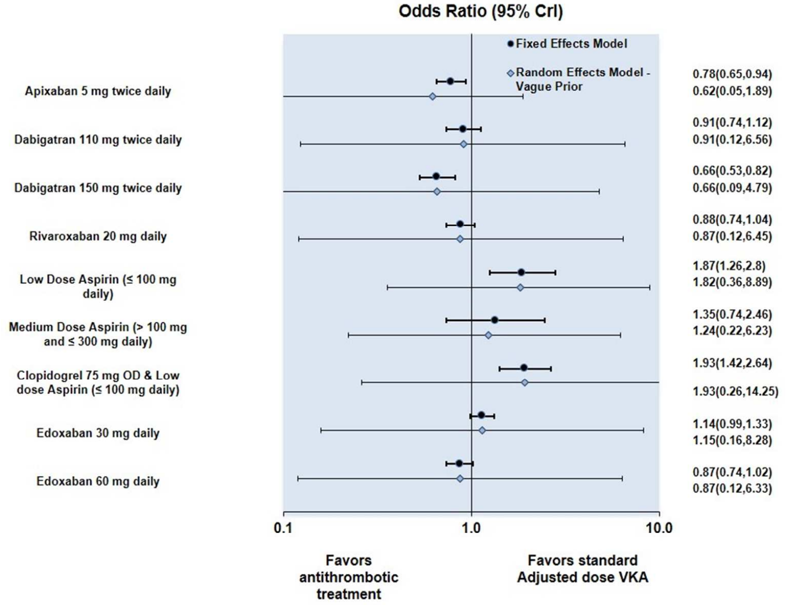

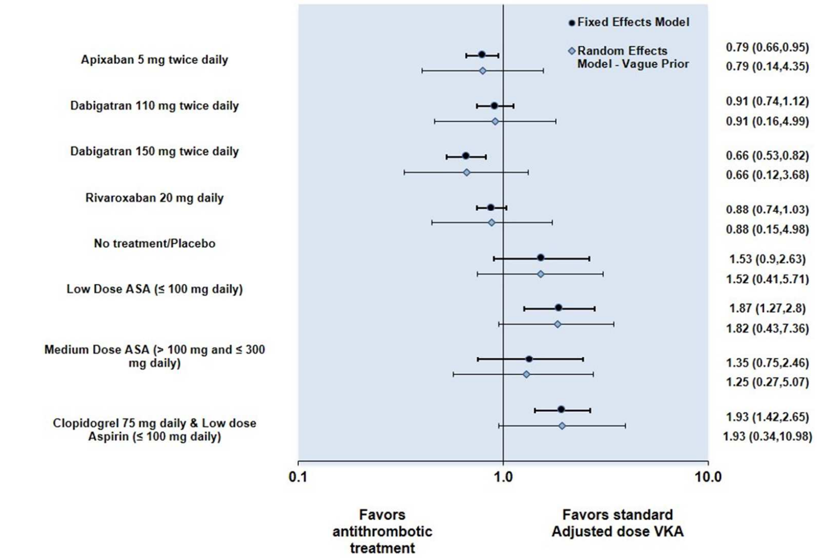

Forest Plot – Odds ratio (95% Credible interval) when using informative prior on the sd (i.e, dlnorm(-3.02,0.29)[9]

For peer review only - http://bmjopen.bmj.com/site/about/guidelines.xhtml

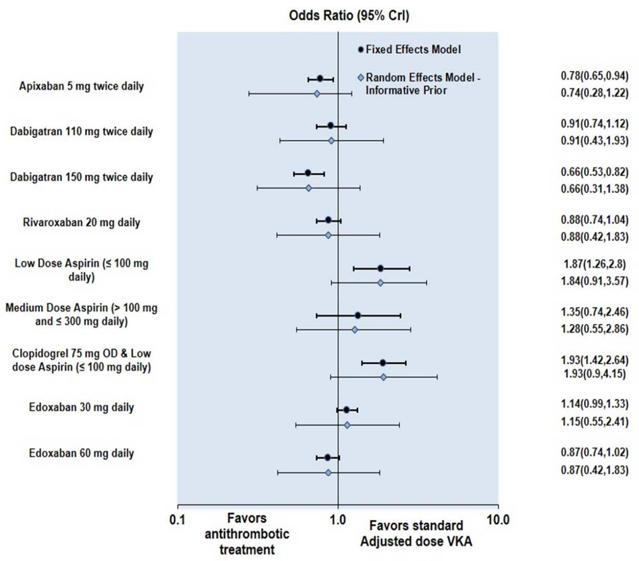

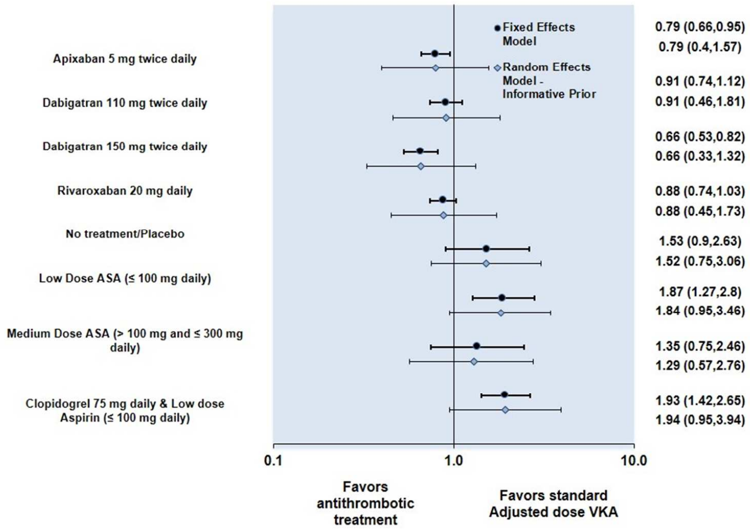

Formatted: Font: (Default) Arial, 11 pt, Bold

Appendix 10: PRISMA Checklist

Section/topic	#	Checklist item	Reported on page #
**TITLE**			
Title	1	Identify the report as a systematic review, meta-analysis, or both.	0
**ABSTRACT**			
Structured summary	2	Provide a structured summary including, as applicable: background; objectives; data sources; study eligibility criteria, participants, and interventions; study appraisal and synthesis methods; results; limitations; conclusions and implications of key findings; systematic review registration number.	0
**INTRODUCTION**			
Rationale	3	Describe the rationale for the review in the context of what is already known.	1, Appendix 13
Objectives	4	Provide an explicit statement of questions being addressed with reference to participants, interventions, comparisons, outcomes, and study design (PICOS).	1
**METHODS**			
Protocol and registration	5	Indicate if a review protocol exists, if and where it can be accessed (e.g., Web address), and, if available, provide registration information including registration number.	2
Eligibility criteria	6	Specify study characteristics (e.g., PICOS, length of follow-up) and report characteristics (e.g., years considered, language, publication status) used as criteria for eligibility, giving rationale.	2-3
Information sources	7	Describe all information sources (e.g., databases with dates of coverage, contact with study authors to identify additional studies) in the search and date last searched.	2-3
Search	8	Present full electronic search strategy for at least one database, including any limits used, such that it could be repeated.	2-3
Study selection	9	State the process for selecting studies (i.e., screening, eligibility, included in systematic review, and, if applicable, included in the meta-analysis).	3
Data collection process	10	Describe method of data extraction from reports (e.g., piloted forms, independently, in duplicate) and any processes for obtaining and confirming data from investigators.	3
Data items	11	List and define all variables for which data were sought (e.g., PICOS, funding sources) and any assumptions and simplifications made.	3-4
Risk of bias in individual studies	12	Describe methods used for assessing risk of bias of individual studies (including specification of whether this was done at the study or outcome level), and how this information is to be used in any data synthesis.	3-~~,~~6
Summary measures	13	State the principal summary measures (e.g., risk ratio, difference in means).	4~~3~~
Synthesis of results	14	Describe the methods of handling data and combining results of studies, if done, including measures of consistency (e.g., I^2) for each meta-analysis.	4-5
Risk of bias across studies	15	Specify any assessment of risk of bias that may affect the cumulative evidence (e.g., publication bias, selective reporting within studies).	7~~6~~
Additional analyses	16	Describe methods of additional analyses (e.g., sensitivity or subgroup analyses, meta-regression), if done, indicating which were pre-specified.	6-7~~5~~
**RESULTS**			
Study selection	17	Give numbers of studies screened, assessed for eligibility, and included in the review, with reasons for exclusions at each stage, ideally with a flow diagram.	~~3,~~5, Appendix 2

For peer review only - http://bmjopen.bmj.com/site/about/guidelines.xhtml

Study characteristics	18	For each study, present characteristics for which data were extracted (e.g., study size, PICOS, follow-up period) and provide the citations.	5-6, Table 1, Online Appendix 3-42
Risk of bias within studies	19	Present data on risk of bias of each study and, if available, any outcome level assessment (see item 12).	76
Results of individual studies	20	For all outcomes considered (benefits or harms), present, for each study: (a) simple summary data for each intervention group (b) effect estimates and confidence intervals, ideally with a forest plot.	Figure 23-4, Appendix 6-7
Synthesis of results	21	Present results of each meta-analysis done, including confidence intervals and measures of consistency.	Figure 2-4, Appendix 6-7Figure 3-4, Online Appendix 4 and 5
Risk of bias across studies	22	Present results of any assessment of risk of bias across studies (see Item 15).	76
Additional analysis	23	Give results of additional analyses, if done (e.g., sensitivity or subgroup analyses, meta-regression [see Item 16]).	Table 12-3, Online Appendix 9, 1210
**DISCUSSION**			
Summary of evidence	24	Summarize the main findings including the strength of evidence for each main outcome; consider their relevance to key groups (e.g., healthcare providers, users, and policy makers).	11-162
Limitations	25	Discuss limitations at study and outcome level (e.g., risk of bias), and at review-level (e.g., incomplete retrieval of identified research, reporting bias).	1413-154
Conclusions	26	Provide a general interpretation of the results in the context of other evidence, and implications for future research.	10-164
**FUNDING**			
Funding	27	Describe sources of funding for the systematic review and other support (e.g., supply of data); role of funders for the systematic review.	0

For peer review only

Appendix 11: Assessment of Inconsistency

We plotted the posterior mean deviance of the individual data points in the inconsistency model against their posterior mean deviance in the consistency model to help identify loops where inconsistency is present. In our analysis, the posterior mean deviance contributions are very similar and close to 1, for both models. The consistency model has a similar posterior mean of the residual deviance (26~~19~~.81~~28~~ vs. 26.72~~19.13~~) and DIC (183~~46~~.~~1~~59 vs. 185~~48~~.36~~04~~) and hence is a similar fit for both models. A few points show a lower value of the posterior mean deviance in the inconsistency model but these values are not too far from 1. Similarly, a few points have higher values of the posterior mean deviance in the consistency model (again with values not too far from 1) suggesting that each model may fit the data points better for certain points. The parameter estimates are also similar for both models and there is considerable overlap in the 95% credible intervals (see table below), suggesting no evidence of inconsistency in the network.

Plot of posterior mean deviance of the individual data points in the inconsistency model against their posterior mean deviance in the consistency model

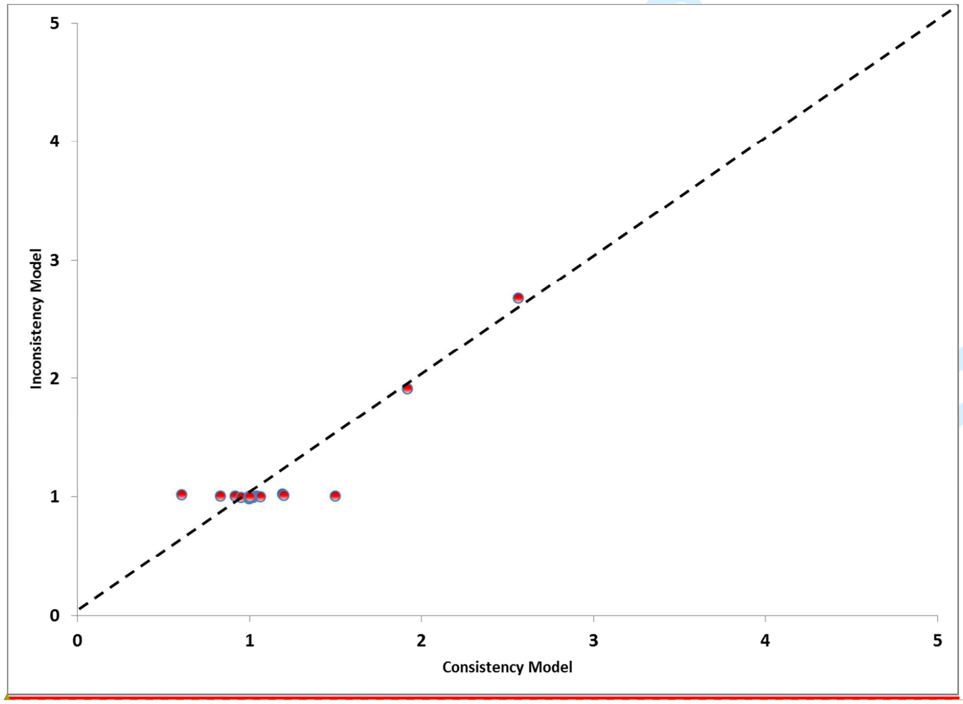

Formatted: Font: (Default) Arial, 11 pt, Bold

For peer review only

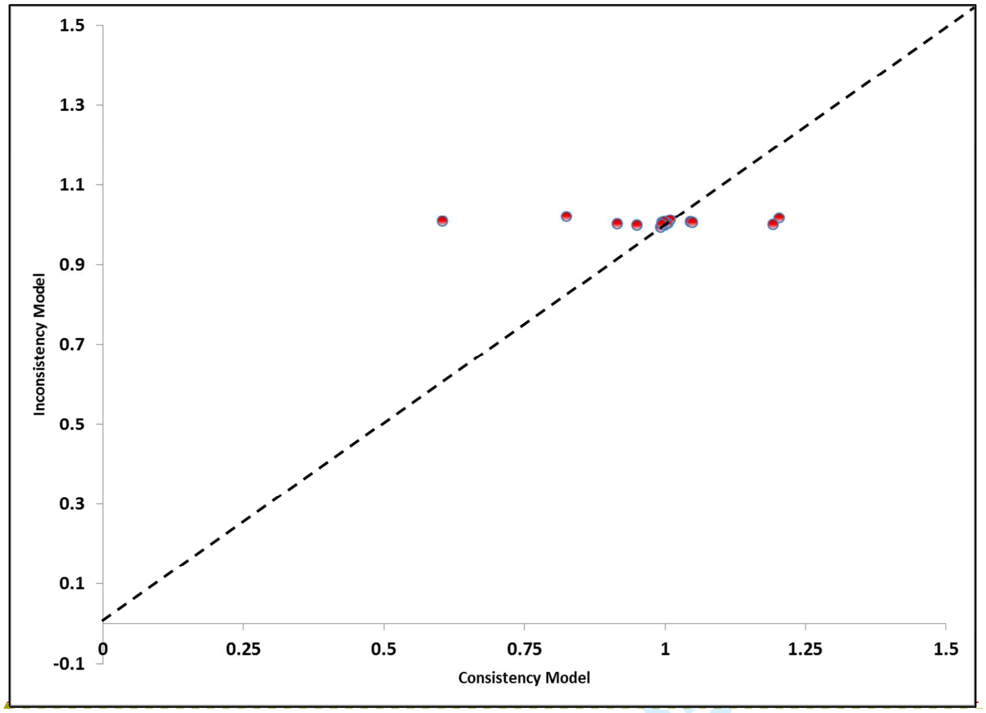

Formatted: Font: Bold

Comparison of effect estimates from consistency and inconsistency models where there is data available for comparison

Comparison	Consistency Model	Inconsistency Model
Apixaban 5 mg twice daily vs Standard Adjusted-Dose VKA~~Apixaban 5 mg twice daily vs Standard Standard adjusted dose VKA~~	0.78 (0.65,0.94)~~0.79 (0.66,0.95)~~	0.78 (0.65,0.94)~~0.79 (0.66,0.95)~~
Dabigatran 110 mg twice daily vs Apixaban 5 mg twice daily vs ~~Dabigatran 110 mg twice daily vs Standard adjusted dose VKA~~	0.91 (0.74,1.12)~~0.91 (0.74,1.12)~~	0.91 (0.74,1.12)~~0.91 (0.74,1.12)~~
Dabigatran 150 mg twice daily vs Standard Adjusted-Dose VKA~~Dabigatran 150 mg twice daily vs Standard adjusted dose VKA~~	0.66 (0.53,0.82)~~0.66 (0.53,0.82)~~	0.66 (0.53,0.82)~~0.66 (0.53,0.82)~~
Rivaroxaban 20 mg daily vs	0.88	0.88

Formatted Table

For peer review only - http://bmjopen.bmj.com/site/about/guidelines.xhtml

Standard Adjusted-Dose VKA~~Rivaroxaban 20 mg daily vs Standard adjusted dose VKA~~	(0.74,1.04)~~0.88 (0.74,1.03)~~	(0.74,1.04)~~0.88 (0.74,1.03)~~
Low Dose Aspirin (≤ 100 mg daily) vs Standard Adjusted-Dose VKA~~No treatment/Placebo vs Standard adjusted dose VKA~~	1.87 (1.26,2.8)~~1.53 (0.9,2.63)~~	2.04 (1.33,3.19)~~1.89 (0.7,5.72)~~
Medium Dose Aspirin (> 100 mg and ≤ 300 mg daily) vs Standard Adjusted-Dose VKA~~Low Dose Aspirin (≤ 100 mg daily) vs Standard adjusted dose VKA~~	1.35 (0.74,2.46)~~1.87 (1.27,2.8)~~	0.82 (0.33,1.97)~~2.04 (1.33,3.19)~~
Clopidogrel 75 mg OD & Low dose Aspirin (≤ 100 mg daily) vs Standard Adjusted-Dose VKA~~Medium Dose Aspirin (> 100 mg and ≤ 300 mg daily) vs Standard adjusted dose VKA~~	1.93 (1.42,2.64)~~1.35 (0.75,2.46)~~	1.93 (1.42,2.64)~~0.82 (0.33,1.99)~~
Edoxaban 30 mg daily vs Standard Adjusted-Dose VKA~~Clopidogrel 75 mg daily & Low dose Aspirin (≤ 100 mg daily) vs Standard adjusted dose VKA~~	1.14 (0.99,1.33)~~1.93 (1.42,2.65)~~	1.14(0.98,1.33)~~1.93 (1.42,2.64)~~
Edoxaban 60 mg daily vs Standard Adjusted-Dose VKA~~Low Dose Aspirin (≤ 100 mg daily) vs No treatment/Placebo~~	0.87 (0.74,1.02)~~1.22 (0.72,2.08)~~	0.87 (0.74,1.02)~~1.00 (0.51,1.98)~~
No treatment/Placebo vs Standard Adjusted-Dose VKA~~Medium Dose Aspirin (> 100 mg and ≤ 300 mg daily) vs No treatment/Placebo~~	1.53 (0.9,2.61)~~0.88 (0.55,1.41)~~	1.89 (0.7,5.71)~~1.05 (0.62,1.76)~~
No treatment/Placebo vs Low Dose Aspirin (≤ 100 mg daily)	0.82 (0.48,1.38)	1 (0.51,1.98)
Residual deviance	26~~19~~.81~~28~~	26~~19~~.72~~13~~
DIC	183~~46~~.59~~15~~	185.36~~48.044~~

For peer review only

Appendix 12: Sensitivity analysis with AVERROES and ACTIVE A

A. Stroke or SE

Evidence Networks for Stroke or SE NMA including AVEROES and ACTIVE A studies

Formatted: Font: Bold

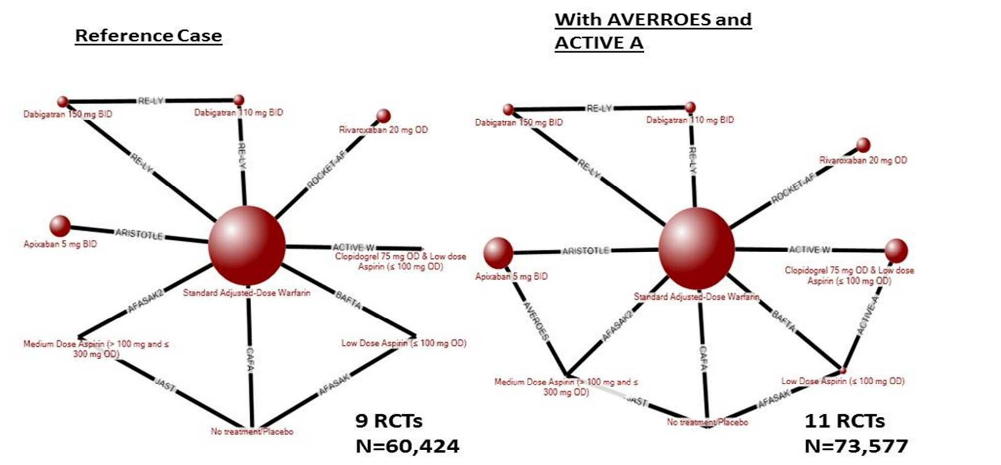

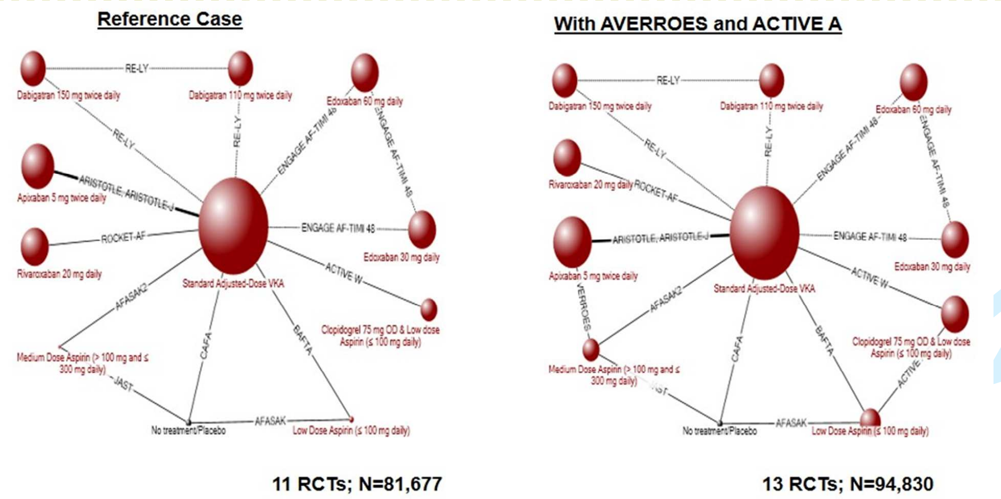

Forest Plot for Stroke or SE NMA including AVERROES

For peer review only - http://bmjopen.bmj.com/site/about/guidelines.xhtml

and ACTIVE A studies

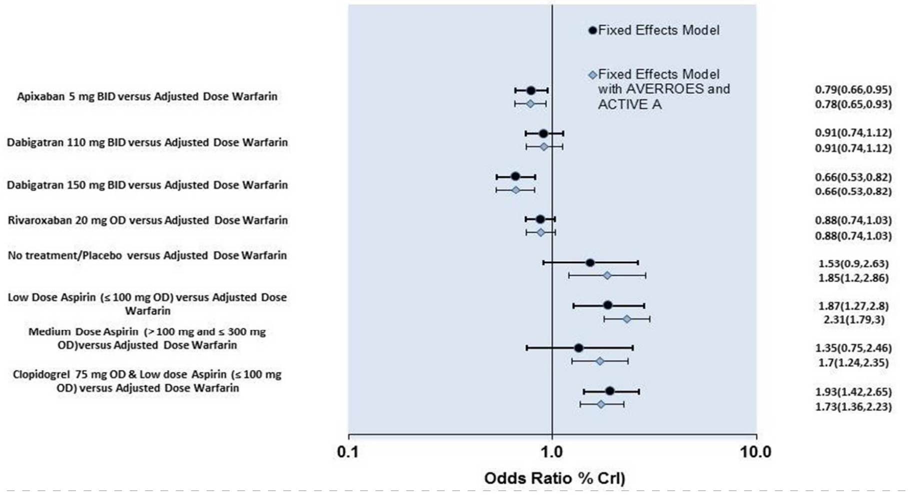

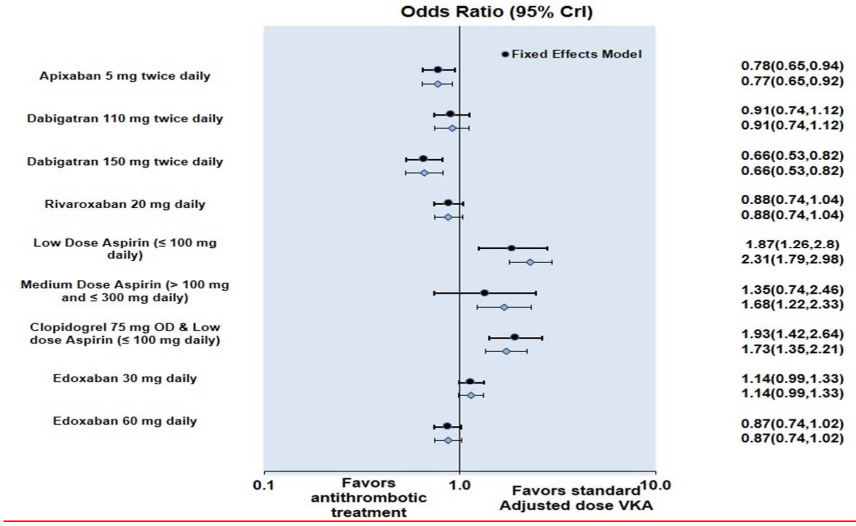

Formatted: Font: Bold

B. Major Bleeding

Evidence Networks for Major Bleeding NMA including AVERROES and ACTIVE A studies

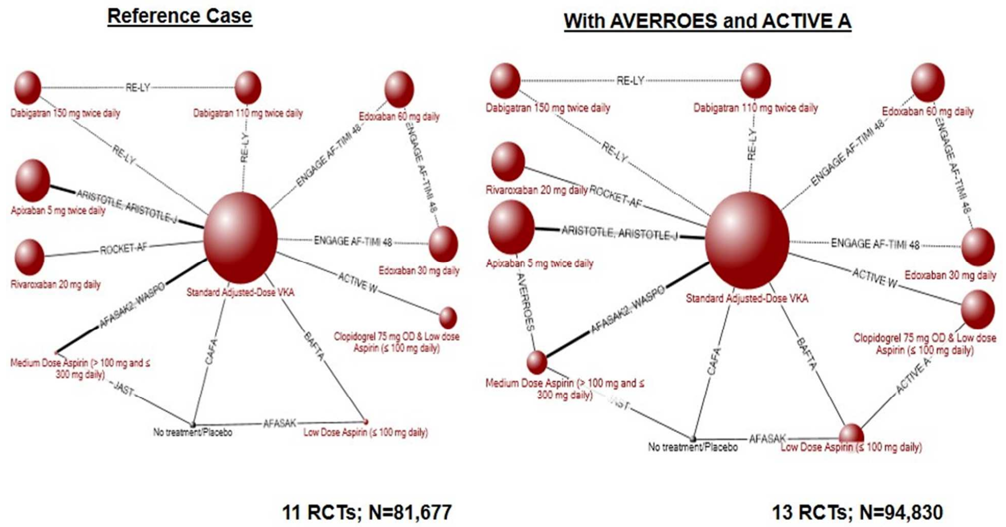

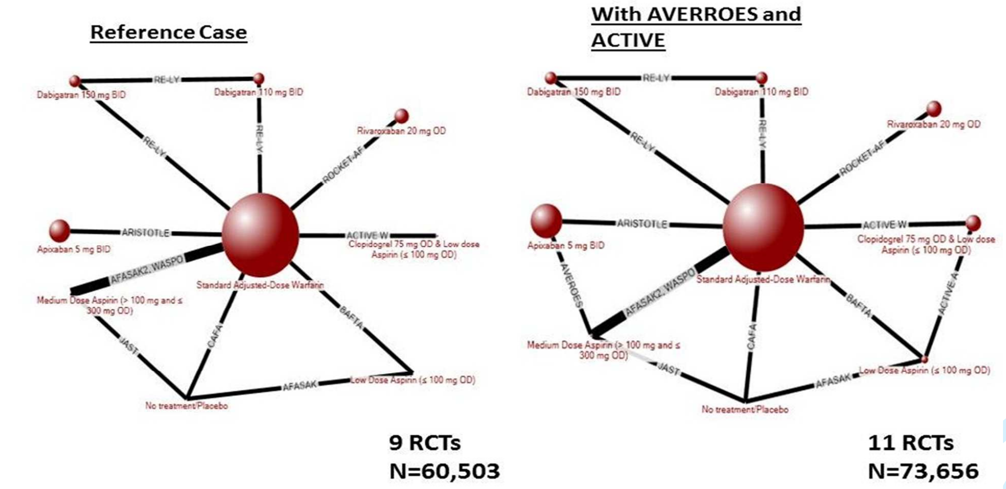

Forest Plot for Major Bleeding NMA including AVERROES and ACTIVE A studies

Formatted: Font: Bold

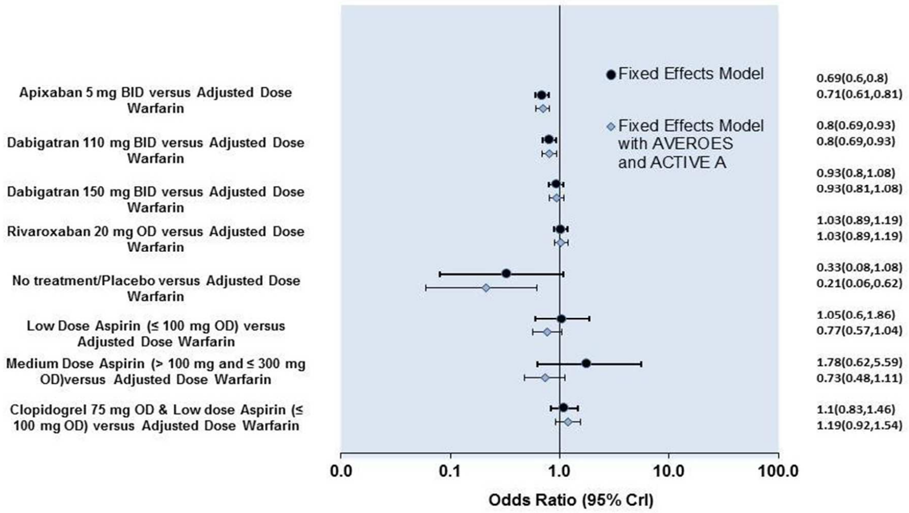

Odds Ratio (95% CrI)

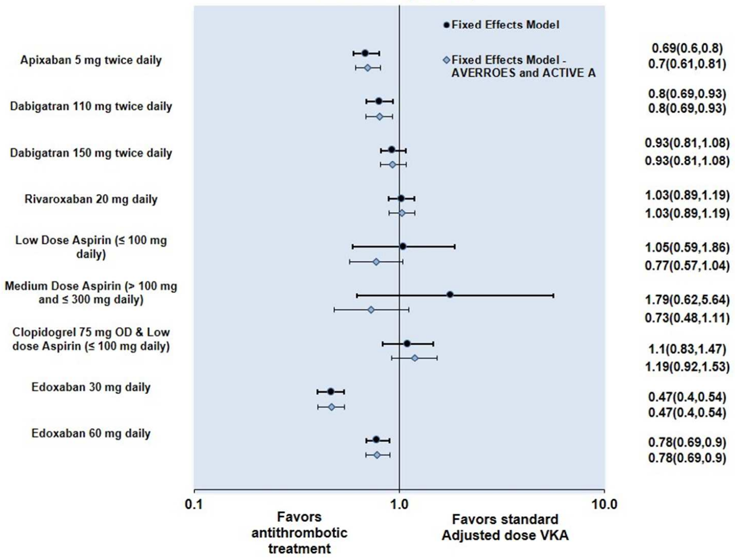

Formatted: Font: Bold

Formatted: Font: (Default) Arial, 11 pt

Formatted: Centered

Appendix 13: Comparison with previously published network meta-analyses since Harenberg et al[25]

Author (Publication Year)	Method	Search Date	Included Treatments	Registered Protocol	Relative Measure(s)	Absolute Measure(s) reported	Subgroup Analyses Reported
Cameron 2014	Bayesian Network Meta-analysis	January 2014	dabigatran, rivaroxaban, apixaban, edoxaban, and vitamin K antagonists (VKA) at a standard adjusted dose (target international normalised ratio 2.0-3.0), acetylsalicylic acid, acetylsalicylic acid and clopidogrel	Yes (PROSPERO registry - CRD42012002721)	OR (95% CrI)	Yes	Yes
Ruff 2013	Traditional Meta-analysis	November 2013	dabigatran, rivaroxaban, apixaban, edoxaban	Not Reported	RR (95%CI)	No	Yes
Dogliotti 2013	Bayesian Network Meta-analysis	May 2012	dabigatran, rivaroxaban, apixaban, vitamin K antagonists (VKA) at a standard adjusted dose, acetylsalicylic acid, acetylsalicylic acid and clopidogrel	Yes (metacardio.org)	OR (95% CrI)	No	No
Assiri 2013	Baysian Network Meta-analysis	August 2012	dabigatran, rivaroxaban, apixaban, vitamin K antagonists (VKA) at a standard adjusted dose, acetylsalicylic acid, acetylsalicylic acid and clopidogrel	Not Reported	RR (95%CrI)	No	No
Bruins 2013	Traditional Meta-analysis	June 2013	antistasin, apixaban, betrixaban, darexaban, DU176b, edoxaban, eribaxaban, fondaparinux, idraparinux, otamixaban, razaxaban, rivaroxaban, yagin, YM150, LY517717, SSR126517E, vitamin K antagonists (warfarin and congeners) with the intensity of anticoagulation dose-adjusted using the International Normalised Ratio (INR)	Yes (Cochrane Library)	OR (95%CI) RR (95%CI) in summary table	Yes	Yes

For peer review only - http://bmjopen.bmj.com/site/about/guidelines.xhtml

Formatted ... [156]
Formatted ... [157]
Formatted ... [159]
Formatted ... [160]
Formatted ... [161]
Formatted ... [162]
Formatted ... [163]
Formatted ... [164]
Formatted ... [165]
Formatted ... [158]
Formatted ... [166]
Formatted ... [167]
Formatted ... [168]
Formatted ... [169]
Formatted ... [170]
Formatted ... [172]
Formatted ... [174]
Formatted ... [175]
Formatted ... [171]
Formatted ... [173]
Formatted ... [176]
Formatted ... [178]
Formatted ... [179]
Formatted ... [180]
Formatted ... [181]
Formatted ... [182]
Formatted ... [184]
Formatted ... [185]
Formatted ... [177]
Formatted ... [183]
Formatted ... [186]
Formatted ... [188]
Formatted ... [189]
Formatted ... [190]
Formatted ... [191]
Formatted ... [193]
Formatted ... [195]
Formatted ... [196]
Formatted ... [187]
Formatted ... [192]
Formatted ... [194]
Formatted ... [197]
Formatted ... [199]
Formatted ... [200]

Appendix 14: Excluded Study List

Inappropriate Study Design

Cardiovasc J S Afr. 2006 Mar;17(2):91.
J Fam Pract. 2006;55(9):753.
Cardiovasc J Afr. 2009 May;20(3):210.
Cardiovasc J Afr. 2009 Sep;20(5):311-2.
Australian Journal of Pharmacy. 2009;90(1073):68.
Formulary. 2010;45(1):16.
Aalbers J. Cardiovasc J Afr. 2010 Nov;21(6):342-3.
Alexander W, et al. PT. 2010;35(10):580-1.
Belavic JM. Nurse Pract. 2011 Sep;36(9):6-7.
Camm AJ. Eur Heart J. 2009 Nov;30(21):2554-5.
Chua D, et al. N Engl J Med. 2009;361(13):1312-3.
Connolly SJ. Eur J Heart Fail. 2009;11(12):1215.
DeSilvey DL. Am J Geriatr Cardiol. 2006 Sep;15(5):326-7.
Dunn A. Ann Intern Med. 2011;154(8):JC4-3.
Eikelboom JW, et al. J Thromb Haemost. 2012;10(5):966-8.
Ezekowitz MD, et al. J Interv Card Electrophysiol. 2011 Dec;32(3):173-80.
Garcia D, et al. Lancet. 2007 Aug 11;370(9586):460-1.
Granger CB. Ann Intern Med. 2012;156(JC1-2):JC1-3.
Hart RG, et al. Ann Intern Med. 2007;147(8):590-2.
Katona A, et al. Eur Heart J. 2006 Jun;27(11):1382.
Kita K, et al. Interventional Cardiology. 2011;3(6):637.
Lopes RD, et al. Evid Based Med. 2011;16(6):187-8.
Nolan J, et al. Br Heart J. 1992 Dec;68(6):544-7.
Paikin JS, et al. Expert Rev Cardiovasc Ther. 2011 Mar;9(3):279-86.
Panichpisal K, et al. Future Neurology. 2011;6(2):155-8.
Roy B, et al. Am J Cardiol. 2012 Feb 1;109(3):370-7.
Sloan MA. Evid Based Med. 2006;11(6):170.
Vassiliou VS. N Engl J Med. 2012;366(1):88-90.
Veloso HH, et al. Chest. 2005 Jul;128(1):475.
Weinberger J. Curr Cardiol Rep. 2010;12(1):1-2.

Inappropriate Intervention

Lancet. 1994 Mar 19;343(8899):687-91.
The Stroke Prevention in Atrial Fibrillation Investigators. Arch Intern Med. 1996 Feb 26;156(4):409-16.
Archer SL, et al. Am Heart J. 1995 Aug;130(2):287-95.
Edvardsson N, et al. J Intern Med. 2003 Jul;254(1):95-101.
Hart RG, et al. Cerebrovasc Dis. 2000 Jan;10(1):39-43.
Hellemons BS, et al. Control Clin Trials. 1999 Aug;20(4):386-93.
Hori M, et al. Circ J. 2012 Jun 5.
Miller VT, et al. Neurology. 1996 Jan;46(1):238-40.
Perez-Gomez F, et al. Eur Heart J. 2007;28(8):996-1003.

For peer review only - http://bmjopen.bmj.com/site/about/guidelines.xhtml

Singer DE, et al. Am Heart J. 1992 Dec;124(6):1567-73.
Connolly S, et al. Eur Heart J. 2013 May:34(20):1498-505

Inappropriate Population
ACTIVE Investigators, et al. N Engl J Med 2009;360(20):2066-78.
Circulation 1991;84(2):527-39.
Connolly SJ, et al. N Engl J Med 2011;364(9):806-17.
Dewilde W, et al. Am Heart J. 2009 Nov;158(5):713-8.
Diener HC, et al. Lancet Neurol. 2012 Mar;11(3):225-31.
EAFT (European Atrial Fibrillation Trial) Study Group. Lancet. 1993;342(8882):1255-62.
Eikelboom JW, et al. J Stroke Cerebrovasc Dis. 2012 Aug;21(6):429-35.
ESPRIT Study Group, et al. Lancet Neurol. 2007 Feb;6(2):115-24.
Hart RG, et al. Cerebrovasc Dis. 2008;25(4):344-7.
N Engl J Med 1990;322(12):863-8.
Wang TH, et al. Eur Heart J. 2007 Sep;28(18):2200-7.

Inappropriate Outcomes
De Caterina R, et al. Eur Heart J. 2010 Sep;31(17):2133-40.
Ezekowitz MD, et al. Circulation. 1995 Oct 15;92(8):2178-82.
Lorenzoni R, et al. Am Heart J. 2004 Jul;148(1):e6.

Others
Cardiovasc J Afr. 2011 May;22(3):164.
Active Steering Committee, et al. Am Heart J. 2006 Jun;151(6):1187-93.
ROCKET AF Study Investigators. Am Heart J. 2010 Mar;159(3):340-7.
The Stroke Prevention in Atrial Fibrillation Investigators. Stroke. 1990 Apr;21(4):538-45.
Boysen G, et al. Stroke. 1990;21(Suppl I):I-92.
De Schryver EL. Cerebrovasc Dis. 2000 Mar;10(2):147-50.
Desai A. Clinical Trials Registry - India (CTRI). 2006.
Eikelboom JW, et al. Am Heart J. 2010 Mar;159(3):348-53.
Ezekowitz MD, et al. Am Heart J. 2009 May;157(5):805-10, 810.e1-2.
Flaker G, et al. J Am Coll Cardiol. 2012 Feb 28;59(9):854-5.
Lopes RD, et al. Lancet. 2012 Oct 2. Epub ahead of print.
Lopes RD, et al. Am Heart J. 2010 Mar;159(3):331-9.
Mant J, et al. Cerebrovasc Dis. 2007;23(Suppl 2):10.
Mant J, et al. Cardiol Rev. 2008;25(7):32-6.
Mant JW, et al. BMC Cardiovasc Disord. 2003 Aug 26;3:9.
Posada IS, et al. Am Heart J. 1999 Jul;138(1 Pt 1):137-43.
Van Latum JC. Ned Tijdschr Geneeskd. 1994;138(20):1025-31.
Yigit Z. Turk Kardiyoloji Dernegi Arsivi. 2000;28(1):8-19+4.

For peer review only

For peer review only - http://bmjopen.bmj.com/site/about/guidelines.xhtml

Appendix 15: Completion rates

Formatted: Font: Bold

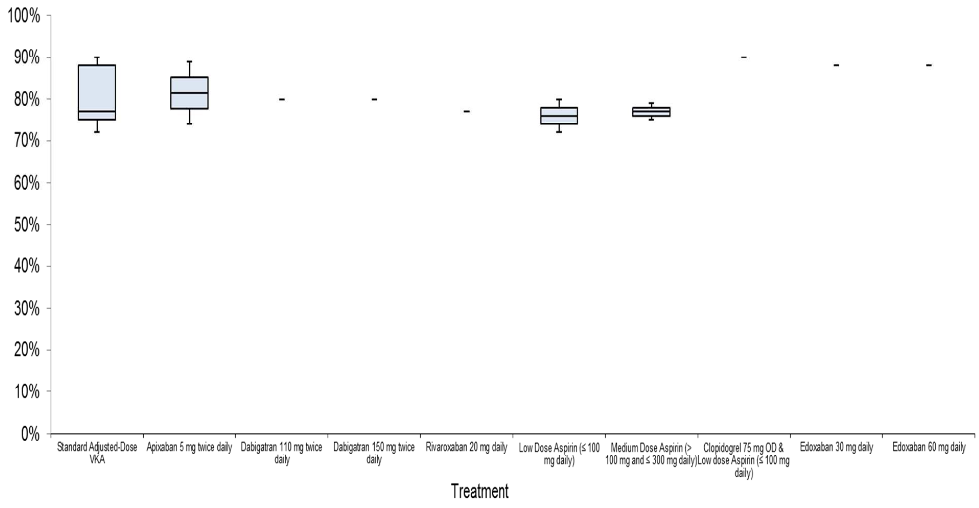

For peer review only - http://bmjopen.bmj.com/site/about/guidelines.xhtml

For peer review only

Page 31: [1] Formatted **Chris Cameron** **2/4/2014 3:36:00 PM**

Font: (Default) Arial, 8 pt

Page 31: [2] Formatted **Chris Cameron** **2/4/2014 3:36:00 PM**

Font: (Default) Arial, 8 pt

Page 31: [3] Formatted **Chris Cameron** **2/4/2014 3:36:00 PM**

Font: (Default) Arial, 8 pt

Page 31: [4] Formatted **Chris Cameron** **2/4/2014 3:36:00 PM**

Font: (Default) Arial, 8 pt

Page 31: [5] Formatted **Chris Cameron** **2/4/2014 3:36:00 PM**

Font: (Default) Arial, 8 pt

Page 31: [6] Formatted **Chris Cameron** **2/4/2014 3:36:00 PM**

Font: (Default) Arial, 8 pt

Page 31: [7] Formatted **Chris Cameron** **2/4/2014 3:36:00 PM**

Font: (Default) Arial, 8 pt

Page 31: [8] Formatted **Chris Cameron** **2/4/2014 3:36:00 PM**

Font: (Default) Arial, 8 pt

Page 31: [9] Formatted **Chris Cameron** **2/4/2014 3:36:00 PM**

Font: (Default) Arial, 8 pt

Page 31: [10] Formatted **Chris Cameron** **2/4/2014 3:36:00 PM**

Font: (Default) Arial, 8 pt

Page 31: [11] Formatted **Chris Cameron** **2/4/2014 3:36:00 PM**

Font: (Default) Arial, 8 pt

Page 31: [12] Formatted **Chris Cameron** **2/4/2014 3:36:00 PM**

Font: (Default) Arial, 8 pt

Page 31: [13] Formatted **Chris Cameron** **2/4/2014 3:36:00 PM**

Font: (Default) Arial, 8 pt

Page 31: [14] Formatted **Chris Cameron** **2/4/2014 3:36:00 PM**

Font: (Default) Arial, 8 pt

Page 31: [15] Formatted **Chris Cameron** **2/4/2014 3:36:00 PM**

Font: (Default) Arial, 8 pt

Page 31: [16] Formatted **Chris Cameron** **2/4/2014 3:36:00 PM**

Font: (Default) Arial, 8 pt

Page 31: [17] Formatted **Chris Cameron** **2/4/2014 3:36:00 PM**

Font: (Default) Arial, 8 pt

Page 31: [18] Formatted **Chris Cameron** **2/4/2014 3:36:00 PM**

Font: (Default) Arial, 8 pt

For peer review only - http://bmjopen.bmj.com/site/about/guidelines.xhtml

Page 31: [19] Formatted **Chris Cameron** **2/4/2014 3:36:00 PM**

Font: (Default) Arial, 8 pt

Font: (Default) Arial, 8 pt

Page 31: [21] Formatted **Chris Cameron** **2/4/2014 3:36:00 PM**

Font: (Default) Arial, 8 pt

Page 31: [22] Formatted **Chris Cameron** **2/4/2014 3:36:00 PM**

Font: (Default) Arial, 8 pt

Page 31: [23] Formatted **Chris Cameron** **2/4/2014 3:36:00 PM**

Font: (Default) Arial, 8 pt

Page 31: [24] Formatted **Chris Cameron** **2/4/2014 3:36:00 PM**

Font: (Default) Arial, 8 pt

Page 31: [25] Formatted **Chris Cameron** **2/4/2014 3:36:00 PM**

Font: (Default) Arial, 8 pt

Page 31: [26] Formatted **Chris Cameron** **2/4/2014 3:36:00 PM**

Font: (Default) Arial, 8 pt

Page 31: [27] Formatted **Chris Cameron** **2/4/2014 3:36:00 PM**

Font: (Default) Arial, 8 pt

Page 31: [28] Formatted **Chris Cameron** **2/4/2014 3:36:00 PM**

Font: (Default) Arial, 8 pt

Page 31: [29] Formatted **Chris Cameron** **2/4/2014 3:36:00 PM**

Font: (Default) Arial, 8 pt

Page 31: [30] Formatted **Chris Cameron** **2/4/2014 3:36:00 PM**

Font: (Default) Arial, 8 pt

Page 31: [31] Formatted **Chris Cameron** **2/4/2014 3:36:00 PM**

Font: (Default) Arial, 8 pt

Page 31: [32] Formatted **Chris Cameron** **2/4/2014 3:36:00 PM**

Font: (Default) Arial, 8 pt

Page 31: [33] Formatted **Chris Cameron** **2/4/2014 3:36:00 PM**

Font: (Default) Arial, 8 pt

Page 31: [34] Formatted **Chris Cameron** **2/4/2014 3:36:00 PM**

Font: (Default) Arial, 8 pt

Page 31: [35] Formatted **Chris Cameron** **2/4/2014 3:36:00 PM**

Font: (Default) Arial, 8 pt

Page 31: [36] Formatted **Chris Cameron** **2/4/2014 3:36:00 PM**

Font: (Default) Arial, 8 pt

Page 31: [37] Formatted **Chris Cameron** **2/4/2014 3:36:00 PM**

Font: (Default) Arial, 8 pt

Page 31: [38] Formatted **Chris Cameron** **2/4/2014 3:36:00 PM**

Font: (Default) Arial, 8 pt

Page 31: [39] Formatted **Chris Cameron** **2/4/2014 3:36:00 PM**

For peer review only

For peer review only - http://bmjopen.bmj.com/site/about/guidelines.xhtml

Page 31: [40] Formatted	Chris Cameron	2/4/2014 3:36:00 PM

Font: (Default) Arial, 8 pt

Page 31: [41] Formatted	Chris Cameron	2/4/2014 3:36:00 PM

Font: (Default) Arial, 8 pt

Page 31: [42] Formatted	Chris Cameron	2/4/2014 3:36:00 PM

Font: (Default) Arial, 8 pt

Page 31: [43] Formatted	Chris Cameron	2/4/2014 3:36:00 PM

Font: (Default) Arial, 8 pt

Page 31: [44] Formatted	Chris Cameron	2/4/2014 3:36:00 PM

Font: (Default) Arial, 8 pt

Page 31: [45] Formatted	Chris Cameron	2/4/2014 3:36:00 PM

Font: (Default) Arial, 8 pt

Page 31: [46] Formatted	Chris Cameron	2/4/2014 3:36:00 PM

Font: (Default) Arial, 8 pt

Page 31: [47] Formatted	Chris Cameron	2/4/2014 3:36:00 PM

Font: (Default) Arial, 8 pt

Page 31: [48] Formatted	Chris Cameron	2/4/2014 3:36:00 PM

Font: (Default) Arial, 8 pt

Page 31: [49] Formatted	Chris Cameron	2/4/2014 3:36:00 PM

Font: (Default) Arial, 8 pt

Page 31: [50] Formatted	Chris Cameron	2/4/2014 3:36:00 PM

Font: (Default) Arial, 8 pt

Page 31: [51] Formatted	Chris Cameron	2/4/2014 3:36:00 PM

Font: (Default) Arial, 8 pt

Page 31: [52] Formatted	Chris Cameron	2/4/2014 3:36:00 PM

Font: (Default) Arial, 8 pt

Page 31: [53] Formatted	Chris Cameron	2/4/2014 3:36:00 PM

Font: (Default) Arial, 8 pt

Page 31: [54] Formatted	Chris Cameron	2/4/2014 3:36:00 PM

Font: (Default) Arial, 8 pt

Page 31: [55] Formatted	Chris Cameron	2/4/2014 3:36:00 PM

Font: (Default) Arial, 8 pt

Page 31: [56] Formatted	Chris Cameron	2/4/2014 3:36:00 PM

Font: (Default) Arial, 8 pt

Page 31: [57] Formatted	Chris Cameron	2/4/2014 3:36:00 PM

Font: (Default) Arial, 8 pt

Page 31: [58] Formatted	Chris Cameron	2/4/2014 3:36:00 PM

Font: (Default) Arial, 8 pt

For peer review only

Font: (Default) Arial, 8 pt

Page 31: [60] Formatted **Chris Cameron** **2/4/2014 3:36:00 PM**

Font: (Default) Arial, 8 pt

Page 31: [61] Formatted **Chris Cameron** **2/4/2014 3:36:00 PM**

Font: (Default) Arial, 8 pt

Page 31: [62] Formatted **Chris Cameron** **2/4/2014 3:36:00 PM**

Font: (Default) Arial, 8 pt

Page 31: [63] Formatted **Chris Cameron** **2/4/2014 3:36:00 PM**

Font: (Default) Arial, 8 pt

Page 31: [64] Formatted **Chris Cameron** **2/4/2014 3:36:00 PM**

Font: (Default) Arial, 8 pt

Page 31: [65] Formatted **Chris Cameron** **2/4/2014 3:36:00 PM**

Font: (Default) Arial, 8 pt

Page 31: [66] Formatted **Chris Cameron** **2/4/2014 3:36:00 PM**

Font: (Default) Arial, 8 pt

Page 31: [67] Formatted **Chris Cameron** **2/4/2014 3:36:00 PM**

Font: (Default) Arial, 8 pt

Page 31: [68] Formatted **Chris Cameron** **2/4/2014 3:36:00 PM**

Font: (Default) Arial, 8 pt

Page 31: [69] Formatted **Chris Cameron** **2/4/2014 3:36:00 PM**

Font: (Default) Arial, 8 pt

Page 31: [70] Formatted **Chris Cameron** **2/4/2014 3:36:00 PM**

Font: (Default) Arial, 8 pt

Page 31: [71] Formatted **Chris Cameron** **2/4/2014 3:36:00 PM**

Font: (Default) Arial, 8 pt

Page 31: [72] Formatted **Chris Cameron** **2/4/2014 3:36:00 PM**

Font: (Default) Arial, 8 pt

Page 31: [73] Formatted **Chris Cameron** **2/4/2014 3:36:00 PM**

Font: (Default) Arial, 8 pt

Page 31: [74] Formatted **Chris Cameron** **2/4/2014 3:36:00 PM**

Font: (Default) Arial, 8 pt

Page 31: [75] Formatted **Chris Cameron** **2/4/2014 3:36:00 PM**

Font: (Default) Arial, 8 pt

Page 31: [76] Formatted **Chris Cameron** **2/4/2014 3:36:00 PM**

Font: (Default) Arial, 8 pt

Page 31: [77] Formatted **Chris Cameron** **2/4/2014 3:36:00 PM**

Font: (Default) Arial, 8 pt

Page 32: [78] Formatted Table **Chris Cameron** **2/4/2014 [illegible] PM**

For peer review only

For peer review only

Page 32: [79] Formatted **Chris Cameron** **2/4/2014 7:09:00 PM**

Font: (Default) Arial, 8 pt

Page 32: [80] Formatted **Chris Cameron** **2/4/2014 7:09:00 PM**

Font: (Default) Arial, 8 pt

Page 32: [81] Formatted **Chris Cameron** **2/4/2014 7:09:00 PM**

Font: (Default) Arial, 8 pt

Page 32: [82] Formatted **Chris Cameron** **2/4/2014 7:09:00 PM**

Font: (Default) Arial, 8 pt

Page 32: [83] Formatted **Chris Cameron** **2/4/2014 7:09:00 PM**

Font: (Default) Arial, 8 pt

Page 32: [84] Formatted **Chris Cameron** **2/4/2014 7:09:00 PM**

Font: (Default) Arial, 8 pt

Page 32: [85] Formatted **Chris Cameron** **2/4/2014 7:09:00 PM**

Font: (Default) Arial, 8 pt

Page 32: [86] Formatted **Chris Cameron** **2/4/2014 7:09:00 PM**

Font: (Default) Arial, 8 pt

Page 32: [87] Formatted **Chris Cameron** **2/4/2014 7:09:00 PM**

Font: (Default) Arial, 8 pt

Page 32: [88] Formatted **Chris Cameron** **2/4/2014 7:09:00 PM**

Font: (Default) Arial, 8 pt

Page 32: [89] Formatted **Chris Cameron** **2/4/2014 7:09:00 PM**

Font: (Default) Arial, 8 pt

Page 32: [90] Formatted **Chris Cameron** **2/4/2014 7:09:00 PM**

Font: (Default) Arial, 8 pt

Page 32: [91] Formatted **Chris Cameron** **2/4/2014 7:09:00 PM**

Font: (Default) Arial, 8 pt

Page 32: [92] Formatted **Chris Cameron** **2/4/2014 7:09:00 PM**

Font: (Default) Arial, 8 pt

Page 32: [93] Formatted **Chris Cameron** **2/4/2014 7:09:00 PM**

Font: (Default) Arial, 8 pt

Page 32: [94] Formatted **Chris Cameron** **2/4/2014 7:09:00 PM**

Font: (Default) Arial, 8 pt

Page 32: [95] Formatted **Chris Cameron** **2/4/2014 7:09:00 PM**

Font: (Default) Arial, 8 pt

Page 32: [96] Formatted **Chris Cameron** **2/4/2014 7:09:00 PM**

Font: (Default) Arial, 8 pt

Page 32: [97] Formatted **Chris Cameron** **2/4/2014 7:09:00 PM**

Font: (Default) Arial, 8 pt

Font: (Default) Arial, 8 pt

Page 32: [99] Formatted **Chris Cameron** **2/4/2014 7:09:00 PM**

Font: (Default) Arial, 8 pt

Page 32: [100] Formatted **Chris Cameron** **2/4/2014 7:09:00 PM**

Font: (Default) Arial, 8 pt

Page 32: [101] Formatted **Chris Cameron** **2/4/2014 7:09:00 PM**

Font: (Default) Arial, 8 pt

Page 32: [102] Formatted **Chris Cameron** **2/4/2014 7:09:00 PM**

Font: (Default) Arial, 8 pt

Page 32: [103] Formatted **Chris Cameron** **2/4/2014 7:09:00 PM**

Font: (Default) Arial, 8 pt

Page 32: [104] Formatted **Chris Cameron** **2/4/2014 7:09:00 PM**

Font: (Default) Arial, 8 pt

Page 32: [105] Formatted **Chris Cameron** **2/4/2014 7:09:00 PM**

Font: (Default) Arial, 8 pt

Page 32: [106] Formatted **Chris Cameron** **2/4/2014 7:09:00 PM**

Font: (Default) Arial, 8 pt

Page 32: [107] Formatted **Chris Cameron** **2/4/2014 7:09:00 PM**

Font: (Default) Arial, 8 pt

Page 32: [108] Formatted **Chris Cameron** **2/4/2014 7:09:00 PM**

Font: (Default) Arial, 8 pt

Page 32: [109] Formatted **Chris Cameron** **2/4/2014 7:09:00 PM**

Font: (Default) Arial, 8 pt

Page 32: [110] Formatted **Chris Cameron** **2/4/2014 7:09:00 PM**

Font: (Default) Arial, 8 pt

Page 32: [111] Formatted **Chris Cameron** **2/4/2014 7:09:00 PM**

Font: (Default) Arial, 8 pt

Page 32: [112] Formatted **Chris Cameron** **2/4/2014 7:09:00 PM**

Font: (Default) Arial, 8 pt

Page 32: [113] Formatted **Chris Cameron** **2/4/2014 7:09:00 PM**

Font: (Default) Arial, 8 pt

Page 32: [114] Formatted **Chris Cameron** **2/4/2014 7:09:00 PM**

Font: (Default) Arial, 8 pt

Page 32: [115] Formatted **Chris Cameron** **2/4/2014 7:09:00 PM**

Font: (Default) Arial, 8 pt

Page 32: [116] Formatted **Chris Cameron** **2/4/2014 7:09:00 PM**

Font: (Default) Arial, 8 pt

Page 32: [117] Formatted **Chris Cameron** **2/4/2014 7:09:00 PM**

For peer review only

For peer review only - http://bmjopen.bmj.com/site/about/guidelines.xhtml

Page 32: [118] Formatted **Chris Cameron** **2/4/2014 7:09:00 PM**
Font: (Default) Arial, 8 pt

Page 32: [119] Formatted **Chris Cameron** **2/4/2014 7:09:00 PM**
Font: (Default) Arial, 8 pt

Page 32: [120] Formatted **Chris Cameron** **2/4/2014 7:09:00 PM**
Font: (Default) Arial, 8 pt

Page 32: [121] Formatted **Chris Cameron** **2/4/2014 7:09:00 PM**
Font: (Default) Arial, 8 pt

Page 32: [122] Formatted **Chris Cameron** **2/4/2014 7:09:00 PM**
Font: (Default) Arial, 8 pt

Page 32: [123] Formatted **Chris Cameron** **2/4/2014 7:09:00 PM**
Font: (Default) Arial, 8 pt

Page 32: [124] Formatted **Chris Cameron** **2/4/2014 7:09:00 PM**
Font: (Default) Arial, 8 pt

Page 32: [125] Formatted **Chris Cameron** **2/4/2014 7:09:00 PM**
Font: (Default) Arial, 8 pt

Page 32: [126] Formatted **Chris Cameron** **2/4/2014 7:09:00 PM**
Font: (Default) Arial, 8 pt

Page 32: [127] Formatted **Chris Cameron** **2/4/2014 7:09:00 PM**
Font: (Default) Arial, 8 pt

Page 32: [128] Formatted **Chris Cameron** **2/4/2014 7:09:00 PM**
Font: (Default) Arial, 8 pt

Page 32: [129] Formatted **Chris Cameron** **2/4/2014 7:09:00 PM**
Font: (Default) Arial, 8 pt

Page 32: [130] Formatted **Chris Cameron** **2/4/2014 7:09:00 PM**
Font: (Default) Arial, 8 pt

Page 32: [131] Formatted **Chris Cameron** **2/4/2014 7:09:00 PM**
Font: (Default) Arial, 8 pt

Page 32: [132] Formatted **Chris Cameron** **2/4/2014 7:09:00 PM**
Font: (Default) Arial, 8 pt

Page 32: [133] Formatted **Chris Cameron** **2/4/2014 7:09:00 PM**
Font: (Default) Arial, 8 pt

Page 32: [134] Formatted **Chris Cameron** **2/4/2014 7:09:00 PM**
Font: (Default) Arial, 8 pt

Page 32: [135] Formatted **Chris Cameron** **2/4/2014 7:09:00 PM**
Font: (Default) Arial, 8 pt

Page 32: [136] Formatted **Chris Cameron** **2/4/2014 7:09:00 PM**
Font: (Default) Arial, 8 pt

For peer review only

Font: (Default) Arial, 8 pt

Page 32: [138] Formatted **Chris Cameron** **2/4/2014 7:09:00 PM**

Font: (Default) Arial, 8 pt

Page 32: [139] Formatted **Chris Cameron** **2/4/2014 7:09:00 PM**

Font: (Default) Arial, 8 pt

Page 32: [140] Formatted **Chris Cameron** **2/4/2014 7:09:00 PM**

Font: (Default) Arial, 8 pt

Page 32: [141] Formatted **Chris Cameron** **2/4/2014 7:09:00 PM**

Font: (Default) Arial, 8 pt

Page 32: [142] Formatted **Chris Cameron** **2/4/2014 7:09:00 PM**

Font: (Default) Arial, 8 pt

Page 32: [143] Formatted **Chris Cameron** **2/4/2014 7:09:00 PM**

Font: (Default) Arial, 8 pt

Page 32: [144] Formatted **Chris Cameron** **2/4/2014 7:09:00 PM**

Font: (Default) Arial, 8 pt

Page 32: [145] Formatted **Chris Cameron** **2/4/2014 7:09:00 PM**

Font: (Default) Arial, 8 pt

Page 32: [146] Formatted **Chris Cameron** **2/4/2014 7:09:00 PM**

Font: (Default) Arial, 8 pt

Page 32: [147] Formatted **Chris Cameron** **2/4/2014 7:09:00 PM**

Font: (Default) Arial, 8 pt

Page 32: [148] Formatted **Chris Cameron** **2/4/2014 7:09:00 PM**

Font: (Default) Arial, 8 pt

Page 32: [149] Formatted **Chris Cameron** **2/4/2014 7:09:00 PM**

Font: (Default) Arial, 8 pt

Page 32: [150] Formatted **Chris Cameron** **2/4/2014 7:09:00 PM**

Font: (Default) Arial, 8 pt

Page 32: [151] Formatted **Chris Cameron** **2/4/2014 7:09:00 PM**

Font: (Default) Arial, 8 pt

Page 32: [152] Formatted **Chris Cameron** **2/4/2014 7:09:00 PM**

Font: (Default) Arial, 8 pt

Page 32: [153] Formatted **Chris Cameron** **2/4/2014 7:09:00 PM**

Font: (Default) Arial, 8 pt

Page 32: [154] Formatted **Chris Cameron** **2/4/2014 7:09:00 PM**

Font: (Default) Arial, 8 pt

Page 32: [155] Formatted **Chris Cameron** **2/4/2014 7:09:00 PM**

Font: (Default) Arial, 8 pt

Page 48: [156] Formatted **Chris Cameron** **2/6/2014 10:07:00 PM**

For peer review only

Page 48: [157] Formatted	Chris Cameron	2/6/2014 10:08:00 PM

Font: 9 pt

Page 48: [157] Formatted	Chris Cameron	2/6/2014 10:08:00 PM

Font: 9 pt

Page 48: [158] Formatted	Chris Cameron	2/6/2014 10:08:00 PM

Font: 9 pt

Page 48: [158] Formatted	Chris Cameron	2/6/2014 10:08:00 PM

Font: 9 pt

Page 48: [159] Formatted	Chris Cameron	2/6/2014 10:08:00 PM

Font: 9 pt

Page 48: [159] Formatted	Chris Cameron	2/6/2014 10:08:00 PM

Font: 9 pt

Page 48: [160] Formatted	Chris Cameron	2/6/2014 10:08:00 PM

Font: 9 pt

Page 48: [160] Formatted	Chris Cameron	2/6/2014 10:08:00 PM

Font: 9 pt

Page 48: [161] Formatted	Chris Cameron	2/6/2014 10:08:00 PM

Font: 9 pt

Page 48: [161] Formatted	Chris Cameron	2/6/2014 10:08:00 PM

Font: 9 pt

Page 48: [162] Formatted	Chris Cameron	2/6/2014 10:08:00 PM

Font: 9 pt

Page 48: [162] Formatted	Chris Cameron	2/6/2014 10:08:00 PM

Font: 9 pt

Page 48: [163] Formatted	Chris Cameron	2/6/2014 10:08:00 PM

Font: 9 pt

Page 48: [163] Formatted	Chris Cameron	2/6/2014 10:08:00 PM

Font: 9 pt

Page 48: [164] Formatted	Chris Cameron	2/6/2014 10:08:00 PM

Font: 9 pt

Page 48: [164] Formatted	Chris Cameron	2/6/2014 10:08:00 PM

Font: 9 pt

Page 48: [165] Formatted	Chris Cameron	2/6/2014 10:08:00 PM

Font: 9 pt

Page 48: [165] Formatted	Chris Cameron	2/6/2014 10:08:00 PM

Font: 9 pt

For peer review only - http://bmjopen.bmj.com/site/about/guidelines.xhtml

Page 48: [166] Formatted	Chris Cameron	2/6/2014 10:08:00 PM

Font: 9 pt

For peer review only

Font: 9 pt

Page 48: [167] Formatted Chris Cameron 2/6/2014 10:08:00 PM

Font: 9 pt

Page 48: [167] Formatted Chris Cameron 2/6/2014 10:08:00 PM

Font: 9 pt

Page 48: [168] Formatted Chris Cameron 2/6/2014 10:08:00 PM

Font: 9 pt

Page 48: [168] Formatted Chris Cameron 2/6/2014 10:08:00 PM

Font: 9 pt

Page 48: [169] Formatted Chris Cameron 2/6/2014 10:08:00 PM

Font: 9 pt

Page 48: [169] Formatted Chris Cameron 2/6/2014 10:08:00 PM

Font: 9 pt

Page 48: [170] Formatted Chris Cameron 2/6/2014 10:08:00 PM

Font: 9 pt

Page 48: [170] Formatted Chris Cameron 2/6/2014 10:08:00 PM

Font: 9 pt

Page 48: [171] Formatted Chris Cameron 2/6/2014 10:08:00 PM

Font: 9 pt

Page 48: [171] Formatted Chris Cameron 2/6/2014 10:08:00 PM

Font: 9 pt

Page 48: [172] Formatted Chris Cameron 2/6/2014 10:08:00 PM

Font: 9 pt

Page 48: [172] Formatted Chris Cameron 2/6/2014 10:08:00 PM

Font: 9 pt

Page 48: [173] Formatted Chris Cameron 2/6/2014 10:08:00 PM

Font: 9 pt

Page 48: [173] Formatted Chris Cameron 2/6/2014 10:08:00 PM

Font: 9 pt

Page 48: [174] Formatted Chris Cameron 2/6/2014 10:08:00 PM

Font: 9 pt

Page 48: [174] Formatted Chris Cameron 2/6/2014 10:08:00 PM

Font: 9 pt

Page 48: [175] Formatted Chris Cameron 2/6/2014 10:08:00 PM

Font: 9 pt

Page 48: [175] Formatted Chris Cameron 2/6/2014 10:08:00 PM

Font: 9 pt

Page 48: [176] Formatted Chris Cameron 2/6/2014 10:08:00 PM

For peer review only

Page 48: [176] Formatted	Chris Cameron	2/6/2014 10:08:00 PM

Font: 9 pt

Page 48: [177] Formatted	Chris Cameron	2/6/2014 10:08:00 PM

Font: 9 pt

Page 48: [177] Formatted	Chris Cameron	2/6/2014 10:08:00 PM

Font: 9 pt

Page 48: [178] Formatted	Chris Cameron	2/6/2014 10:08:00 PM

Font: 9 pt

Page 48: [178] Formatted	Chris Cameron	2/6/2014 10:08:00 PM

Font: 9 pt

Page 48: [179] Formatted	Chris Cameron	2/6/2014 10:08:00 PM

Font: 9 pt

Page 48: [179] Formatted	Chris Cameron	2/6/2014 10:08:00 PM

Font: 9 pt

Page 48: [180] Formatted	Chris Cameron	2/6/2014 10:08:00 PM

Font: 9 pt

Page 48: [180] Formatted	Chris Cameron	2/6/2014 10:08:00 PM

Font: 9 pt

Page 48: [181] Formatted	Chris Cameron	2/6/2014 10:08:00 PM

Font: 9 pt

Page 48: [181] Formatted	Chris Cameron	2/6/2014 10:08:00 PM

Font: 9 pt

Page 48: [182] Formatted	Chris Cameron	2/6/2014 10:08:00 PM

Font: 9 pt

Page 48: [182] Formatted	Chris Cameron	2/6/2014 10:08:00 PM

Font: 9 pt

Page 48: [183] Formatted	Chris Cameron	2/6/2014 10:08:00 PM

Font: 9 pt

Page 48: [183] Formatted	Chris Cameron	2/6/2014 10:08:00 PM

Font: 9 pt

Page 48: [184] Formatted	Chris Cameron	2/6/2014 10:08:00 PM

Font: 9 pt

Page 48: [184] Formatted	Chris Cameron	2/6/2014 10:08:00 PM

Font: 9 pt

Page 48: [185] Formatted	Chris Cameron	2/6/2014 10:08:00 PM

Font: 9 pt

Page 48: [185] Formatted	Chris Cameron	2/6/2014 10:08:00 PM

Font: 9 pt

For peer review only - http://bmjopen.bmj.com/site/about/guidelines.xhtml

Font: 9 pt

Page 48: [186] Formatted Chris Cameron 2/6/2014 10:08:00 PM

Font: 9 pt

Page 48: [187] Formatted Chris Cameron 2/6/2014 10:08:00 PM

Font: 9 pt

Page 48: [187] Formatted Chris Cameron 2/6/2014 10:08:00 PM

Font: 9 pt

Page 48: [188] Formatted Chris Cameron 2/6/2014 10:08:00 PM

Font: 9 pt

Page 48: [188] Formatted Chris Cameron 2/6/2014 10:08:00 PM

Font: 9 pt

Page 48: [189] Formatted Chris Cameron 2/6/2014 10:08:00 PM

Font: 9 pt

Page 48: [189] Formatted Chris Cameron 2/6/2014 10:08:00 PM

Font: 9 pt

Page 48: [190] Formatted Chris Cameron 2/6/2014 10:08:00 PM

Font: 9 pt

Page 48: [190] Formatted Chris Cameron 2/6/2014 10:08:00 PM

Font: 9 pt

Page 48: [191] Formatted Chris Cameron 2/6/2014 10:08:00 PM

Font: 9 pt

Page 48: [191] Formatted Chris Cameron 2/6/2014 10:08:00 PM

Font: 9 pt

Page 48: [192] Formatted Chris Cameron 2/6/2014 10:08:00 PM

Font: 9 pt

Page 48: [192] Formatted Chris Cameron 2/6/2014 10:08:00 PM

Font: 9 pt

Page 48: [193] Formatted Chris Cameron 2/6/2014 10:08:00 PM

Font: 9 pt

Page 48: [193] Formatted Chris Cameron 2/6/2014 10:08:00 PM

Font: 9 pt

Page 48: [194] Formatted Chris Cameron 2/6/2014 10:08:00 PM

Font: 9 pt

Page 48: [194] Formatted Chris Cameron 2/6/2014 10:08:00 PM

Font: 9 pt

Page 48: [195] Formatted Chris Cameron 2/6/2014 10:08:00 PM

Font: 9 pt

Page 48: [195] Formatted Chris Cameron 2/6/2014 10:08:00 PM

For peer review only

Page 48: [196] Formatted	Chris Cameron	2/6/2014 10:08:00 PM

Font: 9 pt

Page 48: [196] Formatted	Chris Cameron	2/6/2014 10:08:00 PM

Font: 9 pt

Page 48: [197] Formatted	Chris Cameron	2/6/2014 10:08:00 PM

Font: 9 pt

Page 48: [197] Formatted	Chris Cameron	2/6/2014 10:08:00 PM

Font: 9 pt

Page 48: [198] Formatted	Chris Cameron	2/6/2014 10:08:00 PM

Font: 9 pt

Page 48: [198] Formatted	Chris Cameron	2/6/2014 10:08:00 PM

Font: 9 pt

Page 48: [199] Formatted	Chris Cameron	2/6/2014 10:08:00 PM

Font: 9 pt

Page 48: [199] Formatted	Chris Cameron	2/6/2014 10:08:00 PM

Font: 9 pt

Page 48: [200] Formatted	Chris Cameron	2/6/2014 10:08:00 PM

Font: 9 pt

Page 48: [200] Formatted	Chris Cameron	2/6/2014 10:08:00 PM

Font: 9 pt

Page 48: [201] Formatted	Chris Cameron	2/6/2014 10:08:00 PM

Font: 9 pt

Page 48: [201] Formatted	Chris Cameron	2/6/2014 10:08:00 PM

Font: 9 pt

Page 48: [202] Formatted	Chris Cameron	2/6/2014 10:08:00 PM

Font: 9 pt

Page 48: [202] Formatted	Chris Cameron	2/6/2014 10:08:00 PM

Font: 9 pt

Page 48: [203] Formatted	Chris Cameron	2/6/2014 10:08:00 PM

Font: 9 pt

Page 48: [203] Formatted	Chris Cameron	2/6/2014 10:08:00 PM

Font: 9 pt

Page 48: [204] Formatted	Chris Cameron	2/6/2014 10:08:00 PM

Font: 9 pt

Page 48: [204] Formatted	Chris Cameron	2/6/2014 10:08:00 PM

Font: 9 pt

For peer review only - http://bmjopen.bmj.com/site/about/guidelines.xhtml

Page 48: [205] Formatted	Chris Cameron	2/6/2014 10:08:00 PM

Font: 9 pt

For peer review only

Font: 9 pt

Page 48: [206] Formatted Chris Cameron 2/6/2014 10:08:00 PM

Font: 9 pt

Page 48: [206] Formatted Chris Cameron 2/6/2014 10:08:00 PM

Font: 9 pt

Page 48: [207] Formatted Chris Cameron 2/6/2014 10:08:00 PM

Font: 9 pt

Page 48: [207] Formatted Chris Cameron 2/6/2014 10:08:00 PM

Font: 9 pt

Page 48: [208] Formatted Chris Cameron 2/6/2014 10:08:00 PM

Font: 9 pt

Page 48: [208] Formatted Chris Cameron 2/6/2014 10:08:00 PM

Font: 9 pt

Page 48: [209] Formatted Chris Cameron 2/6/2014 10:08:00 PM

Font: 9 pt

Page 48: [209] Formatted Chris Cameron 2/6/2014 10:08:00 PM

Font: 9 pt

Page 48: [210] Formatted Chris Cameron 2/6/2014 10:08:00 PM

Font: 9 pt

Page 48: [210] Formatted Chris Cameron 2/6/2014 10:08:00 PM

Font: 9 pt

Page 48: [211] Formatted Chris Cameron 2/6/2014 10:08:00 PM

Font: 9 pt

Page 48: [211] Formatted Chris Cameron 2/6/2014 10:08:00 PM

Font: 9 pt

Page 48: [212] Formatted Chris Cameron 2/6/2014 10:08:00 PM

Font: 9 pt

Page 48: [212] Formatted Chris Cameron 2/6/2014 10:08:00 PM

Font: 9 pt

Page 48: [213] Formatted Chris Cameron 2/6/2014 10:08:00 PM

Font: 9 pt

Page 48: [213] Formatted Chris Cameron 2/6/2014 10:08:00 PM

Font: 9 pt

Page 48: [214] Formatted Chris Cameron 2/6/2014 10:08:00 PM

Font: 9 pt

Page 48: [214] Formatted Chris Cameron 2/6/2014 10:08:00 PM

Font: 9 pt

Page 48: [215] Formatted Chris Cameron 2/6/2014 10:08:00 PM

For peer review only

Page 48: [215] Formatted	Chris Cameron	2/6/2014 10:08:00 PM

Font: 9 pt

Page 48: [216] Formatted	Chris Cameron	2/6/2014 10:08:00 PM

Font: 9 pt

Page 48: [216] Formatted	Chris Cameron	2/6/2014 10:08:00 PM

Font: 9 pt

Page 48: [217] Formatted	Chris Cameron	2/6/2014 10:08:00 PM

Font: 9 pt

Page 48: [217] Formatted	Chris Cameron	2/6/2014 10:08:00 PM

Font: 9 pt

Page 48: [218] Formatted	Chris Cameron	2/6/2014 10:08:00 PM

Font: 9 pt

Page 48: [218] Formatted	Chris Cameron	2/6/2014 10:08:00 PM

Font: 9 pt

Page 48: [219] Formatted	Chris Cameron	2/6/2014 10:08:00 PM

Font: 9 pt

Page 48: [219] Formatted	Chris Cameron	2/6/2014 10:08:00 PM

Font: 9 pt

Page 48: [220] Formatted	Chris Cameron	2/6/2014 10:08:00 PM

Font: 9 pt

Page 48: [220] Formatted	Chris Cameron	2/6/2014 10:08:00 PM

Font: 9 pt

Page 48: [221] Formatted	Chris Cameron	2/6/2014 10:08:00 PM

Font: 9 pt

Page 48: [221] Formatted	Chris Cameron	2/6/2014 10:08:00 PM

Font: 9 pt

Page 48: [222] Formatted	Chris Cameron	2/6/2014 10:08:00 PM

Font: 9 pt

Page 48: [222] Formatted	Chris Cameron	2/6/2014 10:08:00 PM

Font: 9 pt

Page 48: [223] Formatted	Chris Cameron	2/6/2014 10:08:00 PM

Font: 9 pt

Page 48: [223] Formatted	Chris Cameron	2/6/2014 10:08:00 PM

Font: 9 pt

For peer review only

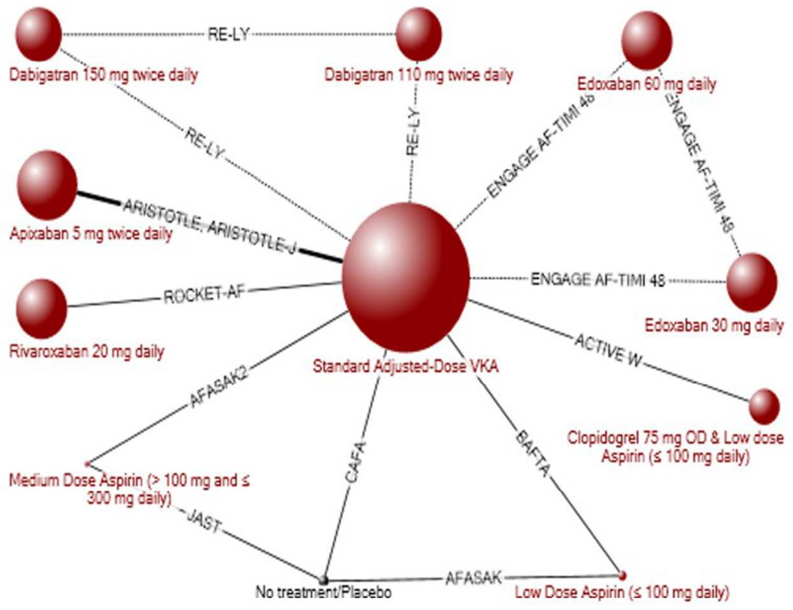

Figure 1
81x60mm (300 x 300 DPI)

For peer review only

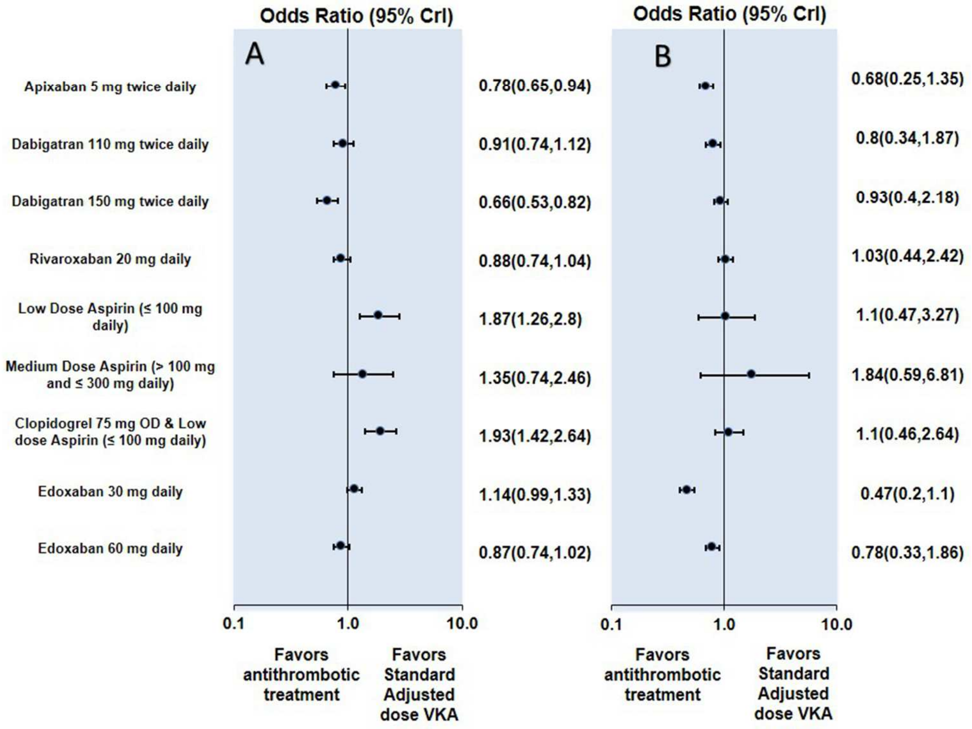

Figure 2
81x60mm (300 x 300 DPI)

Standard Adjusted-Dose VKA	**0.69 (0.6,0.8)**	**0.8 (0.69,0.93)**	0.93 (0.81,1.08)	1.03 (0.89,1.19)	1.05 (0.59,1.86)	1.79 (0.62,5.64)	1.1 (0.83,1.47)	**0.47 (0.4,0.54)**	**0.78 (0.69,0.9)**
**0.78 (0.65,0.94)**	Apixaban 5 mg twice daily	1.16 (0.94,1.43)	**1.35 (1.1,1.65)**	**1.48 (1.21,1.82)**	1.52 (0.84,2.73)	2.59 (0.88,8.2)	**1.59 (1.15,2.19)**	**0.67 (0.54,0.83)**	1.13 (0.93,1.38)
0.91 (0.74,1.12)	1.17 (0.89,1.53)	Dabigatran 110 mg twice daily	1.16 (0.998,1.36)	**1.28 (1.04,1.58)**	1.31 (0.72,2.36)	2.23 (0.76,7.09)	1.38 (0.99,1.9)	**0.58 (0.47,0.72)**	0.98 (0.8,1.2)
**0.66 (0.53,0.82)**	0.84 (0.63,1.12)	**0.72 (0.57,0.9)**	Dabigatran 150 mg twice daily	1.1 (0.9,1.36)	1.13 (0.62,2.03)	1.92 (0.66,6.1)	1.18 (0.86,1.63)	**0.5 (0.4,0.62)**	0.84 (0.69,1.03)
0.88 (0.74,1.04)	1.12 (0.87,1.44)	0.96 (0.74,1.25)	**1.33 (1.01,1.76)**	Rivaroxaban 20 mg daily	1.03 (0.57,1.83)	1.74 (0.6,5.54)	1.07 (0.78,1.48)	**0.45 (0.37,0.56)**	**0.76 (0.63,0.93)**
**1.87 (1.26,2.8)**	**2.39 (1.55,3.72)**	**2.05 (1.31,3.22)**	**2.84 (1.8,4.5)**	**2.14 (1.39,3.3)**	Low Dose Aspirin (≤ 100 mg daily)	1.71 (0.51,6.03)	1.05 (0.55,1.99)	**0.44 (0.25,0.8)**	0.75 (0.42,1.34)
1.35 (0.74,2.46)	1.72 (0.92,3.24)	1.47 (0.78,2.79)	**2.04 (1.08,3.88)**	1.54 (0.83,2.88)	0.72 (0.38,1.35)	Medium Dose Aspirin (> 100 mg and ≤ 300 mg daily)	0.62 (0.19,1.85)	**0.26 (0.08,0.76)**	0.44 (0.14,1.28)
**1.93 (1.42,2.64)**	**2.47 (1.73,3.54)**	**2.11 (1.46,3.07)**	**2.93 (2.01,4.31)**	**2.2 (1.56,3.15)**	1.03 (0.62,1.71)	1.44 (0.73,2.82)	Clopidogrel 75 mg OD & Low dose Aspirin (≤ 100 mg daily)	**0.42 (0.3,0.58)**	**0.71 (0.52,0.98)**
1.14 (0.99,1.33)	**1.46 (1.16,1.85)**	1.25 (0.97,1.62)	**1.74 (1.33,2.28)**	**1.31 (1.04,1.64)**	**0.61 (0.4,0.93)**	0.85 (0.46,1.58)	**0.59 (0.42,0.83)**	Edoxaban 30 mg daily	1.69 (1.44,1.98)
0.87 (0.74,1.02)	1.12 (0.87,1.42)	0.95 (0.73,1.24)	**1.32 (1.01,1.74)**	1 (0.79,1.26)	**0.47 (0.3,0.71)**	0.65 (0.35,1.21)	**0.45 (0.32,0.64)**	**0.76 (0.65,0.89)**	Edoxaban 60 mg daily

Figure 3
81x60mm (300 x 300 DPI)

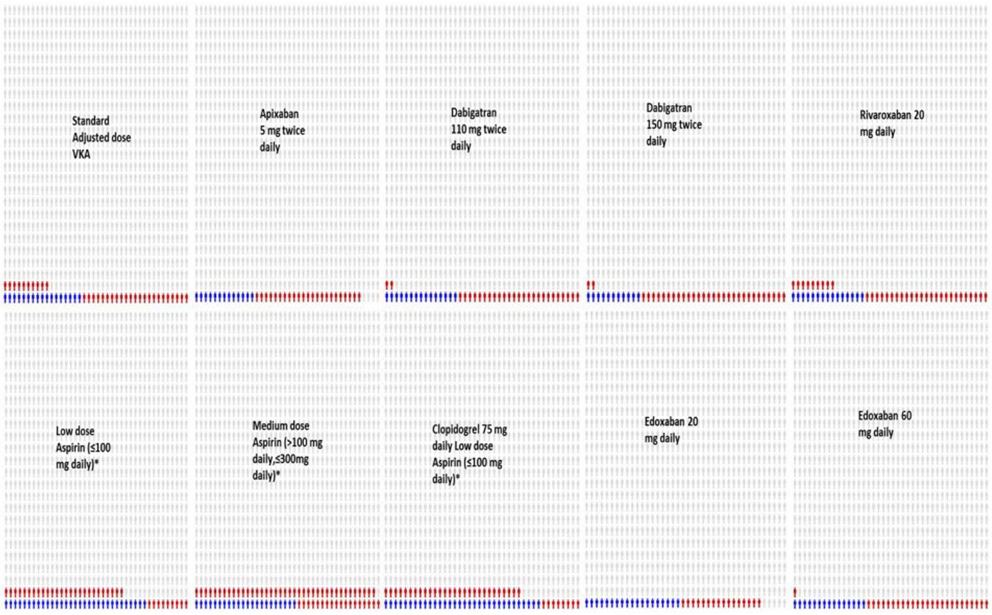

Figure 4
81x60mm (300 x 300 DPI)

SUPPLEMENTARY APPENDIX

Appendix 1: Search Strategy

OVERVIEW

Interface:	Ovid
Databases:	EBM Reviews - Cochrane Central Register of Controlled Trials <May 2012><br>Ovid Embase 1974 to June 6, 2012<br>Ovid MEDLINE 1946 to June 7, 2012<br>Ovid MEDLINE Daily<br>Ovid MEDLINE In-Process & Other Non-Indexed Citations<br>**Note:** Subject headings have been customized for each database. Duplicates between databases were removed in Ovid.
Date of Search:	June 7, 2012; updated January 23, 2014
Alerts:	Monthly search updates began June 11, 2012 and ran until project completion
Study Types:	Randomized Controlled Trials
Limits:	Publication years 1988-present<br>Humans<br>Conference abstracts omitted

SYNTAX GUIDE

/	At the end of a phrase, searches the phrase as a subject heading
.sh	At the end of a phrase, searches the phrase as a subject heading
MeSH	Medical Subject Heading
exp	Explode a subject heading
*	Before a word, indicates that the marked subject heading is a primary topic;<br>or, after a word, a truncation symbol (wildcard) to retrieve plurals or varying endings
#	Truncation symbol for one character
ADJ	Requires words are adjacent to each other (in any order)
ADJ#	Adjacency within # number of words (in any order)
.ti	Title
.ab	Abstract
.hw	Heading Word; usually includes subject headings and controlled vocabulary
.nm.	Name of substance word
.ot	Original title
.pt	Publication type
.rn	CAS registry number
use cctr	Select results from Cochrane database of Clinical Trials
use oemezd	Select results from Embase database
use pmez	Select results from Medline database

Clinical Multi-database Strategy

Line #	Search Strategy
1	(211914-51-1 or 211915-06-9).rn,nm. or (dabigatran* or Pradaxa or Pradax or Prazaxa or Praxada or bibr 1048 or bibr1048 or bibr953 or bibr 953 or rendix).ti,ot,ab,sh,rn,hw,nm.
2	(BMS 562247 or BMS562247 or apixaban* or Eliquis or Eliques).ti,ot,ab,sh,rn,hw,nm. or 503612-47-3.rn,nm.

For peer review only

3	(rivaroxaban* or Xarelto or BAY 59-7939 or BAY59-7939 or BAY597939 or BAY 597939).ti,ot,ab,sh,rn,hw,nm. or 366789-02-8.rn,nm.
4	(90055-48-4 or 113665-84-2).rn,nm. or (clopidogrel* or clopilet or grepid or iscover or plavix or zopya or zylagren or zyllt or duocover or duoplavin).ti,ot,ab,sh,rn,hw,nm.
5	Aspirin/ or 50-78-2.rn,nm. or (acetylsalicylic acid* or ASA* or acenterine or acesal or acetan or acetard or aceticil or aceticyl or acetilum or acetonyl or acetilum or acetonyl or acetophen or acetosal or acetosalicylic acid or acetosalin or acetosalum or acetyl salicylate or acetyl salicylic acid or acetylin or acetylo or acetylon or acetylosalicylic acid or acetylsal or acetysal or acetylsalicyclic acid or acetylsalicyl or acetylsalicylate or acetylsalycic acid or acetylsalycylic acid or oracidulatum or actorin or Acylpyrin or acylpyrine or acytosal or adiro or alabunkun or alasil or albyl-e or alka seltzer or alkASA or Aloxiprimum or anacin or anasprin or anopyrin or ansin or anthrom or aptor or arthralgyl or asaflow or ascription or orasaphen or asapor or asatard or asawin or aspec or aspent or aspergum or aspex or aspilets or aspirem or aspirgran or ASAa or ASAe or ASAine or aspirisucre or aspisol or aspo cid or aspro or asrina or asrivo or asta or asteric or astrix or bebesan or biprin or bokey or boxazin or breoprin or bufferin or cafenol or caprin or cASA or catalgine or catalgix or cemerit or cemirit or claradin or claragine or Colfarit or comoprin or contrheuma or daga or darosal or Dispril or dispirin or dolean or dusil or Easprin or ecasil or Ecotrin or egalgic or emocin or empirin or encaprin or encine em or Endosprin or entaprin or entercin or enteroprin or enterosarine or enterospirine or entrophen or eskotrin or euthermine or extren or genasprin or globentyl or godamed or gotosan or helicon or idotyl or infatabs a or istopirin or istopyrine or ivepirine or juvepirine or keypo or kilios or kinderASA or Magnecyl or measurin or mejoral or melabon or Micristin or micropyrin or mikristin or miniasal or mycrisitin or naspro or novasen or nu seal or nu seals or ortho acetoxybenzoate or ortho acetoxybenzoic acid or ortho acetyloxybenzoate or ortho acetyloxybenzoic acid or ostoprin or pancemol or paracin or paynocil or pengo or plewin or Polopirin or polopiryna or premaspin or primaspan or proprin or pyronoval or reumyl or rhodine or rhonal or ronal or salacetin or salisalido or sargepirine or soldral or solpyron or Solprin or solucetyl or solupsa or Solupsan or spren or super tru or tapal or temagin or tevapirin or th 2152 or thrombo-aspilets or treupahlin or treuphalin or tromalyt or tromcor or turiital or verin or vitalink or xaxa or Zorprin).ti,ot,ab,sh,rn,hw,nm.
6	Warfarin/ or 81-81-2.rn,nm. or (adoisine or aldocumar or athrombin* or befarin or brumolin or carfin or circuvit or compound 42 or coumadan or coumadin or coumadine or coumafen or coumafene or coumefene or coumaphene or coumarins or dagonal or farin or jantoven or kumatox or maforan or marevan or orfarin or panwarfarin or panwarfin or prothromadin or simarc 2 or sofarin or tedicumar or tintorane or waran or warfant or warfar or warfarin or warfarine or warfil or warfilone or warnerin).ti,ot,ab,sh,rn,hw,nm.
7	Acenocoumarol/ or 152-72-7.rn,nm. or (acenocoumarol* or nicoumalone or acenocoumarin or acenocoumarine or acenocoumarole or acenocoumarolum or acenocumarol or acenocumarolo or acenocumerol or acenkumarin or acitrom or ascumar or neo sintrom or neosintrom or neositron or nicoumalone or nicumalon or nitrovarfarian or nitrowarfarin or sincoumar or sincumar or sinkumar or sinthrom or sinthrome or sintrom or sintroma or sintron or syncoumar or syncumar or synthrom or syntrom or syntroma or sintron or syncoumar or syncumar or syntrom or trombostop or zotil or sinthrome or synthrom or syncoumar or syncumar or sinkumar or sintrom or minisintrom or trombostop or zotil).ti,ot,ab,sh,rn,hw,nm.
8	or/1-7
9	Atrial fibrillation/
10	((atrial or atrium or auricular or heart) adj1 fibrillation*).ti,ab.
11	(a-fib or afib).ti,ab.
12	9 or 10 or 11
13	8 and 12
14	13 use pmez
15	13 use cctr
16	14 or 15
17	*dabigatran/ or (dabigatran* or Pradaxa or Pradax or Prazaxa or praxada or bibr 1048 or bibr1048 or bibr953 or bibr 953 or rendix).ti,ab.
18	*apixaban/ or (BMS 562247 or BMS562247 or apixaban* or Eliquis or Eliques).ti,ab.
19	*rivaroxaban/ or (rivaroxaban* or Xarelto or BAY 59-7939 or BAY59-7939 or BAY597939 or BAY 597939).ti,ab.
20	*clopidogrel/ or *acetylsalicylic acid plus clopidogrel/ or (clopidogrel* or clopilet or grepid or iscover or plavix or zopya or zylagren or zyllt or duocover or duoplavin).ti,ab.
21	*warfarin/ or (adoisine or aldocumar or athrombin* or befarin or brumolin or carfin or circuvit or

For peer review only

	compound 42 or coumadan or coumadin or coumadine or coumafen or coumafene or coumefene or coumaphene or coumarins or dagonal or farin or jantoven or kumatox or maforan or marevan or orfarin or panwarfarin or panwarfin or prothromadin or simarc 2 or sofarin or tedicumar or tintorane or waran or warfant or warfar or warfarin* or warfarine or warfil or warfilone or warnerin).ti,ab.
22	*acetylsalicylic acid/ or (acetylsalicylic acid* or ASA* or acenterine or acesal or acetan or acetard or aceticil or aceticyl or acetilum or acetonyl or acetilum or acetonyl or acetophen or acetosal or acetosalicylic acid or acetosalin or acetosalum or acetyl salicylate or acetyl salicylic acid or acetylin or acetylo or acetylon or acetylosalicylic acid or acetylsal or acetysal or acetylsalicyclic acid or acetylsalicyl or acetylsalicylate or acetylsalycic acid or acetylsalycylic acid or oracidulatum or actorin or Acylpyrin or acylpyrine or acytosal or adiro or alabunkun or alasil or albyl-e or alka seltzer or alkASA or Aloxiprimum or anasprin or anopyrin or ansin or anthrom or aptor or arthralgyl or asaflow or orasaphen or asapor or asatard or asawin or aspec or aspent or aspergum or aspex or aspilets or aspirem or aspirgran or ASAa or ASAe or ASAine or aspirisucre or aspisol or aspo cid or aspro or asrina or asrivo or asta or asteric or astrix or bebesan or biprin or bokey or boxazin or breoprin or bufferin or cafenol or caprin or cASA or catalgine or catalgix or cemerit or cemirit or claradin or claragine or Colfarit or comoprin or contrheuma or daga or darosal or Dispril or dispirin or dolean or dusil or Easprin or ecasil or Ecotrin or egalgic or emocin or empirin or encaprin or encine em or Endosprin or entaprin or entercin or enteroprin or enterosarine or enterospirine or entrophen or eskotrin or euthermine or extren or genasprin or globentyl or godamed or gotosan or helicon or idotyl or infatabs a or istopirin or istopyrine or ivepirine or juvepirine or keypo or kilios or kinderASA or Magnecyl or measurin or mejoral or melabon or Micristin or micropyrin or mikristin or miniasal or mycrisitin or naspro or novasen or nu seal or nu seals or ortho acetoxybenzoate or ortho acetoxybenzoic acid or ortho acetyloxybenzoate or ortho acetyloxybenzoic acid or ostoprin or pancemol or paracin or paynocil or pengo or plewin or Polopirin or polopiryna or premaspin or primaspan or proprin or pyronoval or reumyl or rhodine or rhonal or ronal or salacetin or salisalido or sargepirine or soldral or solpyron or Solprin or solucetyl or solupsa or Solupsan or spren or super tru or tapal or temagin or tevapirin or th 2152 or thrombo-aspilets or treupahlin or treuphalin or tromalyt or tromcor or turiital or verin or vitalink or xaxa or Zorprin).ti,ab.
23	*Acenocoumarol/ or (acenocoumarol* or nicoumalone or acenocoumarin or acenocoumarine or acenocoumarole or acenocoumarolum or acenocumarol or acenocumarolo or acenocumerol or acenkumarin or acitrom or ascumar or neo sintrom or neosintrom or neositron or nicoumalone or nicumalon or nitrovarfarian or nitrowarfarin or sincoumar or sincumar or sinkumar or sinthrom or sinthrome or sintrom or sintroma or sintron or syncoumar or syncumar or synthrom or syntrom or syntroma or sintron or syncoumar or syncumar or syntrom or trombostop or zotil or sinthrome or synthrom or syncoumar or syncumar or sinkumar or sintrom or minisintrom or trombostop or zotil).ti,ab.
24	or/17-23
25	Heart atrium fibrillation/
26	((atrial or atrium or auricular or heart) adj1 fibrillation*).ti,ab.
27	(a-fib or afib).ti,ab.
28	25 or 26 or 27
29	24 and 28
30	29 use oemezd
31	30 not conference abstract.pt.
32	16 or 31
33	32
34	limit 33 to english language [Limit not valid in CCTR; records were retained]
35	(Randomized Controlled Trial or Controlled Clinical Trial).pt.
36	Randomized Controlled Trial/
37	Randomized Controlled Trials as Topic/
38	"Randomized Controlled Trial (topic)"/
39	Controlled Clinical Trial/
40	Controlled Clinical Trials as Topic/
41	"Controlled Clinical Trial (topic)"/
42	Randomization/
43	Random Allocation/
44	Double-Blind Method/

For peer review only

45	Double Blind Procedure/
46	Double-Blind Studies/
47	Single-Blind Method/
48	Single Blind Procedure/
49	Single-Blind Studies/
50	Placebos/
51	Placebo/
52	Control Groups/
53	Control Group/
54	(random* or sham or placebo*).ti,ab,hw.
55	((singl* or doubl*) adj (blind* or dumm* or mask*)).ti,ab,hw.
56	((tripl* or trebl*) adj (blind* or dumm* or mask*)).ti,ab,hw.
57	(control* adj3 (study or studies or trial*)).ti,ab.
58	(Nonrandom* or non random* or non-random* or quasi-random* or quasirandom*).ti,ab,hw.
59	allocated.ti,ab,hw.
60	((open label or open-label) adj5 (study or studies or trial*)).ti,ab,hw.
61	or/35-60
62	34 and 61
63	limit 62 to yr = "1988 -Current"
64	remove duplicates from 63

OTHER DATABASES	
PubMed	Same MeSH, keywords, limits, and study types used as per MEDLINE search, with appropriate syntax used.

Grey Literature

**Dates for Search:**	**June 12, 2012 – July 8, 2012; updated January 2014**
**Keywords:**	dabigatran, Pradax, Pradaxa, apixaban, Eliquis, rivaroxaban, Xarelto, clopidogrel, Plavix, acetylsalicylic acid, Aspirin, warfarin, Coumadin, acenocoumarol, Sintrom, Sinthrome<br>Atrial fibrillation
**Limits:**	Publication years 1988-present, English

Relevant items from the following sections of the CADTH grey literature checklist, "Grey matters: a practical tool for evidence-based searching" (http://www.cadth.ca/resources/grey-matters) were searched:

- Health Technology Assessment Agencies
- Health Economic
- Clinical Practice Guidelines
- Databases (free)

Internet Search

Appendix 2: PRISMA Diagram

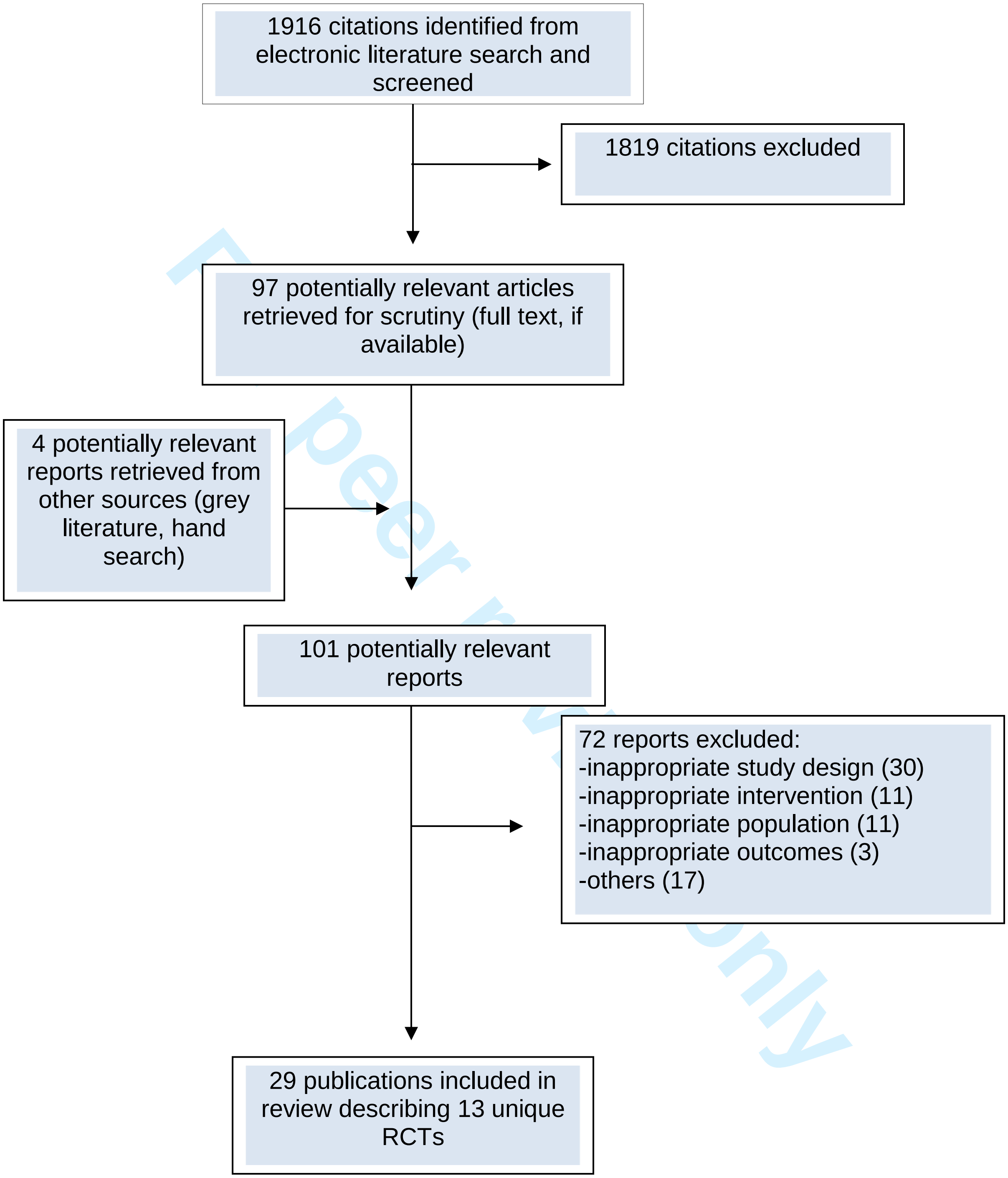

For peer review only

Appendix 3: Summary of Patient Baseline Characteristics

Study	Baseline Characteristics															Centre above TTR Throughout follow-up		
	Age, Mean ± SD (y)	Age Category (%)		Sex (%)		$CHADS_2$, Mean ± SD	$CHADS_2$ Category (%)			Prior Stroke /TIA (%)	DM (%)	Heart Failure (%)	HBP (%)	MI (%)	VKA Naïve (%)	Mean TTR (%)	TTR Category (%)	
		≤75y	>75 y	M	F		0	1	≥2								≤66%	>66%
ACTIVE-W n=6706	70 ± NR	NR	NR	66	34	2 ± 1.1	NR	NR	NR	15	21	30	82	17	23	64	48	52
AFASAK n=672	75 ± NR	NR	NR	54	46	NR ± NR	NR	NR	NR	6	9	53	32	7	NR	N/A	N/A	N/A
AFASAK-2 n=339	73 ± NR	NR	NR	61	39	NR ± NR	NR	NR	NR	8	12	70	45	7	NR	NR	NR	NR
ARISTOTLE n=18201	70 ± NR	69	31	65	35	2.1 ± 1.1	0	34	66	19	25	35	87	14	43	62	NR	NR
ARISTOTLE-J n=222	70 ± NR	69	31	83	17	1.9 ± NR	1	42	57	28	23	1	83	NR	16	60	NR	NR
BAFTA n=973	82 ± 4	0	100	55	45	NR ± NR	NR	NR	NR	13	13	20	54	11	NR	67	NR	NR
CAFA n=378	68 ± NR	NR	NR	75	25	NR ± NR	NR	NR	NR	4	12	22	39	13	NR	44	NR	NR
JAST n=871	65 ± NR	NR	NR	70	30	NR ± NR	NR	NR	NR	3	14	9	39	NR	92	N/A	N/A	N/A
PETRO n=236	70 ± NR	70	30	82	18	NR ± NR	NR	NR	NR	18	25	32	71	NR	0	57	NR	NR
RE-LY n=18113	71 ± NR	60	40	64	36	2.1 ± NR	32		68	20	20	23	32	79	17	64	50	50
ROCKET-AF n=14264	73 ± NR	NR	NR	60	40	3.5 ± NR	0	0	100	55	40	62	91	17	38	55	NR	NR
WASPO n=75	83 ± NR	0	100	47	53	NR ± NR	NR	NR	NR	NR	4	NR	47	NR	NR	69	NR	NR
ENGAGE AF-TIMI 48 n=21105	72 ± NR	60	40	62	38	2.8 ± 1.0	0	0	100	28	36	57	94	12	41	65	52	48

DM = diabetes mellitus; F = female sex; HBP = high blood pressure; M = male sex; MI = myocardial infarction; N/A = not applicable; NR = not reported; SD = standard deviation; TIA = transient ischemic attack; TTR = time in therapeutic range; VKA = vitamin K antagonist; y = year.

Note: Only data from the included relevant treatment arms have been extracted.

Appendix 4: Summary of Included Trials

Study and Design	Disposition	Population	Interventions	Follow-up	Primary Outcome(s)
**ACTIVE-W**<br>OL RCT, 2006.<br>Multicenter, 32 countries (including Europe / America).	Randomized: n=6706<br>Completed: n=6026 (90%)	AF patients eligible / willing to take anticoagulant therapy, with ≥ 1 pre-specified risk factor for stroke.	**Clopidogrel / Low-dose ASA** (n=3335)<br>**Warfarin – INR 2.0 to 3.0** (n=3371)	1.3 years (stopped early)	First occurrence of stroke, non-CNS SE, MI or vascular death.
**AFASAK**<br>OL/DB RCT, 1989.<br>Multicenter, Copenhagen.	Randomized: n=1007<br>Completed: n=785 (80%)	AF patients, with no recent anticoagulation or cerebrovascular event, valve replacement or rheumatic heart disease.	***OL Warfarin – INR 2.8 to 4.2 **** *(n=335)*<br>**Low-dose ASA** (n=336)<br>**PL** (n=336)	2 years	Thromboembolic complication: stroke, TIA or embolic complication to viscera / extremities.
**AFASAK-2**<br>OL RCT, 1998.<br>Multicenter, Copenhagen.	Randomized: n=677<br>Completed: n=507 (75%)	AF patients ≥ 60 years (unless with CV or pulmonary disease), without recent TIA or risk factors for bleeding.	***Fixed-dose Warfarin **** *(n=167)*<br>***Fixed-dose Warfarin / Medium-dose ASA **** *(n=171)*<br>**Medium Dose ASA** (n=169)<br>**Warfarin – INR 2.0 to 3.0** (n=170)	3.5 years	Stroke or SE.
**ARISTOTLE**<br>DB RCT, 2011.<br>Multicenter, 39 countries (including Europe / America).	Randomized: n=18201<br>Completed: n=13468 (74%)	AF patients with ≥ 1 risk factor for stroke, without recent stroke or ASA > 165 mg.	**Apixaban 5 mg BID** (n=9120)<br>**Warfarin – INR 2.0 to 3.0** (n=9081)<br>Concomitant use of **ASA** (≤ 165 mg daily) or **NSAIDs** permitted.	NR	Stroke or SE;<br>Major bleeding.
**ARISTOTLE-J**<br>OL/DB RCT, 2011.<br>Multicenter, Japan.	Randomized: n=222<br>Completed: n=197 (89%)	AF patients with ≥ 1 risk factor for stroke, without recent TIA or ASA > 100 mg.	**Apixaban 2.5 mg BID** (n=74)<br>**Apixaban 5 mg BID** (n=74)<br>**OL Warfarin – INR 2.0 to 3.0** (n=74)<br>Concomitant use of **ASA** (≤ 100 mg daily) permitted.	12 weeks	Composite of major bleeding and CRNM bleeding events.
**BAFTA**<br>OL RCT, 2007.<br>Multicenter, UK.	Randomized: n=973<br>Completed: n=694 (72%)	AF patients ≥ 75 years, with clinical uncertainty around optimal treatment.	**Low-dose ASA** (n=485)<br>**Warfarin – INR 2.0 to 3.0** (n=488)	2.7 years	Disabling stroke, ICH and other clinically significant arterial embolism.
**CAFA**<br>DB RCT, 1991.<br>Multicenter, Canada.	Randomized: n=378<br>Completed: n=286 (76%)	AF patients without recent stroke, TIA or MI, and who did not require antiplatelet drug therapy.	**Warfarin – INR 2.0 to 3.0** (n=187)<br>**PL** (n=191)	1.2 years (stopped early)	Ischemic stroke, SE, ICH or fatal hemorrhage.
**JAST**<br>OL RCT, 2006.<br>Multicenter, Japan.	Randomized: n=871<br>Completed: n=686 (79%)	AF patients without recent symptomatic thromboembolic disease, ICH or GI bleeding.	**Medium-dose ASA** (n=426)<br>**No treatment** (n=445)	2.1 years (stopped early)	CV death, symptomatic brain infarction, or TIA.

Study and Design	Disposition	Population	Interventions	Follow-up	Primary Outcome(s)
**PETRO**<br>OL/DB RCT, 2007.<br>Multicenter, 4 countries (Europe and USA).	Randomized: n=502<br>Completed: n=464 (92%)	AF patients with ≥ 1 risk factor for stroke and with coronary artery disease (removed halfway to facilitate recruitment). No recent MI, stroke, TIA or major bleed. Warfarin pre-treatment required for ≥ 8 weeks.	***Dabigatran 50 mg BID ± Low-dose or High-dose ASA **** *(n=105)*<br>***Dabigatran 300 mg BID ± Low-dose or High-dose ASA **** *(n=161)*<br>**Dabigatran 150 mg BID ± Low-dose or High-dose ASA** † (n=166)<br>**OL Warfarin – INR 2.0 to 3.0** (n=70)	12 weeks	Thromboembolic events and bleeding events.
**RE-LY**<br>OL/DB RCT, 2009.<br>Multicenter, 44 countries (including Europe / America).	Randomized: n=18113<br>Completed: n=14580 (80%)	AF patients with ≥ 1 risk factor for stroke, without recent stroke or increased risk of bleeding.	**Dabigatran 110 mg BID** (n=6015)<br>**Dabigatran 150 mg BID** (n=6076)<br>**OL Warfarin – INR 2.0 to 3.0** (n=6022)<br>Concomitant use of **ASA** (<100 mg daily) or other **antiplatelet agents** permitted.	2 years	Stroke or SE and major haemorrhage.
**ROCKET-AF**<br>DB RCT, 2011.<br>Multicenter, 45 countries (including Europe / America).	Randomized: n=14264<br>Completed: n=10957 (77%)	AF patients at moderate to high risk of stroke (CHADS2 score ≥ 2).	**Rivaroxaban 20 mg OD** (n=7131)<br>**Warfarin – INR 2.0 to 3.0** (n=7133)<br>Over a third of patients in both arms took concomitant ASA.	1.9 years	Composite of stroke and SE;<br>Composite of major and NMCR bleeding.
**WASPO**<br>OL RCT, 2007.<br>Multicenter, UK.	Randomized: n=75<br>Completed: n=64 (85%)	Ambulant AF patients > 80 and < 90 years with Folstein score > 25, without previous fall, ICH or GI bleeding. Patients with prior stroke or TIA were excluded.	**Warfarin – INR 2.0 to 3.0** (n=36)<br>**Medium-dose ASA** (n=39)	1 year	Combined endpoints: death; stroke, TIA or SE; serious bleeding; withdrawal.
**ENGAGE AF-TIMI 48**<br>DB RCT, 2013.<br>Multicenter, 46 countries (including Europe / America).	Randomized: n=21105<br>Completed: n=18635 (88%)	AF patients at moderate to high risk of stroke (CHADS2 score ≥ 2).	**Edoxaban 30 mg OD** (n=7034)<br>**Edoxaban 60 mg OD** (n=7035)<br>**Warfarin – INR 2.0 to 3.0** (n=7036)<br>Almost a third of patients in treatment arms took concomitant ASA.	2.8 years	Stroke or SE and major haemorrhage.

AF = atrial fibrillation; **ASA** = acetylsalicylic acid; **CNS** = central nervous system; **CRNM** = clinically relevant non-major bleeding; **CV** = cardiovascular; **DB** = double-blind; **GI** = gastrointestinal; **ICH** = intracerebral hemorrhage; **MI** = myocardial infarction; **NR** = not reported; **NSAIDs** = non-steroidal anti-inflammatory drugs; **OL** = open label; **PL** = placebo; **RCT** = randomized controlled trial; **SE** = systemic embolism; **TIA** = transient ischemic attack; **UK** = United-Kingdom; **VKA** = vitamin K antagonist.

* No data was extracted from these treatment arms since they did not meet the inclusion criteria specified in the protocol. These trials were nevertheless included in the systematic review because they had at least one relevant comparison between two interventions of interest.

† Data was extracted only for dabigatran without ASA (n=100).

Appendix 5: Evidence network for major bleeding. The width of the lines is proportional to the number of randomized controlled trials (RCTs) comparing each pair of treatments, and the size of each treatment node is proportional to the number of randomised participants (sample size). A dotted line indicates a three-arm RCT and a black node indicates a node included in the analysis but not reported in the main text.

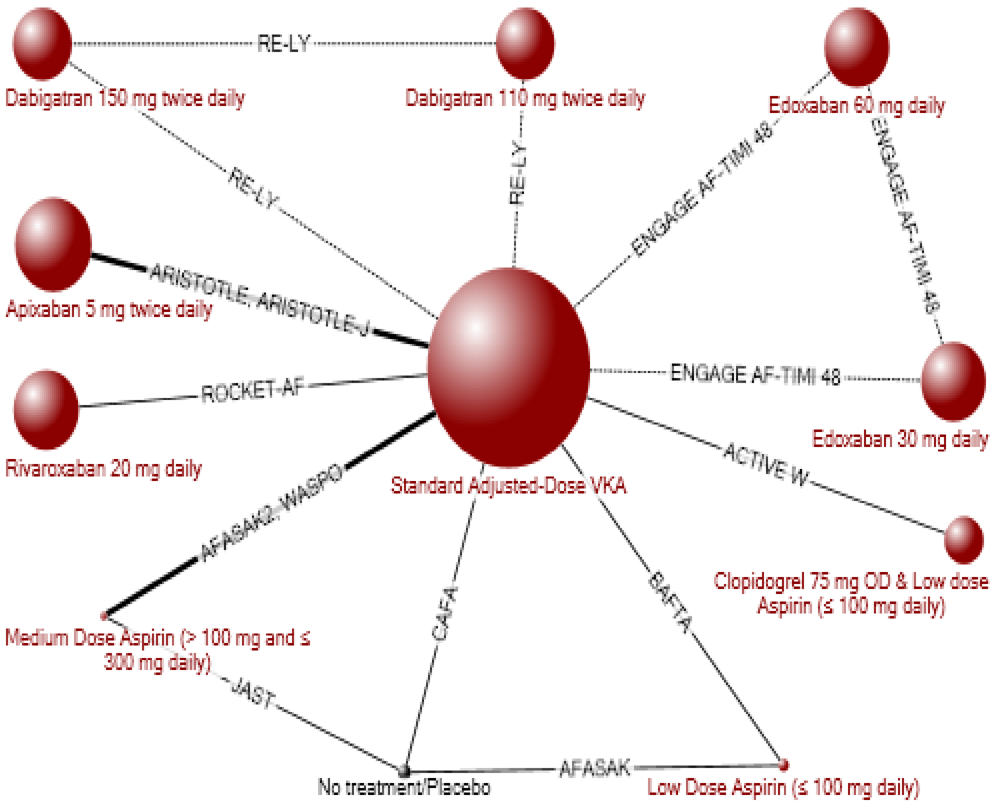

mg= milligram; RCT = randomized controlled trial

Appendix 6: Summary of results from Network Meta-analysis for All-cause Stroke or Systemic Embolism

Treatment	Pair-wise Meta-Analysis, OR (95% CI) versus standard adjusted dose VKA	NMA, Odds Ratio (95% CrI) versus standard adjusted dose VKA	NMA, Absolute Risk Difference per 1,000 treated (95% CrI) versus standard adjusted dose VKA	Probability Best (%) for Outcome	Rank for Outcome	SUCRA
Standard Adjusted-Dose VKA	NA	NA	NA	0.0%	6.0	50.4%
Apixaban 5 mg twice daily	0.79(0.66,0.94)	0.78(0.65,0.94)	4 fewer (6 fewer-1 fewer)	11.1%	2.4	85.5%
Dabigatran 110 mg twice daily	0.91(0.75,1.11)	0.91(0.74,1.12)	2 fewer (5 fewer-2 more)	0.1%	4.5	64.9%
Dabigatran 150 mg twice daily	0.66(0.53,0.82)	0.66(0.53,0.82)	6 fewer (8 fewer-3 fewer)	85.3%	1.2	98.1%
Rivaroxaban 20 mg daily	0.88(0.74,1.05)	0.88(0.74,1.04)	2 fewer (5 fewer-1 more)	1.1%	3.9	71.4%
Low Dose Aspirin (≤ 100 mg daily)	2.03(1.32,3.12)	1.87(1.26,2.8)	14 more (5 more-29 more)	0.0%	10.1	9.4%
Medium Dose Aspirin (> 100 mg and ≤ 300 mg daily)	1.21(0.51,2.87)	1.35(0.74,2.46)	6 more (5 fewer-24 more)	1.2%	7.8	32.2%
Clopidogrel 75 mg OD & Low dose Aspirin (≤ 100 mg daily)	1.93(1.41,2.64)	1.93(1.42,2.64)	15 more (7 more-26 more)	0.0%	10.2	8.3%
Edoxaban 30 mg daily	1.14(0.97,1.33)	1.14(0.99,1.33)	3 more (1 fewer-6 more)	0.0%	7.4	36.2%
Edoxaban 60 mg daily	0.87(0.74,1.02)	0.87(0.74,1.02)	3 fewer (5 fewer-1 more)	1.1%	3.8	72.1%
No treatment/Placebo	1.84(0.66,5.1)	1.53(0.9,2.61)	9 more (2 fewer-26 more)	0.1%	8.8	21.6%

CI = confidence interval; CrI = credible interval; OR= odds ratio

For peer review only

Appendix 7: Summary of results from Network Meta-analysis for Major Bleeding

Treatment	Pair-wise Meta-Analysis, OR (95% CI) versus standard adjusted dose VKA	NMA, Odds Ratio (95% CrI) versus standard adjusted dose VKA	NMA, Absolute Risk Difference per 1,000 treated (95% CrI) versus standard adjusted dose VKA	Probability Best (%) for Outcome	Rank for Outcome	SUCRA
Standard Adjusted-Dose VKA	NA	NA	NA	0.0%	8.0	30.2%
Apixaban 5 mg twice daily	0.69(0.60,0.80)	0.69(0.6,0.8)	10 fewer (13 fewer-7 fewer)	0.0%	3.2	78.0%
Dabigatran 110 mg twice daily	0.8(0.68,0.94)	0.8(0.69,0.93)	7 fewer (10 fewer-3 fewer)	0.0%	4.7	62.6%
Dabigatran 150 mg twice daily	0.93(0.81,1.07)	0.93(0.81,1.08)	3 fewer (7 fewer-3 more)	0.0%	6.8	41.6%
Rivaroxaban 20 mg daily	1.03(0.9,1.18)	1.03(0.89,1.19)	1 more (4 fewer-6 more)	0.0%	8.4	26.2%
Low Dose Aspirin (≤ 100 mg daily)	1.01(0.57,1.78)	1.05(0.59,1.86)	2 more (14 fewer-26 more)	0.1%	7.9	30.7%
Medium Dose Aspirin (> 100 mg and ≤ 300 mg daily)	1.68(0.50,5.69)	1.79(0.62,5.64)	24 more (13 fewer-119 more)	0.0%	10.0	9.9%
Clopidogrel 75 mg OD & Low dose Aspirin (≤ 100 mg daily)	1.1(0.82,1.48)	1.1(0.83,1.47)	4 more (6 fewer-15 more)	0.0%	8.9	20.7%
Edoxaban 30 mg daily	0.47(0.4,0.55)	0.47(0.4,0.54)	18 fewer (20 fewer-15 fewer)	29.3%	1.7	92.8%
Edoxaban 60 mg daily	0.78(0.68,0.89)	0.78(0.69,0.9)	7 fewer (10 fewer-4 fewer)	0.0%	4.5	64.7%
No treatment/Placebo	0.39(0.08,2.02)	0.33(0.08,1.08)	22 fewer (31 fewer-3 more)	70.6%	1.7	92.6%

CI = confidence interval; CrI = credible interval; OR= odds ratio

For peer review only

Appendix 8: Benefit-Harm plot for each treatment (absolute harm difference per 1,000 patients treated). The absolute risk difference of recurrence of Stroke or systemic embolism relative to standard adjusted dose VKA is presented on the x-axis, while the absolute risk difference of major bleeding relative to standard adjusted dose VKA is on the y-axis. The dot is representative of the median estimate for each treatment while the 95% credible intervals are presented as error bars.

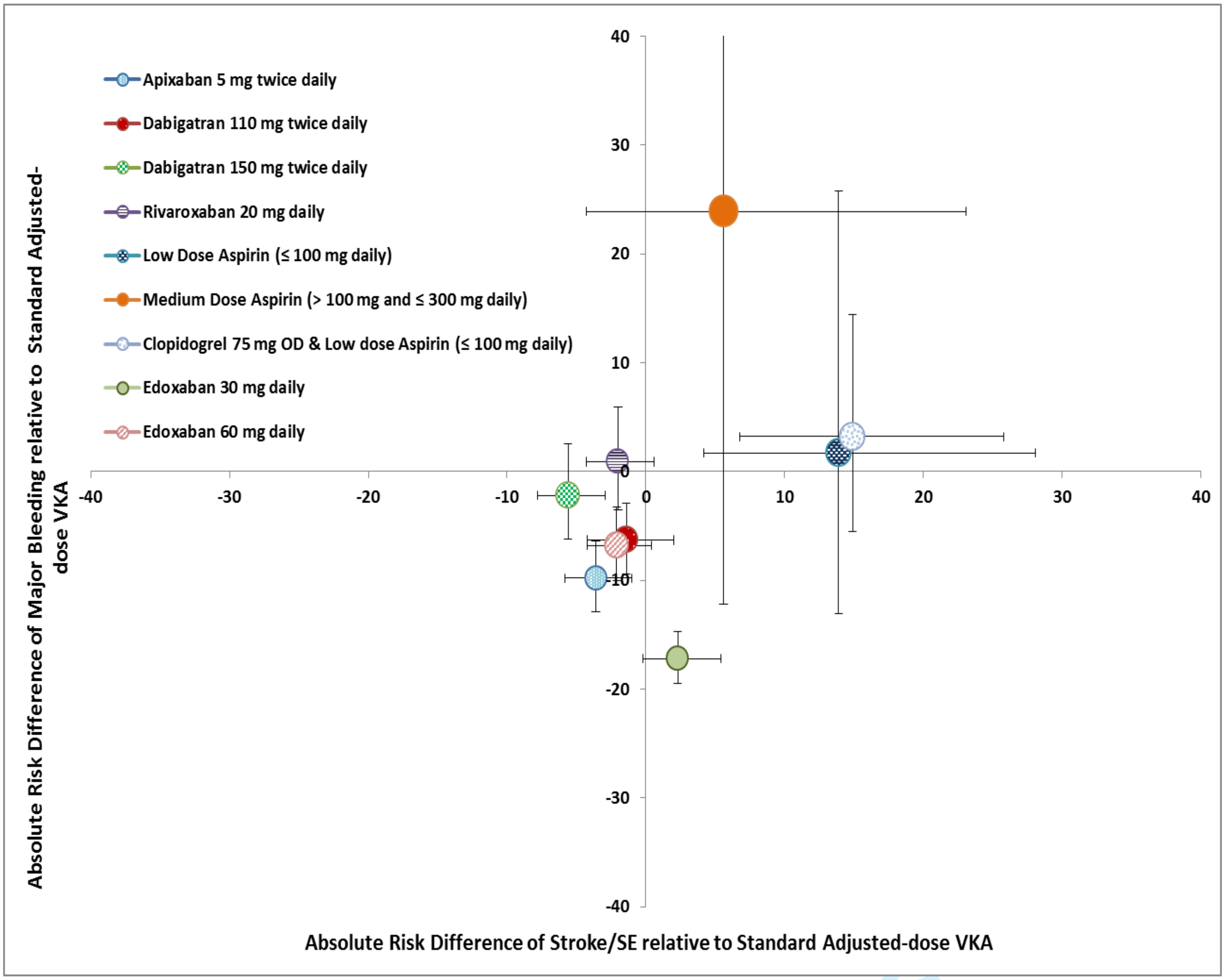

Appendix 9: Rationale of choice of fixed-effects model and limitations

It is challenging to conduct network meta-analyses when evidence structures are comprised of single study connections, especially when there is obvious heterogeneity in the network. The use of a random-effects model with a vague prior on the between study variance exerts a large degree of influence on the credible intervals (CrI's) because there is insufficient studies to reign in the prior and provide an accurate estimate of the between study variance. Accordingly, we also considered informative priors on the between study variance from the recently published reports.[9,39] However, even when these priors were applied on the between study variance, they still did not reign in CrI's effect estimates again because the network was comprised of single study connections. The use of the random effects model with either a vague or informative prior was problematic because treatments that were originally associated with statistically significant reductions in stroke or SE relative to standard adjusted dose VKA were no longer associated with significant reductions, limiting external validity of the model (see Figures below on direct estimates in Online Appendix 4 and 5). While we could have opted to use less vague priors (e.g., ¼ of prior in Turner et al[9]), the choice of the prior would have seemed somewhat arbitrary because it was not based on any empirical evidence. As such, we opted to use the fixed-effects model despite the known issues in doing so. By using the fixed-effects model, treatments that were associated with significant reductions in the risk of stroke or SE relative to standard adjusted dose VKA retained significance. We felt that it would have been inappropriate to remove statistical significance by applying vague priors given the large investment involved in developing clinical trials. However, a consequence of retaining statistical significance is seen when comparing newer oral anticoagulants versus one another in Figure 5. Because we assumed the new oral anticoagulants were all associated with reductions in Stroke or SE relative to standard adjusted dose VKA (if that was the case in the underlying trials), significant reductions in the risk of stroke or SE among new oral anticoagulants are observed in Figure 5. This may or may not be the case in clinical practice. As noted in tables 1 and Online Appendix 2, there is considerable heterogeneity across studies. However, the network structure itself (i.e., single study connections), made it difficult to properly adjust for differences in patient and study characteristics using standard approaches. Accordingly, results in Figure 5 should be interpreted with caution. We conducted simple sub-group analyses (breaking randomization) to help adjust for this heterogeneity, which is a very crude way to adjust for heterogeneity. To fully elucidate the comparative benefits and harms of antithrombotic agents, especially across the various sub-populations, rigorously conducted comparative studies, or network meta-regression analyses of patient-level data are required.

For peer review only

Forest Plot – Odds ratio (95% Credible interval) when using vague prior on the sd (i.e, sd~unif(0,2))

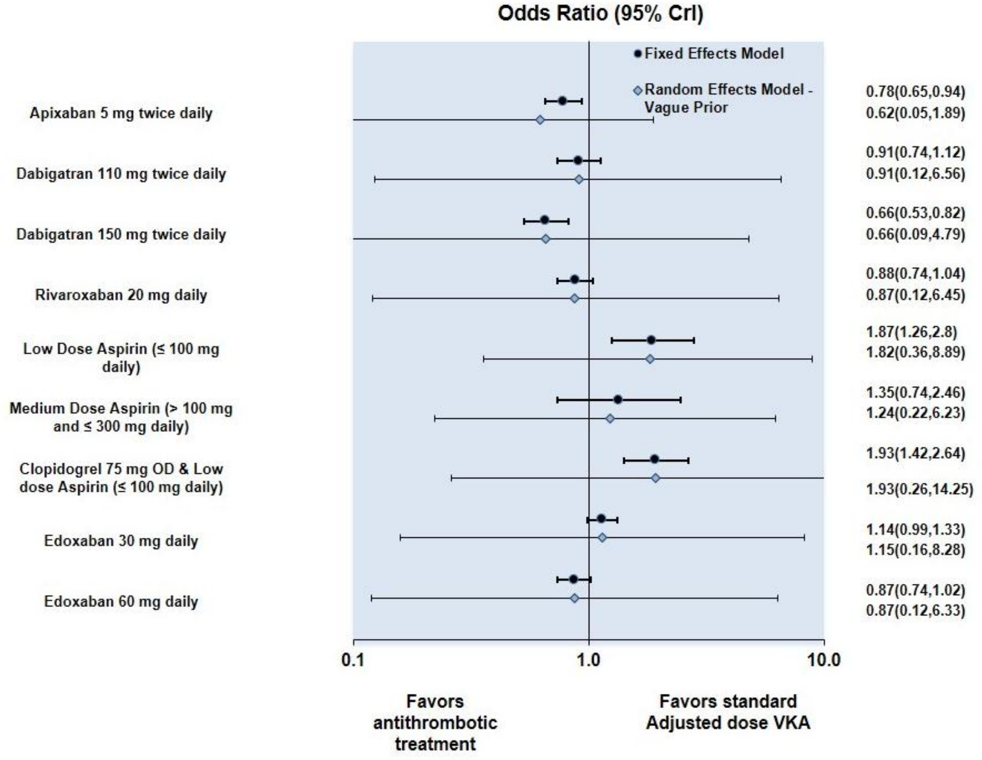

Forest Plot – Odds ratio (95% Credible interval) when using informative prior on the sd (i.e, dlnorm(-3.02,0.29)[9]

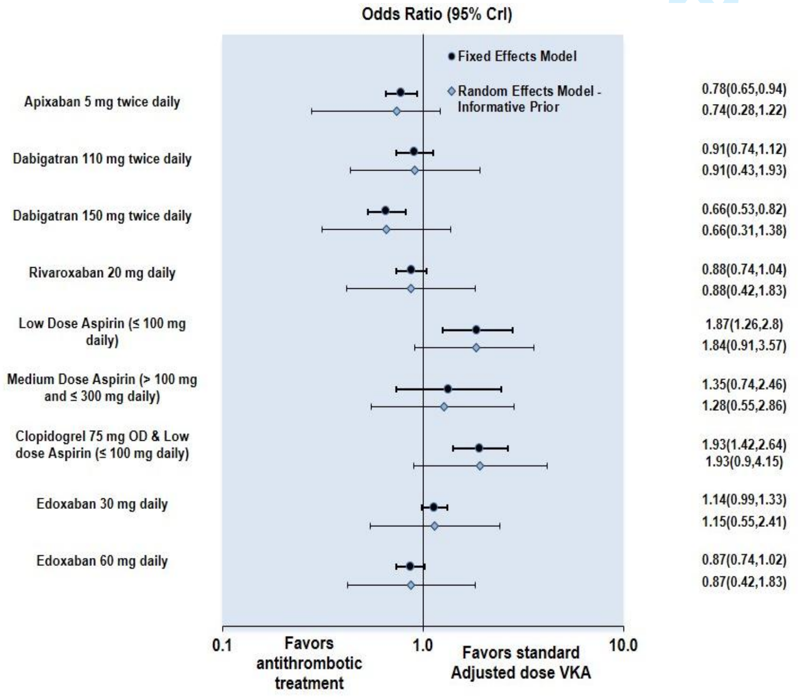

For peer review only - http://bmjopen.bmj.com/site/about/guidelines.xhtml

Appendix 10: PRISMA Checklist

Section/topic	#	Checklist item	Reported on page #
**TITLE**			
Title	1	Identify the report as a systematic review, meta-analysis, or both.	0
**ABSTRACT**			
Structured summary	2	Provide a structured summary including, as applicable: background; objectives; data sources; study eligibility criteria, participants, and interventions; study appraisal and synthesis methods; results; limitations; conclusions and implications of key findings; systematic review registration number.	0
**INTRODUCTION**			
Rationale	3	Describe the rationale for the review in the context of what is already known.	1, Appendix 13
Objectives	4	Provide an explicit statement of questions being addressed with reference to participants, interventions, comparisons, outcomes, and study design (PICOS).	1
**METHODS**			
Protocol and registration	5	Indicate if a review protocol exists, if and where it can be accessed (e.g., Web address), and, if available, provide registration information including registration number.	2
Eligibility criteria	6	Specify study characteristics (e.g., PICOS, length of follow-up) and report characteristics (e.g., years considered, language, publication status) used as criteria for eligibility, giving rationale.	2-3
Information sources	7	Describe all information sources (e.g., databases with dates of coverage, contact with study authors to identify additional studies) in the search and date last searched.	2-3
Search	8	Present full electronic search strategy for at least one database, including any limits used, such that it could be repeated.	2-3
Study selection	9	State the process for selecting studies (i.e., screening, eligibility, included in systematic review, and, if applicable, included in the meta-analysis).	3
Data collection process	10	Describe method of data extraction from reports (e.g., piloted forms, independently, in duplicate) and any processes for obtaining and confirming data from investigators.	3
Data items	11	List and define all variables for which data were sought (e.g., PICOS, funding sources) and any assumptions and simplifications made.	3-4
Risk of bias in individual studies	12	Describe methods used for assessing risk of bias of individual studies (including specification of whether this was done at the study or outcome level), and how this information is to be used in any data synthesis.	3-6
Summary measures	13	State the principal summary measures (e.g., risk ratio, difference in means).	4
Synthesis of results	14	Describe the methods of handling data and combining results of studies, if done, including measures of consistency (e.g., I^2) for each meta-analysis.	4-5
Risk of bias across studies	15	Specify any assessment of risk of bias that may affect the cumulative evidence (e.g., publication bias, selective reporting within studies).	7
Additional analyses	16	Describe methods of additional analyses (e.g., sensitivity or subgroup analyses, meta-regression), if done, indicating which were pre-specified.	6-7
**RESULTS**			

Study selection	17	Give numbers of studies screened, assessed for eligibility, and included in the review, with reasons for exclusions at each stage, ideally with a flow diagram.	5, Appendix 2
Study characteristics	18	For each study, present characteristics for which data were extracted (e.g., study size, PICOS, follow-up period) and provide the citations.	5-6, Appendix 3-4
Risk of bias within studies	19	Present data on risk of bias of each study and, if available, any outcome level assessment (see item 12).	7
Results of individual studies	20	For all outcomes considered (benefits or harms), present, for each study: (a) simple summary data for each intervention group (b) effect estimates and confidence intervals, ideally with a forest plot.	Figure 2-4, Appendix 6-7
Synthesis of results	21	Present results of each meta-analysis done, including confidence intervals and measures of consistency.	Figure 2-4, Appendix 6-7
Risk of bias across studies	22	Present results of any assessment of risk of bias across studies (see Item 15).	7
Additional analysis	23	Give results of additional analyses, if done (e.g., sensitivity or subgroup analyses, meta-regression [see Item 16]).	Table 1, Appendix 9, 12
**DISCUSSION**			
Summary of evidence	24	Summarize the main findings including the strength of evidence for each main outcome; consider their relevance to key groups (e.g., healthcare providers, users, and policy makers).	11-16
Limitations	25	Discuss limitations at study and outcome level (e.g., risk of bias), and at review-level (e.g., incomplete retrieval of identified research, reporting bias).	14-15
Conclusions	26	Provide a general interpretation of the results in the context of other evidence, and implications for future research.	10-16
**FUNDING**			
Funding	27	Describe sources of funding for the systematic review and other support (e.g., supply of data); role of funders for the systematic review.	0

For peer review only

Appendix 11: Assessment of Inconsistency

We plotted the posterior mean deviance of the individual data points in the inconsistency model against their posterior mean deviance in the consistency model to help identify loops where inconsistency is present. In our analysis, the posterior mean deviance contributions are very similar and close to 1, for both models. The consistency model has a similar posterior mean of the residual deviance (26.81 vs. 26.72) and DIC (183.59 vs. 185.36) and hence is a similar fit for both models. A few points show a lower value of the posterior mean deviance in the inconsistency model but these values are not too far from 1. Similarly, a few points have higher values of the posterior mean deviance in the consistency model (again with values not too far from 1) suggesting that each model may fit the data points better for certain points. The parameter estimates are also similar for both models and there is considerable overlap in the 95% credible intervals (see table below), suggesting no evidence of inconsistency in the network.

Plot of posterior mean deviance of the individual data points in the inconsistency model against their posterior mean deviance in the consistency model

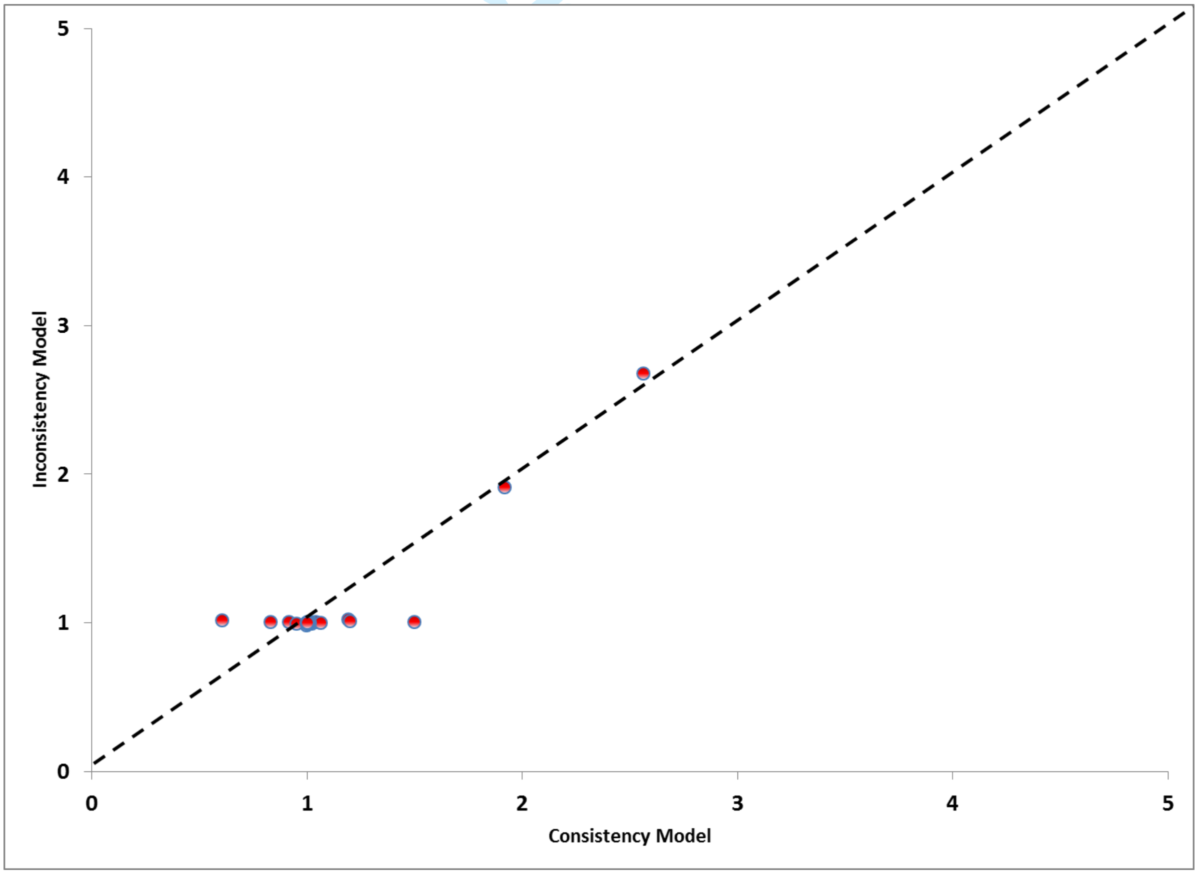

Comparison of effect estimates from consistency and inconsistency models where there is data available for comparison

Comparison	Consistency Model	Inconsistency Model
Apixaban 5 mg twice daily vs Standard Adjusted-Dose VKA	0.78 (0.65,0.94)	0.78 (0.65,0.94)
Dabigatran 110 mg twice daily vs Apixaban 5 mg twice daily vs	0.91 (0.74,1.12)	0.91 (0.74,1.12)
Dabigatran 150 mg twice daily vs Standard Adjusted-Dose VKA	0.66 (0.53,0.82)	0.66 (0.53,0.82)
Rivaroxaban 20 mg daily vs Standard Adjusted-Dose VKA	0.88 (0.74,1.04)	0.88 (0.74,1.04)
Low Dose Aspirin (≤ 100 mg daily) vs Standard Adjusted-Dose VKA	1.87 (1.26,2.8)	2.04 (1.33,3.19)
Medium Dose Aspirin (> 100 mg and ≤ 300 mg daily) vs Standard Adjusted-Dose VKA	1.35 (0.74,2.46)	0.82 (0.33,1.97)
Clopidogrel 75 mg OD & Low dose Aspirin (≤ 100 mg daily) vs Standard Adjusted-Dose VKA	1.93 (1.42,2.64)	1.93 (1.42,2.64)
Edoxaban 30 mg daily vs Standard Adjusted-Dose VKA	1.14 (0.99,1.33)	1.14(0.98,1.33)
Edoxaban 60 mg daily vs Standard Adjusted-Dose VKA	0.87 (0.74,1.02)	0.87 (0.74,1.02)
No treatment/Placebo vs Standard Adjusted-Dose VKA	1.53 (0.9,2.61)	1.89 (0.7,5.71)
No treatment/Placebo vs Low Dose Aspirin (≤ 100 mg daily)	0.82 (0.48,1.38)	1 (0.51,1.98)
Residual deviance	26.81	26.72
DIC	183.59	185.36

Appendix 12: Sensitivity analysis with AVERROES and ACTIVE A

A. Stroke or SE

Evidence Networks for Stroke or SE NMA including AVEROES and ACTIVE A studies

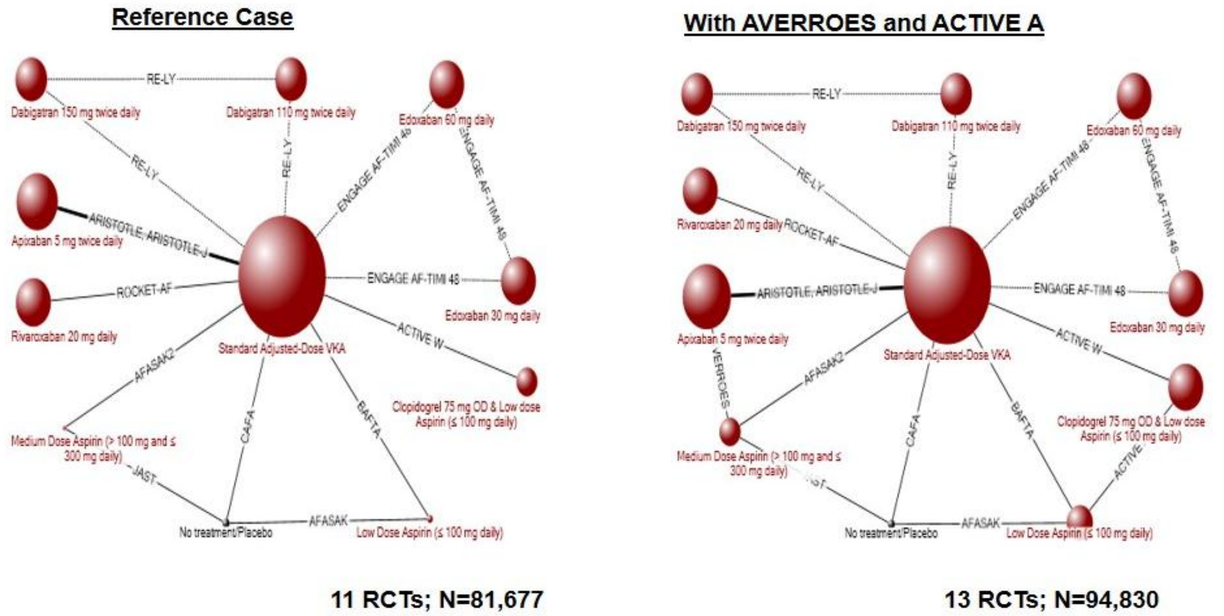

Forest Plot for Stroke or SE NMA including AVERROES and ACTIVE A studies

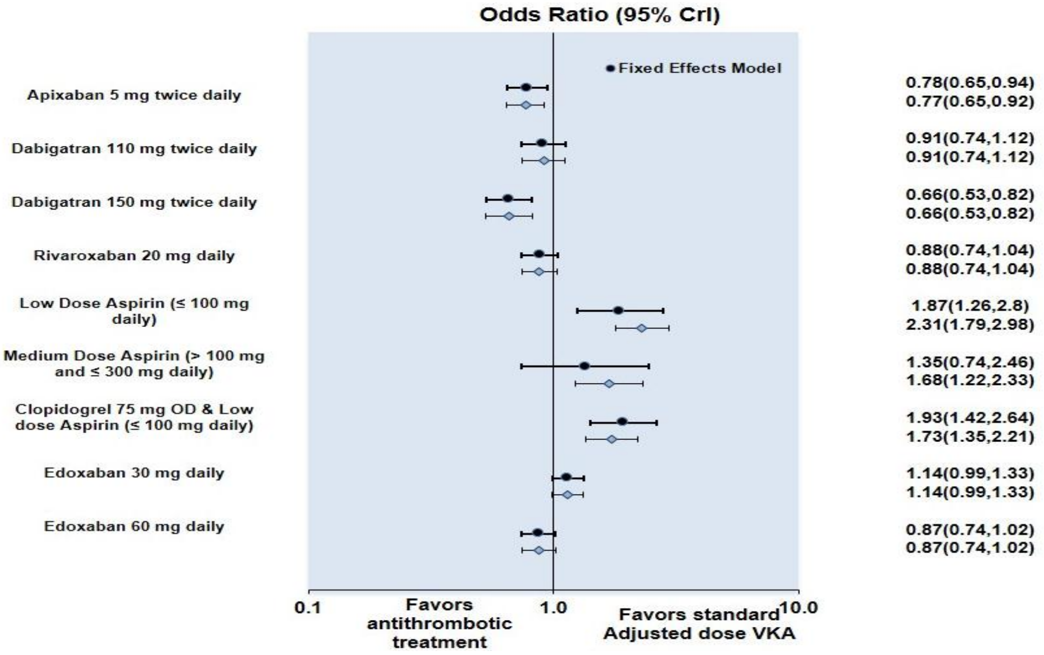

B. Major Bleeding

Evidence Networks for Major Bleeding NMA including AVERROES and ACTIVE A studies

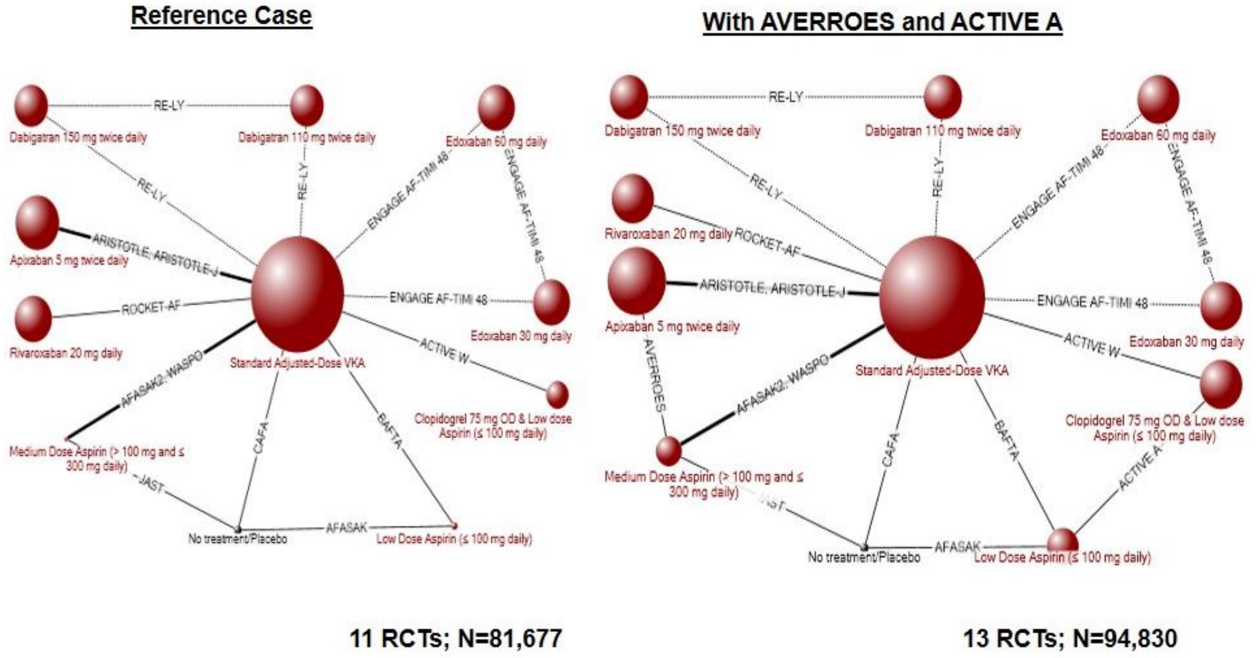

Forest Plot for Major Bleeding NMA including AVERROES and ACTIVE A studies

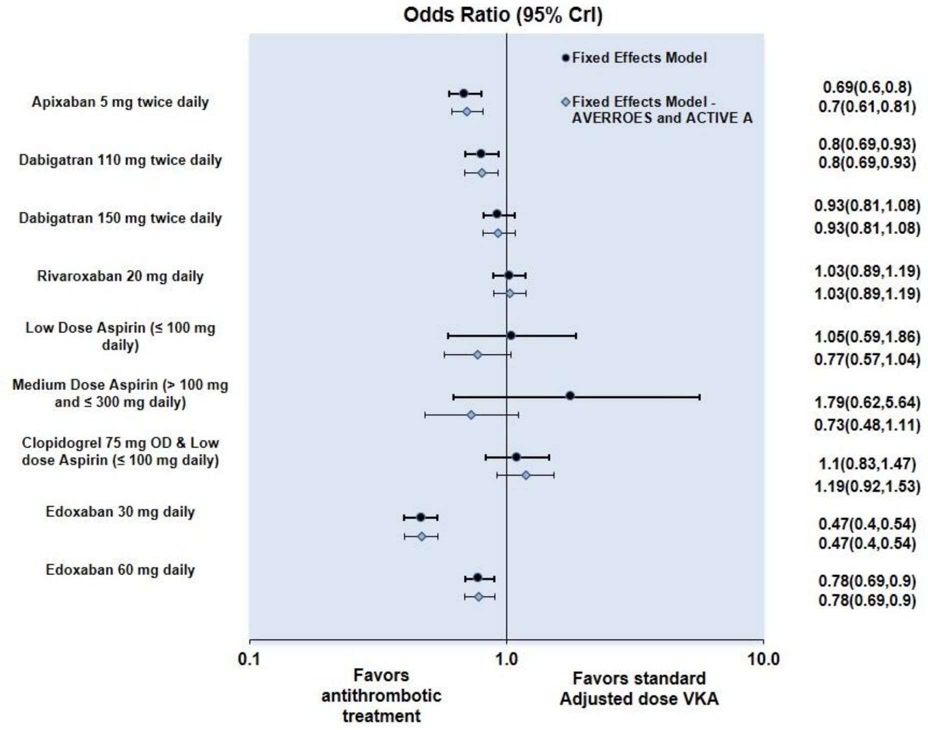

Appendix 13: Comparison with previously published network meta-analyses since Harenberg et al[25]

Author (Publication Year)	Method	Search Date	Included Treatments	Registered Protocol	Relative Measure(s)	Absolute Measure(s) reported	Subgroup Analyses Reported
**Cameron 2014**	Bayesian Network Meta-analysis	January 2014	dabigatran, rivaroxaban, apixaban, edoxaban, and vitamin K antagonists (VKA) at a standard adjusted dose (target international normalised ratio 2.0-3.0), acetylsalicylic acid, acetylsalicylic acid and clopidogrel	Yes (PROSPERO registry - CRD42012002721).	OR (95% CrI)	Yes	Yes
**Ruff 2013**	Traditional Meta-analysis	November 2013	dabigatran, rivaroxaban, apixaban, edoxaban	Not Reported	RR (95%CI)	No	Yes
**Dogliotti 2013**	Bayesian Network Meta-analysis	May 2012	dabigatran, rivaroxaban, apixaban, vitamin K antagonists (VKA) at a standard adjusted dose, acetylsalicylic acid, acetylsalicylic acid and clopidogrel	Yes (metacardio.org)	OR (95% CrI)	No	No
**Assiri 2013**	Baysian Network Meta-analysis	August 2012	dabigatran, rivaroxaban, apixaban, vitamin K antagonists (VKA) at a standard adjusted dose, acetylsalicylic acid, acetylsalicylic acid and clopidogrel	Not Reported	RR (95%CrI)	No	No
**Bruins 2013**	Traditional Meta-analysis	June 2013	antistasin, apixaban, betrixaban, darexaban, DU176b, edoxaban, eribaxaban, fondaparinux, idraparinux, otamixaban, razaxaban, rivaroxaban, yagin, YM150, LY517717, SSR126517E, vitamin K antagonists (warfarin and congeners) with the intensity of anticoagulation dose-adjusted using the International Normalised Ratio (INR)	Yes (Cochrane Library)	OR (95%CI) RR (95%CI) in summary table	Yes	Yes

Appendix 14: Excluded Study List

Inappropriate Study Design

Cardiovasc J S Afr. 2006 Mar;17(2):91.
J Fam Pract. 2006;55(9):753.
Cardiovasc J Afr. 2009 May;20(3):210.
Cardiovasc J Afr. 2009 Sep;20(5):311-2.
Australian Journal of Pharmacy. 2009;90(1073):68.
Formulary. 2010;45(1):16.
Aalbers J. Cardiovasc J Afr. 2010 Nov;21(6):342-3.
Alexander W, et al. PT. 2010;35(10):580-1.
Belavic JM. Nurse Pract. 2011 Sep;36(9):6-7.
Camm AJ. Eur Heart J. 2009 Nov;30(21):2554-5.
Chua D, et al. N Engl J Med. 2009;361(13):1312-3.
Connolly SJ. Eur J Heart Fail. 2009;11(12):1215.
DeSilvey DL. Am J Geriatr Cardiol. 2006 Sep;15(5):326-7.
Dunn A. Ann Intern Med. 2011;154(8):JC4-3.
Eikelboom JW, et al. J Thromb Haemost. 2012;10(5):966-8.
Ezekowitz MD, et al. J Interv Card Electrophysiol. 2011 Dec;32(3):173-80.
Garcia D, et al. Lancet. 2007 Aug 11;370(9586):460-1.
Granger CB. Ann Intern Med. 2012;156(JC1-2):JC1-3.
Hart RG, et al. Ann Intern Med. 2007;147(8):590-2.
Katona A, et al. Eur Heart J. 2006 Jun;27(11):1382.
Kita K, et al. Interventional Cardiology. 2011;3(6):637.
Lopes RD, et al. Evid Based Med. 2011;16(6):187-8.
Nolan J, et al. Br Heart J. 1992 Dec;68(6):544-7.
Paikin JS, et al. Expert Rev Cardiovasc Ther. 2011 Mar;9(3):279-86.
Panichpisal K, et al. Future Neurology. 2011;6(2):155-8.
Roy B, et al. Am J Cardiol. 2012 Feb 1;109(3):370-7.
Sloan MA. Evid Based Med. 2006;11(6):170.
Vassiliou VS. N Engl J Med. 2012;366(1):88-90.
Veloso HH, et al. Chest. 2005 Jul;128(1):475.
Weinberger J. Curr Cardiol Rep. 2010;12(1):1-2.

Inappropriate Intervention

Lancet. 1994 Mar 19;343(8899):687-91.
The Stroke Prevention in Atrial Fibrillation Investigators. Arch Intern Med. 1996 Feb 26;156(4):409-16.
Archer SL, et al. Am Heart J. 1995 Aug;130(2):287-95.
Edvardsson N, et al. J Intern Med. 2003 Jul;254(1):95-101.
Hart RG, et al. Cerebrovasc Dis. 2000 Jan;10(1):39-43.
Hellemons BS, et al. Control Clin Trials. 1999 Aug;20(4):386-93.
Hori M, et al. Circ J. 2012 Jun 5.
Miller VT, et al. Neurology. 1996 Jan;46(1):238-40.
Perez-Gomez F, et al. Eur Heart J. 2007;28(8):996-1003.

For peer review only

Singer DE, et al. Am Heart J. 1992 Dec;124(6):1567-73.
Connolly S, et al. Eur Heart J. 2013 May:34(20):1498-505

Inappropriate Population

ACTIVE Investigators, et al. N Engl J Med 2009;360(20):2066-78.
Circulation 1991;84(2):527-39.
Connolly SJ, et al. N Engl J Med 2011;364(9):806-17.
Dewilde W, et al. Am Heart J. 2009 Nov;158(5):713-8.
Diener HC, et al. Lancet Neurol. 2012 Mar;11(3):225-31.
EAFT (European Atrial Fibrillation Trial) Study Group. Lancet. 1993;342(8882):1255-62.
Eikelboom JW, et al. J Stroke Cerebrovasc Dis. 2012 Aug;21(6):429-35.
ESPRIT Study Group, et al. Lancet Neurol. 2007 Feb;6(2):115-24.
Hart RG, et al. Cerebrovasc Dis. 2008;25(4):344-7.
N Engl J Med 1990;322(12):863-8.
Wang TH, et al. Eur Heart J. 2007 Sep;28(18):2200-7.

Inappropriate Outcomes

De Caterina R, et al. Eur Heart J. 2010 Sep;31(17):2133-40.
Ezekowitz MD, et al. Circulation. 1995 Oct 15;92(8):2178-82.
Lorenzoni R, et al. Am Heart J. 2004 Jul;148(1):e6.

Others

Cardiovasc J Afr. 2011 May;22(3):164.
Active Steering Committee, et al. Am Heart J. 2006 Jun;151(6):1187-93.
ROCKET AF Study Investigators. Am Heart J. 2010 Mar;159(3):340-7.
The Stroke Prevention in Atrial Fibrillation Investigators. Stroke. 1990 Apr;21(4):538-45.
Boysen G, et al. Stroke. 1990;21(Suppl I):I-92.
De Schryver EL. Cerebrovasc Dis. 2000 Mar;10(2):147-50.
Desai A. Clinical Trials Registry - India (CTRI). 2006.
Eikelboom JW, et al. Am Heart J. 2010 Mar;159(3):348-53.
Ezekowitz MD, et al. Am Heart J. 2009 May;157(5):805-10, 810.e1-2.
Flaker G, et al. J Am Coll Cardiol. 2012 Feb 28;59(9):854-5.
Lopes RD, et al. Lancet. 2012 Oct 2. Epub ahead of print.
Lopes RD, et al. Am Heart J. 2010 Mar;159(3):331-9.
Mant J, et al. Cerebrovasc Dis. 2007;23(Suppl 2):10.
Mant J, et al. Cardiol Rev. 2008;25(7):32-6.
Mant JW, et al. BMC Cardiovasc Disord. 2003 Aug 26;3:9.
Posada IS, et al. Am Heart J. 1999 Jul;138(1 Pt 1):137-43.
Van Latum JC. Ned Tijdschr Geneeskd. 1994;138(20):1025-31.
Yigit Z. Turk Kardiyoloji Dernegi Arsivi. 2000;28(1):8-19+4.

For peer review only

Appendix 15: Completion rates

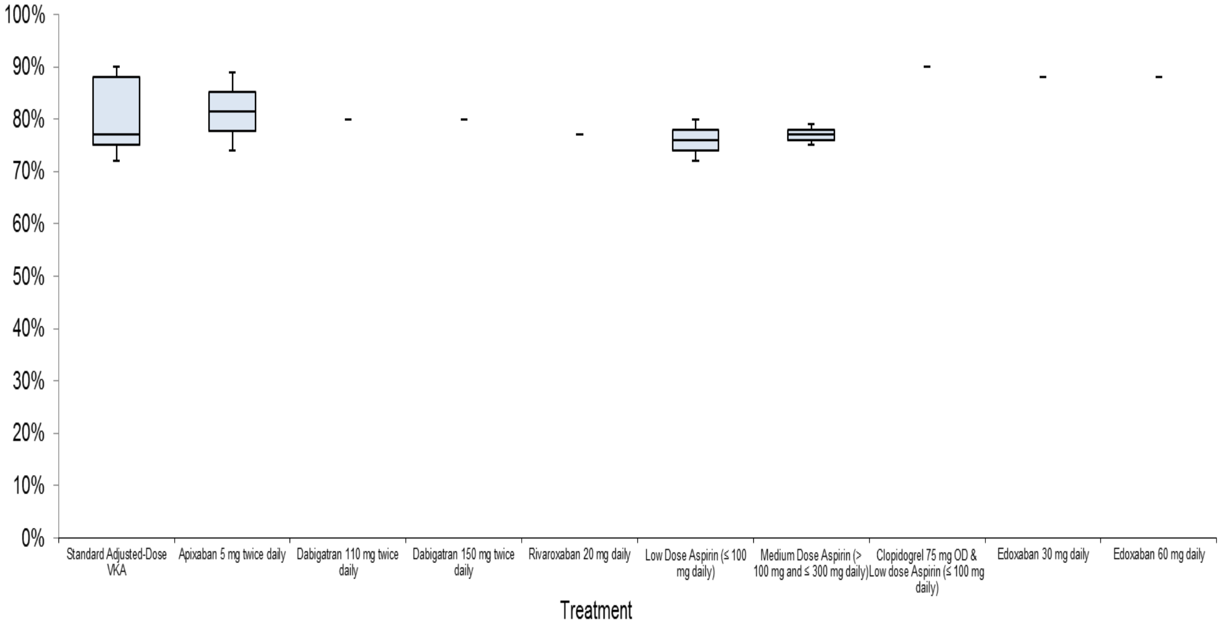

Systematic Review and Network Meta-analysis comparing Antithrombotic Agents for the Prevention of Stroke and Major Bleeding in Patients with Atrial Fibrillation

Journal:	*BMJ Open*
Manuscript ID:	bmjopen-2013-004301.R2
Article Type:	Research
Date Submitted by the Author:	26-Mar-2014

Complete List of Authors:	Cameron, Chris; University of Ottawa, Department of Epidemiology and Community Medicine<br>Coyle, Douglas; University of Ottawa, Epidemiology and Community Medicine<br>Richter, Trevor; Canadian Agency for Drugs and Technologies in Health,<br>Kelly, Shannon; Ottawa Heart Institute,<br>Gauthier, Kasandra; Canadian Agency for Drugs and Technologies in Health,<br>Steiner, Sabine; Medical University of Vienna, Department of Internal Medicine/Cardiology<br>Carrier, Marc; Ottawa Hospital Research Institute, Medicine<br>Coyle, Kathryn; Applied Health Economics Research Unit,<br>Bai, Annie; Canadian Agency for Drugs and Technologies in Health,<br>Moulton, Kristen; Canadian Agency for Drugs and Technologies in Health,<br>Clifford, Tammy; University of Ottawa, Department of Epidemiology and Community Medicine; University of Ottawa, Department of Pediatrics<br>Wells, George ; University of Ottawa, Department of Epidemiology and Community Medicine; University of Ottawa Heart Institute,

**Primary Subject Heading**:	Cardiovascular medicine
Secondary Subject Heading:	Epidemiology, Health policy
Keywords:	Stroke < NEUROLOGY, CARDIOLOGY, EPIDEMIOLOGY

Systematic Review and Network Meta-analysis comparing Antithrombotic Agents for the Prevention of Stroke and Major Bleeding in Patients with Atrial Fibrillation

Chris Cameron, PhD Student[1,2], Doug Coyle, Professor[1], Trevor Richter, Manager of Clinical Research [3], Shannon Kelly, Research coordinator[2] , Kasandra Gauthier, Clinical Research Officer[3], Sabine Steiner, Associate Professor[4], Marc Carrier, Associate Professor[6], Kathryn Coyle, Health Economist[5], Kristen Moulton, Clinical Research Officer[3], Annie Bai , Clinical Research Officer[3], Tammy Clifford, Adjunct Professor[1,7], George Wells, Professor[1,2]

[1] Department of Epidemiology and Community Medicine, University of Ottawa, Ottawa, Canada
[2] University of Ottawa Heart Institute, Ottawa, Canada
[3] Canadian Agency for Drugs and Technologies in Health, Ottawa, Canada
[4] Medical University of Vienna, Vienna, Austria
[5] Applied Health Economics Research Unit, Ottawa, Canada
[6] Thrombosis Program, Division of Hematology, Department of Medicine, University of Ottawa, Ottawa, Canada
[7] Department of Pediatrics, University of Ottawa, Ottawa, Canada

Word count, abstract: 322
Word count, body of essay: 3,987
Number of figures: 4
Number of tables: 1
Number of References: 44
Supplementary Material: 14

Corresponding author:
Dr. George Wells
Professor and Director, Cardiovascular Research Methods Centre
Department of Epidemiology and Community Medicine, University of Ottawa
Ottawa Heart Institute
40 Ruskin Street, Ottawa, Ontario, Canada K1Y 4W7
Phone: (613) 798-5555, ext. 18640
Fax: (613) 761-4412
Email: gawells@ottawaheart.ca

Keywords: atrial fibrillation, antithrombotic, anticoagulants, stroke, bleeding

For peer review only

Abstract

Objective: To examine the comparative efficacy and safety of antithrombotic treatments (dabigatran, rivaroxaban, apixaban, edoxaban, and vitamin K antagonists (VKA) at a standard adjusted dose, acetylsalicylic acid, acetylsalicylic acid and clopidogrel) for non-valvular atrial fibrillation and among sub-populations.

Design: Systematic review and network meta-analysis.

Data sources: A systematic literature search strategy using MEDLINE, EMBASE, Cochrane Register of Controlled Trials, and grey literature including the websites of regulatory agencies for trials published in English from 1988 to January 2014.

Eligibility criteria for selecting studies: Randomized controlled trials were included if they were published in English, used at least one antithrombotic treatment and involved patients with non-valvular atrial fibrillation eligible to receive anticoagulant therapy.

Results: For stroke or systemic embolism, apixaban and dabigatran 150mg twice daily were associated with reductions relative to standard adjusted dose VKA whereas low-dose ASA and the combination of clopidogrel plus low-dose ASA were associated with increases. Absolute risk reductions ranged from 6 fewer events per 1,000 patients treated for dabigatran 150mg twice daily to 15 more events for clopidogrel plus ASA. For major bleeding, apixaban, dabigatran 110mg twice daily, edoxaban 30mg daily, and edoxaban 60mg daily were associated with reductions compared to standard adjusted dose VKA. Absolute risk reductions with these agents ranged from 18 fewer per 1,000 patients treated each year for edoxaban 30 mg daily to 24 more for medium dose ASA.

Conclusions: Compared with standard adjusted dose VKA, new oral anticoagulants were associated with modest reductions in the absolute risk of stroke and major bleeding. People on antiplatelet drugs experienced more strokes compared with anticoagulant drugs without any reduction in bleeding risk. To fully elucidate the comparative benefits and harms of antithrombotic agents across the various sub-populations, rigorously conducted comparative studies or network meta-regression analyses of patient-level data are required.

Registration: PROSPERO-CRD42012002721

For peer review only

Article Summary

Strengths and limitations of this study

- This network meta-analysis includes data on edoxaban from the recently published ENGAGE AF-TIMI 48 trial
- The studies pooled in this meta-analysis include antiplatelet agents in addition to newer oral anticoagulants and standard adjusted dose vitamin K antagonists
- We present findings on both the relative and absolute scale, and graphically illustrate absolute risks using icon arrays
- We report detailed *a priori* sub-group analyses for stroke or systemic embolism and major bleeding using data derived from FDA Public Summary Documents
- Comparisons between antithrombotic treatments were largely comprised of single studies, albeit large studies
- There is notable heterogeneity and the small number of studies limits the analyses that can be conducted to account for heterogeneity in the absence of patient-level data

For peer review only - http://bmjopen.bmj.com/site/about/guidelines.xhtml

INTRODUCTION

Atrial fibrillation (AF) is a common cardiac arrhythmia associated with increased morbidity and mortality.[1,2] Patients with AF sustain an increased risk of arterial thromboembolism and stroke, associated with high recurrence and substantial debilitating impact. Therefore, antithrombotic strategies using anticoagulant drugs and antiplatelet agents are recommended for AF patients presenting with risk factors for stroke. Antithrombotic therapy is also associated with a risk of bleeding; therefore, the beneficial effects on stroke prevention should always be compared against a patient's risk of major bleeding.[1,2]

Existing guidelines recommend anticoagulant therapy for patients at intermediate or high risk of stroke.[1,2] Although standard adjusted dose vitamin K antagonists (VKA) (e.g., warfarin) has been the cornerstone treatment for reducing the risk of stroke or systemic embolism (SE) in this population, it is associated with several drawbacks.[2] which have prompted the development of newer oral anticoagulants such as direct thrombin (dabigatran) and Xa (e.g. apixaban, rivaroxaban, edoxaban) inhibitors. [1,2] While some of these newer treatment options have been demonstrated to be efficacious in preventing stroke or SE compared to standard adjusted dose VKA, the relative efficacy and risk of bleeding of newer oral anticoagulants, both among themselves and in comparison to antiplatelet agents, is not clear, especially in certain sub-populations of patients, e.g., $CHADS_2$ score <2 or ≥ 2; time in therapeutic range (TTR) $< 66\%$ or $\geq 66\%$; Age <75 or ≥ 75 years. Therefore, the objective of this paper was to compare antithrombotic agents for the prevention of stroke and major bleeding in patients with non-valvular AF and among sub-populations.

For peer review only - http://bmjopen.bmj.com/site/about/guidelines.xhtml

METHODS

Published literature was identified by searching MEDLINE, MEDLINE In-Process & Other Non-Indexed Citations, EMBASE, BIOSIS Previews, PubMed and the Cochrane Central Register of Controlled Trials were searched through the Ovid interface to identify English-language clinical articles published from 1988 to January 23, 2014. Regular alerts were also established. Websites of regulatory agencies, Canadian and major international health technology assessment agencies, clinical practice guidelines as well as The Cochrane Library (2012, Issue 6) and University of York Centre for Reviews and Dissemination databases were searched. Complete details of the electronic search strategy, including any limits used, are reported in Appendix 1. The protocol was published online and was registered in the PROSPERO International Prospective Register of Systematic Reviews (PROSPERO registry - CRD42012002721).

The population of interest was individuals with non-valvular AF requiring anticoagulation (including all risk levels and regardless of any comorbidities). The following treatments were included in the review: apixaban, dabigatran, edoxaban, rivaroxaban, standard adjusted dose VKA, acetylsalicylic acid (ASA), ASA and clopidogrel. Numerous outcomes were considered and results are reported elsewhere;[3] however, the focus of this publication is the outcomes of all-cause stroke or SE and major bleeding as defined by the International Society on Thrombosis and Haemostasis [ISTH] definition),[4] which were the primary efficacy and safety outcomes used in the newer oral anticoagulant studies.

For peer review only - http://bmjopen.bmj.com/site/about/guidelines.xhtml

Active and placebo-controlled randomized controlled trials (RCTs) were selected for inclusion if they were published in English, included at least one antithrombotic treatment under review (using pre-specified doses), reported data for stroke/SE or major bleeding, and involved patients with non-valvular AF eligible to receive anticoagulant therapy, regardless the level of stroke risk. Trials that included patients with contraindication to anticoagulant treatment were excluded. VKA trials were included if the dose was adjusted to a target international normalised ratio (INR) 2.0-3.0. Any dose of ASA was considered for inclusion, but ASA dose was stratified in the analysis as low (≤ 100 mg daily), medium (>100 mg to ≤ 300 mg daily), or high (>300 mg daily). We only included new oral anticoagulants which had at least one large phase III study. Three reviewers independently extracted the data on baseline characteristics, intervention(s) evaluated, including dose, duration, and relevant co-medication and results for each included article, using a standardized template. All extracted data was checked for accuracy by three independent reviewers. Any disagreements in the assessment of these data were resolved through discussion until consensus was reached. Quality assessment of RCTs was also performed independently by two reviewers using a standardized table based on major items from the SIGN 50 instrument for internal validity.[5] The trial selection process is presented in a flowchart based on the Preferred Reporting Items for Systematic Reviews and Meta-Analyses (PRISMA)[6] statement (Appendix 2).

Bayesian network meta-analyses were conducted using WinBUGS software (MRC Biostatistics Unit, Cambridge, UK). A binomial likelihood model[7] which accounts for the use of multi-arm trials was used for the analyses given the datasets provided were dichotomous outcomes and included multi-arm trials. Trials with zero cells in both arms or nodes where there were no events

For peer review only - http://bmjopen.bmj.com/site/about/guidelines.xhtml

were excluded from the evidence networks because they do not contribute information or allow interpretable information.[7] Both fixed and random-effects network meta-analyses were conducted, although the fixed-effects model was chosen for the reference case analysis, as the evidence network was largely comprised of single study connections. We modeled point estimates and 95% credible intervals for odds ratios (OR) using Markov Chain Monte Carlo methods. The absolute risk difference per 1000 patients treated each year for each outcome was also calculated, based on the standard adjusted dose VKA arm of the Randomized Evaluation of Long-Term Anti-coagulation Therapy (RE-LY) trial.[8] The RE-LY trial[8] was selected because it contained data for both $CHADS_2<2$ and $CHADS_2 \geq 2$ and had detailed data available from the Food and Drug Administration (FDA) Public Summary Report.[9] We also assessed the probability that each treatment was the most efficacious regimen, the second best, the third best, and so on.[10] Vague or flat priors, such as $N(0, 100^2)$ were assigned for basic parameters throughout, although informative priors were considered.[11] Assessment of model fit was based on deviance information criterion (DIC) and comparison of residual deviance to number of unconstrained data points.[7,12] To ensure convergence was reached, trace plots and the Brooks-Gelman-Rubin statistic were assessed.[13] Three chains were fit in WinBUGS for each analysis, with at least 40,000 iterations, and a burn-in of at least 40,000 iterations. We also qualitatively compared the results from our network meta-analysis with direct pair-wise estimates. Frequentist pair-wise meta-analyses were conducted using R (R Language and Environment for Statistical Computing, Vienna, Austria) and package meta.[14]

A key assumption behind network meta-analysis is that the analysed network is consistent, i.e., there is no conflict between direct and indirect evidence.[15] To assess inconsistency, we compared

For peer review only - http://bmjopen.bmj.com/site/about/guidelines.xhtml

deviance and DIC statistics in fitted consistency and inconsistency models.[15] We also plotted the posterior mean deviance of the individual data points in the inconsistency model against their posterior mean deviance in the consistency model to identify any loops in where inconsistency is present (Appendix 11).[15] Additionally, the results from our network meta-analysis were qualitatively compared with direct frequentist pair-wise estimates.

Network meta-analysis also requires that studies are sufficiently similar in order to pool their results.[16] Available study and patient characteristics were assessed to ensure similarity and to investigate the potential impact of heterogeneity on effect estimates. Clinical and methodological heterogeneity was identified in a number of areas. The issues identified were similar to those reported in previous systematic reviews of anticoagulant drugs (e.g., differences in TTR, $CHADS_2$ score).[17] Heterogeneity was assessed by conducting network meta-analysis with the following pre-specified subgroup data reported in the individual RCTs: TTR $< 66\%$ or $\geq 66\%$; $CHADS_2$ score <2 or ≥ 2; Age <75 or ≥ 75 years.[17] The data to conduct these sub-group analyses were derived from FDA Public Summary Documents when not available in the publications.[9,18,19] Secondary evidence networks were constructed for each of the sub-group analyses. The WinBUGS code[7] and data needed to replicate all analyses are available online.

This research was supported through a grant by the Canadian Institute of Health Research (CIHR) Drug Safety and Effectiveness Network and financial support from the Canadian Agency for Drugs and Technologies in Health (CADTH).

For peer review only - http://bmjopen.bmj.com/site/about/guidelines.xhtml

RESULTS

Study Characteristics of included studies

The systematic review included 16 individual RCTs (reported in 32 publications and FDA reports); all evaluated the efficacy and safety of antithrombotic agents: dabigatran (150 or 110 mg twice daily), rivaroxaban (20 mg daily), apixaban (5 mg twice daily), edoxaban (30 or 60 mg daily), standard adjusted dose VKA, ASA (low dose (< 100 mg daily); medium dose (100-300 mg daily), or low dose-ASA plus clopidogrel (75 mg daily) in patients with non-valvular AF. None of the included studies directly compared one new oral anticoagulant drug with another (Appendix 4). Of the 82,396 randomized patients included in the primary analysis, five large multicentre trials account for 78,296 patients (96%): ENGAGE AF-TIMI 48 (n=21,105),[20] ARISTOTLE (n=18,201),[21] RE-LY (n=18,113),[8] ROCKET-AF (n=14,264)[22] and ACTIVE-W (n=6706).[23] Most trials included stroke and SE as a primary efficacy outcome, while bleeding events were a frequent safety outcome. Follow-up ranged from 12 weeks to 3.5 years. All trials published results between 1989 and 2013. Mean $CHADS_2$ score was reported in eight trials, encompassing the vast majority of patients included in the systematic review. Reported $CHADS_2$ scores were consistent with a high-risk population ($CHADS_2 \geq 2$), with patients from ROCKET-AF[22] showing the highest risk for stroke (mean $CHADS_2 = 3.5$).

Mean age across the included trials ranged from 62 years to 83 years (Appendix 3). All trials included patients of both gender and had a higher proportion of male participants in most studies. However, the proportions of patients with a prior stroke or TIA varied substantially across the included trials, ranging from 3% in JAST to 55% in ROCKET-AF.[22] The same observation can be made regarding the proportions of patients with other concomitant conditions, i.e. congestive

For peer review only - http://bmjopen.bmj.com/site/about/guidelines.xhtml

heart failure, hypertension, diabetes, myocardial infarction, and prior vitamin K antagonist experience. TTR was reported in all but one trial that included a standard adjusted dose VKA treatment arm. Patient baseline characteristics are summarized in Appendix 3.

The studies were individually critically appraised and the details are reported elsewhere.[3] Overall, there was substantial variation in study quality. However, large multicentre trials comparing newer anticoagulants with standard adjusted dose VKA such as ENGAGE AF-TIMI 48 (n=21,105), ARISTOTLE (n = 18,201), RE-LY (n = 18,113), and ROCKET-AF (n = 14,264), which account for the vast majority of patients included in the systematic review, appear to be methodologically rigorous.

Stroke or Systemic Embolism

The evidence network (Figure 1) for the primary analysis was comprised of twelve RCTs representing eight treatments in addition to placebo/observation (N =82,396). Four RCTs (PETRO,[24] WASPO[25], Chung et al,[26] and Yamashita et al[27]) were not included in the analysis because they did not report data for this outcome or had zeroes in both arms.

Apixaban and dabigatran (150 mg twice daily) were associated with reductions in stroke or SE relative to standard adjusted dose VKA (Figure 2). The use of these two agents led to absolute risk reductions ranging from 4 to 6 fewer events per 1,000 patients treated each year. By contrast, low-dose ASA and the combination of clopidogrel plus low-dose ASA appeared to have a higher risk of stroke or SE than standard adjusted dose VKA, leading to an increase in the number of stroke or SE ranging from 14 to 15 more events per 1,000 patients treated each year

For peer review only - http://bmjopen.bmj.com/site/about/guidelines.xhtml

(Figure 4 and Appendix 6). No differences were detected between standard adjusted dose VKA and each of the following interventions: rivaroxaban, edoxaban (30 mg or 60 mg daily), dabigatran (110 mg twice daily), and medium-dose ASA (Figure 3).

The estimates of effect derived from the direct pair-wise comparisons aligned well with those obtained from the network meta-analysis in both direction and magnitude (Appendix 6). Furthermore, the posterior mean residual deviance (29.41) is close to the number of unconstrained data points (27), which is an indication of reasonable model fit. A number of sub-group analyses and alternative modeling strategies were conducted on the primary analysis, including adjusting for $CHADS_2$, TTR, and age (Table 1). We also conducted a sensitivity analysis where we included the AVERROES[28] and ACTIVE A[29] trials (Appendix 12). These studies were excluded from the main analysis because we required patients to be eligible for anticoagulant therapy, including treatment with a VKA. The results differed slightly from the reference case (Appendix 12). The primary analysis was conducted using a fixed-effects model because the network was comprised of single study connections; these results were also compared against those obtained using a random-effects model with informative and vague priors on the standard deviation, and found to be similar in magnitude although wider credible intervals were observed (Appendix 9).

Major bleeding

The evidence network for the primary analysis for major bleeding was comprised of 15 RCTs representing eight treatments in addition to placebo/observation (N =83,015). The evidence network for major bleeding is similar to the evidence network for stroke or SE (Figure 1) but

For peer review only - http://bmjopen.bmj.com/site/about/guidelines.xhtml

includes one extra RCT (WASPO[25]) comparing medium dose ASA with standard adjusted dose VKA, and two extra RCTs (Chung et al,[26] and Yamashita et al[27]) comparing edoxaban with standard adjusted dose VKA (Appendix 5).

Apixaban, dabigatran 110 mg twice daily, edoxaban 30 mg daily, and edoxaban 60 mg daily were associated with reductions in the risk of major bleeding compared with standard adjusted dose VKA (Figure 2). No differences for major bleeding were detected between standard adjusted dose VKA and each of the remaining interventions: rivaroxaban, dabigatran 150 mg twice daily, clopidogrel plus low-dose ASA, and all ASA dosages (Figure 2). The absolute risk difference of major bleeding relative to standard adjusted dose VKA ranged from 18 fewer to 24 more events per 1000 patients treated per year (Figure 4 and Appendix 7). A summary of the results for the Bayesian and direct pair-wise meta-analyses is shown in Appendix 7, and Bayesian network meta-analysis results are represented graphically in Figure 2. Complete results from the Bayesian network meta-analysis for all possible comparisons are presented in Figure 3.

The estimates of effect derived from the direct pair-wise comparisons aligned well with those obtained from the network meta-analysis in both direction and magnitude (Appendix 7). Furthermore, the posterior mean residual deviance (38.0) is close to the number of unconstrained data points (35), which is an indication of reasonable model fit. A number of sub-group analyses and alternative modeling strategies were conducted on the primary analysis (Table 1). We also conducted a sensitivity analysis where we included the AVERROES[28] and ACTIVE A[29] trial (Appendix 12), as well as analyses using a random-effects model with vague and informative priors (Appendix 9).

For peer review only

Benefit-Harm Assessment – Stroke or systemic embolism versus major bleeding

Figure 3 summarises the results for all pair-wise comparisons derived from the Bayesian fixed-effects network meta-analysis (Appendix 9). Results relative to standard adjusted dose VKA have been discussed above. For pair-wise comparisons among newer oral anticoagulants for stroke or SE, dabigatran 150 mg twice daily was associated fewer events versus dabigatran 110 mg twice daily, rivaroxaban, edoxaban 30mg daily, and edoxaban 60 mg daily. Apixaban, and rivaroxaban were also associated with fewer events compared to edoxaban 30mg daily. There were no differences among low-dose ASA, medium-dose ASA, and clopidogrel plus low-dose ASA. However, low-dose ASA and the combination of clopidogrel plus low-dose ASA were associated with increases in stroke or systemic embolism compared to all newer oral anticoagulants. For major bleeding, apixaban, dabigatran 110 mg twice daily were associated with fewer events versus dabigatran 150 mg twice daily and rivaroxaban. Edoxaban 30 mg daily was associated with fewer events than other new oral anticoagulants, while edoxaban 60 mg daily was associated with fewer events compared with rivaroxaban and clopidogrel plus low-dose ASA. The risk of major bleeding for apixaban was lower compared to clopidogrel plus low-dose ASA. There were no differences associated with the ASA treatments, both for comparisons among themselves and compared to the anticoagulant treatments.

Figure 4 plots the absolute risk for stroke or SE and major bleeding per 1000 treated for the 10 treatments analyzed. Data in Figure 4 were obtained from the results presented in Appendix 6 and 7. Examination of the data in Figure 4 suggests that the balance of benefits and harms of the new oral anticoagulants for stroke or systemic embolism and major bleeding is positive compared to standard adjusted dose VKA (decrease in stroke or systemic embolism and/or major bleeding) and largely similar among one another. Comparison of all anticoagulant treatment

For peer review only - http://bmjopen.bmj.com/site/about/guidelines.xhtml

(including standard adjusted dose VKA) to ASA, or ASA in combination with clopidogrel indicates that whereas the new oral anticoagulants decrease the risk of both stroke or systemic embolism and major bleeding, ASA has a less favorable balance of benefits and harms, i.e., fails to minimize the risk of stroke or systemic embolism and/or major bleeding.

DISCUSSION

We identified 16 RCTs comparing antithrombotic agents for the prevention of all cause stroke or systemic embolism and major bleeding in patients with AF. To the best of our knowledge, this is the most up-to-date systematic review and network meta-analysis[30–34] to synthesize the available benefits and harms on newer oral anticoagulants, standard adjusted dose VKA, and antiplatelet agents for the prevention of stroke and major bleeding in patients with non-valvular AF (Appendix 13). Harenberg et al[30] recently summarized the published indirect comparisons and network meta-analyses evaluating newer oral anticoagulants for AF and an earlier unpublished version of our analysis[17] was considered among the most comprehensive analyses at that time.[30] This network meta-analysis expands upon that unpublished report.[17] Overall, our findings align with those reported in previously published network meta-analyses;[30,31,33] however, unlike previous analyses,[30] we include recently published edoxaban data,[20] and also include antiplatelet agents. We also present detailed evidence networks[35] to illustrate the body of evidence for each outcome; report analysis using multiple statistical models (Appendix 9); report detailed *a priori* sub-group analyses for each outcome using data derived from FDA Public Summary Documents;[9,18] present findings on both the relative and absolute scale,[36,37] and graphically illustrate results using icon arrays. Our network meta-analysis was registered in PROSPERO, and also adheres to PRISMA reporting standards (Appendix 10). It also differs from a recently

For peer review only - http://bmjopen.bmj.com/site/about/guidelines.xhtml

published traditional meta-analysis by Ruff et al[32] given we report comparison of newer oral anticoagulants versus each other, and also include comparisons with antiplatelet agents.

For stroke or systemic embolism, apixaban and dabigatran 150 mg twice daily were associated with reductions in stroke or systemic embolism relative to standard adjusted dose VKA. By contrast, low-dose ASA and the combination of clopidogrel plus low-dose ASA appeared were associated with increases in stroke or systemic embolism relative to standard adjusted dose VKA. No differences were detected between standard adjusted dose VKA and each of the following interventions: rivaroxaban, dabigatran 110 mg twice daily, edoxaban 30 and 60 mg daily, medium-dose ASA, and no treatment/placebo. The absolute risk reduction of stroke or systemic embolism relative to standard adjusted dose VKA ranged from 6 fewer to 15 more events per 1000 patients treated per year, with dabigatran 150 mg twice daily yielding the largest aboslute risk reduction and clopidogrel plus low-dose ASA the largest absolute risk increase.

For major bleeding, apixaban, dabigatran 110 mg twice daily, edoxaban 30 mg daily, and edoxaban 60 mg daily were associated with a decreased risk of major bleeding relative to standard adjusted dose VKA. No differences for major bleeding were detected between standard adjusted dose VKA and each of the remaining interventions: rivaroxaban, dabigatran 150 mg twice daily, clopidogrel plus low-dose ASA, and all ASA dosages. The absolute risk difference of major bleeding relative to standard adjusted dose VKA ranged from 18 fewer to 24 more events per 1000 patients treated, with edoxaban 30 mg daily yielding the largest absolute risk reduction and medium dose ASA the largest absolute risk increase. Results of network meta-analyses reflect the relative efficacy and safety profile of antiplatelet and anticoagulant drugs as

For peer review only - http://bmjopen.bmj.com/site/about/guidelines.xhtml

documented by large clinical trials. Thus, international guidelines[1,2] for prevention of stroke in patients with AF also recommended oral anticoagulation over antiplatelet therapy as the latter had only modest protective effects. With respect to the new oral anticoagulants none of the guidelines has given a recommendation to prefer one of the new oral anticoagulants over another.[1,2]

Network meta-analysis involves pooling of trials. We observed variability across studies included in the evidence network in terms of $CHADS_2$, age and TTR (Appendix 3 and 4). For example, patients using rivaroxaban had a higher risk of stroke because ROCKET-AF[22] included patients with $CHADS_2 \geq 2$. As such, we conducted sub-group analysis to adjust for differences in patient and study-level characteristics. We derived data for sub-group analyses from FDA Public Summary documents[9,18,19] which enhanced our ability to account for variability across studies (e.g., use intention to treat (ITT) population for rivaroxaban versus per-protocol as-treated population reported in publication[22]). Results from these sub-group analyses differed slightly from those reported in the primary analysis, indicating that there may be differences in the benefits and harms across sub-groups. This becomes more apparent when sub-group analyses are reported on the absolute scale.[3] It is unclear whether the absolute risk reductions associated with these differences translate into clinically meaningful benefits in practice, especially in sub-populations of patients with a lower baseline risk of stroke or systemic embolism. To fully elucidate the comparative benefits and harms of antithrombotic agents across the various sub-populations, rigorously conducted comparative studies,[38,39] or network meta-regression analyses of patient-level data[40] are required.

For peer review only - http://bmjopen.bmj.com/site/about/guidelines.xhtml

For peer review only

There are several limitations with the analysis we employed. First, there is notable heterogeneity in patients and study characteristics (Appendix 3, 4). However, the small number of studies limits the analyses that can be conducted to account for heterogeneity in the absence of patient-level data. Second, there is insufficient sub-group data available for other outcomes reported in the RCTs included. As a consequence, detailed analysis was limited to two outcomes – stroke and SE and major bleeding. Third, we reported the results from the fixed-effects model in the main text. We felt that this was appropriate as the nodes in evidence networks are connected largely by single studies. Effect estimates derived from the fixed-effects model aligned more closely with the direct estimates. Reporting results from the fixed-effects model in the main text likely biases results in favour of the new oral anticoagulants, as treatments that achieved statistically significant results in the primary RCTs retained statistically significant findings when using the fixed-effects model, but not always when using the random-effects model due to the prior[11] on the between study variance. Nevertheless, the results from the random-effects model have also been reported in Appendix 9, along with detailed discussion around the limitations in using the fixed-effects model. Fourth, the absolute risk reduction for the network meta-analysis was calculated using the event rate in the standard adjusted dose VKA arm of the RE-LY study. The RE-LY trial was selected because it contained data for both $CHADS_2 < 2$ and $CHADS_2 \geq 2$ and had detailed data made publicly available following an FDA review of dabigatran. Use of a different study (e.g., ARISTOTLE) for baseline event rate data is unlikely to change the results substantially given that the event rates in standard adjusted dose VKA arms were similar across studies. Fifth, we present findings using graphics that have been largely applied in the network meta-analysis literature,[41] such as a table with odds ratios for all pair-wise comparisons. This method of presenting findings on the relative scale is problematic when you

For peer review only - http://bmjopen.bmj.com/site/about/guidelines.xhtml

are conducting assessments to compare the balance of benefits and harms, which need to be standardized. As such, we also provided results in absolute terms. We report our findings using icon arrays, although it should be noted that these results do not account for the utility values based upon patient preferences for each of the outcomes,[42,43] nor do they reflect uncertainty although we report absolute reductions on the risk benefit plane reflecting uncertainty in Appendix 8. There are other methods available that can incorporate patient preferences for outcomes.[42,43] Further, estimates of benefit and harm with several of the therapies (e.g., rivaroxaban) come from one trial and thus such data are not particularly robust. Further research is needed comparing the balance of benefit and harms using other research methodologies, incorporating other relevant outcomes, patient preferences for each of the outcomes,[42,43] and additional studies when they become available. Finally, haemorrhagic stroke is also considered a major bleed in underlying studies. Accordingly, we may have double counted haemorrhage stroke in this analysis. Unfortunately, we are not able to account for this issue in all included studies. Nonetheless, it is important to note this limitation and highlight that this potentially biases results in favor of newer oral anticoagulants given these agents were associated with reductions in these events.

CONCLUSIONS

Compared with standard adjusted dose VKA, apixaban and dabigatran 150 mg twice daily were associated with reductions in the incidence of stroke or systemic embolism in patients with AF, whereas, low-dose ASA and the combination of clopidogrel plus low-dose ASA increased the risk relative to standard adjusted dose VKA at preventing stroke or systemic embolism. Apixaban, dabigatran 110 mg twice daily, edoxaban 30 mg daily, and edoxaban 60 mg daily and

For peer review only - http://bmjopen.bmj.com/site/about/guidelines.xhtml

was associated with a reduced risk in major bleeding compared with standard adjusted dose VKA. Although the results of the current review revealed that there were differences in stroke or systemic embolism and major bleeding among antithrombotic agents in the management of patients with AF in relative terms, it is unclear whether the absolute risk reductions associated with these differences translate into clinically meaningful benefits in practice, especially in sub-populations of patients with a lower baseline risk of stroke or systemic embolism. Rigorously conducted comparative studies or network meta-regression analyses of patient-level data are required to fully elucidate the comparative benefits and harms of antithrombotic agents across the various sub-populations.

For peer review only

For peer review only - http://bmjopen.bmj.com/site/about/guidelines.xhtml

TABLES

Table 1: Sub-group and sensitivity analyses for stroke or systemic embolism and major bleeding relative to standard adjusted dose VKA, Odds ratios ± 95% Credible Intervals versus standard adjusted dose VKA

Treatment	Reference Case – Fixed Effect NMA	Subgroup analysis by $CHADS_2$ Score		Subgroup analysis by Age		Subgroup analysis by TTR	
		$CHADS_2 < 2$	$CHADS_2 \geq 2$	Age < 75 years	Age ≥ 75 years	TTR < 66%	TTR ≥ 66%
**Stroke or Systemic Embolism**							
**Apixaban 5 mg twice daily**	**0.78 (0.65,0.94)**	0.86 (0.57,1.28)	**0.78 (0.63,0.95)**	0.85 (0.67,1.07)	**0.71 (0.53,0.95)**	0.79 (0.62,1.003)	0.79 (0.60,1.05)
**Dabigatran 110 mg twice daily**	0.91 (0.74,1.12)	1 (0.64,1.55)	0.89 (0.71,1.12)	0.93 (0.7,1.24)	0.88 (0.66,1.19)	0.91 (0.69,1.19)	0.9 (0.66,1.24)
**Dabigatran 150 mg twice daily**	**0.66 (0.53,0.82)**	0.61 (0.37,1)	**0.66 (0.52,0.85)**	0.63 (0.46,0.87)	**0.66 (0.48,0.9)**	0.54 (0.39,0.73)	0.81 (0.58,1.11)
**Rivaroxaban 20 mg daily**	0.87 (0.74,1.03)	NA	0.88 (0.74,1.04)	0.95 (0.75,1.2)	0.8 (0.63,1.03)	0.81 (0.65,1.01)	0.72 (0.47,1.1)
**Low Dose ASA (≤ 100 mg daily)**	**1.87 (1.26,2.8)**	2.2 (1.18,4.25)	2.1 (0.91,5.15) *	NA	2.04 (1.33,3.20)	NA	NA
**Medium Dose ASA (> 100 mg and ≤ 300 mg daily)**	1.35 (0.74,2.47)	1.97 (0.63,6.73) **	NA	NA	NA	NA	NA
**Clopidogrel 75 mg daily & Low dose Aspirin (≤ 100 mg daily)**	**1.93 (1.42,2.65)**	3.16 (1.39,8.22)	1.15 (0.84,1.57)	NA	NA	1.52 (0.96,2.45)	**2.4 (1.59,3.71)**
**Edoxaban 30 mg daily**	1.14 (0.98,1.32)	NA	1.14 (0.98,1.32)	1.16 (0.94,1.44)	1.13 (0.91,1.4)	1.01 (0.80,1.28)¶	1.24 (1.01,1.24)¶
**Edoxaban 60 mg daily**	0.87 (0.74,1.01)	NA	0.87 (0.74,1.01)	0.91 (0.73,1.14)	0.83 (0.66,1.04)	0.83 (0.65,1.07)¶	0.91 (0.73,1.13)¶
**Major Bleeding**							
**Apixaban 5 mg twice daily**	**0.69 (0.6,0.8)**	**0.59 (0.44,0.78)**	**0.73 (0.62,0.87)**	**0.73 (0.6,0.89)**	**0.65 (0.52,0.80)**	**0.57 (0.45,0.71)**	**0.81 (0.67,0.98)**
**Dabigatran 110 mg twice daily**	**0.8 (0.69,0.93)**	**0.65 (0.47,0.89)**	0.86 (0.73,1.02)	**0.62 (0.49,0.77)**	1.02 (0.84,1.25)	0.74 (0.61,0.91)	0.86 (0.69,1.06)
**Dabigatran 150 mg twice daily**	0.93 (0.8,1.08)	0.77 (0.57,1.04)	1.01 (0.86,1.19)	**0.7 (0.56,0.86)**	1.19 (0.98,1.45)	0.76 (0.62,0.94)	1.15 (0.94,1.4)
**Rivaroxaban 20 mg daily**	1.03 (0.89,1.18)	NA	1.03 (0.89,1.19)	0.93 (0.76,1.13)	1.15 (0.93,1.41)	0.92 (0.77,1.1)	**1.3 (1.01,1.69)**
**Low Dose ASA (≤ 100 mg daily)**	1.05 (0.6,1.86)	0.83 (0.42,1.64)	1.6 (0.55,5.02) *	NA	1.01 (0.57,1.78)	NA	NA

For peer review only - http://bmjopen.bmj.com/site/about/guidelines.xhtml

Treatment	Reference Case – Fixed Effect NMA	Subgroup analysis by CHADS₂ Score		Subgroup analysis by Age		Subgroup analysis by TTR	
		$CHADS_2$ < 2	$CHADS_2$ ≥ 2	Age < 75 years	Age ≥ 75 years	TTR < 66%	TTR ≥ 66%
**Medium Dose ASA (> 100 mg and ≤ 300 mg daily)**	1.79 (0.63,5.67)	1.48 (0.11,19.07)**	NA	NA	NA	NA	NA
**Clopidogrel 75 mg daily & Low dose Aspirin (≤ 100 mg daily)**	1.1 (0.83,1.47)	1.51 (0.89,2.61)	0.97 (0.69,1.36) †	NA	NA	0.66 (0.42,1.02)	1.66 (1.12,2.47)
**Edoxaban 30 mg daily**	**0.46 (0.4,0.54)**	NA	**0.46 (0.4,0.54)**	0.50 (0.41,0.61) ‡	0.51 (0.41-0.62) ‡	0.45 (0.35,0.58) ‡¶	0.51 (0.43,0.61) ‡¶
**Edoxaban 60 mg daily**	**0.79 (0.69,0.9)**	NA	**0.79 (0.69,0.9)**	0.74 (0.62-0.90) ‡	0.82 (0.68-0.98) ‡	0.69 (0.55,0.87) ‡¶	0.83 (0.70,0.97) ‡¶

ASA= acetylsalicylic acid; mg= milligram; NA=not available; NMA= network meta-analysis; OR=odds ratio; TTR= time in therapeutic range; VKA=vitamin K antagonist

* Results may be slightly biased against LDASA. Derived from BAFTA study where $CHADS_2$ subgroup data stratified by $CHADS_2$ 1-2 (vs. 0-1) and $CHADS_2$ 3-6 (vs. 2-6).

** Results may be slightly biased against medium-dose ASA. Based on study level results from CAFA and JAST studies. Although these studies consisted primarily of low-risk populations, some patients may have $CHADS_2$ scores greater than 2.

† Estimated from subgroup study by Healey et al 2008

¶ Data provided from ENGAGE AF-TIMI 48[20] uses TTR greater than 60%

‡ Based on overall time period where overall time period was defined as first dose to the last dose plus 3 days.

For peer review only

For peer review only - http://bmjopen.bmj.com/site/about/guidelines.xhtml

For peer review only

For peer review only - http://bmjopen.bmj.com/site/about/guidelines.xhtml

Acknowledgments: The authors would like to acknowledge Carolyn Spry. She was the Information Specialist from CADTH on the review who conducted the search strategy. We would also like to thank Dr. Robert Giugliano for providing the sub-group data for edoxaban and his thoughtful comments on earlier drafts of the manuscript during the peer review process.

Contributors: CC conceived the study design, performed the data extraction, analysis, and drafted the manuscript. DC and GW conceived the study design, helped with the data analysis, and reviewed the manuscript for important intellectual content. TR conceived the study design, performed the systematic review, data extraction, and reviewed the manuscript for important intellectual content. SK, SS, KG, AB, KM, KC performed the systematic review, data extraction, and reviewed the manuscript for important intellectual content. TC, MC, and KC conceived the study and reviewed the manuscript for important intellectual content. All authors have read and approved the final version of the manuscript. All authors agree to be accountable for all aspects of the work in ensuring that questions related to the accuracy or integrity of any part of the work are appropriately investigated and resolved.

Funding information: This research was supported through a grant by the Canadian Institute of Health Research (CIHR) Drug Safety and Effectiveness Network and financial support from the Canadian Agency for Drugs and Technologies in Health (CADTH). The CIHR Drug Safety and Effectiveness Network played no role in study design, collection, analysis, interpretation of data, writing of the manuscript, or in the decision to submit the paper for publication. They accept no responsibility for the contents. Employees from CADTH were involved in the study design, collection, analysis, interpretation of data, and writing of the manuscript. CADTH, funded by Canadian federal, provincial and territorial governments, is an independent, not-for-profit producer and broker of health technology assessments. Federal, provincial, and territorial health care decision-makers rely on our evidence-based information to make informed decisions about the effectiveness and efficiency of drugs and other health technologies.

Competing interests: All authors have completed the ICMJE uniform disclosure form at www.icmje.org/coi_disclosure.pdf (available on request from the corresponding author) and declare: no support from any organisation for the submitted work; CC is a recipient of a Vanier Canada Graduate Scholarship from the Canadian Institutes of Health Research and has received funding from Canadian Network and Centre for Trials Internationally (CANNeCTIN) and is a trainee on the CIHR Drug Safety and Effectiveness Network team grant. MC is a recipient of a New Investigator Award from the Heart and Stroke Foundation of Canada and holds a T2 research chair in cancer and thrombosis from the University of Ottawa.

Ethical approval: Not required.

Data sharing: The statistical code and dataset used for analyses are reported online: http://www.cadth.ca/media/pdf/TR0003_AntithromboticAgents-AF_ScienceReport-e.pdf. Additional data can be requested by emailing George Wells: gawells@ottawaheart.ca .

The Corresponding Author has the right to grant on behalf of all authors and does grant on behalf of all authors, an exclusive licence (or non exclusive for government employees) on a worldwide basis to the BMJ Publishing Group Ltd to permit this article (if accepted) to be published in BMJ editions and any other BMJPGL products and sublicences such use and exploit all subsidiary rights, as set out in our licence

For peer review only - http://bmjopen.bmj.com/site/about/guidelines.xhtml

REFERENCES

1. Kirchhof, P. *et al.* Atrial fibrillation guidelines across the Atlantic: a comparison of the current recommendations of the European Society of Cardiology/European Heart Rhythm Association/European Association of Cardiothoracic Surgeons, the American College of Cardiology Fou. *Eur. Heart J.* **34,** 1471–1474 (2013).

2. Skanes, A. C. *et al.* Focused 2012 update of the Canadian Cardiovascular Society atrial fibrillation guidelines: recommendations for stroke prevention and rate/rhythm control. *Can. J. Cardiol.* **28,** 125–36 (2012).

3. *Antithrombotic agents for the prevention of stroke and systemic embolism in patients with atrial fibrillation.* (2013). at <http://www.cadth.ca/media/pdf/TR0003_AntithromboticAgents-AF_ScienceReport-e.pdf>

4. Schulman, S. & Kearon, C. Definition of major bleeding in clinical investigations of antihemostatic medicinal products in non-surgical patients. *J. Thromb. Haemost.* **3,** 692–4 (2005).

5. NHS Quality Improvement Scotland (NHS QIS). *SIGN 50. A guideline developer's handbook.* (2008). at <http://www.sign.ac.uk/pdf/sign50.pdf>

6. Moher, D., Liberati, A., Tetzlaff, J. & Altman, D. G. Preferred reporting items for systematic reviews and meta-analyses: the PRISMA statement. *PLoS Med.* **6,** e1000097 (2009).

7. Dias, S., Welton, N. J., Sutton, A. J. *et al. NICE DSU Technical Support Document 2: A Generalised Linear Modelling Framework for Pairwise and Network Meta-Analysis of Randomised Controlled Trials. Trop. Med.* 1–96 (2011). at <www.nicedsu.org.uk>

8. Connolly, S. & Ezekowitz, M. Dabigatran versus warfarin in patients with atrial fibrillation. *N. Engl. J. Med.* **361,** 1139–1151 (2009).

9. Ingelheim, B. *Advisory committee briefing document: dabigatran etexilate [Internet].* 1–168 (2010). at <http://www.fda.gov/downloads/advisorycommittees/committeesmeetingmaterials/drugs/cardiovas>

10. Cipriani, A. *et al.* Comparative efficacy and acceptability of 12 new-generation antidepressants: a multiple-treatments meta-analysis. *Lancet* **373,** 746–58 (2009).

11. Turner, R. M., Davey, J., Clarke, M. J., *et al.* Predicting the extent of heterogeneity in meta-analysis, using empirical data from the Cochrane Database of Systematic Reviews. *Int. J. Epidemiol.* **41,** 818–27 (2012).

For peer review only - http://bmjopen.bmj.com/site/about/guidelines.xhtml

12. Spiegelhalter, D. J., Best, N. G., Carlin, B. P. *et al*. Bayesian measures of model complexity and fit. *J. R. Stat. Soc. Ser. B (Statistical Methodol.* **64,** 583–639 (2002).

13. Spiegelhalter, D., Thomas, A. & Best, N. WinBUGS user manual. *MRC Biostat. Unit* **2,** (2004).

14. meta: Meta-Analysis with R. at <http://cran.r-project.org/web/packages/meta/index.html>

15. Dias, S., Welton, N.J., Sutton, A.J., *et al*. *NICE Technical Support Document 4: Inconsistency in Networks of Evidence Based on Randomised Controlled Trials*. 1–39 (2011). at <www.nicedsu.org.uk>

16. Salanti, G., Marinho, V. & Higgins, J. P. T. A case study of multiple-treatments meta-analysis demonstrates that covariates should be considered. *J. Clin. Epidemiol.* **62,** 857–64 (2009).

17. Wells, G., Coyle, D., Cameron, C., *et al*. *Safety, effectiveness, and costeffectiveness of new oral anticoagulants compared with warfarin in preventing stroke and other cardiovascular events in patients with atrial fibrillation*. (2012). at <http://www.cadth.ca/media/pdf/NOAC_Therapeutic_Review_final_report.pdf>

18. Johnson & Johnson Pharmaceutical Research & Development. *Rivaroxaban for the Prevention of Stroke and Non-Central Nervous System (CNS) Systemic Embolism in Patients With Atrial Fibrillation*. **7939,** 222 (2011).

19. McDonagh, M. S., Peterson, K., Balshem, H. *et al*. US Food and Drug Administration documents can provide unpublished evidence relevant to systematic reviews. *J. Clin. Epidemiol.* (2013). doi:10.1016/j.jclinepi.2013.05.006

20. Giugliano, R. P. *et al*. Edoxaban versus warfarin in patients with atrial fibrillation. *N. Engl. J. Med.* **369,** 2093–104 (2013).

21. Granger, C. & Alexander, J. Apixaban versus warfarin in patients with atrial fibrillation. *N. Engl. J. Med.* 981–992 (2011).

22. Patel, M., Mahaffey, K. & Garg, J. Rivaroxaban versus warfarin in nonvalvular atrial fibrillation. *N. Engl. J. Med.* 883–891 (2011).

23. Connolly, S. *et al*. Clopidogrel plus aspirin versus oral anticoagulation for atrial fibrillation in the Atrial fibrillation Clopidogrel Trial with Irbesartan for prevention of Vascular Events (ACTIVE W): a randomised controlled trial. *Lancet* **367,** 1903–12 (2006).

24. Ezekowitz, M. D. *et al*. Dabigatran with or without concomitant aspirin compared with warfarin alone in patients with nonvalvular atrial fibrillation (PETRO Study). *Am. J. Cardiol.* **100,** 1419–26 (2007).

For peer review only - http://bmjopen.bmj.com/site/about/guidelines.xhtml

25. Rash, A. *et al.* A randomised controlled trial of warfarin versus aspirin for stroke prevention in octogenarians with atrial fibrillation (WASPO). *Age Ageing* **36,** 151–6 (2007).

26. Chung, N. *et al.* Safety of edoxaban, an oral factor Xa inhibitor, in Asian patients with non-valvular atrial fibrillation. *Thromb. Haemost.* **105,** 535–44 (2011).

27. Yamashita, T. *et al.* Randomized, Multicenter, Warfarin-Controlled Phase II Study of Edoxaban in Japanese Patients With Non-Valvular Atrial Fibrillation. *Circ. J.* **76,** 1840–1847 (2012).

28. Connolly, S. & Eikelboom, J. Apixaban in patients with atrial fibrillation. *N. Engl. J. Med.* **364,** 806–817 (2011).

29. Connolly, S. J. *et al.* Effect of clopidogrel added to aspirin in patients with atrial fibrillation. *N. Engl. J. Med.* **360,** 2066–78 (2009).

30. Harenberg, J. & Weiss, C. Clinical trials with new oral anticoagulants. Additive value of indirect comparisons also named network meta-analyses. *Hamostaseologie* **33,** 62–70 (2013).

31. Dogliotti, A., Paolasso, E. & Giugliano, R. P. Current and new oral antithrombotics in non-valvular atrial fibrillation: a network meta-analysis of 79 808 patients. *Heart* (2013). doi:10.1136/heartjnl-2013-304347

32. Ruff, C. T. *et al.* Comparison of the efficacy and safety of new oral anticoagulants with warfarin in patients with atrial fi brillation : a meta-analysis of randomised trials. *Lancet* **6736,** 1–8 (2013).

33. Assiri, A., Al-Majzoub, O., Kanaan, *et al*. Mixed treatment comparison meta-analysis of aspirin, warfarin, and new anticoagulants for stroke prevention in patients with nonvalvular atrial fibrillation. *Clin. Ther.* **35,** 967–984.e2 (2013).

34. Slot, K. & Berge, E. Factor Xa inhibitors versus vitamin K antagonists for preventing cerebral or systemic embolism in patients with atrial fibrillation. *Stroke* (2013). at <http://stroke.ahajournals.org/content/44/12/e165.short>

35. Salanti, Georgia, Kavvoura, F, *et al*. Exploring the geometry of treatment networks. *Ann. Intern. Med.* **148,** 544–553 (2008).

36. Barratt, A. *et al.* Tips for learners of evidence-based medicine: 1. Relative risk reduction, absolute risk reduction and number needed to treat. *Can. Med. Assoc. J.* **171,** 353–8 (2004).

37. Schechtman, E. Odds ratio, relative risk, absolute risk reduction, and the number needed to treat--which of these should we use? *Value Health* **5,** 431–6 (2002).

For peer review only - http://bmjopen.bmj.com/site/about/guidelines.xhtml

38. Berger, M. L., Mamdani, M., Atkins, D. *et al*. Good research practices for comparative effectiveness research: defining, reporting and interpreting nonrandomized studies of treatment effects using secondary data sources: the ISPOR Good Research Practices for Retrospective Database Analysis Task Force . *Value Health* **12,** 1044–52 (2009).

39. Berger, M. L. *et al.* Prospective observational studies to assess comparative effectiveness: the ISPOR good research practices task force report. *Value Health* **15,** 217–30 (2012).

40. Jansen, J. P. Network meta-analysis of individual and aggregate level data. *Res. Synth. Methods* **3,** 177–190 (2012).

41. Salanti, G., Ades, A. & Ioannidis, J. Graphical methods and numerical summaries for presenting results from multiple-treatment meta-analysis: an overview and tutorial. *J. Clin. Epidemiol.* **64,** 163–71 (2011).

42. Guo, J. J. *et al.* A review of quantitative risk-benefit methodologies for assessing drug safety and efficacy-report of the ISPOR risk-benefit management working group. *Value Health* **13,** 657–66 (2010).

43. Boyd, C. *et al. Methods Research Report Methods for Benefit and Harm Assessment in Systematic Reviews*. 52 (2012). at <www.ncbi.nlm.nih.gov/books/NBK115750/>

44. Thorlund, K., Thabane, L. & Mills, E. J. Modelling heterogeneity variances in multiple treatment comparison meta-analysis – Are informative priors the better solution ? *BMC Med. Res. Methodol.* **13,** 1 (2013).

For peer review only

FIGURES

Figure 1: Evidence network for all cause stroke or systemic embolism.* The width of the lines is proportional to the number of randomized controlled trials comparing each pair of treatments, and the size of each treatment node is proportional to the number of randomised participants (sample size). A dotted line indicates a three-arm randomized controlled trial and a black node indicates a node included in the analysis but not reported in the main text.

For peer review only - http://bmjopen.bmj.com/site/about/guidelines.xhtml

ASA= acetylsalicylic acid; mg= milligram; RCT= RCT = randomized controlled trial; VKA= vitamin K antagonist
*Four RCTs (PETRO,[24] WASPO[25], Chung et al,[26] and Yamashita et al[27]) were not included in the analysis because they did not report data for this outcome or had zeroes in both arms

Figure 2: Odds ratio for all cause stroke or systemic embolism (A) and major bleeding (B) in Bayesian network meta-analysis versus standard adjusted dose VKA.

CrI= credible interval; mg= milligram; VKA= vitamin K antagonist

Figure 3: Odds Ratios (OR) from network meta-analyses for stroke or systemic embolism and major bleeding for all pair-wise comparisons.* ORs for recurrence of stroke or systemic embolism are below the diagonal (row-defining treatment versus column-defining treatment) and those for major bleeding are above the diagonal and in blue (column-defining treatment versus row-defining treatment). To obtain ORs for comparisons in the opposite direction, reciprocals should be taken (e.g., the OR for standard dose warfarin compared with apixaban 5 mg twice daily for stroke or systemic embolism is 1/0.78=1.28). Significant results are in bold and underscored (and in larger font).

mg= milligram; VKA= vitamin K antagonist
* Results between individual treatments, especially newer oral anticoagulants, should be interpreted with caution given the limitations associated with using a fixed-effects model. See Appendix 9 for additional detail

Figure 4: Icon array illustrating the absolute risks of Stroke or systemic embolism (blue) and major bleeding episodes (red) per 1000 patients treated*

* Figures do not reflect uncertainty around effect estimates and there is more uncertainty (i.e., wider credible intervals) for low dose ASA, medium dose ASA, and clopidogrel plus low dose ASA (See Appendix 8). Underlying studies may also double count haemorrhage stroke.

For peer review only

For peer review only - http://bmjopen.bmj.com/site/about/guidelines.xhtml

For peer review only

For peer review only - http://bmjopen.bmj.com/site/about/guidelines.xhtml

Systematic Review and Network Meta-analysis comparing Antithrombotic Agents for the Prevention of Stroke and Major Bleeding in Patients with Atrial Fibrillation

Chris Cameron, PhD Student[1,2], Doug Coyle, Professor[1], Trevor Richter, Manager of Clinical Research [3], Shannon Kelly, Research coordinator[2] , Kasandra Gauthier, Clinical Research Officer[3], Sabine Steiner, Associate Professor[4], Marc Carrier, Associate P~~p~~rofessor[6], Kathryn Coyle, Health Economist[5], Kristen Moulton, Clinical Research Officer[3], Annie Bai , Clinical Research Officer[3], Tammy Clifford, Adjunct Professor[1,7], George Wells, Professor[1,2]

[1] Department of ~~Community Medicine and~~ Epidemiology and Community Medicine, University of Ottawa, Ottawa, Canada
[2] University of Ottawa Heart Institute, Ottawa, Canada
[3] Canadian Agency for Drugs and Technologies in Health, Ottawa, Canada
[4] Medical University of Vienna, Vienna, Austria
[5] Applied Health Economics Research Unit, Ottawa, Canada
[6] Thrombosis Program, Division of Hematology, Department of Medicine, University of Ottawa, Ottawa, Canada
[7] Department of Pediatrics, University of Ottawa, Ottawa, Canada

For peer review only

Word count, abstract: 322~~0~~
Word count, body of essay: 3,987
Number of figures: 4
Number of tables: 1
Number of References: 44~~36~~
Supplementary Material: 14~~2~~

Corresponding author:
Dr. George Wells
Professor and Director, Cardiovascular Research Methods Centre
Department of Epidemiology and Community Medicine~~Community Medicine and Epidemiology~~, University of Ottawa
Ottawa Heart Institute
40 Ruskin Street, Ottawa, Ontario, Canada K1Y 4W7
Phone: (613) 798-5555, ext. 18640
Fax: (613) 761-4412
Email: gawells@ottawaheart.ca

Keywords: atrial fibrillation, antithrombotic, anticoagulants, stroke, bleeding

Abstract

Objective: To examine the comparative efficacy and safety of antithrombotic treatments (dabigatran, rivaroxaban, apixaban, edoxaban, and vitamin K antagonists (VKA) at a standard adjusted dose (target international normalised ratio 2.0-3.0), acetylsalicylic acid, acetylsalicylic acid and clopidogrel) for non-valvular atrial fibrillation and among sub-populations.

Design: Systematic review and network meta-analysis.

Data sources: A systematic literature search strategy was designed and carried out using MEDLINE, EMBASE, Cochrane Register of Controlled Trials, and grey literature including the websites of regulatory agencies and health technology assessment organizations for trials published in English from 1988 to January 2014.

Eligibility criteria for selecting studies: Randomized controlled trials were selected for inclusion if they were published in English, included at least one antithrombotic treatment and involved patients with non-valvular atrial fibrillation eligible to receive anticoagulant therapy.

Results: For stroke or systemic embolism, apixaban and dabigatran 150 mg twice daily were associated with reductions relative to standard adjusted dose VKA whereas low-dose ASA and the combination of clopidogrel plus low-dose ASA were associated with increases. Absolute risk reductions ranged from 6 fewer events per 1,000 patients treated for dabigatran 150 mg twice daily to 15 more events for clopidogrel plus ASA. For major bleeding, apixaban, dabigatran 110 mg twice daily, edoxaban 30 mg daily, and edoxaban 60 mg daily were associated with reductions compared to standard adjusted dose VKA. Absolute risk reductions with these agents ranged from 18 fewer per 1,000 patients treated each year for edoxaban 30 mg daily to 243 more for medium dose ASA.

Conclusions: Compared with standard adjusted dose VKA, new oral anticoagulants were associated with modest reductions in the absolute risk of stroke and major bleeding. People on antiplatelet drugs experienced more strokes compared with anticoagulant drugs without any reduction in bleeding risk. To fully elucidate the comparative benefits and harms of antithrombotic agents across the various sub-populations, rigorously conducted comparative studies or network meta-regression analyses of patient-level data are required.

(PROSPERO registry - CRD42012002721).

For peer review only - http://bmjopen.bmj.com/site/about/guidelines.xhtml

Article Summary

Strengths and limitations of this study

- This network meta-analysis includes data on edoxaban from the recently published ENGAGE AF-TIMI 48 trial
- The studies pooled in this meta-analysis include antiplatelet agents in addition to newer oral anticoagulants and standard adjusted dose vitamin K antagonists
- We present findings on both the relative and absolute scale, and graphically illustrate absolute risks using icon arrays
- We report detailed *a priori* sub-group analyses for stroke or systemic embolism and major bleeding using data derived from FDA Public Summary Documents
- Comparisons between antithrombotic treatments were largely comprised of single studies, albeit large studies
- There is notable heterogeneity and the small number of studies limits the analyses that can be conducted to account for heterogeneity in the absence of patient-level data

For peer review only

INTRODUCTION

Atrial fibrillation (AF) is a common cardiac arrhythmia associated with increased morbidity and mortality.[1,2] Patients with AF sustain an increased risk of arterial thromboembolism and stroke, associated with high recurrence and substantial debilitating impact. Therefore, antithrombotic strategies using anticoagulant drugs and antiplatelet agents are recommended for AF patients presenting with risk factors for stroke. Antithrombotic therapy is also associated with a risk of bleeding; therefore, the beneficial effects on stroke prevention should always be compared against a patient's risk of major bleeding.[1,2]

Existing guidelines recommend anticoagulant therapy for patients at intermediate or high risk of stroke.[1,2] Although standard adjusted dose vitamin K antagonists (VKA) (e.g., warfarin) has been the cornerstone treatment for reducing the risk of stroke or systemic embolism (SE) in this population, it is associated with several drawbacks.[2] which have prompted the development of newer oral anticoagulants such as direct thrombin (dabigatran) and Xa (e.g. apixaban, rivaroxaban, edoxaban) inhibitors. [1,2] While some of these newer treatment options have been demonstrated to be efficacious in preventing stroke or SE compared to standard adjusted dose VKA, the relative efficacy and risk of bleeding of newer oral anticoagulants, both among themselves and in comparison to antiplatelet agents, is not clear, especially in certain sub-populations of patients, e.g., $CHADS_2$ score <2 or ≥2; time in therapeutic range (TTR) < 66% or ≥ 66%; Age <75 or ≥ 75 years. Therefore, the objective of this paper was to compare antithrombotic agents for the prevention of stroke and major bleeding in patients with non-valvular AF and among sub-populations.

For peer review only

METHODS

Published literature was identified by searching MEDLINE, MEDLINE In-Process & Other Non-Indexed Citations, EMBASE, BIOSIS Previews, PubMed and the Cochrane Central Register of Controlled Trials were searched through the Ovid interface to identify English-language clinical articles published from 1988 to January 23, 2014. Regular alerts were also established. Websites of regulatory agencies, Canadian and major international health technology assessment agencies, clinical practice guidelines as well as The Cochrane Library (2012, Issue 6) and University of York Centre for Reviews and Dissemination databases were searched. Complete details of the electronic search strategy, including any limits used, are reported in Appendix 1. The protocol was published online and was registered in the PROSPERO International Prospective Register of Systematic Reviews (PROSPERO registry - CRD42012002721).

The population of interest was individuals with non-valvular AF requiring anticoagulation (including all risk levels and regardless of any comorbidities). The following treatments were included in the review: apixaban, dabigatran, edoxaban, rivaroxaban, standard adjusted dose VKA, acetylsalicylic acid (ASA), ASA and clopidogrel. Numerous outcomes were considered and results are reported elsewhere;[3] however, the focus of this publication is the outcomes of all-cause stroke or SE and major bleeding as defined by the International Society on Thrombosis and Haemostasis [ISTH] definition),[4] which were the primary efficacy and safety outcomes used in the newer oral anticoagulant studies.

For peer review only - http://bmjopen.bmj.com/site/about/guidelines.xhtml

Active and placebo-controlled randomized controlled trials (RCTs) were selected for inclusion if they were published in English, included at least one antithrombotic treatment under review (using pre-specified doses), reported data for any of the pre-specified outcomes related to patient safety or clinical efficacystroke/SE or major bleeding, and involved patients with non-valvular AF eligible to receive anticoagulant therapy, regardless the level of stroke risk. Trials that included patients with contraindication to anticoagulant treatment were excluded. VKA trials were included if the dose was adjusted to a target international normalised ratio (INR) 2.0-3.0. Any dose of ASA was considered for inclusion, but ASA dose was stratified in the analysis as low (≤ 100 mg daily), medium (>100 mg to ≤ 300 mg daily), or high (>300 mg daily). We only included new oral anticoagulants which had at least one large phase III study. Three reviewers independently extracted the data on baseline characteristics, intervention(s) evaluated, including dose, duration, and relevant co-medication and results for each included article, using a standardized template. All extracted data was checked for accuracy by three independent reviewers. Any disagreements in the assessment of these data were resolved through discussion until consensus was reached. Quality assessment of RCTs was also performed independently by two reviewers using a standardized table based on major items from the SIGN 50 instrument for internal validity.[5] The trial selection process is presented in a flowchart based on the Preferred Reporting Items for Systematic Reviews and Meta-Analyses (PRISMA)[6] statement (Appendix 2).

Bayesian network meta-analyses were conducted using WinBUGS software (MRC Biostatistics Unit, Cambridge, UK). A binomial likelihood model[7] which accounts for the use of multi-arm trials was used for the analyses given the datasets provided were dichotomous outcomes and

For peer review only - http://bmjopen.bmj.com/site/about/guidelines.xhtml

included multi-arm trials. Trials with zero cells in both arms or nodes where there were no events were excluded from the evidence networks because they do not contribute information or allow interpretable information.[7] Both fixed and random-effects network meta-analyses were conducted, although the fixed-effects model was chosen for the reference case analysis, as the evidence network was largely comprised of single study connections. We modeled point estimates and 95% credible intervals for odds ratios (OR) using Markov Chain Monte Carlo methods. The absolute risk difference per 1000 patients treated each year for each outcome was also calculated, based on the standard adjusted dose VKA arm of the Randomized Evaluation of Long-Term Anti-coagulation Therapy (RE-LY) trial.[8] The RE-LY trial[8] was selected because it contained data for both $CHADS_2<2$ and $CHADS_2\geq2$ and had detailed data available from the Food and Drug Administration (FDA) Public Summary Report.[9] We also assessed the probability that each treatment was the most efficacious regimen, the second best, the third best, and so on.[10] Vague or flat priors, such as $N(0, 100^2)$ were assigned for basic parameters throughout, although informative priors were considered.[11] Assessment of model fit was based on deviance information criterion (DIC) and comparison of residual deviance to number of unconstrained data points.[7,12] To ensure convergence was reached, trace plots and the Brooks-Gelman-Rubin statistic were assessed.[13] Three chains were fit in WinBUGS for each analysis, with at least 40,000 iterations, and a burn-in of at least 40,000 iterations. We also qualitatively compared the results from our network meta-analysis with direct pair-wise estimates. Frequentist pair-wise meta-analyses were conducted using R (R Language and Environment for Statistical Computing, Vienna, Austria) and package meta.[14]

For peer review only

For peer review only - http://bmjopen.bmj.com/site/about/guidelines.xhtml

A key assumption behind network meta-analysis is that the analysed network is consistent, i.e., there is no conflict between direct and indirect evidence.[15] To assess inconsistency, we compared deviance and DIC statistics in fitted consistency and inconsistency models.[15] We also plotted the posterior mean deviance of the individual data points in the inconsistency model against their posterior mean deviance in the consistency model to identify any loops in where inconsistency is present (Appendix 11).[15] Additionally, the results from our network meta-analysis were qualitatively compared with direct frequentist pair-wise estimates.

Network meta-analysis also requires that studies are sufficiently similar in order to pool their results.[16] Available study and patient characteristics were assessed to ensure similarity and to investigate the potential impact of heterogeneity on effect estimates. Clinical and methodological heterogeneity was identified in a number of areas. The issues identified were similar to those reported in previous systematic reviews of anticoagulant drugs (e.g., differences in TTR, $CHADS_2$ score).[17] Heterogeneity was assessed by conducting network meta-analysis with the following pre-specified subgroup data reported in the individual RCTs: TTR $< 66\%$ or $\geq 66\%$; $CHADS_2$ score <2 or ≥ 2; Age <75 or ≥ 75 years.[17] The data to conduct these sub-group analyses were derived from FDA Public Summary Documents when not available in the publications.[9,18,19] Secondary evidence networks were constructed for each of the sub-group analyses. The WinBUGS code[7] and data needed to replicate all analyses are available online.

This research was supported through a grant by the Canadian Institute of Health Research (CIHR) Drug Safety and Effectiveness Network and financial support from the Canadian Agency for Drugs and Technologies in Health (CADTH).

For peer review only

RESULTS

Study Characteristics of included studies

The systematic review included 16~~3~~ individual RCTs (reported in 32~~29~~ publications and FDA reports); all evaluated the efficacy and safety of antithrombotic agents: dabigatran (150 or 110 mg twice daily), rivaroxaban (20 mg daily), apixaban (5 mg twice daily), edoxaban (30 or 60 mg daily), standard adjusted dose VKA, ASA (low dose (< 100 mg daily); medium dose (100-300 mg daily), or low dose-ASA plus clopidogrel (75 mg daily) in patients with non-valvular AF. None of the included studies directly compared one new oral anticoagulant drug with another (Appendix 4). Of the 82~~1~~,396~~677~~ randomized patients included in the primary analysis~~this review~~, five large multicentre trials account for 78,296 patients (96%): ENGAGE AF-TIMI 48 (n=21,105),[20] ARISTOTLE (n=18,201),[21] RE-LY (n=18,113),[8] ROCKET-AF (n=14,264)[22] and ACTIVE-W (n=6706).[23] Most trials included stroke and SE as a primary efficacy outcome, while bleeding events were a frequent safety outcome. Follow-up ranged from 12 weeks to 3.5 years. All trials published results between 1989 and 2013. Mean $CHADS_2$ score was reported in eight ~~six~~ trials, encompassing the vast majority of patients included in the systematic review. Reported $CHADS_2$ scores were consistent with a high-risk population ($CHADS_2 \geq 2$), with patients from ROCKET-AF[22] showing the highest risk for stroke (mean $CHADS_2$ = 3.5).

Mean age across the included trials ranged from 62~~5~~ years to 83 years (Appendix 3). All trials included patients of both gender and had a higher proportion of male participants in most studies. However, the proportions of patients with a prior stroke or TIA varied substantially across the

For peer review only

For peer review only - http://bmjopen.bmj.com/site/about/guidelines.xhtml

included trials, ranging from 3% in JAST to 55% in ROCKET-AF.[22] The same observation can be made regarding the proportions of patients with other concomitant conditions, i.e. congestive heart failure, hypertension, diabetes, myocardial infarction, and prior vitamin K antagonist experience. TTR was reported in all but one trial that included a standard adjusted dose VKA treatment arm. Patient baseline characteristics are summarized in Appendix 3.

The studies were individually critically appraised and the details are reported elsewhere.[3] Overall, there was substantial variation in study quality. However, large multicentre trials comparing newer anticoagulants with standard adjusted dose VKA such as ENGAGE AF-TIMI 48 (n=21,105), ARISTOTLE (n = 18,201), RE-LY (n = 18,113), and ROCKET-AF (n = 14,264), which account for the vast majority of patients included in the systematic review, appear to be methodologically rigorous.

Stroke or Systemic Embolism

The evidence network (Figure 1) for the primary analysis was comprised of twelve eleven RCTs representing eight treatments in addition to placebo/observation (N =821,396677). Four Two RCTs (PETRO,[24] WASPO[25], Chung et al,[26] and Yamashita et al[27]) were not included in the analysis because they did not report data for this outcome or had zeroes in both arms..

Apixaban and dabigatran (150 mg twice daily) were associated with reductions in stroke or SE relative to standard adjusted dose VKA (Figure 2). The use of these two agents led to absolute risk reductions ranging from 4 to 6 fewer events per 1,000 patients treated each year. By contrast, low-dose ASA and the combination of clopidogrel plus low-dose ASA appeared to have

For peer review only

For peer review only - http://bmjopen.bmj.com/site/about/guidelines.xhtml

a higher risk of stroke or SE than standard adjusted dose VKA, leading to an increase in the number of stroke or SE ranging from 14 to 15 more events per 1,000 patients treated each year (Figure 4 and Appendix 6). No differences were detected between standard adjusted dose VKA and each of the following interventions: rivaroxaban, edoxaban (30 mg or 60 mg daily), dabigatran (110 mg twice daily), and medium-dose ASA (Figure 3).

The estimates of effect derived from the direct pair-wise comparisons aligned well with those obtained from the network meta-analysis in both direction and magnitude (Appendix 6). Furthermore, the posterior mean residual deviance (296.4181) is close to the number of unconstrained data points (2724), which is an indication of reasonable model fit. A number of sub-group analyses and alternative modeling strategies were conducted on the primary analysis, including adjusting for $CHADS_2$, TTR, and age (Table 1). We also conducted a sensitivity analysis where we included the AVERROES[28] and ACTIVE A[29] trials (Appendix 12). These studies were excluded from the main analysis because we required patients to be eligible for anticoagulant therapy, including treatment with a VKA. The results differed slightly from the reference case (Appendix 12). The primary analysis was conducted using a fixed-effects model because the network was comprised of single study connections; these results were also compared against those obtained using a random-effects model with vague and informative and vague priors on the standard deviation, and found to be similar in magnitude although wider credible intervals were observed (Appendix 9).

For peer review only

Major bleeding

The evidence network for the primary analysis for major bleeding was comprised of 15~~2~~ RCTs representing eight treatments in addition to placebo/observation (N =83~~1~~,015~~677~~). The evidence network for major bleeding is similar to the evidence network for stroke or SE (Figure 1) but includes one extra RCT (WASPO[25]) comparing medium dose ASA with standard adjusted dose VKA, and two extra RCTs (Chung et al,[26] and Yamashita et al[27]) comparing edoxaban with standard adjusted dose VKA (Appendix 5).

Apixaban, dabigatran 110 mg twice daily, edoxaban 30 mg daily, and -edoxaban 60 mg daily were associated with reductions in the risk of major bleeding compared with standard adjusted dose VKA (Figure 2). No differences for major bleeding were detected between standard adjusted dose VKA and each of the remaining interventions: rivaroxaban, dabigatran 150 mg twice daily, clopidogrel plus low-dose ASA, and all ASA dosages (Figure 2). The absolute risk difference of major bleeding relative to standard adjusted dose VKA ranged from 18 fewer to 24 more events per 1000 patients treated per year **(**Figure 4 and Appendix 7**)**. A summary of the results for the Bayesian and direct pair-wise meta-analyses is shown in Appendix 7, and Bayesian network meta-analysis results are represented graphically in Figure 2. Complete results from the Bayesian network meta-analysis for all possible comparisons are presented in Figure 3**.**

The estimates of effect derived from the direct pair-wise comparisons aligned well with those obtained from the network meta-analysis in both direction and magnitude (Appendix 7). Furthermore, the posterior mean residual deviance (38~~27~~.0~~1~~) is close to the number of unconstrained data points (35~~26~~), which is an indication of reasonable model fit. A number of sub-group analyses and alternative modeling strategies were conducted on the primary analysis (Table 1). We also conducted a sensitivity analysis where we included the AVERROES[28] and

For peer review only - http://bmjopen.bmj.com/site/about/guidelines.xhtml

ACTIVE A[29] trial (Appendix 12), as well as analyses using a random-effects model with vague and informative priors (Appendix 9).

Benefit-Harm Assessment – Stroke or systemic embolism versus major bleeding

Figure 3 summarises the results for all pair-wise comparisons derived from the Bayesian fixed-effects network meta-analysis (Appendix 9). Results relative to standard adjusted dose VKA have been discussed above. For pair-wise comparisons among newer oral anticoagulants for stroke or SE, dabigatran 150 mg twice daily was associated fewer events versus dabigatran 110 mg twice daily, rivaroxaban, edoxaban 30mg daily, and edoxaban 60 mg daily. Apixaban, and rivaroxaban were also associated with fewer events compared to edoxaban 30mg daily. There were no differences among low-dose ASA, medium-dose ASA, and clopidogrel plus low-dose ASA. However, low-dose ASA and the combination of clopidogrel plus low-dose ASA were associated with increases in stroke or systemic embolism compared to all newer oral anticoagulants. For major bleeding, apixaban, dabigatran 110 mg twice daily were associated with fewer events versus dabigatran 150 mg twice daily and rivaroxaban. Edoxaban 30 mg daily was associated with fewer events than other new oral anticoagulants, while edoxaban 60 mg daily was associated with fewer events compared with rivaroxaban and clopidogrel plus low-dose ASA. The risk of major bleeding for apixaban was lower compared to clopidogrel plus low-dose ASA. There were no differences associated with the ASA treatments, both for comparisons among themselves and compared to the anticoagulant treatments.

Figure 4 plots the absolute risk for stroke or SE and major bleeding per 1000 treated for the 10 treatments analyzed. Data in Figure 4 were obtained from the results presented in Appendix 6 and 7. Examination of the data in Figure 4 suggests that the balance of benefits and harms of the

For peer review only

For peer review only - http://bmjopen.bmj.com/site/about/guidelines.xhtml

new oral anticoagulants for stroke or systemic embolism and major bleeding is positive compared to standard adjusted dose VKA (decrease in stroke or systemic embolism and/or major bleeding) and largely similar among one another. Comparison of all anticoagulant treatment (including standard adjusted dose VKA) to ASA, or ASA in combination with clopidogrel indicates that whereas the new oral anticoagulants decrease the risk of both stroke or systemic embolism and major bleeding, ASA has a less favorable balance of benefits and harms, i.e., fails to minimize the risk of stroke or systemic embolism and/or major bleeding.

DISCUSSION

We identified 16~~3~~ RCTs comparing antithrombotic agents for the prevention of all cause stroke or systemic embolism and major bleeding in patients with AF. To the best of our knowledge, this is the most up-to-date systematic review and network meta-analysis[30–34] to synthesize the available benefits and harms on newer oral anticoagulants, standard adjusted dose VKA, and antiplatelet agents for the prevention of stroke and major bleeding in patients with non-valvular AF (Appendix 13). Harenberg et al[30] recently summarized the published indirect comparisons and network meta-analyses evaluating newer oral anticoagulants for AF and an earlier unpublished version of our analysis[17] was considered among the most comprehensive analyses at that time.[30] This network meta-analysis expands upon that unpublished report.[17] Overall, our findings align with those reported in previously published network meta-analyses;[30,31,33] however, unlike previous analyses,[30] we include recently published edoxaban data,[20] and also include antiplatelet agents. We also present detailed evidence networks[35] to illustrate the body of evidence for each outcome; report analysis using multiple statistical models (Appendix 9); report detailed *a priori* sub-group analyses for each outcome using data derived from FDA Public

For peer review only

For peer review only - http://bmjopen.bmj.com/site/about/guidelines.xhtml

Summary Documents;[9,18] present findings on both the relative and absolute scale,[36,37] and graphically illustrate results using icon arrays. Our network meta-analysis is also the only one published to date that was registered in PROSPERO, and also adheres to PRISMA reporting standards (Appendix 10). It also differs from a recently published traditional meta-analysis by Ruff et al[32] given we report comparison of newer oral anticoagulants versus each other, and also include comparisons with antiplatelet agents.

For stroke or systemic embolism, apixaban and dabigatran 150 mg twice daily were associated with reductions in stroke or systemic embolism relative to standard adjusted dose VKA. By contrast, low-dose ASA and the combination of clopidogrel plus low-dose ASA appeared were associated with increases in stroke or systemic embolism relative to standard adjusted dose VKA. No differences were detected between standard adjusted dose VKA and each of the following interventions: rivaroxaban, dabigatran 110 mg twice daily, edoxaban 30 and 60 mg daily, medium-dose ASA, and no treatment/placebo. The absolute risk reduction of stroke or systemic embolism relative to standard adjusted dose VKA ranged from 6 fewer to 15 more events per 1000 patients treated per year, with dabigatran 150 mg twice daily yielding the largest aboslute risk reduction and clopidogrel plus low-dose ASA the largest absolute risk increase.

For major bleeding, apixaban, dabigatran 110 mg twice daily, edoxaban 30 mg daily, and edoxaban 60 mg daily were associated with a decreased n increased -risk of major bleeding relative to standard adjusted dose VKA. No differences for major bleeding were detected between standard adjusted dose VKA and each of the remaining interventions: rivaroxaban, dabigatran 150 mg twice daily, clopidogrel plus low-dose ASA, and all ASA dosages. The

For peer review only - http://bmjopen.bmj.com/site/about/guidelines.xhtml

absolute risk difference of major bleeding relative to standard adjusted dose VKA ranged from 18 fewer to 24 more events per 1000 patients treated, with edoxaban 30 mg daily yielding the largest absolute risk reduction and medium dose ASA the largest absolute risk increase. Results of network meta-analyses reflect the relative efficacy and safety profile of antiplatelet and anticoagulant drugs as documented by large clinical trials. Thus, international guidelines[1,2] for prevention of stroke in patients with AF also recommended oral anticoagulation over antiplatelet therapy as the latter had only modest protective effects. With respect to the new oral anticoagulants none of the guidelines has given a recommendation to prefer one of the new oral anticoagulants over another.[1,2]

Network meta-analysis involves pooling of trials. We observed variability across studies included in the evidence network in terms of $CHADS_2$, age and TTR (Appendix 3 and 4). For example, patients using rivaroxaban had a higher risk of stroke because ROCKET-AF[22] included patients with $CHADS_2 \geq 2$. As such, we conducted sub-group analysis to adjust for differences in patient and study-level characteristics. We derived data for sub-group analyses from FDA Public Summary documents[9,18,19] which enhanced our ability to account for variability across studies (e.g., use intention to treat (ITT) population for rivaroxaban versus per-protocol as-treated population reported in publication[22]). Results from these sub-group analyses differed slightly from those reported in the primary analysis, indicating that there may be differences in the benefits and harms across sub-groups. This becomes more apparent when sub-group analyses are reported on the absolute scale.[3] It is unclear whether the absolute risk reductions associated with these differences translate into clinically meaningful benefits in practice, especially in sub-populations of patients with a lower baseline risk of stroke or systemic embolism. To fully

For peer review only

elucidate the comparative benefits and harms of antithrombotic agents across the various sub-populations, rigorously conducted comparative studies,[38,39] or network meta-regression analyses of patient-level data[40] are required.

There are several limitations with the analysis we employed. First, there is notable heterogeneity in patients and study characteristics (Appendix 3, 4, and 15). For example, there were differences in patients enrolled in the underlying studies and completion rates. However, the small number of studies limits the analyses that can be conducted to account for heterogeneity in the absence of patient-level data. Second, there is insufficient sub-group data available for other outcomes reported in the RCTs included. As a consequence, detailed analysis was limited to two outcomes – stroke and SE and major bleeding. Third, we reported the results from the fixed-effects model in the main text. We felt that this was appropriate as the nodes in evidence networks are connected largely by single studies. Effect estimates derived from the fixed-effects model aligned more closely with the direct estimates. Reporting results from the fixed-effects model in the main text likely biases results in favour of the new oral anticoagulants, as treatments that achieved statistically significant results in the primary RCTs retained statistically significant findings when using the fixed-effects model, but not always when using the random-effects model due to the prior[11] on the between study variance. Nevertheless, the results from the random-effects model have also been reported in Appendix 9, along with detailed discussion around the limitations in using the fixed-effects model. Fourth, the absolute risk reduction for the network meta-analysis was calculated using the event rate in the standard adjusted dose VKA arm of the RE-LY study. The RE-LY trial was selected because it contained data for both $CHADS_2 < 2$ and $CHADS_2 \geq 2$ and had detailed data made publicly available following an FDA

For peer review only

review of dabigatran. Use of a different study (e.g., ARISTOTLE) for baseline event rate data is unlikely to change the results substantially given that the event rates in standard adjusted dose VKA arms were similar across studies. Fifth, we present findings using graphics that have been largely applied in the network meta-analysis literature,[41] such as a table with odds ratios for all pair-wise comparisons. This method of presenting findings on the relative scale is problematic when you are conducting assessments to compare the balance of benefits and harms, which need to be standardized. As such, we also provided results in absolute terms. We report our findings using icon arrays, although it should be noted that these results do not account for the utility values based upon patient preferences for each of the outcomes,[42,43] nor do they reflect uncertainty although we report absolute reductions on the risk benefit plane reflecting uncertainty in Appendix 8. There are other methods available that can incorporate patient preferences for outcomes.[42,43] Further, estimates of benefit and harm with several of the therapies (e.g., rivaroxaban) come from one trial and thus such data are not particularly robust. Further research is needed comparing the balance of benefit and harms using other research methodologies, incorporating other relevant outcomes, patient preferences for each of the outcomes,[42,43] and additional studies when they become available. Finally, haemorrhagic stroke is also considered a major bleed in underlying studies. Accordingly, we may have double counted haemorrhage stroke in this analysis. Unfortunately, we are not able to account for this issue in all included studies. Nonetheless, it is important to note this limitation and highlight that this potentially biases results in favor of newer oral anticoagulants given these agents were associated with reductions in these events.

CONCLUSIONS

For peer review only

Compared with standard adjusted dose VKA, apixaban and dabigatran 150 mg twice daily were associated with reductions in the incidence of stroke or systemic embolism in patients with AF, whereas, low-dose ASA and the combination of clopidogrel plus low-dose ASA increased the risk relative to standard adjusted dose VKA at preventing stroke or systemic embolism. Apixaban, dabigatran 110 mg twice daily, edoxaban 30 mg daily, and edoxaban 60 mg daily and was associated with a reduced risk in major bleeding compared with standard adjusted dose VKA. Although the results of the current review revealed that there were differences in stroke or systemic embolism and major bleeding among antithrombotic agents in the management of patients with AF in relative terms, it is unclear whether the absolute risk reductions associated with these differences translate into clinically meaningful benefits in practice, especially in sub-populations of patients with a lower baseline risk of stroke or systemic embolism. Rigorously conducted comparative studies or network meta-regression analyses of patient-level data are required to fully elucidate the comparative benefits and harms of antithrombotic agents across the various sub-populations.

For peer review only - http://bmjopen.bmj.com/site/about/guidelines.xhtml

TABLES

Table 1: Sub-group and sensitivity analyses for stroke or systemic embolism and major bleeding relative to standard adjusted dose VKA, Odds ratios ± 95% Credible Intervals versus standard adjusted dose VKA

Treatment	Reference Case – Fixed Effect NMA	Subgroup analysis by CHADS₂ Score		Subgroup analysis by Age		Subgroup analysis by TTR	
		CHADS₂ < 2	CHADS₂ ≥ 2	Age < 75 years	Age ≥ 75 years	TTR < 66%	TTR ≥ 66%
**Stroke or Systemic Embolism**							
Apixaban 5 mg twice daily	**0.78 (0.65,0.94)0.79 (0.66,0.95)**	0.86 (0.57,1.28)	**0.78 (0.63,0.95)**	0.85 (0.67,1.07)	**0.71 (0.53,0.95)**	0.79 (0.62,1.0031)	0.79 (0.6059,1.05)
Dabigatran 110 mg twice daily	0.91 (0.74,1.12)0.91 (0.74,1.12)	1 (0.64,1.55)	0.89 (0.71,1.12)	0.93 (0.7,1.243)	0.88 (0.66,1.198)	0.91 (0.69,1.19)	0.9 (0.66,1.24)
Dabigatran 150 mg twice daily	**0.66 (0.53,0.82)0.66 (0.53,0.82)**	0.61 (0.37,1)	**0.66 (0.52,0.85)**	0.63 (0.46,0.87)	**0.66 (0.48,0.9)**	0.54 (0.39,0.73)	0.81 (0.58,1.11)
Rivaroxaban 20 mg daily	0.87 (0.74,1.03)0.88 (0.74,1.03)	NA	0.88 (0.74,1.04)	0.95 (0.75,1.2)	0.8 (0.63,1.032)	0.81 (0.65,1.01)	0.72 (0.47,1.14)
Low Dose ASA (≤ 100 mg daily)	**1.87 (1.26,2.8)1.87 (1.27,2.8)**	2.2 (1.18,4.25)	2.1 (0.91,5.15) *	NA	2.04 (1.332,3.2019)	NA	NA
Medium Dose ASA (> 100 mg and ≤ 300 mg daily)	1.35 (0.74,2.47)1.35 (0.75,2.46)	1.97 (0.63,6.73) **	NA	NA	NA	NA	NA
Clopidogrel 75 mg daily & Low dose Aspirin (≤ 100 mg daily)	**1.93 (1.42,2.65)**1.93 (1.42,2.65)	3.16 (1.39,8.22)	1.15 (0.84,1.57)	NA	NA	1.52 (0.96,2.45)	**2.4 (1.59,3.71)**
Edoxaban 30 mg daily	1.14 (0.98,1.32)1.14(0.99,1.33)	NA	1.14 (0.98,1.32)1.14(0.99,1.33)	1.16 (0.94,1.44)NA	1.13 (0.91,1.4) NA	1.01 (0.80,1.28) NA	1.24 (1.01,1.24) NA
Edoxaban 60 mg daily	0.87 (0.74,1.01)0.87(0.74,1.02)	NA	0.87 (0.74,1.01)0.87(0.74,1.02)	0.91 (0.73,1.14)NA	0.83 (0.66,1.04) NA	0.83 (0.65,1.07) NA	0.91 (0.73,1.13) NA
**Major Bleeding**							
Apixaban 5 mg twice daily	**0.69 (0.6,0.8)0.69 (0.6,0.8)**	**0.59 (0.44,0.78)**	**0.73 (0.62,0.87)**	**0.73 (0.6,0.89)**	**0.65 (0.52,0.801)**	**0.57 (0.45,0.71)**	**0.81 (0.67,0.98)**

Formatted Table

Formatted: Font: 8 pt

Formatted: Font: 8 pt

Formatted: Font: 8 pt

Formatted: Font: 8 pt

Formatted: Font: 8 pt

Formatted: Font: 8 pt

Formatted: Font: 8 pt

Formatted: Font: 8 pt

Formatted: Font: 8 pt

Formatted: Font: 8 pt

For peer review only

For peer review only - http://bmjopen.bmj.com/site/about/guidelines.xhtml

Treatment	Reference Case – Fixed Effect NMA	Subgroup analysis by $CHADS_2$ Score		Subgroup analysis by Age		Subgroup analysis by TTR	
		$CHADS_2 <$ 2	$CHADS_2 \geq 2$	Age < 75 years	Age ≥ 75 years	TTR < 66%	TTR ≥ 66%
**Dabigatran 110 mg twice daily**	**0.8 (0.69,0.93)**~~0.8 (0.69,0.93)~~	**0.65 (0.47,0.89)**	0.86 (0.73,1.02)	**0.62~~1~~ (0.49,0.77)**	1.02 (0.84,1.25)	0.74 (0.61,0.91)	0.86 (0.69,1.06)
**Dabigatran 150 mg twice daily**	0.93 (0.8,1.08)~~0.93 (0.8,1.08)~~	0.77 (0.57,1.04)	1.01 (0.86,1.19)	**0.7 (0.56,0.86)**	1.19 (0.98,1.45)	0.76 (0.62,0.94)	1.15 (0.94,1.4)
**Rivaroxaban 20 mg daily**	1.03 (0.89,1.18)~~1.03 (0.89,1.19)~~	NA	1.03 (0.89,1.19)	0.93 (0.76,1.13)	1.15 (0.93,1.41)	0.92 (0.77,1.1)	**1.3 (1.01,1.69)**
**Low Dose ASA (≤ 100 mg daily)**	1.05 (0.6,1.86)~~1.05 (0.6,1.86)~~	0.83 (0.42,1.64)	1.6 (0.55,5.02) *	NA	1.01 (0.57,1.78~~9~~)	NA	NA
**Medium Dose ASA (> 100 mg and ≤ 300 mg daily)**	1.79 (0.63,5.67)~~1.78 (0.62,5.59)~~	1.48 (0.11,19.07) **	NA	NA	NA	NA	NA
**Clopidogrel 75 mg daily & Low dose Aspirin (≤ 100 mg daily)**	1.1 (0.83,1.47) ~~1.1 (0.83,1.46)~~	1.51 (0.89,2.61)	0.97 (0.69,1.36) †	NA	NA	0.66 (0.42,1.02)	1.66 (1.12,2.47)
**Edoxaban 30 mg daily**	**0.46 (0.4,0.54)**~~0.47(0 .4,0.54)~~	NA	**0.46 (0.4,0.54)**~~0.47(0.4,0.54)~~	0.50 (0.41,0.61) ‡~~NA~~¶	0.51 (0.41-0.62) ‡~~NA~~¶	0.45 (0.35,0.58) ‡ ~~NA~~¶	0.51 (0.43,0.61) ‡¶ ~~NA~~¶
**Edoxaban 60 mg daily**	**0.79 (0.69,0.9)**~~0.78(0 .69,0.9)~~	NA	**0.79 (0.69,0.9)**~~0.78(0.69,0.9)~~	0.74 (0.62-0.90) ‡~~NA~~¶	0.82 (0.68-0.98) ‡~~NA~~¶	0.69 (0.55,0.87) ‡~~NA~~¶	0.83 (0.70,0.97) ‡¶ ~~NA~~¶

ASA= acetylsalicylic acid; mg= milligram; NA=not available; NMA= network meta-analysis; OR=odds ratio; TTR= time in therapeutic range; VKA=vitamin K antagonist

* Results may be slightly biased against LDASA. Derived from BAFTA study where $CHADS_2$ subgroup data stratified by $CHADS_2$ 1-2 (vs. 0-1) and $CHADS_2$ 3-6 (vs. 2-6).

** Results may be slightly biased against medium-dose ASA. Based on study level results from CAFA and JAST studies. Although these studies consisted primarily of low-risk populations, some patients may have $CHADS_2$ scores greater than 2.

† Estimated from subgroup study by Healey et al 2008

¶ Data provided from ENGAGE AF-TIMI 48[20] uses TTR greater than 60%

‡ Based on overall time period where overall time period was defined as first dose to the last dose plus 3 days. ~~Raw data (i.e., total number of patients and patients who experienced an event for each sub-group) were not reported in ENGAGE AF-TIMI 48~~[†]

Formatted Table

Formatted: Font: 8 pt

Formatted: Font: 8 pt

Formatted: Font: 8 pt

Formatted: Font: 8 pt

Formatted: Font: 8 pt

Formatted: Font: 8 pt

Formatted: Font: 8 pt

Formatted: Font: 8 pt

For peer review only

3

For peer review only

FIGURES

Figure 1: Evidence network for all cause stroke or systemic embolism.* The width of the lines is proportional to the number of randomized controlled trials comparing each pair of treatments, and the size of each treatment node is proportional to the number of randomised participants (sample size). A dotted line indicates a three-arm randomized controlled trial and a black node indicates a node included in the analysis but not reported in the main text.

For peer review only

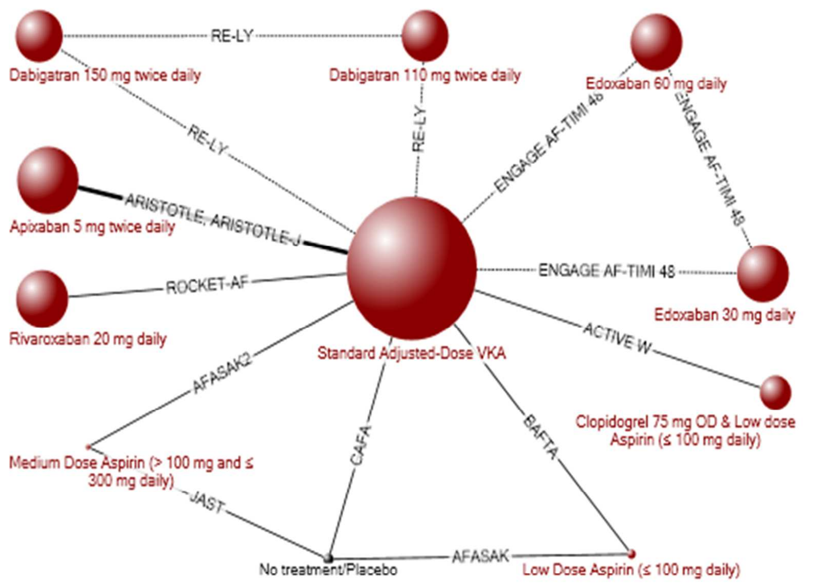

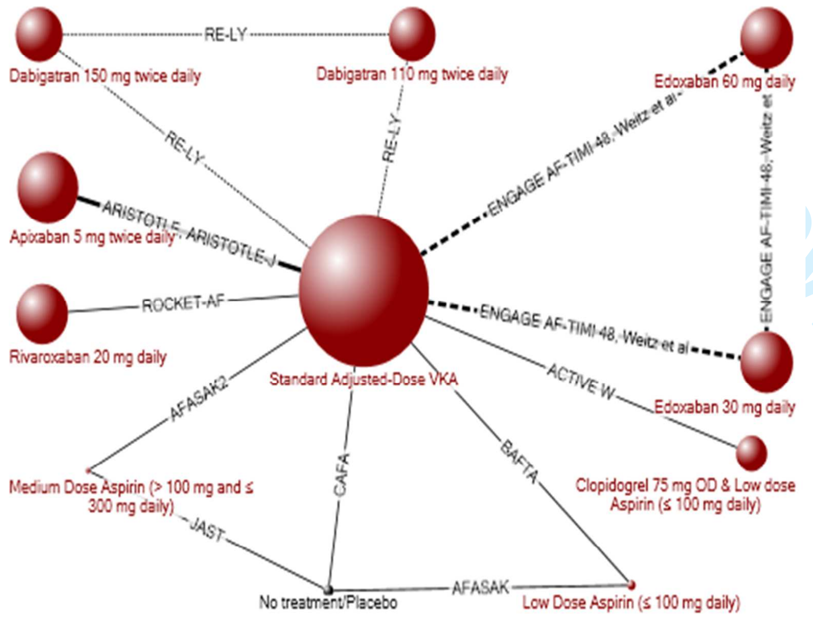

ASA= acetylsalicylic acid; mg= milligram; RCT= RCT = randomized controlled trial; VKA= vitamin K antagonist
*Four RCTs (PETRO,[24] WASPO[25], Chung et al,[26] and Yamashita et al[27]) were not included in the analysis because they did not report data for this outcome or had zeroes in both arms

5

Formatted: Font: (Default) Arial, 11 pt

Formatted: Font: 9 pt

Formatted: Font: (Default) Arial, 9 pt

Formatted: Font: (Default) Arial, 9 pt

Formatted: Font: (Default) Arial, 9 pt

Formatted: Font: (Default) Arial, 9 pt

Formatted: Font: (Default) Arial, 9 pt

Formatted: Font: (Default) Arial, 9 pt

Formatted: Font: (Default) Arial, 9 pt

Formatted: Font: (Default) Arial, 9 pt

Formatted: Font: (Default) Arial, 9 pt

Figure 2: Odds ratio for all cause stroke or systemic embolism (A) and major bleeding (B) in Bayesian network meta-analysis versus standard adjusted dose VKA.

Formatted: Font: (Default) Arial, 11 pt

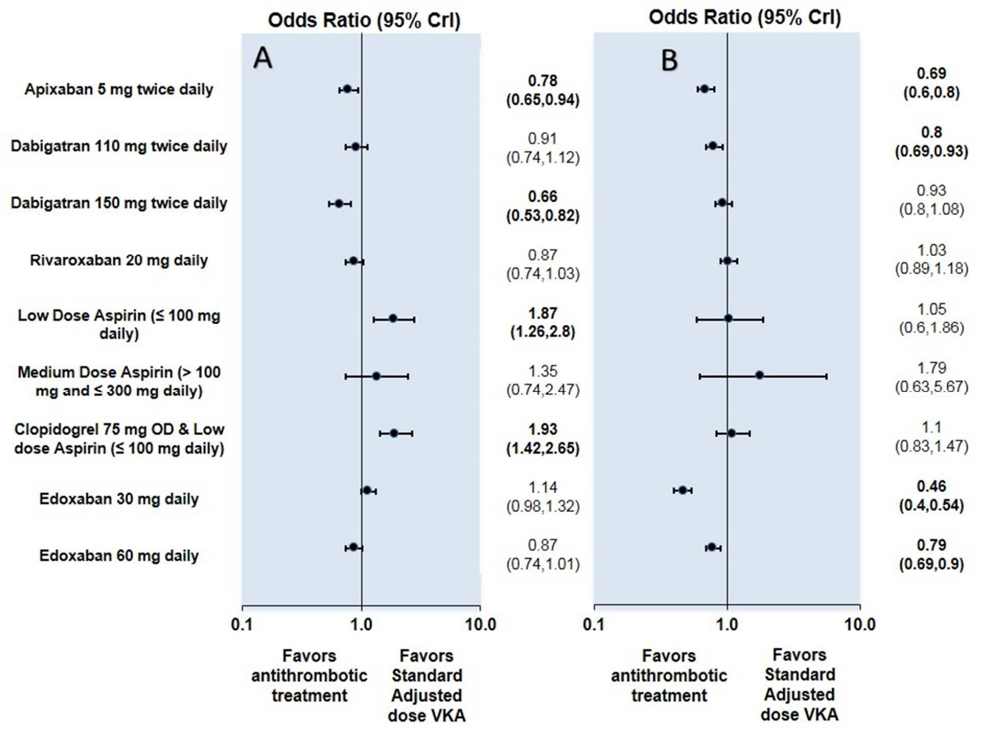

For peer review only - http://bmjopen.bmj.com/site/about/guidelines.xhtml

Formatted: Font: (Default) Arial, 11 pt

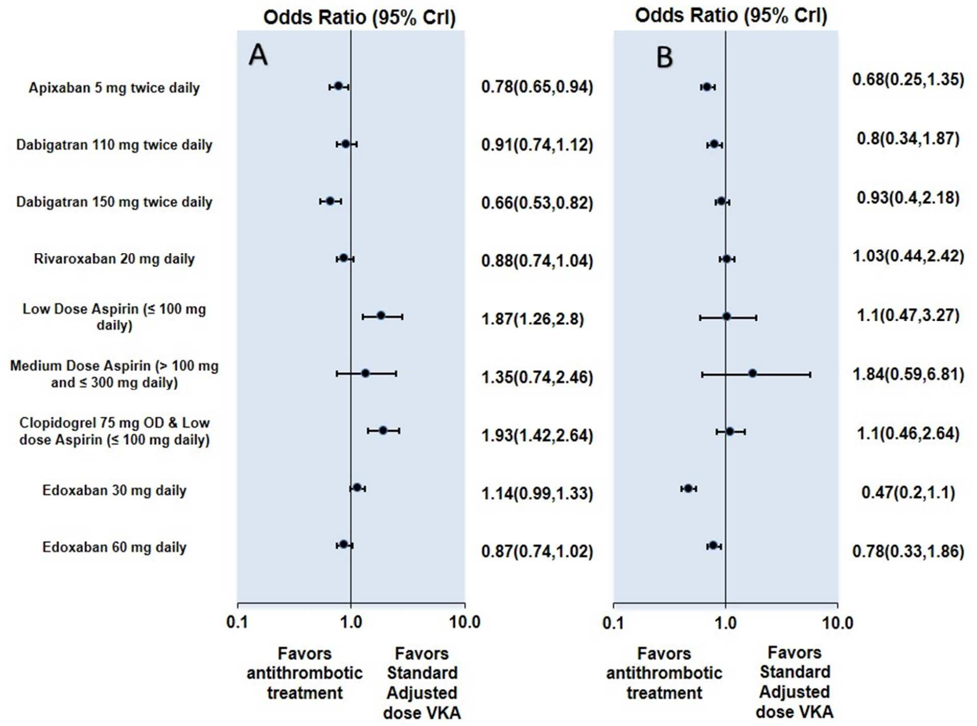

CrI= credible interval; mg= milligram; VKA= vitamin K antagonist

Figure 3: Odds Ratios (OR) from network meta-analyses for stroke or systemic embolism and major bleeding for all pair-wise comparisons.* ORs for recurrence of stroke or systemic embolism are below the diagonal (row-defining treatment versus column-defining treatment) and those for major bleeding are above the diagonal and in blue (column-defining treatment versus row-defining treatment). To obtain ORs for comparisons in the opposite direction, reciprocals should be taken (e.g., the OR for standard dose warfarin compared with apixaban 5 mg twice

For peer review only - http://bmjopen.bmj.com/site/about/guidelines.xhtml

daily for stroke or systemic embolism is 1/0.78~~9~~=1.28~~7~~). Significant results are in bold and underscored (and in larger font).

Standard Adjusted-Dose VKA	**0.69 (0.6,0.8)**	**0.8 (0.69,0.93)**	0.93 (0.8,1.08)	1.03 (0.89,1.18)	1.05 (0.6,1.86)	1.79 (0.63,5.[illegible]	[illegible]	[illegible]	**(0[illegible]**
**0.78 (0.65,0.94)**	Apixaban 5 mg twice daily	1.16 (0.94,1.43)	**1.35 (1.1,1.65)**	**1.48 (1.21,1.82)**	1.52 (0.85,2.74)	2.59 (0.9,8.2[illegible]	[illegible]	[illegible]	(0.[illegible]
0.91 (0.74,1.12)	1.17 (0.89,1.54)	Dabigatran 110 mg twice daily	1.16 (0.9976,1.36)	**1.28 (1.04,1.58)**	1.32 (0.73,2.37)	2.24 (0.77,7.[illegible]	[illegible]	[illegible]	(0[illegible]
**0.66 (0.53,0.82)**	0.84 (0.63,1.12)	**0.72 (0.57,0.91)**	Dabigatran 150 mg twice daily	1.1 (0.9,1.35)	1.13 (0.63,2.04)	1.93 (0.67,6.[illegible]	[illegible]	[illegible]	(0.[illegible]
0.87 (0.74,1.03)	1.12 (0.87,1.43)	0.96 (0.74,1.25)	**1.33 (1.01,1.75)**	Rivaroxaban 20 mg daily	1.03 (0.57,1.85)	1.75 (0.6,5.5[illegible]	[illegible]	[illegible]	**(0.[illegible]**
**1.87 (1.26,2.8)**	**2.4 (1.55,3.72)**	**2.05 (1.32,3.23)**	**2.85 (1.81,4.5)**	**2.14 (1.4,3.31)**	Low Dose Aspirin (≤ 100 mg daily)	1.7 (0.52,6.[illegible]	[illegible]	[illegible]	(0.[illegible]
1.35 (0.74,2.47)	1.72 (0.92,3.25)	1.48 (0.78,2.79)	**2.05 (1.08,3.9)**	1.54 (0.83,2.88)	0.72 (0.38,1.35)	Medium Dose Aspirin (> 100 mg and ≤[illegible] mg dai[illegible]	[illegible]	[illegible]	(0.[illegible]
**1.93 (1.42,2.65)**	**2.47 (1.73,3.55)**	**2.12 (1.46,3.08)**	**2.93 (2.01,4.31)**	**2.21 (1.56,3.15)**	1.03 (0.62,1.71)	1.43 (0.73,2.[illegible]	Clopidogrel 75 mg OD & Low dose [illegible]	[illegible]	**(0.[illegible]**
1.14 (0.98,1.32)	**1.45 (1.15,1.84)**	1.25 (0.97,1.61)	**1.73 (1.32,2.26)**	**1.3 (1.04,1.63)**	**0.61 (0.4,0.92)**	0.84 (0.45,1.[illegible]	[illegible]	[illegible]	(1.[illegible]
0.87 (0.74,1.01)	1.11 (0.87,1.41)	0.95 (0.73,1.23)	**1.31 (1,1.73)**	0.99 (0.79,1.25)	**0.46 (0.3,0.7)**	0.64 (0.34,1.[illegible]	[illegible]	[illegible]	[illegible]doxa[illegible]

Formatted: English (U.S.)

Formatted: Font: (Default) Arial, 9 pt, Bold, Font color: Black, English (U.S.)

Formatted: Font: (Default) Arial, 9 pt, Bold, Font color: Black, English (U.S.)

Formatted: Font: (Default) Arial, 9 pt, Font color: Black, English (U.S.)

Formatted: Font: (Default) Arial, 9 pt, Bold, Font color: Black, English (U.S.)

Formatted: Font: (Default) Arial, 9 pt, Font color: Black, English (U.S.)

Formatted: Font: (Default) Arial, 9 pt, Bold, Font color: Black, English (U.S.)

Formatted: Font: (Default) Arial, 9 pt, Font color: Black, English (U.S.)

Formatted: Font: (Default) Arial, 9 pt, Bold, Font color: Black, English (U.S.)

Formatted: Font: (Default) Arial, 9 pt, Font color: Black, English (U.S.)

Formatted: Font: (Default) Arial, 9 pt, Font color: Black, English (U.S.)

For peer review only - http://bmjopen.bmj.com/site/about/guidelines.xhtml

Standard Adjusted-Dose VKA	**0.69 (0.6,0.8)**	**0.8 (0.69,0.93)**	0.93 (0.8,1.08)	1.03 (0.89,1.18)	1.05 (0.6,1.86)	1.79 (0.63,5.67)	1.1 (0.83,1.47)	**0.46 (0.4,0.54)**	**0.79 (0.69,0.9)**
**0.78 (0.65,0.94)**	Apixaban 5 mg twice daily	1.16 (0.94,1.43)	**1.35 (1.1,1.65)**	**1.48 (1.21,1.82)**	1.52 (0.85,2.74)	2.59 (0.9,8.26)	**1.59 (1.15,2.19)**	**0.67 (0.54,0.83)**	1.13 (0.93,1.38)
0.91 (0.74,1.12)	1.17 (0.89,1.54)	Dabigatran 110 mg twice daily	1.16 (0.9976,1.36)	**1.28 (1.04,1.58)**	1.32 (0.73,2.37)	2.24 (0.77,7.16)	1.37 (0.99,1.9)	**0.58 (0.47,0.72)**	0.98 (0.8,1.2)
**0.66 (0.53,0.82)**	0.84 (0.63,1.12)	**0.72 (0.57,0.91)**	Dabigatran 150 mg twice daily	1.1 (0.9,1.35)	1.13 (0.63,2.04)	1.93 (0.67,6.15)	1.18 (0.85,1.63)	**0.5 (0.4,0.61)**	0.84 (0.69,1.03)
0.87 (0.74,1.03)	1.12 (0.87,1.43)	0.96 (0.74,1.25)	**1.33 (1.01,1.75)**	Rivaroxaban 20 mg daily	1.03 (0.57,1.85)	1.75 (0.6,5.56)	1.07 (0.78,1.48)	**0.45 (0.37,0.56)**	**0.77 (0.63,0.93)**
**1.87 (1.26,2.8)**	**2.4 (1.55,3.72)**	**2.05 (1.32,3.23)**	**2.85 (1.81,4.5)**	**2.14 (1.4,3.31)**	Low Dose Aspirin (≤ 100 mg daily)	1.7 (0.52,6.08)	1.05 (0.55,1.97)	**0.44 (0.24,0.79)**	0.75 (0.42,1.33)
1.35 (0.74,2.47)	1.72 (0.92,3.25)	1.48 (0.78,2.79)	**2.05 (1.08,3.9)**	1.54 (0.83,2.88)	0.72 (0.38,1.35)	Medium Dose Aspirin (> 100 mg and ≤ 300 mg daily)	0.61 (0.19,1.83)	**0.26 (0.08,0.75)**	0.44 (0.14,1.27)
**1.93 (1.42,2.65)**	**2.47 (1.73,3.55)**	**2.12 (1.46,3.08)**	**2.93 (2.01,4.31)**	**2.21 (1.56,3.15)**	1.03 (0.62,1.71)	1.43 (0.73,2.81)	Clopidogrel 75 mg OD & Low dose Aspirin (≤ 100 mg daily)	**0.42 (0.3,0.58)**	**0.71 (0.52,0.98)**
1.14 (0.98,1.32)	**1.45 (1.15,1.84)**	1.25 (0.97,1.61)	**1.73 (1.32,2.26)**	**1.3 (1.04,1.63)**	**0.61 (0.4,0.92)**	0.84 (0.45,1.56)	**0.59 (0.42,0.83)**	Edoxaban 30 mg daily	1.7 (1.45,1.99)
0.87 (0.74,1.01)	1.11 (0.87,1.41)	0.95 (0.73,1.23)	**1.31 (1,1.73)**	0.99 (0.79,1.25)	**0.46 (0.3,0.7)**	0.64 (0.34,1.19)	**0.45 (0.32,0.63)**	**0.76 (0.65,0.89)**	Edoxaban 60 mg daily

~~Standard Adjusted-Dose VKA~~	~~**0.69 (0.6,0.8)**~~	~~**0.8 (0.69,0.93)**~~	~~0.93 (0.81,1.08)~~	~~1.03 (0.89,1.19)~~	~~1.05 (0.59,1.86)~~	~~1.79 (0.62,5.64)~~	~~1.1 (0.83,1.47)~~	~~**0.47 (0.4,0.54)**~~	~~**0.78 (0.69,0.9)**~~
~~**0.78 (0.65,0.94)**~~	~~Apixaban 5 mg twice daily~~	~~1.16 (0.94,1.43)~~	~~**1.35 (1.1,1.65)**~~	~~**1.48 (1.21,1.82)**~~	~~1.52 (0.84,2.73)~~	~~2.59 (0.88,8.2)~~	~~**1.59 (1.15,2.19)**~~	~~**0.67 (0.54,0.83)**~~	~~1.13 (0.93,1.38)~~
~~0.91 (0.74,1.12)~~	~~1.17 (0.89,1.53)~~	~~Dabigatran 110 mg twice daily~~	~~1.16 (0.998,1.36)~~	~~**1.28 (1.04,1.58)**~~	~~1.31 (0.72,2.36)~~	~~2.23 (0.76,7.09)~~	~~1.38 (0.99,1.9)~~	~~**0.58 (0.47,0.72)**~~	~~0.98 (0.8,1.2)~~
~~**0.66 (0.53,0.82)**~~	~~0.84 (0.63,1.12)~~	~~**0.72 (0.57,0.9)**~~	~~Dabigatran 150 mg twice daily~~	~~1.1 (0.9,1.36)~~	~~1.13 (0.62,2.03)~~	~~1.92 (0.66,6.1)~~	~~1.18 (0.86,1.63)~~	~~**0.5 (0.4,0.62)**~~	~~0.84 (0.69,1.03)~~
~~0.88 (0.74,1.04)~~	~~1.12 (0.87,1.44)~~	~~0.96 (0.74,1.25)~~	~~**1.33 (1.01,1.76)**~~	~~Rivaroxaban 20 mg daily~~	~~1.03 (0.57,1.83)~~	~~1.74 (0.6,5.54)~~	~~1.07 (0.78,1.48)~~	~~**0.45 (0.37,0.56)**~~	~~**0.76 (0.63,0.93)**~~
~~**1.87 (1.26,2.8)**~~	~~**2.39 (1.55,3.72)**~~	~~**2.05 (1.31,3.22)**~~	~~**2.84 (1.8,4.5)**~~	~~**2.14 (1.39,3.3)**~~	~~Low Dose Aspirin (≤ 100 mg daily)~~	~~1.71 (0.51,6.03)~~	~~1.05 (0.55,1.99)~~	~~**0.44 (0.25,0.8)**~~	~~0.75 (0.42,1.34)~~

For peer review only - http://bmjopen.bmj.com/site/about/guidelines.xhtml

~~1.35 (0.74,2.46)~~	~~1.72 (0.92,3.24)~~	~~1.47 (0.78,2.79)~~	~~**2.04 (1.08,3.88)**~~	~~1.54 (0.83,2.88)~~	~~0.72 (0.38,1.35)~~	~~Medium Dose Aspirin (> 100 mg and ≤ 300 mg daily)~~	~~0.62 (0.19,1.85)~~	~~**0.26 (0.08,0.76)**~~	~~0.44 (0.14,1.28)~~
~~**1.93 (1.42,2.64)**~~	~~**2.47 (1.73,3.54)**~~	~~**2.11 (1.46,3.07)**~~	~~**2.93 (2.01,4.31)**~~	~~**2.2 (1.56,3.15)**~~	~~1.03 (0.62,1.71)~~	~~1.44 (0.73,2.82)~~	~~Clopidogrel 75 mg OD & Low dose Aspirin (≤ 100 mg daily)~~	~~**0.42 (0.3,0.58)**~~	~~**0.71 (0.52,0.98)**~~
~~1.14 (0.99,1.33)~~	~~**1.46 (1.16,1.85)**~~	~~1.25 (0.97,1.62)~~	~~**1.74 (1.33,2.28)**~~	~~**1.31 (1.04,1.64)**~~	~~**0.61 (0.4,0.93)**~~	~~0.85 (0.46,1.58)~~	~~**0.59 (0.42,0.83)**~~	~~Edoxaban 30 mg daily~~	~~1.69 (1.44,1.98)~~
~~0.87 (0.74,1.02)~~	~~1.12 (0.87,1.42)~~	~~0.95 (0.73,1.24)~~	~~**1.32 (1.01,1.74)**~~	~~1 (0.79,1.26)~~	~~**0.47 (0.3,0.71)**~~	~~0.65 (0.35,1.21)~~	~~**0.45 (0.32,0.64)**~~	~~**0.76 (0.65,0.89)**~~	~~Edoxaban 60 mg daily~~

mg= milligram; VKA= vitamin K antagonist

* Results between individual treatments, especially newer oral anticoagulants, should be interpreted with caution given the limitations associated with using a fixed-effects model. See Appendix 9~~7~~ for additional details.

For peer review only - http://bmjopen.bmj.com/site/about/guidelines.xhtml

Figure 4: Icon array illustrating the absolute risks of Stroke or systemic embolism (blue) and major bleeding episodes (red) per 1000 patients treated*

For peer review only

For peer review only - http://bmjopen.bmj.com/site/about/guidelines.xhtml

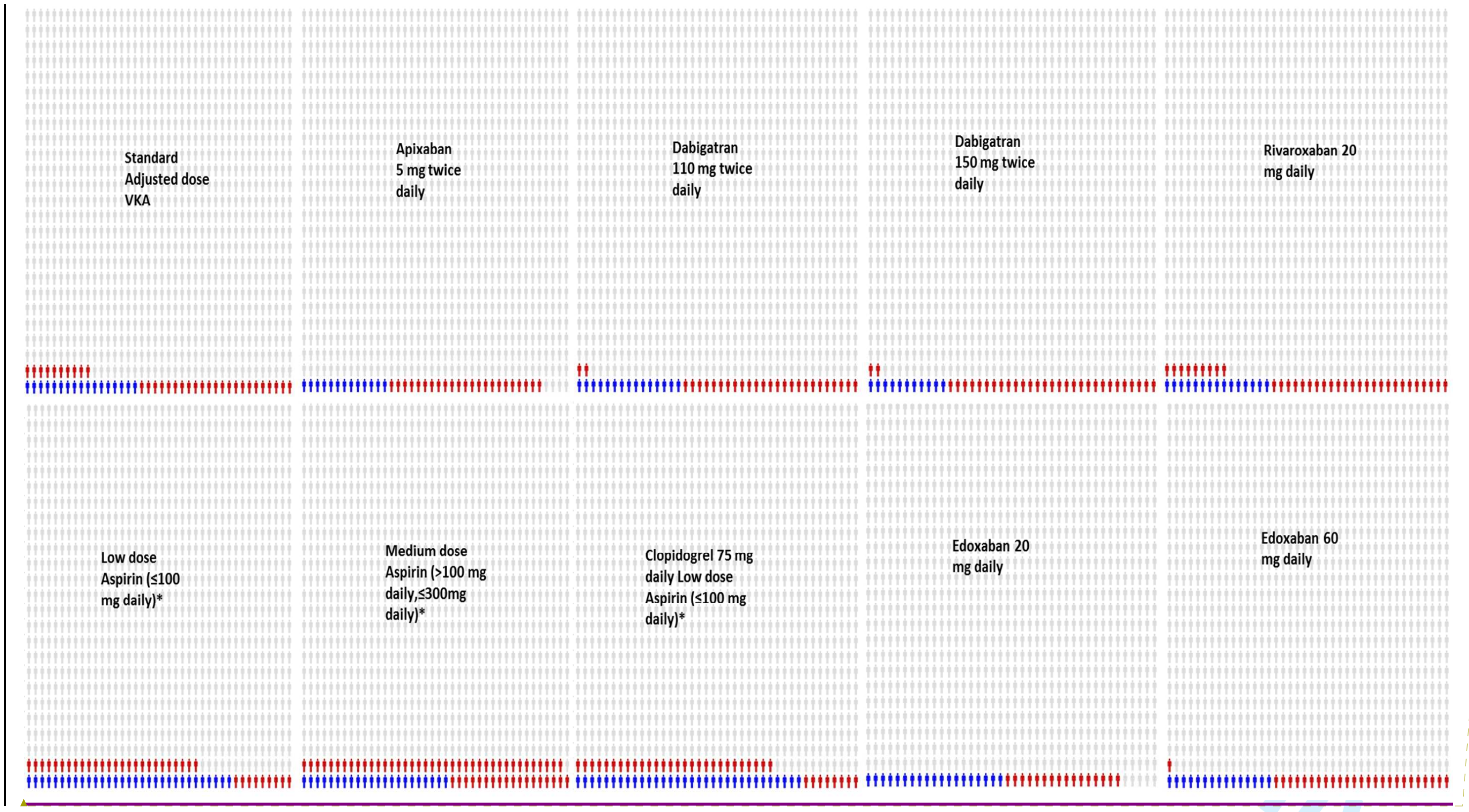

Formatted: Font: (Default) Arial, 11 pt, Bold

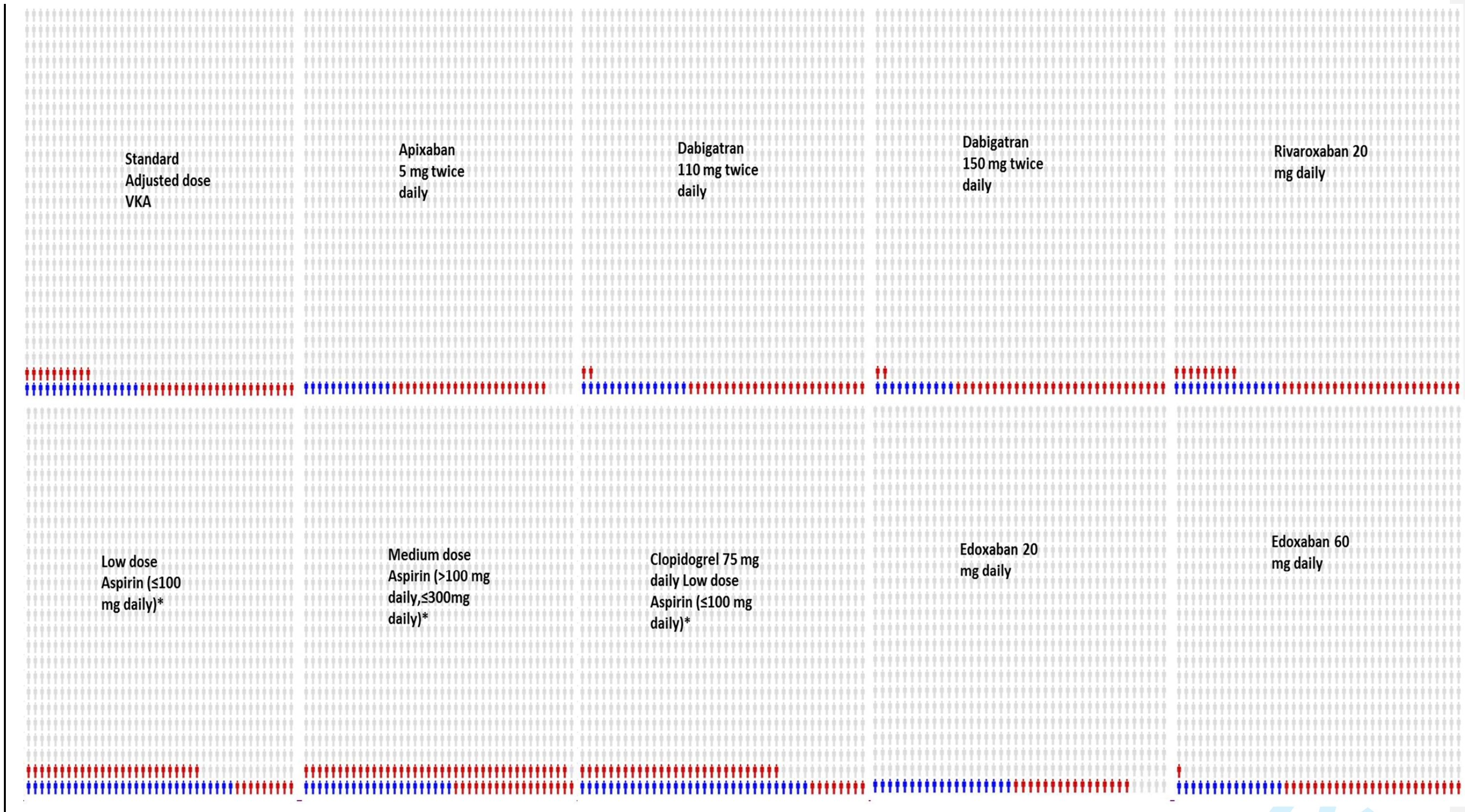

* Figures do not reflect uncertainty around effect estimates and there is more uncertainty (i.e., wider credible intervals) for low dose ASA, medium dose ASA, and clopidogrel plus low dose ASA (See Appendix 86). Underlying studies may also double count haemorrhage stroke.

Acknowledgments: The authors would like to acknowledge Carolyn Spry. She was the Information Specialist from CADTH on the review who conducted the search strategy. We would also like to thank Dr. Robert Giugliano for providing the sub-group data for edoxaban and his thoughtful comments on earlier drafts of the manuscript provided during the peer-review process.

Formatted: Font: (Default) Times New Roman, 11 pt

Contributors: CC conceived the study design, performed the data extraction, analysis, and drafted the manuscript. DC and GW conceived the study design, helped with the data analysis, and reviewed the manuscript for important intellectual content. TR conceived the study design, performed the systematic review, data extraction, and reviewed the manuscript for important intellectual content. SK, SS, KG, AB, KM, KC performed the systematic review, data extraction, and reviewed the manuscript for important intellectual content. TC, MC, and KC conceived the study and reviewed the manuscript for important intellectual content. All authors have read and approved the final version of the manuscript. All authors agree to be accountable for all aspects of the work in ensuring that questions related to the accuracy or integrity of any part of the work are appropriately investigated and resolved.

Funding information: This research was supported through a grant by the Canadian Institute of Health Research (CIHR) Drug Safety and Effectiveness Network and financial support from the Canadian Agency for Drugs and Technologies in Health (CADTH). The CIHR Drug Safety and Effectiveness Network played no role in study design, collection, analysis, interpretation of data, writing of the manuscript, or in the decision to submit the paper for publication. They accept no responsibility for the contents. Employees from CADTH were involved in the study design, collection, analysis, interpretation of data, and writing of the manuscript. CADTH, funded by Canadian federal, provincial and territorial governments, is an independent, not-for-profit producer and broker of health technology assessments. Federal, provincial, and territorial health care decision-makers rely on our evidence-based information to make informed decisions about the effectiveness and efficiency of drugs and other health technologies.

Competing interests: All authors have completed the ICMJE uniform disclosure form at www.icmje.org/coi_disclosure.pdf (available on request from the corresponding author) and declare: no support from any organisation for the submitted work; CC is a recipient of a Vanier Canada Graduate Scholarship from the Canadian Institutes of Health Research and has received funding from Canadian Network and Centre for Trials Internationally (CANNeCTIN) and is a trainee on the CIHR Drug Safety and Effectiveness Network team grant. MC is a recipient of a New Investigator Award from the Heart and Stroke Foundation of Canada and holds a T2 research chair in cancer and thrombosis from the University of Ottawa.

Ethical approval: Not required.

Data sharing: The statistical code and dataset used for analyses are reported online: http://www.cadth.ca/media/pdf/TR0003_AntithromboticAgents-AF_ScienceReport-e.pdf. Additional data can be requested by emailing George Wells: gawells@ottawaheart.ca .

The Corresponding Author has the right to grant on behalf of all authors and does grant on behalf of all authors, an exclusive licence (or non exclusive for government employees) on a worldwide basis to the BMJ Publishing Group Ltd to permit this article (if accepted) to be published in BMJ editions and any other BMJPGL products and sublicences such use and exploit all subsidiary rights, as set out in our licence

For peer review only - http://bmjopen.bmj.com/site/about/guidelines.xhtml

REFERENCES

1. Kirchhof, P. *et al.* Atrial fibrillation guidelines across the Atlantic: a comparison of the current recommendations of the European Society of Cardiology/European Heart Rhythm Association/European Association of Cardiothoracic Surgeons, the American College of Cardiology Fou. *Eur. Heart J.* **34,** 1471–1474 (2013).

2. Skanes, A. C. *et al.* Focused 2012 update of the Canadian Cardiovascular Society atrial fibrillation guidelines: recommendations for stroke prevention and rate/rhythm control. *Can. J. Cardiol.* **28,** 125–36 (2012).

3. *Antithrombotic agents for the prevention of stroke and systemic embolism in patients with atrial fibrillation*. (2013). at <http://www.cadth.ca/media/pdf/TR0003_AntithromboticAgents-AF_ScienceReport-e.pdf>

4. Schulman, S. & Kearon, C. Definition of major bleeding in clinical investigations of antihemostatic medicinal products in non-surgical patients. *J. Thromb. Haemost.* **3,** 692–4 (2005).

5. NHS Quality Improvement Scotland (NHS QIS). *SIGN 50. A guideline developer's handbook*. (2008). at <http://www.sign.ac.uk/pdf/sign50.pdf>

6. Moher, D., Liberati, A., Tetzlaff, J. & Altman, D. G. Preferred reporting items for systematic reviews and meta-analyses: the PRISMA statement. *PLoS Med.* **6,** e1000097 (2009).

7. Dias, S., Welton, N. J., Sutton, A. J. & Ades, A. *NICE DSU Technical Support Document 2: A Generalised Linear Modelling Framework for Pairwise and Network Meta-Analysis of Randomised Controlled Trials*. *Trop. Med.* 1–96 (2011). at <www.nicedsu.org.uk>

8. Connolly, S. & Ezekowitz, M. Dabigatran versus warfarin in patients with atrial fibrillation. *N. Engl. J. Med.* **361,** 1139–1151 (2009).

9. Ingelheim, B. *Advisory committee briefing document: dabigatran etexilate [Internet]*. 1–168 (2010). at <http://www.fda.gov/downloads/advisorycommittees/committeesmeetingmaterials/drugs/cardiovas>

10. Cipriani, A. *et al.* Comparative efficacy and acceptability of 12 new-generation antidepressants: a multiple-treatments meta-analysis. *Lancet* **373,** 746–58 (2009).

For peer review only

For peer review only - http://bmjopen.bmj.com/site/about/guidelines.xhtml

11. Turner, R. M., Davey, J., Clarke, M. J., Thompson, S. G. & Higgins, J. P. Predicting the extent of heterogeneity in meta-analysis, using empirical data from the Cochrane Database of Systematic Reviews. *Int. J. Epidemiol.* **41,** 818–27 (2012).

12. Spiegelhalter, D. J., Best, N. G., Carlin, B. P. & van der Linde, A. Bayesian measures of model complexity and fit. *J. R. Stat. Soc. Ser. B (Statistical Methodol.* **64,** 583–639 (2002).

13. Spiegelhalter, D., Thomas, A. & Best, N. WinBUGS user manual. *MRC Biostat. Unit* **2,** (2004).

14. meta: Meta-Analysis with R. at <http://cran.r-project.org/web/packages/meta/index.html>

15. Dias, S., Welton, N.J., Sutton, A.J., Caldwell, D.M., Guobing, L. & Ades, A. E. *NICE Technical Support Document 4: Inconsistency in Networks of Evidence Based on Randomised Controlled Trials*. 1–39 (2011). at <www.nicedsu.org.uk>

16. Salanti, G., Marinho, V. & Higgins, J. P. T. A case study of multiple-treatments meta-analysis demonstrates that covariates should be considered. *J. Clin. Epidemiol.* **62,** 857–64 (2009).

17. Wells, G., Coyle, D., Cameron, C., Steiner, S. & Kelly, S. *Safety, effectiveness, and costeffectiveness of new oral anticoagulants compared with warfarin in preventing stroke and other cardiovascular events in patients with atrial fibrillation*. (2012). at <http://www.cadth.ca/media/pdf/NOAC_Therapeutic_Review_final_report.pdf>

18. Johnson & Johnson Pharmaceutical Research & Development. *Rivaroxaban for the Prevention of Stroke and Non-Central Nervous System (CNS) Systemic Embolism in Patients With Atrial Fibrillation*. **7939,** 222 (2011).

19. McDonagh, M. S., Peterson, K., Balshem, H. & Helfand, M. US Food and Drug Administration documents can provide unpublished evidence relevant to systematic reviews. *J. Clin. Epidemiol.* (2013). doi:10.1016/j.jclinepi.2013.05.006

20. Giugliano, R. P. *et al.* Edoxaban versus warfarin in patients with atrial fibrillation. *N. Engl. J. Med.* **369,** 2093–104 (2013).

21. Granger, C. & Alexander, J. Apixaban versus warfarin in patients with atrial fibrillation. *N. Engl. J. Med.* 981–992 (2011).

22. Patel, M., Mahaffey, K. & Garg, J. Rivaroxaban versus warfarin in nonvalvular atrial fibrillation. *N. Engl. J. Med.* 883–891 (2011).

23. Connolly, S. *et al.* Clopidogrel plus aspirin versus oral anticoagulation for atrial fibrillation in the Atrial fibrillation Clopidogrel Trial with Irbesartan for prevention of

For peer review only

Vascular Events (ACTIVE W): a randomised controlled trial. *Lancet* **367,** 1903–12 (2006).

24. Ezekowitz, M. D. *et al.* Dabigatran with or without concomitant aspirin compared with warfarin alone in patients with nonvalvular atrial fibrillation (PETRO Study). *Am. J. Cardiol.* **100,** 1419–26 (2007).

25. Rash, A. *et al.* A randomised controlled trial of warfarin versus aspirin for stroke prevention in octogenarians with atrial fibrillation (WASPO). *Age Ageing* **36,** 151–6 (2007).

26. Chung, N. *et al.* Safety of edoxaban, an oral factor Xa inhibitor, in Asian patients with non-valvular atrial fibrillation. *Thromb. Haemost.* **105,** 535–44 (2011).

27. Yamashita, T. *et al.* Randomized, Multicenter, Warfarin-Controlled Phase II Study of Edoxaban in Japanese Patients With Non-Valvular Atrial Fibrillation. *Circ. J.* **76,** 1840–1847 (2012).

28. Connolly, S. & Eikelboom, J. Apixaban in patients with atrial fibrillation. *N. Engl. J. Med.* **364,** 806–817 (2011).

29. Connolly, S. J. *et al.* Effect of clopidogrel added to aspirin in patients with atrial fibrillation. *N. Engl. J. Med.* **360,** 2066–78 (2009).

30. Harenberg, J. & Weiss, C. Clinical trials with new oral anticoagulants. Additive value of indirect comparisons also named network meta-analyses. *Hamostaseologie* **33,** 62–70 (2013).

31. Dogliotti, A., Paolasso, E. & Giugliano, R. P. Current and new oral antithrombotics in non-valvular atrial fibrillation: a network meta-analysis of 79 808 patients. *Heart* (2013). doi:10.1136/heartjnl-2013-304347

32. Ruff, C. T. *et al.* Comparison of the efficacy and safety of new oral anticoagulants with warfarin in patients with atrial fi brillation□: a meta-analysis of randomised trials. *Lancet* **6736,** 1–8 (2013).

33. Assiri, A., Al-Majzoub, O., Kanaan, A. O., Donovan, J. L. & Silva, M. Mixed treatment comparison meta-analysis of aspirin, warfarin, and new anticoagulants for stroke prevention in patients with nonvalvular atrial fibrillation. *Clin. Ther.* **35,** 967–984.e2 (2013).

34. Slot, K. & Berge, E. Factor Xa inhibitors versus vitamin K antagonists for preventing cerebral or systemic embolism in patients with atrial fibrillation. *Stroke* (2013). at <http://stroke.ahajournals.org/content/44/12/e165.short>

For peer review only

35. Salanti, Georgia, Kavvoura, F, Ioannidis, J. Exploring the geometry of treatment networks. *Ann. Intern. Med.* **148,** 544–553 (2008).

36. Barratt, A. *et al.* Tips for learners of evidence-based medicine: 1. Relative risk reduction, absolute risk reduction and number needed to treat. *Can. Med. Assoc. J.* **171,** 353–8 (2004).

37. Schechtman, E. Odds ratio, relative risk, absolute risk reduction, and the number needed to treat--which of these should we use? *Value Health* **5,** 431–6 (2002).

38. Berger, M. L., Mamdani, M., Atkins, D. & Johnson, M. L. Good research practices for comparative effectiveness research: defining, reporting and interpreting nonrandomized studies of treatment effects using secondary data sources: the ISPOR Good Research Practices for Retrospective Database Analysis Task Force . *Value Health* **12,** 1044–52 (2009).

39. Berger, M. L. *et al.* Prospective observational studies to assess comparative effectiveness: the ISPOR good research practices task force report. *Value Health* **15,** 217–30 (2012).

40. Jansen, J. P. Network meta-analysis of individual and aggregate level data. *Res. Synth. Methods* **3,** 177–190 (2012).

41. Salanti, G., Ades, A. & Ioannidis, J. Graphical methods and numerical summaries for presenting results from multiple-treatment meta-analysis: an overview and tutorial. *J. Clin. Epidemiol.* **64,** 163–71 (2011).

42. Guo, J. J. *et al.* A review of quantitative risk-benefit methodologies for assessing drug safety and efficacy-report of the ISPOR risk-benefit management working group. *Value Health* **13,** 657–66 (2010).

43. Boyd, C. *et al. Methods Research Report Methods for Benefit and Harm Assessment in Systematic Reviews*. 52 (2012). at <www.ncbi.nlm.nih.gov/books/NBK115750/>

44. Thorlund, K., Thabane, L. & Mills, E. J. Modelling heterogeneity variances in multiple treatment comparison meta-analysis – Are informative priors the better solution□? *BMC Med. Res. Methodol.* **13,** 1 (2013).

For peer review only

For peer review only - http://bmjopen.bmj.com/site/about/guidelines.xhtml

SUPPLEMENTARY APPENDIX

Appendix 1: Search Strategy

**OVERVIEW**	
Interface:	Ovid
Databases:	EBM Reviews - Cochrane Central Register of Controlled Trials <May 2012><br>Ovid Embase 1974 to June 6, 2012<br>Ovid MEDLINE 1946 to June 7, 2012<br>Ovid MEDLINE Daily<br>Ovid MEDLINE In-Process & Other Non-Indexed Citations<br>**Note:** Subject headings have been customized for each database. Duplicates between databases were removed in Ovid.
Date of Search:	June 7, 2012; updated January 23, 2014
Alerts:	Monthly search updates began June 11, 2012 and ran until project completion
Study Types:	Randomized Controlled Trials
Limits:	Publication years 1988-present<br>Humans<br>Conference abstracts omitted

**SYNTAX GUIDE**	
/	At the end of a phrase, searches the phrase as a subject heading
.sh	At the end of a phrase, searches the phrase as a subject heading
MeSH	Medical Subject Heading
exp	Explode a subject heading
*	Before a word, indicates that the marked subject heading is a primary topic;<br>or, after a word, a truncation symbol (wildcard) to retrieve plurals or varying endings
#	Truncation symbol for one character
ADJ	Requires words are adjacent to each other (in any order)
ADJ#	Adjacency within # number of words (in any order)

For peer review only

For peer review only - http://bmjopen.bmj.com/site/about/guidelines.xhtml

.ti	Title
.ab	Abstract
.hw	Heading Word; usually includes subject headings and controlled vocabulary
.nm.	Name of substance word
.ot	Original title
.pt	Publication type
.rn	CAS registry number
use cctr	Select results from Cochrane database of Clinical Trials
use oemezd	Select results from Embase database
use pmez	Select results from Medline database

Clinical Multi-database Strategy

Line #	Search Strategy
1	(211914-51-1 or 211915-06-9).rn,nm. or (dabigatran* or Pradaxa or Pradax or Prazaxa or Praxada or bibr 1048 or bibr1048 or bibr953 or bibr 953 or rendix).ti,ot,ab,sh,rn,hw,nm.
2	(BMS 562247 or BMS562247 or apixaban* or Eliquis or Eliques).ti,ot,ab,sh,rn,hw,nm. or 503612-47-3.rn,nm.
3	(rivaroxaban* or Xarelto or BAY 59-7939 or BAY59-7939 or BAY597939 or BAY 597939).ti,ot,ab,sh,rn,hw,nm. or 366789-02-8.rn,nm.
4	(90055-48-4 or 113665-84-2).rn,nm. or (clopidogrel* or clopilet or grepid or iscover or plavix or zopya or zylagren or zyllt or duocover or duoplavin).ti,ot,ab,sh,rn,hw,nm.
5	Aspirin/ or 50-78-2.rn,nm. or (acetylsalicylic acid* or ASA* or acenterine or acesal or acetan or acetard or aceticil or aceticyl or acetilum or acetonyl or acetilum or acetonyl or acetophen or acetosal or acetosalicylic acid or acetosalin or acetosalum or acetyl salicylate or acetyl salicylic acid or acetylin or acetylo or acetylon or acetylosalicylic acid or acetylsal or acetysal or acetylsalicyclic acid or acetylsalicyl or acetylsalicylate or acetylsalycic acid or acetylsalycylic acid or oracidulatum or actorin or Acylpyrin or acylpyrine or acytosal or adiro or alabunkun or alasil or albyl-e or alka seltzer or alkASA or Aloxiprimum or anacin or anasprin or anopyrin or ansin or anthrom or aptor or arthralgyl or asaflow or ascription or orasaphen or asapor or asatard or asawin or aspec or aspent or aspergum or aspex or aspilets or aspirem or aspirgran or ASAa or ASAe or ASAine or aspirisucre or aspisol or aspo cid or aspro or asrina or asrivo or asta or asteric or astrix or bebesan or biprin or bokey or boxazin or breoprin or bufferin or cafenol or caprin or cASA or catalgine or catalgix or cemerit or cemirit or claradin or claragine or Colfarit or comoprin or contrheuma or daga or darosal or Dispril or dispirin or dolean or dusil or Easprin or ecasil or Ecotrin or egalgic or emocin or empirin or encaprin or encine em or Endosprin or entaprin or entercin or enteroprin or enterosarine or enterospirine or entrophen or eskotrin or euthermine or extren or genasprin or globentyl or godamed or gotosan or helicon or idotyl or infatabs a or istopirin or istopyrine or ivepirine or juvepirine or keypo or kilios or kinderASA or Magnecyl or measurin or mejoral or melabon or Micristin or micropyrin or mikristin or miniasal or mycrisitin or naspro or novasen or nu seal or nu seals or ortho acetoxybenzoate or ortho acetoxybenzoic acid or ortho acetyloxybenzoate or ortho acetyloxybenzoic acid or ostoprin or pancemol or paracin or paynocil or pengo or plewin or Polopirin or polopiryna or premaspin or primaspan or proprin or pyronoval or reumyl or rhodine or rhonal or ronal or salacetin or salisalido or sargepirine or soldral or solpyron or Solprin or solucetyl or solupsa or Solupsan or spren or super tru or tapal or temagin or tevapirin or th 2152 or thrombo-aspilets or treupahlin or treuphalin or tromalyt or tromcor or turiital or verin or vitalink or xaxa or Zorprin).ti,ot,ab,sh,rn,hw,nm.
6	Warfarin/ or 81-81-2.rn,nm. or (adoisine or aldocumar or athrombin* or befarin or brumolin or carfin or circuvit or compound 42 or coumadan or coumadin or coumadine or coumafen or coumafene or coumefene or coumaphene or coumarins or dagonal or farin or jantoven or kumatox or maforan or marevan or orfarin or panwarfarin or panwarfin or prothromadin or simarc 2 or sofarin or tedicumar or tintorane or waran or warfant or warfar or warfarin or warfarine or warfil or warfilone or warnerin).ti,ot,ab,sh,rn,hw,nm.
7	Acenocoumarol/ or 152-72-7.rn,nm. or (acenocoumarol* or nicoumalone or acenocoumarin or acenocoumarine or acenocoumarole or acenocoumarolum or acenocumarol or acenocumarolo or acenocumerol or acenkumarin or acitrom or ascumar or neo sintrom or neosintrom or neositron or nicoumalone or nicumalon or nitrovarfarian or nitrowarfarin or sincoumar or sincumar or sinkumar or sinthrom or sinthrome or sintrom or sintroma or sintron or syncoumar or syncumar or synthrom or syntrom or syntroma or sintron or syncoumar or syncumar or syntrom or trombostop or zotil or

For peer review only - http://bmjopen.bmj.com/site/about/guidelines.xhtml

	sinthrome or synthrom or syncoumar or syncumar or sinkumar or sintrom or minisintrom or trombostop or zotil).ti,ot,ab,sh,rn,hw,nm.
8	or/1-7
9	Atrial fibrillation/
10	((atrial or atrium or auricular or heart) adj1 fibrillation*).ti,ab.
11	(a-fib or afib).ti,ab.
12	9 or 10 or 11
13	8 and 12
14	13 use pmez
15	13 use cctr
16	14 or 15
17	*dabigatran/ or (dabigatran* or Pradaxa or Pradax or Prazaxa or praxada or bibr 1048 or bibr1048 or bibr953 or bibr 953 or rendix).ti,ab.
18	*apixaban/ or (BMS 562247 or BMS562247 or apixaban* or Eliquis or Eliques).ti,ab.
19	*rivaroxaban/ or (rivaroxaban* or Xarelto or BAY 59-7939 or BAY59-7939 or BAY597939 or BAY 597939).ti,ab.
20	*clopidogrel/ or *acetylsalicylic acid plus clopidogrel/ or (clopidogrel* or clopilet or grepid or iscover or plavix or zopya or zylagren or zyllt or duocover or duoplavin).ti,ab.
21	*warfarin/ or (adoisine or aldocumar or athrombin* or befarin or brumolin or carfin or circuvit or compound 42 or coumadan or coumadin or coumadine or coumafen or coumafene or coumefene or coumaphene or coumarins or dagonal or farin or jantoven or kumatox or maforan or marevan or orfarin or panwarfarin or panwarfin or prothromadin or simarc 2 or sofarin or tedicumar or tintorane or waran or warfant or warfar or warfarin* or warfarine or warfil or warfilone or warnerin).ti,ab.
22	*acetylsalicylic acid/ or (acetylsalicylic acid* or ASA* or acenterine or acesal or acetan or acetard or aceticil or aceticyl or acetilum or acetonyl or acetilum or acetonyl or acetophen or acetosal or acetosalicylic acid or acetosalin or acetosalum or acetyl salicylate or acetyl salicylic acid or acetylin or acetylo or acetylon or acetylosalicylic acid or acetylsal or acetysal or acetylsalicyclic acid or acetylsalicyl or acetylsalicylate or acetylsalycic acid or acetylsalycylic acid or oracidulatum or actorin or Acylpyrin or acylpyrine or acytosal or adiro or alabunkun or alasil or albyl-e or alka seltzer or alkASA or Aloxiprimum or anasprin or anopyrin or ansin or anthrom or aptor or arthralgyl or asaflow or orasaphen or asapor or asatard or asawin or aspec or aspent or aspergum or aspex or aspilets or aspirem or aspirgran or ASAa or ASAe or ASAine or aspirisucre or aspisol or aspo cid or aspro or asrina or asrivo or asta or asteric or astrix or bebesan or biprin or bokey or boxazin or breoprin or bufferin or cafenol or caprin or cASA or catalgine or catalgix or cemerit or cemirit or claradin or claragine or Colfarit or comoprin or contrheuma or daga or darosal or Dispril or dispirin or dolean or dusil or Easprin or ecasil or Ecotrin or egalgic or emocin or empirin or encaprin or encine em or Endosprin or entaprin or entercin or enteroprin or enterosarine or enterospirine or entrophen or eskotrin or euthermine or extren or genasprin or globentyl or godamed or gotosan or helicon or idotyl or infatabs a or istopirin or istopyrine or ivepirine or juvepirine or keypo or kilios or kinderASA or Magnecyl or measurin or mejoral or melabon or Micristin or micropyrin or mikristin or miniasal or mycrisitin or naspro or novasen or nu seal or nu seals or ortho acetoxybenzoate or ortho acetoxybenzoic acid or ortho acetyloxybenzoate or ortho acetyloxybenzoic acid or ostoprin or pancemol or paracin or paynocil or pengo or plewin or Polopirin or polopiryna or premaspin or primaspan or proprin or pyronoval or reumyl or rhodine or rhonal or ronal or salacetin or salisalido or sargepirine or soldral or solpyron or Solprin or solucetyl or solupsa or Solupsan or spren or super tru or tapal or temagin or tevapirin or th 2152 or thrombo-aspilets or treupahlin or treuphalin or tromalyt or tromcor or turiital or verin or vitalink or xaxa or Zorprin).ti,ab.
23	*Acenocoumarol/ or (acenocoumarol* or nicoumalone or acenocoumarin or acenocoumarine or acenocoumarole or acenocoumarolum or acenocumarol or acenocumarolo or acenocumerol or acenkumarin or acitrom or ascumar or neo sintrom or neosintrom or neositron or nicoumalone or nicumalon or nitrovarfarian or nitrowarfarin or sincoumar or sincumar or sinkumar or sinthrom or sinthrome or sintrom or sintroma or sintron or syncoumar or syncumar or synthrom or syntrom or syntroma or sintron or syncoumar or syncumar or syntrom or trombostop or zotil or sinthrome or synthrom or syncoumar or syncumar or sinkumar or sintrom or minisintrom or trombostop or zotil).ti,ab.
24	or/17-23
25	Heart atrium fibrillation/
26	((atrial or atrium or auricular or heart) adj1 fibrillation*).ti,ab.

For peer review only

27	(a-fib or afib).ti,ab.
28	25 or 26 or 27
29	24 and 28
30	29 use oemezd
31	30 not conference abstract.pt.
32	16 or 31
33	32
34	limit 33 to english language [Limit not valid in CCTR; records were retained]
35	(Randomized Controlled Trial or Controlled Clinical Trial).pt.
36	Randomized Controlled Trial/
37	Randomized Controlled Trials as Topic/
38	"Randomized Controlled Trial (topic)"/
39	Controlled Clinical Trial/
40	Controlled Clinical Trials as Topic/
41	"Controlled Clinical Trial (topic)"/
42	Randomization/
43	Random Allocation/
44	Double-Blind Method/
45	Double Blind Procedure/
46	Double-Blind Studies/
47	Single-Blind Method/
48	Single Blind Procedure/
49	Single-Blind Studies/
50	Placebos/
51	Placebo/
52	Control Groups/
53	Control Group/
54	(random* or sham or placebo*).ti,ab,hw.
55	((singl* or doubl*) adj (blind* or dumm* or mask*)).ti,ab,hw.
56	((tripl* or trebl*) adj (blind* or dumm* or mask*)).ti,ab,hw.
57	(control* adj3 (study or studies or trial*)).ti,ab.
58	(Nonrandom* or non random* or non-random* or quasi-random* or quasirandom*).ti,ab,hw.
59	allocated.ti,ab,hw.
60	((open label or open-label) adj5 (study or studies or trial*)).ti,ab,hw.
61	or/35-60
62	34 and 61
63	limit 62 to yr = "1988 -Current"
64	remove duplicates from 63

OTHER DATABASES	
PubMed	Same MeSH, keywords, limits, and study types used as per MEDLINE search, with appropriate syntax used.

Grey Literature

Dates for Search:	**June 12, 2012 – July 8, 2012; updated January 2014**
**Keywords:**	dabigatran, Pradax, Pradaxa, apixaban, Eliquis, rivaroxaban, Xarelto, clopidogrel, Plavix, acetylsalicylic acid, Aspirin, warfarin, Coumadin, acenocoumarol, Sintrom, Sinthrome<br>Atrial fibrillation

For peer review only

Limits:	Publication years 1988-present, English

Relevant items from the following sections of the CADTH grey literature checklist, "Grey matters: a practical tool for evidence-based searching" (http://www.cadth.ca/resources/grey-matters) were searched:

- Health Technology Assessment Agencies
- Health Economic
- Clinical Practice Guidelines
- Databases (free)

Internet Search

Appendix 2: PRISMA Diagram

For peer review only

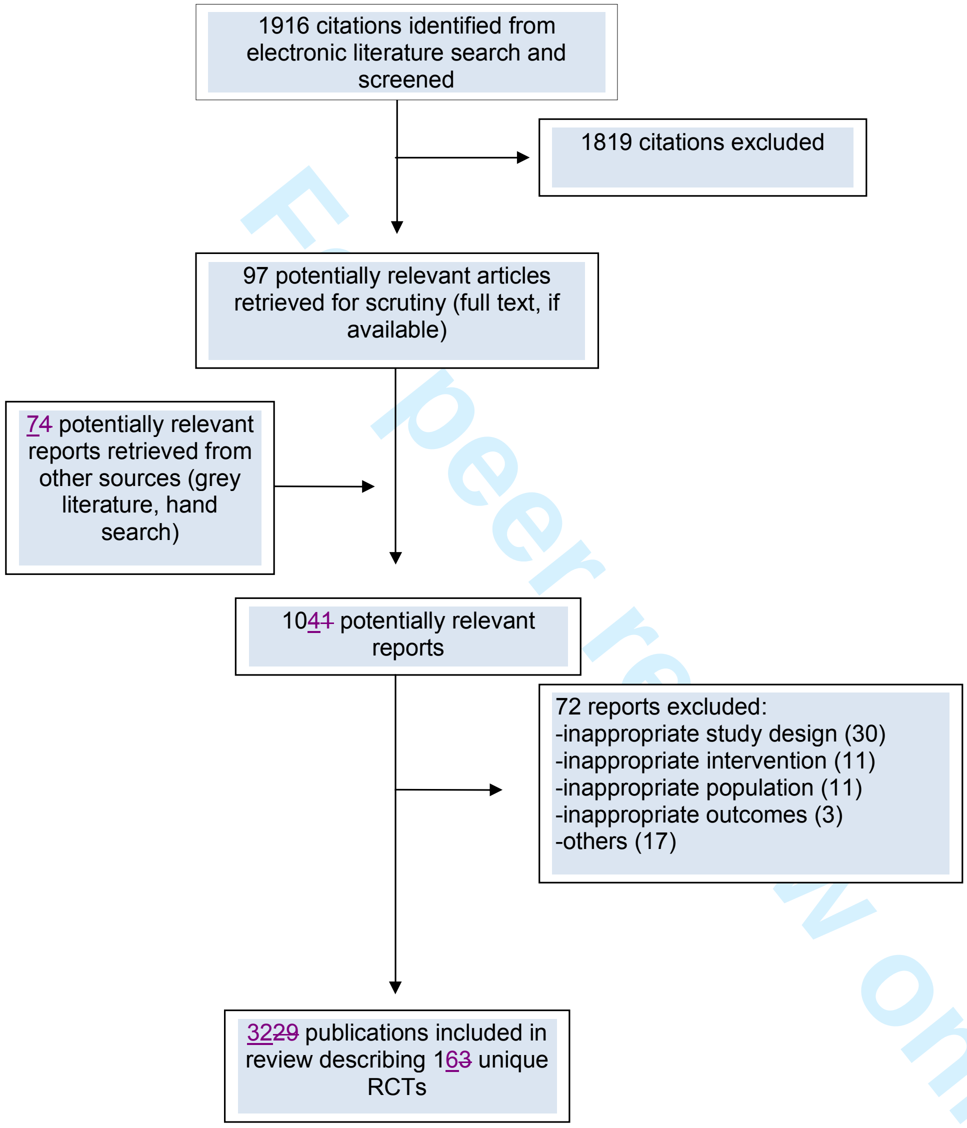

For peer review only - http://bmjopen.bmj.com/site/about/guidelines.xhtml

Appendix 3: Summary of Patient Baseline Characteristics

Study	Baseline Characteristics															Centre above TTR Throughout follow-up		
	Age, Mean ± SD (y)	Age Category (%)		Sex (%)		$CHADS_2$, Mean ± SD	$CHADS_2$ Category (%)			Prior Stroke /TIA (%)	DM (%)	Heart Failure (%)	HBP (%)	MI (%)	VKA Naïve (%)	Mean TTR (%)	TTR Category (%)	
		≤75y	>75 y	M	F		0	1	≥2								≤66%	>66%
ACTIVE-W n=6706	70 ± NR	NR	NR	66	34	2 ± 1.1	NR	NR	NR	15	21	30	82	17	23	64	48	52
AFASAK n=672	75 ± NR	NR	NR	54	46	NR ± NR	NR	NR	NR	6	9	53	32	7	NR	N/A	N/A	N/A
AFASAK-2 n=339	73 ± NR	NR	NR	61	39	NR ± NR	NR	NR	NR	8	12	70	45	7	NR	NR	NR	NR
ARISTOTLE n=18201	70 ± NR	69	31	65	35	2.1 ± 1.1	0	34	66	19	25	35	87	14	43	62	NR	NR
ARISTOTLE-J n=222	70 ± NR	69	31	83	17	1.9 ± NR	1	42	57	28	23	1	83	NR	16	60	NR	NR
BAFTA n=973	82 ± 4	0	100	55	45	NR ± NR	NR	NR	NR	13	13	20	54	11	NR	67	NR	NR
CAFA n=378	68 ± NR	NR	NR	75	25	NR ± NR	NR	NR	NR	4	12	22	39	13	NR	44	NR	NR
Chung et al 2011 n=234	65 ± 9	87	13	65	35	1.9 ± 1.1	0	47	53	24	30	2~~8~~9	71	NR	48	45	NR	NR
ENGAGE AF-TIMI 48 n=21105	72 ± NR	60	40	62	38	2.8 ± 1.0	0	0	100	28	36	57	94	12	41	65	52	48
JAST n=871	65 ± NR	NR	NR	70	30	NR ± NR	NR	NR	NR	3	14	9	39	NR	92	N/A	N/A	N/A
PETRO n=236	70 ± NR	70	30	82	18	NR ± NR	NR	NR	NR	18	25	32	71	NR	0	57	NR	NR
RE-LY n=18113	71 ± NR	60	40	64	36	2.1 ± NR	32		68	20	20	23	32	79	17	64	50	50
ROCKET-AF n=14264	73 ± NR	NR	NR	60	40	3.5 ± NR	0	0	100	55	40	62	91	17	38	55	NR	NR
WASPO n=75	83 ± NR	0	100	47	53	NR ± NR	NR	NR	NR	NR	4	NR	47	NR	NR	69	NR	NR
~~ENGAGE AF-TIMI 48 n=21105~~	~~72 ± NR~~	~~60~~	~~40~~	~~62~~	~~38~~	~~2.8 ± 1.0~~	~~0~~	~~0~~	~~100~~	~~28~~	~~36~~	~~57~~	~~94~~	~~12~~	~~41~~	~~65~~	~~52~~	~~48~~
Weitz et al 2010 n=719	65 ± 9	NR	NR	62	38	NR ± NR	0	0	100	NR	NR	NR	NR	NR	66	50	NR	NR

For peer review only - http://bmjopen.bmj.com/site/about/guidelines.xhtml

Study	Baseline Characteristics															Centre above TTR Throughout follow-up		
	Age, Mean ± SD (y)	Age Category (%)		Sex (%)		$CHADS_2$, Mean ± SD	$CHADS_2$ Category (%)			Prior Stroke /TIA (%)	DM (%)	Heart Failure (%)	HBP (%)	MI (%)	VKA Naïve (%)	Mean TTR (%)	TTR Category (%)	
		≤75y	>75 y	M	F		0	1	≥2								≤66%	>66%
Yamashita et al 2012 n=385	69 ± NR	71	29	83	17	2.10 ± NR	0		100	28	23	27	73	NR	15	73% FOR <70Y; 83% FOR ≥70Y;	NR	NR

DM = diabetes mellitus; F = female sex; HBP = high blood pressure; M = male sex; MI = myocardial infarction; N/A = not applicable; NR = not reported; SD = standard deviation; TIA = transient ischemic attack; TTR = time in therapeutic range; VKA = vitamin K antagonist; y = year.

Note: Only data from the included relevant treatment arms have been extracted.

For peer review only

For peer review only - http://bmjopen.bmj.com/site/about/guidelines.xhtml

Appendix 4: Summary of Included Trials

Study and Design	Disposition	Population	Interventions	Follow-up	Primary Outcome(s)
**ACTIVE-W**<br>OL RCT, 2006.<br>Multicenter, 32 countries (including Europe / America).	Randomized: n=6706<br>~~Completed: n=6026 (90%)~~	AF patients eligible / willing to take anticoagulant therapy, with ≥ 1 pre-specified risk factor for stroke.	**Clopidogrel / Low-dose ASA** (n=3335)<br>**Warfarin – INR 2.0 to 3.0** (n=3371)	1.3 years (stopped early)	First occurrence of stroke, non-CNS SE, MI or vascular death.
**AFASAK**<br>OL/DB RCT, 1989.<br>Multicenter, Copenhagen.	Randomized: n=1007<br>~~Completed: n=785 (80%)~~	AF patients, with no recent anticoagulation or cerebrovascular event, valve replacement or rheumatic heart disease.	***OL Warfarin – INR 2.8 to 4.2*** ** (n=335)*<br>**Low-dose ASA** (n=336)<br>**PL** (n=336)	2 years	Thromboembolic complication: stroke, TIA or embolic complication to viscera / extremities.
**AFASAK-2**<br>OL RCT, 1998.<br>Multicenter, Copenhagen.	Randomized: n=677<br>~~Completed: n=507 (75%)~~	AF patients ≥ 60 years (unless with CV or pulmonary disease), without recent TIA or risk factors for bleeding.	***Fixed-dose Warfarin*** ** (n=167)*<br>***Fixed-dose Warfarin / Medium-dose ASA*** ** (n=171)*<br>**Medium Dose ASA** (n=169)<br>**Warfarin – INR 2.0 to 3.0** (n=170)	3.5 years	Stroke or SE.
**ARISTOTLE**<br>DB RCT, 2011.<br>Multicenter, 39 countries (including Europe / America).	Randomized: n=18201<br>~~Completed: n=13468 (74%)~~	AF patients with ≥ 1 risk factor for stroke, without recent stroke or ASA > 165 mg.	**Apixaban 5 mg BID** (n=9120)<br>**Warfarin – INR 2.0 to 3.0** (n=9081)<br>Concomitant use of **ASA** (≤ 165 mg daily) or **NSAIDs** permitted.	NR	Stroke or SE;<br>Major bleeding.
**ARISTOTLE-J**<br>OL/DB RCT, 2011.<br>Multicenter, Japan.	Randomized: n=222<br>~~Completed: n=197 (89%)~~	AF patients with ≥ 1 risk factor for stroke, without recent TIA or ASA > 100 mg.	**Apixaban 2.5 mg BID** (n=74)<br>**Apixaban 5 mg BID** (n=74)<br>**OL Warfarin – INR 2.0 to 3.0** (n=74)<br>Concomitant use of **ASA** (≤ 100 mg daily) permitted.	12 weeks	Composite of major bleeding and CRNM bleeding events.
**BAFTA**<br>OL RCT, 2007.<br>Multicenter, UK.	Randomized: n=973<br>~~Completed: n=694 (72%)~~	AF patients ≥ 75 years, with clinical uncertainty around optimal treatment.	**Low-dose ASA** (n=485)<br>**Warfarin – INR 2.0 to 3.0** (n=488)	2.7 years	Disabling stroke, ICH and other clinically significant arterial embolism.
**CAFA**<br>DB RCT, 1991.<br>Multicenter, Canada.	Randomized: n=378<br>~~Completed: n=286 (76%)~~	AF patients without recent stroke, TIA or MI, and who did not require antiplatelet drug therapy.	**Warfarin – INR 2.0 to 3.0** (n=187)<br>**PL** (n=191)	1.2 years (stopped early)	Ischemic stroke, SE, ICH or fatal hemorrhage.

For peer review only - http://bmjopen.bmj.com/site/about/guidelines.xhtml

Study and Design	Disposition	Population	Interventions	Follow-up	Primary Outcome(s)
**Chung et al 2011** OL RCT, 2011. Multicenter, 4 countries (including Asia).	Randomized: n=234	AF patients at moderate to high risk of stroke (CHADS2 score ≥ 1).	**Edoxaban 30 mg OD** (n=79) **Edoxaban 60 mg OD** (n=80) **Warfarin – INR 2.0 to 3.0** (n=75)	12 weeks	All bleeding events
**ENGAGE AF-TIMI 48** DB RCT, 2013. Multicenter, 46 countries (including Europe / America).	Randomized: n=21105	AF patients at moderate to high risk of stroke (CHADS2 score ≥ 2).	**Edoxaban 30 mg OD** (n=7034) **Edoxaban 60 mg OD** (n=7035) **Warfarin – INR 2.0 to 3.0** (n=7036) Almost a third of patients in treatment arms took concomitant ASA.	2.8 years	Stroke or SE and major haemorrhage.
**JAST** OL RCT, 2006. Multicenter, Japan.	Randomized: n=871 ~~Completed: n=686 (79%)~~	AF patients without recent symptomatic thromboembolic disease, ICH or GI bleeding.	**Medium-dose ASA** (n=426) **No treatment** (n=445)	2.1 years (stopped early)	CV death, symptomatic brain infarction, or TIA.
**PETRO** OL/DB RCT, 2007. Multicenter, 4 countries (Europe and USA).	Randomized: n=502 ~~Completed: n=464 (92%)~~	AF patients with ≥ 1 risk factor for stroke and with coronary artery disease (removed halfway to facilitate recruitment). No recent MI, stroke, TIA or major bleed. Warfarin pre-treatment required for ≥ 8 weeks.	***Dabigatran 50 mg BID ± Low-dose or High-dose ASA*** ** (n=105)* ***Dabigatran 300 mg BID ± Low-dose or High-dose ASA*** ** (n=161)* **Dabigatran 150 mg BID ± Low-dose or High-dose ASA** † (n=166) **OL Warfarin – INR 2.0 to 3.0** (n=70)	12 weeks	Thromboembolic events and bleeding events.
**RE-LY** OL/DB RCT, 2009. Multicenter, 44 countries (including Europe / America).	Randomized: n=18113 ~~Completed: n=14580 (80%)~~	AF patients with ≥ 1 risk factor for stroke, without recent stroke or increased risk of bleeding.	**Dabigatran 110 mg BID** (n=6015) **Dabigatran 150 mg BID** (n=6076) **OL Warfarin – INR 2.0 to 3.0** (n=6022) Concomitant use of **ASA** (<100 mg daily) or other **antiplatelet agents** permitted.	2 years	Stroke or SE and major haemorrhage.
**ROCKET-AF** DB RCT, 2011. Multicenter, 45 countries (including Europe / America).	Randomized: n=14264 ~~Completed: n=10957 (77%)~~	AF patients at moderate to high risk of stroke (CHADS2 score ≥ 2).	**Rivaroxaban 20 mg OD** (n=7131) **Warfarin – INR 2.0 to 3.0** (n=7133) Over a third of patients in both arms took concomitant ASA.	1.9 years	Composite of stroke and SE; Composite of major and NMCR bleeding.

For peer review only - http://bmjopen.bmj.com/site/about/guidelines.xhtml

Study and Design	Disposition	Population	Interventions	Follow-up	Primary Outcome(s)
**WASPO** OL RCT, 2007. Multicenter, UK.	Randomized: n=75 ~~Completed: n=64 (85%)~~	Ambulant AF patients > 80 and < 90 years with Folstein score > 25, without previous fall, ICH or GI bleeding. Patients with prior stroke or TIA were excluded.	**Warfarin – INR 2.0 to 3.0** (n=36) **Medium-dose ASA** (n=39)	1 year	Combined endpoints: death; stroke, TIA or SE; serious bleeding; withdrawal.
~~**ENGAGE AF-TIMI 48** DB RCT, 2013. Multicenter, 46 countries (including Europe / America).~~	~~Randomized: n=21105 Completed: n=18635 (88%)~~	~~AF patients at moderate to high risk of stroke (CHADS2 score ≥ 2).~~	~~**Edoxaban 30 mg OD** (n=7034) **Edoxaban 60 mg OD** (n=7035) **Warfarin – INR 2.0 to 3.0** (n=7036) Almost a third of patients in treatment arms took concomitant ASA.~~	~~2.8 years~~	~~Stroke or SE and major haemorrhage.~~
**Weitz et al 2010** DB RCT, 2010. Multicenter, 12 countries (including Eastern Europe / America).	Randomized: n=719	AF patients at moderate to high risk of stroke (CHADS2 score ≥ 2).	**Edoxaban 30 mg OD** (n=235) **Edoxaban 60 mg OD** (n=234) **Warfarin – INR 2.0 to 3.0** (n=250)	12 weeks	Bleeding and abnormalities of hepatic function
**Yamashita et al 2012** DB RCT, 2012. Multicenter, 1 country (Japan).	Randomized: n=385	AF patients at moderate to high risk of stroke (CHADS2 score ≥ 1).	**Edoxaban 30 mg OD** (n=130) **Edoxaban 60 mg OD** (n=130) **Warfarin – INR 2.0 to 3.0** (n=125)	12 weeks	All bleeding events

AF = atrial fibrillation; **ASA** = acetylsalicylic acid; **CNS** = central nervous system; **CRNM** = clinically relevant non-major bleeding; **CV** = cardiovascular; **DB** = double-blind; **GI** = gastrointestinal; **ICH** = intracerebral hemorrhage; **MI** = myocardial infarction; **NR** = not reported; **NSAIDs** = non-steroidal anti-inflammatory drugs; **OL** = open label; **PL** = placebo; **RCT** = randomized controlled trial; **SE** = systemic embolism; **TIA** = transient ischemic attack; **UK** = United-Kingdom; **VKA** = vitamin K antagonist.

* No data was extracted from these treatment arms since they did not meet the inclusion criteria specified in the protocol. These trials were nevertheless included in the systematic review because they had at least one relevant comparison between two interventions of interest.

† Data was extracted only for dabigatran without ASA (n=100).

For peer review only - http://bmjopen.bmj.com/site/about/guidelines.xhtml

Appendix 5: Evidence network for major bleeding. The width of the lines is proportional to the number of randomized controlled trials (RCTs) comparing each pair of treatments, and the size of each treatment node is proportional to the number of randomised participants (sample size). A dotted line indicates a three-arm RCT and a black node indicates a node included in the analysis but not reported in the main text.

For peer review only

For peer review only - http://bmjopen.bmj.com/site/about/guidelines.xhtml

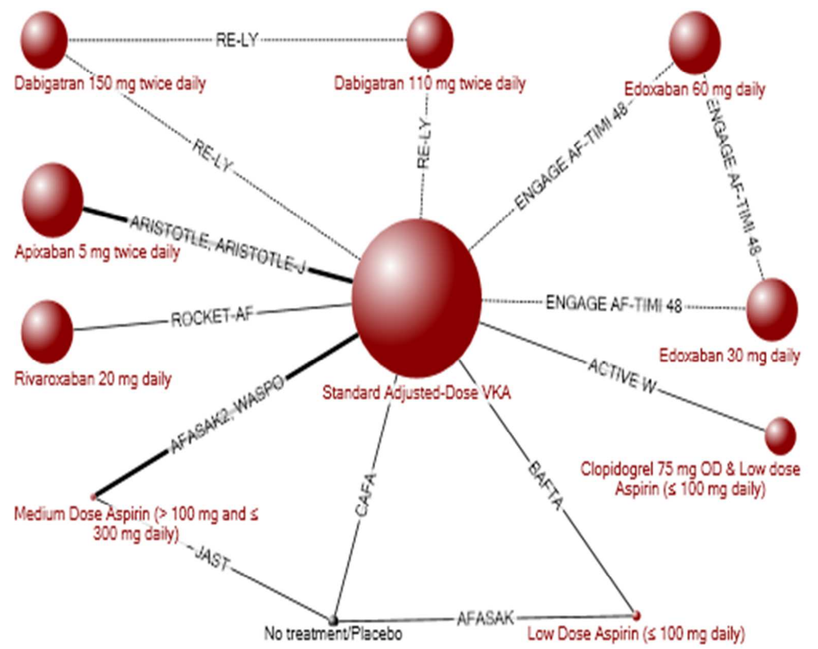

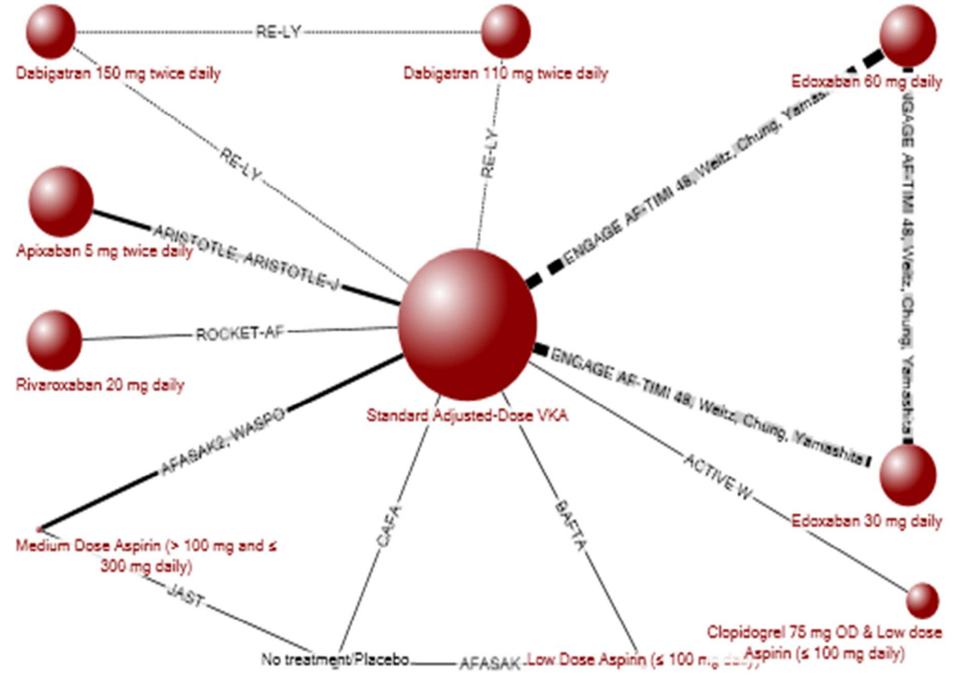

Formatted: Font: (Default) Arial, 11 pt

mg= milligram; RCT = randomized controlled trial

For peer review only

Appendix 6: Summary of results from Network Meta-analysis for All-cause Stroke or Systemic Embolism

Treatment	Pair-wise Meta-Analysis, OR (95% CI) versus standard adjusted dose VKA	NMA, Odds Ratio (95% CrI) versus standard adjusted dose VKA	NMA, Absolute Risk Difference per 1,000 treated (95% CrI) versus standard adjusted dose VKA	Probability Best (%) for Outcome	Rank for Outcome	SUCRA
Standard Adjusted-Dose VKA	NA~~NA~~	NA~~NA~~	NA~~NA~~	0.0%~~0.0%~~	6.0~~6.0~~	50.2%~~50.4%~~
Apixaban 5 mg twice daily	0.79(0.66,0.94)~~0.79(0.66,0.94)~~	0.78 (0.65,0.94)~~0.78(0.65,0.94)~~	4 fewer (6 fewer-1 fewer)~~4 fewer (6 fewer-1 fewer)~~	11.2%~~11.1%~~	2.5~~2.4~~	85.4%~~85.5%~~
Dabigatran 110 mg twice daily	0.91(0.75,1.11)~~0.91(0.75,1.11)~~	0.91 (0.74,1.12)~~0.91(0.74,1.12)~~	2 fewer (5 fewer-2 more)~~2 fewer (5 fewer-2 more)~~	0.1%~~0.1%~~	4.5~~4.5~~	64.7%~~64.9%~~
Dabigatran 150 mg twice daily	0.66(0.53,0.82)~~0.66(0.53,0.82)~~	0.66 (0.53,0.82)~~0.66(0.53,0.82)~~	6 fewer (8 fewer-3 fewer)~~6 fewer (8 fewer-3 fewer)~~	85.2%~~85.3%~~	1.2~~1.2~~	98.1%~~98.1%~~
Rivaroxaban 20 mg daily	0.88(0.74,1.05)~~0.88(0.74,1.05)~~	0.87 (0.74,1.03)~~0.88(0.74,1.04)~~	3 fewer (5 fewer-1 more)~~2 fewer (5 fewer-1 more)~~	1.0%~~1.1%~~	3.9~~3.9~~	71.2%~~71.4%~~
Low Dose Aspirin (≤ 100 mg daily)	2.03(1.32,3.12)~~2.03(1.32,3.12)~~	1.87 (1.26,2.8)~~1.87(1.26,2.8)~~	14 more (5 more-29 more)~~14 more (5 more-29 more)~~	0.0%~~0.0%~~	10.1~~10.1~~	9.4%~~9.4%~~
Medium Dose Aspirin (> 100 mg and ≤ 300 mg daily)	1.21(0.51,2.87)~~1.21(0.51,2.87)~~	1.35 (0.74,2.47)~~1.35(0.74,2.46)~~	6 more (5 fewer-24 more)~~6 more (5 fewer-24 more)~~	1.2%~~1.2%~~	7.8~~7.8~~	32.0%~~32.2%~~
Clopidogrel 75 mg OD & Low dose Aspirin (≤ 100 mg daily)	1.93(1.41,2.64)~~1.93(1.41,2.64)~~	1.93 (1.42,2.65)~~1.93(1.42,2.64)~~	15 more (7 more-26 more)~~15 more (7 more-26 more)~~	0.0%~~0.0%~~	10.2~~10.2~~	8.3%~~8.3%~~
Edoxaban 30 mg daily	1.14(0.94,1.37)~~1.14(0.97,1.33)~~	1.14 (0.98,1.32)~~1.14(0.99,1.33)~~	3 more (1 fewer-6 more)~~3 more (1 fewer-6 more)~~	0.0%~~0.0%~~	7.4~~7.4~~	36.5%~~36.2%~~
Edoxaban 60 mg daily	0.87(0.71,1.05)~~0.87(0.74,1.02)~~	0.87 (0.74,1.01)~~0.87(0.74,1.02)~~	3 fewer (5 fewer-1 more)~~3 fewer (5 fewer-1 more)~~	1.2%~~1.1%~~	3.7~~3.8~~	72.8%~~72.1%~~

Formatted ... [6]
Formatted ... [1]
Formatted ... [2]
Formatted ... [3]
Formatted ... [4]
Formatted ... [5]
Formatted ... [8]
Formatted ... [7]
Formatted ... [9]
Formatted ... [10]
Formatted ... [12]
Formatted ... [11]
Formatted ... [14]
Formatted ... [13]
Formatted ... [15]
Formatted ... [18]
Formatted ... [16]
Formatted ... [17]
Formatted ... [20]
Formatted ... [19]
Formatted ... [21]
Formatted ... [22]
Formatted ... [24]
Formatted ... [23]
Formatted ... [26]
Formatted ... [25]
Formatted ... [27]
Formatted ... [30]
Formatted ... [28]
Formatted ... [29]
Formatted ... [32]
Formatted ... [33]
Formatted ... [31]
Formatted ... [35]
Formatted ... [36]
Formatted ... [34]
Formatted ... [38]
Formatted ... [37]
Formatted ... [39]
Formatted ... [42]
Formatted ... [40]
Formatted ... [41]
Formatted ... [44]
Formatted ... [45]
Formatted ... [43]
Formatted ... [47]
Formatted ... [48]
Formatted ... [46]
Formatted ... [50]
Formatted ... [49]
Formatted ... [51]
Formatted ... [54]
Formatted ... [52]
Formatted ... [53]
Formatted ... [56]
Formatted ... [55]
Formatted ... [57]
Formatted ... [60]

For peer review only - http://bmjopen.bmj.com/site/about/guidelines.xhtml

No treatment/Placebo	1.84(0.66,5.1)1.84(0.66,5.1)	1.53 (0.9,2.63)1.53(0.9,2.61)	9 more (2 fewer-26 more)9 more (2 fewer-26 more)	0.1%0.1%	8.98.8	21.5%21.6%

CI = confidence interval; CrI = credible interval; OR= odds ratio

Bayesian network meta-analysis also allows the analyst to estimate the probability that a treatment is best, second best, and so on for a particular outcome. A simple numerical summary of these probabilities – the surface under the cumulative ranking (SUCRA) – for each treatment can also be calculated [50]. The SUCRA would be 100% when a treatment is certain to be the best and 0% when a treatment is certain to be the worst. SUCRA values enable the ranking of treatments overall for a particular outcome. For example, for Dabigatran 150 mg twice daily for stroke/SE the SUCRA value for is 98.1%, which is better than other treatments. By contrast, Clopidogrel 75 mg OD & Low dose Aspirin (≤ 100 mg daily) has a SUCRA of 8.3% for stroke/SE meaning it is certain to be worst for this outcome among active treatments.

Formatted: Font: (Default) Arial, 8 pt
Formatted: Font: (Default) Arial, 8 pt
Formatted: Font: (Default) Arial, 8 pt
Formatted: Font: (Default) Arial, 8 pt
Formatted: Font: (Default) Arial, 8 pt
Formatted: Font: (Default) Arial, 8 pt
Formatted: Font: 10 pt
Formatted: Font: 10 pt
Formatted: Font: 10 pt
Formatted: Font: 10 pt

Appendix 7: Summary of results from Network Meta-analysis for Major Bleeding

Treatment	Pair-wise Meta-Analysis, OR (95% CI) versus standard adjusted dose VKA	NMA, Odds Ratio (95% CrI) versus standard adjusted dose VKA	NMA, Absolute Risk Difference per 1,000 treated (95% CrI) versus standard adjusted dose VKA	Probability Best (%) for Outcome	Rank for Outcome	SUCRA
Standard Adjusted-Dose VKA	NANA	NANA	NANA	0.0%0.0%	8.08.0	30.2%30.2%
Apixaban 5 mg twice daily	0.69(0.60,0.80)0.69(0.60,0.80)	0.69(0.6,0.8)0.69(0.6,0.8)	10 fewer (13 fewer-7 fewer)10 fewer (13 fewer-7 fewer)	0.0%0.0%	3.23.2	78.1%78.0%
Dabigatran 110 mg twice daily	0.8(0.68,0.94)0.8(0.68,0.94)	0.8(0.69,0.93)0.8(0.69,0.93)	7 fewer (10 fewer-3 fewer)7 fewer (10 fewer-3 fewer)	0.0%0.0%	4.74.7	62.7%62.6%
Dabigatran 150 mg	0.93(0.81,1.07)0.93	0.93(0.8,1.08)0.	3 fewer (7 fewer-3 more)3	0.0%0.0	6.86.8	41.7%41.

Formatted: Font: (Default) Arial, 8 pt
Formatted: Font: (Default) Arial, 8 pt
Formatted: Font: (Default) Arial, 8 pt
Formatted: Font: (Default) Arial, 8 pt
Formatted: Font: (Default) Arial, 8 pt
Formatted: Font: (Default) Arial, 8 pt
Formatted: Font: (Default) Arial, 8 pt
Formatted: Font: (Default) Arial, 8 pt
Formatted: Font: (Default) Arial, 8 pt
Formatted: Font: (Default) Arial, 8 pt
Formatted: Font: (Default) Arial, 8 pt
Formatted: Font: (Default) Arial, 8 pt
Formatted: Font: (Default) Arial, 8 pt
Formatted: Font: (Default) Arial, 8 pt
Formatted: Font: (Default) Arial, 8 pt
Formatted: Font: (Default) Arial, 8 pt
Formatted: Font: (Default) Arial, 8 pt
Formatted: Font: (Default) Arial, 8 pt
Formatted: Font: (Default) Arial, 8 pt
Formatted: Font: (Default) Arial, 8 pt
Formatted: Font: (Default) Arial, 8 pt
Formatted: Font: (Default) Arial, 8 pt
Formatted: Font: (Default) Arial, 8 pt
Formatted: Font: (Default) Arial, 8 pt

twice daily	(0.81,1.07)	93(0.81,1.08)	fewer (7 fewer-3 more)	%		6%
Rivaroxaban 20 mg daily	1.03(0.9,1.18)1.03(0.9,1.18)	1.03(0.89,1.18) 1.03(0.89,1.19)	1 more (4 fewer-6 more)1 more (4 fewer-6 more)	0.0%0.0 %	8.48.4	26.3%26.2%
Low Dose Aspirin (≤ 100 mg daily)	1.01(0.57,1.78)1.01 (0.57,1.78)	1.05(0.6,1.86)1.05(0.59,1.86)	2 more (13 fewer-26 more)2 more (14 fewer-26 more)	0.1%0.1 %	7.97.9	30.6%30.7%
Medium Dose Aspirin (> 100 mg and ≤ 300 mg daily)	1.68(0.50,5.69)1.68 (0.50,5.69)	1.79(0.63,5.67) 1.79(0.62,5.64)	24 more (12 fewer-120 more)24 more (13 fewer-119 more)	0.0%0.0 %	10.010.0	9.8%9.9 %
Clopidogrel 75 mg OD & Low dose Aspirin (≤ 100 mg daily)	1.1(0.82,1.48)1.1(0.82,1.48)	1.1(0.83,1.47)1.1(0.83,1.47)	4 more (6 fewer-15 more)4 more (6 fewer-15 more)	0.0%0.0 %	8.98.9	20.8%20.7%
Edoxaban 30 mg daily	0.46(0.39,0.55)0.47 (0.4,0.55)	0.46(0.4,0.54)0.47(0.4,0.54)	18 fewer (20 fewer-15 fewer)18 fewer (20 fewer-15 fewer)	29.7%29.3%	1.71.7	92.9%92.8%
Edoxaban 60 mg daily	0.78(0.67,0.92)0.78 (0.68,0.89)	0.79(0.69,0.9)0.78(0.69,0.9)	7 fewer (10 fewer-4 fewer)7 fewer (10 fewer-4 fewer)	0.0%0.0 %	4.54.5	64.6%64.7%
No treatment/Placebo	0.39(0.08,2.02)0.39 (0.08,2.02)	0.33(0.08,1.08) 0.33(0.08,1.08)	22 fewer (31 fewer-3 more)22 fewer (31 fewer-3 more)	70.2%70.6%	1.71.7	92.6%92.6%

CI = confidence interval; CrI = credible interval; OR= odds ratio

Bayesian network meta-analysis also allows the analyst to estimate the probability that a treatment is best, second best, and so on for a particular outcome. A simple numerical summary of these probabilities – the surface under the cumulative ranking (SUCRA) – for each treatment can also be calculated [50]. The SUCRA would be 100% when a treatment is certain to be the best and 0% when a treatment is certain to be the worst. SUCRA values enable the ranking of treatments overall for a particular outcome. For example, for major bleeding the SUCRA value for Edoxaban 60 mg is 92.8%, which is better than other treatments. By contrast, Medium Dose Aspirin (> 100 mg and ≤ 300 mg daily) has a SUCRA of 9.9% for major bleeding meaning it is certain to be worst for this outcome.

Formatted ... [61]
Formatted ... [62]
Formatted ... [63]
Formatted ... [64]
Formatted ... [66]
Formatted ... [65]
Formatted ... [67]
Formatted ... [68]
Formatted ... [69]
Formatted ... [70]
Formatted ... [72]
Formatted ... [71]
Formatted ... [75]
Formatted ... [73]
Formatted ... [74]
Formatted ... [76]
Formatted ... [78]
Formatted ... [77]
Formatted ... [79]
Formatted ... [80]
Formatted ... [81]
Formatted ... [82]
Formatted ... [84]
Formatted ... [83]
Formatted ... [87]
Formatted ... [85]
Formatted ... [86]
Formatted ... [88]
Formatted ... [90]
Formatted ... [89]
Formatted ... [91]
Formatted ... [92]
Formatted ... [93]
Formatted ... [94]
Formatted ... [96]
Formatted ... [95]
Formatted ... [99]
Formatted ... [97]
Formatted ... [98]
Formatted ... [100]
Formatted ... [102]
Formatted ... [101]
Formatted ... [103]
Formatted ... [104]
Formatted ... [105]

For peer review only

For peer review only - http://bmjopen.bmj.com/site/about/guidelines.xhtml

Appendix 8: Benefit-Harm plot for each treatment (absolute harm difference per 1,000 patients treated). The absolute risk difference of recurrence of Stroke or systemic embolism relative to standard adjusted dose VKA is presented on the x-axis, while the absolute risk difference of major bleeding relative to standard adjusted dose VKA is on the y-axis. The dot is representative of the median estimate for each treatment while the 95% credible intervals are presented as error bars.

For peer review only

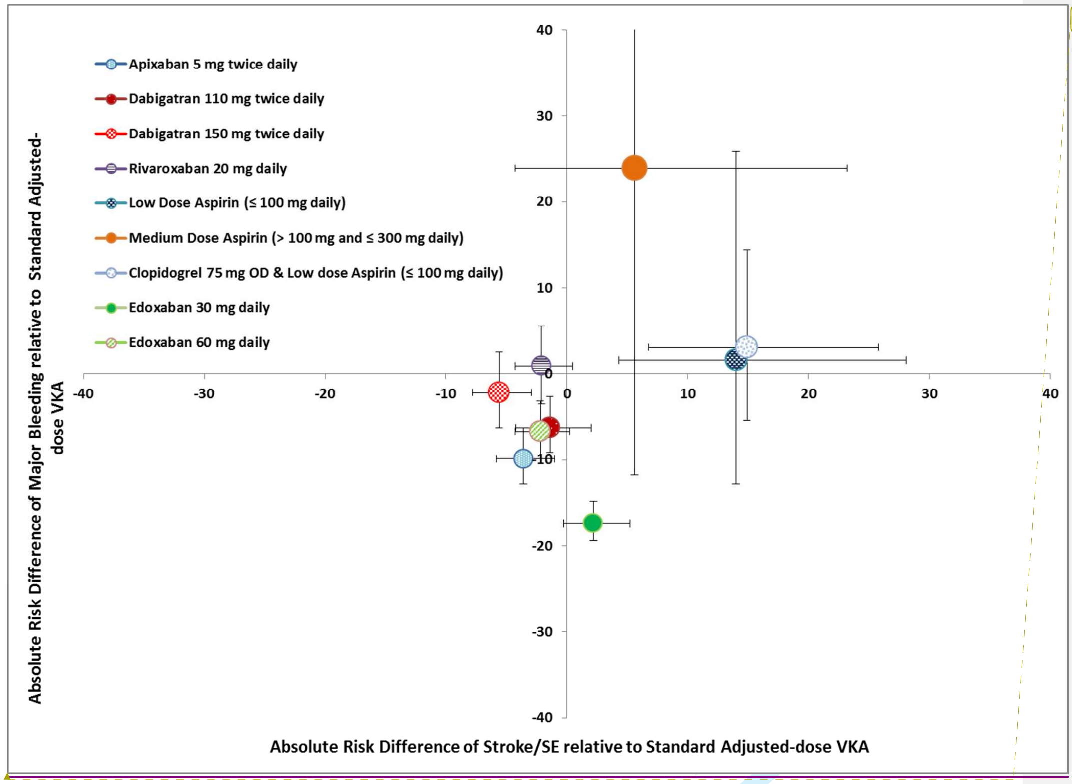

Formatted: Font:

For peer review only - http://bmjopen.bmj.com/site/about/guidelines.xhtml

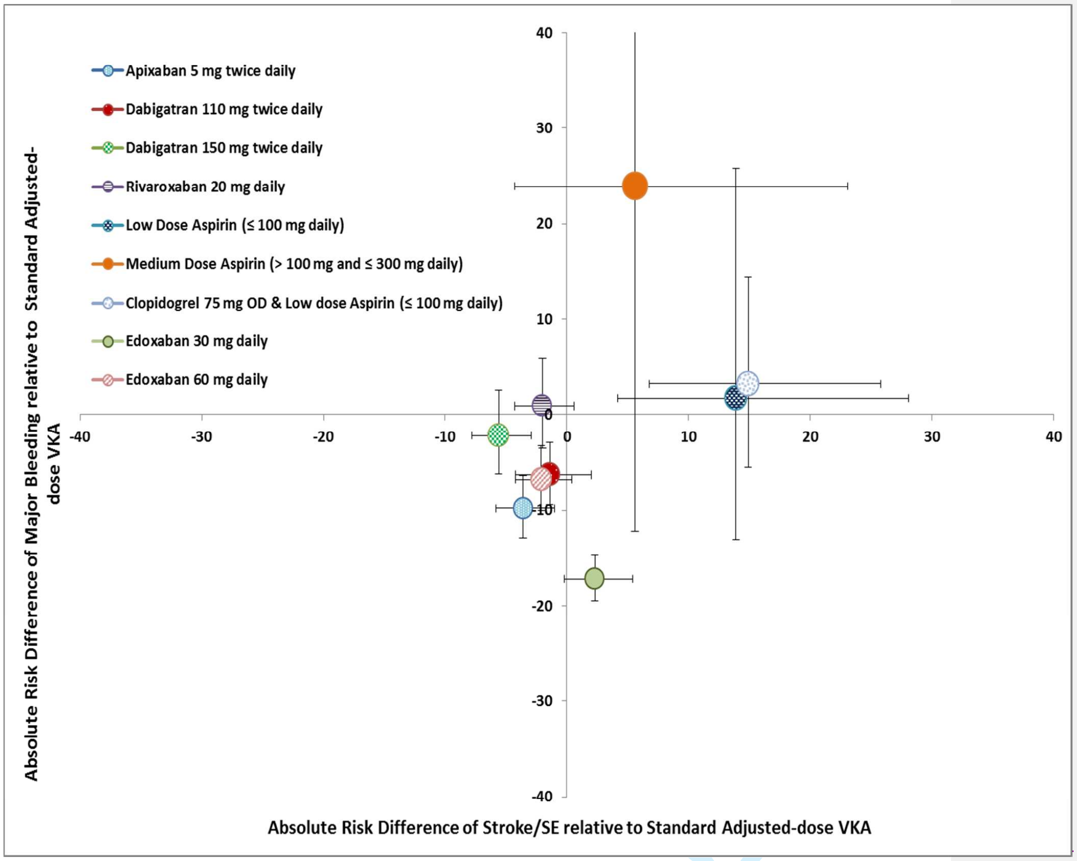

For peer review only - http://bmjopen.bmj.com/site/about/guidelines.xhtml

Appendix 9: Rationale of choice of fixed-effects model and limitations

It is challenging to conduct network meta-analyses when evidence structures are comprised of single study connections, especially when there is obvious heterogeneity in the network. The use of a random-effects model with a vague prior on the between study variance exerts a large degree of influence on the credible intervals (CrI's) because there is insufficient studies to reign in the prior and provide an accurate estimate of the between study variance. As a result, results often appear unrealistic (e.g., wide credible intervals for a treatment that was shown to be associated with a statistically significant decrease). Accordingly, we also considered informative priors on the between study variance from the recently published reports.[11,44] However, even when these priors were applied on the between study variance, they still did not reign in CrI's effect estimates again because the network was comprised of single study connections. The use of the random effects model with either a vague or informative prior was problematic because treatments that were originally associated with statistically significant reductions in stroke or SE relative to standard adjusted dose VKA were no longer associated with significant reductions, limiting external validity of the model (see Figures below on direct estimates in Online Appendix 4 and 5). While we could have opted to use less vague priors (e.g., ¼ of prior in Turner et al[11]), the choice of the prior would have seemed somewhat arbitrary because it was not based on any empirical evidence. As such, we opted to use the fixed-effects model despite the known issues in doing so. By using the fixed-effects model, treatments that were associated with significant reductions in the risk of stroke or SE relative to standard adjusted dose VKA retained significance. We felt that it would have been inappropriate to remove statistical significance by applying vague priors given the large investment involved in developing clinical trials. However, a consequence of retaining statistical significance is seen when comparing newer oral anticoagulants versus one another in Figure 5. Because we assumed the new oral anticoagulants were all associated with reductions in Stroke or SE relative to standard adjusted dose VKA (if that was the case in the underlying trials), significant reductions in the risk of stroke or SE among new oral anticoagulants are observed in Figure 5. This may or may not be the case in clinical practice. As noted in tables 1 and Online Appendix 2, there is considerable heterogeneity across studies. However, the network structure itself (i.e., single study connections), made it difficult to properly adjust for differences in patient and study characteristics using standard approaches. Accordingly, results in Figure 5 should be interpreted with caution. We conducted simple sub-group analyses (breaking randomization) to help adjust for this heterogeneity, which is a very crude way to adjust for heterogeneity. To fully elucidate the comparative benefits and harms of antithrombotic agents, especially across the various sub-populations, rigorously conducted comparative studies, or network meta-regression analyses of patient-level data are required.

For peer review only - http://bmjopen.bmj.com/site/about/guidelines.xhtml

~~Forest Plot – Odds ratio (95% Credible interval) when using vague prior on the sd (i.e, sd~unif(0,2))~~

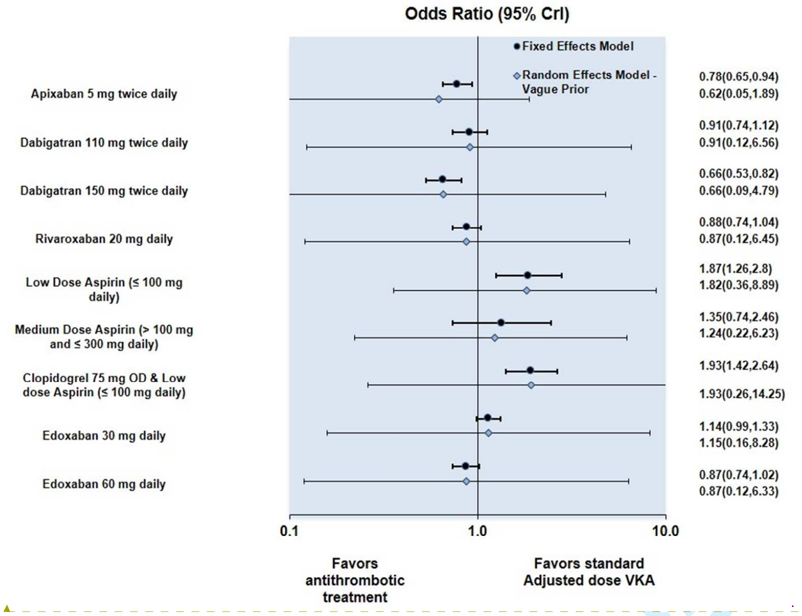

Formatted: Font: (Default) Arial, 11 pt, Bold

Forest Plot – Odds ratio (95% Credible interval) when using informative prior on the sd (i.e, dlnorm(-3.02,0.29)[11]

For peer review only - http://bmjopen.bmj.com/site/about/guidelines.xhtml

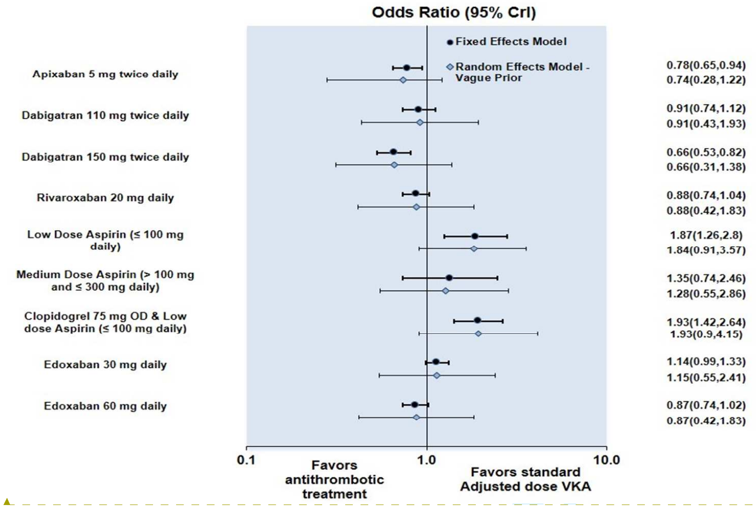

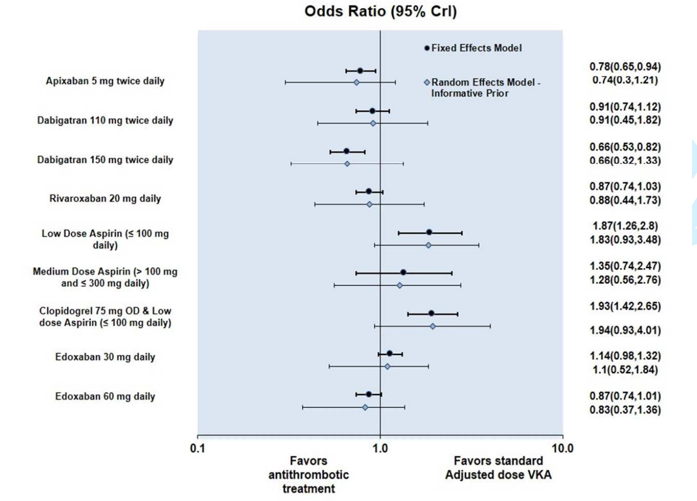

Forest Plot – Odds ratio (95% Credible interval) when using vague prior on the sd (i.e, duniform(0,2))

Formatted: Font: (Default) Arial, 11 pt, Bold

For peer review only - http://bmjopen.bmj.com/site/about/guidelines.xhtml

Formatted: Font: (Default) Arial, 11 pt, Bold

Formatted: Space After: 0 pt, Don't adjust space between Latin and Asian text, Don't adjust space between Asian text and numbers

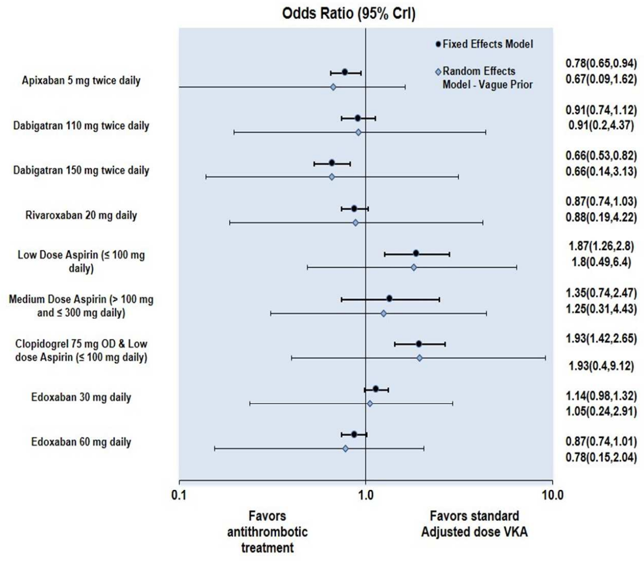

Appendix 10: PRISMA Checklist

Section/topic	#	Checklist item	Reported on page #
**TITLE**			
Title	1	Identify the report as a systematic review, meta-analysis, or both.	0
**ABSTRACT**			
Structured summary	2	Provide a structured summary including, as applicable: background; objectives; data sources; study eligibility criteria, participants, and interventions; study appraisal and synthesis methods; results; limitations; conclusions and implications of key findings; systematic review registration number.	0
**INTRODUCTION**			
Rationale	3	Describe the rationale for the review in the context of what is already known.	1
Objectives	4	Provide an explicit statement of questions being addressed with reference to participants, interventions, comparisons, outcomes, and study design (PICOS).	1
**METHODS**			
Protocol and registration	5	Indicate if a review protocol exists, if and where it can be accessed (e.g., Web address), and, if available, provide registration information including registration number.	2
Eligibility criteria	6	Specify study characteristics (e.g., PICOS, length of follow-up) and report characteristics (e.g., years considered, language, publication status) used as criteria for eligibility, giving rationale.	2-3

For peer review only - http://bmjopen.bmj.com/site/about/guidelines.xhtml

Information sources	7	Describe all information sources (e.g., databases with dates of coverage, contact with study authors to identify additional studies) in the search and date last searched.	2-3
Search	8	Present full electronic search strategy for at least one database, including any limits used, such that it could be repeated.	2-3
Study selection	9	State the process for selecting studies (i.e., screening, eligibility, included in systematic review, and, if applicable, included in the meta-analysis).	3
Data collection process	10	Describe method of data extraction from reports (e.g., piloted forms, independently, in duplicate) and any processes for obtaining and confirming data from investigators.	3
Data items	11	List and define all variables for which data were sought (e.g., PICOS, funding sources) and any assumptions and simplifications made.	3-4
Risk of bias in individual studies	12	Describe methods used for assessing risk of bias of individual studies (including specification of whether this was done at the study or outcome level), and how this information is to be used in any data synthesis.	3,6
Summary measures	13	State the principal summary measures (e.g., risk ratio, difference in means).	3
Synthesis of results	14	Describe the methods of handling data and combining results of studies, if done, including measures of consistency (e.g., I^2) for each meta-analysis.	4
Risk of bias across studies	15	Specify any assessment of risk of bias that may affect the cumulative evidence (e.g., publication bias, selective reporting within studies).	5
Additional analyses	16	Describe methods of additional analyses (e.g., sensitivity or subgroup analyses, meta-regression), if done, indicating which were pre-specified.	5
**RESULTS**			
Study selection	17	Give numbers of studies screened, assessed for eligibility, and included in the review, with reasons for exclusions at each stage, ideally with a flow diagram.	3,5
Study characteristics	18	For each study, present characteristics for which data were extracted (e.g., study size, PICOS, follow-up period) and provide the citations.	5-6, Table 1, Online Appendix 2
Risk of bias within studies	19	Present data on risk of bias of each study and, if available, any outcome level assessment (see item 12).	5
Results of individual studies	20	For all outcomes considered (benefits or harms), present, for each study: (a) simple summary data for each intervention group (b) effect estimates and confidence intervals, ideally with a forest plot.	Figure 3-4
Synthesis of results	21	Present results of each meta-analysis done, including confidence intervals and measures of consistency.	Figure 3-4, Online Appendix 4 and 5
Risk of bias across studies	22	Present results of any assessment of risk of bias across studies (see Item 15).	5
Additional analysis	23	Give results of additional analyses, if done (e.g., sensitivity or subgroup analyses, meta-regression [see Item 16]).	Table 2-3, Online Appendix 10
**DISCUSSION**			
Summary of evidence	24	Summarize the main findings including the strength of evidence for each main outcome; consider their relevance to key groups (e.g., healthcare providers, users, and policy makers).	11-12
Limitations	25	Discuss limitations at study and outcome level (e.g., risk of bias), and at review-level (e.g., incomplete retrieval of identified research, reporting bias).	13-14
Conclusions	26	Provide a general interpretation of the results in the context of other evidence, and implications for future research.	10-14
**FUNDING**			

For peer review only

Funding	27	Describe sources of funding for the systematic review and other support (e.g., supply of data); role of funders for the systematic review.	0

Appendix 11: Assessment of Inconsistency

We plotted the posterior mean deviance of the individual data points in the inconsistency model against their posterior mean deviance in the consistency model to help identify loops where inconsistency is present. In our analysis, the posterior mean deviance contributions are very similar and close to 1, for both models. The consistency model has a similar posterior mean of the residual deviance (296.481 vs. 296.3772) and DIC (19583.0059 vs. 19685.8736) and hence is a similar fit for both models. A few points show a lower value of the posterior mean deviance in the inconsistency model but these values are not too far from 1. Similarly, a few points have higher values of the posterior mean deviance in the consistency model (again with values not too far from 1) suggesting that each model may fit the data points better for certain points. The parameter estimates are also similar for both models and there is considerable overlap in the 95% credible intervals (see table below), suggesting no evidence of inconsistency in the network.

Plot of posterior mean deviance of the individual data points in the inconsistency model against their posterior mean deviance in the consistency model

For peer review only

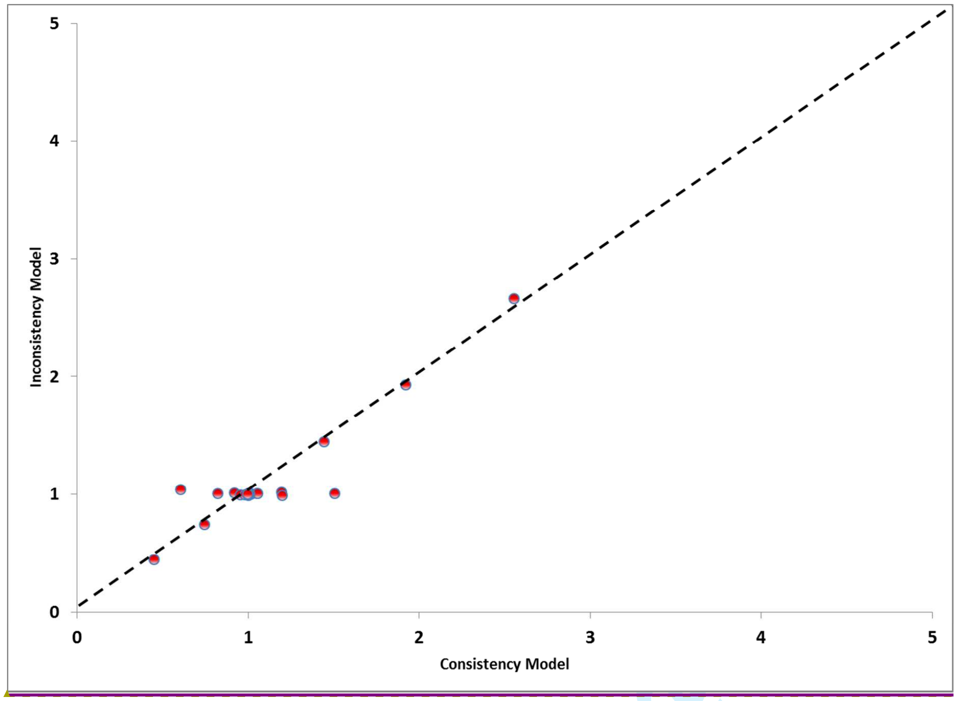

Formatted: Font: (Default) Arial, 11 pt, Bold

For peer review only

For peer review only - http://bmjopen.bmj.com/site/about/guidelines.xhtml

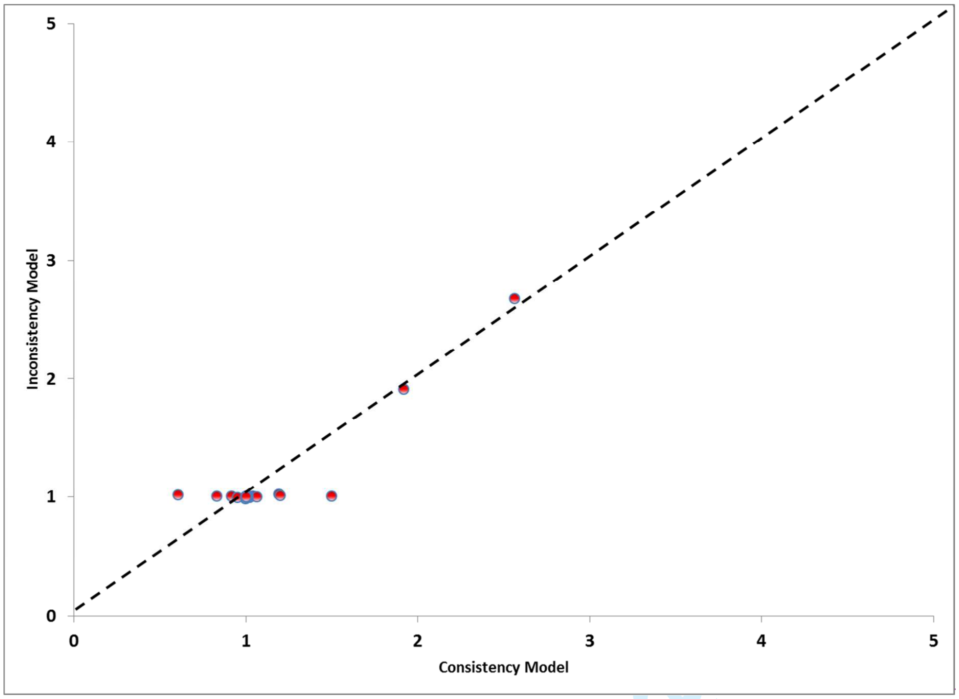

Comparison of effect estimates from consistency and inconsistency models where there is data available for comparison

Comparison	Consistency Model	Inconsistency Model
Apixaban 5 mg twice daily vs Standard Adjusted-Dose VKA	0.78 (0.65,0.94) 0.78 (0.65,0.94)	0.78 (0.65,0.94) 0.78 (0.65,0.94)
Dabigatran 110 mg twice daily vs Apixaban 5 mg twice daily vs	0.91 (0.74,1.12) 0.91 (0.74,1.12)	0.91 (0.74,1.12) 0.91 (0.74,1.12)
Dabigatran 150 mg twice daily vs Standard Adjusted-Dose VKA	0.66 (0.53,0.82) 0.66 (0.53,0.82)	0.66 (0.53,0.82) 0.66 (0.53,0.82)
Rivaroxaban 20 mg daily vs Standard Adjusted-Dose VKA	0.87 (0.74,1.03) 0.88 (0.74,1.04)	0.88 (0.74,1.03) 0.88 (0.74,1.04)
Low Dose Aspirin (≤ 100 mg daily) vs Standard Adjusted-Dose VKA	1.87 (1.26,2.8) 1.87 (1.26,2.8)	2.04 (1.33,3.19) 2.04 (1.33,3.19)
Medium Dose Aspirin (> 100 mg and ≤ 300 mg daily) vs Standard	1.35 (0.74,2.47) 1.35	0.82 (0.33,1.98) 0.82

Formatted: Font: (Default) Arial, 9 pt
Formatted: Font: (Default) Arial, 9 pt, Bold
Formatted: Font: (Default) Arial, 9 pt
Formatted: Font: (Default) Arial, 9 pt, Bold
Formatted: Font: (Default) Arial, 9 pt
Formatted: Font: (Default) Arial, 9 pt, Bold
Formatted: Font: (Default) Arial, 9 pt
Formatted: Font: (Default) Arial, 9 pt
Formatted: Font: (Default) Arial, 9 pt, Bold
Formatted: Font: (Default) Arial, 9 pt
Formatted: Font: (Default) Arial, 9 pt
Formatted: Font: (Default) Arial, 9 pt, Bold
Formatted: Font: (Default) Arial, 9 pt
Formatted: Font: (Default) Arial, 9 pt
Formatted: Font: (Default) Arial, 9 pt, Bold
Formatted: Font: (Default) Arial, 9 pt
Formatted: Font: (Default) Arial, 9 pt
Formatted: Font: (Default) Arial, 9 pt, Bold
Formatted: Font: (Default) Arial, 9 pt
Formatted: Font: (Default) Arial, 9 pt
Formatted: Font: (Default) Arial, 9 pt, Bold
Formatted: Font: (Default) Arial, 9 pt
Formatted: Font: (Default) Arial, 9 pt

For peer review only - http://bmjopen.bmj.com/site/about/guidelines.xhtml

**Adjusted-Dose VKA**	~~(0.74,2.46)~~	~~(0.33,1.97)~~
**Clopidogrel 75 mg OD & Low dose Aspirin (≤ 100 mg daily) vs Standard Adjusted-Dose VKA**	1.93 (1.42,2.65)~~1.93 (1.42,2.64)~~	1.93 (1.42,2.65)~~1.93 (1.42,2.64)~~
**Edoxaban 30 mg daily vs Standard Adjusted-Dose VKA**	1.14 (0.98,1.32)~~1.14 (0.99,1.33)~~	1.14 (0.98,1.32)~~1.14(0.98,1.33)~~
**Edoxaban 60 mg daily vs Standard Adjusted-Dose VKA**	0.87 (0.74,1.01)~~0.87 (0.74,1.02)~~	0.87 (0.74,1.01)~~0.87 (0.74,1.02)~~
**No treatment/Placebo vs Standard Adjusted-Dose VKA**	1.53 (0.9,2.63)~~1.53 (0.9,2.61)~~	1.91 (0.7,5.81)~~1.89 (0.7,5.71)~~
**No treatment/Placebo vs Low Dose Aspirin (≤ 100 mg daily)**	0.82 (0.48,1.38)~~0.82 (0.48,1.38)~~	1 (0.51,1.98)~~1 (0.51,1.98)~~
**Residual deviance**	296.4181	296.3772
**DIC**	19583.0059	19685.8736

Formatted: Font: (Default) Arial, 9 pt, Bold
Formatted: Font: (Default) Arial, 9 pt
Formatted: Font: (Default) Arial, 9 pt
Formatted: Font: (Default) Arial, 9 pt, Bold
Formatted: Font: (Default) Arial, 9 pt
Formatted: Font: (Default) Arial, 9 pt
Formatted: Font: (Default) Arial, 9 pt, Bold
Formatted: Font: (Default) Arial, 9 pt
Formatted: Font: (Default) Arial, 9 pt
Formatted: Font: (Default) Arial, 9 pt, Bold
Formatted: Font: (Default) Arial, 9 pt
Formatted: Font: (Default) Arial, 9 pt
Formatted: Font: (Default) Arial, 9 pt, Bold
Formatted: Font: (Default) Arial, 9 pt
Formatted: Font: (Default) Arial, 9 pt
Formatted: Font: (Default) Arial, 9 pt, Bold
Formatted: Font: (Default) Arial, 9 pt
Formatted: Font: (Default) Arial, 9 pt, Bold
Formatted: Font: (Default) Arial, 9 pt
Formatted: Font: (Default) Arial, 9 pt
Formatted: Font: (Default) Arial, 9 pt, Bold
Formatted: Font: (Default) Arial, 9 pt

Appendix 12: Sensitivity analysis with AVERROES and ACTIVE A

A. Stroke or SE

Evidence Networks for Stroke or SE NMA including AVEROES and ACTIVE A studies

For peer review only - http://bmjopen.bmj.com/site/about/guidelines.xhtml

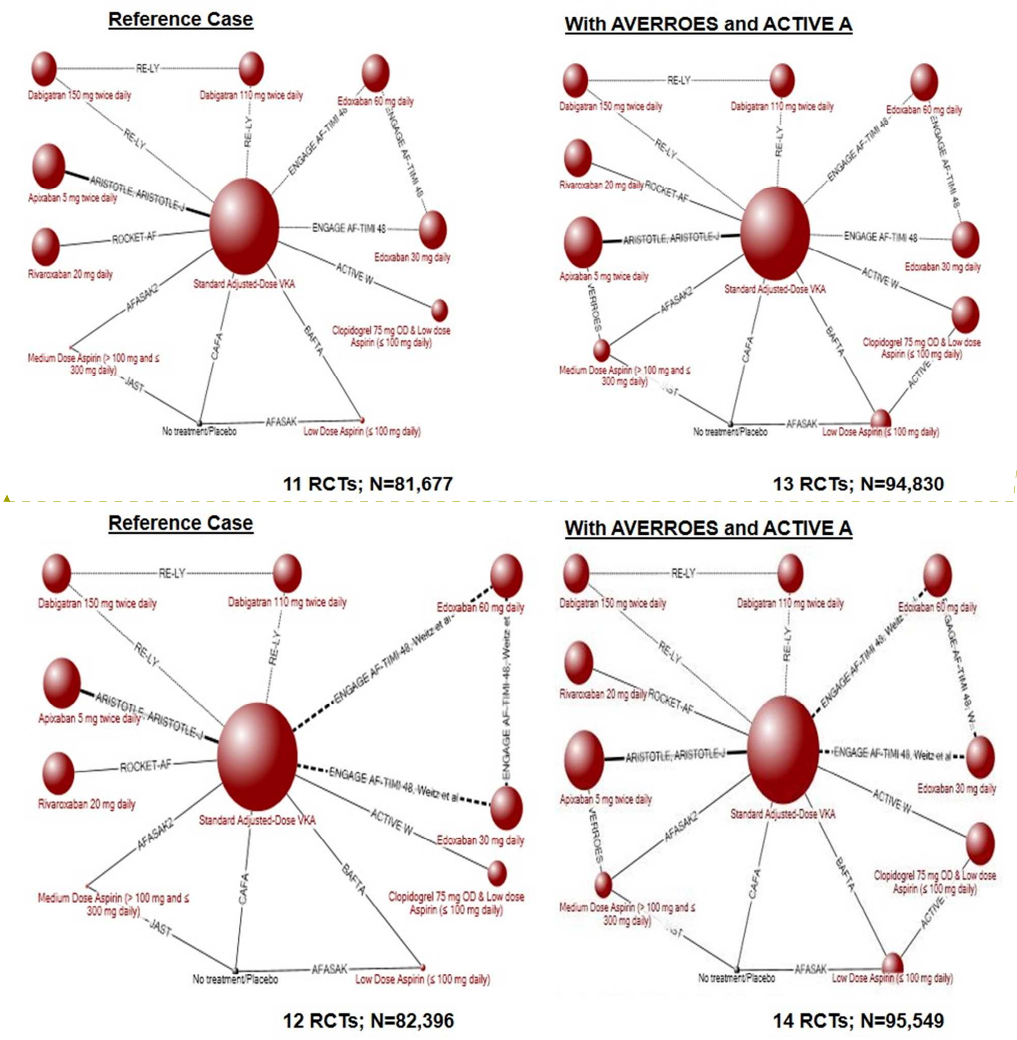

Formatted: **Font:** Bold

Forest Plot for Stroke or SE NMA including AVERROES

and ACTIVE A studies

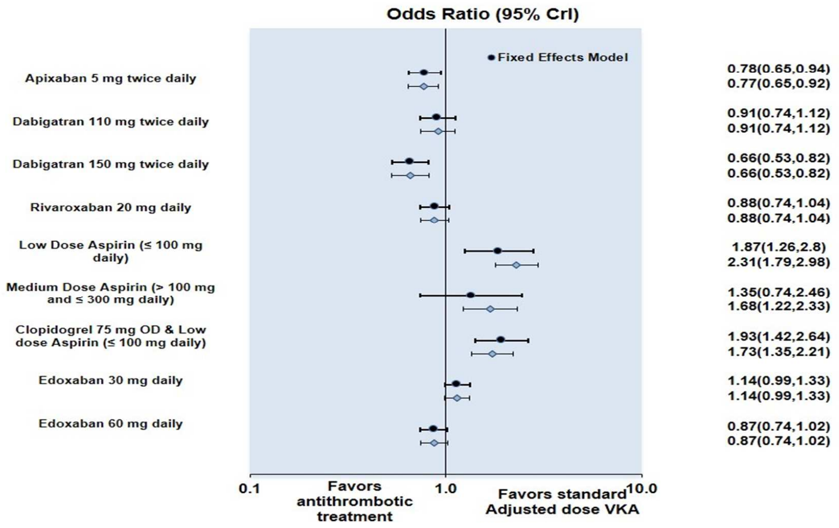

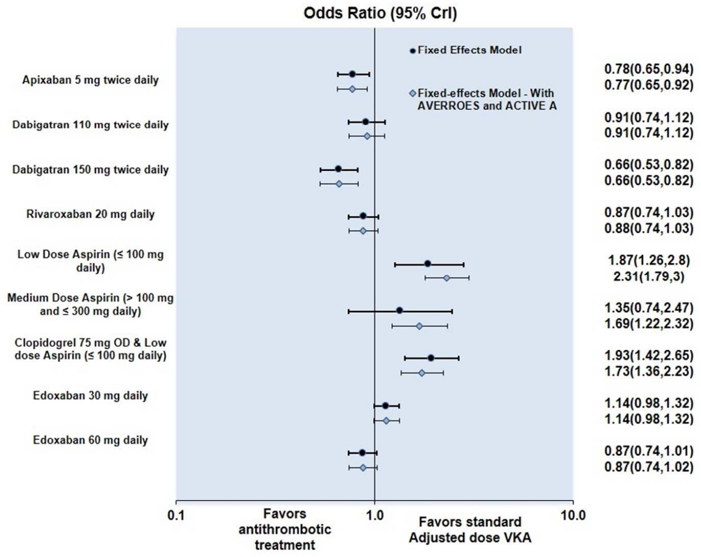

For peer review only - http://bmjopen.bmj.com/site/about/guidelines.xhtml

B. Major Bleeding

Evidence Networks for Major Bleeding NMA including AVERROES and ACTIVE A studies

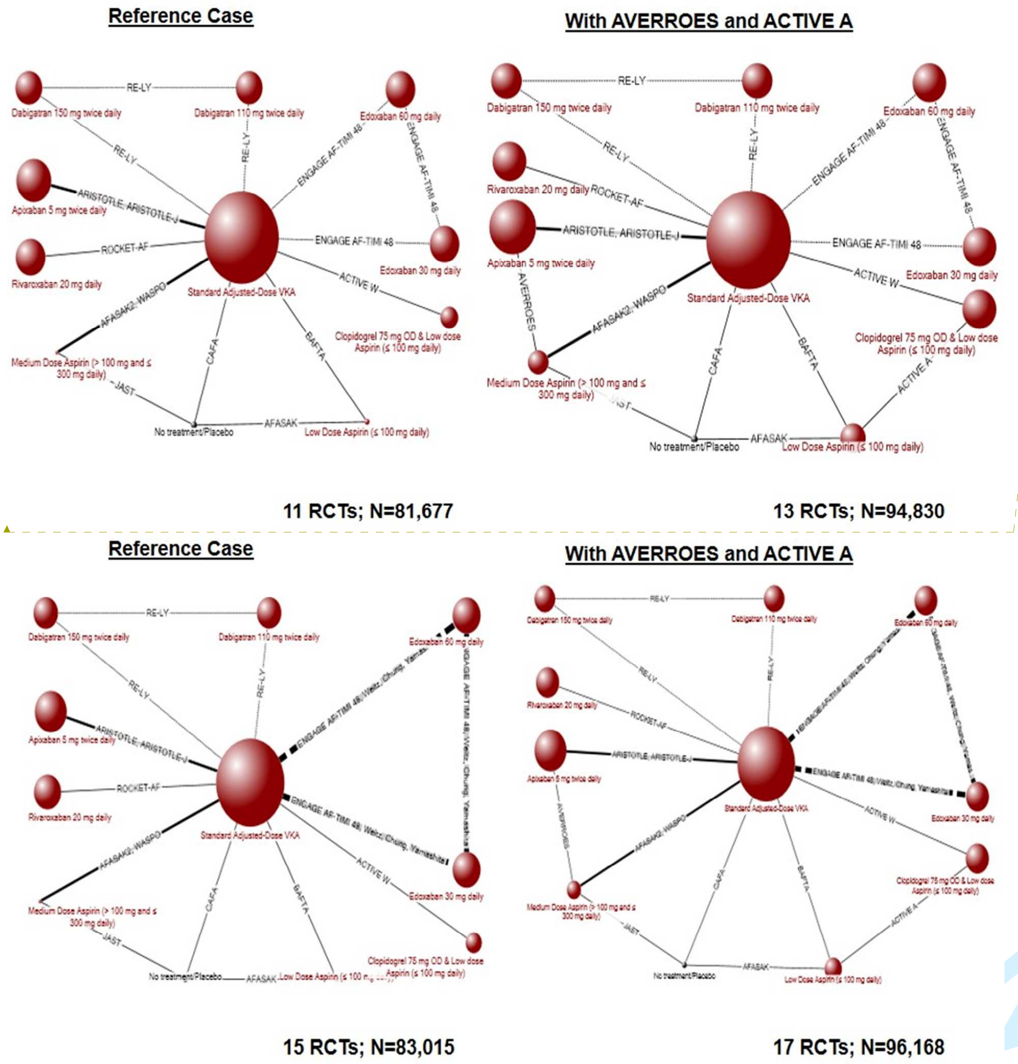

Formatted: Font: Bold

Forest Plot for Major Bleeding NMA including AVERROES and ACTIVE A studies

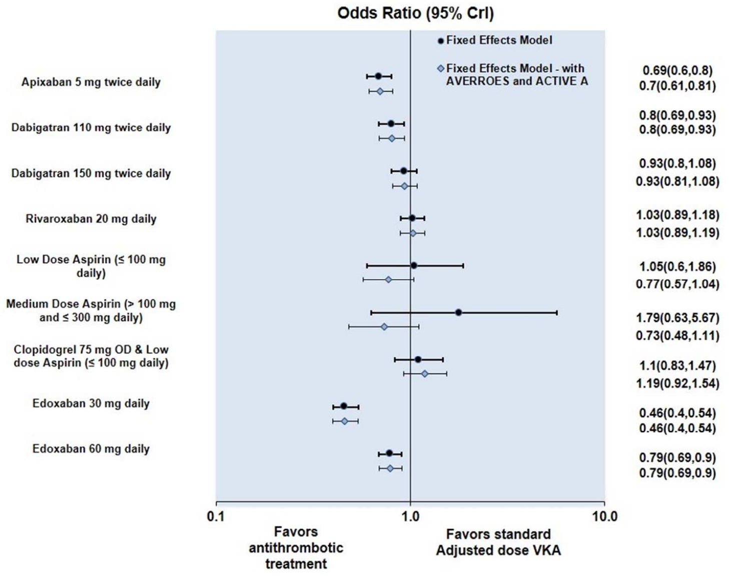

Formatted: Font: 11 pt

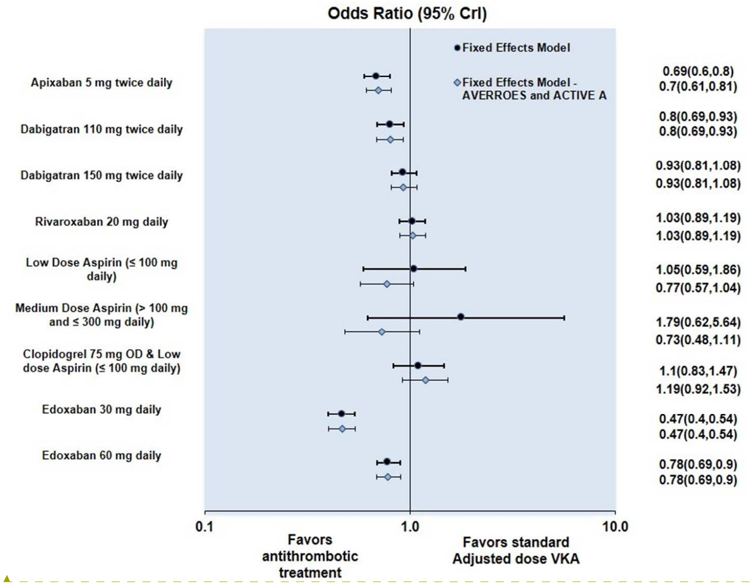

Formatted: Font: (Default) Arial, 11 pt

For peer review only - http://bmjopen.bmj.com/site/about/guidelines.xhtml

Appendix 13: Comparison with previously published network meta-analyses since Harenberg et al[30]

Author (Publication Year)	Method	Search Date	Included Treatments	Registered Protocol	Relative Measure(s)	Absolute Measure(s) reported	Subgroup Analyses Reported
**Cameron 2014**	Bayesian Network Meta-analysis	January 2014	dabigatran, rivaroxaban, apixaban, edoxaban, and vitamin K antagonists (VKA) at a standard adjusted dose (target international normalised ratio 2.0-3.0), acetylsalicylic acid, acetylsalicylic acid and clopidogrel	Yes (PROSPERO registry - CRD42012002721).	OR (95% CrI)	Yes	Yes
**Ruff 2013**	Traditional Meta-analysis	November 2013	dabigatran, rivaroxaban, apixaban, edoxaban	Not Reported	RR (95%CI)	No	Yes
**Dogliotti 2013**	Bayesian Network Meta-analysis	May 2012	dabigatran, rivaroxaban, apixaban, vitamin K antagonists (VKA) at a standard adjusted dose, acetylsalicylic acid, acetylsalicylic acid and clopidogrel	Yes (metacardio.org)	OR (95% CrI)	No	No
**Assiri 2013**	Baysian Network Meta-analysis	August 2012	dabigatran, rivaroxaban, apixaban, vitamin K antagonists (VKA) at a standard adjusted dose, acetylsalicylic acid, acetylsalicylic acid and clopidogrel	Not Reported	RR (95%CrI)	No	No
**Bruins 2013**	Traditional Meta-analysis	June 2013	antistasin, apixaban, betrixaban, darexaban, DU176b, edoxaban, eribaxaban, fondaparinux, idraparinux, otamixaban, razaxaban, rivaroxaban, yagin, YM150, LY517717, SSR126517E, vitamin K antagonists (warfarin and congeners) with the intensity of anticoagulation dose-adjusted using the International Normalised Ratio (INR)	Yes (Cochrane Library)	OR (95%CI) RR (95%CI) in summary table	Yes	Yes

For peer review only - http://bmjopen.bmj.com/site/about/guidelines.xhtml

Appendix 14: Excluded Study List

Inappropriate Study Design

Cardiovasc J S Afr. 2006 Mar;17(2):91.
J Fam Pract. 2006;55(9):753.
Cardiovasc J Afr. 2009 May;20(3):210.
Cardiovasc J Afr. 2009 Sep;20(5):311-2.
Australian Journal of Pharmacy. 2009;90(1073):68.
Formulary. 2010;45(1):16.
Aalbers J. Cardiovasc J Afr. 2010 Nov;21(6):342-3.
Alexander W, et al. PT. 2010;35(10):580-1.
Belavic JM. Nurse Pract. 2011 Sep;36(9):6-7.
Camm AJ. Eur Heart J. 2009 Nov;30(21):2554-5.
Chua D, et al. N Engl J Med. 2009;361(13):1312-3.
Connolly SJ. Eur J Heart Fail. 2009;11(12):1215.
DeSilvey DL. Am J Geriatr Cardiol. 2006 Sep;15(5):326-7.
Dunn A. Ann Intern Med. 2011;154(8):JC4-3.
Eikelboom JW, et al. J Thromb Haemost. 2012;10(5):966-8.
Ezekowitz MD, et al. J Interv Card Electrophysiol. 2011 Dec;32(3):173-80.
Garcia D, et al. Lancet. 2007 Aug 11;370(9586):460-1.
Granger CB. Ann Intern Med. 2012;156(JC1-2):JC1-3.
Hart RG, et al. Ann Intern Med. 2007;147(8):590-2.
Katona A, et al. Eur Heart J. 2006 Jun;27(11):1382.
Kita K, et al. Interventional Cardiology. 2011;3(6):637.
Lopes RD, et al. Evid Based Med. 2011;16(6):187-8.
Nolan J, et al. Br Heart J. 1992 Dec;68(6):544-7.
Paikin JS, et al. Expert Rev Cardiovasc Ther. 2011 Mar;9(3):279-86.
Panichpisal K, et al. Future Neurology. 2011;6(2):155-8.
Roy B, et al. Am J Cardiol. 2012 Feb 1;109(3):370-7.
Sloan MA. Evid Based Med. 2006;11(6):170.
Vassiliou VS. N Engl J Med. 2012;366(1):88-90.
Veloso HH, et al. Chest. 2005 Jul;128(1):475.
Weinberger J. Curr Cardiol Rep. 2010;12(1):1-2.

Inappropriate Intervention

Lancet. 1994 Mar 19;343(8899):687-91.
The Stroke Prevention in Atrial Fibrillation Investigators. Arch Intern Med. 1996 Feb 26;156(4):409-16.
Archer SL, et al. Am Heart J. 1995 Aug;130(2):287-95.
Edvardsson N, et al. J Intern Med. 2003 Jul;254(1):95-101.
Hart RG, et al. Cerebrovasc Dis. 2000 Jan;10(1):39-43.
Hellemons BS, et al. Control Clin Trials. 1999 Aug;20(4):386-93.
Hori M, et al. Circ J. 2012 Jun 5.
Miller VT, et al. Neurology. 1996 Jan;46(1):238-40.
Perez-Gomez F, et al. Eur Heart J. 2007;28(8):996-1003.

For peer review only

Singer DE, et al. Am Heart J. 1992 Dec;124(6):1567-73.
Connolly S, et al. Eur Heart J. 2013 May:34(20):1498-505

Inappropriate Population

ACTIVE Investigators, et al. N Engl J Med 2009;360(20):2066-78.
Circulation 1991;84(2):527-39.
Connolly SJ, et al. N Engl J Med 2011;364(9):806-17.
Dewilde W, et al. Am Heart J. 2009 Nov;158(5):713-8.
Diener HC, et al. Lancet Neurol. 2012 Mar;11(3):225-31.
EAFT (European Atrial Fibrillation Trial) Study Group. Lancet. 1993;342(8882):1255-62.
Eikelboom JW, et al. J Stroke Cerebrovasc Dis. 2012 Aug;21(6):429-35.
ESPRIT Study Group, et al. Lancet Neurol. 2007 Feb;6(2):115-24.
Hart RG, et al. Cerebrovasc Dis. 2008;25(4):344-7.
N Engl J Med 1990;322(12):863-8.
Wang TH, et al. Eur Heart J. 2007 Sep;28(18):2200-7.

Inappropriate Outcomes

De Caterina R, et al. Eur Heart J. 2010 Sep;31(17):2133-40.
Ezekowitz MD, et al. Circulation. 1995 Oct 15;92(8):2178-82.
Lorenzoni R, et al. Am Heart J. 2004 Jul;148(1):e6.

Others

Cardiovasc J Afr. 2011 May;22(3):164.
Active Steering Committee, et al. Am Heart J. 2006 Jun;151(6):1187-93.
ROCKET AF Study Investigators. Am Heart J. 2010 Mar;159(3):340-7.
The Stroke Prevention in Atrial Fibrillation Investigators. Stroke. 1990 Apr;21(4):538-45.
Boysen G, et al. Stroke. 1990;21(Suppl I):I-92.
De Schryver EL. Cerebrovasc Dis. 2000 Mar;10(2):147-50.
Desai A. Clinical Trials Registry - India (CTRI). 2006.
Eikelboom JW, et al. Am Heart J. 2010 Mar;159(3):348-53.
Ezekowitz MD, et al. Am Heart J. 2009 May;157(5):805-10, 810.e1-2.
Flaker G, et al. J Am Coll Cardiol. 2012 Feb 28;59(9):854-5.
Lopes RD, et al. Lancet. 2012 Oct 2. Epub ahead of print.
Lopes RD, et al. Am Heart J. 2010 Mar;159(3):331-9.
Mant J, et al. Cerebrovasc Dis. 2007;23(Suppl 2):10.
Mant J, et al. Cardiol Rev. 2008;25(7):32-6.
Mant JW, et al. BMC Cardiovasc Disord. 2003 Aug 26;3:9.
Posada IS, et al. Am Heart J. 1999 Jul;138(1 Pt 1):137-43.
Van Latum JC. Ned Tijdschr Geneeskd. 1994;138(20):1025-31.
Yigit Z. Turk Kardiyoloji Dernegi Arsivi. 2000;28(1):8-19+4.

For peer review only

~~Appendix 15: Completion rates~~

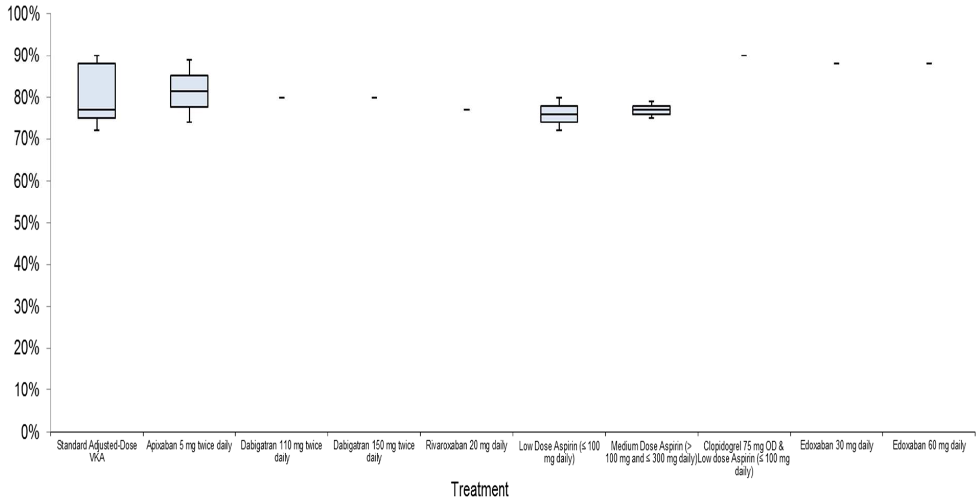

Formatted: Font: Bold

review only

For peer review only

Page 32: [1] Formatted **Chris Cameron** **3/18/2014 1:57:00 PM**

Font: (Default) Arial, 8 pt

Page 32: [2] Formatted **Chris Cameron** **3/18/2014 1:57:00 PM**

Font: (Default) Arial, 8 pt

Page 32: [3] Formatted **Chris Cameron** **3/18/2014 1:57:00 PM**

Font: (Default) Arial, 8 pt

Page 32: [4] Formatted **Chris Cameron** **3/18/2014 1:57:00 PM**

Font: (Default) Arial, 8 pt

Page 32: [5] Formatted **Chris Cameron** **3/18/2014 1:57:00 PM**

Font: (Default) Arial, 8 pt

Page 32: [6] Formatted **Chris Cameron** **3/18/2014 1:57:00 PM**

Font: (Default) Arial, 8 pt

Page 32: [7] Formatted **Chris Cameron** **3/18/2014 1:57:00 PM**

Font: (Default) Arial, 8 pt

Page 32: [8] Formatted **Chris Cameron** **3/18/2014 1:57:00 PM**

Font: (Default) Arial, 8 pt

Page 32: [9] Formatted **Chris Cameron** **3/18/2014 1:57:00 PM**

Font: (Default) Arial, 8 pt

Page 32: [10] Formatted **Chris Cameron** **3/18/2014 1:57:00 PM**

Font: (Default) Arial, 8 pt

Page 32: [11] Formatted **Chris Cameron** **3/18/2014 1:57:00 PM**

Font: (Default) Arial, 8 pt

Page 32: [12] Formatted **Chris Cameron** **3/18/2014 1:57:00 PM**

Font: (Default) Arial, 8 pt

Page 32: [13] Formatted **Chris Cameron** **3/18/2014 1:57:00 PM**

Font: (Default) Arial, 8 pt

Page 32: [14] Formatted **Chris Cameron** **3/18/2014 1:57:00 PM**

Font: (Default) Arial, 8 pt

Page 32: [15] Formatted **Chris Cameron** **3/18/2014 1:57:00 PM**

Font: (Default) Arial, 8 pt

Page 32: [16] Formatted **Chris Cameron** **3/18/2014 1:57:00 PM**

Font: (Default) Arial, 8 pt

Page 32: [17] Formatted **Chris Cameron** **3/18/2014 1:57:00 PM**

Font: (Default) Arial, 8 pt

Page 32: [18] Formatted **Chris Cameron** **3/18/2014 1:57:00 PM**

Font: (Default) Arial, 8 pt

Page 32: [19] Formatted **Chris Cameron** **3/18/2014 1:57:00 PM**

Font: (Default) Arial, 8 pt

Font: (Default) Arial, 8 pt

Page 32: [21] Formatted Chris Cameron 3/18/2014 1:57:00 PM

Font: (Default) Arial, 8 pt

Page 32: [22] Formatted Chris Cameron 3/18/2014 1:57:00 PM

Font: (Default) Arial, 8 pt

Page 32: [23] Formatted Chris Cameron 3/18/2014 1:57:00 PM

Font: (Default) Arial, 8 pt

Page 32: [24] Formatted Chris Cameron 3/18/2014 1:57:00 PM

Font: (Default) Arial, 8 pt

Page 32: [25] Formatted Chris Cameron 3/18/2014 1:57:00 PM

Font: (Default) Arial, 8 pt

Page 32: [26] Formatted Chris Cameron 3/18/2014 1:57:00 PM

Font: (Default) Arial, 8 pt

Page 32: [27] Formatted Chris Cameron 3/18/2014 1:57:00 PM

Font: (Default) Arial, 8 pt

Page 32: [28] Formatted Chris Cameron 3/18/2014 1:57:00 PM

Font: (Default) Arial, 8 pt

Page 32: [29] Formatted Chris Cameron 3/18/2014 1:57:00 PM

Font: (Default) Arial, 8 pt

Page 32: [30] Formatted Chris Cameron 3/18/2014 1:57:00 PM

Font: (Default) Arial, 8 pt

Page 32: [31] Formatted Chris Cameron 3/18/2014 1:57:00 PM

Font: (Default) Arial, 8 pt

Page 32: [32] Formatted Chris Cameron 3/18/2014 1:57:00 PM

Font: (Default) Arial, 8 pt

Page 32: [33] Formatted Chris Cameron 3/18/2014 1:57:00 PM

Font: (Default) Arial, 8 pt

Page 32: [34] Formatted Chris Cameron 3/18/2014 1:57:00 PM

Font: (Default) Arial, 8 pt

Page 32: [35] Formatted Chris Cameron 3/18/2014 1:57:00 PM

Font: (Default) Arial, 8 pt

Page 32: [36] Formatted Chris Cameron 3/18/2014 1:57:00 PM

Font: (Default) Arial, 8 pt

Page 32: [37] Formatted Chris Cameron 3/18/2014 1:57:00 PM

Font: (Default) Arial, 8 pt

Page 32: [38] Formatted Chris Cameron 3/18/2014 1:57:00 PM

Font: (Default) Arial, 8 pt

Page 32: [39] Formatted Chris Cameron 3/18/2014 1:57:00 PM

For peer review only

Page 32: [40] Formatted Chris Cameron 3/18/2014 1:57:00 PM

Font: (Default) Arial, 8 pt

Page 32: [41] Formatted Chris Cameron 3/18/2014 1:57:00 PM

Font: (Default) Arial, 8 pt

Page 32: [42] Formatted Chris Cameron 3/18/2014 1:57:00 PM

Font: (Default) Arial, 8 pt

Page 32: [43] Formatted Chris Cameron 3/18/2014 1:57:00 PM

Font: (Default) Arial, 8 pt

Page 32: [44] Formatted Chris Cameron 3/18/2014 1:57:00 PM

Font: (Default) Arial, 8 pt

Page 32: [45] Formatted Chris Cameron 3/18/2014 1:57:00 PM

Font: (Default) Arial, 8 pt

Page 32: [46] Formatted Chris Cameron 3/18/2014 1:57:00 PM

Font: (Default) Arial, 8 pt

Page 32: [47] Formatted Chris Cameron 3/18/2014 1:57:00 PM

Font: (Default) Arial, 8 pt

Page 32: [48] Formatted Chris Cameron 3/18/2014 1:57:00 PM

Font: (Default) Arial, 8 pt

Page 32: [49] Formatted Chris Cameron 3/18/2014 1:57:00 PM

Font: (Default) Arial, 8 pt

Page 32: [50] Formatted Chris Cameron 3/18/2014 1:57:00 PM

Font: (Default) Arial, 8 pt

Page 32: [51] Formatted Chris Cameron 3/18/2014 1:57:00 PM

Font: (Default) Arial, 8 pt

Page 32: [52] Formatted Chris Cameron 3/18/2014 1:57:00 PM

Font: (Default) Arial, 8 pt

Page 32: [53] Formatted Chris Cameron 3/18/2014 1:57:00 PM

Font: (Default) Arial, 8 pt

Page 32: [54] Formatted Chris Cameron 3/18/2014 1:57:00 PM

Font: (Default) Arial, 8 pt

Page 32: [55] Formatted Chris Cameron 3/18/2014 1:57:00 PM

Font: (Default) Arial, 8 pt

Page 32: [56] Formatted Chris Cameron 3/18/2014 1:57:00 PM

Font: (Default) Arial, 8 pt

Page 32: [57] Formatted Chris Cameron 3/18/2014 1:57:00 PM

Font: (Default) Arial, 8 pt

Page 32: [58] Formatted Chris Cameron 3/18/2014 1:57:00 PM

Font: (Default) Arial, 8 pt

For peer review only

Font: (Default) Arial, 8 pt

Page 32: [60] Formatted Chris Cameron 3/18/2014 1:57:00 PM

Font: (Default) Arial, 8 pt

Page 34: [61] Formatted Chris Cameron 3/18/2014 2:54:00 PM

Font: (Default) Arial, 8 pt

Page 34: [62] Formatted Chris Cameron 3/18/2014 2:54:00 PM

Font: (Default) Arial, 8 pt

Page 34: [63] Formatted Chris Cameron 3/18/2014 2:54:00 PM

Font: (Default) Arial, 8 pt

Page 34: [64] Formatted Chris Cameron 3/18/2014 2:54:00 PM

Font: (Default) Arial, 8 pt

Page 34: [65] Formatted Chris Cameron 3/18/2014 2:54:00 PM

Font: (Default) Arial, 8 pt

Page 34: [66] Formatted Chris Cameron 3/18/2014 2:54:00 PM

Font: (Default) Arial, 8 pt

Page 34: [67] Formatted Chris Cameron 3/18/2014 2:54:00 PM

Font: (Default) Arial, 8 pt

Page 34: [68] Formatted Chris Cameron 3/18/2014 2:54:00 PM

Font: (Default) Arial, 8 pt

Page 34: [69] Formatted Chris Cameron 3/18/2014 2:54:00 PM

Font: (Default) Arial, 8 pt

Page 34: [70] Formatted Chris Cameron 3/18/2014 2:54:00 PM

Font: (Default) Arial, 8 pt

Page 34: [71] Formatted Chris Cameron 3/18/2014 2:54:00 PM

Font: (Default) Arial, 8 pt

Page 34: [72] Formatted Chris Cameron 3/18/2014 2:54:00 PM

Font: (Default) Arial, 8 pt

Page 34: [73] Formatted Chris Cameron 3/18/2014 2:54:00 PM

Font: (Default) Arial, 8 pt

Page 34: [74] Formatted Chris Cameron 3/18/2014 2:54:00 PM

Font: (Default) Arial, 8 pt

Page 34: [75] Formatted Chris Cameron 3/18/2014 2:54:00 PM

Font: (Default) Arial, 8 pt

Page 34: [76] Formatted Chris Cameron 3/18/2014 2:54:00 PM

Font: (Default) Arial, 8 pt

Page 34: [77] Formatted Chris Cameron 3/18/2014 2:54:00 PM

Font: (Default) Arial, 8 pt

Page 34: [78] Formatted Chris Cameron 3/18/2014 2:54:00 PM

For peer review only

Page 34: [79] Formatted	Chris Cameron	3/18/2014 2:54:00 PM

Font: (Default) Arial, 8 pt

Page 34: [80] Formatted	Chris Cameron	3/18/2014 2:54:00 PM

Font: (Default) Arial, 8 pt

Page 34: [81] Formatted	Chris Cameron	3/18/2014 2:54:00 PM

Font: (Default) Arial, 8 pt

Page 34: [82] Formatted	Chris Cameron	3/18/2014 2:54:00 PM

Font: (Default) Arial, 8 pt

Page 34: [83] Formatted	Chris Cameron	3/18/2014 2:54:00 PM

Font: (Default) Arial, 8 pt

Page 34: [84] Formatted	Chris Cameron	3/18/2014 2:54:00 PM

Font: (Default) Arial, 8 pt

Page 34: [85] Formatted	Chris Cameron	3/18/2014 2:54:00 PM

Font: (Default) Arial, 8 pt

Page 34: [86] Formatted	Chris Cameron	3/18/2014 2:54:00 PM

Font: (Default) Arial, 8 pt

Page 34: [87] Formatted	Chris Cameron	3/18/2014 2:54:00 PM

Font: (Default) Arial, 8 pt

Page 34: [88] Formatted	Chris Cameron	3/18/2014 2:54:00 PM

Font: (Default) Arial, 8 pt

Page 34: [89] Formatted	Chris Cameron	3/18/2014 2:54:00 PM

Font: (Default) Arial, 8 pt

Page 34: [90] Formatted	Chris Cameron	3/18/2014 2:54:00 PM

Font: (Default) Arial, 8 pt

Page 34: [91] Formatted	Chris Cameron	3/18/2014 2:54:00 PM

Font: (Default) Arial, 8 pt

Page 34: [92] Formatted	Chris Cameron	3/18/2014 2:54:00 PM

Font: (Default) Arial, 8 pt

Page 34: [93] Formatted	Chris Cameron	3/18/2014 2:54:00 PM

Font: (Default) Arial, 8 pt

Page 34: [94] Formatted	Chris Cameron	3/18/2014 2:54:00 PM

Font: (Default) Arial, 8 pt

Page 34: [95] Formatted	Chris Cameron	3/18/2014 2:54:00 PM

Font: (Default) Arial, 8 pt

Page 34: [96] Formatted	Chris Cameron	3/18/2014 2:54:00 PM

Font: (Default) Arial, 8 pt

Page 34: [97] Formatted	Chris Cameron	3/18/2014 2:54:00 PM

Font: (Default) Arial, 8 pt

For peer review only

Font: (Default) Arial, 8 pt

Page 34: [99] Formatted	Chris Cameron	3/18/2014 2:54:00 PM

Font: (Default) Arial, 8 pt

Page 34: [100] Formatted	Chris Cameron	3/18/2014 2:54:00 PM

Font: (Default) Arial, 8 pt

Page 34: [101] Formatted	Chris Cameron	3/18/2014 2:54:00 PM

Font: (Default) Arial, 8 pt

Page 34: [102] Formatted	Chris Cameron	3/18/2014 2:54:00 PM

Font: (Default) Arial, 8 pt

Page 34: [103] Formatted	Chris Cameron	3/18/2014 1:58:00 PM

Font: 10 pt

Page 34: [104] Formatted	Chris Cameron	3/18/2014 1:58:00 PM

Font: 10 pt

Page 34: [105] Formatted	Chris Cameron	3/18/2014 1:58:00 PM

Font: 10 pt

For peer review only

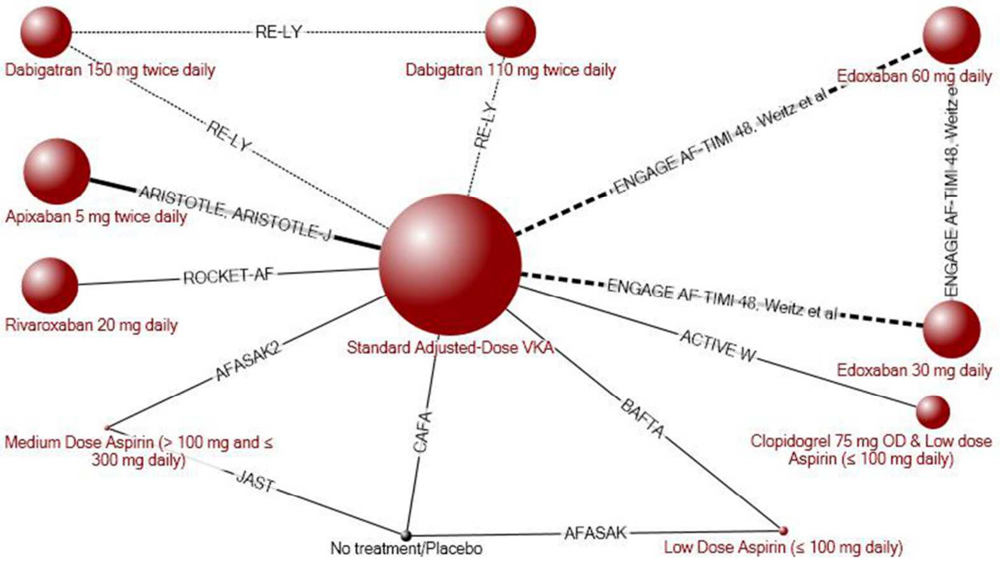

Figure 1: Evidence network for all cause stroke or systemic embolism.* The width of the lines is proportional to the number of randomized controlled trials comparing each pair of treatments, and the size of each treatment node is proportional to the number of randomised participants (sample size). A dotted line indicates a three-arm randomized controlled trial and a black node indicates a node included in the analysis but not reported in the main text.

67x40mm (300 x 300 DPI)

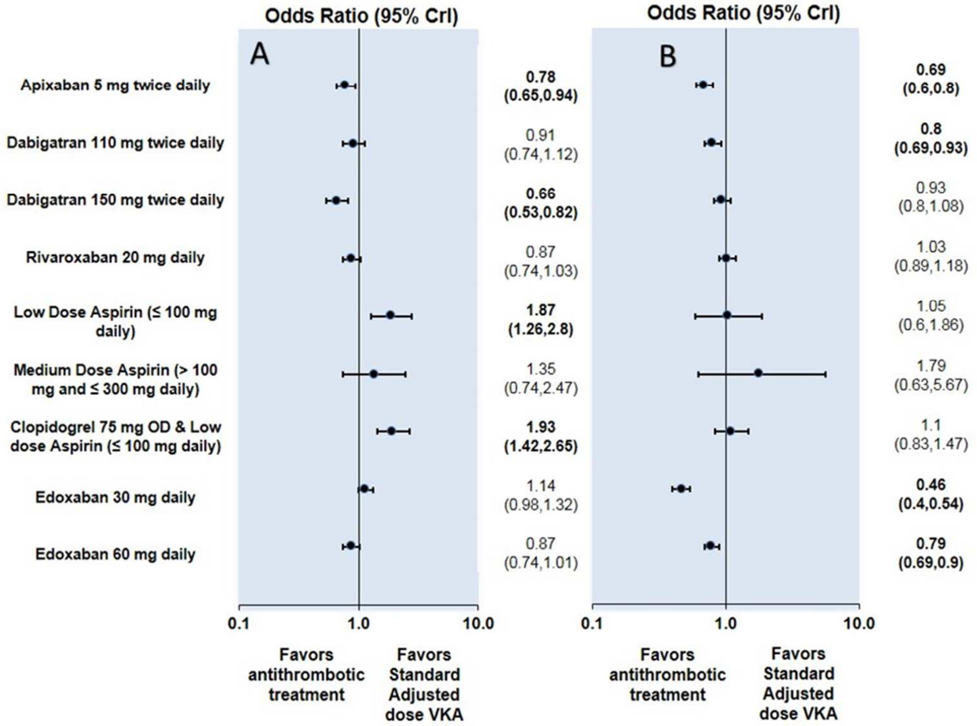

Figure 2: Odds ratio for all cause stroke or systemic embolism (A) and major bleeding (B) in Bayesian network meta-analysis versus standard adjusted dose VKA.
82x61mm (300 x 300 DPI)

For peer review only - http://bmjopen.bmj.com/site/about/guidelines.xhtml

Standard Adjusted-Dose VKA	**0.69 (0.6,0.8)**	**0.8 (0.69,0.93)**	0.93 (0.8,1.08)	1.03 (0.89,1.18)	1.05 (0.6,1.86)	1.79 (0.63,5.67)	1.1 (0.83,1.47)	**0.46 (0.4,0.54)**	**0.79 (0.69,0.9)**
**0.78 (0.65,0.94)**	Apixaban 5 mg twice daily	1.16 (0.94,1.43)	**1.35 (1.1,1.65)**	**1.48 (1.21,1.82)**	1.52 (0.85,2.74)	2.59 (0.9,8.26)	**1.59 (1.15,2.19)**	**0.67 (0.54,0.83)**	1.13 (0.93,1.38)
0.91 (0.74,1.12)	1.17 (0.89,1.54)	Dabigatran 110 mg twice daily	1.16 (0.9976,1.36)	**1.28 (1.04,1.58)**	1.32 (0.73,2.37)	2.24 (0.77,7.16)	1.37 (0.99,1.9)	**0.58 (0.47,0.72)**	0.98 (0.8,1.2)
**0.66 (0.53,0.82)**	0.84 (0.63,1.12)	**0.72 (0.57,0.91)**	Dabigatran 150 mg twice daily	1.1 (0.9,1.35)	1.13 (0.63,2.04)	1.93 (0.67,6.15)	1.18 (0.85,1.63)	**0.5 (0.4,0.61)**	0.84 (0.69,1.03)
0.87 (0.74,1.03)	1.12 (0.87,1.43)	0.96 (0.74,1.25)	**1.33 (1.01,1.75)**	Rivaroxaban 20 mg daily	1.03 (0.57,1.85)	1.75 (0.6,5.56)	1.07 (0.78,1.48)	**0.45 (0.37,0.56)**	**0.77 (0.63,0.93)**
**1.87 (1.26,2.8)**	**2.4 (1.55,3.72)**	**2.05 (1.32,3.23)**	**2.85 (1.81,4.5)**	**2.14 (1.4,3.31)**	Low Dose Aspirin (≤ 100 mg daily)	1.7 (0.52,6.08)	1.05 (0.55,1.97)	**0.44 (0.24,0.79)**	0.75 (0.42,1.33)
1.35 (0.74,2.47)	1.72 (0.92,3.25)	1.48 (0.78,2.79)	**2.05 (1.08,3.9)**	1.54 (0.83,2.88)	0.72 (0.38,1.35)	Medium Dose Aspirin (> 100 mg and ≤ 300 mg daily)	0.61 (0.19,1.83)	**0.26 (0.08,0.75)**	0.44 (0.14,1.27)
**1.93 (1.42,2.65)**	**2.47 (1.73,3.55)**	**2.12 (1.46,3.08)**	**2.93 (2.01,4.31)**	**2.21 (1.56,3.15)**	1.03 (0.62,1.71)	1.43 (0.73,2.81)	Clopidogrel 75 mg OD & Low dose Aspirin (≤ 100 mg daily)	**0.42 (0.3,0.58)**	**0.71 (0.52,0.98)**
1.14 (0.98,1.32)	**1.45 (1.15,1.84)**	1.25 (0.97,1.61)	**1.73 (1.32,2.26)**	**1.3 (1.04,1.63)**	**0.61 (0.4,0.92)**	0.84 (0.45,1.56)	**0.59 (0.42,0.83)**	Edoxaban 30 mg daily	1.7 (1.45,1.99)
0.87 (0.74,1.01)	1.11 (0.87,1.41)	0.95 (0.73,1.23)	**1.31 (1,1.73)**	0.99 (0.79,1.25)	**0.46 (0.3,0.7)**	0.64 (0.34,1.19)	**0.45 (0.32,0.63)**	**0.76 (0.65,0.89)**	Edoxaban 60 mg daily

Figure 3: Odds Ratios (OR) from network meta-analyses for stroke or systemic embolism and major bleeding for all pair-wise comparisons.* ORs for recurrence of stroke or systemic embolism are below the diagonal (row-defining treatment versus column-defining treatment) and those for major bleeding are above the diagonal and in blue (column-defining treatment versus row-defining treatment). To obtain ORs for comparisons in the opposite direction, reciprocals should be taken (e.g., the OR for standard dose warfarin compared with apixaban 5 mg twice daily for stroke or systemic embolism is 1/0.78=1.28). Significant results are in bold and underscored (and in larger font).
82x64mm (300 x 300 DPI)

only

For peer review only - http://bmjopen.bmj.com/site/about/guidelines.xhtml

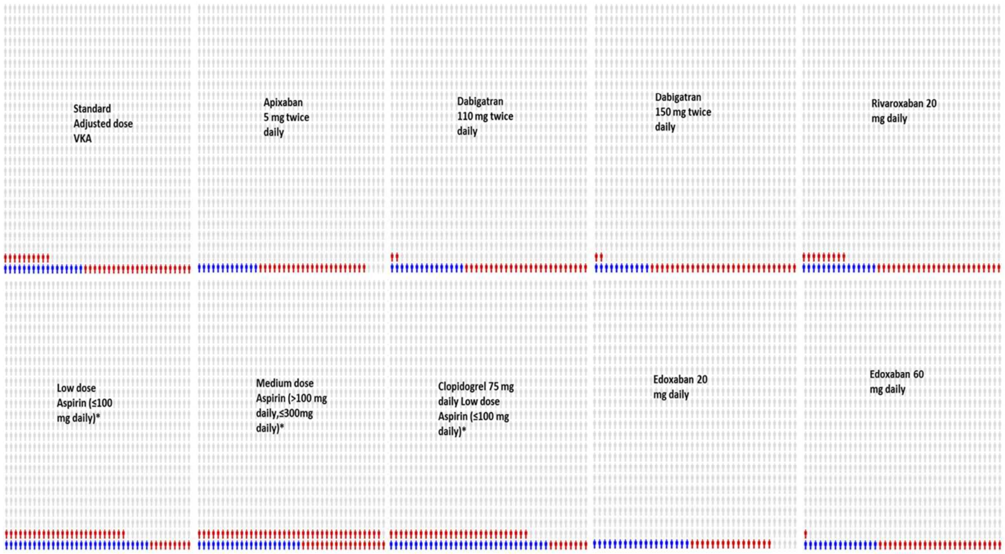

Figure 4: Icon array illustrating the absolute risks of Stroke or systemic embolism (blue) and major bleeding episodes (red) per 1000 patients treated*
114x63mm (300 x 300 DPI)

review only

SUPPLEMENTARY APPENDIX

Appendix 1: Search Strategy

**OVERVIEW**	
Interface:	Ovid
Databases:	EBM Reviews - Cochrane Central Register of Controlled Trials <May 2012><br>Ovid Embase 1974 to June 6, 2012<br>Ovid MEDLINE 1946 to June 7, 2012<br>Ovid MEDLINE Daily<br>Ovid MEDLINE In-Process & Other Non-Indexed Citations<br>**Note:** Subject headings have been customized for each database. Duplicates between databases were removed in Ovid.
Date of Search:	June 7, 2012; updated January 23, 2014
Alerts:	Monthly search updates began June 11, 2012 and ran until project completion
Study Types:	Randomized Controlled Trials
Limits:	Publication years 1988-present<br>Humans<br>Conference abstracts omitted

**SYNTAX GUIDE**	
/	At the end of a phrase, searches the phrase as a subject heading
.sh	At the end of a phrase, searches the phrase as a subject heading
MeSH	Medical Subject Heading
exp	Explode a subject heading
*	Before a word, indicates that the marked subject heading is a primary topic;<br>or, after a word, a truncation symbol (wildcard) to retrieve plurals or varying endings
#	Truncation symbol for one character
ADJ	Requires words are adjacent to each other (in any order)
ADJ#	Adjacency within # number of words (in any order)
.ti	Title
.ab	Abstract
.hw	Heading Word; usually includes subject headings and controlled vocabulary
.nm.	Name of substance word
.ot	Original title
.pt	Publication type
.rn	CAS registry number
use cctr	Select results from Cochrane database of Clinical Trials
use oemezd	Select results from Embase database
use pmez	Select results from Medline database

Clinical Multi-database Strategy

Line #	Search Strategy
1	(211914-51-1 or 211915-06-9).rn,nm. or (dabigatran* or Pradaxa or Pradax or Prazaxa or Praxada or bibr 1048 or bibr1048 or bibr953 or bibr 953 or rendix).ti,ot,ab,sh,rn,hw,nm.
2	(BMS 562247 or BMS562247 or apixaban* or Eliquis or Eliques).ti,ot,ab,sh,rn,hw,nm. or 503612-47-3.rn,nm.
3	(rivaroxaban* or Xarelto or BAY 59-7939 or BAY59-7939 or BAY597939 or BAY

	597939).ti,ot,ab,sh,rn,hw,nm. or 366789-02-8.rn,nm.
4	(90055-48-4 or 113665-84-2).rn,nm. or (clopidogrel* or clopilet or grepid or iscover or plavix or zopya or zylagren or zyllt or duocover or duoplavin).ti,ot,ab,sh,rn,hw,nm.
5	Aspirin/ or 50-78-2.rn,nm. or (acetylsalicylic acid* or ASA* or acenterine or acesal or acetan or acetard or aceticil or aceticyl or acetilum or acetonyl or acetilum or acetonyl or acetophen or acetosal or acetosalicylic acid or acetosalin or acetosalum or acetyl salicylate or acetyl salicylic acid or acetylin or acetylo or acetylon or acetylosalicylic acid or acetylsal or acetysal or acetylsalicyclic acid or acetylsalicyl or acetylsalicylate or acetylsalycic acid or acetylsalycylic acid or oracidulatum or actorin or Acylpyrin or acylpyrine or acytosal or adiro or alabunkun or alasil or albyl-e or alka seltzer or alkASA or Aloxiprimum or anacin or anasprin or anopyrin or ansin or anthrom or aptor or arthralgyl or asaflow or ascription or orasaphen or asapor or asatard or asawin or aspec or aspent or aspergum or aspex or aspilets or aspirem or aspirgran or ASAa or ASAe or ASAine or aspirisucre or aspisol or aspo cid or aspro or asrina or asrivo or asta or asteric or astrix or bebesan or biprin or bokey or boxazin or breoprin or bufferin or cafenol or caprin or cASA or catalgine or catalgix or cemerit or cemirit or claradin or claragine or Colfarit or comoprin or contrheuma or daga or darosal or Dispril or dispirin or dolean or dusil or Easprin or ecasil or Ecotrin or egalgic or emocin or empirin or encaprin or encine em or Endosprin or entaprin or entercin or enteroprin or enterosarine or enterospirine or entrophen or eskotrin or euthermine or extren or genasprin or globentyl or godamed or gotosan or helicon or idotyl or infatabs a or istopirin or istopyrine or ivepirine or juvepirine or keypo or kilios or kinderASA or Magnecyl or measurin or mejoral or melabon or Micristin or micropyrin or mikristin or miniasal or mycrisitin or naspro or novasen or nu seal or nu seals or ortho acetoxybenzoate or ortho acetoxybenzoic acid or ortho acetyloxybenzoate or ortho acetyloxybenzoic acid or ostoprin or pancemol or paracin or paynocil or pengo or plewin or Polopirin or polopiryna or premaspin or primaspan or proprin or pyronoval or reumyl or rhodine or rhonal or ronal or salacetin or salisalido or sargepirine or soldral or solpyron or Solprin or solucetyl or solupsa or Solupsan or spren or super tru or tapal or temagin or tevapirin or th 2152 or thrombo-aspilets or treupahlin or treuphalin or tromalyt or tromcor or turiital or verin or vitalink or xaxa or Zorprin).ti,ot,ab,sh,rn,hw,nm.
6	Warfarin/ or 81-81-2.rn,nm. or (adoisine or aldocumar or athrombin* or befarin or brumolin or carfin or circuvit or compound 42 or coumadan or coumadin or coumadine or coumafen or coumafene or coumefene or coumaphene or coumarins or dagonal or farin or jantoven or kumatox or maforan or marevan or orfarin or panwarfarin or panwarfin or prothromadin or simarc 2 or sofarin or tedicumar or tintorane or waran or warfant or warfar or warfarin or warfarine or warfil or warfilone or warnerin).ti,ot,ab,sh,rn,hw,nm.
7	Acenocoumarol/ or 152-72-7.rn,nm. or (acenocoumarol* or nicoumalone or acenocoumarin or acenocoumarine or acenocoumarole or acenocoumarolum or acenocumarol or acenocumarolo or acenocumerol or acenkumarin or acitrom or ascumar or neo sintrom or neosintrom or neositron or nicoumalone or nicumalon or nitrovarfarian or nitrowarfarin or sincoumar or sincumar or sinkumar or sinthrom or sinthrome or sintrom or sintroma or sintron or syncoumar or syncumar or synthrom or syntrom or syntroma or sintron or syncoumar or syncumar or syntrom or trombostop or zotil or sinthrome or synthrom or syncoumar or syncumar or sinkumar or sintrom or minisintrom or trombostop or zotil).ti,ot,ab,sh,rn,hw,nm.
8	or/1-7
9	Atrial fibrillation/
10	((atrial or atrium or auricular or heart) adj1 fibrillation*).ti,ab.
11	(a-fib or afib).ti,ab.
12	9 or 10 or 11
13	8 and 12
14	13 use pmez
15	13 use cctr
16	14 or 15
17	*dabigatran/ or (dabigatran* or Pradaxa or Pradax or Prazaxa or praxada or bibr 1048 or bibr1048 or bibr953 or bibr 953 or rendix).ti,ab.
18	*apixaban/ or (BMS 562247 or BMS562247 or apixaban* or Eliquis or Eliques).ti,ab.
19	*rivaroxaban/ or (rivaroxaban* or Xarelto or BAY 59-7939 or BAY59-7939 or BAY597939 or BAY 597939).ti,ab.
20	*clopidogrel/ or *acetylsalicylic acid plus clopidogrel/ or (clopidogrel* or clopilet or grepid or iscover or plavix or zopya or zylagren or zyllt or duocover or duoplavin).ti,ab.
21	*warfarin/ or (adoisine or aldocumar or athrombin* or befarin or brumolin or carfin or circuvit or compound 42 or coumadan or coumadin or coumadine or coumafen or coumafene or coumefene or

For peer review only

	coumaphene or coumarins or dagonal or farin or jantoven or kumatox or maforan or marevan or orfarin or panwarfarin or panwarfin or prothromadin or simarc 2 or sofarin or tedicumar or tintorane or waran or warfant or warfar or warfarin* or warfarine or warfil or warfilone or warnerin).ti,ab.
22	*acetylsalicylic acid/ or (acetylsalicylic acid* or ASA* or acenterine or acesal or acetan or acetard or aceticil or aceticyl or acetilum or acetonyl or acetilum or acetonyl or acetophen or acetosal or acetosalicylic acid or acetosalin or acetosalum or acetyl salicylate or acetyl salicylic acid or acetylin or acetylo or acetylon or acetylosalicylic acid or acetylsal or acetysal or acetylsalicyclic acid or acetylsalicyl or acetylsalicylate or acetylsalycic acid or acetylsalycylic acid or oracidulatum or actorin or Acylpyrin or acylpyrine or acytosal or adiro or alabunkun or alasil or albyl-e or alka seltzer or alkASA or Aloxiprimum or anasprin or anopyrin or ansin or anthrom or aptor or arthralgyl or asaflow or orasaphen or asapor or asatard or asawin or aspec or aspent or aspergum or aspex or aspilets or aspirem or aspirgran or ASAa or ASAe or ASAine or aspirisucre or aspisol or aspo cid or aspro or asrina or asrivo or asta or asteric or astrix or bebesan or biprin or bokey or boxazin or breoprin or bufferin or cafenol or caprin or cASA or catalgine or catalgix or cemerit or cemirit or claradin or claragine or Colfarit or comoprin or contrheuma or daga or darosal or Dispril or dispirin or dolean or dusil or Easprin or ecasil or Ecotrin or egalgic or emocin or empirin or encaprin or encine em or Endosprin or entaprin or entercin or enteroprin or enterosarine or enterospirine or entrophen or eskotrin or euthermine or extren or genasprin or globentyl or godamed or gotosan or helicon or idotyl or infatabs a or istopirin or istopyrine or ivepirine or juvepirine or keypo or kilios or kinderASA or Magnecyl or measurin or mejoral or melabon or Micristin or micropyrin or mikristin or miniasal or mycrisitin or naspro or novasen or nu seal or nu seals or ortho acetoxybenzoate or ortho acetoxybenzoic acid or ortho acetyloxybenzoate or ortho acetyloxybenzoic acid or ostoprin or pancemol or paracin or paynocil or pengo or plewin or Polopirin or polopiryna or premaspin or primaspan or proprin or pyronoval or reumyl or rhodine or rhonal or ronal or salacetin or salisalido or sargepirine or soldral or solpyron or Solprin or solucetyl or solupsa or Solupsan or spren or super tru or tapal or temagin or tevapirin or th 2152 or thrombo-aspilets or treupahlin or treuphalin or tromalyt or tromcor or turiital or verin or vitalink or xaxa or Zorprin).ti,ab.
23	*Acenocoumarol/ or (acenocoumarol* or nicoumalone or acenocoumarin or acenocoumarine or acenocoumarole or acenocoumarolum or acenocumarol or acenocumarolo or acenocumerol or acenkumarin or acitrom or ascumar or neo sintrom or neosintrom or neositron or nicoumalone or nicumalon or nitrovarfarian or nitrowarfarin or sincoumar or sincumar or sinkumar or sinthrom or sinthrome or sintrom or sintroma or sintron or syncoumar or syncumar or synthrom or syntrom or syntroma or sintron or syncoumar or syncumar or syntrom or trombostop or zotil or sinthrome or synthrom or syncoumar or syncumar or sinkumar or sintrom or minisintrom or trombostop or zotil).ti,ab.
24	or/17-23
25	Heart atrium fibrillation/
26	((atrial or atrium or auricular or heart) adj1 fibrillation*).ti,ab.
27	(a-fib or afib).ti,ab.
28	25 or 26 or 27
29	24 and 28
30	29 use oemezd
31	30 not conference abstract.pt.
32	16 or 31
33	32
34	limit 33 to english language [Limit not valid in CCTR; records were retained]
35	(Randomized Controlled Trial or Controlled Clinical Trial).pt.
36	Randomized Controlled Trial/
37	Randomized Controlled Trials as Topic/
38	"Randomized Controlled Trial (topic)"/
39	Controlled Clinical Trial/
40	Controlled Clinical Trials as Topic/
41	"Controlled Clinical Trial (topic)"/
42	Randomization/
43	Random Allocation/
44	Double-Blind Method/
45	Double Blind Procedure/

For peer review only

46	Double-Blind Studies/
47	Single-Blind Method/
48	Single Blind Procedure/
49	Single-Blind Studies/
50	Placebos/
51	Placebo/
52	Control Groups/
53	Control Group/
54	(random* or sham or placebo*).ti,ab,hw.
55	((singl* or doubl*) adj (blind* or dumm* or mask*)).ti,ab,hw.
56	((tripl* or trebl*) adj (blind* or dumm* or mask*)).ti,ab,hw.
57	(control* adj3 (study or studies or trial*)).ti,ab.
58	(Nonrandom* or non random* or non-random* or quasi-random* or quasirandom*).ti,ab,hw.
59	allocated.ti,ab,hw.
60	((open label or open-label) adj5 (study or studies or trial*)).ti,ab,hw.
61	or/35-60
62	34 and 61
63	limit 62 to yr = "1988 -Current"
64	remove duplicates from 63

OTHER DATABASES	
PubMed	Same MeSH, keywords, limits, and study types used as per MEDLINE search, with appropriate syntax used.

Grey Literature

Dates for Search:	**June 12, 2012 – July 8, 2012; updated January 2014**
**Keywords:**	dabigatran, Pradax, Pradaxa, apixaban, Eliquis, rivaroxaban, Xarelto, clopidogrel, Plavix, acetylsalicylic acid, Aspirin, warfarin, Coumadin, acenocoumarol, Sintrom, Sinthrome<br>Atrial fibrillation
**Limits:**	Publication years 1988-present, English

Relevant items from the following sections of the CADTH grey literature checklist, “Grey matters: a practical tool for evidence-based searching” (http://www.cadth.ca/resources/grey-matters) were searched:

- Health Technology Assessment Agencies
- Health Economic
- Clinical Practice Guidelines
- Databases (free)

Internet Search

For peer review only

Appendix 2: PRISMA Diagram

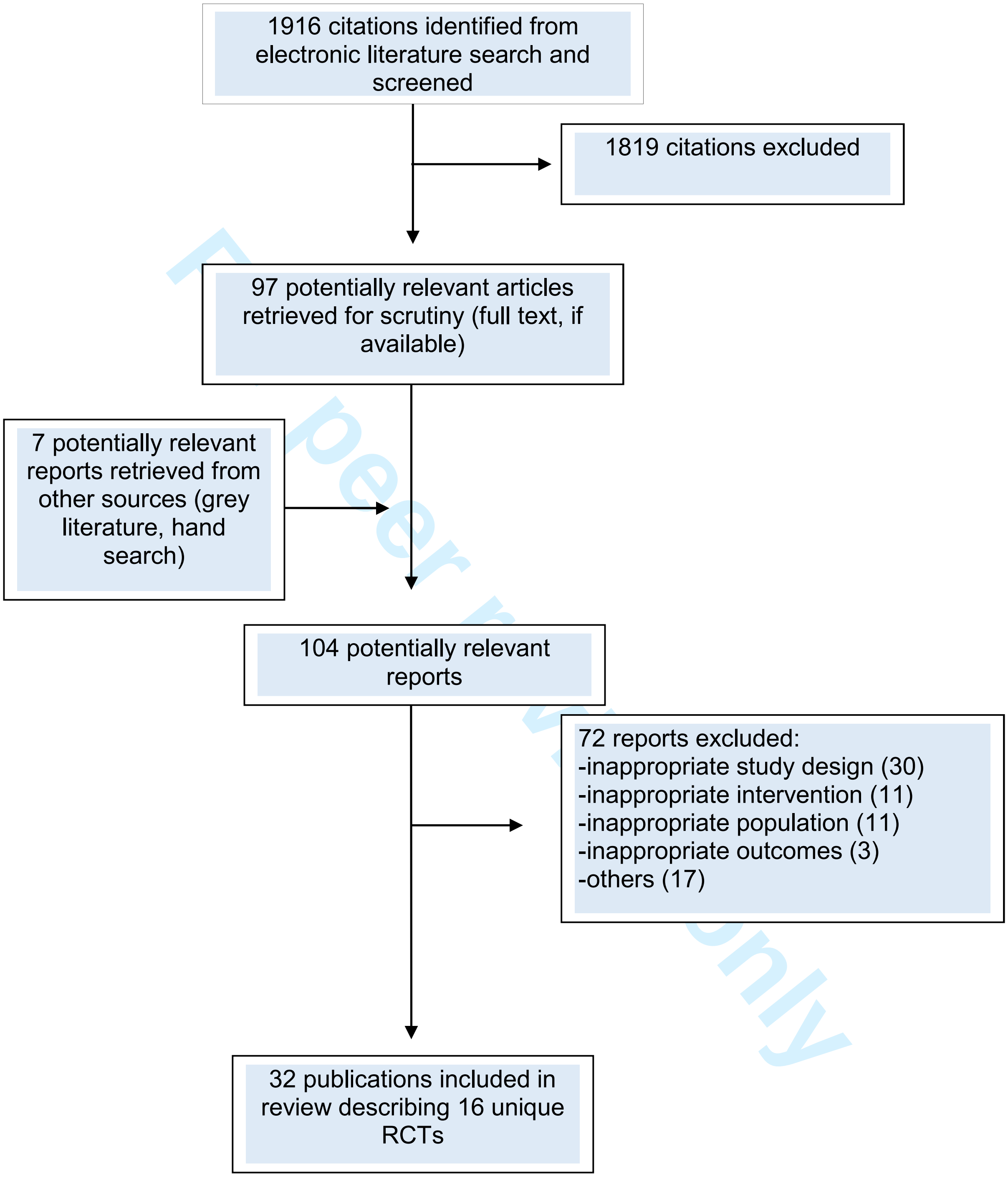

For peer review only

Appendix 3: Summary of Patient Baseline Characteristics

Study	Baseline Characteristics															Centre above TTR Throughout follow-up		
	Age, Mean ± SD (y)	Age Category (%)		Sex (%)		$CHADS_2$, Mean ± SD	$CHADS_2$ Category (%)			Prior Stroke /TIA (%)	DM (%)	Heart Failure (%)	HBP (%)	MI (%)	VKA Naïve (%)	Mean TTR (%)	TTR Category (%)	
		≤75y	>75 y	M	F		0	1	≥2								≤66%	>66%
ACTIVE-W n=6706	70 ± NR	NR	NR	66	34	2 ± 1.1	NR	NR	NR	15	21	30	82	17	23	64	48	52
AFASAK n=672	75 ± NR	NR	NR	54	46	NR ± NR	NR	NR	NR	6	9	53	32	7	NR	N/A	N/A	N/A
AFASAK-2 n=339	73 ± NR	NR	NR	61	39	NR ± NR	NR	NR	NR	8	12	70	45	7	NR	NR	NR	NR
ARISTOTLE n=18201	70 ± NR	69	31	65	35	2.1 ± 1.1	0	34	66	19	25	35	87	14	43	62	NR	NR
ARISTOTLE-J n=222	70 ± NR	69	31	83	17	1.9 ± NR	1	42	57	28	23	1	83	NR	16	60	NR	NR
BAFTA n=973	82 ± 4	0	100	55	45	NR ± NR	NR	NR	NR	13	13	20	54	11	NR	67	NR	NR
CAFA n=378	68 ± NR	NR	NR	75	25	NR ± NR	NR	NR	NR	4	12	22	39	13	NR	44	NR	NR
Chung et al 2011 n=234	65 ± 9	87	13	65	35	1.9 ± 1.1	0	47	53	24	30	29	71	NR	48	45	NR	NR
ENGAGE AF-TIMI 48 n=21105	72 ± NR	60	40	62	38	2.8 ± 1.0	0	0	100	28	36	57	94	12	41	65	52	48
JAST n=871	65 ± NR	NR	NR	70	30	NR ± NR	NR	NR	NR	3	14	9	39	NR	92	N/A	N/A	N/A
PETRO n=236	70 ± NR	70	30	82	18	NR ± NR	NR	NR	NR	18	25	32	71	NR	0	57	NR	NR
RE-LY n=18113	71 ± NR	60	40	64	36	2.1 ± NR	32		68	20	20	23	32	79	17	64	50	50
ROCKET-AF n=14264	73 ± NR	NR	NR	60	40	3.5 ± NR	0	0	100	55	40	62	91	17	38	55	NR	NR
WASPO n=75	83 ± NR	0	100	47	53	NR ± NR	NR	NR	NR	NR	4	NR	47	NR	NR	69	NR	NR
Weitz et al 2010 n=719	65 ± 9	NR	NR	62	38	NR ± NR	0	0	100	NR	NR	NR	NR	NR	66	50	NR	NR

Study	Baseline Characteristics																Centre above TTR Throughout follow-up		
	Age, Mean ± SD (y)	Age Category (%)		Sex (%)		CHADS$_2$, Mean ± SD	CHADS$_2$ Category (%)			Prior Stroke /TIA (%)	DM (%)	Heart Failure (%)	HBP (%)	MI (%)	VKA Naïve (%)	Mean TTR (%)	TTR Category (%)	
		≤75y	>75 y	M	F		0	1	≥2								≤66%	>66%
Yamashita et al 2012 n=385	69 ± NR	71	29	83	17	2.1 ± NR	0	100		28	23	27	73	NR	15	73% FOR <70Y; 83% FOR ≥70Y;	NR	NR

DM = diabetes mellitus; F = female sex; HBP = high blood pressure; M = male sex; MI = myocardial infarction; N/A = not applicable; NR = not reported; SD = standard deviation; TIA = transient ischemic attack; TTR = time in therapeutic range; VKA = vitamin K antagonist; y = year.

Note: Only data from the included relevant treatment arms have been extracted.

For peer review only

Appendix 4: Summary of Included Trials

Study and Design	Disposition	Population	Interventions	Follow-up	Primary Outcome(s)
**ACTIVE-W** OL RCT, 2006. Multicenter, 32 countries (including Europe / America).	Randomized: n=6706	AF patients eligible / willing to take anticoagulant therapy, with ≥ 1 pre-specified risk factor for stroke.	**Clopidogrel / Low-dose ASA** (n=3335)<br>**Warfarin – INR 2.0 to 3.0** (n=3371)	1.3 years (stopped early)	First occurrence of stroke, non-CNS SE, MI or vascular death.
**AFASAK** OL/DB RCT, 1989. Multicenter, Copenhagen.	Randomized: n=1007	AF patients, with no recent anticoagulation or cerebrovascular event, valve replacement or rheumatic heart disease.	***OL Warfarin – INR 2.8 to 4.2 **** *(n=335)*<br>**Low-dose ASA** (n=336)<br>**PL** (n=336)	2 years	Thromboembolic complication: stroke, TIA or embolic complication to viscera / extremities.
**AFASAK-2** OL RCT, 1998. Multicenter, Copenhagen.	Randomized: n=677	AF patients ≥ 60 years (unless with CV or pulmonary disease), without recent TIA or risk factors for bleeding.	***Fixed-dose Warfarin **** *(n=167)*<br>***Fixed-dose Warfarin / Medium-dose ASA **** *(n=171)*<br>**Medium Dose ASA** (n=169)<br>**Warfarin – INR 2.0 to 3.0** (n=170)	3.5 years	Stroke or SE.
**ARISTOTLE** DB RCT, 2011. Multicenter, 39 countries (including Europe / America).	Randomized: n=18201	AF patients with ≥ 1 risk factor for stroke, without recent stroke or ASA > 165 mg.	**Apixaban 5 mg BID** (n=9120)<br>**Warfarin – INR 2.0 to 3.0** (n=9081)<br>Concomitant use of **ASA** (≤ 165 mg daily) or **NSAIDs** permitted.	NR	Stroke or SE;<br>Major bleeding.
**ARISTOTLE-J** OL/DB RCT, 2011. Multicenter, Japan.	Randomized: n=222	AF patients with ≥ 1 risk factor for stroke, without recent TIA or ASA > 100 mg.	**Apixaban 2.5 mg BID** (n=74)<br>**Apixaban 5 mg BID** (n=74)<br>**OL Warfarin – INR 2.0 to 3.0** (n=74)<br>Concomitant use of **ASA** (≤ 100 mg daily) permitted.	12 weeks	Composite of major bleeding and CRNM bleeding events.
**BAFTA** OL RCT, 2007. Multicenter, UK.	Randomized: n=973	AF patients ≥ 75 years, with clinical uncertainty around optimal treatment.	**Low-dose ASA** (n=485)<br>**Warfarin – INR 2.0 to 3.0** (n=488)	2.7 years	Disabling stroke, ICH and other clinically significant arterial embolism.
**CAFA** DB RCT, 1991. Multicenter, Canada.	Randomized: n=378	AF patients without recent stroke, TIA or MI, and who did not require antiplatelet drug therapy.	**Warfarin – INR 2.0 to 3.0** (n=187)<br>**PL** (n=191)	1.2 years (stopped early)	Ischemic stroke, SE, ICH or fatal hemorrhage.

Study and Design	Disposition	Population	Interventions	Follow-up	Primary Outcome(s)
**Chung et al 2011**<br>OL RCT, 2011. Multicenter, 4 countries (including Asia).	Randomized: n=234	AF patients at moderate to high risk of stroke (CHADS2 score ≥ 1).	**Edoxaban 30 mg OD** (n=79)<br>**Edoxaban 60 mg OD** (n=80)<br>**Warfarin – INR 2.0 to 3.0** (n=75)	12 weeks	All bleeding events
**ENGAGE AF-TIMI 48**<br>DB RCT, 2013. Multicenter, 46 countries (including Europe / America).	Randomized: n=21105	AF patients at moderate to high risk of stroke (CHADS2 score ≥ 2).	**Edoxaban 30 mg OD** (n=7034)<br>**Edoxaban 60 mg OD** (n=7035)<br>**Warfarin – INR 2.0 to 3.0** (n=7036)<br>Almost a third of patients in treatment arms took concomitant ASA.	2.8 years	Stroke or SE and major haemorrhage.
**JAST**<br>OL RCT, 2006. Multicenter, Japan.	Randomized: n=871	AF patients without recent symptomatic thromboembolic disease, ICH or GI bleeding.	**Medium-dose ASA** (n=426)<br>**No treatment** (n=445)	2.1 years (stopped early)	CV death, symptomatic brain infarction, or TIA.
**PETRO**<br>OL/DB RCT, 2007. Multicenter, 4 countries (Europe and USA).	Randomized: n=502	AF patients with ≥ 1 risk factor for stroke and with coronary artery disease (removed halfway to facilitate recruitment). No recent MI, stroke, TIA or major bleed. Warfarin pre-treatment required for ≥ 8 weeks.	***Dabigatran 50 mg BID ± Low-dose or High-dose ASA **** *(n=105)*<br>***Dabigatran 300 mg BID ± Low-dose or High-dose ASA **** *(n=161)*<br>**Dabigatran 150 mg BID ± Low-dose or High-dose ASA** † (n=166)<br>**OL Warfarin – INR 2.0 to 3.0** (n=70)	12 weeks	Thromboembolic events and bleeding events.
**RE-LY**<br>OL/DB RCT, 2009. Multicenter, 44 countries (including Europe / America).	Randomized: n=18113	AF patients with ≥ 1 risk factor for stroke, without recent stroke or increased risk of bleeding.	**Dabigatran 110 mg BID** (n=6015)<br>**Dabigatran 150 mg BID** (n=6076)<br>**OL Warfarin – INR 2.0 to 3.0** (n=6022)<br>Concomitant use of **ASA** (<100 mg daily) or other **antiplatelet agents** permitted.	2 years	Stroke or SE and major haemorrhage.
**ROCKET-AF**<br>DB RCT, 2011. Multicenter, 45 countries (including Europe / America).	Randomized: n=14264	AF patients at moderate to high risk of stroke (CHADS2 score ≥ 2).	**Rivaroxaban 20 mg OD** (n=7131)<br>**Warfarin – INR 2.0 to 3.0** (n=7133)<br>Over a third of patients in both arms took concomitant ASA.	1.9 years	Composite of stroke and SE;<br>Composite of major and NMCR bleeding.

For peer review only

Study and Design	Disposition	Population	Interventions	Follow-up	Primary Outcome(s)
**WASPO** OL RCT, 2007. Multicenter, UK.	Randomized: n=75	Ambulant AF patients > 80 and < 90 years with Folstein score > 25, without previous fall, ICH or GI bleeding. Patients with prior stroke or TIA were excluded.	**Warfarin – INR 2.0 to 3.0** (n=36)<br>**Medium-dose ASA** (n=39)	1 year	Combined endpoints: death; stroke, TIA or SE; serious bleeding; withdrawal.
**Weitz et al 2010** DB RCT, 2010. Multicenter, 12 countries (including Eastern Europe / America).	Randomized: n=719	AF patients at moderate to high risk of stroke (CHADS2 score ≥ 2).	**Edoxaban 30 mg OD** (n=235)<br>**Edoxaban 60 mg OD** (n=234)<br>**Warfarin – INR 2.0 to 3.0** (n=250)	12 weeks	Bleeding and abnormalities of hepatic function
**Yamashita et al 2012** DB RCT, 2012. Multicenter, 1 country (Japan).	Randomized: n=385	AF patients at moderate to high risk of stroke (CHADS2 score ≥ 1).	**Edoxaban 30 mg OD** (n=130)<br>**Edoxaban 60 mg OD** (n=130)<br>**Warfarin – INR 2.0 to 3.0** (n=125)	12 weeks	All bleeding events

AF = atrial fibrillation; **ASA =** acetylsalicylic acid; **CNS =** central nervous system; **CRNM =** clinically relevant non-major bleeding; **CV =** cardiovascular; **DB =** double-blind; **GI =** gastrointestinal; **ICH =** intracerebral hemorrhage; **MI =** myocardial infarction; **NR =** not reported; **NSAIDs =** non-steroidal anti-inflammatory drugs; **OL =** open label; **PL =** placebo; **RCT =** randomized controlled trial; **SE =** systemic embolism; **TIA =** transient ischemic attack; **UK =** United-Kingdom; **VKA =** vitamin K antagonist.

* No data was extracted from these treatment arms since they did not meet the inclusion criteria specified in the protocol. These trials were nevertheless included in the systematic review because they had at least one relevant comparison between two interventions of interest.

† Data was extracted only for dabigatran without ASA (n=100).

For peer review only

Appendix 5: Evidence network for major bleeding. The width of the lines is proportional to the number of randomized controlled trials (RCTs) comparing each pair of treatments, and the size of each treatment node is proportional to the number of randomised participants (sample size). A dotted line indicates a three-arm RCT and a black node indicates a node included in the analysis but not reported in the main text.

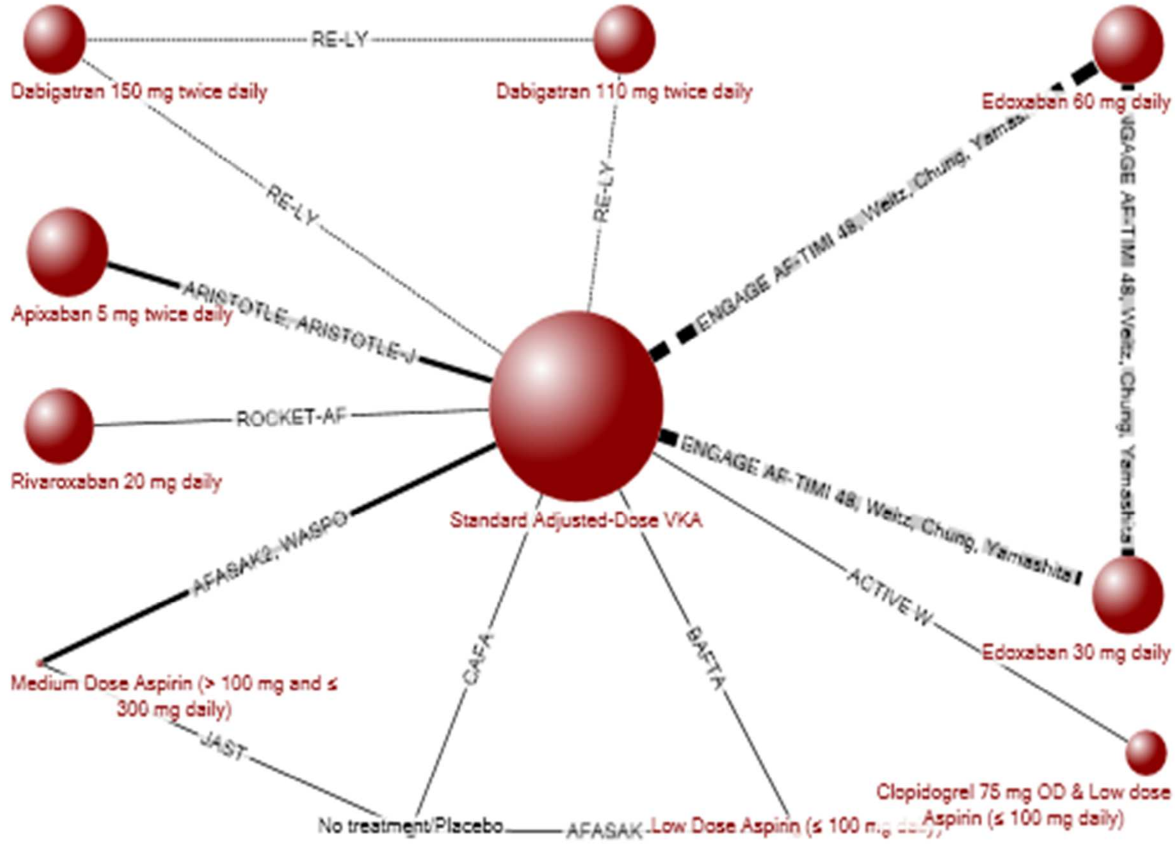

mg= milligram; RCT = randomized controlled trial

Appendix 6: Summary of results from Network Meta-analysis for All-cause Stroke or Systemic Embolism

Treatment	Pair-wise Meta-Analysis, OR (95% CI) versus standard adjusted dose VKA	NMA, Odds Ratio (95% CrI) versus standard adjusted dose VKA	NMA, Absolute Risk Difference per 1,000 treated (95% CrI) versus standard adjusted dose VKA	Probability Best (%) for Outcome	Rank for Outcome	SUCRA
Standard Adjusted-Dose VKA	NA	NA	NA	0.0%	6.0	50.2%
Apixaban 5 mg twice daily	0.79(0.66,0.94)	0.78 (0.65,0.94)	4 fewer (6 fewer-1 fewer)	11.2%	2.5	85.4%
Dabigatran 110 mg twice daily	0.91(0.75,1.11)	0.91 (0.74,1.12)	2 fewer (5 fewer-2 more)	0.1%	4.5	64.7%
Dabigatran 150 mg twice daily	0.66(0.53,0.82)	0.66 (0.53,0.82)	6 fewer (8 fewer-3 fewer)	85.2%	1.2	98.1%
Rivaroxaban 20 mg daily	0.88(0.74,1.05)	0.87 (0.74,1.03)	3 fewer (5 fewer-1 more)	1.0%	3.9	71.2%
Low Dose Aspirin (≤ 100 mg daily)	2.03(1.32,3.12)	1.87 (1.26,2.8)	14 more (5 more-29 more)	0.0%	10.1	9.4%
Medium Dose Aspirin (> 100 mg and ≤ 300 mg daily)	1.21(0.51,2.87)	1.35 (0.74,2.47)	6 more (5 fewer-24 more)	1.2%	7.8	32.0%
Clopidogrel 75 mg OD & Low dose Aspirin (≤ 100 mg daily)	1.93(1.41,2.64)	1.93 (1.42,2.65)	15 more (7 more-26 more)	0.0%	10.2	8.3%
Edoxaban 30 mg daily	1.14(0.94,1.37)	1.14 (0.98,1.32)	3 more (1 fewer-6 more)	0.0%	7.4	36.5%
Edoxaban 60 mg daily	0.87(0.71,1.05)	0.87 (0.74,1.01)	3 fewer (5 fewer-1 more)	1.2%	3.7	72.8%
No treatment/Placebo	1.84(0.66,5.1)	1.53 (0.9,2.63)	9 more (2 fewer-26 more)	0.1%	8.9	21.5%

CI = confidence interval; CrI = credible interval; OR= odds ratio

Bayesian network meta-analysis also allows the analyst to estimate the probability that a treatment is best, second best, and so on for a particular outcome. A simple numerical summary of these probabilities – the surface under the cumulative ranking (SUCRA) – for each treatment can also be calculated [50]. The SUCRA would be 100% when a treatment is certain to be the best and 0% when a treatment is certain to be the worst. SUCRA values enable the ranking of treatments overall for a particular outcome. For example, for Dabigatran 150 mg twice daily for stroke/SE the SUCRA value for is 98.1%, which is better than other treatments. By contrast, Clopidogrel 75 mg OD & Low dose Aspirin (≤ 100 mg daily) has a SUCRA of 8.3% for stroke/SE meaning it is certain to be worst for this outcome among active treatments.

For peer review only

Appendix 7: Summary of results from Network Meta-analysis for Major Bleeding

Treatment	Pair-wise Meta-Analysis, OR (95% CI) versus standard adjusted dose VKA	NMA, Odds Ratio (95% CrI) versus standard adjusted dose VKA	NMA, Absolute Risk Difference per 1,000 treated (95% CrI) versus standard adjusted dose VKA	Probability Best (%) for Outcome	Rank for Outcome	SUCRA
Standard Adjusted-Dose VKA	NA	NA	NA	0.0%	8.0	30.2%
Apixaban 5 mg twice daily	0.69(0.60,0.80)	0.69(0.6,0.8)	10 fewer (13 fewer-7 fewer)	0.0%	3.2	78.1%
Dabigatran 110 mg twice daily	0.8(0.68,0.94)	0.8(0.69,0.93)	7 fewer (10 fewer-3 fewer)	0.0%	4.7	62.7%
Dabigatran 150 mg twice daily	0.93(0.81,1.07)	0.93(0.8,1.08)	3 fewer (7 fewer-3 more)	0.0%	6.8	41.7%
Rivaroxaban 20 mg daily	1.03(0.9,1.18)	1.03(0.89,1.18)	1 more (4 fewer-6 more)	0.0%	8.4	26.3%
Low Dose Aspirin (≤ 100 mg daily)	1.01(0.57,1.78)	1.05(0.6,1.86)	2 more (13 fewer-26 more)	0.1%	7.9	30.6%
Medium Dose Aspirin (> 100 mg and ≤ 300 mg daily)	1.68(0.50,5.69)	1.79(0.63,5.67)	24 more (12 fewer-120 more)	0.0%	10.0	9.8%
Clopidogrel 75 mg OD & Low dose Aspirin (≤ 100 mg daily)	1.1(0.82,1.48)	1.1(0.83,1.47)	4 more (6 fewer-15 more)	0.0%	8.9	20.8%
Edoxaban 30 mg daily	0.46(0.39,0.55)	0.46(0.4,0.54)	18 fewer (20 fewer-15 fewer)	29.7%	1.7	92.9%
Edoxaban 60 mg daily	0.78(0.67,0.92)	0.79(0.69,0.9)	7 fewer (10 fewer-4 fewer)	0.0%	4.5	64.6%
No treatment/Placebo	0.39(0.08,2.02)	0.33(0.08,1.08)	22 fewer (31 fewer-3 more)	70.2%	1.7	92.6%

CI = confidence interval; CrI = credible interval; OR= odds ratio

Bayesian network meta-analysis also allows the analyst to estimate the probability that a treatment is best, second best, and so on for a particular outcome. A simple numerical summary of these probabilities – the surface under the cumulative ranking (SUCRA) – for each treatment can also be calculated [50]. The SUCRA would be 100% when a treatment is certain to be the best and 0% when a treatment is certain to be the worst. SUCRA values enable the ranking of treatments overall for a particular outcome. For example, for major bleeding the SUCRA value for Edoxaban 60 mg is 92.8%, which is better than other treatments. By contrast, Medium Dose Aspirin (> 100 mg and ≤ 300 mg daily) has a SUCRA of 9.9% for major bleeding meaning it is certain to be worst for this outcome.

For peer review only

Appendix 8: Benefit-Harm plot for each treatment (absolute harm difference per 1,000 patients treated). The absolute risk difference of recurrence of Stroke or systemic embolism relative to standard adjusted dose VKA is presented on the x-axis, while the absolute risk difference of major bleeding relative to standard adjusted dose VKA is on the y-axis. The dot is representative of the median estimate for each treatment while the 95% credible intervals are presented as error bars.

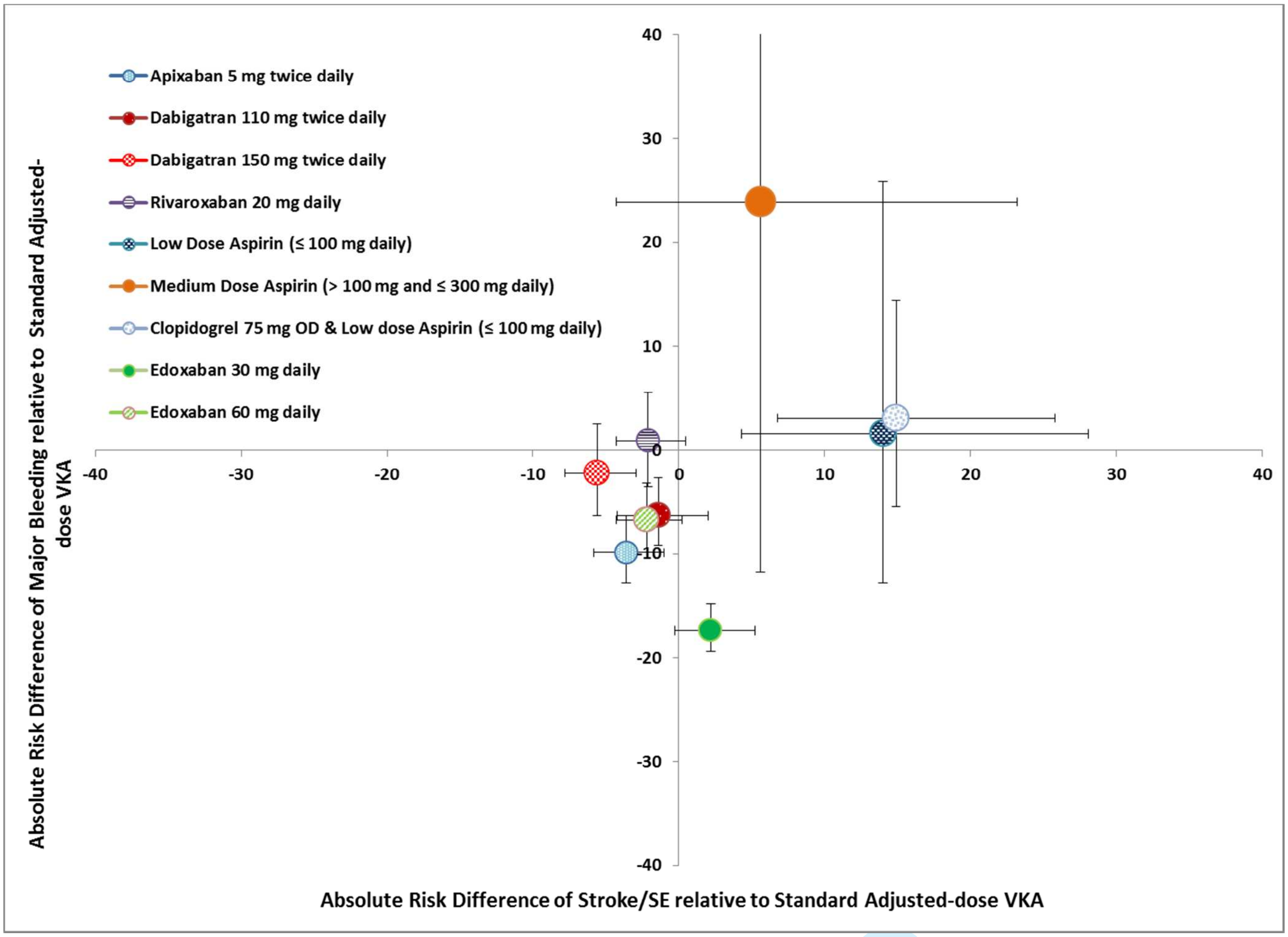

Appendix 9: Rationale of choice of fixed-effects model and limitations

It is challenging to conduct network meta-analyses when evidence structures are comprised of single study connections, especially when there is obvious heterogeneity in the network. The use of a random-effects model with a vague prior on the between study variance exerts a large degree of influence on the credible intervals (CrI's) because there is insufficient studies to reign in the prior and provide an accurate estimate of the between study variance. As a result, results often appear unrealistic (e.g., wide credible intervals for a treatment that was shown to be associated with a statistically significant decrease). Accordingly, we also considered informative priors on the between study variance from the recently published reports.[12,44] However, even when these priors were applied on the between study variance, they still did not reign in CrI's effect estimates again because the network was comprised of single study connections. The use of the random effects model with either a vague or informative prior was problematic because treatments that were originally associated with statistically significant reductions in stroke or SE relative to standard adjusted dose VKA were no longer associated with significant reductions, limiting external validity of the model (see Figures below on direct estimates in Online Appendix 4 and 5). While we could have opted to use less vague priors (e.g., ¼ of prior in Turner et al[12]), the choice of the prior would have seemed somewhat arbitrary because it was not based on any empirical evidence. As such, we opted to use the fixed-effects model despite the known issues in doing so. By using the fixed-effects model, treatments that were associated with significant reductions in the risk of stroke or SE relative to standard adjusted dose VKA retained significance. We felt that it would have been inappropriate to remove statistical significance by applying vague priors given the large investment involved in developing clinical trials. However, a consequence of retaining statistical significance is seen when comparing newer oral anticoagulants versus one another in Figure 5. Because we assumed the new oral anticoagulants were all associated with reductions in Stroke or SE relative to standard adjusted dose VKA (if that was the case in the underlying trials), significant reductions in the risk of stroke or SE among new oral anticoagulants are observed in Figure 5. This may or may not be the case in clinical practice. As noted in tables 1 and Online Appendix 2, there is considerable heterogeneity across studies. However, the network structure itself (i.e., single study connections), made it difficult to properly adjust for differences in patient and study characteristics using standard approaches. Accordingly, results in Figure 5 should be interpreted with caution. We conducted simple sub-group analyses (breaking randomization) to help adjust for this heterogeneity, which is a very crude way to adjust for heterogeneity. To fully elucidate the comparative benefits and harms of antithrombotic agents, especially across the various sub-populations, rigorously conducted comparative studies, or network meta-regression analyses of patient-level data are required.

For peer review only

Forest Plot – Odds ratio (95% Credible interval) when using informative prior on the sd (i.e, dlnorm(-3.02,0.29)[12]

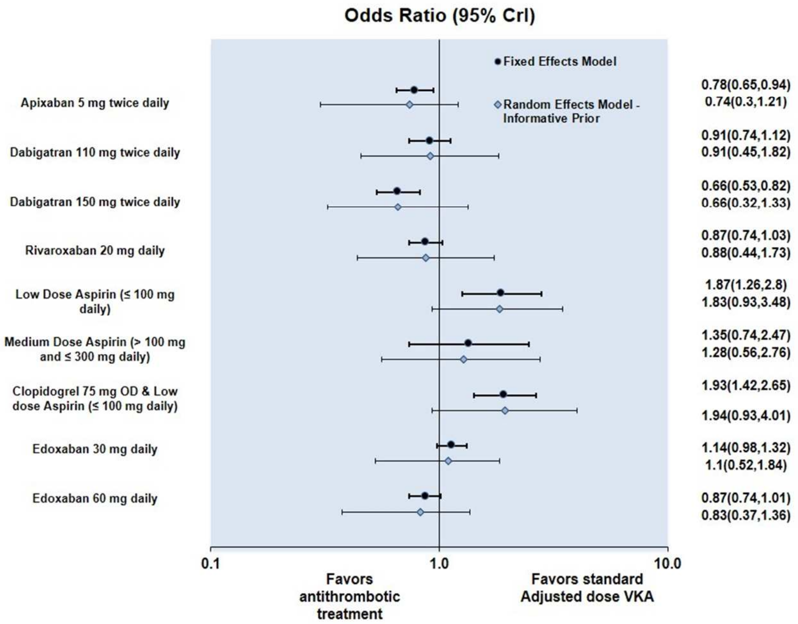

Forest Plot – Odds ratio (95% Credible interval) when using vague prior on the sd (i.e, duniform(0,2))

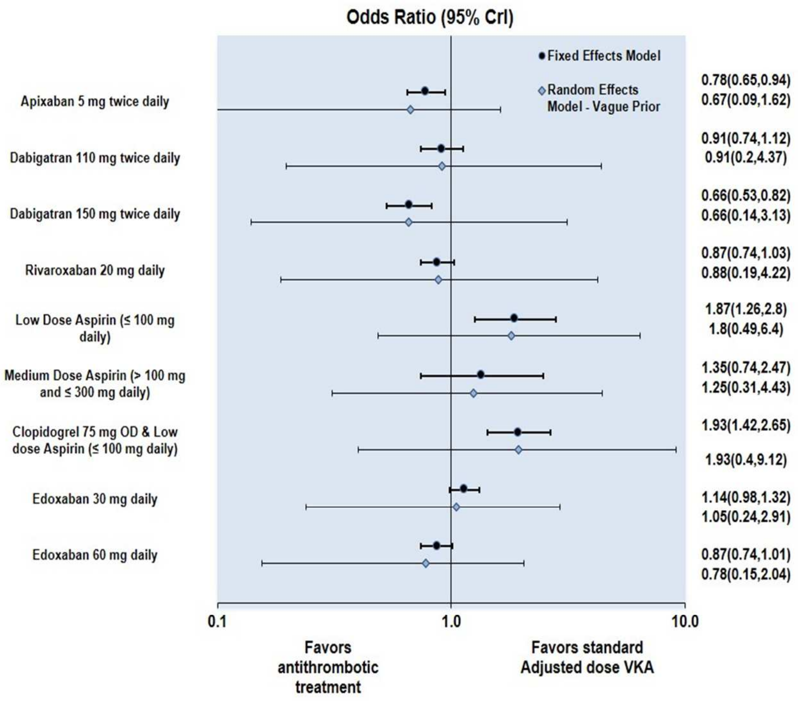

Appendix 10: PRISMA Checklist

Section/topic	#	Checklist item	Reported on page #
**TITLE**			
Title	1	Identify the report as a systematic review, meta-analysis, or both.	0
**ABSTRACT**			
Structured summary	2	Provide a structured summary including, as applicable: background; objectives; data sources; study eligibility criteria, participants, and interventions; study appraisal and synthesis methods; results; limitations; conclusions and implications of key findings; systematic review registration number.	0
**INTRODUCTION**			
Rationale	3	Describe the rationale for the review in the context of what is already known.	1
Objectives	4	Provide an explicit statement of questions being addressed with reference to participants, interventions, comparisons, outcomes, and study design (PICOS).	1
**METHODS**			
Protocol and registration	5	Indicate if a review protocol exists, if and where it can be accessed (e.g., Web address), and, if available, provide registration information including registration number.	2
Eligibility criteria	6	Specify study characteristics (e.g., PICOS, length of follow-up) and report characteristics (e.g., years considered, language, publication status) used as criteria for eligibility, giving rationale.	2-3
Information sources	7	Describe all information sources (e.g., databases with dates of coverage, contact with study authors to identify additional studies) in the search and date last searched.	2-3
Search	8	Present full electronic search strategy for at least one database, including any limits used, such that it could be repeated.	2-3
Study selection	9	State the process for selecting studies (i.e., screening, eligibility, included in systematic review, and, if applicable, included in the meta-analysis).	3
Data collection process	10	Describe method of data extraction from reports (e.g., piloted forms, independently, in duplicate) and any processes for obtaining and confirming data from investigators.	3
Data items	11	List and define all variables for which data were sought (e.g., PICOS, funding sources) and any assumptions and simplifications made.	3-4
Risk of bias in individual studies	12	Describe methods used for assessing risk of bias of individual studies (including specification of whether this was done at the study or outcome level), and how this information is to be used in any data synthesis.	3,6
Summary measures	13	State the principal summary measures (e.g., risk ratio, difference in means).	3
Synthesis of results	14	Describe the methods of handling data and combining results of studies, if done, including measures of consistency (e.g., I^2) for each meta-analysis.	4
Risk of bias across studies	15	Specify any assessment of risk of bias that may affect the cumulative evidence (e.g., publication bias, selective reporting within studies).	5
Additional analyses	16	Describe methods of additional analyses (e.g., sensitivity or subgroup analyses, meta-regression), if done, indicating which were pre-specified.	5
**RESULTS**			

For peer review only

Study selection	17	Give numbers of studies screened, assessed for eligibility, and included in the review, with reasons for exclusions at each stage, ideally with a flow diagram.	3,5
Study characteristics	18	For each study, present characteristics for which data were extracted (e.g., study size, PICOS, follow-up period) and provide the citations.	5-6, Table 1, Online Appendix 2
Risk of bias within studies	19	Present data on risk of bias of each study and, if available, any outcome level assessment (see item 12).	5
Results of individual studies	20	For all outcomes considered (benefits or harms), present, for each study: (a) simple summary data for each intervention group (b) effect estimates and confidence intervals, ideally with a forest plot.	Figure 3-4
Synthesis of results	21	Present results of each meta-analysis done, including confidence intervals and measures of consistency.	Figure 3-4, Online Appendix 4 and 5
Risk of bias across studies	22	Present results of any assessment of risk of bias across studies (see Item 15).	5
Additional analysis	23	Give results of additional analyses, if done (e.g., sensitivity or subgroup analyses, meta-regression [see Item 16]).	Table 2-3, Online Appendix 10
**DISCUSSION**			
Summary of evidence	24	Summarize the main findings including the strength of evidence for each main outcome; consider their relevance to key groups (e.g., healthcare providers, users, and policy makers).	11-12
Limitations	25	Discuss limitations at study and outcome level (e.g., risk of bias), and at review-level (e.g., incomplete retrieval of identified research, reporting bias).	13-14
Conclusions	26	Provide a general interpretation of the results in the context of other evidence, and implications for future research.	10-14
**FUNDING**			
Funding	27	Describe sources of funding for the systematic review and other support (e.g., supply of data); role of funders for the systematic review.	0

For peer review only

For peer review only - http://bmjopen.bmj.com/site/about/guidelines.xhtml

Appendix 11: Assessment of Inconsistency

We plotted the posterior mean deviance of the individual data points in the inconsistency model against their posterior mean deviance in the consistency model to help identify loops where inconsistency is present. In our analysis, the posterior mean deviance contributions are very similar and close to 1, for both models. The consistency model has a similar posterior mean of the residual deviance (29.41 vs. 29.37) and DIC (195.00 vs. 196.87) and hence is a similar fit for both models. A few points show a lower value of the posterior mean deviance in the inconsistency model but these values are not too far from 1. Similarly, a few points have higher values of the posterior mean deviance in the consistency model (again with values not too far from 1) suggesting that each model may fit the data points better for certain points. The parameter estimates are also similar for both models and there is considerable overlap in the 95% credible intervals (see table below), suggesting no evidence of inconsistency in the network.

Plot of posterior mean deviance of the individual data points in the inconsistency model against their posterior mean deviance in the consistency model

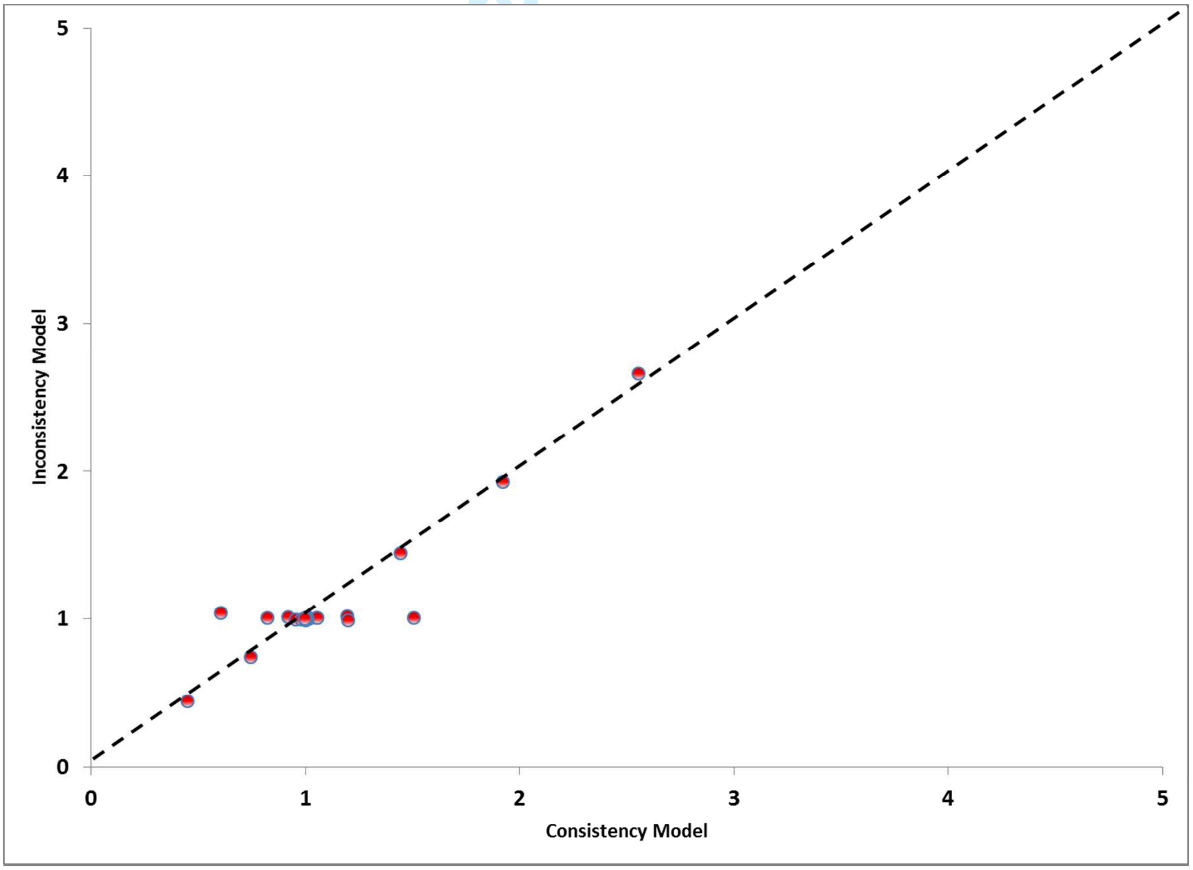

Comparison of effect estimates from consistency and inconsistency models where there is data available for comparison

Comparison	Consistency Model	Inconsistency Model
Apixaban 5 mg twice daily vs Standard Adjusted-Dose VKA	0.78 (0.65,0.94)	0.78 (0.65,0.94)
Dabigatran 110 mg twice daily vs Apixaban 5 mg twice daily vs	0.91 (0.74,1.12)	0.91 (0.74,1.12)
Dabigatran 150 mg twice daily vs Standard Adjusted-Dose VKA	0.66 (0.53,0.82)	0.66 (0.53,0.82)
Rivaroxaban 20 mg daily vs Standard Adjusted-Dose VKA	0.87 (0.74,1.03)	0.88 (0.74,1.03)
Low Dose Aspirin (≤ 100 mg daily) vs Standard Adjusted-Dose VKA	1.87 (1.26,2.8)	2.04 (1.33,3.19)
Medium Dose Aspirin (> 100 mg and ≤ 300 mg daily) vs Standard Adjusted-Dose VKA	1.35 (0.74,2.47)	0.82 (0.33,1.98)
Clopidogrel 75 mg OD & Low dose Aspirin (≤ 100 mg daily) vs Standard Adjusted-Dose VKA	1.93 (1.42,2.65)	1.93 (1.42,2.65)
Edoxaban 30 mg daily vs Standard Adjusted-Dose VKA	1.14 (0.98,1.32)	1.14 (0.98,1.32)
Edoxaban 60 mg daily vs Standard Adjusted-Dose VKA	0.87 (0.74,1.01)	0.87 (0.74,1.01)
No treatment/Placebo vs Standard Adjusted-Dose VKA	1.53 (0.9,2.63)	1.91 (0.7,5.81)
No treatment/Placebo vs Low Dose Aspirin (≤ 100 mg daily)	0.82 (0.48,1.38)	1 (0.51,1.98)
Residual deviance	29.41	29.37
DIC	195.00	196.87

For peer review only

Appendix 12: Sensitivity analysis with AVERROES and ACTIVE A

A. Stroke or SE

Evidence Networks for Stroke or SE NMA including AVEROES and ACTIVE A studies

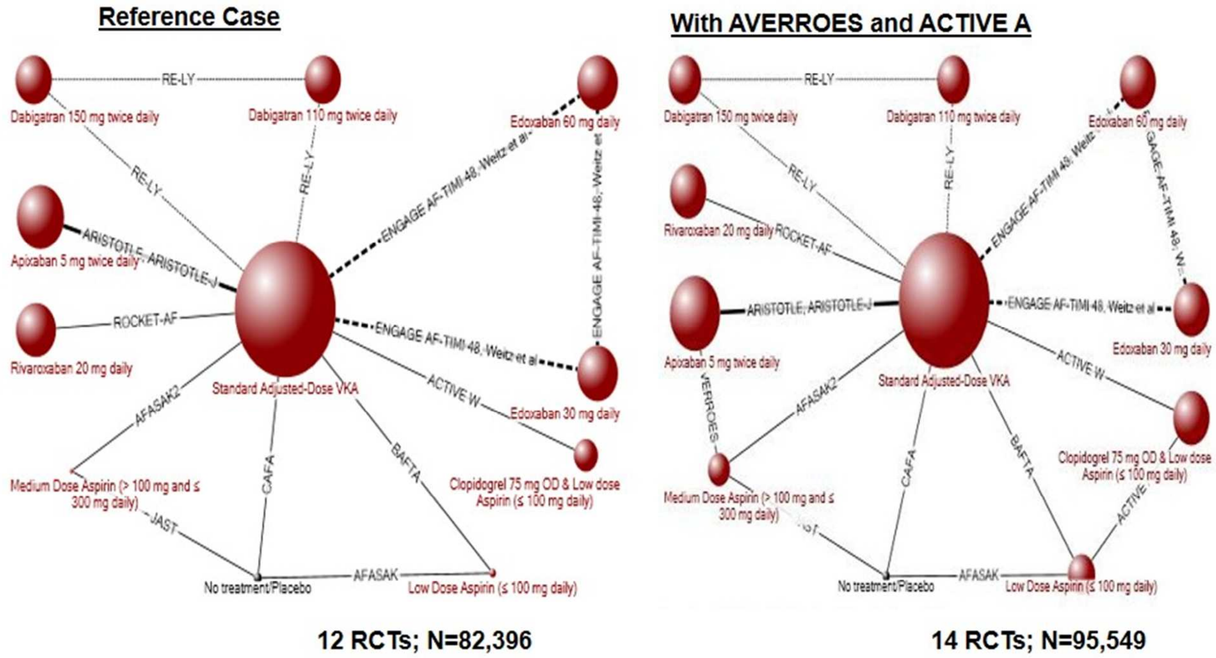

Forest Plot for Stroke or SE NMA including AVERROES and ACTIVE A studies

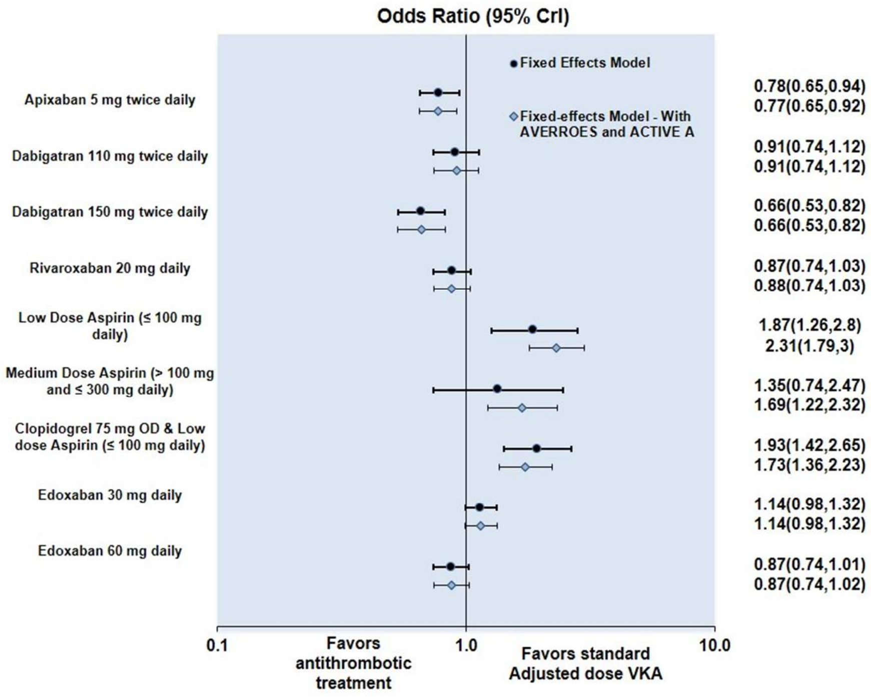

B. Major Bleeding

Evidence Networks for Major Bleeding NMA including AVERROES and ACTIVE A studies

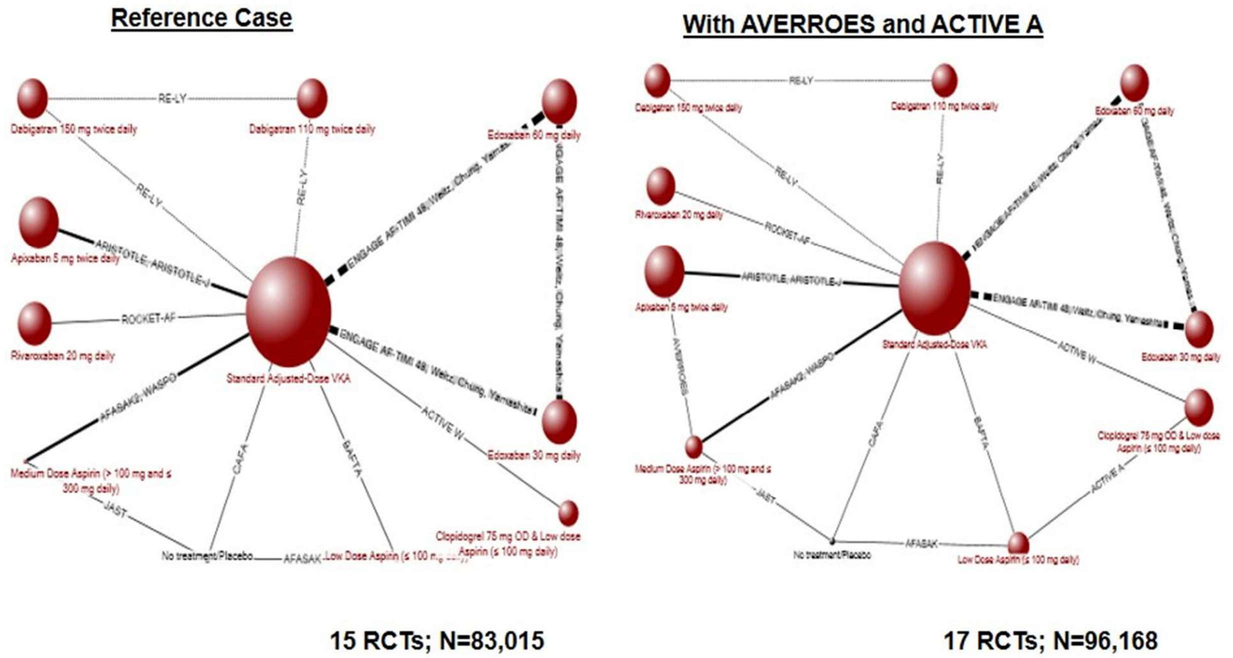

Forest Plot for Major Bleeding NMA including AVERROES and ACTIVE A studies

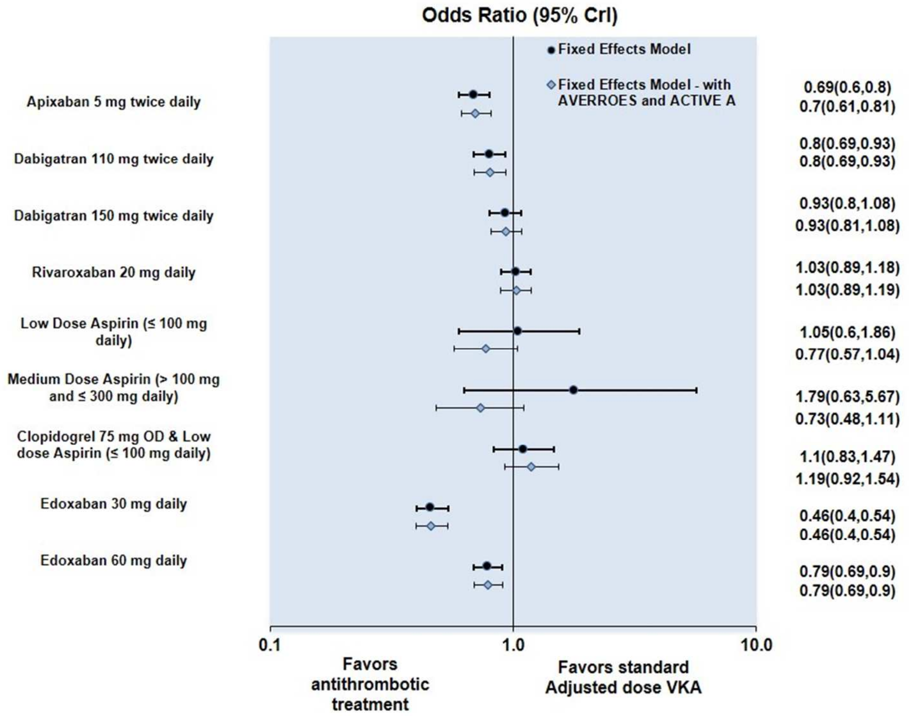

Appendix 13: Comparison with previously published network meta-analyses since Harenberg et al[30]

Author (Publication Year)	Method	Search Date	Included Treatments	Registered Protocol	Relative Measure(s)	Absolute Measure(s) reported	Subgroup Analyses Reported
**Cameron 2014**	Bayesian Network Meta-analysis	January 2014	dabigatran, rivaroxaban, apixaban, edoxaban, and vitamin K antagonists (VKA) at a standard adjusted dose (target international normalised ratio 2.0-3.0), acetylsalicylic acid, acetylsalicylic acid and clopidogrel	Yes (PROSPERO registry - CRD42012002721).	OR (95% CrI)	Yes	Yes
**Ruff 2013**	Traditional Meta-analysis	November 2013	dabigatran, rivaroxaban, apixaban, edoxaban	Not Reported	RR (95%CI)	No	Yes
**Dogliotti 2013**	Bayesian Network Meta-analysis	May 2012	dabigatran, rivaroxaban, apixaban, vitamin K antagonists (VKA) at a standard adjusted dose, acetylsalicylic acid, acetylsalicylic acid and clopidogrel	Yes (metacardio.org)	OR (95% CrI)	No	No
**Assiri 2013**	Baysian Network Meta-analysis	August 2012	dabigatran, rivaroxaban, apixaban, vitamin K antagonists (VKA) at a standard adjusted dose, acetylsalicylic acid, acetylsalicylic acid and clopidogrel	Not Reported	RR (95%CrI)	No	No
**Bruins 2013**	Traditional Meta-analysis	June 2013	antistasin, apixaban, betrixaban, darexaban, DU176b, edoxaban, eribaxaban, fondaparinux, idraparinux, otamixaban, razaxaban, rivaroxaban, yagin, YM150, LY517717, SSR126517E, vitamin K antagonists (warfarin and congeners) with the intensity of anticoagulation dose-adjusted using the International Normalised Ratio (INR)	Yes (Cochrane Library)	OR (95%CI) RR (95%CI) in summary table	Yes	Yes

Appendix 14: Excluded Study List

Inappropriate Study Design

Cardiovasc J S Afr. 2006 Mar;17(2):91.
J Fam Pract. 2006;55(9):753.
Cardiovasc J Afr. 2009 May;20(3):210.
Cardiovasc J Afr. 2009 Sep;20(5):311-2.
Australian Journal of Pharmacy. 2009;90(1073):68.
Formulary. 2010;45(1):16.
Aalbers J. Cardiovasc J Afr. 2010 Nov;21(6):342-3.
Alexander W, et al. PT. 2010;35(10):580-1.
Belavic JM. Nurse Pract. 2011 Sep;36(9):6-7.
Camm AJ. Eur Heart J. 2009 Nov;30(21):2554-5.
Chua D, et al. N Engl J Med. 2009;361(13):1312-3.
Connolly SJ. Eur J Heart Fail. 2009;11(12):1215.
DeSilvey DL. Am J Geriatr Cardiol. 2006 Sep;15(5):326-7.
Dunn A. Ann Intern Med. 2011;154(8):JC4-3.
Eikelboom JW, et al. J Thromb Haemost. 2012;10(5):966-8.
Ezekowitz MD, et al. J Interv Card Electrophysiol. 2011 Dec;32(3):173-80.
Garcia D, et al. Lancet. 2007 Aug 11;370(9586):460-1.
Granger CB. Ann Intern Med. 2012;156(JC1-2):JC1-3.
Hart RG, et al. Ann Intern Med. 2007;147(8):590-2.
Katona A, et al. Eur Heart J. 2006 Jun;27(11):1382.
Kita K, et al. Interventional Cardiology. 2011;3(6):637.
Lopes RD, et al. Evid Based Med. 2011;16(6):187-8.
Nolan J, et al. Br Heart J. 1992 Dec;68(6):544-7.
Paikin JS, et al. Expert Rev Cardiovasc Ther. 2011 Mar;9(3):279-86.
Panichpisal K, et al. Future Neurology. 2011;6(2):155-8.
Roy B, et al. Am J Cardiol. 2012 Feb 1;109(3):370-7.
Sloan MA. Evid Based Med. 2006;11(6):170.
Vassiliou VS. N Engl J Med. 2012;366(1):88-90.
Veloso HH, et al. Chest. 2005 Jul;128(1):475.
Weinberger J. Curr Cardiol Rep. 2010;12(1):1-2.

Inappropriate Intervention

Lancet. 1994 Mar 19;343(8899):687-91.
The Stroke Prevention in Atrial Fibrillation Investigators. Arch Intern Med. 1996 Feb 26;156(4):409-16.
Archer SL, et al. Am Heart J. 1995 Aug;130(2):287-95.
Edvardsson N, et al. J Intern Med. 2003 Jul;254(1):95-101.
Hart RG, et al. Cerebrovasc Dis. 2000 Jan;10(1):39-43.
Hellemons BS, et al. Control Clin Trials. 1999 Aug;20(4):386-93.
Hori M, et al. Circ J. 2012 Jun 5.
Miller VT, et al. Neurology. 1996 Jan;46(1):238-40.
Perez-Gomez F, et al. Eur Heart J. 2007;28(8):996-1003.

For peer review only

Singer DE, et al. Am Heart J. 1992 Dec;124(6):1567-73.
Connolly S, et al. Eur Heart J. 2013 May:34(20):1498-505

Inappropriate Population
ACTIVE Investigators, et al. N Engl J Med 2009;360(20):2066-78.
Circulation 1991;84(2):527-39.
Connolly SJ, et al. N Engl J Med 2011;364(9):806-17.
Dewilde W, et al. Am Heart J. 2009 Nov;158(5):713-8.
Diener HC, et al. Lancet Neurol. 2012 Mar;11(3):225-31.
EAFT (European Atrial Fibrillation Trial) Study Group. Lancet. 1993;342(8882):1255-62.
Eikelboom JW, et al. J Stroke Cerebrovasc Dis. 2012 Aug;21(6):429-35.
ESPRIT Study Group, et al. Lancet Neurol. 2007 Feb;6(2):115-24.
Hart RG, et al. Cerebrovasc Dis. 2008;25(4):344-7.
N Engl J Med 1990;322(12):863-8.
Wang TH, et al. Eur Heart J. 2007 Sep;28(18):2200-7.

Inappropriate Outcomes
De Caterina R, et al. Eur Heart J. 2010 Sep;31(17):2133-40.
Ezekowitz MD, et al. Circulation. 1995 Oct 15;92(8):2178-82.
Lorenzoni R, et al. Am Heart J. 2004 Jul;148(1):e6.

Others
Cardiovasc J Afr. 2011 May;22(3):164.
Active Steering Committee, et al. Am Heart J. 2006 Jun;151(6):1187-93.
ROCKET AF Study Investigators. Am Heart J. 2010 Mar;159(3):340-7.
The Stroke Prevention in Atrial Fibrillation Investigators. Stroke. 1990 Apr;21(4):538-45.
Boysen G, et al. Stroke. 1990;21(Suppl I):I-92.
De Schryver EL. Cerebrovasc Dis. 2000 Mar;10(2):147-50.
Desai A. Clinical Trials Registry - India (CTRI). 2006.
Eikelboom JW, et al. Am Heart J. 2010 Mar;159(3):348-53.
Ezekowitz MD, et al. Am Heart J. 2009 May;157(5):805-10, 810.e1-2.
Flaker G, et al. J Am Coll Cardiol. 2012 Feb 28;59(9):854-5.
Lopes RD, et al. Lancet. 2012 Oct 2. Epub ahead of print.
Lopes RD, et al. Am Heart J. 2010 Mar;159(3):331-9.
Mant J, et al. Cerebrovasc Dis. 2007;23(Suppl 2):10.
Mant J, et al. Cardiol Rev. 2008;25(7):32-6.
Mant JW, et al. BMC Cardiovasc Disord. 2003 Aug 26;3:9.
Posada IS, et al. Am Heart J. 1999 Jul;138(1 Pt 1):137-43.
Van Latum JC. Ned Tijdschr Geneeskd. 1994;138(20):1025-31.
Yigit Z. Turk Kardiyoloji Dernegi Arsivi. 2000;28(1):8-19+4.

For peer review only

BMJ Open

Systematic Review and Network Meta-analysis comparing Antithrombotic Agents for the Prevention of Stroke and Major Bleeding in Patients with Atrial Fibrillation

Journal:	*BMJ Open*
Manuscript ID:	bmjopen-2013-004301.R3
Article Type:	Research
Date Submitted by the Author:	06-May-2014
Complete List of Authors:	Cameron, Chris; University of Ottawa, Department of Epidemiology and Community Medicine<br>Coyle, Douglas; University of Ottawa, Epidemiology and Community Medicine<br>Richter, Trevor; Canadian Agency for Drugs and Technologies in Health,<br>Kelly, Shannon; Ottawa Heart Institute,<br>Gauthier, Kasandra; Canadian Agency for Drugs and Technologies in Health,<br>Steiner, Sabine; Medical University of Vienna, Department of Internal Medicine/Cardiology<br>Carrier, Marc; Ottawa Hospital Research Institute, Medicine<br>Coyle, Kathryn; Applied Health Economics Research Unit,<br>Bai, Annie; Canadian Agency for Drugs and Technologies in Health,<br>Moulton, Kristen; Canadian Agency for Drugs and Technologies in Health,<br>Clifford, Tammy; University of Ottawa, Department of Epidemiology and Community Medicine; University of Ottawa, Department of Pediatrics<br>Wells, George ; University of Ottawa, Department of Epidemiology and Community Medicine; University of Ottawa Heart Institute,
**Primary Subject Heading**:	Cardiovascular medicine
Secondary Subject Heading:	Epidemiology, Health policy
Keywords:	Stroke < NEUROLOGY, CARDIOLOGY, EPIDEMIOLOGY

SCHOLARONE™
Manuscripts

Systematic Review and Network Meta-analysis comparing Antithrombotic Agents for the Prevention of Stroke and Major Bleeding in Patients with Atrial Fibrillation

Chris Cameron, PhD Student[1,2], Doug Coyle, Professor[1], Trevor Richter, Manager of Clinical Research [3], Shannon Kelly, Research coordinator[2] , Kasandra Gauthier, Clinical Research Officer[3], Sabine Steiner, Associate Professor[4], Marc Carrier, Associate Professor[6], Kathryn Coyle, Health Economist[5], Annie Bai , Clinical Research Officer[3], Kristen Moulton, Clinical Research Officer[3], Tammy Clifford, Adjunct Professor[1,7], George Wells, Professor[1,2]

[1] Department of Epidemiology and Community Medicine, University of Ottawa, Ottawa, Canada
[2] University of Ottawa Heart Institute, Ottawa, Canada
[3] Canadian Agency for Drugs and Technologies in Health, Ottawa, Canada
[4] Medical University of Vienna, Vienna, Austria
[5] Applied Health Economics Research Unit, Ottawa, Canada
[6] Thrombosis Program, Division of Hematology, Department of Medicine, University of Ottawa, Ottawa, Canada
[7] Department of Pediatrics, University of Ottawa, Ottawa, Canada

Word count, abstract: 322
Word count, body of essay: 3,987
Number of figures: 4
Number of tables: 1
Number of References: 44
Supplementary Material: 14

Corresponding author:
Dr. George Wells
Professor and Director, Cardiovascular Research Methods Centre
Department of Epidemiology and Community Medicine, University of Ottawa
Ottawa Heart Institute
40 Ruskin Street, Ottawa, Ontario, Canada K1Y 4W7
Phone: (613) 798-5555, ext. 18640
Fax: (613) 761-4412
Email: gawells@ottawaheart.ca

Keywords: atrial fibrillation, antithrombotic, anticoagulants, stroke, bleeding

For peer review only

Abstract

Objective: To examine the comparative efficacy and safety of antithrombotic treatments (apixaban, dabigatran, edoxaban, rivaroxaban, and vitamin K antagonists (VKA) at a standard adjusted dose (target international normalised ratio 2.0-3.0), acetylsalicylic acid, acetylsalicylic acid and clopidogrel) for non-valvular atrial fibrillation and among sub-populations.

Design: Systematic review and network meta-analysis.

Data sources: A systematic literature search strategy was designed and carried out using MEDLINE, EMBASE, Cochrane Register of Controlled Trials, and grey literature including the websites of regulatory agencies and health technology assessment organizations for trials published in English from 1988 to January 2014.

Eligibility criteria for selecting studies: Randomized controlled trials were selected for inclusion if they were published in English, included at least one antithrombotic treatment and involved patients with non-valvular atrial fibrillation eligible to receive anticoagulant therapy.

Results: For stroke or systemic embolism, dabigatran 150 mg and apixaban twice daily were associated with reductions relative to standard adjusted dose VKA whereas low-dose ASA and the combination of clopidogrel plus low-dose ASA were associated with increases. Absolute risk reductions ranged from 6 fewer events per 1,000 patients treated for dabigatran 150 mg twice daily to 15 more events for clopidogrel plus ASA. For major bleeding, edoxaban 30 mg daily, apixaban, edoxaban 60 mg daily, and dabigatran 110 mg twice daily were associated with reductions compared to standard adjusted dose VKA. Absolute risk reductions with these agents ranged from 18 fewer per 1,000 patients treated each year for edoxaban 30 mg daily to 24 more for medium dose ASA.

Conclusions: Compared with standard adjusted dose VKA, new oral anticoagulants were associated with modest reductions in the absolute risk of stroke and major bleeding. People on antiplatelet drugs experienced more strokes compared with anticoagulant drugs without any reduction in bleeding risk. To fully elucidate the comparative benefits and harms of antithrombotic agents across the various sub-populations, rigorously conducted comparative studies or network meta-regression analyses of patient-level data are required.

(PROSPERO registry - CRD42012002721).

For peer review only

Article Summary

Strengths and limitations of this study

- This network meta-analysis includes data on edoxaban from the recently published ENGAGE AF-TIMI 48 trial
- The studies pooled in this meta-analysis include antiplatelet agents in addition to newer oral anticoagulants and standard adjusted dose vitamin K antagonists
- We present findings on both the relative and absolute scale, and graphically illustrate absolute risks using icon arrays
- We report detailed *a priori* sub-group analyses for stroke or systemic embolism and major bleeding using data derived from FDA Public Summary Documents
- Comparisons between antithrombotic treatments were largely comprised of single studies, albeit large studies
- There is notable heterogeneity and the small number of studies limits the analyses that can be conducted to account for heterogeneity in the absence of patient-level data
- Icon arrays only consider the primary efficacy and safety endpoints of underlying studies and do not account for different clinical consequences associated with each of the outcomes

For peer review only

INTRODUCTION

Atrial fibrillation (AF) is a common cardiac arrhythmia associated with increased morbidity and mortality.[1,2] Patients with AF sustain an increased risk of arterial thromboembolism and stroke, associated with high recurrence and substantial debilitating impact. Therefore, antithrombotic strategies using anticoagulant drugs and antiplatelet agents are recommended for AF patients presenting with risk factors for stroke. Antithrombotic therapy is also associated with a risk of bleeding; therefore, the beneficial effects on stroke prevention should always be compared against a patient's risk of major bleeding.[1,2]

Existing guidelines recommend anticoagulant therapy for patients at intermediate or high risk of stroke.[1,2] Although standard adjusted dose vitamin K antagonists (VKA) (e.g., warfarin) has been the cornerstone treatment for reducing the risk of stroke or systemic embolism (SE) in this population, it is associated with several drawbacks.[2] which have prompted the development of newer oral anticoagulants such as direct thrombin (dabigatran) and Xa (e.g. apixaban, edoxaban rivaroxaban) inhibitors. [1,2] While some of these newer treatment options have been demonstrated to be efficacious in preventing stroke or SE compared to standard adjusted dose VKA, the relative efficacy and risk of bleeding of newer oral anticoagulants, both among themselves and in comparison to antiplatelet agents, is not clear, especially in certain sub-populations of patients, e.g., $CHADS_2$ score <2 or ≥2; time in therapeutic range (TTR) < 66% or ≥ 66%; Age <75 or ≥ 75 years. Therefore, the objective of this paper was to compare antithrombotic agents for the prevention of stroke and major bleeding in patients with non-valvular AF and among sub-populations.

For peer review only - http://bmjopen.bmj.com/site/about/guidelines.xhtml

METHODS

Published literature was identified by searching MEDLINE, MEDLINE In-Process & Other Non-Indexed Citations, EMBASE, BIOSIS Previews, PubMed and the Cochrane Central Register of Controlled Trials were searched through the Ovid interface to identify English-language clinical articles published from 1988 to January 23, 2014. Regular alerts were also established. Websites of regulatory agencies, Canadian and major international health technology assessment agencies, clinical practice guidelines as well as The Cochrane Library (2012, Issue 6) and University of York Centre for Reviews and Dissemination databases were searched. Complete details of the electronic search strategy, including any limits used, are reported in Appendix 1. The protocol was published online and was registered in the PROSPERO International Prospective Register of Systematic Reviews (PROSPERO registry - CRD42012002721).

The population of interest was individuals with non-valvular AF requiring anticoagulation (including all risk levels and regardless of any comorbidities). The following treatments were included in the review: apixaban, dabigatran, edoxaban, rivaroxaban, standard adjusted dose VKA, acetylsalicylic acid (ASA), ASA and clopidogrel. Numerous outcomes were considered and results are reported elsewhere;[3] however, the focus of this publication is the outcomes of all-cause stroke or SE and major bleeding as defined by the International Society on Thrombosis and Haemostasis [ISTH] definition),[4] which were the primary efficacy and safety outcomes used in the newer oral anticoagulant studies.

For peer review only - http://bmjopen.bmj.com/site/about/guidelines.xhtml

Active and placebo-controlled randomized controlled trials (RCTs) were selected for inclusion if they were published in English, included at least one antithrombotic treatment under review (using pre-specified doses), reported data for stroke/SE or major bleeding, and involved patients with non-valvular AF eligible to receive anticoagulant therapy, regardless the level of stroke risk. Trials that included patients with contraindication to anticoagulant treatment were excluded. VKA trials were included if the dose was adjusted to a target international normalised ratio (INR) 2.0-3.0. Any dose of ASA was considered for inclusion, but ASA dose was stratified in the analysis as low (≤ 100 mg daily), medium (>100 mg to ≤ 300 mg daily), or high (>300 mg daily). We only included new oral anticoagulants which had at least one large phase III study. Three reviewers independently extracted the data on baseline characteristics, intervention(s) evaluated, including dose, duration, and relevant co-medication and results for each included article, using a standardized template. All extracted data was checked for accuracy by three independent reviewers. Any disagreements in the assessment of these data were resolved through discussion until consensus was reached. Quality assessment of RCTs was also performed independently by two reviewers using a standardized table based on major items from the SIGN 50 instrument for internal validity.[5] The trial selection process is presented in a flowchart based on the Preferred Reporting Items for Systematic Reviews and Meta-Analyses (PRISMA)[6] statement (Appendix 2).

Bayesian network meta-analyses were conducted using WinBUGS software (MRC Biostatistics Unit, Cambridge, UK). A binomial likelihood model[7] which accounts for the use of multi-arm trials was used for the analyses given the datasets provided were dichotomous outcomes and included multi-arm trials. Trials with zero cells in both arms or nodes where there were no events

For peer review only - http://bmjopen.bmj.com/site/about/guidelines.xhtml

were excluded from the evidence networks because they do not contribute information or allow interpretable information.[7] Both fixed and random-effects network meta-analyses were conducted, although the fixed-effects model was chosen for the reference case analysis, as the evidence network was largely comprised of single study connections. We modeled point estimates and 95% credible intervals for odds ratios (OR) using Markov Chain Monte Carlo methods. The absolute risk difference per 1000 patients treated each year for each outcome was also calculated, based on the standard adjusted dose VKA arm of the Randomized Evaluation of Long-Term Anti-coagulation Therapy (RE-LY) trial.[8] The RE-LY trial[8] was selected because it contained data for both $CHADS_2<2$ and $CHADS_2\geq2$ and had detailed data available from the Food and Drug Administration (FDA) Public Summary Report.[9] We also assessed the probability that each treatment was the most efficacious regimen, the second best, the third best, and so on.[10] Vague or flat priors, such as $N(0, 100^2)$ were assigned for basic parameters throughout, although informative priors were considered.[11] Assessment of model fit was based on deviance information criterion (DIC) and comparison of residual deviance to number of unconstrained data points.[7,12] To ensure convergence was reached, trace plots and the Brooks-Gelman-Rubin statistic were assessed.[13] Three chains were fit in WinBUGS for each analysis, with at least 40,000 iterations, and a burn-in of at least 40,000 iterations. We also qualitatively compared the results from our network meta-analysis with direct pair-wise estimates. Frequentist pair-wise meta-analyses were conducted using R (R Language and Environment for Statistical Computing, Vienna, Austria) and package meta.[14]

A key assumption behind network meta-analysis is that the analysed network is consistent, i.e., there is no conflict between direct and indirect evidence.[15] To assess inconsistency, we compared

For peer review only - http://bmjopen.bmj.com/site/about/guidelines.xhtml

deviance and DIC statistics in fitted consistency and inconsistency models.[15] We also plotted the posterior mean deviance of the individual data points in the inconsistency model against their posterior mean deviance in the consistency model to identify any loops in where inconsistency is present (Appendix 11).[15] Additionally, the results from our network meta-analysis were qualitatively compared with direct frequentist pair-wise estimates.

Network meta-analysis also requires that studies are sufficiently similar in order to pool their results.[16] Available study and patient characteristics were assessed to ensure similarity and to investigate the potential impact of heterogeneity on effect estimates. Clinical and methodological heterogeneity was identified in a number of areas. The issues identified were similar to those reported in previous systematic reviews of anticoagulant drugs (e.g., differences in TTR, $CHADS_2$ score).[17] Heterogeneity was assessed by conducting network meta-analysis with the following pre-specified subgroup data reported in the individual RCTs: TTR $< 66\%$ or $\geq 66\%$; $CHADS_2$ score <2 or ≥ 2; Age <75 or ≥ 75 years.[17] The data to conduct these sub-group analyses were derived from FDA Public Summary Documents when not available in the publications.[9,18,19] Secondary evidence networks were constructed for each of the sub-group analyses. The WinBUGS code[7] and data needed to replicate all analyses are available online.

This research was supported through a grant by the Canadian Institute of Health Research (CIHR) Drug Safety and Effectiveness Network and financial support from the Canadian Agency for Drugs and Technologies in Health (CADTH).

For peer review only

RESULTS

Study Characteristics of included studies

The systematic review included 16 individual RCTs (reported in 32 publications and FDA reports); all evaluated the efficacy and safety of antithrombotic agents: apixaban (5 mg twice daily), dabigatran (150 or 110 mg twice daily), edoxaban (30 or 60 mg daily), rivaroxaban (20 mg daily), standard adjusted dose VKA, ASA (low dose (< 100 mg daily); medium dose (100-300 mg daily), or low dose-ASA plus clopidogrel (75 mg daily) in patients with non-valvular AF. None of the included studies directly compared one new oral anticoagulant drug with another (Appendix 4). Of the 82,396 randomized patients included in the primary analysis, five large multicentre trials account for 78,296 patients (96%): ENGAGE AF-TIMI 48 (n=21,105),[20] ARISTOTLE (n=18,201),[21] RE-LY (n=18,113),[8] ROCKET-AF (n=14,264)[22] and ACTIVE-W (n=6706).[23] Most trials included stroke and SE as a primary efficacy outcome, while bleeding events were a frequent safety outcome. Follow-up ranged from 12 weeks to 3.5 years. All trials published results between 1989 and 2013. Mean $CHADS_2$ score was reported in eight trials, encompassing the vast majority of patients included in the systematic review. Reported $CHADS_2$ scores were consistent with a high-risk population ($CHADS_2 \geq 2$), with patients from ROCKET-AF[22] showing the highest risk for stroke (mean $CHADS_2$ = 3.5).

Mean age across the included trials ranged from 62 years to 83 years (Appendix 3). All trials included patients of both gender and had a higher proportion of male participants in most studies. However, the proportions of patients with a prior stroke or TIA varied substantially across the included trials, ranging from 3% in JAST to 55% in ROCKET-AF.[22] The same observation can be made regarding the proportions of patients with other concomitant conditions, i.e. congestive

For peer review only - http://bmjopen.bmj.com/site/about/guidelines.xhtml

heart failure, hypertension, diabetes, myocardial infarction, and prior vitamin K antagonist experience. TTR was reported in all but one trial that included a standard adjusted dose VKA treatment arm. Patient baseline characteristics are summarized in Appendix 3.

The studies were individually critically appraised and the details are reported elsewhere.[3] Overall, there was substantial variation in study quality. However, large multicentre trials comparing newer anticoagulants with standard adjusted dose VKA such as ENGAGE AF-TIMI 48 (n=21,105), ARISTOTLE (n = 18,201), RE-LY (n = 18,113), and ROCKET-AF (n = 14,264), which account for the vast majority of patients included in the systematic review, appear to be methodologically rigorous.

Stroke or Systemic Embolism

The evidence network (Figure 1) for the primary analysis was comprised of twelve RCTs representing eight treatments in addition to placebo/observation (N =82,396). Four RCTs (Chung et al,[26] PETRO,[24] WASPO[25], and Yamashita et al[27]) were not included in the analysis because they did not report data for this outcome or had zeroes in both arms.

Dabigatran (150 mg twice daily) and apixaban were associated with reductions in stroke or SE relative to standard adjusted dose VKA (Figure 2). The use of these two agents led to absolute risk reductions ranging from 4 to 6 fewer events per 1,000 patients treated each year. By contrast, low-dose ASA and the combination of clopidogrel plus low-dose ASA appeared to have a higher risk of stroke or SE than standard adjusted dose VKA, leading to an increase in the number of stroke or SE ranging from 14 to 15 more events per 1,000 patients treated each year

For peer review only - http://bmjopen.bmj.com/site/about/guidelines.xhtml

(Figure 4 and Appendix 6). No differences were detected between standard adjusted dose VKA and each of the following interventions: dabigatran (110 mg twice daily), edoxaban (30 mg or 60 mg daily), rivaroxaban, and medium-dose ASA (Figure 3).

The estimates of effect derived from the direct pair-wise comparisons aligned well with those obtained from the network meta-analysis in both direction and magnitude (Appendix 6). Furthermore, the posterior mean residual deviance (29.41) is close to the number of unconstrained data points (27), which is an indication of reasonable model fit. A number of sub-group analyses and alternative modeling strategies were conducted on the primary analysis, including adjusting for $CHADS_2$, TTR, and age (Table 1). We also conducted a sensitivity analysis where we included the AVERROES[28] and ACTIVE A[29] trials (Appendix 12). These studies were excluded from the main analysis because we required patients to be eligible for anticoagulant therapy, including treatment with a VKA. The results differed slightly from the reference case (Appendix 12). The primary analysis was conducted using a fixed-effects model because the network was comprised of single study connections; these results were also compared against those obtained using a random-effects model with informative and vague priors on the standard deviation, and found to be similar in magnitude although wider credible intervals were observed (Appendix 9).

Major bleeding

The evidence network for the primary analysis for major bleeding was comprised of 15 RCTs representing eight treatments in addition to placebo/observation (N =83,015). The evidence network for major bleeding is similar to the evidence network for stroke or SE (Figure 1) but

For peer review only - http://bmjopen.bmj.com/site/about/guidelines.xhtml

includes one extra RCT (WASPO[25]) comparing medium dose ASA with standard adjusted dose VKA, and two extra RCTs (Chung et al,[26] and Yamashita et al[27]) comparing edoxaban with standard adjusted dose VKA (Appendix 5).

Edoxaban 30 mg daily, apixaban, edoxaban 60 mg daily, and dabigatran 110 mg twice daily were associated with reductions in the risk of major bleeding compared with standard adjusted dose VKA (Figure 2). No differences for major bleeding were detected between standard adjusted dose VKA and each of the remaining interventions: dabigatran 150 mg twice daily, rivaroxaban, clopidogrel plus low-dose ASA, and all ASA dosages (Figure 2). The absolute risk difference of major bleeding relative to standard adjusted dose VKA ranged from 18 fewer to 24 more events per 1000 patients treated per year **(**Figure 4 and Appendix 7**)**. A summary of the results for the Bayesian and direct pair-wise meta-analyses is shown in Appendix 7, and Bayesian network meta-analysis results are represented graphically in Figure 2. Complete results from the Bayesian network meta-analysis for all possible comparisons are presented in Figure 3**.**

The estimates of effect derived from the direct pair-wise comparisons aligned well with those obtained from the network meta-analysis in both direction and magnitude (Appendix 7). Furthermore, the posterior mean residual deviance (38.0) is close to the number of unconstrained data points (35), which is an indication of reasonable model fit. A number of sub-group analyses and alternative modeling strategies were conducted on the primary analysis (Table 1). We also conducted a sensitivity analysis where we included the AVERROES[28] and ACTIVE A[29] trial (Appendix 12), as well as analyses using a random-effects model with vague and informative priors (Appendix 9).

For peer review only - http://bmjopen.bmj.com/site/about/guidelines.xhtml

Benefit-Harm Assessment – Stroke or systemic embolism versus major bleeding

Figure 3 summarises the results for all pair-wise comparisons derived from the Bayesian fixed-effects network meta-analysis (Appendix 9). Results relative to standard adjusted dose VKA have been discussed above. For pair-wise comparisons among newer oral anticoagulants for stroke or SE, dabigatran 150 mg twice daily was associated fewer events versus dabigatran 110 mg twice daily, edoxaban 30mg daily, edoxaban 60 mg daily, and rivaroxaban. Apixaban and rivaroxaban were also associated with fewer events compared to edoxaban 30mg daily. There were no differences among low-dose ASA, medium-dose ASA, and clopidogrel plus low-dose ASA. However, low-dose ASA and the combination of clopidogrel plus low-dose ASA were associated with increases in stroke or systemic embolism compared to all newer oral anticoagulants. For major bleeding, apixaban, dabigatran 110 mg twice daily were associated with fewer events versus dabigatran 150 mg twice daily and rivaroxaban. Edoxaban 30 mg daily was associated with fewer events than other new oral anticoagulants, while edoxaban 60 mg daily was associated with fewer events compared with rivaroxaban and clopidogrel plus low-dose ASA. The risk of major bleeding for apixaban was lower compared to clopidogrel plus low-dose ASA. There were no differences associated with the ASA treatments, both for comparisons among themselves and compared to the anticoagulant treatments.

Figure 4 plots the absolute risk for stroke or SE and major bleeding per 1000 treated for the 10 treatments analyzed. Data in Figure 4 were obtained from the results presented in Appendix 6 and 7. Examination of the data in Figure 4 suggests that the balance of benefits and harms of the new oral anticoagulants for stroke or systemic embolism and major bleeding is positive compared to standard adjusted dose VKA (decrease in stroke or systemic embolism and/or major bleeding) and largely similar among one another. Comparison of all anticoagulant treatment

For peer review only - http://bmjopen.bmj.com/site/about/guidelines.xhtml

(including standard adjusted dose VKA) to ASA, or ASA in combination with clopidogrel indicates that whereas the new oral anticoagulants decrease the risk of both stroke or systemic embolism and major bleeding, ASA has a less favorable balance of benefits and harms, i.e., fails to minimize the risk of stroke or systemic embolism and/or major bleeding.

DISCUSSION

We identified 16 RCTs comparing antithrombotic agents for the prevention of all cause stroke or systemic embolism and major bleeding in patients with AF. To the best of our knowledge, this is the most up-to-date systematic review and network meta-analysis[30–34] to synthesize the available benefits and harms on newer oral anticoagulants, standard adjusted dose VKA, and antiplatelet agents for the prevention of stroke and major bleeding in patients with non-valvular AF (Appendix 13). Harenberg et al[30] recently summarized the published indirect comparisons and network meta-analyses evaluating newer oral anticoagulants for AF and an earlier unpublished version of our analysis[17] was considered among the most comprehensive analyses at that time.[30] This network meta-analysis expands upon that unpublished report.[17] Overall, our findings align with those reported in previously published network meta-analyses;[30,31,33] however, unlike previous analyses,[30] we include recently published edoxaban data,[20] and also include antiplatelet agents. We also present detailed evidence networks[35] to illustrate the body of evidence for each outcome; report analysis using multiple statistical models (Appendix 9); report detailed *a priori* sub-group analyses for each outcome using data derived from FDA Public Summary Documents;[9,18] present findings on both the relative and absolute scale,[36,37] and graphically illustrate results using icon arrays. Our network meta-analysis was registered in PROSPERO, and also adheres to PRISMA reporting standards (Appendix 10). It also differs from a recently

For peer review only - http://bmjopen.bmj.com/site/about/guidelines.xhtml

published traditional meta-analysis by Ruff et al[32] given we report comparison of newer oral anticoagulants versus each other, and also include comparisons with antiplatelet agents.

For stroke or systemic embolism, dabigatran 150 mg and apixaban twice daily were associated with reductions in stroke or systemic embolism relative to standard adjusted dose VKA. By contrast, low-dose ASA and the combination of clopidogrel plus low-dose ASA appeared were associated with increases in stroke or systemic embolism relative to standard adjusted dose VKA. No differences were detected between standard adjusted dose VKA and each of the following interventions: dabigatran 110 mg twice daily, edoxaban 30 and 60 mg daily, rivaroxaban, medium-dose ASA, and no treatment/placebo. The absolute risk reduction of stroke or systemic embolism relative to standard adjusted dose VKA ranged from 6 fewer to 15 more events per 1000 patients treated per year, with dabigatran 150 mg twice daily yielding the largest aboslute risk reduction and clopidogrel plus low-dose ASA the largest absolute risk increase.

For major bleeding, edoxaban 30 mg daily, apixaban, edoxaban 60 mg daily, and dabigatran 110 mg twice daily were associated with a decreased risk of major bleeding relative to standard adjusted dose VKA. No differences for major bleeding were detected between standard adjusted dose VKA and each of the remaining interventions: dabigatran 150 mg twice daily, rivaroxaban, clopidogrel plus low-dose ASA, and all ASA dosages. The absolute risk difference of major bleeding relative to standard adjusted dose VKA ranged from 18 fewer to 24 more events per 1000 patients treated, with edoxaban 30 mg daily yielding the largest absolute risk reduction and medium dose ASA the largest absolute risk increase. Results of network meta-analyses reflect the relative efficacy and safety profile of antiplatelet and anticoagulant drugs as documented by

For peer review only - http://bmjopen.bmj.com/site/about/guidelines.xhtml

For peer review only

large clinical trials. Thus, international guidelines[1,2] for prevention of stroke in patients with AF also recommended oral anticoagulation over antiplatelet therapy as the latter had only modest protective effects. With respect to the new oral anticoagulants none of the guidelines has given a recommendation to prefer one of the new oral anticoagulants over another.[1,2]

Network meta-analysis involves pooling of trials. We observed variability across studies included in the evidence network in terms of $CHADS_2$, age and TTR (Appendix 3 and 4). For example, patients using rivaroxaban had a higher risk of stroke because ROCKET-AF[22] included patients with $CHADS_2 \geq 2$. As such, we conducted sub-group analysis to adjust for differences in patient and study-level characteristics. We derived data for sub-group analyses from FDA Public Summary documents[9,18,19] which enhanced our ability to account for variability across studies (e.g., use intention to treat (ITT) population for rivaroxaban versus per-protocol as-treated population reported in publication[22]). Results from these sub-group analyses differed slightly from those reported in the primary analysis, indicating that there may be differences in the benefits and harms across sub-groups. This becomes more apparent when sub-group analyses are reported on the absolute scale.[3] It is unclear whether the absolute risk reductions associated with these differences translate into clinically meaningful benefits in practice, especially in sub-populations of patients with a lower baseline risk of stroke or systemic embolism. To fully elucidate the comparative benefits and harms of antithrombotic agents across the various sub-populations, rigorously conducted comparative studies,[38,39] or network meta-regression analyses of patient-level data[40] are required.

For peer review only - http://bmjopen.bmj.com/site/about/guidelines.xhtml

There are several limitations with the analysis we employed. First, there is notable heterogeneity in patients and study characteristics (Appendix 3, 4). However, the small number of studies limits the analyses that can be conducted to account for heterogeneity in the absence of patient-level data. Second, there is insufficient sub-group data available for other outcomes reported in the RCTs included. As a consequence, detailed analysis was limited to two outcomes – stroke and SE and major bleeding. Third, we reported the results from the fixed-effects model in the main text. We felt that this was appropriate as the nodes in evidence networks are connected largely by single studies. Effect estimates derived from the fixed-effects model aligned more closely with the direct estimates. Reporting results from the fixed-effects model in the main text likely biases results in favour of the new oral anticoagulants, as treatments that achieved statistically significant results in the primary RCTs retained statistically significant findings when using the fixed-effects model, but not always when using the random-effects model due to the prior[11] on the between study variance. Nevertheless, the results from the random-effects model have also been reported in Appendix 9, along with detailed discussion around the limitations in using the fixed-effects model. Fourth, the absolute risk reduction for the network meta-analysis was calculated using the event rate in the standard adjusted dose VKA arm of the RE-LY study. The RE-LY trial was selected because it contained data for both $CHADS_2 < 2$ and $CHADS_2 \geq 2$ and had detailed data made publicly available following an FDA review of dabigatran. Use of a different study (e.g., ARISTOTLE) for baseline event rate data is unlikely to change the results substantially given that the event rates in standard adjusted dose VKA arms were similar across studies. Fifth, we present findings using graphics that have been largely applied in the network meta-analysis literature,[41] such as a table with odds ratios for all pair-wise comparisons. This method of presenting findings on the relative scale is problematic when you

For peer review only - http://bmjopen.bmj.com/site/about/guidelines.xhtml

are conducting assessments to compare the balance of benefits and harms, which need to be standardized. As such, we also provided results in absolute terms. We report our findings using icon arrays, although it should be noted that these results do not account for the utility values based upon patient preferences for each of the outcomes,[42,43] nor do they reflect uncertainty although we report absolute reductions on the risk benefit plane reflecting uncertainty in Appendix 8. There are other methods available that can incorporate patient preferences for outcomes.[42,43] Further, estimates of benefit and harm with several of the therapies (e.g., rivaroxaban) come from one trial and thus such data are not particularly robust. Further research is needed comparing the balance of benefit and harms using other research methodologies, incorporating other relevant outcomes, patient preferences for each of the outcomes,[42,43] and additional studies when they become available. Finally, haemorrhagic stroke is also considered a major bleed in underlying studies. Accordingly, we may have double counted haemorrhage stroke in this analysis. Unfortunately, we are not able to account for this issue in all included studies. Nonetheless, it is important to note this limitation and highlight that this potentially biases results in favor of newer oral anticoagulants given these agents were associated with reductions in these events.

CONCLUSIONS

Compared with standard adjusted dose VKA, dabigatran 150 mg and apixaban twice daily were associated with reductions in the incidence of stroke or systemic embolism in patients with AF, whereas, low-dose ASA and the combination of clopidogrel plus low-dose ASA increased the risk relative to standard adjusted dose VKA at preventing stroke or systemic embolism. Edoxaban 30 mg daily, apixaban, edoxaban 60 mg daily, and dabigatran 110 mg twice daily and

For peer review only - http://bmjopen.bmj.com/site/about/guidelines.xhtml

For peer review only

was associated with a reduced risk in major bleeding compared with standard adjusted dose VKA. Although the results of the current review revealed that there were differences in stroke or systemic embolism and major bleeding among antithrombotic agents in the management of patients with AF in relative terms, it is unclear whether the absolute risk reductions associated with these differences translate into clinically meaningful benefits in practice, especially in sub-populations of patients with a lower baseline risk of stroke or systemic embolism. Rigorously conducted comparative studies or network meta-regression analyses of patient-level data are required to fully elucidate the comparative benefits and harms of antithrombotic agents across the various sub-populations.

For peer review only

TABLES

Table 1: Sub-group and sensitivity analyses for stroke or systemic embolism and major bleeding relative to standard adjusted dose VKA, Odds ratios ± 95% Credible Intervals versus standard adjusted dose VKA

Treatment	Reference Case – Fixed Effect NMA	Subgroup analysis by CHADS$_2$ Score		Subgroup analysis by Age		Subgroup analysis by TTR	
		CHADS$_2$ < 2	CHADS$_2$ ≥ 2	Age < 75 years	Age ≥ 75 years	TTR < 66%	TTR ≥ 66%
**Stroke or Systemic Embolism**							
**Dabigatran 150 mg twice daily**	**0.66 (0.53,0.82)**	0.61 (0.37,1)	**0.66 (0.52,0.85)**	0.63 (0.46,0.87)	**0.66 (0.48,0.9)**	0.54 (0.39,0.73)	0.81 (0.58,1.11)
**Apixaban 5 mg twice daily**	**0.78 (0.65,0.94)**	0.86 (0.57,1.28)	**0.78 (0.63,0.95)**	0.85 (0.67,1.07)	**0.71 (0.53,0.95)**	0.79 (0.62,1.003)	0.79 (0.60,1.05)
**Edoxaban 60 mg daily**	0.87 (0.74,1.02)	NA	0.87 (0.74,1.01)	0.91 (0.73,1.14)	0.83 (0.66,1.04)	0.83 (0.65,1.07)¶	0.91 (0.73,1.13)¶
**Rivaroxaban 20 mg daily**	0.88 (0.74,1.04)	NA	0.88 (0.74,1.04)	0.95 (0.75,1.2)	0.8 (0.63,1.03)	0.81 (0.65,1.01)	0.72 (0.47,1.1)
**Dabigatran 110 mg twice daily**	0.91 (0.74,1.12)	1 (0.64,1.55)	0.89 (0.71,1.12)	0.93 (0.7,1.24)	0.88 (0.66,1.19)	0.91 (0.69,1.19)	0.9 (0.66,1.24)
**Edoxaban 30 mg daily**	1.14 (0.98,1.32)	NA	1.14 (0.98,1.32)	1.16 (0.94,1.44)	1.13 (0.91,1.4)	1.01 (0.80,1.28)¶	1.24 (1.01,1.24)¶
**Medium Dose ASA (> 100 mg and ≤ 300 mg daily)**	1.35 (0.74,2.47)	1.97 (0.63,6.73) **	NA	NA	NA	NA	NA
**Low Dose ASA (≤ 100 mg daily)**	**1.87 (1.26,2.8)**	2.2 (1.18,4.25)	2.1 (0.91,5.15) *	NA	2.04 (1.33,3.20)	NA	NA
**Clopidogrel 75 mg daily & Low dose Aspirin (≤ 100 mg daily)**	**1.93 (1.42,2.64)**	3.16 (1.39,8.22)	1.15 (0.84,1.57)	NA	NA	1.52 (0.96,2.45)	**2.4 (1.59,3.71)**
**Major Bleeding**							
**Edoxaban 30 mg daily**	**0.46 (0.4,0.54)**	NA	**0.46 (0.4,0.54)**	0.50 (0.41,0.61) ‡	0.51 (0.41-0.62) ‡	0.45 (0.35,0.58) ‡ ¶	0.51 (0.43,0.61) ‡¶
**Apixaban 5 mg twice daily**	**0.69 (0.6,0.8)**	**0.59 (0.44,0.78)**	**0.73 (0.62,0.87)**	**0.73 (0.6,0.89)**	**0.65 (0.52,0.80)**	**0.57 (0.45,0.71)**	**0.81 (0.67,0.98)**
**Edoxaban 60 mg daily**	**0.79 (0.69,0.9)**	NA	**0.79 (0.69,0.9)**	0.74 (0.62-0.90) ‡	0.82 (0.68-0.98) ‡	0.69 (0.55,0.87) ‡¶	0.83 (0.70,0.97) ‡¶
**Dabigatran 110 mg twice daily**	**0.8 (0.69,0.93)**	**0.65 (0.47,0.89)**	0.86 (0.73,1.02)	**0.62 (0.49,0.77)**	1.02 (0.84,1.25)	0.74 (0.61,0.91)	0.86 (0.69,1.06)

For peer review only - http://bmjopen.bmj.com/site/about/guidelines.xhtml

For peer review only

Treatment	Reference Case – Fixed Effect NMA	Subgroup analysis by $CHADS_2$ Score		Subgroup analysis by Age		Subgroup analysis by TTR	
		$CHADS_2 < 2$	$CHADS_2 \geq 2$	Age < 75 years	Age ≥ 75 years	TTR < 66%	TTR ≥ 66%
**Dabigatran 150 mg twice daily**	0.93 (0.81,1.08)	0.77 (0.57,1.04)	1.01 (0.86,1.19)	**0.7 (0.56,0.86)**	1.19 (0.98,1.45)	0.76 (0.62,0.94)	1.15 (0.94,1.4)
**Rivaroxaban 20 mg daily**	1.03 (0.89,1.19)	NA	1.03 (0.89,1.19)	0.93 (0.76,1.13)	1.15 (0.93,1.41)	0.92 (0.77,1.1)	**1.3 (1.01,1.69)**
**Low Dose ASA (≤ 100 mg daily)**	1.05 (0.6,1.87)	0.83 (0.42,1.64)	1.6 (0.55,5.02) *	NA	1.01 (0.57,1.78)	NA	NA
**Clopidogrel 75 mg daily & Low dose Aspirin (≤ 100 mg daily)**	1.1 (0.83,1.47)	1.51 (0.89,2.61)	0.97 (0.69,1.36) †	NA	NA	0.66 (0.42,1.02)	1.66 (1.12,2.47)
**Medium Dose ASA (> 100 mg and ≤ 300 mg daily)**	1.79 (0.63,5.67)	1.48 (0.11,19.07)**	NA	NA	NA	NA	NA

ASA= acetylsalicylic acid; mg= milligram; NA=not available; NMA= network meta-analysis; OR=odds ratio; TTR= time in therapeutic range; VKA=vitamin K antagonist
* Results may be slightly biased against LDASA. Derived from BAFTA study where $CHADS_2$ subgroup data stratified by $CHADS_2$ 1-2 (vs. 0-1) and $CHADS_2$ 3-6 (vs. 2-6).
** Results may be slightly biased against medium-dose ASA. Based on study level results from CAFA and JAST studies. Although these studies consisted primarily of low-risk populations, some patients may have $CHADS_2$ scores greater than 2.
† Estimated from subgroup study by Healey et al 2008
¶ Data provided from ENGAGE AF-TIMI 48[20] uses TTR greater than 60%
‡ Based on overall time period where overall time period was defined as first dose to the last dose plus 3 days.

FIGURES

Figure 1: Evidence network for all cause stroke or systemic embolism.* The width of the lines is proportional to the number of randomized controlled trials comparing each pair of treatments, and the size of each treatment node is proportional to the number of randomised participants (sample size). A dotted line indicates a three-arm randomized controlled trial and a black node indicates a node included in the analysis but not reported in the main text.

ASA= acetylsalicylic acid; mg= milligram; RCT= RCT = randomized controlled trial; VKA= vitamin K antagonist
*Four RCTs (PETRO,[24] WASPO[25], Chung et al,[26] and Yamashita et al[27]) were not included in the analysis because they did not report data for this outcome or had zeroes in both arm

Figure 2: Odds ratio for all cause stroke or systemic embolism (A) and major bleeding (B) in Bayesian network meta-analysis versus standard adjusted dose VKA.

CrI= credible interval; mg= milligram; VKA= vitamin K antagonist

Figure 3: Odds Ratios (OR) from network meta-analyses for stroke or systemic embolism and major bleeding for all pair-wise comparisons.* ORs for recurrence of stroke or systemic embolism are below the diagonal (row-defining treatment versus column-defining treatment) and those for major bleeding are above the diagonal and in blue (column-defining treatment versus row-defining treatment). To obtain ORs for comparisons in the opposite direction, reciprocals should be taken (e.g., the OR for standard dose warfarin compared with apixaban 5 mg twice daily for stroke or systemic embolism is 1/0.78=1.28). Significant results are in bold and underscored (and in larger font).

mg= milligram; VKA= vitamin K antagonist
* Results between individual treatments, especially newer oral anticoagulants, should be interpreted with caution given the limitations associated with using a fixed-effects model. See Appendix 9 for additional details.

Figure 4: Icon array illustrating the absolute risks of Stroke or systemic embolism (blue) and major bleeding episodes (red) per 1000 patients treated*

* Figures do not reflect uncertainty around effect estimates and there is more uncertainty (i.e., wider credible intervals) for low dose ASA, medium dose ASA, and clopidogrel plus low dose ASA (See Appendix 8). Underlying studies may also double count haemorrhage stroke.

For peer review only - http://bmjopen.bmj.com/site/about/guidelines.xhtml

For peer review only

For peer review only - http://bmjopen.bmj.com/site/about/guidelines.xhtml

Acknowledgments: The authors would like to acknowledge Carolyn Spry. She was the Information Specialist from CADTH on the review who conducted the search strategy. We would also like to thank Dr. Robert Giugliano for providing the sub-group data for edoxaban and his thoughtful comments on earlier drafts of the manuscript during the peer review process.

Contributors: CC conceived the study design, performed the data extraction, analysis, and drafted the manuscript. DC and GW conceived the study design, helped with the data analysis, and reviewed the manuscript for important intellectual content. TR conceived the study design, performed the systematic review, data extraction, and reviewed the manuscript for important intellectual content. SK, SS, KG, AB, KM, KC performed the systematic review, data extraction, and reviewed the manuscript for important intellectual content. TC, MC, and KC conceived the study and reviewed the manuscript for important intellectual content. All authors have read and approved the final version of the manuscript. All authors agree to be accountable for all aspects of the work in ensuring that questions related to the accuracy or integrity of any part of the work are appropriately investigated and resolved.

Funding information: This research was supported through a grant by the Canadian Institute of Health Research (CIHR) Drug Safety and Effectiveness Network and financial support from the Canadian Agency for Drugs and Technologies in Health (CADTH). The CIHR Drug Safety and Effectiveness Network played no role in study design, collection, analysis, interpretation of data, writing of the manuscript, or in the decision to submit the paper for publication. They accept no responsibility for the contents. Employees from CADTH were involved in the study design, collection, analysis, interpretation of data, and writing of the manuscript. CADTH, funded by Canadian federal, provincial and territorial governments, is an independent, not-for-profit producer and broker of health technology assessments. Federal, provincial, and territorial health care decision-makers rely on our evidence-based information to make informed decisions about the effectiveness and efficiency of drugs and other health technologies.

Competing interests: All authors have completed the ICMJE uniform disclosure form at www.icmje.org/coi_disclosure.pdf (available on request from the corresponding author) and declare: no support from any organisation for the submitted work; CC is a recipient of a Vanier Canada Graduate Scholarship from the Canadian Institutes of Health Research and has received funding from Canadian Network and Centre for Trials Internationally (CANNeCTIN) and is a trainee on the CIHR Drug Safety and Effectiveness Network team grant. MC is a recipient of a New Investigator Award from the Heart and Stroke Foundation of Canada and holds a T2 research chair in cancer and thrombosis from the University of Ottawa.

Ethical approval: Not required.

Data sharing: The statistical code and dataset used for analyses are reported online: http://www.cadth.ca/media/pdf/TR0003_AntithromboticAgents-AF_ScienceReport-e.pdf. Additional data can be requested by emailing George Wells: gawells@ottawaheart.ca .

The Corresponding Author has the right to grant on behalf of all authors and does grant on behalf of all authors, an exclusive licence (or non exclusive for government employees) on a worldwide basis to the BMJ Publishing Group Ltd to permit this article (if accepted) to be published in BMJ editions and any other BMJPGL products and sublicences such use and exploit all subsidiary rights, as set out in our licence

For peer review only - http://bmjopen.bmj.com/site/about/guidelines.xhtml

REFERENCES

1. Kirchhof, P. *et al.* Atrial fibrillation guidelines across the Atlantic: a comparison of the current recommendations of the European Society of Cardiology/European Heart Rhythm Association/European Association of Cardiothoracic Surgeons, the American College of Cardiology Fou. *Eur. Heart J.* **34,** 1471–1474 (2013).

2. Skanes, A. C. *et al.* Focused 2012 update of the Canadian Cardiovascular Society atrial fibrillation guidelines: recommendations for stroke prevention and rate/rhythm control. *Can. J. Cardiol.* **28,** 125–36 (2012).

3. *Antithrombotic agents for the prevention of stroke and systemic embolism in patients with atrial fibrillation*. (2013). at <http://www.cadth.ca/media/pdf/TR0003_AntithromboticAgents-AF_ScienceReport-e.pdf>

4. Schulman, S. & Kearon, C. Definition of major bleeding in clinical investigations of antihemostatic medicinal products in non-surgical patients. *J. Thromb. Haemost.* **3,** 692–4 (2005).

5. NHS Quality Improvement Scotland (NHS QIS). *SIGN 50. A guideline developer's handbook*. (2008). at <http://www.sign.ac.uk/pdf/sign50.pdf>

6. Moher, D., Liberati, A., Tetzlaff, J. et al. Preferred reporting items for systematic reviews and meta-analyses: the PRISMA statement. *PLoS Med.* **6,** e1000097 (2009).

7. Dias, S., Welton, N. J., Sutton, A. J. et al. *NICE DSU Technical Support Document 2: A Generalised Linear Modelling Framework for Pairwise and Network Meta-Analysis of Randomised Controlled Trials*. *Trop. Med.* 1–96 (2011). at <www.nicedsu.org.uk>

8. Connolly, S. & Ezekowitz, M. Dabigatran versus warfarin in patients with atrial fibrillation. *N. Engl. J. Med.* **361,** 1139–1151 (2009).

9. Ingelheim, B. *Advisory committee briefing document: dabigatran etexilate [Internet]*. 1–168 (2010). at <http://www.fda.gov/downloads/advisorycommittees/committeesmeetingmaterials/drugs/cardiovas>

10. Cipriani, A. *et al.* Comparative efficacy and acceptability of 12 new-generation antidepressants: a multiple-treatments meta-analysis. *Lancet* **373,** 746–58 (2009).

11. Turner, R. M., Davey, J., Clarke, M. J., et al. Predicting the extent of heterogeneity in meta-analysis, using empirical data from the Cochrane Database of Systematic Reviews. *Int. J. Epidemiol.* **41,** 818–27 (2012).

For peer review only

12. Spiegelhalter, D. J., Best, N. G., Carlin, B. P. et al. Bayesian measures of model complexity and fit. *J. R. Stat. Soc. Ser. B (Statistical Methodol.* **64,** 583–639 (2002).

13. Spiegelhalter, D., Thomas, A. & Best, N. WinBUGS user manual. *MRC Biostat. Unit* **2,** (2004).

14. meta: Meta-Analysis with R. at <http://cran.r-project.org/web/packages/meta/index.html>

15. Dias, S., Welton, N.J., Sutton, A.J., et al. *NICE Technical Support Document 4: Inconsistency in Networks of Evidence Based on Randomised Controlled Trials*. 1–39 (2011). at <www.nicedsu.org.uk>

16. Salanti, G., Marinho, V. & Higgins, J. P. T. A case study of multiple-treatments meta-analysis demonstrates that covariates should be considered. *J. Clin. Epidemiol.* **62,** 857–64 (2009).

17. Wells, G., Coyle, D., Cameron, C., et al. *Safety, effectiveness, and costeffectiveness of new oral anticoagulants compared with warfarin in preventing stroke and other cardiovascular events in patients with atrial fibrillation*. (2012). at <http://www.cadth.ca/media/pdf/NOAC_Therapeutic_Review_final_report.pdf>

18. Johnson & Johnson Pharmaceutical Research & Development. *Rivaroxaban for the Prevention of Stroke and Non-Central Nervous System (CNS) Systemic Embolism in Patients With Atrial Fibrillation*. **7939,** 222 (2011).

19. McDonagh, M. S., Peterson, K., Balshem, H. et al. US Food and Drug Administration documents can provide unpublished evidence relevant to systematic reviews. *J. Clin. Epidemiol.* (2013). doi:10.1016/j.jclinepi.2013.05.006

20. Giugliano, R. P. *et al.* Edoxaban versus warfarin in patients with atrial fibrillation. *N. Engl. J. Med.* **369,** 2093–104 (2013).

21. Granger, C. & Alexander, J. Apixaban versus warfarin in patients with atrial fibrillation. *N. Engl. J. Med.* 981–992 (2011).

22. Patel, M., Mahaffey, K. & Garg, J. Rivaroxaban versus warfarin in nonvalvular atrial fibrillation. *N. Engl. J. Med.* 883–891 (2011).

23. Connolly, S. *et al.* Clopidogrel plus aspirin versus oral anticoagulation for atrial fibrillation in the Atrial fibrillation Clopidogrel Trial with Irbesartan for prevention of Vascular Events (ACTIVE W): a randomised controlled trial. *Lancet* **367,** 1903–12 (2006).

24. Ezekowitz, M. D. *et al.* Dabigatran with or without concomitant aspirin compared with warfarin alone in patients with nonvalvular atrial fibrillation (PETRO Study). *Am. J. Cardiol.* **100,** 1419–26 (2007).

For peer review only

For peer review only - http://bmjopen.bmj.com/site/about/guidelines.xhtml

25. Rash, A. *et al.* A randomised controlled trial of warfarin versus aspirin for stroke prevention in octogenarians with atrial fibrillation (WASPO). *Age Ageing* **36,** 151–6 (2007).

26. Chung, N. *et al.* Safety of edoxaban, an oral factor Xa inhibitor, in Asian patients with non-valvular atrial fibrillation. *Thromb. Haemost.* **105,** 535–44 (2011).

27. Yamashita, T. *et al.* Randomized, Multicenter, Warfarin-Controlled Phase II Study of Edoxaban in Japanese Patients With Non-Valvular Atrial Fibrillation. *Circ. J.* **76,** 1840–1847 (2012).

28. Connolly, S. & Eikelboom, J. Apixaban in patients with atrial fibrillation. *N. Engl. J. Med.* **364,** 806–817 (2011).

29. Connolly, S. J. *et al.* Effect of clopidogrel added to aspirin in patients with atrial fibrillation. *N. Engl. J. Med.* **360,** 2066–78 (2009).

30. Harenberg, J. & Weiss, C. Clinical trials with new oral anticoagulants. Additive value of indirect comparisons also named network meta-analyses. *Hamostaseologie* **33,** 62–70 (2013).

31. Dogliotti, A., Paolasso, E. & Giugliano, R. P. Current and new oral antithrombotics in non-valvular atrial fibrillation: a network meta-analysis of 79 808 patients. *Heart* (2013). doi:10.1136/heartjnl-2013-304347

32. Ruff, C. T. *et al.* Comparison of the efficacy and safety of new oral anticoagulants with warfarin in patients with atrial fi brillation□: a meta-analysis of randomised trials. *Lancet* **6736,** 1–8 (2013).

33. Assiri, A., Al-Majzoub, O., Kanaan, A. O., et al. Mixed treatment comparison meta-analysis of aspirin, warfarin, and new anticoagulants for stroke prevention in patients with nonvalvular atrial fibrillation. *Clin. Ther.* **35,** 967–984.e2 (2013).

34. Slot, K. & Berge, E. Factor Xa inhibitors versus vitamin K antagonists for preventing cerebral or systemic embolism in patients with atrial fibrillation. *Stroke* (2013). at <http://stroke.ahajournals.org/content/44/12/e165.short>

35. Salanti, Georgia, Kavvoura, F, et al. Exploring the geometry of treatment networks. *Ann. Intern. Med.* **148,** 544–553 (2008).

36. Barratt, A. *et al.* Tips for learners of evidence-based medicine: 1. Relative risk reduction, absolute risk reduction and number needed to treat. *Can. Med. Assoc. J.* **171,** 353–8 (2004).

37. Schechtman, E. Odds ratio, relative risk, absolute risk reduction, and the number needed to treat--which of these should we use? *Value Health* **5,** 431–6 (2002).

For peer review only - http://bmjopen.bmj.com/site/about/guidelines.xhtml

38. Berger, M. L., Mamdani, M., Atkins, D. et al. Good research practices for comparative effectiveness research: defining, reporting and interpreting nonrandomized studies of treatment effects using secondary data sources: the ISPOR Good Research Practices for Retrospective Database Analysis Task Force . *Value Health* **12,** 1044–52 (2009).

39. Berger, M. L. *et al.* Prospective observational studies to assess comparative effectiveness: the ISPOR good research practices task force report. *Value Health* **15,** 217–30 (2012).

40. Jansen, J. P. Network meta-analysis of individual and aggregate level data. *Res. Synth. Methods* **3,** 177–190 (2012).

41. Salanti, G., Ades, A. & Ioannidis, J. Graphical methods and numerical summaries for presenting results from multiple-treatment meta-analysis: an overview and tutorial. *J. Clin. Epidemiol.* **64,** 163–71 (2011).

42. Guo, J. J. *et al.* A review of quantitative risk-benefit methodologies for assessing drug safety and efficacy-report of the ISPOR risk-benefit management working group. *Value Health* **13,** 657–66 (2010).

43. Boyd, C. *et al. Methods Research Report Methods for Benefit and Harm Assessment in Systematic Reviews*. 52 (2012). at <www.ncbi.nlm.nih.gov/books/NBK115750/>

44. Thorlund, K., Thabane, L. & Mills, E. J. Modelling heterogeneity variances in multiple treatment comparison meta-analysis – Are informative priors the better solution□? *BMC Med. Res. Methodol.* **13,** 1 (2013).

For peer review only

For peer review only - http://bmjopen.bmj.com/site/about/guidelines.xhtml

Systematic Review and Network Meta-analysis comparing Antithrombotic Agents for the Prevention of Stroke and Major Bleeding in Patients with Atrial Fibrillation

Chris Cameron, PhD Student[1,2], Doug Coyle, Professor[1], Trevor Richter, Manager of Clinical Research [3], Shannon Kelly, Research coordinator[2] , Kasandra Gauthier, Clinical Research Officer[3], Sabine Steiner, Associate Professor[4], Marc Carrier, Associate Professor[6], Kathryn Coyle, Health Economist[5], Kristen Moulton, Clinical Research Officer[3], Annie Bai , Clinical Research Officer[3], Tammy Clifford, Adjunct Professor[1,7], George Wells, Professor[1,2]

[1] Department of Epidemiology and Community Medicine, University of Ottawa, Ottawa, Canada
[2] University of Ottawa Heart Institute, Ottawa, Canada
[3] Canadian Agency for Drugs and Technologies in Health, Ottawa, Canada
[4] Medical University of Vienna, Vienna, Austria
[5] Applied Health Economics Research Unit, Ottawa, Canada
[6] Thrombosis Program, Division of Hematology, Department of Medicine, University of Ottawa, Ottawa, Canada
[7] Department of Pediatrics, University of Ottawa, Ottawa, Canada

Word count, abstract: 322
Word count, body of essay: 3,987
Number of figures: 4
Number of tables: 1
Number of References: 44
Supplementary Material: 14

Corresponding author:
Dr. George Wells
Professor and Director, Cardiovascular Research Methods Centre
Department of Epidemiology and Community Medicine, University of Ottawa
Ottawa Heart Institute
40 Ruskin Street, Ottawa, Ontario, Canada K1Y 4W7
Phone: (613) 798-5555, ext. 18640
Fax: (613) 761-4412
Email: gawells@ottawaheart.ca

Keywords: atrial fibrillation, antithrombotic, anticoagulants, stroke, bleeding

For peer review only

Abstract

Objective: To examine the comparative efficacy and safety of antithrombotic treatments (apixaban, dabigatran, edoxaban, rivaroxaban, ~~apixaban, edoxaban,~~ and vitamin K antagonists (VKA) at a standard adjusted dose (target international normalised ratio 2.0-3.0), acetylsalicylic acid, acetylsalicylic acid and clopidogrel) for non-valvular atrial fibrillation and among sub-populations.

Design: Systematic review and network meta-analysis.

Data sources: A systematic literature search strategy was designed and carried out using MEDLINE, EMBASE, Cochrane Register of Controlled Trials, and grey literature including the websites of regulatory agencies and health technology assessment organizations for trials published in English from 1988 to January 2014.

Eligibility criteria for selecting studies: Randomized controlled trials were selected for inclusion if they were published in English, included at least one antithrombotic treatment and involved patients with non-valvular atrial fibrillation eligible to receive anticoagulant therapy.

Results: For stroke or systemic embolism, ~~apixaban and~~ dabigatran 150 mg and apixaban twice daily were associated with reductions relative to standard adjusted dose VKA whereas low-dose ASA and the combination of clopidogrel plus low-dose ASA were associated with increases. Absolute risk reductions ranged from 6 fewer events per 1,000 patients treated for dabigatran 150 mg twice daily to 15 more events for clopidogrel plus ASA. For major bleeding, edoxaban 30 mg daily, apixaban, edoxaban 60 mg daily, and dabigatran 110 mg twice daily~~, edoxaban 30 mg daily, and edoxaban 60 mg daily~~ were associated with reductions compared to standard adjusted dose VKA. Absolute risk reductions with these agents ranged from 18 fewer per 1,000 patients treated each year for edoxaban 30 mg daily to 24 more for medium dose ASA.

Conclusions: Compared with standard adjusted dose VKA, new oral anticoagulants were associated with modest reductions in the absolute risk of stroke and major bleeding. People on antiplatelet drugs experienced more strokes compared with anticoagulant drugs without any reduction in bleeding risk. To fully elucidate the comparative benefits and harms of antithrombotic agents across the various sub-populations, rigorously conducted comparative studies or network meta-regression analyses of patient-level data are required.

(PROSPERO registry - CRD42012002721).

For peer review only

For peer review only - http://bmjopen.bmj.com/site/about/guidelines.xhtml

Article Summary

Strengths and limitations of this study

- This network meta-analysis includes data on edoxaban from the recently published ENGAGE AF-TIMI 48 trial
- The studies pooled in this meta-analysis include antiplatelet agents in addition to newer oral anticoagulants and standard adjusted dose vitamin K antagonists
- We present findings on both the relative and absolute scale, and graphically illustrate absolute risks using icon arrays
- We report detailed *a priori* sub-group analyses for stroke or systemic embolism and major bleeding using data derived from FDA Public Summary Documents
- Comparisons between antithrombotic treatments were largely comprised of single studies, albeit large studies
- There is notable heterogeneity and the small number of studies limits the analyses that can be conducted to account for heterogeneity in the absence of patient-level data
- Icon arrays only consider the primary efficacy and safety endpoints of underlying studies and do not account for different clinical consequences associated with each of the outcomes

For peer review only - http://bmjopen.bmj.com/site/about/guidelines.xhtml

INTRODUCTION

Atrial fibrillation (AF) is a common cardiac arrhythmia associated with increased morbidity and mortality.[1,2] Patients with AF sustain an increased risk of arterial thromboembolism and stroke, associated with high recurrence and substantial debilitating impact. Therefore, antithrombotic strategies using anticoagulant drugs and antiplatelet agents are recommended for AF patients presenting with risk factors for stroke. Antithrombotic therapy is also associated with a risk of bleeding; therefore, the beneficial effects on stroke prevention should always be compared against a patient's risk of major bleeding.[1,2]

Existing guidelines recommend anticoagulant therapy for patients at intermediate or high risk of stroke.[1,2] Although standard adjusted dose vitamin K antagonists (VKA) (e.g., warfarin) has been the cornerstone treatment for reducing the risk of stroke or systemic embolism (SE) in this population, it is associated with several drawbacks.[2] which have prompted the development of newer oral anticoagulants such as direct thrombin (dabigatran) and Xa (e.g. apixaban, edoxaban rivaroxaban, edoxaban) inhibitors. [1,2] While some of these newer treatment options have been demonstrated to be efficacious in preventing stroke or SE compared to standard adjusted dose VKA, the relative efficacy and risk of bleeding of newer oral anticoagulants, both among themselves and in comparison to antiplatelet agents, is not clear, especially in certain sub-populations of patients, e.g., $CHADS_2$ score <2 or ≥2; time in therapeutic range (TTR) < 66% or ≥ 66%; Age <75 or ≥ 75 years. Therefore, the objective of this paper was to compare antithrombotic agents for the prevention of stroke and major bleeding in patients with non-valvular AF and among sub-populations.

For peer review only - http://bmjopen.bmj.com/site/about/guidelines.xhtml

For peer review only

METHODS

Published literature was identified by searching MEDLINE, MEDLINE In-Process & Other Non-Indexed Citations, EMBASE, BIOSIS Previews, PubMed and the Cochrane Central Register of Controlled Trials were searched through the Ovid interface to identify English-language clinical articles published from 1988 to January 23, 2014. Regular alerts were also established. Websites of regulatory agencies, Canadian and major international health technology assessment agencies, clinical practice guidelines as well as The Cochrane Library (2012, Issue 6) and University of York Centre for Reviews and Dissemination databases were searched. Complete details of the electronic search strategy, including any limits used, are reported in Appendix 1. The protocol was published online and was registered in the PROSPERO International Prospective Register of Systematic Reviews (PROSPERO registry - CRD42012002721).

The population of interest was individuals with non-valvular AF requiring anticoagulation (including all risk levels and regardless of any comorbidities). The following treatments were included in the review: apixaban, dabigatran, edoxaban, rivaroxaban, standard adjusted dose VKA, acetylsalicylic acid (ASA), ASA and clopidogrel. Numerous outcomes were considered and results are reported elsewhere;[3] however, the focus of this publication is the outcomes of all-cause stroke or SE and major bleeding as defined by the International Society on Thrombosis and Haemostasis [ISTH] definition),[4] which were the primary efficacy and safety outcomes used in the newer oral anticoagulant studies.

For peer review only - http://bmjopen.bmj.com/site/about/guidelines.xhtml

Active and placebo-controlled randomized controlled trials (RCTs) were selected for inclusion if they were published in English, included at least one antithrombotic treatment under review (using pre-specified doses), reported data for stroke/SE or major bleeding, and involved patients with non-valvular AF eligible to receive anticoagulant therapy, regardless the level of stroke risk. Trials that included patients with contraindication to anticoagulant treatment were excluded. VKA trials were included if the dose was adjusted to a target international normalised ratio (INR) 2.0-3.0. Any dose of ASA was considered for inclusion, but ASA dose was stratified in the analysis as low (≤ 100 mg daily), medium (>100 mg to ≤ 300 mg daily), or high (>300 mg daily). We only included new oral anticoagulants which had at least one large phase III study. Three reviewers independently extracted the data on baseline characteristics, intervention(s) evaluated, including dose, duration, and relevant co-medication and results for each included article, using a standardized template. All extracted data was checked for accuracy by three independent reviewers. Any disagreements in the assessment of these data were resolved through discussion until consensus was reached. Quality assessment of RCTs was also performed independently by two reviewers using a standardized table based on major items from the SIGN 50 instrument for internal validity.[5] The trial selection process is presented in a flowchart based on the Preferred Reporting Items for Systematic Reviews and Meta-Analyses (PRISMA)[6] statement (Appendix 2).

Bayesian network meta-analyses were conducted using WinBUGS software (MRC Biostatistics Unit, Cambridge, UK). A binomial likelihood model[7] which accounts for the use of multi-arm trials was used for the analyses given the datasets provided were dichotomous outcomes and included multi-arm trials. Trials with zero cells in both arms or nodes where there were no events

For peer review only - http://bmjopen.bmj.com/site/about/guidelines.xhtml

were excluded from the evidence networks because they do not contribute information or allow interpretable information.[7] Both fixed and random-effects network meta-analyses were conducted, although the fixed-effects model was chosen for the reference case analysis, as the evidence network was largely comprised of single study connections. We modeled point estimates and 95% credible intervals for odds ratios (OR) using Markov Chain Monte Carlo methods. The absolute risk difference per 1000 patients treated each year for each outcome was also calculated, based on the standard adjusted dose VKA arm of the Randomized Evaluation of Long-Term Anti-coagulation Therapy (RE-LY) trial.[8] The RE-LY trial[8] was selected because it contained data for both $CHADS_2<2$ and $CHADS_2\geq2$ and had detailed data available from the Food and Drug Administration (FDA) Public Summary Report.[9] We also assessed the probability that each treatment was the most efficacious regimen, the second best, the third best, and so on.[10] Vague or flat priors, such as $N(0, 100^2)$ were assigned for basic parameters throughout, although informative priors were considered.[11] Assessment of model fit was based on deviance information criterion (DIC) and comparison of residual deviance to number of unconstrained data points.[7,12] To ensure convergence was reached, trace plots and the Brooks-Gelman-Rubin statistic were assessed.[13] Three chains were fit in WinBUGS for each analysis, with at least 40,000 iterations, and a burn-in of at least 40,000 iterations. We also qualitatively compared the results from our network meta-analysis with direct pair-wise estimates. Frequentist pair-wise meta-analyses were conducted using R (R Language and Environment for Statistical Computing, Vienna, Austria) and package meta.[14]

A key assumption behind network meta-analysis is that the analysed network is consistent, i.e., there is no conflict between direct and indirect evidence.[15] To assess inconsistency, we compared

For peer review only

For peer review only - http://bmjopen.bmj.com/site/about/guidelines.xhtml

deviance and DIC statistics in fitted consistency and inconsistency models.[15] We also plotted the posterior mean deviance of the individual data points in the inconsistency model against their posterior mean deviance in the consistency model to identify any loops in where inconsistency is present (Appendix 11).[15] Additionally, the results from our network meta-analysis were qualitatively compared with direct frequentist pair-wise estimates.

Network meta-analysis also requires that studies are sufficiently similar in order to pool their results.[16] Available study and patient characteristics were assessed to ensure similarity and to investigate the potential impact of heterogeneity on effect estimates. Clinical and methodological heterogeneity was identified in a number of areas. The issues identified were similar to those reported in previous systematic reviews of anticoagulant drugs (e.g., differences in TTR, $CHADS_2$ score).[17] Heterogeneity was assessed by conducting network meta-analysis with the following pre-specified subgroup data reported in the individual RCTs: TTR < 66% or ≥ 66%; $CHADS_2$ score <2 or ≥2; Age <75 or ≥ 75 years.[17] The data to conduct these sub-group analyses were derived from FDA Public Summary Documents when not available in the publications.[9,18,19] Secondary evidence networks were constructed for each of the sub-group analyses. The WinBUGS code[7] and data needed to replicate all analyses are available online.

This research was supported through a grant by the Canadian Institute of Health Research (CIHR) Drug Safety and Effectiveness Network and financial support from the Canadian Agency for Drugs and Technologies in Health (CADTH).

For peer review only - http://bmjopen.bmj.com/site/about/guidelines.xhtml

RESULTS

Study Characteristics of included studies

The systematic review included 16 individual RCTs (reported in 32 publications and FDA reports); all evaluated the efficacy and safety of antithrombotic agents: apixaban (5 mg twice daily), dabigatran (150 or 110 mg twice daily), edoxaban (30 or 60 mg daily), rivaroxaban (20 mg daily), ~~apixaban (5 mg twice daily), edoxaban (30 or 60 mg daily),~~ standard adjusted dose VKA, ASA (low dose (< 100 mg daily); medium dose (100-300 mg daily), or low dose-ASA plus clopidogrel (75 mg daily) in patients with non-valvular AF. None of the included studies directly compared one new oral anticoagulant drug with another (Appendix 4). Of the 82,396 randomized patients included in the primary analysis, five large multicentre trials account for 78,296 patients (96%): ENGAGE AF-TIMI 48 (n=21,105),[20] ARISTOTLE (n=18,201),[21] RE-LY (n=18,113),[8] ROCKET-AF (n=14,264)[22] and ACTIVE-W (n=6706).[23] Most trials included stroke and SE as a primary efficacy outcome, while bleeding events were a frequent safety outcome. Follow-up ranged from 12 weeks to 3.5 years. All trials published results between 1989 and 2013. Mean $CHADS_2$ score was reported in eight trials, encompassing the vast majority of patients included in the systematic review. Reported $CHADS_2$ scores were consistent with a high-risk population ($CHADS_2 \geq 2$), with patients from ROCKET-AF[22] showing the highest risk for stroke (mean $CHADS_2 = 3.5$).

Mean age across the included trials ranged from 62 years to 83 years (Appendix 3). All trials included patients of both gender and had a higher proportion of male participants in most studies. However, the proportions of patients with a prior stroke or TIA varied substantially across the included trials, ranging from 3% in JAST to 55% in ROCKET-AF.[22] The same observation can

For peer review only - http://bmjopen.bmj.com/site/about/guidelines.xhtml

be made regarding the proportions of patients with other concomitant conditions, i.e. congestive heart failure, hypertension, diabetes, myocardial infarction, and prior vitamin K antagonist experience. TTR was reported in all but one trial that included a standard adjusted dose VKA treatment arm. Patient baseline characteristics are summarized in Appendix 3.

The studies were individually critically appraised and the details are reported elsewhere.[3] Overall, there was substantial variation in study quality. However, large multicentre trials comparing newer anticoagulants with standard adjusted dose VKA such as ENGAGE AF-TIMI 48 (n=21,105), ARISTOTLE (n = 18,201), RE-LY (n = 18,113), and ROCKET-AF (n = 14,264), which account for the vast majority of patients included in the systematic review, appear to be methodologically rigorous.

Stroke or Systemic Embolism

The evidence network (Figure 1) for the primary analysis was comprised of twelve RCTs representing eight treatments in addition to placebo/observation (N =82,396). Four RCTs (PETRO,[24] WASPO[25], Chung et al,[26] PETRO,[24] WASPO[25], and Yamashita et al[27]) were not included in the analysis because they did not report data for this outcome or had zeroes in both arms.

Apixaban and Ddabigatran (150 mg twice daily) and apixaban were associated with reductions in stroke or SE relative to standard adjusted dose VKA (Figure 2). The use of these two agents led to absolute risk reductions ranging from 4 to 6 fewer events per 1,000 patients treated each year. By contrast, low-dose ASA and the combination of clopidogrel plus low-dose ASA appeared to

For peer review only

have a higher risk of stroke or SE than standard adjusted dose VKA, leading to an increase in the number of stroke or SE ranging from 14 to 15 more events per 1,000 patients treated each year (Figure 4 and Appendix 6). No differences were detected between standard adjusted dose VKA and each of the following interventions: rivaroxaban, edoxaban (30 mg or 60 mg daily), dabigatran (110 mg twice daily), edoxaban (30 mg or 60 mg daily), rivaroxaban, and medium-dose ASA (Figure 3).

The estimates of effect derived from the direct pair-wise comparisons aligned well with those obtained from the network meta-analysis in both direction and magnitude (Appendix 6). Furthermore, the posterior mean residual deviance (29.41) is close to the number of unconstrained data points (27), which is an indication of reasonable model fit. A number of sub-group analyses and alternative modeling strategies were conducted on the primary analysis, including adjusting for $CHADS_2$, TTR, and age (Table 1). We also conducted a sensitivity analysis where we included the AVERROES[28] and ACTIVE A[29] trials (Appendix 12). These studies were excluded from the main analysis because we required patients to be eligible for anticoagulant therapy, including treatment with a VKA. The results differed slightly from the reference case (Appendix 12). The primary analysis was conducted using a fixed-effects model because the network was comprised of single study connections; these results were also compared against those obtained using a random-effects model with informative and vague priors on the standard deviation, and found to be similar in magnitude although wider credible intervals were observed (Appendix 9).

For peer review only

Major bleeding

The evidence network for the primary analysis for major bleeding was comprised of 15 RCTs representing eight treatments in addition to placebo/observation (N =83,015). The evidence network for major bleeding is similar to the evidence network for stroke or SE (Figure 1) but includes one extra RCT (WASPO[25]) comparing medium dose ASA with standard adjusted dose VKA, and two extra RCTs (Chung et al,[26] and Yamashita et al[27]) comparing edoxaban with standard adjusted dose VKA (-Appendix 5).

Edoxaban 30 mg daily, Aapixaban, edoxaban 60 mg daily, and dabigatran 110 mg twice daily, edoxaban 30 mg daily, and edoxaban 60 mg daily were associated with reductions in the risk of major bleeding compared with standard adjusted dose VKA (Figure 2). No differences for major bleeding were detected between standard adjusted dose VKA and each of the remaining interventions: rivaroxaban, dabigatran 150 mg twice daily, rivaroxaban, clopidogrel plus low-dose ASA, and all ASA dosages (Figure 2). The absolute risk difference of major bleeding relative to standard adjusted dose VKA ranged from 18 fewer to 24 more events per 1000 patients treated per year **(**Figure 4 and Appendix 7**)**. A summary of the results for the Bayesian and direct pair-wise meta-analyses is shown in Appendix 7, and Bayesian network meta-analysis results are represented graphically in Figure 2. Complete results from the Bayesian network meta-analysis for all possible comparisons are presented in Figure 3**.**

The estimates of effect derived from the direct pair-wise comparisons aligned well with those obtained from the network meta-analysis in both direction and magnitude (Appendix 7). Furthermore, the posterior mean residual deviance (38.0) is close to the number of unconstrained

For peer review only

data points (35), which is an indication of reasonable model fit. A number of sub-group analyses and alternative modeling strategies were conducted on the primary analysis (Table 1). We also conducted a sensitivity analysis where we included the AVERROES[28] and ACTIVE A[29] trial (Appendix 12), as well as analyses using a random-effects model with vague and informative priors (Appendix 9).

Benefit-Harm Assessment – Stroke or systemic embolism versus major bleeding

Figure 3 summarises the results for all pair-wise comparisons derived from the Bayesian fixed-effects network meta-analysis (Appendix 9). Results relative to standard adjusted dose VKA have been discussed above. For pair-wise comparisons among newer oral anticoagulants for stroke or SE, dabigatran 150 mg twice daily was associated fewer events versus dabigatran 110 mg twice daily, rivaroxaban, edoxaban 30mg daily, and edoxaban 60 mg daily, and rivaroxaban. Apixaban , and rivaroxaban were also associated with fewer events compared to edoxaban 30mg daily. There were no differences among low-dose ASA, medium-dose ASA, and clopidogrel plus low-dose ASA. However, low-dose ASA and the combination of clopidogrel plus low-dose ASA were associated with increases in stroke or systemic embolism compared to all newer oral anticoagulants. For major bleeding, apixaban, dabigatran 110 mg twice daily were associated with fewer events versus dabigatran 150 mg twice daily and rivaroxaban. Edoxaban 30 mg daily was associated with fewer events than other new oral anticoagulants, while edoxaban 60 mg daily was associated with fewer events compared with rivaroxaban and clopidogrel plus low-dose ASA. The risk of major bleeding for apixaban was lower compared to clopidogrel plus low-dose ASA. There were no differences associated with the ASA treatments, both for comparisons among themselves and compared to the anticoagulant treatments.

For peer review only

Figure 4 plots the absolute risk for stroke or SE and major bleeding per 1000 treated for the 10 treatments analyzed. Data in Figure 4 were obtained from the results presented in Appendix 6 and 7. Examination of the data in Figure 4 suggests that the balance of benefits and harms of the new oral anticoagulants for stroke or systemic embolism and major bleeding is positive compared to standard adjusted dose VKA (decrease in stroke or systemic embolism and/or major bleeding) and largely similar among one another. Comparison of all anticoagulant treatment (including standard adjusted dose VKA) to ASA, or ASA in combination with clopidogrel indicates that whereas the new oral anticoagulants decrease the risk of both stroke or systemic embolism and major bleeding, ASA has a less favorable balance of benefits and harms, i.e., fails to minimize the risk of stroke or systemic embolism and/or major bleeding.

DISCUSSION

We identified 16 RCTs comparing antithrombotic agents for the prevention of all cause stroke or systemic embolism and major bleeding in patients with AF. To the best of our knowledge, this is the most up-to-date systematic review and network meta-analysis[30–34] to synthesize the available benefits and harms on newer oral anticoagulants, standard adjusted dose VKA, and antiplatelet agents for the prevention of stroke and major bleeding in patients with non-valvular AF (Appendix 13). Harenberg et al[30] recently summarized the published indirect comparisons and network meta-analyses evaluating newer oral anticoagulants for AF and an earlier unpublished version of our analysis[17] was considered among the most comprehensive analyses at that time.[30] This network meta-analysis expands upon that unpublished report.[17] Overall, our findings align with those reported in previously published network meta-analyses;[30,31,33] however, unlike

For peer review only

previous analyses,[30] we include recently published edoxaban data,[20] and also include antiplatelet agents. We also present detailed evidence networks[35] to illustrate the body of evidence for each outcome; report analysis using multiple statistical models (Appendix 9); report detailed *a priori* sub-group analyses for each outcome using data derived from FDA Public Summary Documents;[9,18] present findings on both the relative and absolute scale,[36,37] and graphically illustrate results using icon arrays. Our network meta-analysis was registered in PROSPERO, and also adheres to PRISMA reporting standards (Appendix 10). It also differs from a recently published traditional meta-analysis by Ruff et al[32] given we report comparison of newer oral anticoagulants versus each other, and also include comparisons with antiplatelet agents.

For stroke or systemic embolism, ~~apixaban and~~ dabigatran 150 mg and apixaban twice daily were associated with reductions in stroke or systemic embolism relative to standard adjusted dose VKA. By contrast, low-dose ASA and the combination of clopidogrel plus low-dose ASA appeared were associated with increases in stroke or systemic embolism relative to standard adjusted dose VKA. No differences were detected between standard adjusted dose VKA and each of the following interventions: ~~rivaroxaban,~~ dabigatran 110 mg twice daily, edoxaban 30 and 60 mg daily, rivaroxaban, medium-dose ASA, and no treatment/placebo. The absolute risk reduction of stroke or systemic embolism relative to standard adjusted dose VKA ranged from 6 fewer to 15 more events per 1000 patients treated per year, with dabigatran 150 mg twice daily yielding the largest aboslute risk reduction and clopidogrel plus low-dose ASA the largest absolute risk increase.

For peer review only - http://bmjopen.bmj.com/site/about/guidelines.xhtml

For major bleeding, edoxaban 30 mg daily, apixaban, edoxaban 60 mg daily, and dabigatran 110 mg twice daily , edoxaban 30 mg daily, and edoxaban 60 mg daily were associated with a decreased -risk of major bleeding relative to standard adjusted dose VKA. No differences for major bleeding were detected between standard adjusted dose VKA and each of the remaining interventions: rivaroxaban, dabigatran 150 mg twice daily, rivaroxaban, clopidogrel plus low-dose ASA, and all ASA dosages. The absolute risk difference of major bleeding relative to standard adjusted dose VKA ranged from 18 fewer to 24 more events per 1000 patients treated, with edoxaban 30 mg daily yielding the largest absolute risk reduction and medium dose ASA the largest absolute risk increase. Results of network meta-analyses reflect the relative efficacy and safety profile of antiplatelet and anticoagulant drugs as documented by large clinical trials. Thus, international guidelines[1,2] for prevention of stroke in patients with AF also recommended oral anticoagulation over antiplatelet therapy as the latter had only modest protective effects. With respect to the new oral anticoagulants none of the guidelines has given a recommendation to prefer one of the new oral anticoagulants over another.[1,2]

Network meta-analysis involves pooling of trials. We observed variability across studies included in the evidence network in terms of $CHADS_2$, age and TTR (Appendix 3 and 4). For example, patients using rivaroxaban had a higher risk of stroke because ROCKET-AF[22] included patients with $CHADS_2 \geq 2$. As such, we conducted sub-group analysis to adjust for differences in patient and study-level characteristics. We derived data for sub-group analyses from FDA Public Summary documents[9,18,19] which enhanced our ability to account for variability across studies (e.g., use intention to treat (ITT) population for rivaroxaban versus per-protocol as-treated population reported in publication[22]). Results from these sub-group analyses differed slightly

For peer review only - http://bmjopen.bmj.com/site/about/guidelines.xhtml

from those reported in the primary analysis, indicating that there may be differences in the benefits and harms across sub-groups. This becomes more apparent when sub-group analyses are reported on the absolute scale.[3] It is unclear whether the absolute risk reductions associated with these differences translate into clinically meaningful benefits in practice, especially in sub-populations of patients with a lower baseline risk of stroke or systemic embolism. To fully elucidate the comparative benefits and harms of antithrombotic agents across the various sub-populations, rigorously conducted comparative studies,[38,39] or network meta-regression analyses of patient-level data[40] are required.

There are several limitations with the analysis we employed. First, there is notable heterogeneity in patients and study characteristics (Appendix 3, 4). However, the small number of studies limits the analyses that can be conducted to account for heterogeneity in the absence of patient-level data. Second, there is insufficient sub-group data available for other outcomes reported in the RCTs included. As a consequence, detailed analysis was limited to two outcomes – stroke and SE and major bleeding. Third, we reported the results from the fixed-effects model in the main text. We felt that this was appropriate as the nodes in evidence networks are connected largely by single studies. Effect estimates derived from the fixed-effects model aligned more closely with the direct estimates. Reporting results from the fixed-effects model in the main text likely biases results in favour of the new oral anticoagulants, as treatments that achieved statistically significant results in the primary RCTs retained statistically significant findings when using the fixed-effects model, but not always when using the random-effects model due to the prior[11] on the between study variance. Nevertheless, the results from the random-effects model have also been reported in Appendix 9, along with detailed discussion around the

For peer review only

limitations in using the fixed-effects model. Fourth, the absolute risk reduction for the network meta-analysis was calculated using the event rate in the standard adjusted dose VKA arm of the RE-LY study. The RE-LY trial was selected because it contained data for both $CHADS_2 < 2$ and $CHADS_2 \geq 2$ and had detailed data made publicly available following an FDA review of dabigatran. Use of a different study (e.g., ARISTOTLE) for baseline event rate data is unlikely to change the results substantially given that the event rates in standard adjusted dose VKA arms were similar across studies. Fifth, we present findings using graphics that have been largely applied in the network meta-analysis literature,[41] such as a table with odds ratios for all pair-wise comparisons. This method of presenting findings on the relative scale is problematic when you are conducting assessments to compare the balance of benefits and harms, which need to be standardized. As such, we also provided results in absolute terms. We report our findings using icon arrays, although it should be noted that these results do not account for the utility values based upon patient preferences for each of the outcomes,[42,43] nor do they reflect uncertainty although we report absolute reductions on the risk benefit plane reflecting uncertainty in Appendix 8. There are other methods available that can incorporate patient preferences for outcomes.[42,43] Further, estimates of benefit and harm with several of the therapies (e.g., rivaroxaban) come from one trial and thus such data are not particularly robust. Further research is needed comparing the balance of benefit and harms using other research methodologies, incorporating other relevant outcomes, patient preferences for each of the outcomes,[42,43] and additional studies when they become available. Finally, haemorrhagic stroke is also considered a major bleed in underlying studies. Accordingly, we may have double counted haemorrhage stroke in this analysis. Unfortunately, we are not able to account for this issue in all included studies. Nonetheless, it is important to note this limitation and highlight that this potentially

For peer review only - http://bmjopen.bmj.com/site/about/guidelines.xhtml

biases results in favor of newer oral anticoagulants given these agents were associated with reductions in these events.

CONCLUSIONS

Compared with standard adjusted dose VKA, ~~apixaban and~~ dabigatran 150 mg and apixaban twice daily were associated with reductions in the incidence of stroke or systemic embolism in patients with AF, whereas, low-dose ASA and the combination of clopidogrel plus low-dose ASA increased the risk relative to standard adjusted dose VKA at preventing stroke or systemic embolism. ~~Apixaban, dabigatran 110 mg twice daily, E~~edoxaban 30 mg daily, apixaban, edoxaban 60 mg daily, and dabigatran 110 mg twice daily ~~edoxaban 60 mg daily~~ and was associated with a reduced risk in major bleeding compared with standard adjusted dose VKA. Although the results of the current review revealed that there were differences in stroke or systemic embolism and major bleeding among antithrombotic agents in the management of patients with AF in relative terms, it is unclear whether the absolute risk reductions associated with these differences translate into clinically meaningful benefits in practice, especially in sub-populations of patients with a lower baseline risk of stroke or systemic embolism. Rigorously conducted comparative studies or network meta-regression analyses of patient-level data are required to fully elucidate the comparative benefits and harms of antithrombotic agents across the various sub-populations.

For peer review only

For peer review only - http://bmjopen.bmj.com/site/about/guidelines.xhtml

TABLES

Table 1: Sub-group and sensitivity analyses for stroke or systemic embolism and major bleeding relative to standard adjusted dose VKA, Odds ratios ± 95% Credible Intervals versus standard adjusted dose VKA

Treatment	Reference Case – Fixed Effect NMA	Subgroup analysis by CHADS$_2$ Score		Subgroup analysis by Age		Subgroup analysis by TTR	
		CHADS$_2$ < 2	CHADS$_2$ ≥ 2	Age < 75 years	Age ≥ 75 years	TTR < 66%	TTR ≥ 66%
**Stroke or Systemic Embolism**							
~~**Apixaban 5 mg twice daily**~~	~~**0.78<br>(0.65,0.94)**~~	~~0.86<br>(0.57,1.28)~~	~~**0.78<br>(0.63,0.95)**~~	~~0.85<br>(0.67,1.07)~~	~~**0.71<br>(0.53,0.95)**~~	~~0.79<br>(0.62,1.003)~~	~~0.79<br>(0.60,1.05)~~
~~**Dabigatran 110 mg twice daily**~~	~~0.91<br>(0.74,1.12)~~	~~1<br>(0.64,1.55)~~	~~0.89<br>(0.71,1.12)~~	~~0.93<br>(0.7,1.24)~~	~~0.88<br>(0.66,1.19)~~	~~0.91<br>(0.69,1.19)~~	~~0.9<br>(0.66,1.24)~~
**Dabigatran 150 mg twice daily**	**0.66<br>(0.53,0.82)**	0.61<br>(0.37,1)	**0.66<br>(0.52,0.85)**	0.63<br>(0.46,0.87)	**0.66<br>(0.48,0.9)**	0.54<br>(0.39,0.73)	0.81<br>(0.58,1.11)
**Apixaban 5 mg twice daily**	**0.78<br>(0.65,0.94)**	0.86<br>(0.57,1.28)	**0.78<br>(0.63,0.95)**	0.85<br>(0.67,1.07)	**0.71<br>(0.53,0.95)**	0.79<br>(0.62,1.003)	0.79<br>(0.60,1.05)
**Edoxaban 60 mg daily**	0.87<br>(0.74,1.02)	NA	0.87<br>(0.74,1.01)	0.91<br>(0.73,1.14)	0.83<br>(0.66,1.04)	0.83<br>(0.65,1.07)¶	0.91<br>(0.73,1.13)¶
**Rivaroxaban 20 mg daily**	0.88<br>(0.74,1.04)	NA	0.88<br>(0.74,1.04)	0.95<br>(0.75,1.2)	0.8<br>(0.63,1.03)	0.81<br>(0.65,1.01)	0.72<br>(0.47,1.1)
**Dabigatran 110 mg twice daily**	0.91<br>(0.74,1.12)	1<br>(0.64,1.55)	0.89<br>(0.71,1.12)	0.93<br>(0.7,1.24)	0.88<br>(0.66,1.19)	0.91<br>(0.69,1.19)	0.9<br>(0.66,1.24)
**Edoxaban 30 mg daily**	1.14<br>(0.98,1.32)	NA	1.14<br>(0.98,1.32)	1.16<br>(0.94,1.44)	1.13<br>(0.91,1.4)	1.01<br>(0.80,1.28)¶	1.24<br>(1.01,1.24)¶
~~**Rivaroxaban 20 mg daily**~~	~~0.87<br>(0.74,1.03)~~	~~NA~~	~~0.88<br>(0.74,1.04)~~	~~0.95<br>(0.75,1.2)~~	~~0.8<br>(0.63,1.03)~~	~~0.81<br>(0.65,1.01)~~	~~0.72<br>(0.47,1.1)~~
~~**Low Dose ASA (≤ 100 mg daily)**~~	~~**1.87<br>(1.26,2.8)**~~	~~2.2<br>(1.18,4.25)~~	~~2.1<br>(0.91,5.15) *~~	~~NA~~	~~2.04<br>(1.33,3.20)~~	~~NA~~	~~NA~~
**Medium Dose ASA (> 100 mg and ≤ 300 mg daily)**	1.35<br>(0.74,2.47)	1.97<br>(0.63,6.73) **	NA	NA	NA	NA	NA
**Low Dose ASA (≤ 100 mg daily)**	**1.87<br>(1.26,2.8)**	2.2<br>(1.18,4.25)	2.1<br>(0.91,5.15) *	NA	2.04<br>(1.33,3.20)	NA	NA
**Clopidogrel 75 mg daily & Low dose Aspirin (≤ 100 mg daily)**	**1.93<br>(1.42,2.645)**	3.16<br>(1.39,8.22)	1.15<br>(0.84,1.57)	NA	NA	1.52<br>(0.96,2.45)	**2.4<br>(1.59,3.71)**
~~**Edoxaban 30 mg daily**~~	~~1.14<br>(0.98,1.32)~~	~~NA~~	~~1.14<br>(0.98,1.32)~~	~~1.16<br>(0.94,1.44)~~	~~1.13<br>(0.91,1.4)~~	~~1.01<br>(0.80,1.28)¶~~	~~1.24<br>(1.01,1.24)¶~~
~~**Edoxaban 60 mg daily**~~	~~0.87~~		~~0.87~~				

Formatted Table

Formatted Table

1

For peer review only

Treatment	Reference Case – Fixed Effect NMA	Subgroup analysis by CHADS 2 Score		Subgroup analysis by Age		Subgroup analysis by TTR	
		$CHADS_2 < 2$	$CHADS_2 \geq 2$	Age < 75 years	Age ≥ 75 years	TTR < 66%	TTR ≥ 66%
	(0.74,1.01)	NA	(0.74,1.01)	0.91 (0.73,1.14)	0.83 (0.66,1.04)	0.83 (0.65,1.07)¶	0.91 (0.73,1.13)¶
**Major Bleeding**							
Apixaban 5 mg twice daily	0.69 (0.6,0.8)	0.59 (0.44,0.78)	0.73 (0.62,0.87)	0.73 (0.6,0.89)	0.65 (0.52,0.80)	0.57 (0.45,0.71)	0.81 (0.67,0.98)
Edoxaban 30 mg daily	0.46 (0.4,0.54)	NA	0.46 (0.4,0.54)	0.50 (0.41,0.61) ‡	0.51 (0.41-0.62) ‡	0.45 (0.35,0.58) ‡¶	0.51 (0.43,0.61) ‡¶
Apixaban 5 mg twice daily	0.69 (0.6,0.8)	0.59 (0.44,0.78)	0.73 (0.62,0.87)	0.73 (0.6,0.89)	0.65 (0.52,0.80)	0.57 (0.45,0.71)	0.81 (0.67,0.98)
Edoxaban 60 mg daily	0.79 (0.69,0.9)	NA	0.79 (0.69,0.9)	0.74 (0.62-0.90) ‡	0.82 (0.68-0.98) ‡	0.69 (0.55,0.87) ‡¶	0.83 (0.70,0.97) ‡¶
Dabigatran 110 mg twice daily	0.8 (0.69,0.93)	0.65 (0.47,0.89)	0.86 (0.73,1.02)	0.62 (0.49,0.77)	1.02 (0.84,1.25)	0.74 (0.61,0.91)	0.86 (0.69,1.06)
Dabigatran 150 mg twice daily	0.93 (0.81,1.08)	0.77 (0.57,1.04)	1.01 (0.86,1.19)	0.7 (0.56,0.86)	1.19 (0.98,1.45)	0.76 (0.62,0.94)	1.15 (0.94,1.4)
Rivaroxaban 20 mg daily	1.03 (0.89,1.19)	NA	1.03 (0.89,1.19)	0.93 (0.76,1.13)	1.15 (0.93,1.41)	0.92 (0.77,1.1)	1.3 (1.01,1.69)
Dabigatran 110 mg twice daily	0.8 (0.69,0.93)	0.65 (0.47,0.89)	0.86 (0.73,1.02)	0.62 (0.49,0.77)	1.02 (0.84,1.25)	0.74 (0.61,0.91)	0.86 (0.69,1.06)
Dabigatran 150 mg twice daily	0.93 (0.8,1.08)	0.77 (0.57,1.04)	1.01 (0.86,1.19)	0.7 (0.56,0.86)	1.19 (0.98,1.45)	0.76 (0.62,0.94)	1.15 (0.94,1.4)
Rivaroxaban 20 mg daily	1.03 (0.89,1.18)	NA	1.03 (0.89,1.19)	0.93 (0.76,1.13)	1.15 (0.93,1.41)	0.92 (0.77,1.1)	1.3 (1.01,1.69)
Low Dose ASA (≤ 100 mg daily)	1.05 (0.6,1.876)	0.83 (0.42,1.64)	1.6 (0.55,5.02) *	NA	1.01 (0.57,1.78)	NA	NA
Clopidogrel 75 mg daily & Low dose Aspirin (≤ 100 mg daily)	1.1 (0.83,1.47)	1.51 (0.89,2.61)	0.97 (0.69,1.36) †	NA	NA	0.66 (0.42,1.02)	1.66 (1.12,2.47)
Medium Dose ASA (> 100 mg and ≤ 300 mg daily)	1.79 (0.63,5.67)	1.48 (0.11,19.07)**	NA	NA	NA	NA	NA
Clopidogrel 75 mg daily & Low dose Aspirin (≤ 100 mg daily)	1.1 (0.83,1.47)	1.51 (0.89,2.61)	0.97 (0.69,1.36) †	NA	NA	0.66 (0.42,1.02)	1.66 (1.12,2.47)
Edoxaban 30 mg daily	0.46 (0.4,0.54)	NA	0.46 (0.4,0.54)	0.50 (0.41,0.61) ‡	0.51 (0.41-0.62) ‡	0.45 (0.35,0.58) ‡¶	0.51 (0.43,0.61) ‡¶

Formatted Table

Formatted Table

Formatted Table

Formatted Table

Formatted Table

Formatted Table

Treatment	Reference Case – Fixed Effect NMA	Subgroup analysis by $CHADS_2$ Score		Subgroup analysis by Age		Subgroup analysis by TTR	
		$CHADS_2 < 2$	$CHADS_2 \geq 2$	Age < 75 years	Age ≥ 75 years	TTR < 66%	TTR ≥ 66%
~~Edoxaban 60 mg daily~~	~~0.79 (0.69,0.9)~~	~~NA~~	~~0.79 (0.69,0.9)~~	~~0.74 (0.62-0.90) ‡~~	~~0.82 (0.68-0.98) ‡~~	~~0.69 (0.55,0.87) ‡[¶]~~	~~0.83 (0.70,0.97) ‡[¶]~~

ASA= acetylsalicylic acid; mg= milligram; NA=not available; NMA= network meta-analysis; OR=odds ratio; TTR= time in therapeutic range; VKA=vitamin K antagonist

* Results may be slightly biased against LDASA. Derived from BAFTA study where $CHADS_2$ subgroup data stratified by $CHADS_2$ 1-2 (vs. 0-1) and $CHADS_2$ 3-6 (vs. 2-6).

** Results may be slightly biased against medium-dose ASA. Based on study level results from CAFA and JAST studies. Although these studies consisted primarily of low-risk populations, some patients may have $CHADS_2$ scores greater than 2.

[†] Estimated from subgroup study by Healey et al 2008

¶ Data provided from ENGAGE AF-TIMI 48[20] uses TTR greater than 60%

‡ Based on overall time period where overall time period was defined as first dose to the last dose plus 3 days.

For peer review only

For peer review only - http://bmjopen.bmj.com/site/about/guidelines.xhtml

FIGURES

Figure 1: Evidence network for all cause stroke or systemic embolism.* The width of the lines is proportional to the number of randomized controlled trials comparing each pair of treatments, and the size of each treatment node is proportional to the number of randomised participants (sample size). A dotted line indicates a three-arm randomized controlled trial and a black node indicates a node included in the analysis but not reported in the main text.

For peer review only

Formatted: Font: (Default) Arial, 11 pt

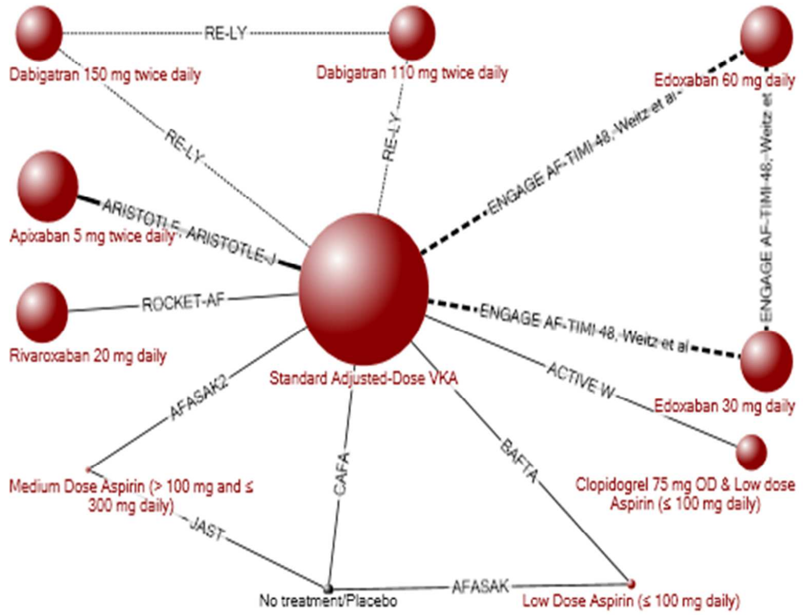

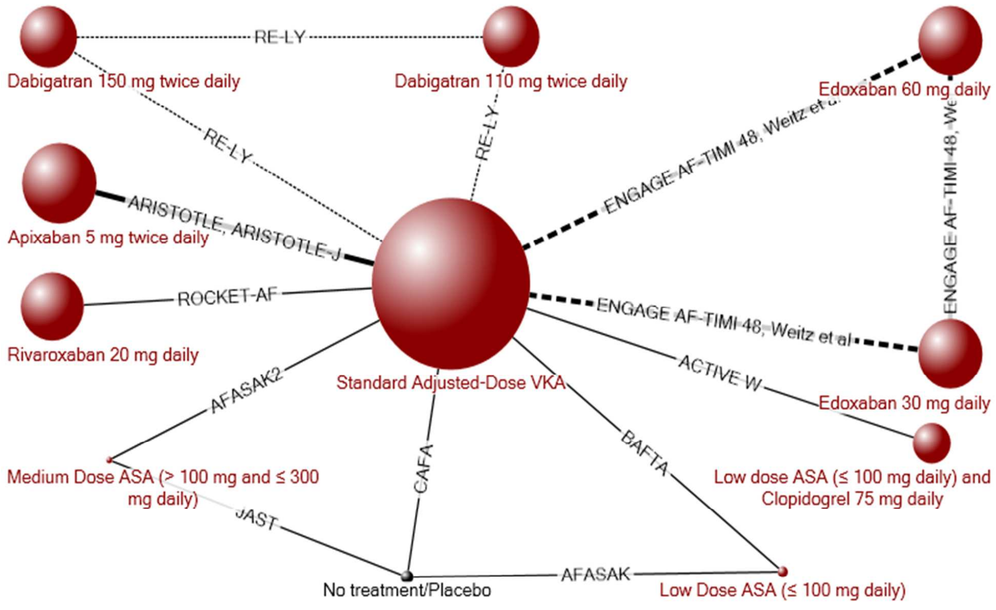

ASA= acetylsalicylic acid; mg= milligram; RCT= RCT = randomized controlled trial; VKA= vitamin K antagonist
*Four RCTs (PETRO,[24] WASPO[25], Chung et al,[26] and Yamashita et al[27]) were not included in the analysis because they did not report data for this outcome or had zeroes in both arms

Figure 2: Odds ratio for all cause stroke or systemic embolism (A) and major bleeding (B) in Bayesian network meta-analysis versus standard adjusted dose VKA.

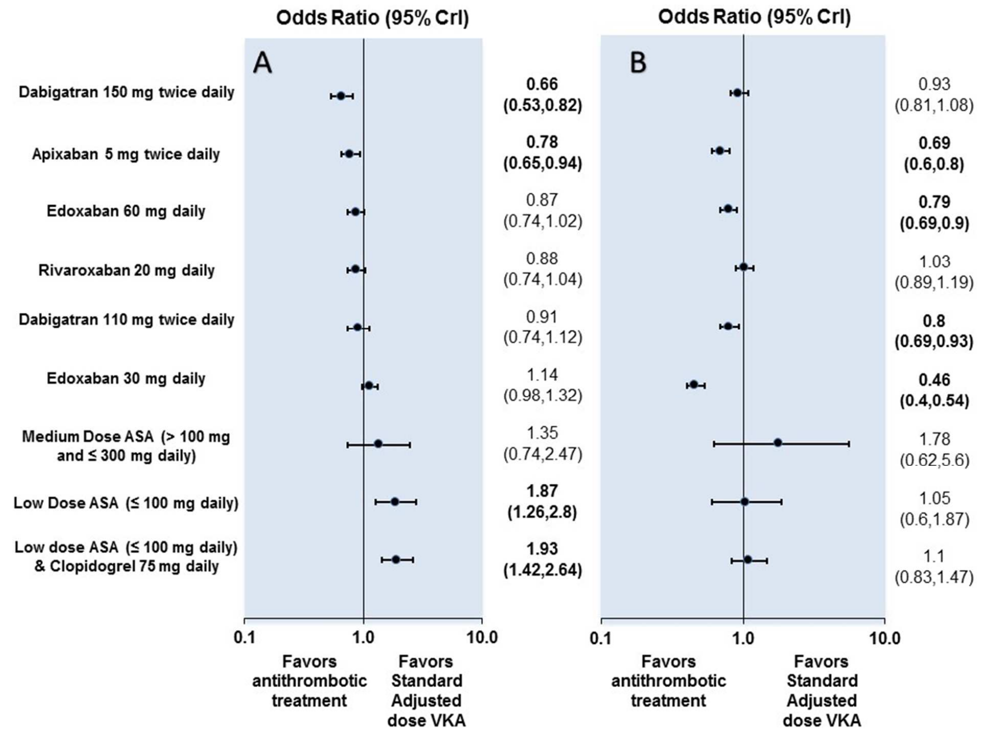

Formatted: Font: (Default) Arial, 11 pt

For peer review only - http://bmjopen.bmj.com/site/about/guidelines.xhtml

Formatted: Font: (Default) Arial, 11 pt

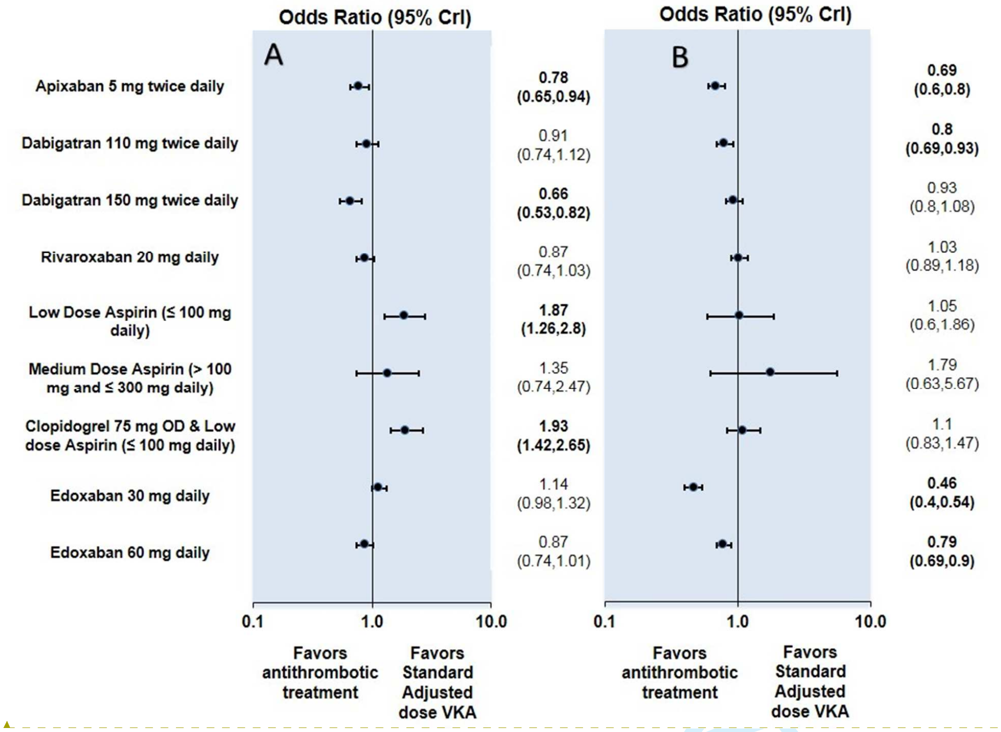

CrI= credible interval; mg= milligram; VKA= vitamin K antagonist

Figure 3: Odds Ratios (OR) from network meta-analyses for stroke or systemic embolism and major bleeding for all pair-wise comparisons.* ORs for recurrence of stroke or systemic embolism are below the diagonal (row-defining treatment versus column-defining treatment) and

7

view only

those for major bleeding are above the diagonal and in blue (column-defining treatment versus row-defining treatment). To obtain ORs for comparisons in the opposite direction, reciprocals should be taken (e.g., the OR for standard dose warfarin compared with apixaban 5 mg twice daily for stroke or systemic embolism is 1/0.78=1.28). Significant results are in bold and underscored (and in larger font).

Standard Adjusted-Dose VKA	0.93 (0.81,1.08)	**0.69 (0.6,0.8)**	**0.79 (0.69,0.9)**	1.03 (0.89,1.19)	**0.8 (0.69,0.93)**	**0.46 (0.4,0.54)**	1.78 (0.62,5.6)	1.05 (0.6,1.87)	1.1 (0.83,1.47)
**0.66 (0.53,0.82)**	Dabigatran 150 mg twice daily	**0.74 (0.6,0.91)**	0.84 (0.69,1.03)	1.1 (0.9,1.35)	0.86 (0.74,1.003)	**0.5 (0.4,0.61)**	1.91 (0.66,6.08)	1.13 (0.63,2.04)	1.18 (0.85,1.63)
**0.78 (0.65,0.94)**	1.19 (0.89,1.58)	Apixaban 5 mg twice daily	1.14 (0.93,1.38)	**1.49 (1.21,1.82)**	1.16 (0.94,1.43)	**0.67 (0.54,0.83)**	2.57 (0.89,8.18)	1.52 (0.85,2.75)	**1.59 (1.16,2.2)**
0.87 (0.74,1.02)	**1.31 (1.002,1.73)**	1.11 (0.87,1.41)	Edoxaban 60 mg daily	**1.31 (1.08,1.59)**	1.02 (0.83,1.25)	**0.59 (0.5,0.69)**	2.27 (0.78,7.17)	1.34 (0.75,2.41)	**1.4 (1.02,1.92)**
0.88 (0.74,1.04)	**1.33 (1.01,1.76)**	1.12 (0.87,1.43)	1.01 (0.8,1.28)	Rivaroxaban 20 mg daily	**0.78 (0.63,0.96)**	**0.45 (0.37,0.56)**	1.74 (0.6,5.48)	1.02 (0.57,1.85)	1.07 (0.78,1.48)
0.91 (0.74,1.12)	**1.38 (1.11,1.74)**	1.17 (0.88,1.53)	1.05 (0.81,1.37)	1.04 (0.8,1.36)	Dabigatran 110 mg twice daily	**0.58 (0.47,0.72)**	2.22 (0.77,7.03)	1.31 (0.73,2.37)	1.37 (0.99,1.9)
1.14 (0.98,1.32)	**1.73 (1.32,2.26)**	**1.45 (1.15,1.84)**	**1.31 (1.13,1.54)**	**1.3 (1.04,1.63)**	1.25 (0.97,1.61)	Edoxaban 30 mg daily	**3.85 (1.32,12.18)**	**2.27 (1.26,4.1)**	**2.38 (1.72,3.3)**
1.35 (0.74,2.47)	**2.04 (1.08,3.9)**	1.72 (0.92,3.24)	1.56 (0.84,2.91)	1.54 (0.83,2.88)	1.48 (0.79,2.8)	1.18 (0.64,2.21)	Medium Dose ASA (> 100 mg and ≤ 300 mg daily)	0.59 (0.17,1.94)	0.62 (0.19,1.84)
**1.87 (1.26,2.8)**	**2.84 (1.81,4.5)**	**2.39 (1.55,3.72)**	**2.16 (1.42,3.34)**	**2.14 (1.4,3.31)**	**2.05 (1.32,3.23)**	**1.64 (1.08,2.53)**	1.39 (0.74,2.61)	Low Dose ASA (≤ 100 mg daily)	1.05 (0.55,1.97)
**1.93 (1.42,2.64)**	**2.93 (2.01,4.3)**	**2.46 (1.73,3.54)**	**2.23 (1.58,3.17)**	**2.2 (1.55,3.14)**	**2.11 (1.46,3.07)**	**1.69 (1.21,2.4)**	1.43 (0.73,2.81)	1.03 (0.62,1.7)	Low dose ASA (≤ 100 mg daily) & Clopidogrel 75 mg daily

~~Standard Adjusted-Dose VKA~~	~~**0.69 (0.6,0.8)**~~	~~**0.8 (0.69,0.93)**~~	~~0.93 (0.8,1.08)~~	~~1.03 (0.89,1.18)~~	~~1.05 (0.6,1.86)~~	~~1.79 (0.63,5.67)~~	~~1.1 (0.83,1.47)~~	~~**0.46 (0.4,0.54)**~~	~~**(0**~~
~~**0.78 (0.65,0.94)**~~	~~Apixaban 5 mg twice daily~~	~~1.16 (0.94,1.43)~~	~~**1.35 (1.1,1.65)**~~	~~**1.48 (1.21,1.82)**~~	~~1.52 (0.85,2.74)~~	~~2.59 (0.9,8.26)~~	~~**1.59 (1.15,2.19)**~~	~~**0.67 (0.54,0.83)**~~	~~(0.~~
~~0.91 (0.74,1.12)~~	~~1.17 (0.89,1.54)~~	~~Dabigatran 110 mg twice daily~~	~~1.16 (0.9976,1.36)~~	~~**1.28 (1.04,1.58)**~~	~~1.32 (0.73,2.37)~~	~~2.24 (0.77,7.16)~~	~~1.37 (0.99,1.9)~~	~~**0.58 (0.47,0.72)**~~	~~(0~~

For peer review only - http://bmjopen.bmj.com/site/about/guidelines.xhtml

~~**0.66 (0.53,0.82)**~~	~~0.84 (0.63,1.12)~~	~~**0.72 (0.57,0.91)**~~	~~Dabigatran 150 mg twice daily~~	~~1.1 (0.9,1.35)~~	~~1.13 (0.63,2.04)~~	~~1.93 (0.67,6.15)~~	~~1.18 (0.85,1.63)~~	~~**0.5 (0.4,0.61)**~~	~~(0.~~
~~0.87 (0.74,1.03)~~	~~1.12 (0.87,1.43)~~	~~0.96 (0.74,1.25)~~	~~**1.33 (1.01,1.75)**~~	~~Rivaroxaban 20 mg daily~~	~~1.03 (0.57,1.85)~~	~~1.75 (0.6,5.56)~~	~~1.07 (0.78,1.48)~~	~~**0.45 (0.37,0.56)**~~	~~**(0.**~~
~~**1.87 (1.26,2.8)**~~	~~**2.4 (1.55,3.72)**~~	~~**2.05 (1.32,3.23)**~~	~~**2.85 (1.81,4.5)**~~	~~**2.14 (1.4,3.31)**~~	~~Low Dose Aspirin (≤ 100 mg daily)~~	~~1.7 (0.52,6.08)~~	~~1.05 (0.55,1.97)~~	~~**0.44 (0.24,0.79)**~~	~~(0.~~
~~1.35 (0.74,2.47)~~	~~1.72 (0.92,3.25)~~	~~1.48 (0.78,2.79)~~	~~**2.05 (1.08,3.9)**~~	~~1.54 (0.83,2.88)~~	~~0.72 (0.38,1.35)~~	~~Medium Dose Aspirin (> 100 mg and ≤ 300 mg daily)~~	~~0.61 (0.19,1.83)~~	~~**0.26 (0.08,0.75)**~~	~~(0.~~
~~**1.93 (1.42,2.65)**~~	~~**2.47 (1.73,3.55)**~~	~~**2.12 (1.46,3.08)**~~	~~**2.93 (2.01,4.31)**~~	~~**2.21 (1.56,3.15)**~~	~~1.03 (0.62,1.71)~~	~~1.43 (0.73,2.81)~~	~~Clopidogrel 75 mg OD & Low dose Aspirin (≤ 100 mg daily)~~	~~**0.42 (0.3,0.58)**~~	~~**(0.**~~
~~1.14 (0.98,1.32)~~	~~**1.45 (1.15,1.84)**~~	~~1.25 (0.97,1.61)~~	~~**1.73 (1.32,2.26)**~~	~~**1.3 (1.04,1.63)**~~	~~**0.61 (0.4,0.92)**~~	~~0.84 (0.45,1.56)~~	~~**0.59 (0.42,0.83)**~~	~~Edoxaban 30 mg daily~~	~~(1.~~
~~0.87 (0.74,1.01)~~	~~1.11 (0.87,1.41)~~	~~0.95 (0.73,1.23)~~	~~**1.31 (1,1.73)**~~	~~0.99 (0.79,1.25)~~	~~**0.46 (0.3,0.7)**~~	~~0.64 (0.34,1.19)~~	~~**0.45 (0.32,0.63)**~~	~~**0.76 (0.65,0.89)**~~	~~Edox~~

review only

For peer review only - http://bmjopen.bmj.com/site/about/guidelines.xhtml

Standard Adjusted-Dose VKA	**0.69 (0.6,0.8)**	**0.8 (0.69,0.93)**	0.93 (0.8,1.08)	1.03 (0.89,1.18)	1.05 (0.6,1.86)	1.79 (0.63,5.67)	1.1 (0.83,1.47)	**0.46 (0.4,0.54)**	**0.79 (0.69,0.9)**
**0.78 (0.65,0.94)**	Apixaban 5 mg twice daily	1.16 (0.94,1.43)	**1.35 (1.1,1.65)**	**1.48 (1.21,1.82)**	1.52 (0.85,2.74)	2.59 (0.9,8.26)	**1.59 (1.15,2.19)**	**0.67 (0.54,0.83)**	1.13 (0.93,1.38)
0.91 (0.74,1.12)	1.17 (0.89,1.54)	Dabigatran 110 mg twice daily	1.16 (0.9976,1.36)	**1.28 (1.04,1.58)**	1.32 (0.73,2.37)	2.24 (0.77,7.16)	1.37 (0.99,1.9)	**0.58 (0.47,0.72)**	0.98 (0.8,1.2)
**0.66 (0.53,0.82)**	0.84 (0.63,1.12)	**0.72 (0.57,0.91)**	Dabigatran 150 mg twice daily	1.1 (0.9,1.35)	1.13 (0.63,2.04)	1.93 (0.67,6.15)	1.18 (0.85,1.63)	**0.5 (0.4,0.61)**	0.84 (0.69,1.03)
0.87 (0.74,1.03)	1.12 (0.87,1.43)	0.96 (0.74,1.25)	**1.33 (1.01,1.75)**	Rivaroxaban 20 mg daily	1.03 (0.57,1.85)	1.75 (0.6,5.56)	1.07 (0.78,1.48)	**0.45 (0.37,0.56)**	**0.77 (0.63,0.93)**
**1.87 (1.26,2.8)**	**2.4 (1.55,3.72)**	**2.05 (1.32,3.23)**	**2.85 (1.81,4.5)**	**2.14 (1.4,3.31)**	Low Dose Aspirin (≤ 100 mg daily)	1.7 (0.52,6.08)	1.05 (0.55,1.97)	**0.44 (0.24,0.79)**	0.75 (0.42,1.33)
1.35 (0.74,2.47)	1.72 (0.92,3.25)	1.48 (0.78,2.79)	**2.05 (1.08,3.9)**	1.54 (0.83,2.88)	0.72 (0.38,1.35)	Medium Dose Aspirin (> 100 mg and ≤ 300 mg daily)	0.61 (0.19,1.83)	**0.26 (0.08,0.75)**	0.44 (0.14,1.27)
**1.93 (1.42,2.65)**	**2.47 (1.73,3.55)**	**2.12 (1.46,3.08)**	**2.93 (2.01,4.31)**	**2.21 (1.56,3.15)**	1.03 (0.62,1.71)	1.43 (0.73,2.81)	Clopidogrel 75 mg OD & Low dose Aspirin (≤ 100 mg daily)	**0.42 (0.3,0.58)**	**0.71 (0.52,0.98)**
1.14 (0.98,1.32)	**1.45 (1.15,1.84)**	1.25 (0.97,1.61)	**1.73 (1.32,2.26)**	**1.3 (1.04,1.63)**	**0.61 (0.4,0.92)**	0.84 (0.45,1.56)	**0.59 (0.42,0.83)**	Edoxaban 30 mg daily	1.7 (1.45,1.99)
0.87 (0.74,1.01)	1.11 (0.87,1.41)	0.95 (0.73,1.23)	**1.31 (1,1.73)**	0.99 (0.79,1.25)	**0.46 (0.3,0.7)**	0.64 (0.34,1.19)	**0.45 (0.32,0.63)**	**0.76 (0.65,0.89)**	Edoxaban 60 mg daily

mg= milligram; VKA= vitamin K antagonist
* Results between individual treatments, especially newer oral anticoagulants, should be interpreted with caution given the limitations associated with using a fixed-effects model. See Appendix 9 for additional details.

Formatted: Don't adjust space between Latin and Asian text, Don't adjust space between Asian text and numbers

For peer review only - http://bmjopen.bmj.com/site/about/guidelines.xhtml

Figure 4: Icon array illustrating the absolute risks of Stroke or systemic embolism (blue) and major bleeding episodes (red) per 1000 patients treated*

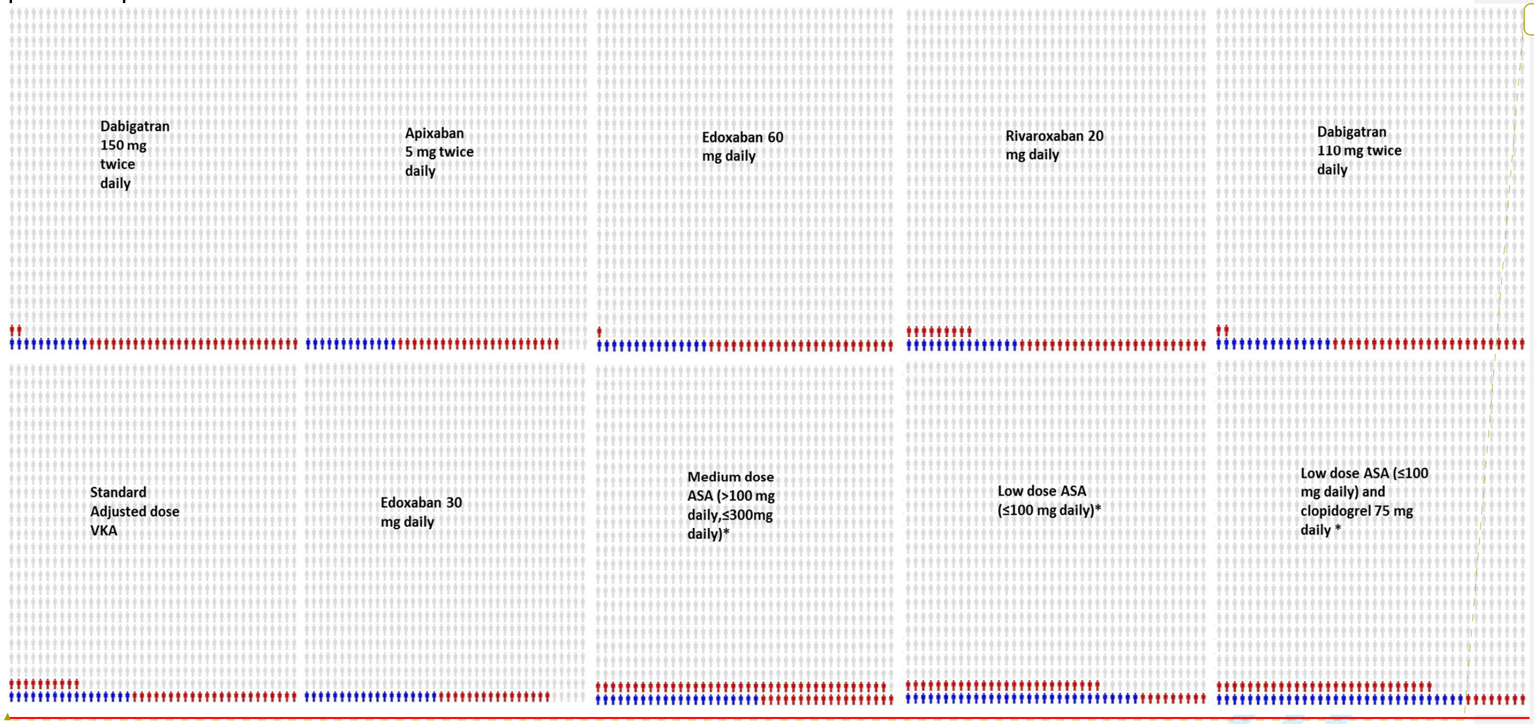

Formatted: Font: (Default) Arial

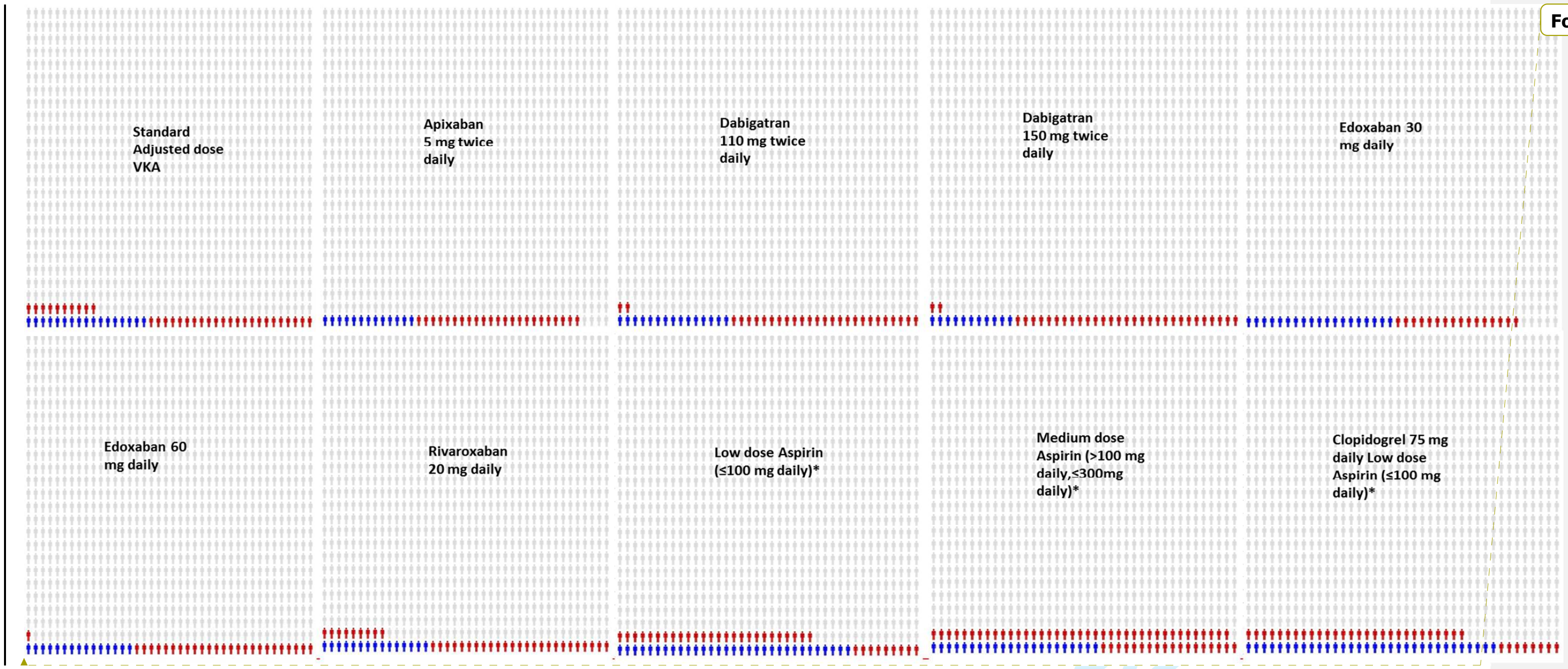

Formatted: Font: (Default) Arial

12

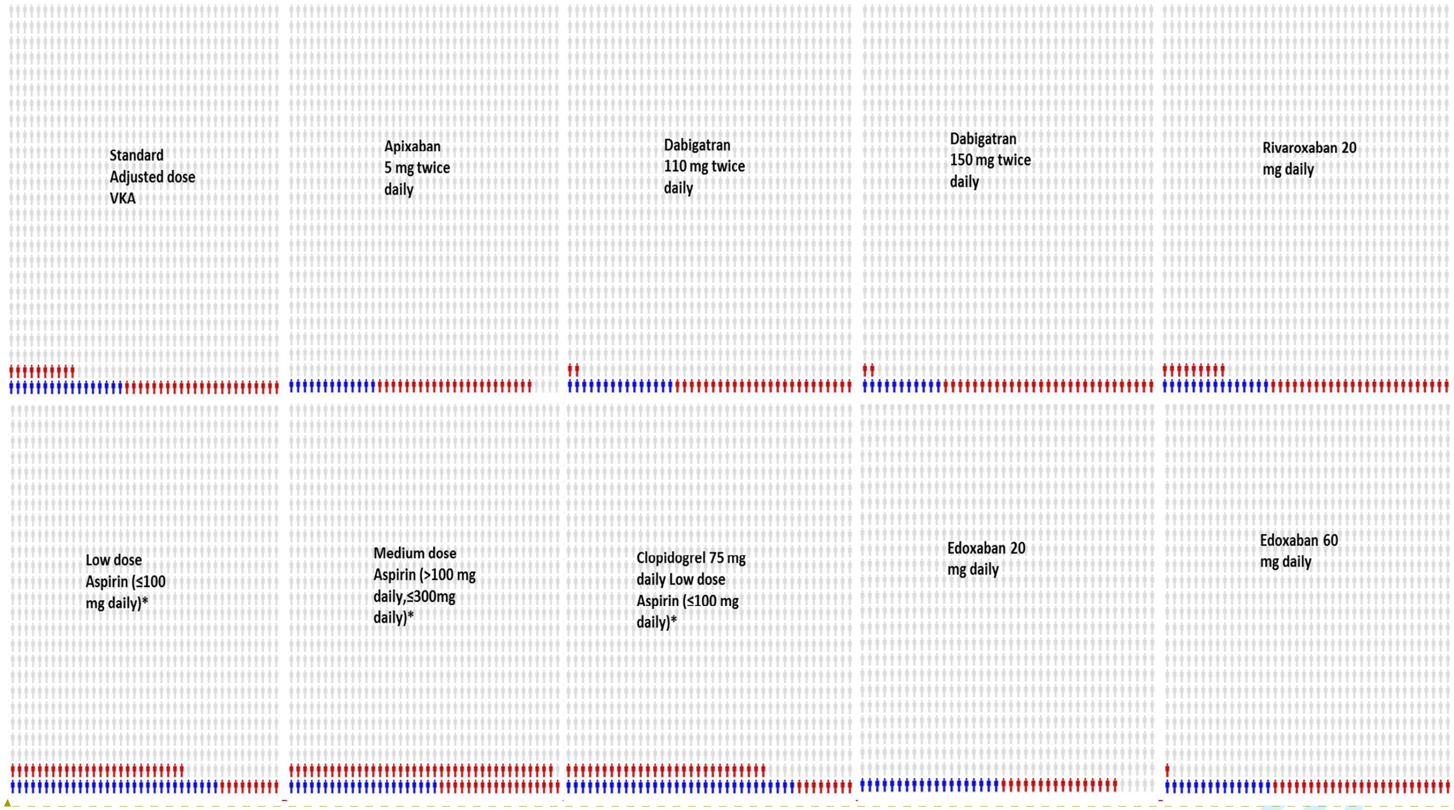

Formatted: Font: (Default) Arial, 11 pt, Bold

* Figures do not reflect uncertainty around effect estimates and there is more uncertainty (i.e., wider credible intervals) for low dose ASA, medium dose ASA, and clopidogrel plus low dose ASA (See Appendix 8). Underlying studies may also double count haemorrhage stroke.

For peer review only - http://bmjopen.bmj.com/site/about/guidelines.xhtml

Acknowledgments: The authors would like to acknowledge Carolyn Spry. She was the Information Specialist from CADTH on the review who conducted the search strategy. We would also like to thank Dr. Robert Giugliano for providing the sub-group data for edoxaban and his thoughtful comments on earlier drafts of the manuscript during the peer review process.

Contributors: CC conceived the study design, performed the data extraction, analysis, and drafted the manuscript. DC and GW conceived the study design, helped with the data analysis, and reviewed the manuscript for important intellectual content. TR conceived the study design, performed the systematic review, data extraction, and reviewed the manuscript for important intellectual content. SK, SS, KG, AB, KM, KC performed the systematic review, data extraction, and reviewed the manuscript for important intellectual content. TC, MC, and KC conceived the study and reviewed the manuscript for important intellectual content. All authors have read and approved the final version of the manuscript. All authors agree to be accountable for all aspects of the work in ensuring that questions related to the accuracy or integrity of any part of the work are appropriately investigated and resolved.

Funding information: This research was supported through a grant by the Canadian Institute of Health Research (CIHR) Drug Safety and Effectiveness Network and financial support from the Canadian Agency for Drugs and Technologies in Health (CADTH). The CIHR Drug Safety and Effectiveness Network played no role in study design, collection, analysis, interpretation of data, writing of the manuscript, or in the decision to submit the paper for publication. They accept no responsibility for the contents. Employees from CADTH were involved in the study design, collection, analysis, interpretation of data, and writing of the manuscript. CADTH, funded by Canadian federal, provincial and territorial governments, is an independent, not-for-profit producer and broker of health technology assessments. Federal, provincial, and territorial health care decision-makers rely on our evidence-based information to make informed decisions about the effectiveness and efficiency of drugs and other health technologies.

Competing interests: All authors have completed the ICMJE uniform disclosure form at www.icmje.org/coi_disclosure.pdf (available on request from the corresponding author) and declare: no support from any organisation for the submitted work; CC is a recipient of a Vanier Canada Graduate Scholarship from the Canadian Institutes of Health Research and has received funding from Canadian Network and Centre for Trials Internationally (CANNeCTIN) and is a trainee on the CIHR Drug Safety and Effectiveness Network team grant. MC is a recipient of a New Investigator Award from the Heart and Stroke Foundation of Canada and holds a T2 research chair in cancer and thrombosis from the University of Ottawa.

Ethical approval: Not required.

Data sharing: The statistical code and dataset used for analyses are reported online: http://www.cadth.ca/media/pdf/TR0003_AntithromboticAgents-AF_ScienceReport-e.pdf. Additional data can be requested by emailing George Wells: gawells@ottawaheart.ca .

The Corresponding Author has the right to grant on behalf of all authors and does grant on behalf of all authors, an exclusive licence (or non exclusive for government employees) on a worldwide basis to the BMJ Publishing Group Ltd to permit this article (if accepted) to be published in BMJ editions and any other BMJPGL products and sublicences such use and exploit all subsidiary rights, as set out in our licence

For peer review only - http://bmjopen.bmj.com/site/about/guidelines.xhtml

REFERENCES

1. Kirchhof, P. *et al.* Atrial fibrillation guidelines across the Atlantic: a comparison of the current recommendations of the European Society of Cardiology/European Heart Rhythm Association/European Association of Cardiothoracic Surgeons, the American College of Cardiology Fou. *Eur. Heart J.* **34,** 1471–1474 (2013).

2. Skanes, A. C. *et al.* Focused 2012 update of the Canadian Cardiovascular Society atrial fibrillation guidelines: recommendations for stroke prevention and rate/rhythm control. *Can. J. Cardiol.* **28,** 125–36 (2012).

3. *Antithrombotic agents for the prevention of stroke and systemic embolism in patients with atrial fibrillation*. (2013). at <http://www.cadth.ca/media/pdf/TR0003_AntithromboticAgents-AF_ScienceReport-e.pdf>

4. Schulman, S. & Kearon, C. Definition of major bleeding in clinical investigations of antihemostatic medicinal products in non-surgical patients. *J. Thromb. Haemost.* **3,** 692–4 (2005).

5. NHS Quality Improvement Scotland (NHS QIS). *SIGN 50. A guideline developer's handbook*. (2008). at <http://www.sign.ac.uk/pdf/sign50.pdf>

6. Moher, D., Liberati, A., Tetzlaff, J. & Altman, D. G. Preferred reporting items for systematic reviews and meta-analyses: the PRISMA statement. *PLoS Med.* **6,** e1000097 (2009).

7. Dias, S., Welton, N. J., Sutton, A. J. & Ades, A. *NICE DSU Technical Support Document 2: A Generalised Linear Modelling Framework for Pairwise and Network Meta-Analysis of Randomised Controlled Trials*. *Trop. Med.* 1–96 (2011). at <www.nicedsu.org.uk>

8. Connolly, S. & Ezekowitz, M. Dabigatran versus warfarin in patients with atrial fibrillation. *N. Engl. J. Med.* **361,** 1139–1151 (2009).

9. Ingelheim, B. *Advisory committee briefing document: dabigatran etexilate [Internet]*. 1–168 (2010). at <http://www.fda.gov/downloads/advisorycommittees/committeesmeetingmaterials/drugs/cardiovas>

10. Cipriani, A. *et al.* Comparative efficacy and acceptability of 12 new-generation antidepressants: a multiple-treatments meta-analysis. *Lancet* **373,** 746–58 (2009).

For peer review only

11. Turner, R. M., Davey, J., Clarke, M. J., Thompson, S. G. & Higgins, J. P. Predicting the extent of heterogeneity in meta-analysis, using empirical data from the Cochrane Database of Systematic Reviews. *Int. J. Epidemiol.* **41,** 818–27 (2012).

12. Spiegelhalter, D. J., Best, N. G., Carlin, B. P. & van der Linde, A. Bayesian measures of model complexity and fit. *J. R. Stat. Soc. Ser. B (Statistical Methodol.* **64,** 583–639 (2002).

13. Spiegelhalter, D., Thomas, A. & Best, N. WinBUGS user manual. *MRC Biostat. Unit* **2,** (2004).

14. meta: Meta-Analysis with R. at <http://cran.r-project.org/web/packages/meta/index.html>

15. Dias, S., Welton, N.J., Sutton, A.J., Caldwell, D.M., Guobing, L. & Ades, A. E. *NICE Technical Support Document 4: Inconsistency in Networks of Evidence Based on Randomised Controlled Trials*. 1–39 (2011). at <www.nicedsu.org.uk>

16. Salanti, G., Marinho, V. & Higgins, J. P. T. A case study of multiple-treatments meta-analysis demonstrates that covariates should be considered. *J. Clin. Epidemiol.* **62,** 857–64 (2009).

17. Wells, G., Coyle, D., Cameron, C., Steiner, S. & Kelly, S. *Safety, effectiveness, and costeffectiveness of new oral anticoagulants compared with warfarin in preventing stroke and other cardiovascular events in patients with atrial fibrillation*. (2012). at <http://www.cadth.ca/media/pdf/NOAC_Therapeutic_Review_final_report.pdf>

18. Johnson & Johnson Pharmaceutical Research & Development. *Rivaroxaban for the Prevention of Stroke and Non-Central Nervous System (CNS) Systemic Embolism in Patients With Atrial Fibrillation*. **7939,** 222 (2011).

19. McDonagh, M. S., Peterson, K., Balshem, H. & Helfand, M. US Food and Drug Administration documents can provide unpublished evidence relevant to systematic reviews. *J. Clin. Epidemiol.* (2013). doi:10.1016/j.jclinepi.2013.05.006

20. Giugliano, R. P. *et al.* Edoxaban versus warfarin in patients with atrial fibrillation. *N. Engl. J. Med.* **369,** 2093–104 (2013).

21. Granger, C. & Alexander, J. Apixaban versus warfarin in patients with atrial fibrillation. *N. Engl. J. Med.* 981–992 (2011).

22. Patel, M., Mahaffey, K. & Garg, J. Rivaroxaban versus warfarin in nonvalvular atrial fibrillation. *N. Engl. J. Med.* 883–891 (2011).

23. Connolly, S. *et al.* Clopidogrel plus aspirin versus oral anticoagulation for atrial fibrillation in the Atrial fibrillation Clopidogrel Trial with Irbesartan for prevention of

For peer review only

Vascular Events (ACTIVE W): a randomised controlled trial. *Lancet* **367,** 1903–12 (2006).

24. Ezekowitz, M. D. *et al.* Dabigatran with or without concomitant aspirin compared with warfarin alone in patients with nonvalvular atrial fibrillation (PETRO Study). *Am. J. Cardiol.* **100,** 1419–26 (2007).

25. Rash, A. *et al.* A randomised controlled trial of warfarin versus aspirin for stroke prevention in octogenarians with atrial fibrillation (WASPO). *Age Ageing* **36,** 151–6 (2007).

26. Chung, N. *et al.* Safety of edoxaban, an oral factor Xa inhibitor, in Asian patients with non-valvular atrial fibrillation. *Thromb. Haemost.* **105,** 535–44 (2011).

27. Yamashita, T. *et al.* Randomized, Multicenter, Warfarin-Controlled Phase II Study of Edoxaban in Japanese Patients With Non-Valvular Atrial Fibrillation. *Circ. J.* **76,** 1840–1847 (2012).

28. Connolly, S. & Eikelboom, J. Apixaban in patients with atrial fibrillation. *N. Engl. J. Med.* **364,** 806–817 (2011).

29. Connolly, S. J. *et al.* Effect of clopidogrel added to aspirin in patients with atrial fibrillation. *N. Engl. J. Med.* **360,** 2066–78 (2009).

30. Harenberg, J. & Weiss, C. Clinical trials with new oral anticoagulants. Additive value of indirect comparisons also named network meta-analyses. *Hamostaseologie* **33,** 62–70 (2013).

31. Dogliotti, A., Paolasso, E. & Giugliano, R. P. Current and new oral antithrombotics in non-valvular atrial fibrillation: a network meta-analysis of 79 808 patients. *Heart* (2013). doi:10.1136/heartjnl-2013-304347

32. Ruff, C. T. *et al.* Comparison of the efficacy and safety of new oral anticoagulants with warfarin in patients with atrial fi brillation□: a meta-analysis of randomised trials. *Lancet* **6736,** 1–8 (2013).

33. Assiri, A., Al-Majzoub, O., Kanaan, A. O., Donovan, J. L. & Silva, M. Mixed treatment comparison meta-analysis of aspirin, warfarin, and new anticoagulants for stroke prevention in patients with nonvalvular atrial fibrillation. *Clin. Ther.* **35,** 967–984.e2 (2013).

34. Slot, K. & Berge, E. Factor Xa inhibitors versus vitamin K antagonists for preventing cerebral or systemic embolism in patients with atrial fibrillation. *Stroke* (2013). at <http://stroke.ahajournals.org/content/44/12/e165.short>

For peer review only

35. Salanti, Georgia, Kavvoura, F, Ioannidis, J. Exploring the geometry of treatment networks. *Ann. Intern. Med.* **148,** 544–553 (2008).

36. Barratt, A. *et al.* Tips for learners of evidence-based medicine: 1. Relative risk reduction, absolute risk reduction and number needed to treat. *Can. Med. Assoc. J.* **171,** 353–8 (2004).

37. Schechtman, E. Odds ratio, relative risk, absolute risk reduction, and the number needed to treat--which of these should we use? *Value Health* **5,** 431–6 (2002).

38. Berger, M. L., Mamdani, M., Atkins, D. & Johnson, M. L. Good research practices for comparative effectiveness research: defining, reporting and interpreting nonrandomized studies of treatment effects using secondary data sources: the ISPOR Good Research Practices for Retrospective Database Analysis Task Force . *Value Health* **12,** 1044–52 (2009).

39. Berger, M. L. *et al.* Prospective observational studies to assess comparative effectiveness: the ISPOR good research practices task force report. *Value Health* **15,** 217–30 (2012).

40. Jansen, J. P. Network meta-analysis of individual and aggregate level data. *Res. Synth. Methods* **3,** 177–190 (2012).

41. Salanti, G., Ades, A. & Ioannidis, J. Graphical methods and numerical summaries for presenting results from multiple-treatment meta-analysis: an overview and tutorial. *J. Clin. Epidemiol.* **64,** 163–71 (2011).

42. Guo, J. J. *et al.* A review of quantitative risk-benefit methodologies for assessing drug safety and efficacy-report of the ISPOR risk-benefit management working group. *Value Health* **13,** 657–66 (2010).

43. Boyd, C. *et al. Methods Research Report Methods for Benefit and Harm Assessment in Systematic Reviews*. 52 (2012). at <www.ncbi.nlm.nih.gov/books/NBK115750/>

44. Thorlund, K., Thabane, L. & Mills, E. J. Modelling heterogeneity variances in multiple treatment comparison meta-analysis – Are informative priors the better solution□? *BMC Med. Res. Methodol.* **13,** 1 (2013).

For peer review only

SUPPLEMENTARY APPENDIX

Appendix 1: Search Strategy

OVERVIEW	
Interface:	Ovid
Databases:	EBM Reviews - Cochrane Central Register of Controlled Trials <May 2012><br>Ovid Embase 1974 to June 6, 2012<br>Ovid MEDLINE 1946 to June 7, 2012<br>Ovid MEDLINE Daily<br>Ovid MEDLINE In-Process & Other Non-Indexed Citations<br>**Note:** Subject headings have been customized for each database. Duplicates between databases were removed in Ovid.
Date of Search:	June 7, 2012; updated January 23, 2014
Alerts:	Monthly search updates began June 11, 2012 and ran until project completion
Study Types:	Randomized Controlled Trials
Limits:	Publication years 1988-present<br>Humans<br>Conference abstracts omitted

SYNTAX GUIDE	
/	At the end of a phrase, searches the phrase as a subject heading
.sh	At the end of a phrase, searches the phrase as a subject heading
MeSH	Medical Subject Heading
exp	Explode a subject heading
*	Before a word, indicates that the marked subject heading is a primary topic;<br>or, after a word, a truncation symbol (wildcard) to retrieve plurals or varying endings
#	Truncation symbol for one character
ADJ	Requires words are adjacent to each other (in any order)
ADJ#	Adjacency within # number of words (in any order)
.ti	Title
.ab	Abstract
.hw	Heading Word; usually includes subject headings and controlled vocabulary
.nm.	Name of substance word
.ot	Original title
.pt	Publication type
.rn	CAS registry number
use cctr	Select results from Cochrane database of Clinical Trials
use oemezd	Select results from Embase database
use pmez	Select results from Medline database

Clinical Multi-database Strategy

Line #	Search Strategy
1	(211914-51-1 or 211915-06-9).rn,nm. or (dabigatran* or Pradaxa or Pradax or Prazaxa or Praxada or

For peer review only

	bibr 1048 or bibr1048 or bibr953 or bibr 953 or rendix).ti,ot,ab,sh,rn,hw,nm.
2	(BMS 562247 or BMS562247 or apixaban* or Eliquis or Eliques).ti,ot,ab,sh,rn,hw,nm. or 503612-47-3.rn,nm.
3	(rivaroxaban* or Xarelto or BAY 59-7939 or BAY59-7939 or BAY597939 or BAY 597939).ti,ot,ab,sh,rn,hw,nm. or 366789-02-8.rn,nm.
4	(90055-48-4 or 113665-84-2).rn,nm. or (clopidogrel* or clopilet or grepid or iscover or plavix or zopya or zylagren or zyllt or duocover or duoplavin).ti,ot,ab,sh,rn,hw,nm.
5	Aspirin/ or 50-78-2.rn,nm. or (acetylsalicylic acid* or ASA* or acenterine or acesal or acetan or acetard or aceticil or aceticyl or acetilum or acetonyl or acetilum or acetonyl or acetophen or acetosal or acetosalicylic acid or acetosalin or acetosalum or acetyl salicylate or acetyl salicylic acid or acetylin or acetylo or acetylon or acetylosalicylic acid or acetylsal or acetysal or acetylsalicyclic acid or acetylsalicyl or acetylsalicylate or acetylsalycic acid or acetylsalycylic acid or oracidulatum or actorin or Acylpyrin or acylpyrine or acytosal or adiro or alabunkun or alasil or albyl-e or alka seltzer or alkASA or Aloxiprimum or anacin or anasprin or anopyrin or ansin or anthrom or aptor or arthralgyl or asaflow or ascription or orasaphen or asapor or asatard or asawin or aspec or aspent or aspergum or aspex or aspilets or aspirem or aspirgran or ASAa or ASAe or ASAine or aspirisucre or aspisol or aspo cid or aspro or asrina or asrivo or asta or asteric or astrix or bebesan or biprin or bokey or boxazin or breoprin or bufferin or cafenol or caprin or cASA or catalgine or catalgix or cemerit or cemirit or claradin or claragine or Colfarit or comoprin or contrheuma or daga or darosal or Dispril or dispirin or dolean or dusil or Easprin or ecasil or Ecotrin or egalgic or emocin or empirin or encaprin or encine em or Endosprin or entaprin or entercin or enteroprin or enterosarine or enterospirine or entrophen or eskotrin or euthermine or extren or genasprin or globentyl or godamed or gotosan or helicon or idotyl or infatabs a or istopirin or istopyrine or ivepirine or juvepirine or keypo or kilios or kinderASA or Magnecyl or measurin or mejoral or melabon or Micristin or micropyrin or mikristin or miniasal or mycrisitin or naspro or novasen or nu seal or nu seals or ortho acetoxybenzoate or ortho acetoxybenzoic acid or ortho acetyloxybenzoate or ortho acetyloxybenzoic acid or ostoprin or pancemol or paracin or paynocil or pengo or plewin or Polopirin or polopiryna or premaspin or primaspan or proprin or pyronoval or reumyl or rhodine or rhonal or ronal or salacetin or salisalido or sargepirine or soldral or solpyron or Solprin or solucetyl or solupsa or Solupsan or spren or super tru or tapal or temagin or tevapirin or th 2152 or thrombo-aspilets or treupahlin or treuphalin or tromalyt or tromcor or turiital or verin or vitalink or xaxa or Zorprin).ti,ot,ab,sh,rn,hw,nm.
6	Warfarin/ or 81-81-2.rn,nm. or (adoisine or aldocumar or athrombin* or befarin or brumolin or carfin or circuvit or compound 42 or coumadan or coumadin or coumadine or coumafen or coumafene or coumefene or coumaphene or coumarins or dagonal or farin or jantoven or kumatox or maforan or marevan or orfarin or panwarfarin or panwarfin or prothromadin or simarc 2 or sofarin or tedicumar or tintorane or waran or warfant or warfar or warfarin or warfarine or warfil or warfilone or warnerin).ti,ot,ab,sh,rn,hw,nm.
7	Acenocoumarol/ or 152-72-7.rn,nm. or (acenocoumarol* or nicoumalone or acenocoumarin or acenocoumarine or acenocoumarole or acenocoumarolum or acenocumarol or acenocumarolo or acenocumerol or acenkumarin or acitrom or ascumar or neo sintrom or neosintrom or neositron or nicoumalone or nicumalon or nitrovarfarian or nitrowarfarin or sincoumar or sincumar or sinkumar or sinthrom or sinthrome or sintrom or sintroma or sintron or syncoumar or syncumar or synthrom or syntrom or syntroma or sintron or syncoumar or syncumar or syntrom or trombostop or zotil or sinthrome or synthrom or syncoumar or syncumar or sinkumar or sintrom or minisintrom or trombostop or zotil).ti,ot,ab,sh,rn,hw,nm.
8	or/1-7
9	Atrial fibrillation/
10	((atrial or atrium or auricular or heart) adj1 fibrillation*).ti,ab.
11	(a-fib or afib).ti,ab.
12	9 or 10 or 11
13	8 and 12
14	13 use pmez
15	13 use cctr
16	14 or 15
17	*dabigatran/ or (dabigatran* or Pradaxa or Pradax or Prazaxa or praxada or bibr 1048 or bibr1048 or bibr953 or bibr 953 or rendix).ti,ab.
18	*apixaban/ or (BMS 562247 or BMS562247 or apixaban* or Eliquis or Eliques).ti,ab.
19	*rivaroxaban/ or (rivaroxaban* or Xarelto or BAY 59-7939 or BAY59-7939 or BAY597939 or BAY 597939).ti,ab.

For peer review only - http://bmjopen.bmj.com/site/about/guidelines.xhtml

20	*clopidogrel/ or *acetylsalicylic acid plus clopidogrel/ or (clopidogrel* or clopilet or grepid or iscover or plavix or zopya or zylagren or zyllt or duocover or duoplavin).ti,ab.
21	*warfarin/ or (adoisine or aldocumar or athrombin* or befarin or brumolin or carfin or circuvit or compound 42 or coumadan or coumadin or coumadine or coumafen or coumafene or coumefene or coumaphene or coumarins or dagonal or farin or jantoven or kumatox or maforan or marevan or orfarin or panwarfarin or panwarfin or prothromadin or simarc 2 or sofarin or tedicumar or tintorane or waran or warfant or warfar or warfarin* or warfarine or warfil or warfilone or warnerin).ti,ab.
22	*acetylsalicylic acid/ or (acetylsalicylic acid* or ASA* or acenterine or acesal or acetan or acetard or aceticil or aceticyl or acetilum or acetonyl or acetilum or acetonyl or acetophen or acetosal or acetosalicylic acid or acetosalin or acetosalum or acetyl salicylate or acetyl salicylic acid or acetylin or acetylo or acetylon or acetylosalicylic acid or acetylsal or acetysal or acetylsalicyclic acid or acetylsalicyl or acetylsalicylate or acetylsalycic acid or acetylsalycylic acid or oracidulatum or actorin or Acylpyrin or acylpyrine or acytosal or adiro or alabunkun or alasil or albyl-e or alka seltzer or alkASA or Aloxiprimum or anasprin or anopyrin or ansin or anthrom or aptor or arthralgyl or asaflow or orasaphen or asapor or asatard or asawin or aspec or aspent or aspergum or aspex or aspilets or aspirem or aspirgran or ASAa or ASAe or ASAine or aspirisucre or aspisol or aspo cid or aspro or asrina or asrivo or asta or asteric or astrix or bebesan or biprin or bokey or boxazin or breoprin or bufferin or cafenol or caprin or cASA or catalgine or catalgix or cemerit or cemirit or claradin or claragine or Colfarit or comoprin or contrheuma or daga or darosal or Dispril or dispirin or dolean or dusil or Easprin or ecasil or Ecotrin or egalgic or emocin or empirin or encaprin or encine em or Endosprin or entaprin or entercin or enteroprin or enterosarine or enterospirine or entrophen or eskotrin or euthermine or extren or genasprin or globentyl or godamed or gotosan or helicon or idotyl or infatabs a or istopirin or istopyrine or ivepirine or juvepirine or keypo or kilios or kinderASA or Magnecyl or measurin or mejoral or melabon or Micristin or micropyrin or mikristin or miniasal or mycrisitin or naspro or novasen or nu seal or nu seals or ortho acetoxybenzoate or ortho acetoxybenzoic acid or ortho acetyloxybenzoate or ortho acetyloxybenzoic acid or ostoprin or pancemol or paracin or paynocil or pengo or plewin or Polopirin or polopiryna or premaspin or primaspan or proprin or pyronoval or reumyl or rhodine or rhonal or ronal or salacetin or salisalido or sargepirine or soldral or solpyron or Solprin or solucetyl or solupsa or Solupsan or spren or super tru or tapal or temagin or tevapirin or th 2152 or thrombo-aspilets or treupahlin or treuphalin or tromalyt or tromcor or turiital or verin or vitalink or xaxa or Zorprin).ti,ab.
23	*Acenocoumarol/ or (acenocoumarol* or nicoumalone or acenocoumarin or acenocoumarine or acenocoumarole or acenocoumarolum or acenocumarol or acenocumarolo or acenocumerol or acenkumarin or acitrom or ascumar or neo sintrom or neosintrom or neositron or nicoumalone or nicumalon or nitrovarfarian or nitrowarfarin or sincoumar or sincumar or sinkumar or sinthrom or sinthrome or sintrom or sintroma or sintron or syncoumar or syncumar or synthrom or syntrom or syntroma or sintron or syncoumar or syncumar or syntrom or trombostop or zotil or sinthrome or synthrom or syncoumar or syncumar or sinkumar or sintrom or minisintrom or trombostop or zotil).ti,ab.
24	or/17-23
25	Heart atrium fibrillation/
26	((atrial or atrium or auricular or heart) adj1 fibrillation*).ti,ab.
27	(a-fib or afib).ti,ab.
28	25 or 26 or 27
29	24 and 28
30	29 use oemezd
31	30 not conference abstract.pt.
32	16 or 31
33	32
34	limit 33 to english language [Limit not valid in CCTR; records were retained]
35	(Randomized Controlled Trial or Controlled Clinical Trial).pt.
36	Randomized Controlled Trial/
37	Randomized Controlled Trials as Topic/
38	"Randomized Controlled Trial (topic)"/
39	Controlled Clinical Trial/
40	Controlled Clinical Trials as Topic/
41	"Controlled Clinical Trial (topic)"/
42	Randomization/

For peer review only

43	Random Allocation/
44	Double-Blind Method/
45	Double Blind Procedure/
46	Double-Blind Studies/
47	Single-Blind Method/
48	Single Blind Procedure/
49	Single-Blind Studies/
50	Placebos/
51	Placebo/
52	Control Groups/
53	Control Group/
54	(random* or sham or placebo*).ti,ab,hw.
55	((singl* or doubl*) adj (blind* or dumm* or mask*)).ti,ab,hw.
56	((tripl* or trebl*) adj (blind* or dumm* or mask*)).ti,ab,hw.
57	(control* adj3 (study or studies or trial*)).ti,ab.
58	(Nonrandom* or non random* or non-random* or quasi-random* or quasirandom*).ti,ab,hw.
59	allocated.ti,ab,hw.
60	((open label or open-label) adj5 (study or studies or trial*)).ti,ab,hw.
61	or/35-60
62	34 and 61
63	limit 62 to yr = "1988 -Current"
64	remove duplicates from 63

**OTHER DATABASES**	
PubMed	Same MeSH, keywords, limits, and study types used as per MEDLINE search, with appropriate syntax used.

Grey Literature

Dates for Search:	**June 12, 2012 – July 8, 2012; updated January 2014**
**Keywords:**	dabigatran, Pradax, Pradaxa, apixaban, Eliquis, rivaroxaban, Xarelto, clopidogrel, Plavix, acetylsalicylic acid, Aspirin, warfarin, Coumadin, acenocoumarol, Sintrom, Sinthrome<br>Atrial fibrillation
**Limits:**	Publication years 1988-present, English

Relevant items from the following sections of the CADTH grey literature checklist, “Grey matters: a practical tool for evidence-based searching” (http://www.cadth.ca/resources/grey-matters) were searched:

- Health Technology Assessment Agencies
- Health Economic
- Clinical Practice Guidelines
- Databases (free)

Internet Search

For peer review only - http://bmjopen.bmj.com/site/about/guidelines.xhtml

Appendix 2: PRISMA Diagram

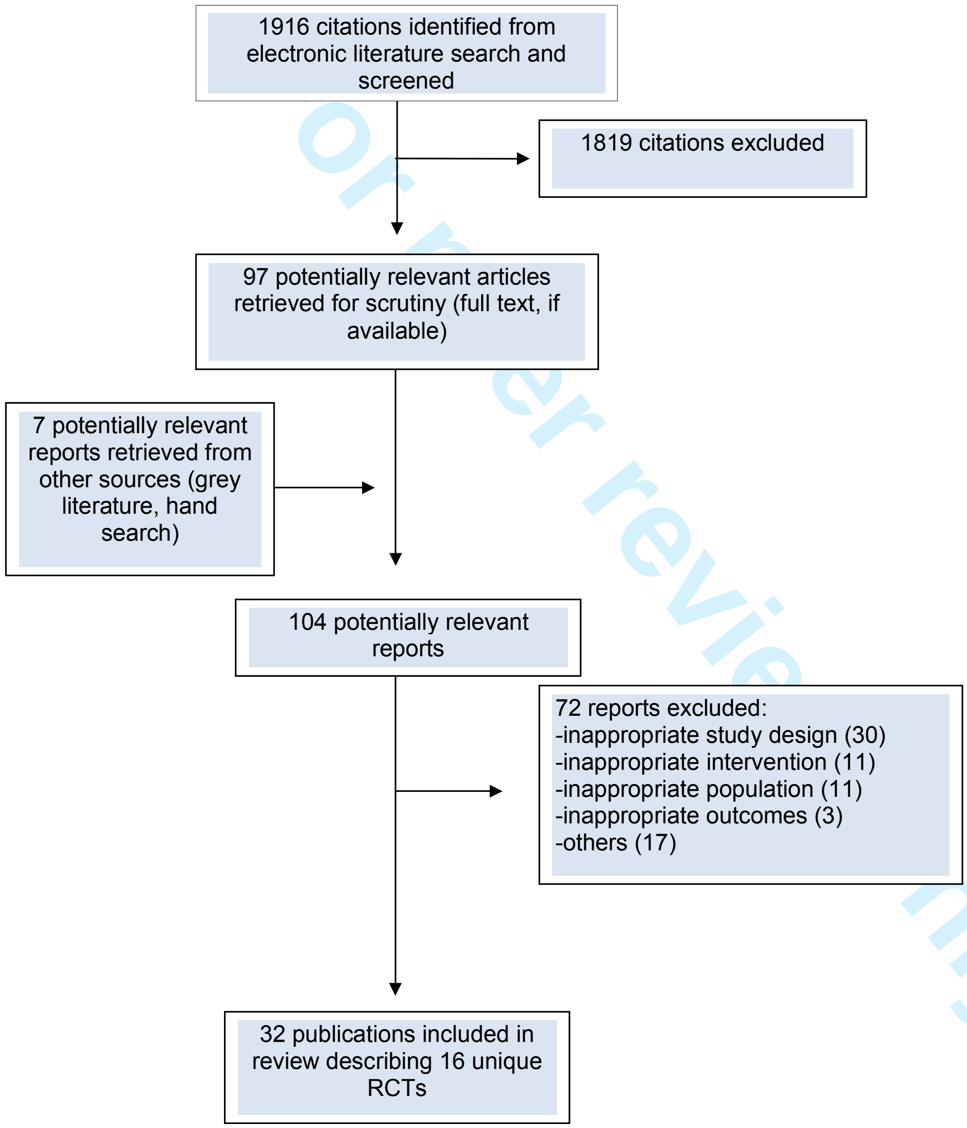

For peer review only - http://bmjopen.bmj.com/site/about/guidelines.xhtml

Appendix 3: Summary of Patient Baseline Characteristics

Study	Baseline Characteristics: Age, Mean ± SD (y)	Age Category (%)		Sex (%)		$CHADS_2$, Mean ± SD	$CHADS_2$ Category (%)			Prior Stroke /TIA (%)	DM (%)	Heart Failure (%)	HBP (%)	MI (%)	VKA Naïve (%)	Centre above TTR Throughout follow-up: Mean TTR (%)	TTR Category (%)	
		≤75y	>75 y	M	F		0	1	≥2								≤66%	>66%
ACTIVE-W n=6706	70 ± NR	NR	NR	66	34	2 ± 1.1	NR	NR	NR	15	21	30	82	17	23	64	48	52
AFASAK n=672	75 ± NR	NR	NR	54	46	NR ± NR	NR	NR	NR	6	9	53	32	7	NR	N/A	N/A	N/A
AFASAK-2 n=339	73 ± NR	NR	NR	61	39	NR ± NR	NR	NR	NR	8	12	70	45	7	NR	NR	NR	NR
ARISTOTLE n=18201	70 ± NR	69	31	65	35	2.1 ± 1.1	0	34	66	19	25	35	87	14	43	62	NR	NR
ARISTOTLE-J n=222	70 ± NR	69	31	83	17	1.9 ± NR	1	42	57	28	23	1	83	NR	16	60	NR	NR
BAFTA n=973	82 ± 4	0	100	55	45	NR ± NR	NR	NR	NR	13	13	20	54	11	NR	67	NR	NR
CAFA n=378	68 ± NR	NR	NR	75	25	NR ± NR	NR	NR	NR	4	12	22	39	13	NR	44	NR	NR
Chung et al 2011 n=234	65 ± 9	87	13	65	35	1.9 ± 1.1	0	47	53	24	30	29	71	NR	48	45	NR	NR
ENGAGE AF-TIMI 48 n=21105	72 ± NR	60	40	62	38	2.8 ± 1.0	0	0	100	28	36	57	94	12	41	65	52	48
JAST n=871	65 ± NR	NR	NR	70	30	NR ± NR	NR	NR	NR	3	14	9	39	NR	92	N/A	N/A	N/A
PETRO n=236	70 ± NR	70	30	82	18	NR ± NR	NR	NR	NR	18	25	32	71	NR	0	57	NR	NR
RE-LY n=18113	71 ± NR	60	40	64	36	2.1 ± NR	32		68	20	20	23	32	79	17	64	50	50
ROCKET-AF n=14264	73 ± NR	NR	NR	60	40	3.5 ± NR	0	0	100	55	40	62	91	17	38	55	NR	NR
WASPO n=75	83 ± NR	0	100	47	53	NR ± NR	NR	NR	NR	NR	4	NR	47	NR	NR	69	NR	NR
Weitz et al 2010 n=719	65 ± 9	NR	NR	62	38	NR ± NR	0	0	100	NR	NR	NR	NR	NR	66	50	NR	NR

For peer review only - http://bmjopen.bmj.com/site/about/guidelines.xhtml

Study	Baseline Characteristics																Centre above TTR Throughout follow-up		
	Age, Mean ± SD (y)	Age Category (%)		Sex (%)		CHADS₂, Mean ± SD	CHADS₂ Category (%)			Prior Stroke /TIA (%)	DM (%)	Heart Failure (%)	HBP (%)	MI (%)	VKA Naïve (%)		Mean TTR (%)	TTR Category (%)	
		≤75y	>75 y	M	F		0	1	≥2									≤66%	>66%
Yamashita et al 2012 n=385	69 ± NR	71	29	83	17	2.1 ± NR	0	100		28	23	27	73	NR	15		73% FOR <70Y; 83% FOR ≥70Y;	NR	NR

DM = diabetes mellitus; F = female sex; HBP = high blood pressure; M = male sex; MI = myocardial infarction; N/A = not applicable; NR = not reported; SD = standard deviation; TIA = transient ischemic attack; TTR = time in therapeutic range; VKA = vitamin K antagonist; y = year.

Note: Only data from the included relevant treatment arms have been extracted.

For peer review only - http://bmjopen.bmj.com/site/about/guidelines.xhtml

Appendix 4: Summary of Included Trials

Study and Design	Disposition	Population	Interventions	Follow-up	Primary Outcome(s)
**ACTIVE-W**<br>OL RCT, 2006.<br>Multicenter, 32 countries (including Europe / America).	Randomized: n=6706	AF patients eligible / willing to take anticoagulant therapy, with ≥ 1 pre-specified risk factor for stroke.	**Clopidogrel / Low-dose ASA** (n=3335)<br>**Warfarin – INR 2.0 to 3.0** (n=3371)	1.3 years (stopped early)	First occurrence of stroke, non-CNS SE, MI or vascular death.
**AFASAK**<br>OL/DB RCT, 1989.<br>Multicenter, Copenhagen.	Randomized: n=1007	AF patients, with no recent anticoagulation or cerebrovascular event, valve replacement or rheumatic heart disease.	***OL Warfarin – INR 2.8 to 4.2 **** *(n=335)*<br>**Low-dose ASA** (n=336)<br>**PL** (n=336)	2 years	Thromboembolic complication: stroke, TIA or embolic complication to viscera / extremities.
**AFASAK-2**<br>OL RCT, 1998.<br>Multicenter, Copenhagen.	Randomized: n=677	AF patients ≥ 60 years (unless with CV or pulmonary disease), without recent TIA or risk factors for bleeding.	***Fixed-dose Warfarin **** *(n=167)*<br>***Fixed-dose Warfarin / Medium-dose ASA **** *(n=171)*<br>**Medium Dose ASA** (n=169)<br>**Warfarin – INR 2.0 to 3.0** (n=170)	3.5 years	Stroke or SE.
**ARISTOTLE**<br>DB RCT, 2011.<br>Multicenter, 39 countries (including Europe / America).	Randomized: n=18201	AF patients with ≥ 1 risk factor for stroke, without recent stroke or ASA > 165 mg.	**Apixaban 5 mg BID** (n=9120)<br>**Warfarin – INR 2.0 to 3.0** (n=9081)<br>Concomitant use of **ASA** (≤ 165 mg daily) or **NSAIDs** permitted.	NR	Stroke or SE;<br>Major bleeding.
**ARISTOTLE-J**<br>OL/DB RCT, 2011.<br>Multicenter, Japan.	Randomized: n=222	AF patients with ≥ 1 risk factor for stroke, without recent TIA or ASA > 100 mg.	**Apixaban 2.5 mg BID** (n=74)<br>**Apixaban 5 mg BID** (n=74)<br>**OL Warfarin – INR 2.0 to 3.0** (n=74)<br>Concomitant use of **ASA** (≤ 100 mg daily) permitted.	12 weeks	Composite of major bleeding and CRNM bleeding events.
**BAFTA**<br>OL RCT, 2007.<br>Multicenter, UK.	Randomized: n=973	AF patients ≥ 75 years, with clinical uncertainty around optimal treatment.	**Low-dose ASA** (n=485)<br>**Warfarin – INR 2.0 to 3.0** (n=488)	2.7 years	Disabling stroke, ICH and other clinically significant arterial embolism.
**CAFA**<br>DB RCT, 1991.<br>Multicenter, Canada.	Randomized: n=378	AF patients without recent stroke, TIA or MI, and who did not require antiplatelet drug therapy.	**Warfarin – INR 2.0 to 3.0** (n=187)<br>**PL** (n=191)	1.2 years (stopped early)	Ischemic stroke, SE, ICH or fatal hemorrhage.

For peer review only - http://bmjopen.bmj.com/site/about/guidelines.xhtml

Study and Design	Disposition	Population	Interventions	Follow-up	Primary Outcome(s)
**Chung et al 2011**<br>OL RCT, 2011.<br>Multicenter, 4 countries (including Asia).	Randomized: n=234	AF patients at moderate to high risk of stroke (CHADS2 score ≥ 1).	**Edoxaban 30 mg OD** (n=79)<br>**Edoxaban 60 mg OD** (n=80)<br>**Warfarin – INR 2.0 to 3.0** (n=75)	12 weeks	All bleeding events
**ENGAGE AF-TIMI 48**<br>DB RCT, 2013.<br>Multicenter, 46 countries (including Europe / America).	Randomized: n=21105	AF patients at moderate to high risk of stroke (CHADS2 score ≥ 2).	**Edoxaban 30 mg OD** (n=7034)<br>**Edoxaban 60 mg OD** (n=7035)<br>**Warfarin – INR 2.0 to 3.0** (n=7036)<br>Almost a third of patients in treatment arms took concomitant ASA.	2.8 years	Stroke or SE and major haemorrhage.
**JAST**<br>OL RCT, 2006.<br>Multicenter, Japan.	Randomized: n=871	AF patients without recent symptomatic thromboembolic disease, ICH or GI bleeding.	**Medium-dose ASA** (n=426)<br>**No treatment** (n=445)	2.1 years (stopped early)	CV death, symptomatic brain infarction, or TIA.
**PETRO**<br>OL/DB RCT, 2007.<br>Multicenter, 4 countries (Europe and USA).	Randomized: n=502	AF patients with ≥ 1 risk factor for stroke and with coronary artery disease (removed halfway to facilitate recruitment). No recent MI, stroke, TIA or major bleed. Warfarin pre-treatment required for ≥ 8 weeks.	***Dabigatran 50 mg BID ± Low-dose or High-dose ASA*** ** (n=105)*<br>***Dabigatran 300 mg BID ± Low-dose or High-dose ASA*** ** (n=161)*<br>**Dabigatran 150 mg BID ± Low-dose or High-dose ASA** † (n=166)<br>**OL Warfarin – INR 2.0 to 3.0** (n=70)	12 weeks	Thromboembolic events and bleeding events.
**RE-LY**<br>OL/DB RCT, 2009.<br>Multicenter, 44 countries (including Europe / America).	Randomized: n=18113	AF patients with ≥ 1 risk factor for stroke, without recent stroke or increased risk of bleeding.	**Dabigatran 110 mg BID** (n=6015)<br>**Dabigatran 150 mg BID** (n=6076)<br>**OL Warfarin – INR 2.0 to 3.0** (n=6022)<br>Concomitant use of **ASA** (<100 mg daily) or other **antiplatelet agents** permitted.	2 years	Stroke or SE and major haemorrhage.
**ROCKET-AF**<br>DB RCT, 2011.<br>Multicenter, 45 countries (including Europe / America).	Randomized: n=14264	AF patients at moderate to high risk of stroke (CHADS2 score ≥ 2).	**Rivaroxaban 20 mg OD** (n=7131)<br>**Warfarin – INR 2.0 to 3.0** (n=7133)<br>Over a third of patients in both arms took concomitant ASA.	1.9 years	Composite of stroke and SE;<br>Composite of major and NMCR bleeding.

For peer review only - http://bmjopen.bmj.com/site/about/guidelines.xhtml

Study and Design	Disposition	Population	Interventions	Follow-up	Primary Outcome(s)
**WASPO**<br>OL RCT, 2007.<br>Multicenter, UK.	Randomized: n=75	Ambulant AF patients > 80 and < 90 years with Folstein score > 25, without previous fall, ICH or GI bleeding. Patients with prior stroke or TIA were excluded.	**Warfarin – INR 2.0 to 3.0** (n=36)<br>**Medium-dose ASA** (n=39)	1 year	Combined endpoints: death; stroke, TIA or SE; serious bleeding; withdrawal.
**Weitz et al 2010**<br>DB RCT, 2010.<br>Multicenter, 12 countries (including Eastern Europe / America).	Randomized: n=719	AF patients at moderate to high risk of stroke (CHADS2 score ≥ 2).	**Edoxaban 30 mg OD** (n=235)<br>**Edoxaban 60 mg OD** (n=234)<br>**Warfarin – INR 2.0 to 3.0** (n=250)	12 weeks	Bleeding and abnormalities of hepatic function
**Yamashita et al 2012**<br>DB RCT, 2012.<br>Multicenter, 1 country (Japan).	Randomized: n=385	AF patients at moderate to high risk of stroke (CHADS2 score ≥ 1).	**Edoxaban 30 mg OD** (n=130)<br>**Edoxaban 60 mg OD** (n=130)<br>**Warfarin – INR 2.0 to 3.0** (n=125)	12 weeks	All bleeding events

AF = atrial fibrillation; **ASA** = acetylsalicylic acid; **CNS** = central nervous system; **CRNM** = clinically relevant non-major bleeding; **CV** = cardiovascular; **DB** = double-blind; **GI** = gastrointestinal; **ICH** = intracerebral hemorrhage; **MI** = myocardial infarction; **NR** = not reported; **NSAIDs** = non-steroidal anti-inflammatory drugs; **OL** = open label; **PL** = placebo; **RCT** = randomized controlled trial; **SE** = systemic embolism; **TIA** = transient ischemic attack; **UK** = United-Kingdom; **VKA** = vitamin K antagonist.

* No data was extracted from these treatment arms since they did not meet the inclusion criteria specified in the protocol. These trials were nevertheless included in the systematic review because they had at least one relevant comparison between two interventions of interest.

† Data was extracted only for dabigatran without ASA (n=100).

For peer review only - http://bmjopen.bmj.com/site/about/guidelines.xhtml

Appendix 5: Evidence network for major bleeding. The width of the lines is proportional to the number of randomized controlled trials (RCTs) comparing each pair of treatments, and the size of each treatment node is proportional to the number of randomised participants (sample size). A dotted line indicates a three-arm RCT and a black node indicates a node included in the analysis but not reported in the main text.

For peer review only

For peer review only - http://bmjopen.bmj.com/site/about/guidelines.xhtml

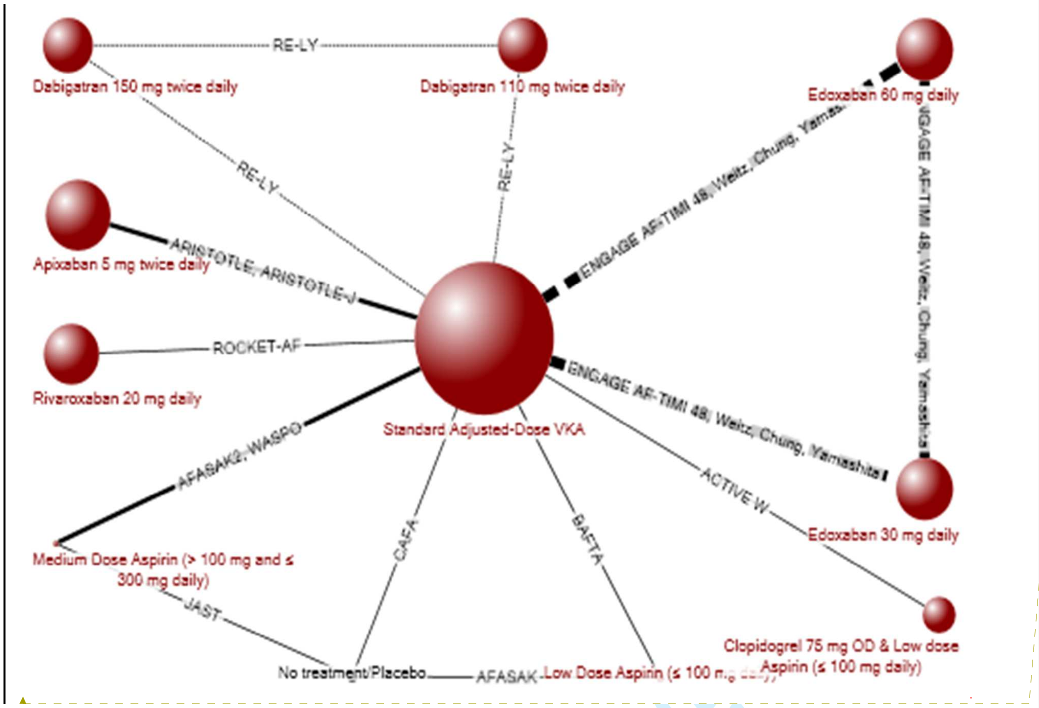

Formatted: Font: (Default) Arial, 11 pt

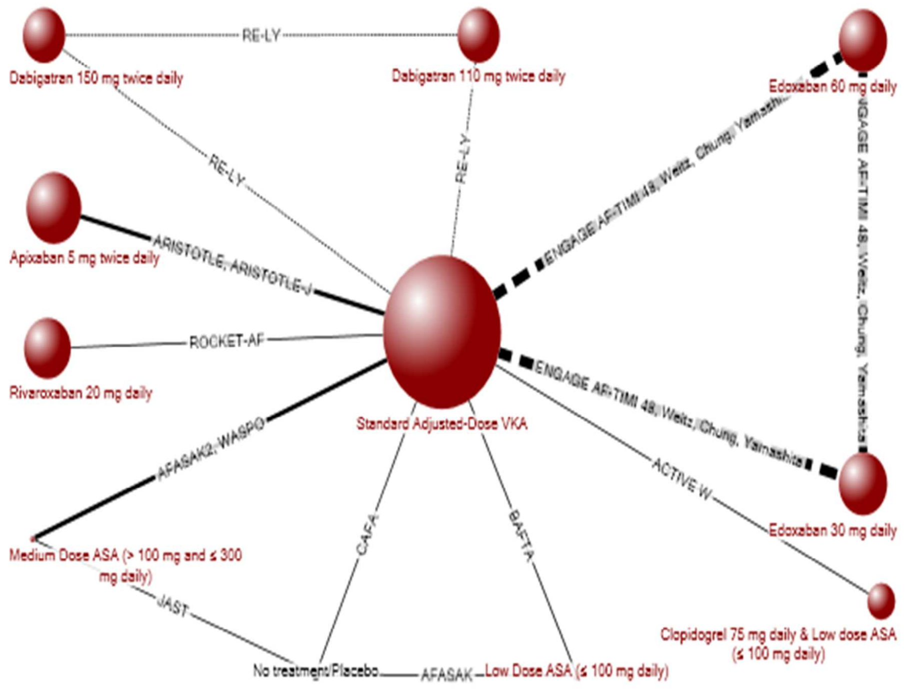

mg= milligram; RCT = randomized controlled trial

Appendix 6: Summary of results from Network Meta-analysis for All-cause Stroke or Systemic Embolism

Treatment	Pair-wise Meta-Analysis, OR (95% CI) versus standard adjusted dose VKA	NMA, Odds Ratio (95% CrI) versus standard adjusted dose	NMA, Absolute Risk Difference per 1,000 treated (95% CrI) versus standard adjusted dose VKA	Probability Best (%) for Outcome	Rank for Outcome	SUCRA

Formatted Table

31

		VKA				
~~Standard Adjusted-Dose VKA~~	~~NA~~	~~NA~~	~~NA~~	~~0.0%~~	~~6.0~~	~~50.2%~~
~~Apixaban 5 mg twice daily~~	~~0.79(0.66,0.94)~~	~~0.78 (0.65,0.94)~~	~~4 fewer (6 fewer-1 fewer)~~	~~11.2%~~	~~2.5~~	~~85.4%~~
Dabigatran 150 mg twice daily	0.66(0.53,0.82)	0.66 (0.53,0.82)	6 fewer (8 fewer-3 fewer)	85.2%	1.2	98.1%
Apixaban 5 mg twice daily	0.79(0.66,0.94)	0.78 (0.65,0.94)	4 fewer (6 fewer-1 fewer)	11.2%	2.5	85.4%
Edoxaban 60 mg daily	0.87(0.71,1.05)	0.87 (0.74,1.01)	3 fewer (5 fewer-1 more)	1.2%	3.7	72.8%
Rivaroxaban 20 mg daily	0.88(0.74,1.05)	0.87 (0.74,1.03)	3 fewer (5 fewer-1 more)	1.0%	3.9	71.2%
Dabigatran 110 mg twice daily	0.91(0.75,1.11)	0.91 (0.74,1.12)	2 fewer (5 fewer-2 more)	0.1%	4.5	64.7%
Standard Adjusted-Dose VKA	NA	NA	NA	0.0%	6.0	50.2%
Edoxaban 30 mg daily	1.14(0.94,1.37)	1.14 (0.98,1.32)	3 more (1 fewer-6 more)	0.0%	7.4	36.5%
Medium Dose Aspirin (> 100 mg and ≤ 300 mg daily)	1.21(0.51,2.87)	1.35 (0.74,2.47)	6 more (5 fewer-24 more)	1.2%	7.8	32.0%
No treatment/Placebo	1.84(0.66,5.1)	1.53 (0.9,2.63)	9 more (2 fewer-26 more)	0.1%	8.9	21.5%
~~Dabigatran 110 mg twice daily~~	~~0.91(0.75,1.11)~~	~~0.91 (0.74,1.12)~~	~~2 fewer (5 fewer-2 more)~~	~~0.1%~~	~~4.5~~	~~64.7%~~
~~Dabigatran 150 mg twice daily~~	~~0.66(0.53,0.82)~~	~~0.66 (0.53,0.82)~~	~~6 fewer (8 fewer-3 fewer)~~	~~85.2%~~	~~1.2~~	~~98.1%~~
~~Rivaroxaban 20 mg daily~~	~~0.88(0.74,1.05)~~	~~0.87 (0.74,1.03)~~	~~3 fewer (5 fewer-1 more)~~	~~1.0%~~	~~3.9~~	~~71.2%~~
Low Dose Aspirin (≤ 100 mg daily)	2.03(1.32,3.12)	1.87 (1.26,2.8)	14 more (5 more-29 more)	0.0%	10.1	9.4%
~~Medium Dose Aspirin (> 100 mg and ≤ 300 mg daily)~~	~~1.21(0.51,2.87)~~	~~1.35 (0.74,2.47)~~	~~6 more (5 fewer-24 more)~~	~~1.2%~~	~~7.8~~	~~32.0%~~
Clopidogrel 75 mg OD & Low dose Aspirin (≤ 100 mg daily)	1.93(1.41,2.64)	1.93 (1.42,2.65)	15 more (7 more-26 more)	0.0%	10.2	8.3%
~~Edoxaban 30 mg daily~~	~~1.14(0.94,1.37)~~	~~1.14 (0.98,1.32)~~	~~3 more (1 fewer-6 more)~~	~~0.0%~~	~~7.4~~	~~36.5%~~
~~Edoxaban 60 mg daily~~	~~0.87(0.71,1.05)~~	~~0.87 (0.74,1.01)~~	~~3 fewer (5 fewer-1 more)~~	~~1.2%~~	~~3.7~~	~~72.8%~~
~~No treatment/Placebo~~	~~1.84(0.66,5.1)~~	~~1.53 (0.9,2.63)~~	~~9 more (2 fewer-26 more)~~	~~0.1%~~	~~8.9~~	~~21.5%~~

CI = confidence interval; CrI = credible interval; OR= odds ratio

Bayesian network meta-analysis also allows the analyst to estimate the probability that a treatment is best, second best, and so on for a particular outcome. A simple numerical summary of these probabilities – the surface under the cumulative ranking (SUCRA) – for each treatment can also be calculated [50]. The SUCRA would be 100% when a treatment is certain to be the best and 0% when a treatment is certain to be the worst. SUCRA values enable the ranking of treatments overall for a particular outcome. For example, for Dabigatran 150 mg twice daily for stroke/SE the SUCRA value for is 98.1%, which is better than other treatments. By contrast, Clopidogrel 75 mg OD & Low dose Aspirin (≤ 100 mg daily) has a SUCRA of 8.3% for stroke/SE meaning it is certain to be worst for this outcome among active treatments.

For peer review only

Appendix 7: Summary of results from Network Meta-analysis for Major Bleeding

Treatment	Pair-wise Meta-Analysis, OR (95% CI) versus standard adjusted dose VKA	NMA, Odds Ratio (95% CrI) versus standard adjusted dose VKA	NMA, Absolute Risk Difference per 1,000 treated (95% CrI) versus standard adjusted dose VKA	Probability Best (%) for Outcome	Rank for Outcome	SUCRA
Standard Adjusted-Dose VKA	NA	NA	NA	0.0%	8.0	30.2%
Edoxaban 30 mg daily	0.46(0.39,0.55)	0.46(0.4,0.54)	18 fewer (20 fewer-15 fewer)	29.7%	1.7	92.9%
No treatment/Placebo	0.39(0.08,2.02)	0.33(0.08,1.08)	22 fewer (31 fewer-3 more)	70.2%	1.7	92.6%
Apixaban 5 mg twice daily	0.69(0.60,0.80)	0.69(0.6,0.8)	10 fewer (13 fewer-7 fewer)	0.0%	3.2	78.1%
Edoxaban 60 mg daily	0.78(0.67,0.92)	0.79(0.69,0.9)	7 fewer (10 fewer-4 fewer)	0.0%	4.5	64.6%
Dabigatran 110 mg twice daily	0.8(0.68,0.94)	0.8(0.69,0.93)	7 fewer (10 fewer-3 fewer)	0.0%	4.7	62.7%
Dabigatran 150 mg twice daily	0.93(0.81,1.07)	0.93(0.8,1.08)	3 fewer (7 fewer-3 more)	0.0%	6.8	41.7%
Low Dose Aspirin (≤ 100 mg daily)	1.01(0.57,1.78)	1.05(0.6,1.86)	2 more (13 fewer-26 more)	0.1%	7.9	30.6%
Standard Adjusted-Dose VKA	NA	NA	NA	0.0%	8.0	30.2%
Rivaroxaban 20 mg daily	1.03(0.9,1.18)	1.03(0.89,1.18)	1 more (4 fewer-6 more)	0.0%	8.4	26.3%
Clopidogrel 75 mg OD & Low dose Aspirin (≤ 100 mg daily)	1.1(0.82,1.48)	1.1(0.83,1.47)	4 more (6 fewer-15 more)	0.0%	8.9	20.8%
Low Dose Aspirin (≤ 100 mg daily)	1.01(0.57,1.78)	1.05(0.6,1.86)	2 more (13 fewer-26 more)	0.1%	7.9	30.6%
Medium Dose Aspirin (> 100 mg and ≤ 300 mg daily)	1.68(0.50,5.69)	1.79(0.63,5.67)	24 more (12 fewer-120 more)	0.0%	10.0	9.8%
Clopidogrel 75 mg OD & Low dose Aspirin (≤ 100 mg daily)	1.1(0.82,1.48)	1.1(0.83,1.47)	4 more (6 fewer-15 more)	0.0%	8.9	20.8%
Edoxaban 30 mg daily	0.46(0.39,0.55)	0.46(0.4,0.54)	18 fewer (20 fewer-15 fewer)	29.7%	1.7	92.9%
Edoxaban 60 mg daily	0.78(0.67,0.92)	0.79(0.69,0.9)	7 fewer (10 fewer-4 fewer)	0.0%	4.5	64.6%
No treatment/Placebo	0.39(0.08,2.02)	0.33(0.08,1.08)	22 fewer (31 fewer-3 more)	70.2%	1.7	92.6%

Formatted Table

CI = confidence interval; CrI = credible interval; OR= odds ratio

Bayesian network meta-analysis also allows the analyst to estimate the probability that a treatment is best, second best, and so on for a particular outcome. A simple numerical summary of these probabilities – the surface under the cumulative ranking (SUCRA) – for each treatment can also be calculated [50]. The

For peer review only - http://bmjopen.bmj.com/site/about/guidelines.xhtml

SUCRA would be 100% when a treatment is certain to be the best and 0% when a treatment is certain to be the worst. SUCRA values enable the ranking of treatments overall for a particular outcome. For example, for major bleeding the SUCRA value for Edoxaban 60 mg is 92.8%, which is better than other treatments. By contrast, Medium Dose Aspirin (> 100 mg and ≤ 300 mg daily) has a SUCRA of 9.9% for major bleeding meaning it is certain to be worst for this outcome.

For peer review only

Appendix 8: Benefit-Harm plot for each treatment (absolute harm difference per 1,000 patients treated). The absolute risk difference of recurrence of Stroke or systemic embolism relative to standard adjusted dose VKA is presented on the x-axis, while the absolute risk difference of major bleeding relative to standard adjusted dose VKA is on the y-axis. The dot is representative of the median estimate for each treatment while the 95% credible intervals are presented as error bars.

For peer review only - http://bmjopen.bmj.com/site/about/guidelines.xhtml

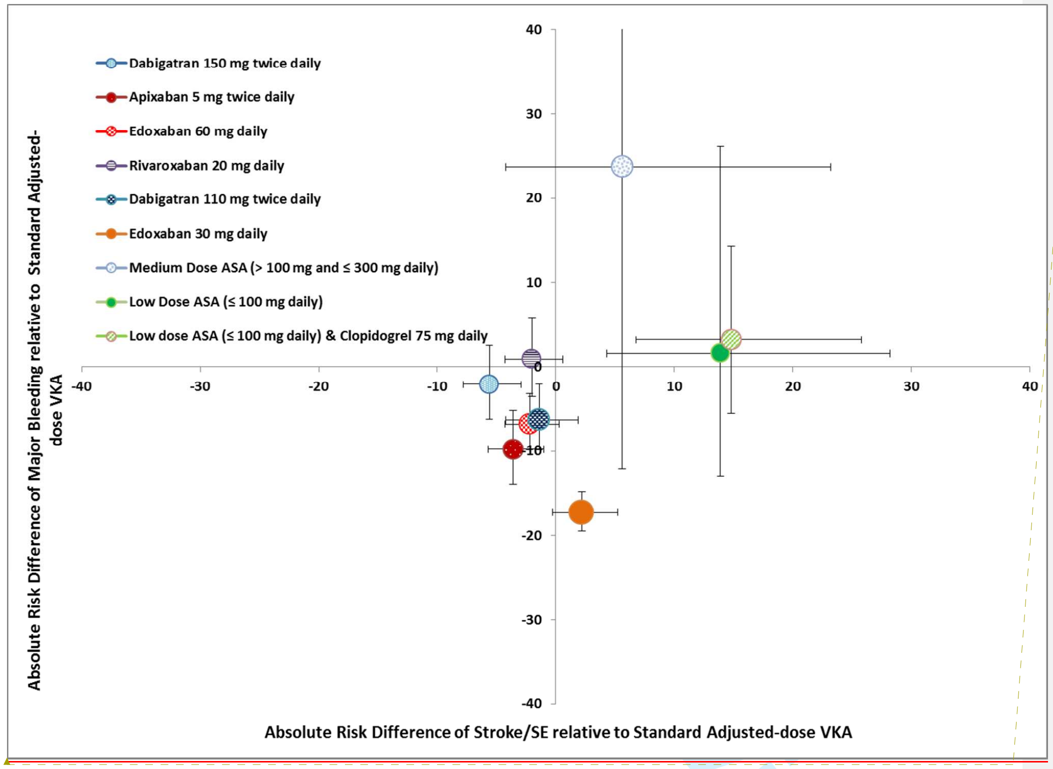

Formatted: Font:

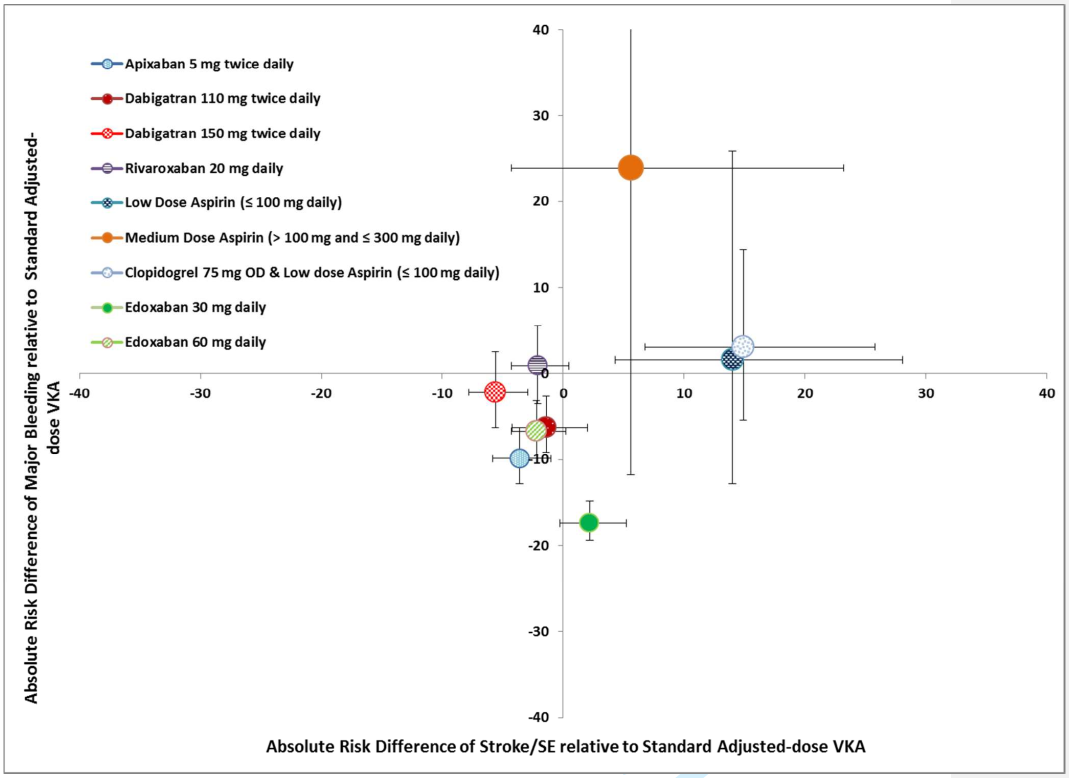
Apixaban 5 mg twice daily
Dabigatran 110 mg twice daily
Dabigatran 150 mg twice daily
Rivaroxaban 20 mg daily
Low Dose Aspirin (≤ 100 mg daily)
Medium Dose Aspirin (> 100 mg and ≤ 300 mg daily)
Clopidogrel 75 mg OD & Low dose Aspirin (≤ 100 mg daily)
Edoxaban 30 mg daily
Edoxaban 60 mg daily
Absolute Risk Difference of Major Bleeding relative to Standard Adjusted-dose VKA
Absolute Risk Difference of Stroke/SE relative to Standard Adjusted-dose VKA
40
30
20
10
0
-10
-20
-30
-40

Appendix 9: Rationale of choice of fixed-effects model and limitations

It is challenging to conduct network meta-analyses when evidence structures are comprised of single study connections, especially when there is obvious heterogeneity in the network. The use of a random-effects model with a vague prior on the between study variance exerts a large degree of influence on the credible intervals (CrI's) because there is insufficient studies to reign in the prior and provide an accurate estimate of the between study variance. As a result, results often appear unrealistic (e.g., wide credible intervals for a treatment that was shown to be associated with a statistically significant decrease). Accordingly, we also considered informative priors on the between study variance from the recently published reports.[11,44] However, even when these priors were applied on the between study variance, they still did not reign in CrI's effect estimates again because the network was comprised of single study connections. The use of the random effects model with either a vague or informative prior was problematic because treatments that were originally associated with statistically significant reductions in stroke or SE relative to standard adjusted dose VKA were no longer associated with significant reductions, limiting external validity of the model (see Figures below on direct estimates in Online Appendix 4 and 5). While we could have opted to use less vague priors (e.g., ¼ of prior in Turner et al[11]), the choice of the prior would have seemed somewhat arbitrary because it was not based on any empirical evidence. As such, we opted to use the fixed-effects model despite the known issues in doing so. By using the fixed-effects model, treatments that were associated with significant reductions in the risk of stroke or SE relative to standard adjusted dose VKA retained significance. We felt that it would have been inappropriate to remove statistical significance by applying vague priors given the large investment involved in developing clinical trials. However, a consequence of retaining statistical significance is seen when comparing newer oral anticoagulants versus one another in Figure 3~~5~~. Because we assumed the new oral anticoagulants were all associated with reductions in Stroke or SE relative to standard adjusted dose VKA (if that was the case in the underlying trials), significant reductions in the risk of stroke or SE among new oral anticoagulants are observed in Figure 5. This may or may not be the case in clinical practice. As noted in tables 1 and Online Appendix 2, there is considerable heterogeneity across studies. However, the network structure itself (i.e., single study connections), made it difficult to properly adjust for differences in patient and study characteristics using standard approaches. Accordingly, results in Figure 3~~5~~ should be interpreted with caution. We conducted simple sub-group analyses (breaking randomization) to help adjust for this heterogeneity, which is a very crude way to adjust for heterogeneity. To fully elucidate the comparative benefits and harms of antithrombotic agents, especially across the various sub-populations, rigorously conducted comparative studies, or network meta-regression analyses of patient-level data are required.

For peer review only - http://bmjopen.bmj.com/site/about/guidelines.xhtml

Forest Plot – Odds ratio (95% Credible interval) when using informative prior on the sd (i.e, dlnorm(-3.02,0.29)[11]

Formatted: Font: (Default) Arial, 11 pt, Bold

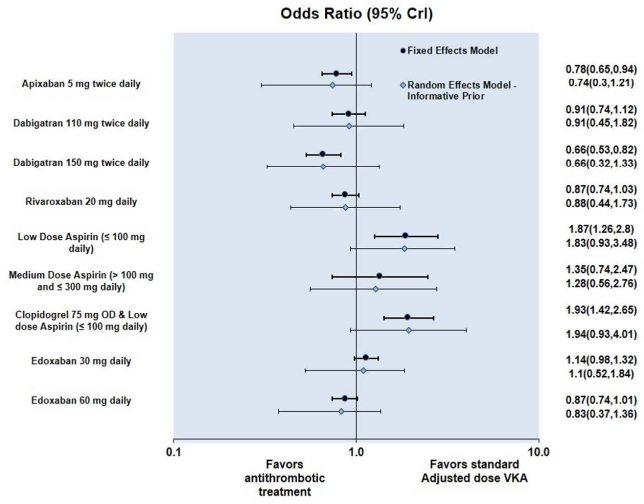

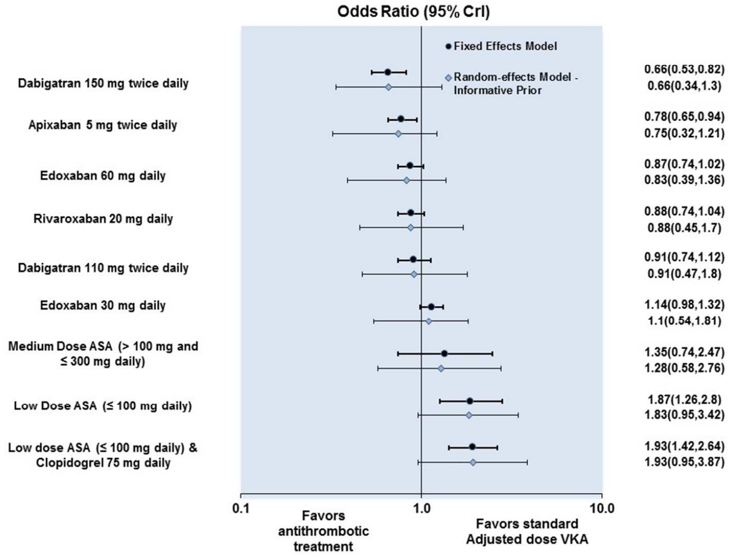

Forest Plot – Odds ratio (95% Credible interval) when using vague prior on the sd (i.e, duniform(0,2))

For peer review only - http://bmjopen.bmj.com/site/about/guidelines.xhtml

Formatted: Font: (Default) Arial, 11 pt, Bold

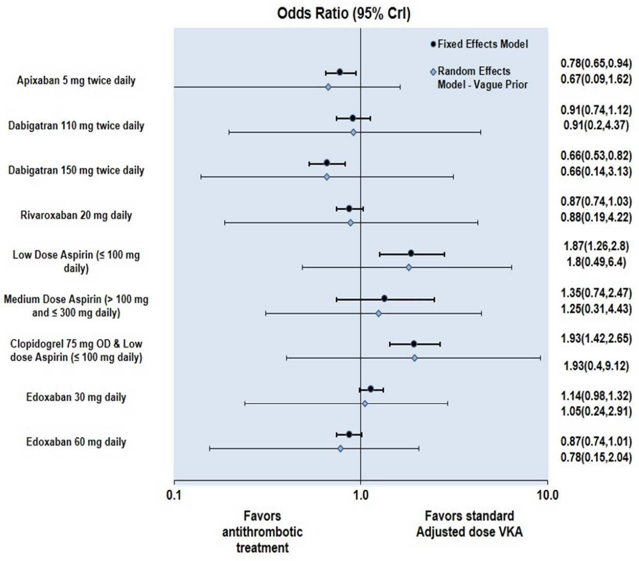

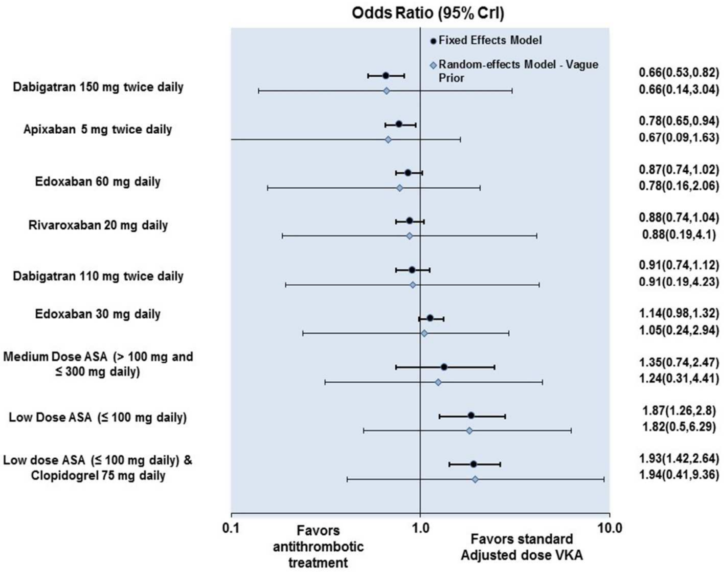

Appendix 10: PRISMA Checklist

For peer review only - http://bmjopen.bmj.com/site/about/guidelines.xhtml

Section/topic	#	Checklist item	Reported on page #
**TITLE**			
Title	1	Identify the report as a systematic review, meta-analysis, or both.	0
**ABSTRACT**			
Structured summary	2	Provide a structured summary including, as applicable: background; objectives; data sources; study eligibility criteria, participants, and interventions; study appraisal and synthesis methods; results; limitations; conclusions and implications of key findings; systematic review registration number.	0
**INTRODUCTION**			
Rationale	3	Describe the rationale for the review in the context of what is already known.	1
Objectives	4	Provide an explicit statement of questions being addressed with reference to participants, interventions, comparisons, outcomes, and study design (PICOS).	1
**METHODS**			
Protocol and registration	5	Indicate if a review protocol exists, if and where it can be accessed (e.g., Web address), and, if available, provide registration information including registration number.	2
Eligibility criteria	6	Specify study characteristics (e.g., PICOS, length of follow-up) and report characteristics (e.g., years considered, language, publication status) used as criteria for eligibility, giving rationale.	2-3
Information sources	7	Describe all information sources (e.g., databases with dates of coverage, contact with study authors to identify additional studies) in the search and date last searched.	2-3
Search	8	Present full electronic search strategy for at least one database, including any limits used, such that it could be repeated.	2-3
Study selection	9	State the process for selecting studies (i.e., screening, eligibility, included in systematic review, and, if applicable, included in the meta-analysis).	3
Data collection process	10	Describe method of data extraction from reports (e.g., piloted forms, independently, in duplicate) and any processes for obtaining and confirming data from investigators.	3
Data items	11	List and define all variables for which data were sought (e.g., PICOS, funding sources) and any assumptions and simplifications made.	3-4
Risk of bias in individual studies	12	Describe methods used for assessing risk of bias of individual studies (including specification of whether this was done at the study or outcome level), and how this information is to be used in any data synthesis.	3,6
Summary measures	13	State the principal summary measures (e.g., risk ratio, difference in means).	3
Synthesis of results	14	Describe the methods of handling data and combining results of studies, if done, including measures of consistency (e.g., I^2) for each meta-analysis.	4-5
Risk of bias across studies	15	Specify any assessment of risk of bias that may affect the cumulative evidence (e.g., publication bias, selective reporting within studies).	5
Additional analyses	16	Describe methods of additional analyses (e.g., sensitivity or subgroup analyses, meta-regression), if done, indicating which were pre-specified.	5
**RESULTS**			
Study selection	17	Give numbers of studies screened, assessed for eligibility, and included in the review, with reasons for exclusions at each stage, ideally with a flow diagram.	6, Online Appendix 2~~3,5~~

For peer review only - http://bmjopen.bmj.com/site/about/guidelines.xhtml

Study characteristics	18	For each study, present characteristics for which data were extracted (e.g., study size, PICOS, follow-up period) and provide the citations.	5-6, ~~Table 1,~~ Online Appendix 3~~2~~
Risk of bias within studies	19	Present data on risk of bias of each study and, if available, any outcome level assessment (see item 12).	7~~5~~
Results of individual studies	20	For all outcomes considered (benefits or harms), present, for each study: (a) simple summary data for each intervention group (b) effect estimates and confidence intervals, ideally with a forest plot.	Figure 2~~3~~-4
Synthesis of results	21	Present results of each meta-analysis done, including confidence intervals and measures of consistency.	Figure 2~~3~~-4, Online Appendix 6,7~~4~~ and 9~~5~~
Risk of bias across studies	22	Present results of any assessment of risk of bias across studies (see Item 15).	7~~5~~
Additional analysis	23	Give results of additional analyses, if done (e.g., sensitivity or subgroup analyses, meta-regression [see Item 16]).	Table 1~~-2-3~~, Online Appendix 12~~0~~
**DISCUSSION**			
Summary of evidence	24	Summarize the main findings including the strength of evidence for each main outcome; consider their relevance to key groups (e.g., healthcare providers, users, and policy makers).	11-12~~2~~
Limitations	25	Discuss limitations at study and outcome level (e.g., risk of bias), and at review-level (e.g., incomplete retrieval of identified research, reporting bias).	14~~3~~-15~~4~~
Conclusions	26	Provide a general interpretation of the results in the context of other evidence, and implications for future research.	10-16~~4~~
**FUNDING**			
Funding	27	Describe sources of funding for the systematic review and other support (e.g., supply of data); role of funders for the systematic review.	0

Appendix 11: Assessment of Inconsistency

For peer review only - http://bmjopen.bmj.com/site/about/guidelines.xhtml

We plotted the posterior mean deviance of the individual data points in the inconsistency model against their posterior mean deviance in the consistency model to help identify loops where inconsistency is present. In our analysis, the posterior mean deviance contributions are very similar and close to 1, for both models. The consistency model has a similar posterior mean of the residual deviance (29.41 vs. 29.37) and DIC (195.00 vs. 196.87) and hence is a similar fit for both models. A few points show a lower value of the posterior mean deviance in the inconsistency model but these values are not too far from 1. Similarly, a few points have higher values of the posterior mean deviance in the consistency model (again with values not too far from 1) suggesting that each model may fit the data points better for certain points. The parameter estimates are also similar for both models and there is considerable overlap in the 95% credible intervals (see table below), suggesting no evidence of inconsistency in the network.

Plot of posterior mean deviance of the individual data points in the inconsistency model against their posterior mean deviance in the consistency model

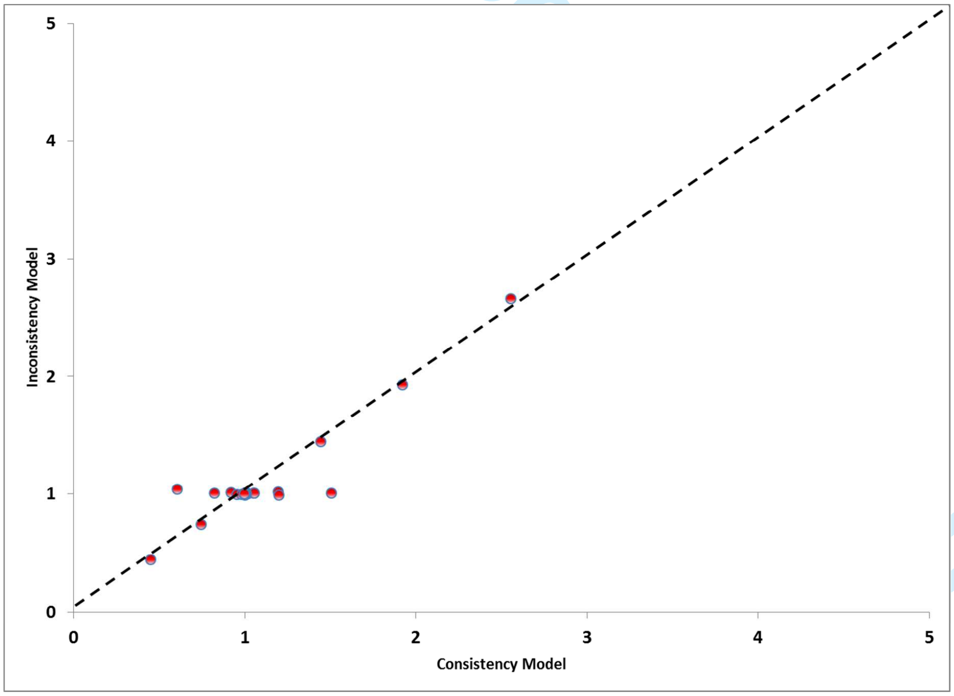

For peer review only - http://bmjopen.bmj.com/site/about/guidelines.xhtml

Comparison of effect estimates from consistency and inconsistency models where there is data available for comparison

Comparison	Consistency Model	Inconsistency Model
~~Apixaban 5 mg twice daily vs Standard Adjusted-Dose VKA~~	~~0.78 (0.65,0.94)~~	~~0.78 (0.65,0.94)~~
Dabigatran 150 mg twice daily vs Standard Adjusted-Dose VKA	0.66 (0.53,0.82)	0.66 (0.53,0.82)
Apixaban 5 mg twice daily vs Standard Adjusted-Dose VKA	0.78 (0.65,0.94)	0.78 (0.65,0.94)
Edoxaban 60 mg daily vs Standard Adjusted-Dose VKA	0.87 (0.74,1.01)	0.87 (0.74,1.01)
Rivaroxaban 20 mg daily vs Standard Adjusted-Dose VKA	0.87 (0.74,1.03)	0.88 (0.74,1.03)
Dabigatran 110 mg twice daily vs Apixaban 5 mg twice daily vs	0.91 (0.74,1.12)	0.91 (0.74,1.12)
Edoxaban 30 mg daily vs Standard Adjusted-Dose VKA	1.14 (0.98,1.32)	1.14 (0.98,1.32)
Medium Dose Aspirin (> 100 mg and ≤ 300 mg daily) vs Standard Adjusted-Dose VKA	1.35 (0.74,2.47)	0.82 (0.33,1.98)
Low Dose Aspirin (≤ 100 mg daily) vs Standard Adjusted-Dose VKA	1.87 (1.26,2.8)	2.04 (1.33,3.19)
Clopidogrel 75 mg OD & Low dose Aspirin (≤ 100 mg daily) vs Standard Adjusted-Dose VKA	1.93 (1.42,2.64)	1.93 (1.42,2.65)
No treatment/Placebo vs Low Dose Aspirin (≤ 100 mg daily)	0.82 (0.48,1.38)	1 (0.51,1.98)
~~Dabigatran 110 mg twice daily vs Apixaban 5 mg twice daily vs~~	~~0.91 (0.74,1.12)~~	~~0.91 (0.74,1.12)~~
~~Dabigatran 150 mg twice daily vs Standard Adjusted-Dose VKA~~	~~0.66 (0.53,0.82)~~	~~0.66 (0.53,0.82)~~
~~Rivaroxaban 20 mg daily vs Standard Adjusted-Dose VKA~~	~~0.87 (0.74,1.03)~~	~~0.88 (0.74,1.03)~~
~~Low Dose Aspirin (≤ 100 mg daily) vs Standard Adjusted-Dose VKA~~	~~1.87 (1.26,2.8)~~	~~2.04 (1.33,3.19)~~
~~Medium Dose Aspirin (> 100 mg and ≤ 300 mg daily) vs Standard Adjusted-Dose VKA~~	~~1.35 (0.74,2.47)~~	~~0.82 (0.33,1.98)~~
~~Clopidogrel 75 mg OD & Low dose Aspirin (≤ 100 mg daily) vs Standard Adjusted-Dose VKA~~	~~1.93 (1.42,2.65)~~	~~1.93 (1.42,2.65)~~
~~Edoxaban 30 mg daily vs Standard Adjusted-Dose VKA~~	~~1.14 (0.98,1.32)~~	~~1.14 (0.98,1.32)~~
~~Edoxaban 60 mg daily vs Standard Adjusted-Dose VKA~~	~~0.87 (0.74,1.01)~~	~~0.87 (0.74,1.01)~~
~~No treatment/Placebo vs Standard Adjusted-Dose VKA~~	~~1.53 (0.9,2.63)~~	~~1.91 (0.7,5.81)~~
~~No treatment/Placebo vs Low Dose Aspirin (≤ 100 mg daily)~~	~~0.82 (0.48,1.38)~~	~~1 (0.51,1.98)~~
**Residual deviance**	29.41	29.37
**DIC**	195.00	196.87

For peer review only - http://bmjopen.bmj.com/site/about/guidelines.xhtml

Appendix 12: Sensitivity analysis with AVERROES and ACTIVE A

A. Stroke or SE

Evidence Networks for Stroke or SE NMA including AVEROES and ACTIVE A studies

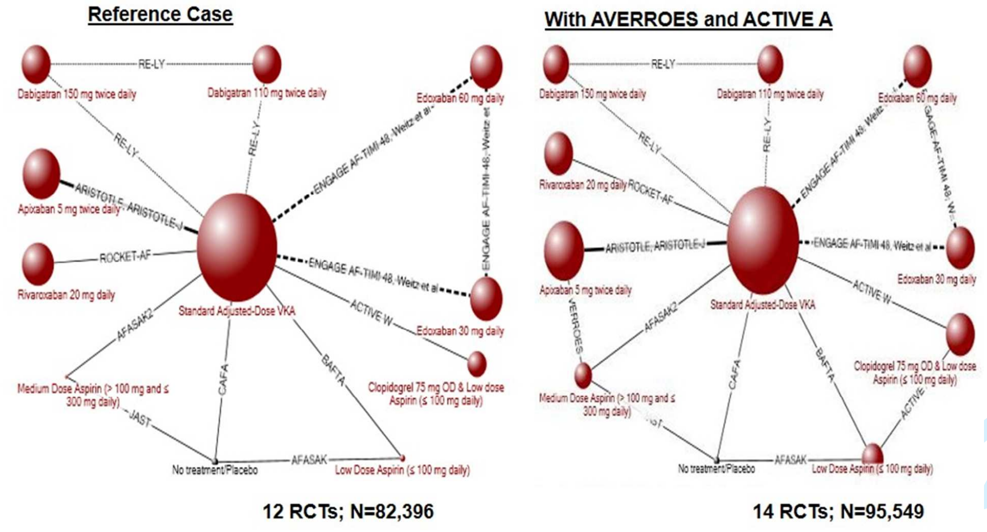

Forest Plot for Stroke or SE NMA including AVERROES

For peer review only - http://bmjopen.bmj.com/site/about/guidelines.xhtml

and ACTIVE A studies

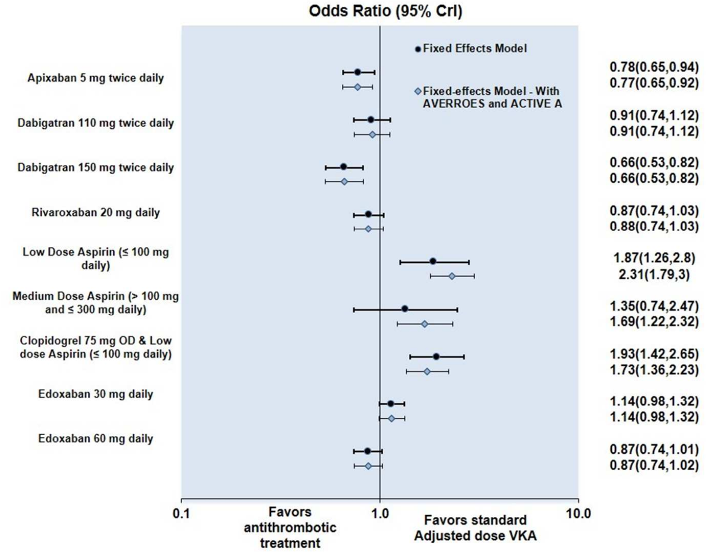

Formatted: Font: (Default) Times New Roman, Font color: Auto

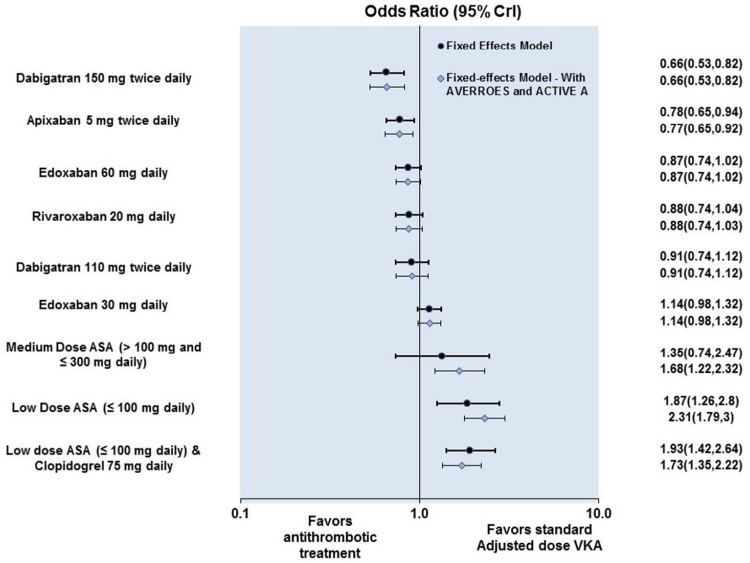

Formatted: Font: (Default) Times New Roman, Font color: Auto

For peer review only - http://bmjopen.bmj.com/site/about/guidelines.xhtml

B. Major Bleeding

Evidence Networks for Major Bleeding NMA including AVERROES and ACTIVE A studies

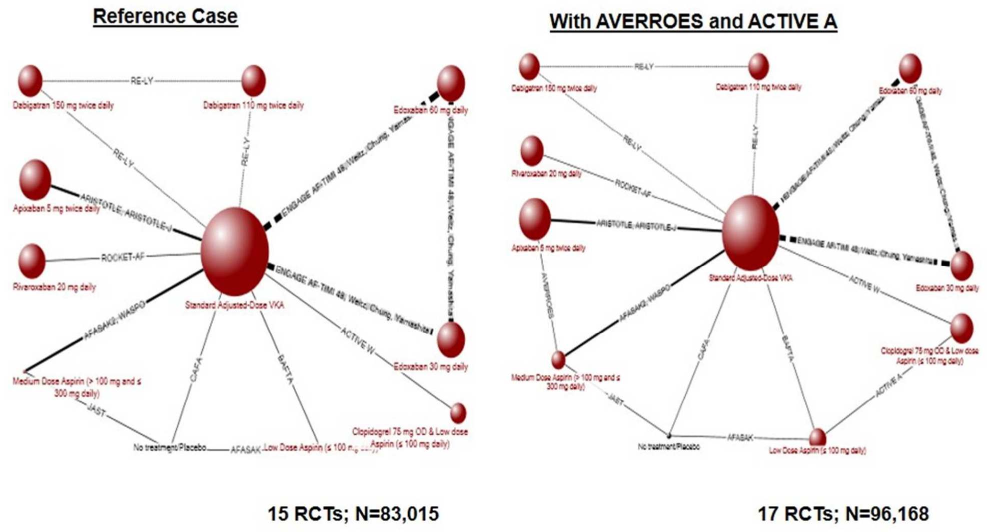

Forest Plot for Major Bleeding NMA including AVERROES and ACTIVE A studies

For peer review only - http://bmjopen.bmj.com/site/about/guidelines.xhtml

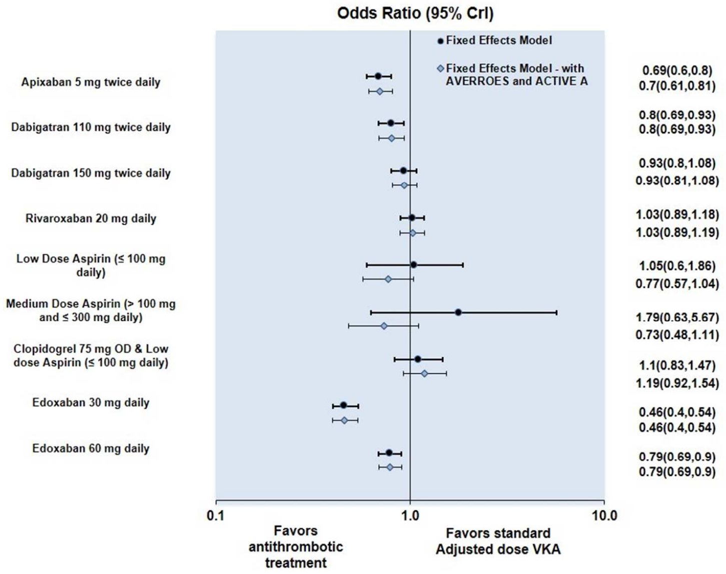

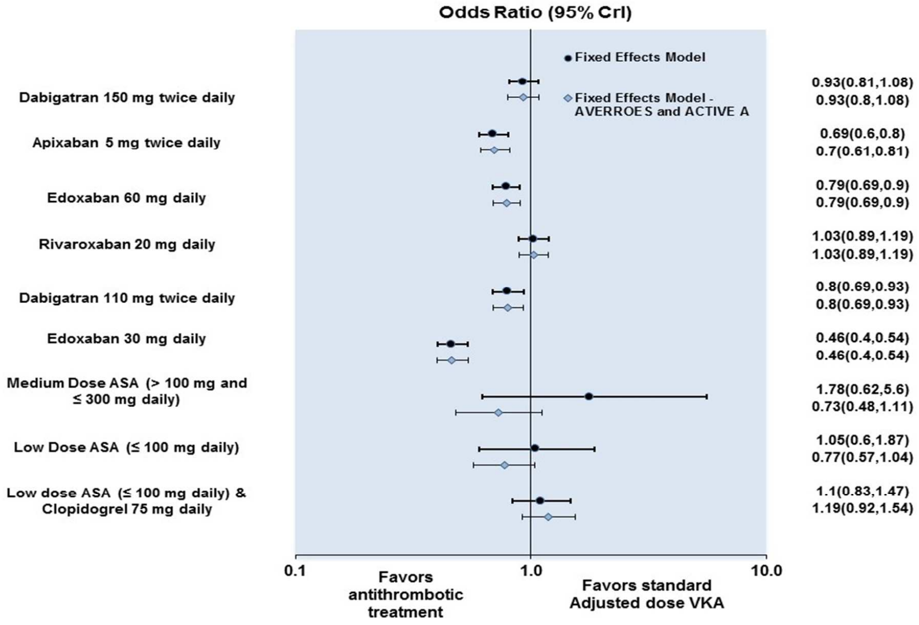

Formatted: Font: (Default) Times New Roman, 11 pt, Font color: Auto

For peer review only - http://bmjopen.bmj.com/site/about/guidelines.xhtml

Appendix 13: Comparison with previously published network meta-analyses since Harenberg et al[30]

Author (Publication Year)	Method	Search Date	Included Treatments	Registered Protocol	Relative Measure(s)	Absolute Measure(s) reported	Subgroup Analyses Reported
**Cameron 2014**	Bayesian Network Meta-analysis	January 2014	dabigatran, rivaroxaban, apixaban, edoxaban, and vitamin K antagonists (VKA) at a standard adjusted dose (target international normalised ratio 2.0-3.0), acetylsalicylic acid, acetylsalicylic acid and clopidogrel	Yes (PROSPERO registry - CRD42012002721).	OR (95% CrI)	Yes	Yes
**Ruff 2013**	Traditional Meta-analysis	November 2013	dabigatran, rivaroxaban, apixaban, edoxaban	Not Reported	RR (95%CI)	No	Yes
**Dogliotti 2013**	Bayesian Network Meta-analysis	May 2012	dabigatran, rivaroxaban, apixaban, vitamin K antagonists (VKA) at a standard adjusted dose, acetylsalicylic acid, acetylsalicylic acid and clopidogrel	Yes (metacardio.org)	OR (95% CrI)	No	No
**Assiri 2013**	Baysian Network Meta-analysis	August 2012	dabigatran, rivaroxaban, apixaban, vitamin K antagonists (VKA) at a standard adjusted dose, acetylsalicylic acid, acetylsalicylic acid and clopidogrel	Not Reported	RR (95%CrI)	No	No
**Bruins 2013**	Traditional Meta-analysis	June 2013	antistasin, apixaban, betrixaban, darexaban, DU176b, edoxaban, eribaxaban, fondaparinux, idraparinux, otamixaban, razaxaban, rivaroxaban, yagin, YM150, LY517717, SSR126517E, vitamin K antagonists (warfarin and congeners) with the intensity of anticoagulation dose-adjusted using the International Normalised Ratio (INR)	Yes (Cochrane Library)	OR (95%CI)<br>RR (95%CI) in summary table	Yes	Yes

For peer review only - http://bmjopen.bmj.com/site/about/guidelines.xhtml

Appendix 14: Excluded Study List

Inappropriate Study Design

Cardiovasc J S Afr. 2006 Mar;17(2):91.
J Fam Pract. 2006;55(9):753.
Cardiovasc J Afr. 2009 May;20(3):210.
Cardiovasc J Afr. 2009 Sep;20(5):311-2.
Australian Journal of Pharmacy. 2009;90(1073):68.
Formulary. 2010;45(1):16.
Aalbers J. Cardiovasc J Afr. 2010 Nov;21(6):342-3.
Alexander W, et al. PT. 2010;35(10):580-1.
Belavic JM. Nurse Pract. 2011 Sep;36(9):6-7.
Camm AJ. Eur Heart J. 2009 Nov;30(21):2554-5.
Chua D, et al. N Engl J Med. 2009;361(13):1312-3.
Connolly SJ. Eur J Heart Fail. 2009;11(12):1215.
DeSilvey DL. Am J Geriatr Cardiol. 2006 Sep;15(5):326-7.
Dunn A. Ann Intern Med. 2011;154(8):JC4-3.
Eikelboom JW, et al. J Thromb Haemost. 2012;10(5):966-8.
Ezekowitz MD, et al. J Interv Card Electrophysiol. 2011 Dec;32(3):173-80.
Garcia D, et al. Lancet. 2007 Aug 11;370(9586):460-1.
Granger CB. Ann Intern Med. 2012;156(JC1-2):JC1-3.
Hart RG, et al. Ann Intern Med. 2007;147(8):590-2.
Katona A, et al. Eur Heart J. 2006 Jun;27(11):1382.
Kita K, et al. Interventional Cardiology. 2011;3(6):637.
Lopes RD, et al. Evid Based Med. 2011;16(6):187-8.
Nolan J, et al. Br Heart J. 1992 Dec;68(6):544-7.
Paikin JS, et al. Expert Rev Cardiovasc Ther. 2011 Mar;9(3):279-86.
Panichpisal K, et al. Future Neurology. 2011;6(2):155-8.
Roy B, et al. Am J Cardiol. 2012 Feb 1;109(3):370-7.
Sloan MA. Evid Based Med. 2006;11(6):170.
Vassiliou VS. N Engl J Med. 2012;366(1):88-90.
Veloso HH, et al. Chest. 2005 Jul;128(1):475.
Weinberger J. Curr Cardiol Rep. 2010;12(1):1-2.

Inappropriate Intervention

Lancet. 1994 Mar 19;343(8899):687-91.
The Stroke Prevention in Atrial Fibrillation Investigators. Arch Intern Med. 1996 Feb 26;156(4):409-16.
Archer SL, et al. Am Heart J. 1995 Aug;130(2):287-95.
Edvardsson N, et al. J Intern Med. 2003 Jul;254(1):95-101.
Hart RG, et al. Cerebrovasc Dis. 2000 Jan;10(1):39-43.
Hellemons BS, et al. Control Clin Trials. 1999 Aug;20(4):386-93.
Hori M, et al. Circ J. 2012 Jun 5.
Miller VT, et al. Neurology. 1996 Jan;46(1):238-40.
Perez-Gomez F, et al. Eur Heart J. 2007;28(8):996-1003.

For peer review only

Singer DE, et al. Am Heart J. 1992 Dec;124(6):1567-73.
Connolly S, et al. Eur Heart J. 2013 May:34(20):1498-505

Inappropriate Population

ACTIVE Investigators, et al. N Engl J Med 2009;360(20):2066-78.
Circulation 1991;84(2):527-39.
Connolly SJ, et al. N Engl J Med 2011;364(9):806-17.
Dewilde W, et al. Am Heart J. 2009 Nov;158(5):713-8.
Diener HC, et al. Lancet Neurol. 2012 Mar;11(3):225-31.
EAFT (European Atrial Fibrillation Trial) Study Group. Lancet. 1993;342(8882):1255-62.
Eikelboom JW, et al. J Stroke Cerebrovasc Dis. 2012 Aug;21(6):429-35.
ESPRIT Study Group, et al. Lancet Neurol. 2007 Feb;6(2):115-24.
Hart RG, et al. Cerebrovasc Dis. 2008;25(4):344-7.
N Engl J Med 1990;322(12):863-8.
Wang TH, et al. Eur Heart J. 2007 Sep;28(18):2200-7.

Inappropriate Outcomes

De Caterina R, et al. Eur Heart J. 2010 Sep;31(17):2133-40.
Ezekowitz MD, et al. Circulation. 1995 Oct 15;92(8):2178-82.
Lorenzoni R, et al. Am Heart J. 2004 Jul;148(1):e6.

Others

Cardiovasc J Afr. 2011 May;22(3):164.
Active Steering Committee, et al. Am Heart J. 2006 Jun;151(6):1187-93.
ROCKET AF Study Investigators. Am Heart J. 2010 Mar;159(3):340-7.
The Stroke Prevention in Atrial Fibrillation Investigators. Stroke. 1990 Apr;21(4):538-45.
Boysen G, et al. Stroke. 1990;21(Suppl I):I-92.
De Schryver EL. Cerebrovasc Dis. 2000 Mar;10(2):147-50.
Desai A. Clinical Trials Registry - India (CTRI). 2006.
Eikelboom JW, et al. Am Heart J. 2010 Mar;159(3):348-53.
Ezekowitz MD, et al. Am Heart J. 2009 May;157(5):805-10, 810.e1-2.
Flaker G, et al. J Am Coll Cardiol. 2012 Feb 28;59(9):854-5.
Lopes RD, et al. Lancet. 2012 Oct 2. Epub ahead of print.
Lopes RD, et al. Am Heart J. 2010 Mar;159(3):331-9.
Mant J, et al. Cerebrovasc Dis. 2007;23(Suppl 2):10.
Mant J, et al. Cardiol Rev. 2008;25(7):32-6.
Mant JW, et al. BMC Cardiovasc Disord. 2003 Aug 26;3:9.
Posada IS, et al. Am Heart J. 1999 Jul;138(1 Pt 1):137-43.
Van Latum JC. Ned Tijdschr Geneeskd. 1994;138(20):1025-31.
Yigit Z. Turk Kardiyoloji Dernegi Arsivi. 2000;28(1):8-19+4.

For peer review only

SUPPLEMENTARY APPENDIX

Appendix 1: Search Strategy

OVERVIEW

Interface:	Ovid
Databases:	EBM Reviews - Cochrane Central Register of Controlled Trials <May 2012><br>Ovid Embase 1974 to June 6, 2012<br>Ovid MEDLINE 1946 to June 7, 2012<br>Ovid MEDLINE Daily<br>Ovid MEDLINE In-Process & Other Non-Indexed Citations<br>**Note:** Subject headings have been customized for each database. Duplicates between databases were removed in Ovid.
Date of Search:	June 7, 2012; updated January 23, 2014
Alerts:	Monthly search updates began June 11, 2012 and ran until project completion
Study Types:	Randomized Controlled Trials
Limits:	Publication years 1988-present<br>Humans<br>Conference abstracts omitted

SYNTAX GUIDE

/	At the end of a phrase, searches the phrase as a subject heading
.sh	At the end of a phrase, searches the phrase as a subject heading
MeSH	Medical Subject Heading
exp	Explode a subject heading
*	Before a word, indicates that the marked subject heading is a primary topic;<br>or, after a word, a truncation symbol (wildcard) to retrieve plurals or varying endings
#	Truncation symbol for one character
ADJ	Requires words are adjacent to each other (in any order)
ADJ#	Adjacency within # number of words (in any order)
.ti	Title
.ab	Abstract
.hw	Heading Word; usually includes subject headings and controlled vocabulary
.nm.	Name of substance word
.ot	Original title
.pt	Publication type
.rn	CAS registry number
use cctr	Select results from Cochrane database of Clinical Trials
use oemezd	Select results from Embase database
use pmez	Select results from Medline database

Clinical Multi-database Strategy

Line #	Search Strategy
1	(211914-51-1 or 211915-06-9).rn,nm. or (dabigatran* or Pradaxa or Pradax or Prazaxa or Praxada or bibr 1048 or bibr1048 or bibr953 or bibr 953 or rendix).ti,ot,ab,sh,rn,hw,nm.
2	(BMS 562247 or BMS562247 or apixaban* or Eliquis or Eliques).ti,ot,ab,sh,rn,hw,nm. or 503612-47-3.rn,nm.
3	(rivaroxaban* or Xarelto or BAY 59-7939 or BAY59-7939 or BAY597939 or BAY

For peer review only

	597939).ti,ot,ab,sh,rn,hw,nm. or 366789-02-8.rn,nm.
4	(90055-48-4 or 113665-84-2).rn,nm. or (clopidogrel* or clopilet or grepid or iscover or plavix or zopya or zylagren or zyllt or duocover or duoplavin).ti,ot,ab,sh,rn,hw,nm.
5	Aspirin/ or 50-78-2.rn,nm. or (acetylsalicylic acid* or ASA* or acenterine or acesal or acetan or acetard or aceticil or aceticyl or acetilum or acetonyl or acetilum or acetonyl or acetophen or acetosal or acetosalicylic acid or acetosalin or acetosalum or acetyl salicylate or acetyl salicylic acid or acetylin or acetylo or acetylon or acetylosalicylic acid or acetylsal or acetysal or acetylsalicyclic acid or acetylsalicyl or acetylsalicylate or acetylsalycic acid or acetylsalycylic acid or oracidulatum or actorin or Acylpyrin or acylpyrine or acytosal or adiro or alabunkun or alasil or albyl-e or alka seltzer or alkASA or Aloxiprimum or anacin or anasprin or anopyrin or ansin or anthrom or aptor or arthralgyl or asaflow or ascription or orasaphen or asapor or asatard or asawin or aspec or aspent or aspergum or aspex or aspilets or aspirem or aspirgran or ASAa or ASAe or ASAine or aspirisucre or aspisol or aspo cid or aspro or asrina or asrivo or asta or asteric or astrix or bebesan or biprin or bokey or boxazin or breoprin or bufferin or cafenol or caprin or cASA or catalgine or catalgix or cemerit or cemirit or claradin or claragine or Colfarit or comoprin or contrheuma or daga or darosal or Dispril or dispirin or dolean or dusil or Easprin or ecasil or Ecotrin or egalgic or emocin or empirin or encaprin or encine em or Endosprin or entaprin or entercin or enteroprin or enterosarine or enterospirine or entrophen or eskotrin or euthermine or extren or genasprin or globentyl or godamed or gotosan or helicon or idotyl or infatabs a or istopirin or istopyrine or ivepirine or juvepirine or keypo or kilios or kinderASA or Magnecyl or measurin or mejoral or melabon or Micristin or micropyrin or mikristin or miniasal or mycrisitin or naspro or novasen or nu seal or nu seals or ortho acetoxybenzoate or ortho acetoxybenzoic acid or ortho acetyloxybenzoate or ortho acetyloxybenzoic acid or ostoprin or pancemol or paracin or paynocil or pengo or plewin or Polopirin or polopiryna or premaspin or primaspan or proprin or pyronoval or reumyl or rhodine or rhonal or ronal or salacetin or salisalido or sargepirine or soldral or solpyron or Solprin or solucetyl or solupsa or Solupsan or spren or super tru or tapal or temagin or tevapirin or th 2152 or thrombo-aspilets or treupahlin or treuphalin or tromalyt or tromcor or turiital or verin or vitalink or xaxa or Zorprin).ti,ot,ab,sh,rn,hw,nm.
6	Warfarin/ or 81-81-2.rn,nm. or (adoisine or aldocumar or athrombin* or befarin or brumolin or carfin or circuvit or compound 42 or coumadan or coumadin or coumadine or coumafen or coumafene or coumefene or coumaphene or coumarins or dagonal or farin or jantoven or kumatox or maforan or marevan or orfarin or panwarfarin or panwarfin or prothromadin or simarc 2 or sofarin or tedicumar or tintorane or waran or warfant or warfar or warfarin or warfarine or warfil or warfilone or warnerin).ti,ot,ab,sh,rn,hw,nm.
7	Acenocoumarol/ or 152-72-7.rn,nm. or (acenocoumarol* or nicoumalone or acenocoumarin or acenocoumarine or acenocoumarole or acenocoumarolum or acenocumarol or acenocumarolo or acenocumerol or acenkumarin or acitrom or ascumar or neo sintrom or neosintrom or neositron or nicoumalone or nicumalon or nitrovarfarian or nitrowarfarin or sincoumar or sincumar or sinkumar or sinthrom or sinthrome or sintrom or sintroma or sintron or syncoumar or syncumar or synthrom or syntrom or syntroma or sintron or syncoumar or syncumar or syntrom or trombostop or zotil or sinthrome or synthrom or syncoumar or syncumar or sinkumar or sintrom or minisintrom or trombostop or zotil).ti,ot,ab,sh,rn,hw,nm.
8	or/1-7
9	Atrial fibrillation/
10	((atrial or atrium or auricular or heart) adj1 fibrillation*).ti,ab.
11	(a-fib or afib).ti,ab.
12	9 or 10 or 11
13	8 and 12
14	13 use pmez
15	13 use cctr
16	14 or 15
17	*dabigatran/ or (dabigatran* or Pradaxa or Pradax or Prazaxa or praxada or bibr 1048 or bibr1048 or bibr953 or bibr 953 or rendix).ti,ab.
18	*apixaban/ or (BMS 562247 or BMS562247 or apixaban* or Eliquis or Eliques).ti,ab.
19	*rivaroxaban/ or (rivaroxaban* or Xarelto or BAY 59-7939 or BAY59-7939 or BAY597939 or BAY 597939).ti,ab.
20	*clopidogrel/ or *acetylsalicylic acid plus clopidogrel/ or (clopidogrel* or clopilet or grepid or iscover or plavix or zopya or zylagren or zyllt or duocover or duoplavin).ti,ab.
21	*warfarin/ or (adoisine or aldocumar or athrombin* or befarin or brumolin or carfin or circuvit or compound 42 or coumadan or coumadin or coumadine or coumafen or coumafene or coumefene or

For peer review only

	coumaphene or coumarins or dagonal or farin or jantoven or kumatox or maforan or marevan or orfarin or panwarfarin or panwarfin or prothromadin or simarc 2 or sofarin or tedicumar or tintorane or waran or warfant or warfar or warfarin* or warfarine or warfil or warfilone or warnerin).ti,ab.
22	*acetylsalicylic acid/ or (acetylsalicylic acid* or ASA* or acenterine or acesal or acetan or acetard or aceticil or aceticyl or acetilum or acetonyl or acetilum or acetonyl or acetophen or acetosal or acetosalicylic acid or acetosalin or acetosalum or acetyl salicylate or acetyl salicylic acid or acetylin or acetylo or acetylon or acetylosalicylic acid or acetylsal or acetysal or acetylsalicyclic acid or acetylsalicyl or acetylsalicylate or acetylsalycic acid or acetylsalycylic acid or oracidulatum or actorin or Acylpyrin or acylpyrine or acytosal or adiro or alabunkun or alasil or albyl-e or alka seltzer or alkASA or Aloxiprimum or anasprin or anopyrin or ansin or anthrom or aptor or arthralgyl or asaflow or orasaphen or asapor or asatard or asawin or aspec or aspent or aspergum or aspex or aspilets or aspirem or aspirgran or ASAa or ASAe or ASAine or aspirisucre or aspisol or aspo cid or aspro or asrina or asrivo or asta or asteric or astrix or bebesan or biprin or bokey or boxazin or breoprin or bufferin or cafenol or caprin or cASA or catalgine or catalgix or cemerit or cemirit or claradin or claragine or Colfarit or comoprin or contrheuma or daga or darosal or Dispril or dispirin or dolean or dusil or Easprin or ecasil or Ecotrin or egalgic or emocin or empirin or encaprin or encine em or Endosprin or entaprin or entercin or enteroprin or enterosarine or enterospirine or entrophen or eskotrin or euthermine or extren or genasprin or globentyl or godamed or gotosan or helicon or idotyl or infatabs a or istopirin or istopyrine or ivepirine or juvepirine or keypo or kilios or kinderASA or Magnecyl or measurin or mejoral or melabon or Micristin or micropyrin or mikristin or miniasal or mycrisitin or naspro or novasen or nu seal or nu seals or ortho acetoxybenzoate or ortho acetoxybenzoic acid or ortho acetyloxybenzoate or ortho acetyloxybenzoic acid or ostoprin or pancemol or paracin or paynocil or pengo or plewin or Polopirin or polopiryna or premaspin or primaspan or proprin or pyronoval or reumyl or rhodine or rhonal or ronal or salacetin or salisalido or sargepirine or soldral or solpyron or Solprin or solucetyl or solupsa or Solupsan or spren or super tru or tapal or temagin or tevapirin or th 2152 or thrombo-aspilets or treupahlin or treuphalin or tromalyt or tromcor or turiital or verin or vitalink or xaxa or Zorprin).ti,ab.
23	*Acenocoumarol/ or (acenocoumarol* or nicoumalone or acenocoumarin or acenocoumarine or acenocoumarole or acenocoumarolum or acenocumarol or acenocumarolo or acenocumerol or acenkumarin or acitrom or ascumar or neo sintrom or neosintrom or neositron or nicoumalone or nicumalon or nitrovarfarian or nitrowarfarin or sincoumar or sincumar or sinkumar or sinthrom or sinthrome or sintrom or sintroma or sintron or syncoumar or syncumar or synthrom or syntrom or syntroma or sintron or syncoumar or syncumar or syntrom or trombostop or zotil or sinthrome or synthrom or syncoumar or syncumar or sinkumar or sintrom or minisintrom or trombostop or zotil).ti,ab.
24	or/17-23
25	Heart atrium fibrillation/
26	((atrial or atrium or auricular or heart) adj1 fibrillation*).ti,ab.
27	(a-fib or afib).ti,ab.
28	25 or 26 or 27
29	24 and 28
30	29 use oemezd
31	30 not conference abstract.pt.
32	16 or 31
33	32
34	limit 33 to english language [Limit not valid in CCTR; records were retained]
35	(Randomized Controlled Trial or Controlled Clinical Trial).pt.
36	Randomized Controlled Trial/
37	Randomized Controlled Trials as Topic/
38	"Randomized Controlled Trial (topic)"/
39	Controlled Clinical Trial/
40	Controlled Clinical Trials as Topic/
41	"Controlled Clinical Trial (topic)"/
42	Randomization/
43	Random Allocation/
44	Double-Blind Method/
45	Double Blind Procedure/

For peer review only

46	Double-Blind Studies/
47	Single-Blind Method/
48	Single Blind Procedure/
49	Single-Blind Studies/
50	Placebos/
51	Placebo/
52	Control Groups/
53	Control Group/
54	(random* or sham or placebo*).ti,ab,hw.
55	((singl* or doubl*) adj (blind* or dumm* or mask*)).ti,ab,hw.
56	((tripl* or trebl*) adj (blind* or dumm* or mask*)).ti,ab,hw.
57	(control* adj3 (study or studies or trial*)).ti,ab.
58	(Nonrandom* or non random* or non-random* or quasi-random* or quasirandom*).ti,ab,hw.
59	allocated.ti,ab,hw.
60	((open label or open-label) adj5 (study or studies or trial*)).ti,ab,hw.
61	or/35-60
62	34 and 61
63	limit 62 to yr = "1988 -Current"
64	remove duplicates from 63

**OTHER DATABASES**	
PubMed	Same MeSH, keywords, limits, and study types used as per MEDLINE search, with appropriate syntax used.

Grey Literature

Dates for Search:	**June 12, 2012 – July 8, 2012; updated January 2014**
**Keywords:**	dabigatran, Pradax, Pradaxa, apixaban, Eliquis, rivaroxaban, Xarelto, clopidogrel, Plavix, acetylsalicylic acid, Aspirin, warfarin, Coumadin, acenocoumarol, Sintrom, Sinthrome<br>Atrial fibrillation
**Limits:**	Publication years 1988-present, English

Relevant items from the following sections of the CADTH grey literature checklist, “Grey matters: a practical tool for evidence-based searching” (http://www.cadth.ca/resources/grey-matters) were searched:

- Health Technology Assessment Agencies
- Health Economic
- Clinical Practice Guidelines
- Databases (free)

Internet Search

For peer review only

Appendix 2: PRISMA Diagram

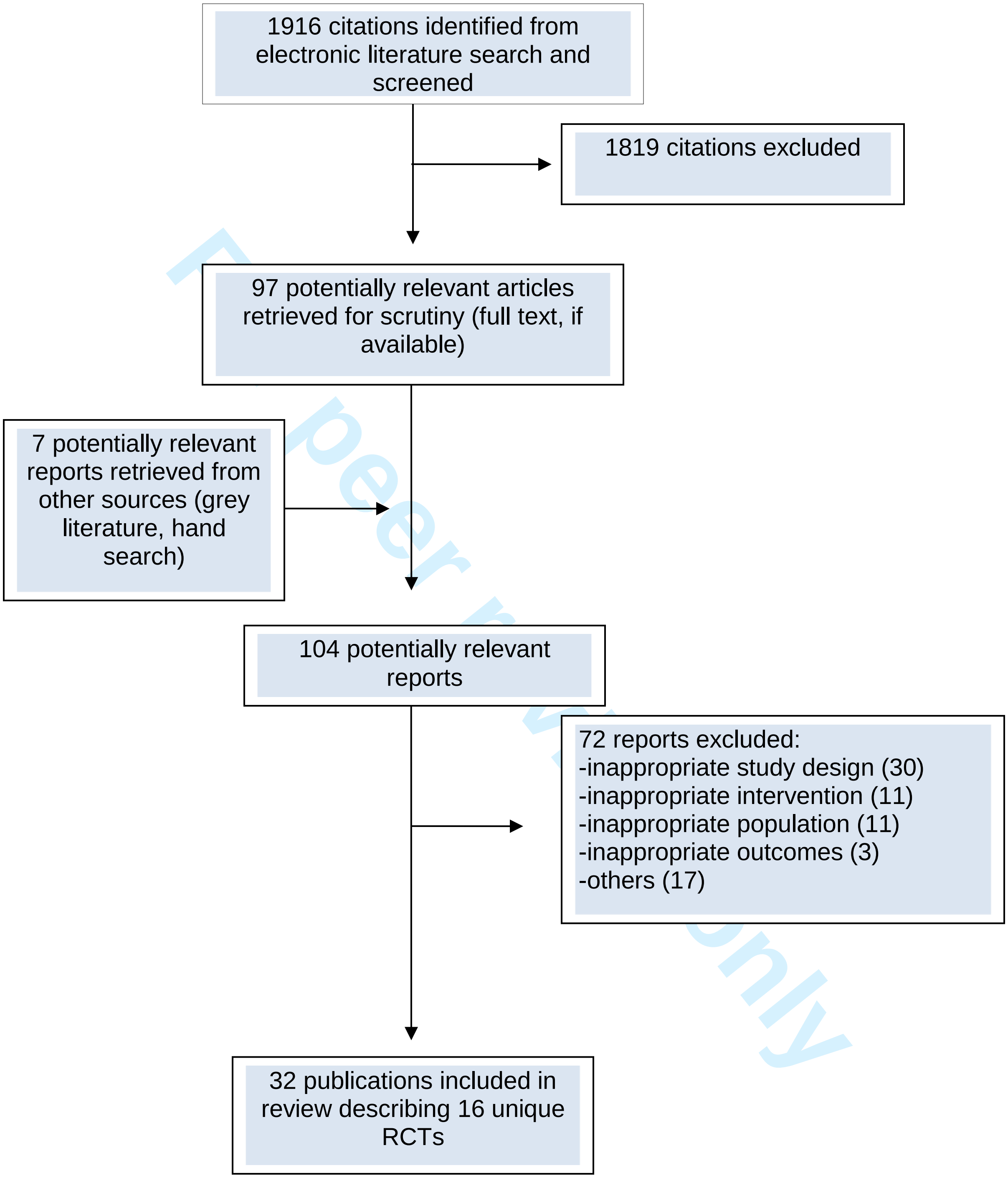

Appendix 3: Summary of Patient Baseline Characteristics

Study	Baseline Characteristics															Centre above TTR Throughout follow-up		
	Age, Mean ± SD (y)	Age Category (%)		Sex (%)		$CHADS_2$, Mean ± SD	$CHADS_2$ Category (%)			Prior Stroke /TIA (%)	DM (%)	Heart Failure (%)	HBP (%)	MI (%)	VKA Naïve (%)	Mean TTR (%)	TTR Category (%)	
		≤75y	>75 y	M	F		0	1	≥2								≤66%	>66%
ACTIVE-W n=6706	70 ± NR	NR	NR	66	34	2 ± 1.1	NR	NR	NR	15	21	30	82	17	23	64	48	52
AFASAK n=672	75 ± NR	NR	NR	54	46	NR ± NR	NR	NR	NR	6	9	53	32	7	NR	N/A	N/A	N/A
AFASAK-2 n=339	73 ± NR	NR	NR	61	39	NR ± NR	NR	NR	NR	8	12	70	45	7	NR	NR	NR	NR
ARISTOTLE n=18201	70 ± NR	69	31	65	35	2.1 ± 1.1	0	34	66	19	25	35	87	14	43	62	NR	NR
ARISTOTLE-J n=222	70 ± NR	69	31	83	17	1.9 ± NR	1	42	57	28	23	1	83	NR	16	60	NR	NR
BAFTA n=973	82 ± 4	0	100	55	45	NR ± NR	NR	NR	NR	13	13	20	54	11	NR	67	NR	NR
CAFA n=378	68 ± NR	NR	NR	75	25	NR ± NR	NR	NR	NR	4	12	22	39	13	NR	44	NR	NR
Chung et al 2011 n=234	65 ± 9	87	13	65	35	1.9 ± 1.1	0	47	53	24	30	29	71	NR	48	45	NR	NR
ENGAGE AF-TIMI 48 n=21105	72 ± NR	60	40	62	38	2.8 ± 1.0	0	0	100	28	36	57	94	12	41	65	52	48
JAST n=871	65 ± NR	NR	NR	70	30	NR ± NR	NR	NR	NR	3	14	9	39	NR	92	N/A	N/A	N/A
PETRO n=236	70 ± NR	70	30	82	18	NR ± NR	NR	NR	NR	18	25	32	71	NR	0	57	NR	NR
RE-LY n=18113	71 ± NR	60	40	64	36	2.1 ± NR	32		68	20	20	23	32	79	17	64	50	50
ROCKET-AF n=14264	73 ± NR	NR	NR	60	40	3.5 ± NR	0	0	100	55	40	62	91	17	38	55	NR	NR
WASPO n=75	83 ± NR	0	100	47	53	NR ± NR	NR	NR	NR	NR	4	NR	47	NR	NR	69	NR	NR
Weitz et al 2010 n=719	65 ± 9	NR	NR	62	38	NR ± NR	0	0	100	NR	NR	NR	NR	NR	66	50	NR	NR

For peer review only - http://bmjopen.bmj.com/site/about/guidelines.xhtml

Study	Baseline Characteristics															Centre above TTR Throughout follow-up		
	Age, Mean ± SD (y)	Age Category (%)		Sex (%)		CHADS$_2$, Mean ± SD	CHADS$_2$ Category (%)			Prior Stroke /TIA (%)	DM (%)	Heart Failure (%)	HBP (%)	MI (%)	VKA Naïve (%)	Mean TTR (%)	TTR Category (%)	
		≤75y	>75 y	M	F		0	1	≥2								≤66%	>66%
Yamashita et al 2012 n=385	69 ± NR	71	29	83	17	2.1 ± NR	0	100		28	23	27	73	NR	15	73% FOR <70Y; 83% FOR ≥70Y;	NR	NR

DM = diabetes mellitus; F = female sex; HBP = high blood pressure; M = male sex; MI = myocardial infarction; N/A = not applicable; NR = not reported; SD = standard deviation; TIA = transient ischemic attack; TTR = time in therapeutic range; VKA = vitamin K antagonist; y = year.

Note: Only data from the included relevant treatment arms have been extracted.

For peer review only

Appendix 4: Summary of Included Trials

Study and Design	Disposition	Population	Interventions	Follow-up	Primary Outcome(s)
**ACTIVE-W**<br>OL RCT, 2006.<br>Multicenter, 32 countries (including Europe / America).	Randomized: n=6706	AF patients eligible / willing to take anticoagulant therapy, with ≥ 1 pre-specified risk factor for stroke.	**Clopidogrel / Low-dose ASA** (n=3335)<br>**Warfarin – INR 2.0 to 3.0** (n=3371)	1.3 years (stopped early)	First occurrence of stroke, non-CNS SE, MI or vascular death.
**AFASAK**<br>OL/DB RCT, 1989.<br>Multicenter, Copenhagen.	Randomized: n=1007	AF patients, with no recent anticoagulation or cerebrovascular event, valve replacement or rheumatic heart disease.	***OL Warfarin – INR 2.8 to 4.2 **** *(n=335)*<br>**Low-dose ASA** (n=336)<br>**PL** (n=336)	2 years	Thromboembolic complication: stroke, TIA or embolic complication to viscera / extremities.
**AFASAK-2**<br>OL RCT, 1998.<br>Multicenter, Copenhagen.	Randomized: n=677	AF patients ≥ 60 years (unless with CV or pulmonary disease), without recent TIA or risk factors for bleeding.	***Fixed-dose Warfarin **** *(n=167)*<br>***Fixed-dose Warfarin / Medium-dose ASA **** *(n=171)*<br>**Medium Dose ASA** (n=169)<br>**Warfarin – INR 2.0 to 3.0** (n=170)	3.5 years	Stroke or SE.
**ARISTOTLE**<br>DB RCT, 2011.<br>Multicenter, 39 countries (including Europe / America).	Randomized: n=18201	AF patients with ≥ 1 risk factor for stroke, without recent stroke or ASA > 165 mg.	**Apixaban 5 mg BID** (n=9120)<br>**Warfarin – INR 2.0 to 3.0** (n=9081)<br>Concomitant use of **ASA** (≤ 165 mg daily) or **NSAIDs** permitted.	NR	Stroke or SE;<br>Major bleeding.
**ARISTOTLE-J**<br>OL/DB RCT, 2011.<br>Multicenter, Japan.	Randomized: n=222	AF patients with ≥ 1 risk factor for stroke, without recent TIA or ASA > 100 mg.	**Apixaban 2.5 mg BID** (n=74)<br>**Apixaban 5 mg BID** (n=74)<br>**OL Warfarin – INR 2.0 to 3.0** (n=74)<br>Concomitant use of **ASA** (≤ 100 mg daily) permitted.	12 weeks	Composite of major bleeding and CRNM bleeding events.
**BAFTA**<br>OL RCT, 2007.<br>Multicenter, UK.	Randomized: n=973	AF patients ≥ 75 years, with clinical uncertainty around optimal treatment.	**Low-dose ASA** (n=485)<br>**Warfarin – INR 2.0 to 3.0** (n=488)	2.7 years	Disabling stroke, ICH and other clinically significant arterial embolism.
**CAFA**<br>DB RCT, 1991.<br>Multicenter, Canada.	Randomized: n=378	AF patients without recent stroke, TIA or MI, and who did not require antiplatelet drug therapy.	**Warfarin – INR 2.0 to 3.0** (n=187)<br>**PL** (n=191)	1.2 years (stopped early)	Ischemic stroke, SE, ICH or fatal hemorrhage.

For peer review only

Study and Design	Disposition	Population	Interventions	Follow-up	Primary Outcome(s)
**Chung et al 2011**<br>OL RCT, 2011.<br>Multicenter, 4 countries (including Asia).	Randomized: n=234	AF patients at moderate to high risk of stroke (CHADS2 score ≥ 1).	**Edoxaban 30 mg OD** (n=79)<br>**Edoxaban 60 mg OD** (n=80)<br>**Warfarin – INR 2.0 to 3.0** (n=75)	12 weeks	All bleeding events
**ENGAGE AF-TIMI 48**<br>DB RCT, 2013.<br>Multicenter, 46 countries (including Europe / America).	Randomized: n=21105	AF patients at moderate to high risk of stroke (CHADS2 score ≥ 2).	**Edoxaban 30 mg OD** (n=7034)<br>**Edoxaban 60 mg OD** (n=7035)<br>**Warfarin – INR 2.0 to 3.0** (n=7036)<br>Almost a third of patients in treatment arms took concomitant ASA.	2.8 years	Stroke or SE and major haemorrhage.
**JAST**<br>OL RCT, 2006.<br>Multicenter, Japan.	Randomized: n=871	AF patients without recent symptomatic thromboembolic disease, ICH or GI bleeding.	**Medium-dose ASA** (n=426)<br>**No treatment** (n=445)	2.1 years (stopped early)	CV death, symptomatic brain infarction, or TIA.
**PETRO**<br>OL/DB RCT, 2007.<br>Multicenter, 4 countries (Europe and USA).	Randomized: n=502	AF patients with ≥ 1 risk factor for stroke and with coronary artery disease (removed halfway to facilitate recruitment). No recent MI, stroke, TIA or major bleed. Warfarin pre-treatment required for ≥ 8 weeks.	***Dabigatran 50 mg BID ± Low-dose or High-dose ASA **** *(n=105)*<br>***Dabigatran 300 mg BID ± Low-dose or High-dose ASA **** *(n=161)*<br>**Dabigatran 150 mg BID ± Low-dose or High-dose ASA** † (n=166)<br>**OL Warfarin – INR 2.0 to 3.0** (n=70)	12 weeks	Thromboembolic events and bleeding events.
**RE-LY**<br>OL/DB RCT, 2009.<br>Multicenter, 44 countries (including Europe / America).	Randomized: n=18113	AF patients with ≥ 1 risk factor for stroke, without recent stroke or increased risk of bleeding.	**Dabigatran 110 mg BID** (n=6015)<br>**Dabigatran 150 mg BID** (n=6076)<br>**OL Warfarin – INR 2.0 to 3.0** (n=6022)<br>Concomitant use of **ASA** (<100 mg daily) or other **antiplatelet agents** permitted.	2 years	Stroke or SE and major haemorrhage.
**ROCKET-AF**<br>DB RCT, 2011.<br>Multicenter, 45 countries (including Europe / America).	Randomized: n=14264	AF patients at moderate to high risk of stroke (CHADS2 score ≥ 2).	**Rivaroxaban 20 mg OD** (n=7131)<br>**Warfarin – INR 2.0 to 3.0** (n=7133)<br>Over a third of patients in both arms took concomitant ASA.	1.9 years	Composite of stroke and SE;<br>Composite of major and NMCR bleeding.

For peer review only - http://bmjopen.bmj.com/site/about/guidelines.xhtml

Study and Design	Disposition	Population	Interventions	Follow-up	Primary Outcome(s)
**WASPO**<br>OL RCT, 2007.<br>Multicenter, UK.	Randomized: n=75	Ambulant AF patients > 80 and < 90 years with Folstein score > 25, without previous fall, ICH or GI bleeding. Patients with prior stroke or TIA were excluded.	**Warfarin – INR 2.0 to 3.0** (n=36)<br>**Medium-dose ASA** (n=39)	1 year	Combined endpoints: death; stroke, TIA or SE; serious bleeding; withdrawal.
**Weitz et al 2010**<br>DB RCT, 2010.<br>Multicenter, 12 countries (including Eastern Europe / America).	Randomized: n=719	AF patients at moderate to high risk of stroke (CHADS2 score ≥ 2).	**Edoxaban 30 mg OD** (n=235)<br>**Edoxaban 60 mg OD** (n=234)<br>**Warfarin – INR 2.0 to 3.0** (n=250)	12 weeks	Bleeding and abnormalities of hepatic function
**Yamashita et al 2012**<br>DB RCT, 2012.<br>Multicenter, 1 country (Japan).	Randomized: n=385	AF patients at moderate to high risk of stroke (CHADS2 score ≥ 1).	**Edoxaban 30 mg OD** (n=130)<br>**Edoxaban 60 mg OD** (n=130)<br>**Warfarin – INR 2.0 to 3.0** (n=125)	12 weeks	All bleeding events

AF = atrial fibrillation; **ASA =** acetylsalicylic acid; **CNS =** central nervous system; **CRNM =** clinically relevant non-major bleeding; **CV =** cardiovascular; **DB =** double-blind; **GI =** gastrointestinal; **ICH =** intracerebral hemorrhage; **MI =** myocardial infarction; **NR =** not reported; **NSAIDs =** non-steroidal anti-inflammatory drugs; **OL =** open label; **PL =** placebo; **RCT =** randomized controlled trial; **SE =** systemic embolism; **TIA =** transient ischemic attack; **UK =** United-Kingdom; **VKA =** vitamin K antagonist.

* No data was extracted from these treatment arms since they did not meet the inclusion criteria specified in the protocol. These trials were nevertheless included in the systematic review because they had at least one relevant comparison between two interventions of interest.

† Data was extracted only for dabigatran without ASA (n=100).

For peer review only

Appendix 5: Evidence network for major bleeding. The width of the lines is proportional to the number of randomized controlled trials (RCTs) comparing each pair of treatments, and the size of each treatment node is proportional to the number of randomised participants (sample size). A dotted line indicates a three-arm RCT and a black node indicates a node included in the analysis but not reported in the main text.

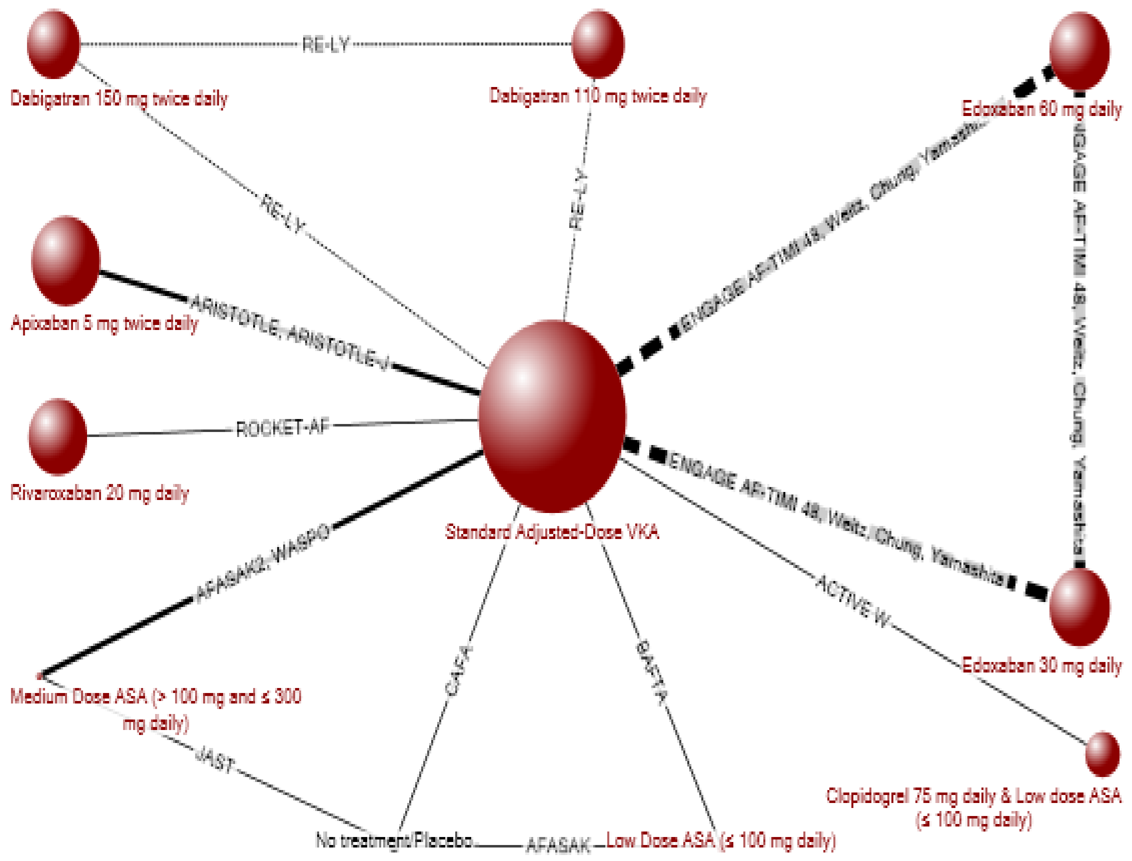

mg= milligram; RCT = randomized controlled trial

Appendix 6: Summary of results from Network Meta-analysis for All-cause Stroke or Systemic Embolism

Treatment	Pair-wise Meta-Analysis, OR (95% CI) versus standard adjusted dose VKA	NMA, Odds Ratio (95% CrI) versus standard adjusted dose VKA	NMA, Absolute Risk Difference per 1,000 treated (95% CrI) versus standard adjusted dose VKA	Probability Best (%) for Outcome	Rank for Outcome	SUCRA
Dabigatran 150 mg twice daily	0.66(0.53,0.82)	0.66 (0.53,0.82)	6 fewer (8 fewer-3 fewer)	85.2%	1.2	98.1%
Apixaban 5 mg twice daily	0.79(0.66,0.94)	0.78 (0.65,0.94)	4 fewer (6 fewer-1 fewer)	11.2%	2.5	85.4%
Edoxaban 60 mg daily	0.87(0.71,1.05)	0.87 (0.74,1.01)	3 fewer (5 fewer-1 more)	1.2%	3.7	72.8%
Rivaroxaban 20 mg daily	0.88(0.74,1.05)	0.87 (0.74,1.03)	3 fewer (5 fewer-1 more)	1.0%	3.9	71.2%
Dabigatran 110 mg twice daily	0.91(0.75,1.11)	0.91 (0.74,1.12)	2 fewer (5 fewer-2 more)	0.1%	4.5	64.7%
Standard Adjusted-Dose VKA	NA	NA	NA	0.0%	6.0	50.2%
Edoxaban 30 mg daily	1.14(0.94,1.37)	1.14 (0.98,1.32)	3 more (1 fewer-6 more)	0.0%	7.4	36.5%
Medium Dose Aspirin (> 100 mg and ≤ 300 mg daily)	1.21(0.51,2.87)	1.35 (0.74,2.47)	6 more (5 fewer-24 more)	1.2%	7.8	32.0%
No treatment/Placebo	1.84(0.66,5.1)	1.53 (0.9,2.63)	9 more (2 fewer-26 more)	0.1%	8.9	21.5%
Low Dose Aspirin (≤ 100 mg daily)	2.03(1.32,3.12)	1.87 (1.26,2.8)	14 more (5 more-29 more)	0.0%	10.1	9.4%
Clopidogrel 75 mg OD & Low dose Aspirin (≤ 100 mg daily)	1.93(1.41,2.64)	1.93 (1.42,2.65)	15 more (7 more-26 more)	0.0%	10.2	8.3%

CI = confidence interval; CrI = credible interval; OR= odds ratio

Bayesian network meta-analysis also allows the analyst to estimate the probability that a treatment is best, second best, and so on for a particular outcome. A simple numerical summary of these probabilities – the surface under the cumulative ranking (SUCRA) – for each treatment can also be calculated [50]. The SUCRA would be 100% when a treatment is certain to be the best and 0% when a treatment is certain to be the worst. SUCRA values enable the ranking of treatments overall for a particular outcome. For example, for Dabigatran 150 mg twice daily for stroke/SE the SUCRA value for is 98.1%, which is better than other treatments. By contrast, Clopidogrel 75 mg OD & Low dose Aspirin (≤ 100 mg daily) has a SUCRA of 8.3% for stroke/SE meaning it is certain to be worst for this outcome among active treatments.

For peer review only

Appendix 7: Summary of results from Network Meta-analysis for Major Bleeding

Treatment	Pair-wise Meta-Analysis, OR (95% CI) versus standard adjusted dose VKA	NMA, Odds Ratio (95% CrI) versus standard adjusted dose VKA	NMA, Absolute Risk Difference per 1,000 treated (95% CrI) versus standard adjusted dose VKA	Probability Best (%) for Outcome	Rank for Outcome	SUCRA
Edoxaban 30 mg daily	0.46(0.39,0.55)	0.46(0.4,0.54)	18 fewer (20 fewer-15 fewer)	29.7%	1.7	92.9%
No treatment/Placebo	0.39(0.08,2.02)	0.33(0.08,1.08)	22 fewer (31 fewer-3 more)	70.2%	1.7	92.6%
Apixaban 5 mg twice daily	0.69(0.60,0.80)	0.69(0.6,0.8)	10 fewer (13 fewer-7 fewer)	0.0%	3.2	78.1%
Edoxaban 60 mg daily	0.78(0.67,0.92)	0.79(0.69,0.9)	7 fewer (10 fewer-4 fewer)	0.0%	4.5	64.6%
Dabigatran 110 mg twice daily	0.8(0.68,0.94)	0.8(0.69,0.93)	7 fewer (10 fewer-3 fewer)	0.0%	4.7	62.7%
Dabigatran 150 mg twice daily	0.93(0.81,1.07)	0.93(0.8,1.08)	3 fewer (7 fewer-3 more)	0.0%	6.8	41.7%
Low Dose Aspirin (≤ 100 mg daily)	1.01(0.57,1.78)	1.05(0.6,1.86)	2 more (13 fewer-26 more)	0.1%	7.9	30.6%
Standard Adjusted-Dose VKA	NA	NA	NA	0.0%	8.0	30.2%
Rivaroxaban 20 mg daily	1.03(0.9,1.18)	1.03(0.89,1.18)	1 more (4 fewer-6 more)	0.0%	8.4	26.3%
Clopidogrel 75 mg OD & Low dose Aspirin (≤ 100 mg daily)	1.1(0.82,1.48)	1.1(0.83,1.47)	4 more (6 fewer-15 more)	0.0%	8.9	20.8%
Medium Dose Aspirin (> 100 mg and ≤ 300 mg daily)	1.68(0.50,5.69)	1.79(0.63,5.67)	24 more (12 fewer-120 more)	0.0%	10.0	9.8%

CI = confidence interval; CrI = credible interval; OR= odds ratio

Bayesian network meta-analysis also allows the analyst to estimate the probability that a treatment is best, second best, and so on for a particular outcome. A simple numerical summary of these probabilities – the surface under the cumulative ranking (SUCRA) – for each treatment can also be calculated [50]. The SUCRA would be 100% when a treatment is certain to be the best and 0% when a treatment is certain to be the worst. SUCRA values enable the ranking of treatments overall for a particular outcome. For example, for major bleeding the SUCRA value for Edoxaban 60 mg is 92.8%, which is better than other treatments. By contrast, Medium Dose Aspirin (> 100 mg and ≤ 300 mg daily) has a SUCRA of 9.9% for major bleeding meaning it is certain to be worst for this outcome.

For peer review only

Appendix 8: Benefit-Harm plot for each treatment (absolute harm difference per 1,000 patients treated). The absolute risk difference of recurrence of Stroke or systemic embolism relative to standard adjusted dose VKA is presented on the x-axis, while the absolute risk difference of major bleeding relative to standard adjusted dose VKA is on the y-axis. The dot is representative of the median estimate for each treatment while the 95% credible intervals are presented as error bars.

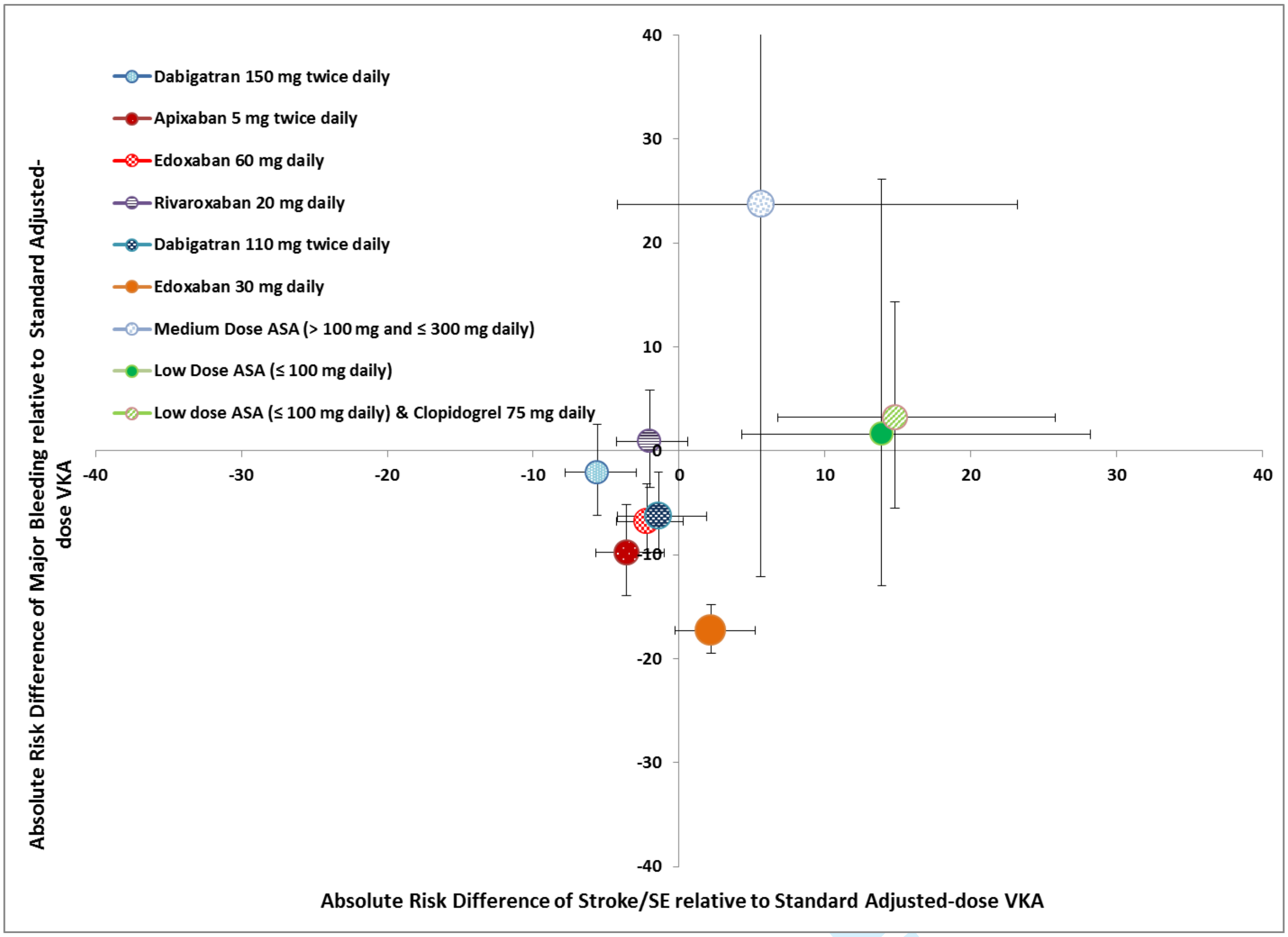

Appendix 9: Rationale of choice of fixed-effects model and limitations

It is challenging to conduct network meta-analyses when evidence structures are comprised of single study connections, especially when there is obvious heterogeneity in the network. The use of a random-effects model with a vague prior on the between study variance exerts a large degree of influence on the credible intervals (CrI's) because there is insufficient studies to reign in the prior and provide an accurate estimate of the between study variance. As a result, results often appear unrealistic (e.g., wide credible intervals for a treatment that was shown to be associated with a statistically significant decrease). Accordingly, we also considered informative priors on the between study variance from the recently published reports.[11,44] However, even when these priors were applied on the between study variance, they still did not reign in CrI's effect estimates again because the network was comprised of single study connections. The use of the random effects model with either a vague or informative prior was problematic because treatments that were originally associated with statistically significant reductions in stroke or SE relative to standard adjusted dose VKA were no longer associated with significant reductions, limiting external validity of the model (see Figures below on direct estimates in Online Appendix 4 and 5). While we could have opted to use less vague priors (e.g., ¼ of prior in Turner et al[11]), the choice of the prior would have seemed somewhat arbitrary because it was not based on any empirical evidence. As such, we opted to use the fixed-effects model despite the known issues in doing so. By using the fixed-effects model, treatments that were associated with significant reductions in the risk of stroke or SE relative to standard adjusted dose VKA retained significance. We felt that it would have been inappropriate to remove statistical significance by applying vague priors given the large investment involved in developing clinical trials. However, a consequence of retaining statistical significance is seen when comparing newer oral anticoagulants versus one another in Figure 3. Because we assumed the new oral anticoagulants were all associated with reductions in Stroke or SE relative to standard adjusted dose VKA (if that was the case in the underlying trials), significant reductions in the risk of stroke or SE among new oral anticoagulants are observed in Figure 5. This may or may not be the case in clinical practice. As noted in tables 1 and Online Appendix 2, there is considerable heterogeneity across studies. However, the network structure itself (i.e., single study connections), made it difficult to properly adjust for differences in patient and study characteristics using standard approaches. Accordingly, results in Figure 3 should be interpreted with caution. We conducted simple sub-group analyses (breaking randomization) to help adjust for this heterogeneity, which is a very crude way to adjust for heterogeneity. To fully elucidate the comparative benefits and harms of antithrombotic agents, especially across the various sub-populations, rigorously conducted comparative studies, or network meta-regression analyses of patient-level data are required.

For peer review only

Forest Plot – Odds ratio (95% Credible interval) when using informative prior on the sd (i.e, dlnorm(-3.02,0.29)[11]

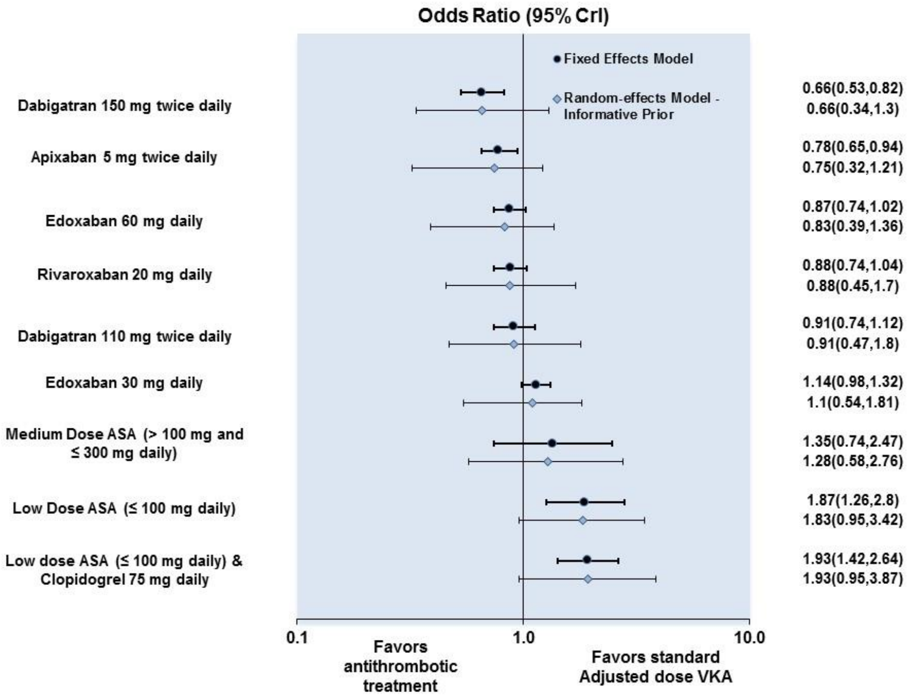

Forest Plot – Odds ratio (95% Credible interval) when using vague prior on the sd (i.e, duniform(0,2))

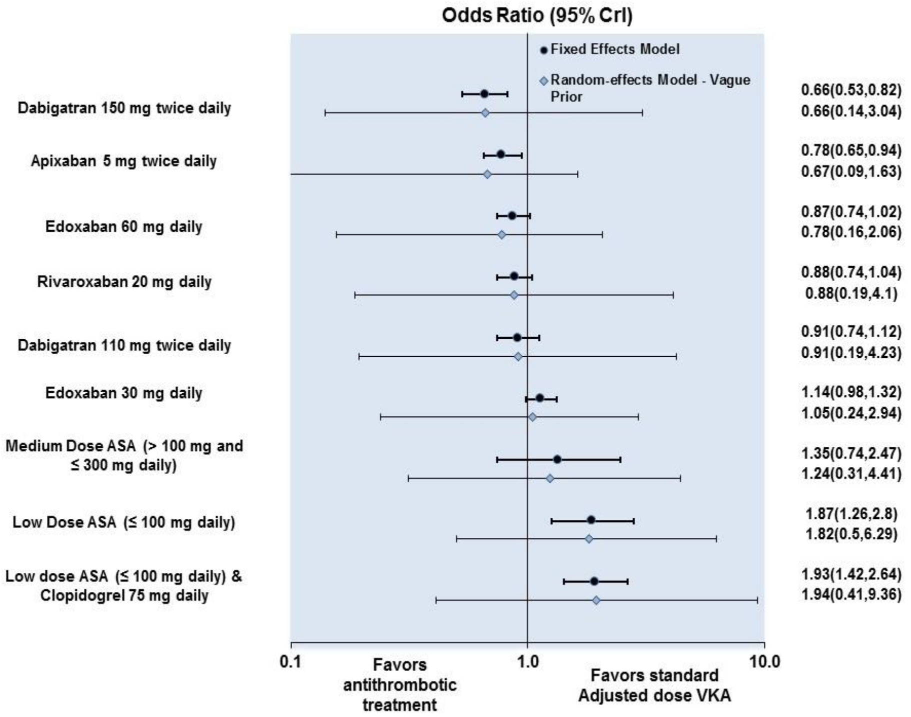

Appendix 10: PRISMA Checklist

Section/topic	#	Checklist item	Reported on page #
**TITLE**			
Title	1	Identify the report as a systematic review, meta-analysis, or both.	0
**ABSTRACT**			
Structured summary	2	Provide a structured summary including, as applicable: background; objectives; data sources; study eligibility criteria, participants, and interventions; study appraisal and synthesis methods; results; limitations; conclusions and implications of key findings; systematic review registration number.	0
**INTRODUCTION**			
Rationale	3	Describe the rationale for the review in the context of what is already known.	1
Objectives	4	Provide an explicit statement of questions being addressed with reference to participants, interventions, comparisons, outcomes, and study design (PICOS).	1
**METHODS**			
Protocol and registration	5	Indicate if a review protocol exists, if and where it can be accessed (e.g., Web address), and, if available, provide registration information including registration number.	2
Eligibility criteria	6	Specify study characteristics (e.g., PICOS, length of follow-up) and report characteristics (e.g., years considered, language, publication status) used as criteria for eligibility, giving rationale.	2-3
Information sources	7	Describe all information sources (e.g., databases with dates of coverage, contact with study authors to identify additional studies) in the search and date last searched.	2-3
Search	8	Present full electronic search strategy for at least one database, including any limits used, such that it could be repeated.	2-3
Study selection	9	State the process for selecting studies (i.e., screening, eligibility, included in systematic review, and, if applicable, included in the meta-analysis).	3
Data collection process	10	Describe method of data extraction from reports (e.g., piloted forms, independently, in duplicate) and any processes for obtaining and confirming data from investigators.	3
Data items	11	List and define all variables for which data were sought (e.g., PICOS, funding sources) and any assumptions and simplifications made.	3-4
Risk of bias in individual studies	12	Describe methods used for assessing risk of bias of individual studies (including specification of whether this was done at the study or outcome level), and how this information is to be used in any data synthesis.	3,6
Summary measures	13	State the principal summary measures (e.g., risk ratio, difference in means).	3
Synthesis of results	14	Describe the methods of handling data and combining results of studies, if done, including measures of consistency (e.g., I^2) for each meta-analysis.	4-5
Risk of bias across studies	15	Specify any assessment of risk of bias that may affect the cumulative evidence (e.g., publication bias, selective reporting within studies).	5
Additional analyses	16	Describe methods of additional analyses (e.g., sensitivity or subgroup analyses, meta-regression), if done, indicating which were pre-specified.	5
**RESULTS**			

For peer review only

Study selection	17	Give numbers of studies screened, assessed for eligibility, and included in the review, with reasons for exclusions at each stage, ideally with a flow diagram.	6, Online Appendix 2
Study characteristics	18	For each study, present characteristics for which data were extracted (e.g., study size, PICOS, follow-up period) and provide the citations.	5-6, Online Appendix 3
Risk of bias within studies	19	Present data on risk of bias of each study and, if available, any outcome level assessment (see item 12).	7
Results of individual studies	20	For all outcomes considered (benefits or harms), present, for each study: (a) simple summary data for each intervention group (b) effect estimates and confidence intervals, ideally with a forest plot.	Figure 2-4
Synthesis of results	21	Present results of each meta-analysis done, including confidence intervals and measures of consistency.	Figure 2-4, Online Appendix 6,7 and 9
Risk of bias across studies	22	Present results of any assessment of risk of bias across studies (see Item 15).	7
Additional analysis	23	Give results of additional analyses, if done (e.g., sensitivity or subgroup analyses, meta-regression [see Item 16]).	Table 1, Online Appendix 12
**DISCUSSION**			
Summary of evidence	24	Summarize the main findings including the strength of evidence for each main outcome; consider their relevance to key groups (e.g., healthcare providers, users, and policy makers).	11-12
Limitations	25	Discuss limitations at study and outcome level (e.g., risk of bias), and at review-level (e.g., incomplete retrieval of identified research, reporting bias).	14-15
Conclusions	26	Provide a general interpretation of the results in the context of other evidence, and implications for future research.	10-16
**FUNDING**			
Funding	27	Describe sources of funding for the systematic review and other support (e.g., supply of data); role of funders for the systematic review.	0

For peer review only

Appendix 11: Assessment of Inconsistency

We plotted the posterior mean deviance of the individual data points in the inconsistency model against their posterior mean deviance in the consistency model to help identify loops where inconsistency is present. In our analysis, the posterior mean deviance contributions are very similar and close to 1, for both models. The consistency model has a similar posterior mean of the residual deviance (29.41 vs. 29.37) and DIC (195.00 vs. 196.87) and hence is a similar fit for both models. A few points show a lower value of the posterior mean deviance in the inconsistency model but these values are not too far from 1. Similarly, a few points have higher values of the posterior mean deviance in the consistency model (again with values not too far from 1) suggesting that each model may fit the data points better for certain points. The parameter estimates are also similar for both models and there is considerable overlap in the 95% credible intervals (see table below), suggesting no evidence of inconsistency in the network.

Plot of posterior mean deviance of the individual data points in the inconsistency model against their posterior mean deviance in the consistency model

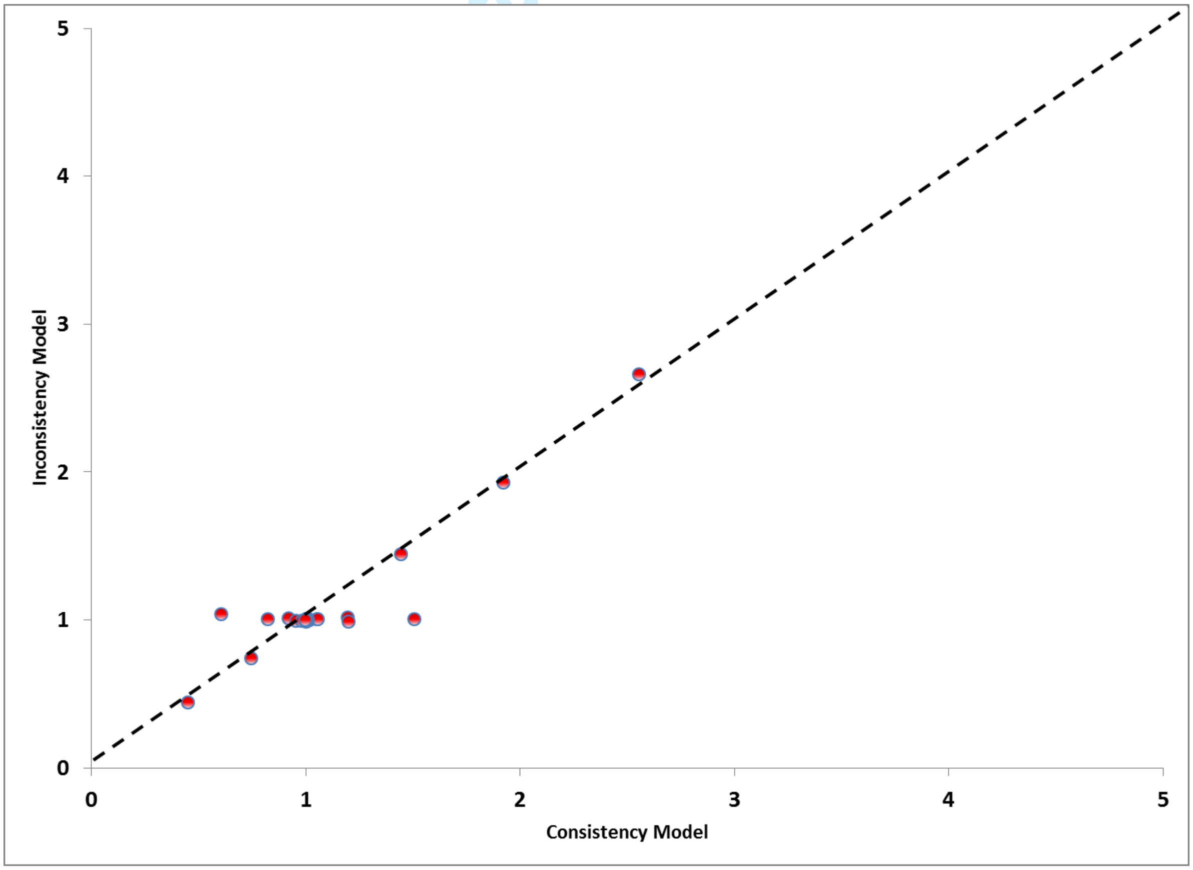

Comparison of effect estimates from consistency and inconsistency models where there is data available for comparison

Comparison	Consistency Model	Inconsistency Model
Dabigatran 150 mg twice daily vs Standard Adjusted-Dose VKA	0.66 (0.53,0.82)	0.66 (0.53,0.82)
Apixaban 5 mg twice daily vs Standard Adjusted-Dose VKA	0.78 (0.65,0.94)	0.78 (0.65,0.94)
Edoxaban 60 mg daily vs Standard Adjusted-Dose VKA	0.87 (0.74,1.01)	0.87 (0.74,1.01)
Rivaroxaban 20 mg daily vs Standard Adjusted-Dose VKA	0.87 (0.74,1.03)	0.88 (0.74,1.03)
Dabigatran 110 mg twice daily vs Apixaban 5 mg twice daily vs	0.91 (0.74,1.12)	0.91 (0.74,1.12)
Edoxaban 30 mg daily vs Standard Adjusted-Dose VKA	1.14 (0.98,1.32)	1.14 (0.98,1.32)
Medium Dose Aspirin (> 100 mg and ≤ 300 mg daily) vs Standard Adjusted-Dose VKA	1.35 (0.74,2.47)	0.82 (0.33,1.98)
Low Dose Aspirin (≤ 100 mg daily) vs Standard Adjusted-Dose VKA	1.87 (1.26,2.8)	2.04 (1.33,3.19)
Clopidogrel 75 mg OD & Low dose Aspirin (≤ 100 mg daily) vs Standard Adjusted-Dose VKA	1.93 (1.42,2.64)	1.93 (1.42,2.65)
No treatment/Placebo vs Low Dose Aspirin (≤ 100 mg daily)	0.82 (0.48,1.38)	1 (0.51,1.98)
Residual deviance	29.41	29.37
DIC	195.00	196.87

For peer review only

Appendix 12: Sensitivity analysis with AVERROES and ACTIVE A

A. Stroke or SE

Evidence Networks for Stroke or SE NMA including AVEROES and ACTIVE A studies

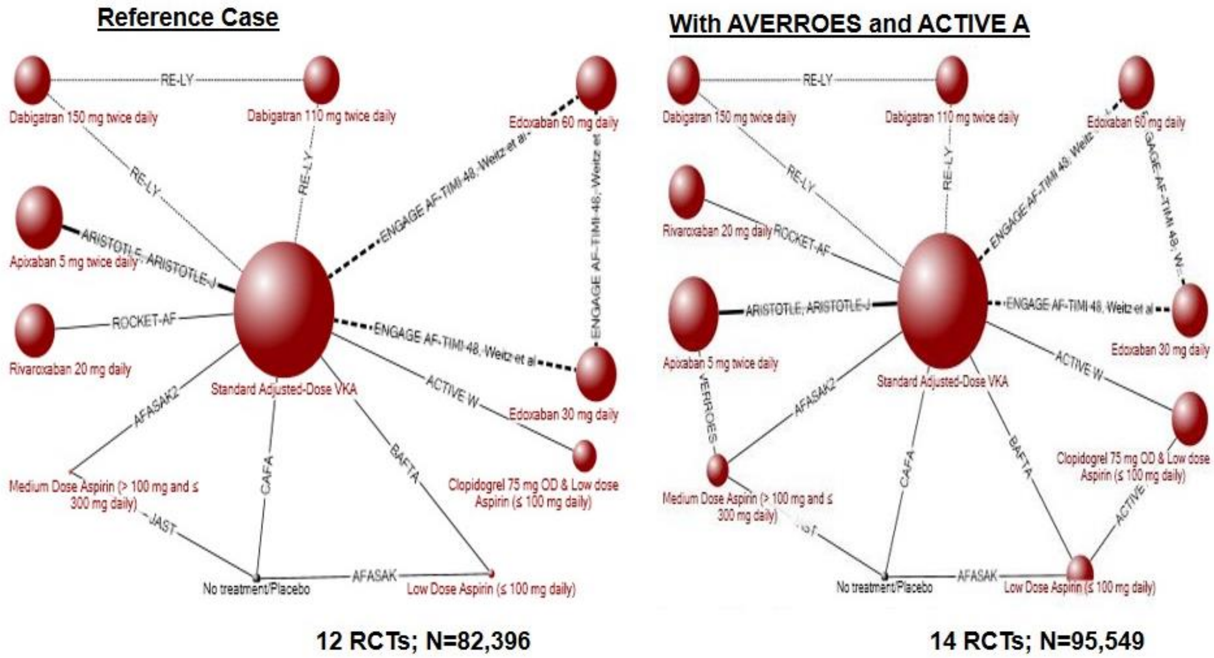

Forest Plot for Stroke or SE NMA including AVERROES and ACTIVE A studies

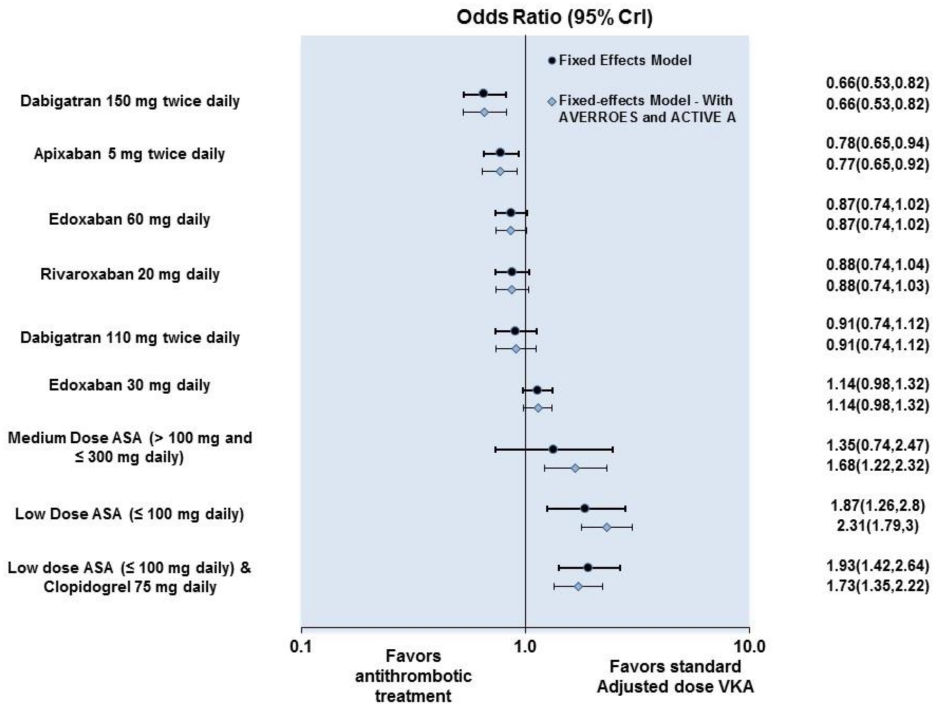

B. Major Bleeding

Evidence Networks for Major Bleeding NMA including AVERROES and ACTIVE A studies

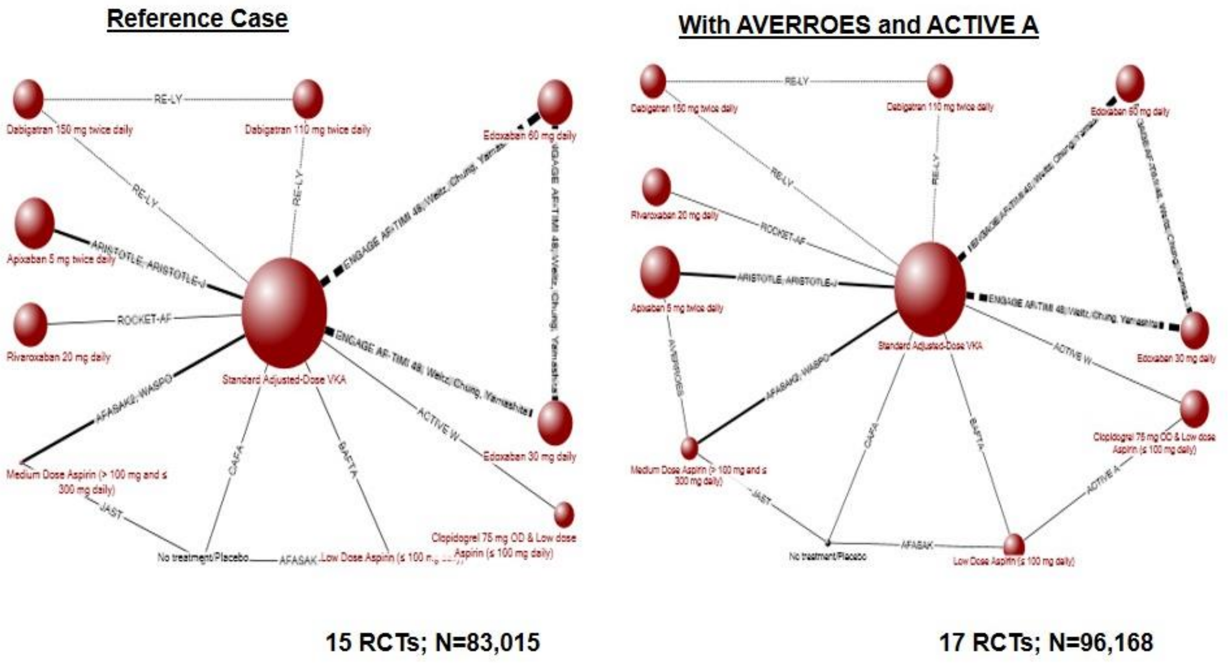

Forest Plot for Major Bleeding NMA including AVERROES and ACTIVE A studies

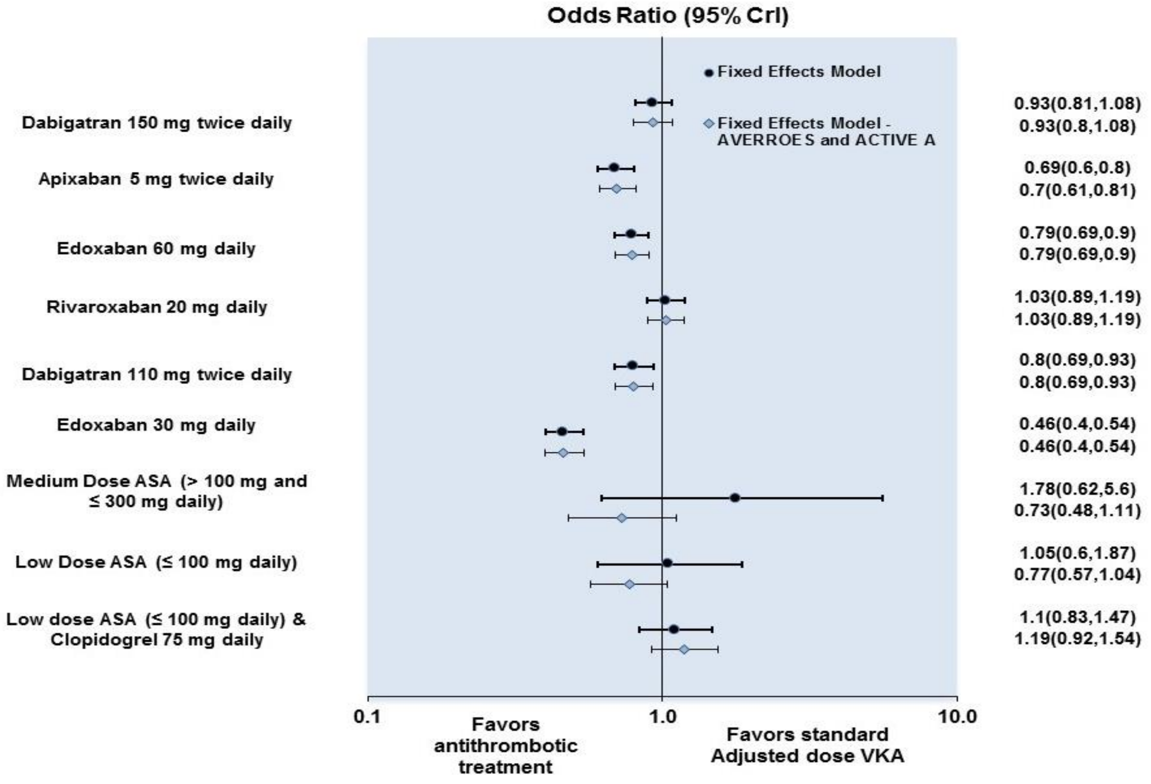

Appendix 13: Comparison with previously published network meta-analyses since Harenberg et al[30]

Author (Publication Year)	Method	Search Date	Included Treatments	Registered Protocol	Relative Measure(s)	Absolute Measure(s) reported	Subgroup Analyses Reported
**Cameron 2014**	Bayesian Network Meta-analysis	January 2014	dabigatran, rivaroxaban, apixaban, edoxaban, and vitamin K antagonists (VKA) at a standard adjusted dose (target international normalised ratio 2.0-3.0), acetylsalicylic acid, acetylsalicylic acid and clopidogrel	Yes (PROSPERO registry - CRD42012002721).	OR (95% CrI)	Yes	Yes
**Ruff 2013**	Traditional Meta-analysis	November 2013	dabigatran, rivaroxaban, apixaban, edoxaban	Not Reported	RR (95%CI)	No	Yes
**Dogliotti 2013**	Bayesian Network Meta-analysis	May 2012	dabigatran, rivaroxaban, apixaban, vitamin K antagonists (VKA) at a standard adjusted dose, acetylsalicylic acid, acetylsalicylic acid and clopidogrel	Yes (metacardio.org)	OR (95% CrI)	No	No
**Assiri 2013**	Baysian Network Meta-analysis	August 2012	dabigatran, rivaroxaban, apixaban, vitamin K antagonists (VKA) at a standard adjusted dose, acetylsalicylic acid, acetylsalicylic acid and clopidogrel	Not Reported	RR (95%CrI)	No	No
**Bruins 2013**	Traditional Meta-analysis	June 2013	antistasin, apixaban, betrixaban, darexaban, DU176b, edoxaban, eribaxaban, fondaparinux, idraparinux, otamixaban, razaxaban, rivaroxaban, yagin, YM150, LY517717, SSR126517E, vitamin K antagonists (warfarin and congeners) with the intensity of anticoagulation dose-adjusted using the International Normalised Ratio (INR)	Yes (Cochrane Library)	OR (95%CI) RR (95%CI) in summary table	Yes	Yes

Appendix 14: Excluded Study List

Inappropriate Study Design

Cardiovasc J S Afr. 2006 Mar;17(2):91.
J Fam Pract. 2006;55(9):753.
Cardiovasc J Afr. 2009 May;20(3):210.
Cardiovasc J Afr. 2009 Sep;20(5):311-2.
Australian Journal of Pharmacy. 2009;90(1073):68.
Formulary. 2010;45(1):16.
Aalbers J. Cardiovasc J Afr. 2010 Nov;21(6):342-3.
Alexander W, et al. PT. 2010;35(10):580-1.
Belavic JM. Nurse Pract. 2011 Sep;36(9):6-7.
Camm AJ. Eur Heart J. 2009 Nov;30(21):2554-5.
Chua D, et al. N Engl J Med. 2009;361(13):1312-3.
Connolly SJ. Eur J Heart Fail. 2009;11(12):1215.
DeSilvey DL. Am J Geriatr Cardiol. 2006 Sep;15(5):326-7.
Dunn A. Ann Intern Med. 2011;154(8):JC4-3.
Eikelboom JW, et al. J Thromb Haemost. 2012;10(5):966-8.
Ezekowitz MD, et al. J Interv Card Electrophysiol. 2011 Dec;32(3):173-80.
Garcia D, et al. Lancet. 2007 Aug 11;370(9586):460-1.
Granger CB. Ann Intern Med. 2012;156(JC1-2):JC1-3.
Hart RG, et al. Ann Intern Med. 2007;147(8):590-2.
Katona A, et al. Eur Heart J. 2006 Jun;27(11):1382.
Kita K, et al. Interventional Cardiology. 2011;3(6):637.
Lopes RD, et al. Evid Based Med. 2011;16(6):187-8.
Nolan J, et al. Br Heart J. 1992 Dec;68(6):544-7.
Paikin JS, et al. Expert Rev Cardiovasc Ther. 2011 Mar;9(3):279-86.
Panichpisal K, et al. Future Neurology. 2011;6(2):155-8.
Roy B, et al. Am J Cardiol. 2012 Feb 1;109(3):370-7.
Sloan MA. Evid Based Med. 2006;11(6):170.
Vassiliou VS. N Engl J Med. 2012;366(1):88-90.
Veloso HH, et al. Chest. 2005 Jul;128(1):475.
Weinberger J. Curr Cardiol Rep. 2010;12(1):1-2.

Inappropriate Intervention

Lancet. 1994 Mar 19;343(8899):687-91.
The Stroke Prevention in Atrial Fibrillation Investigators. Arch Intern Med. 1996 Feb 26;156(4):409-16.
Archer SL, et al. Am Heart J. 1995 Aug;130(2):287-95.
Edvardsson N, et al. J Intern Med. 2003 Jul;254(1):95-101.
Hart RG, et al. Cerebrovasc Dis. 2000 Jan;10(1):39-43.
Hellemons BS, et al. Control Clin Trials. 1999 Aug;20(4):386-93.
Hori M, et al. Circ J. 2012 Jun 5.
Miller VT, et al. Neurology. 1996 Jan;46(1):238-40.
Perez-Gomez F, et al. Eur Heart J. 2007;28(8):996-1003.

For peer review only

Singer DE, et al. Am Heart J. 1992 Dec;124(6):1567-73.
Connolly S, et al. Eur Heart J. 2013 May:34(20):1498-505

Inappropriate Population

ACTIVE Investigators, et al. N Engl J Med 2009;360(20):2066-78.
Circulation 1991;84(2):527-39.
Connolly SJ, et al. N Engl J Med 2011;364(9):806-17.
Dewilde W, et al. Am Heart J. 2009 Nov;158(5):713-8.
Diener HC, et al. Lancet Neurol. 2012 Mar;11(3):225-31.
EAFT (European Atrial Fibrillation Trial) Study Group. Lancet. 1993;342(8882):1255-62.
Eikelboom JW, et al. J Stroke Cerebrovasc Dis. 2012 Aug;21(6):429-35.
ESPRIT Study Group, et al. Lancet Neurol. 2007 Feb;6(2):115-24.
Hart RG, et al. Cerebrovasc Dis. 2008;25(4):344-7.
N Engl J Med 1990;322(12):863-8.
Wang TH, et al. Eur Heart J. 2007 Sep;28(18):2200-7.

Inappropriate Outcomes

De Caterina R, et al. Eur Heart J. 2010 Sep;31(17):2133-40.
Ezekowitz MD, et al. Circulation. 1995 Oct 15;92(8):2178-82.
Lorenzoni R, et al. Am Heart J. 2004 Jul;148(1):e6.

Others

Cardiovasc J Afr. 2011 May;22(3):164.
Active Steering Committee, et al. Am Heart J. 2006 Jun;151(6):1187-93.
ROCKET AF Study Investigators. Am Heart J. 2010 Mar;159(3):340-7.
The Stroke Prevention in Atrial Fibrillation Investigators. Stroke. 1990 Apr;21(4):538-45.
Boysen G, et al. Stroke. 1990;21(Suppl I):I-92.
De Schryver EL. Cerebrovasc Dis. 2000 Mar;10(2):147-50.
Desai A. Clinical Trials Registry - India (CTRI). 2006.
Eikelboom JW, et al. Am Heart J. 2010 Mar;159(3):348-53.
Ezekowitz MD, et al. Am Heart J. 2009 May;157(5):805-10, 810.e1-2.
Flaker G, et al. J Am Coll Cardiol. 2012 Feb 28;59(9):854-5.
Lopes RD, et al. Lancet. 2012 Oct 2. Epub ahead of print.
Lopes RD, et al. Am Heart J. 2010 Mar;159(3):331-9.
Mant J, et al. Cerebrovasc Dis. 2007;23(Suppl 2):10.
Mant J, et al. Cardiol Rev. 2008;25(7):32-6.
Mant JW, et al. BMC Cardiovasc Disord. 2003 Aug 26;3:9.
Posada IS, et al. Am Heart J. 1999 Jul;138(1 Pt 1):137-43.
Van Latum JC. Ned Tijdschr Geneeskd. 1994;138(20):1025-31.
Yigit Z. Turk Kardiyoloji Dernegi Arsivi. 2000;28(1):8-19+4.

For peer review only

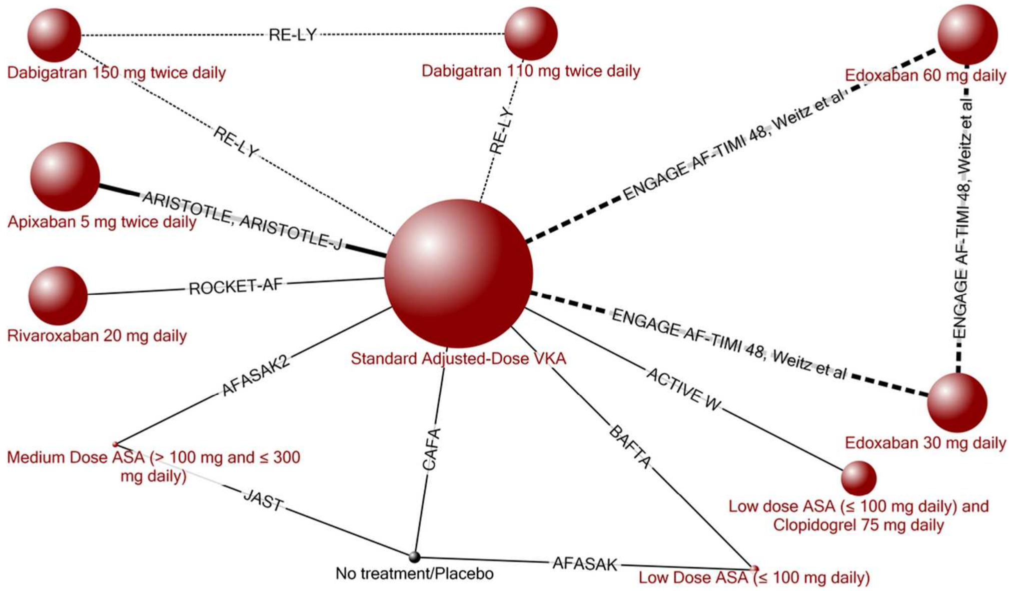

Figure 1: Evidence network for all cause stroke or systemic embolism.* The width of the lines is proportional to the number of randomized controlled trials comparing each pair of treatments, and the size of each treatment node is proportional to the number of randomised participants (sample size). A dotted line indicates a three-arm randomized controlled trial and a black node indicates a node included in the analysis but not reported in the main text.
101x60mm (300 x 300 DPI)

For peer review only

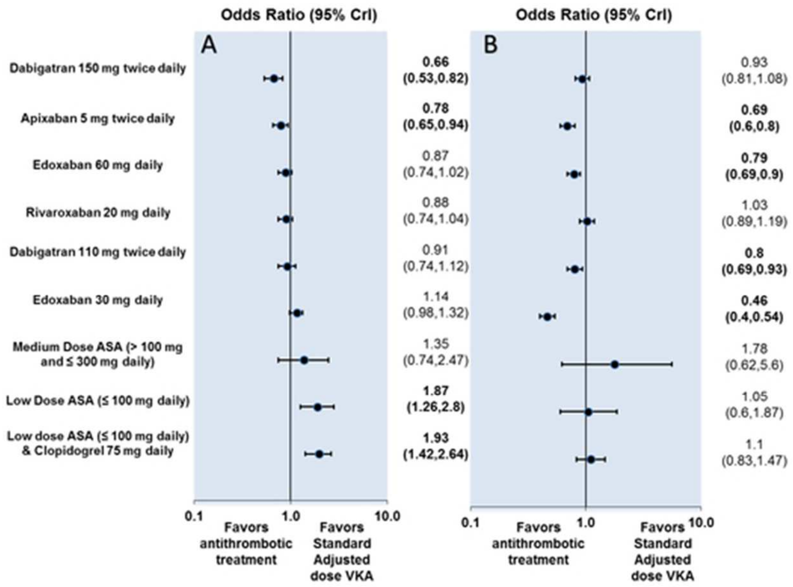

Figure 2: Odds ratio for all cause stroke or systemic embolism (A) and major bleeding (B) in Bayesian network meta-analysis versus standard adjusted dose VKA.
28x16mm (600 x 600 DPI)

For peer review only - http://bmjopen.bmj.com/site/about/guidelines.xhtml

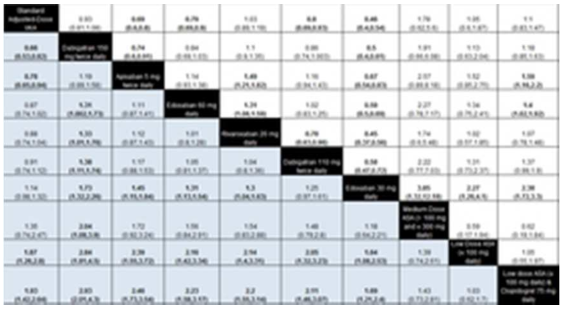

Figure 3: Odds Ratios (OR) from network meta-analyses for stroke or systemic embolism and major bleeding for all pair-wise comparisons.* ORs for recurrence of stroke or systemic embolism are below the diagonal (row-defining treatment versus column-defining treatment) and those for major bleeding are above the diagonal and in blue (column-defining treatment versus row-defining treatment). To obtain ORs for comparisons in the opposite direction, reciprocals should be taken (e.g., the OR for standard dose warfarin compared with apixaban 5 mg twice daily for stroke or systemic embolism is 1/0.78=1.28). Significant results are in bold and underscored (and in larger font).
23x12mm (300 x 300 DPI)

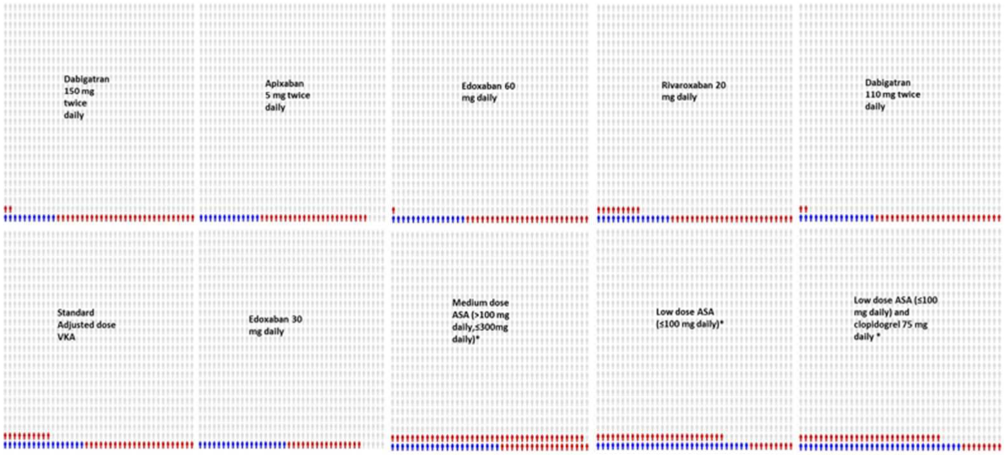

Figure 4: Icon array illustrating the absolute risks of Stroke or systemic embolism (blue) and major bleeding episodes (red) per 1000 patients treated*
66x30mm (300 x 300 DPI)

PRISMA Checklist

Section/topic	#	Checklist item	Reported on page #
**TITLE**			
Title	1	Identify the report as a systematic review, meta-analysis, or both.	0
**ABSTRACT**			
Structured summary	2	Provide a structured summary including, as applicable: background; objectives; data sources; study eligibility criteria, participants, and interventions; study appraisal and synthesis methods; results; limitations; conclusions and implications of key findings; systematic review registration number.	0
**INTRODUCTION**			
Rationale	3	Describe the rationale for the review in the context of what is already known.	1
Objectives	4	Provide an explicit statement of questions being addressed with reference to participants, interventions, comparisons, outcomes, and study design (PICOS).	1
**METHODS**			
Protocol and registration	5	Indicate if a review protocol exists, if and where it can be accessed (e.g., Web address), and, if available, provide registration information including registration number.	2
Eligibility criteria	6	Specify study characteristics (e.g., PICOS, length of follow-up) and report characteristics (e.g., years considered, language, publication status) used as criteria for eligibility, giving rationale.	2-3
Information sources	7	Describe all information sources (e.g., databases with dates of coverage, contact with study authors to identify additional studies) in the search and date last searched.	2-3
Search	8	Present full electronic search strategy for at least one database, including any limits used, such that it could be repeated.	2-3
Study selection	9	State the process for selecting studies (i.e., screening, eligibility, included in systematic review, and, if applicable, included in the meta-analysis).	3
Data collection process	10	Describe method of data extraction from reports (e.g., piloted forms, independently, in duplicate) and any processes for obtaining and confirming data from investigators.	3
Data items	11	List and define all variables for which data were sought (e.g., PICOS, funding sources) and any assumptions and simplifications made.	3-4
Risk of bias in individual studies	12	Describe methods used for assessing risk of bias of individual studies (including specification of whether this was done at the study or outcome level), and how this information is to be used in any data synthesis.	3,6
Summary measures	13	State the principal summary measures (e.g., risk ratio, difference in means).	3
Synthesis of results	14	Describe the methods of handling data and combining results of studies, if done, including measures of consistency (e.g., I^2) for each meta-analysis.	4-5
Risk of bias across studies	15	Specify any assessment of risk of bias that may affect the cumulative evidence (e.g., publication bias, selective reporting within studies).	5
Additional analyses	16	Describe methods of additional analyses (e.g., sensitivity or subgroup analyses, meta-regression), if done, indicating which were pre-specified.	5
**RESULTS**			

For peer review only

Study selection	17	Give numbers of studies screened, assessed for eligibility, and included in the review, with reasons for exclusions at each stage, ideally with a flow diagram.	6, Online Appendix 2
Study characteristics	18	For each study, present characteristics for which data were extracted (e.g., study size, PICOS, follow-up period) and provide the citations.	5-6, Online Appendix 3
Risk of bias within studies	19	Present data on risk of bias of each study and, if available, any outcome level assessment (see item 12).	7
Results of individual studies	20	For all outcomes considered (benefits or harms), present, for each study: (a) simple summary data for each intervention group (b) effect estimates and confidence intervals, ideally with a forest plot.	Figure 2-4
Synthesis of results	21	Present results of each meta-analysis done, including confidence intervals and measures of consistency.	Figure 2-4, Online Appendix 6,7 and 9
Risk of bias across studies	22	Present results of any assessment of risk of bias across studies (see Item 15).	7
Additional analysis	23	Give results of additional analyses, if done (e.g., sensitivity or subgroup analyses, meta-regression [see Item 16]).	Table 1, Online Appendix 12
**DISCUSSION**			
Summary of evidence	24	Summarize the main findings including the strength of evidence for each main outcome; consider their relevance to key groups (e.g., healthcare providers, users, and policy makers).	11-12
Limitations	25	Discuss limitations at study and outcome level (e.g., risk of bias), and at review-level (e.g., incomplete retrieval of identified research, reporting bias).	14-15
Conclusions	26	Provide a general interpretation of the results in the context of other evidence, and implications for future research.	10-16
**FUNDING**			
Funding	27	Describe sources of funding for the systematic review and other support (e.g., supply of data); role of funders for the systematic review.	0

For peer review only
